# Whole-Transcriptome Survey of the Putative ATP-Binding Cassette (ABC) Transporter Family Genes in the Latex-Producing Laticifers of *Hevea brasiliensis*

**DOI:** 10.1371/journal.pone.0116857

**Published:** 2015-01-23

**Authors:** Nie Zhiyi, Kang Guijuan, Li Yu, Dai Longjun, Zeng Rizhong

**Affiliations:** Key Laboratory of Biology and Genetic Resources of Rubber Tree, Ministry of Agriculture & Rubber Research Institute, Chinese Academy of Tropical Agricultural Sciences, Danzhou, Hainan 571737, China; USDA/ARS, UNITED STATES

## Abstract

The ATP-binding cassette (ABC) proteins or transporters constitute a large protein family in plants and are involved in many different cellular functions and processes, including solute transportation, channel regulation and molecular switches, etc. Through transcriptome sequencing, a transcriptome-wide survey and expression analysis of the ABC protein genes were carried out using the laticiferous latex from *Hevea brasiliensis* (rubber tree). A total of 46 putative ABC family proteins were identified in the *H. brasiliensis* latex. These consisted of 12 ‘full-size’, 21 ‘half-size’ and 13 other putative ABC proteins, and all of them showed strong conservation with their *Arabidopsis thaliana* counterparts. This study indicated that all eight plant ABC protein paralog subfamilies were identified in the *H. brasiliensis* latex, of which ABCB, ABCG and ABCI were the most abundant. Real-time quantitative reverse transcription-polymerase chain reaction assays demonstrated that gene expression of several latex ABC proteins was regulated by ethylene, jasmonic acid or bark tapping (a wound stress) stimulation, and that *HbABCB15*, *HbABCB19*, *HbABCD1* and *HbABCG21* responded most significantly of all to the abiotic stresses. The identification and expression analysis of the latex ABC family proteins could facilitate further investigation into their physiological involvement in latex metabolism and rubber biosynthesis by *H. brasiliensis*.

## Introduction

The ATP-binding cassette (ABC) protein family, especially the intrinsic membrane subfamilies, mediates a large number of fundamental cellular functions and processes that utilize ATP hydrolysis to energize the transport of solutes across membranes. These include substrate translocation, lipid trafficking, protein targeting and phytohormone transport [1–5], etc. The plant ABC proteins constitute one of the largest and most diverse protein families, and can be organized phylogenetically into eight clusters, namely the ABCA-ABCI subfamilies (ABCH is not found in plants) [6]. Two major types of ABC proteins can be characterized in plants. One is the membrane-integrated ABC protein, including the ABCA–ABCD and ABCG subfamilies, which contain both a nucleotide binding domain (NBD) and a trans-membrane domain (TMD), and the other is the soluble ABC proteins, which mainly cluster in the ABCE, ABCF and ABCI subfamilies and only contain a NBD domain [7, 8]. Whole genome sequencing has led to complete inventories of plant ABC transporters for model plants, such as *Arabidopsis thaliana* [9], rice (*Oryza sativa* L.) [10] and other plant species [11, 12], whereas little is known about ABC proteins in *Hevea brasiliensis* (Willd. *ex* Adr. de Juss.) Muell.-Arg. (para rubber tree), which is the most important natural rubber (NR)-producing plant in the world.

NR is a long chain *cis*-1,4-polyisoprene polymer and is a strategically indispensable industrial raw material for more than 40,000 products. *H. brasiliensis*, a member of the Euphorbiaceae, is almost the only species that can produce commercially viable quantities of high-quality NR [13]. NR synthesis is a typical plant secondary metabolism process that occurs in the latex vessels of rubber trees via glycolysis, followed by the mevalonate or 2-C-methyl-D-erythritol 4-phosphate pathways, which provide the direct precursor of isopentenyl diphosphate (IPP) [14]. On the rubber particle, NR is formed through sequential condensation of hundreds of thousands of IPP units and is finally compartmentalised within a special organelle that is suspended in the latex of the laticiferous cells of rubber trees [15–17]. The increasing demand for NR by the world economy has prompted investigations into the underlying molecular mechanisms behind latex metabolism and NR biosynthesis [18–20], which may lead to an improvement in the latex yield of rubber trees.

One of the latex yield-limiting factors is the regeneration potential of the latex in the *H. brasiliensis* laticifers between two consecutive tappings. Latex regeneration consists of several biological and molecular events, including sucrose importation into laticifers from neighboring cells [21, 22], ontogenesis, the development of rubber particles derived from endoplasmic reticulum [23] and the assembly and breakdown of complex lipids in the latex [24], etc. All the biological processes involve substrate transportation and trafficking or the turnover of intracellular components. Much less is known about the related process of solute transportation that occurs within the highly specialized and mature laticifer system, which has no plasmodesmata and is therefore apoplastically isolated from the adjacent cells in the inner bark of *H. brasiliensis* [25].

A hallmark biological feature of rubber trees is their reticulate network of laticiferous cells, within which NR biosynthesis is carried out. The latex collected through bark tapping is actually the pure cytoplasm of the laticiferous vessels [26]. Therefore, the latex, together with the laticiferous cells of *H. brasiliensis*, is a good experimental system for biochemical and molecular investigations into a particular type of cell that has specially evolved for NR biosynthesis. Characterization of the ABC transporters in the laticifers would help reveal the physiological and molecular processes underlying latex metabolism and NR biosynthesis in rubber trees. Recently, 270 ABC transporter gene contigs were identified from the bark transcriptome of rubber trees [27], but no more information is available about the ABC transporters in rubber trees. In this study, the global inventory of the ABC proteins in the laticifers of *H. brasiliensis* was first identified by sequencing the latex transcriptome and then the expression patterns of several ABC proteins genes were analyzed using real-time quantitative reverse transcript-polymerase chain reaction (RT-qPCR). This research provides valuable information for further investigations into the physiological roles of the ABC transporters during latex metabolism and NR biosynthesis in rubber trees.

## Materials and Methods

### Latex Collection and Total Latex RNA Preparation for Transcriptome Sequencing

Rubber trees (Clone Reyan 7–33–97) were planted on the experimental farm at the Chinese Academy of Tropical Agricultural Sciences in Hainan, P.R. China. Trees that had homogeneous stem girths and had been tapped for 2 years using an S/2 d/3 system (tapped every 3 days in a half spiral), were selected. The fresh latex sample was collected in a thermo bottle containing liquid nitrogen for 10 min and then immediately stored at—80°C. Total RNAs were isolated from the collected latex using our previously described method [28] and treated with DNase I (Invitrogen, Carlsbad, CA, USA) to remove genomic DNA. The quality and integrity of the RNAs were evaluated using Nanodrop 2000 (Thermo Scientific, Wilmington, DE, USA) and Bioanalyzer Chip RNA 7500 series II (Agilent, Santa Clara, USA) instruments.

### Construction of the cDNA Library and Transcriptome Sequencing

Sequencing of the latex transcriptome was performed using an Illumina mRNA-Seq Sample Prep kit (Illumina Inc., San Diego, CA), according to the manufacturer’s instructions. Briefly, poly (A) mRNA was isolated from total RNA using oligo (dT) magnetic beads and fragmented into small pieces using divalent cations at an elevated temperature. The cleaved mRNA fragments were used for the first strand cDNA synthesis, together with reverse transcriptase and random hexamer-primers. The second strand cDNAs were synthesized using RNaseH and DNA polymerase I. The double-stranded cDNA fragments were purified with a QiaQuick PCR extraction kit and eluted with EB buffer for end repair and the ligation of sequencing adapters. The products were purified by agarose gel electrophoresis and the fragments that were around 200 bp in length were selected as templates and enriched by linear PCR amplification, which created the final cDNA library for transcriptome paired end sequencing via the Illumina HiSeq-2000 sequencing platform. Image analysis, base calling and quality value calculations were conducted using the Illumina/Solexa data processing pipeline.

### *De novo* Assembly of the Latex Transcriptome

Before assembly, the raw reads that contained adaptors or unknown nucleotides larger than 5% with quality scores of Q ≤ 10 were trimmed using software found in Filter-fq (BGI, Shenzhen, China). The clean reads, which were randomly split into 25 bp K-mers for assembly into contigs using the de Bruijn graph, were then used for transcriptome *de novo* assembly using the Trinity software short reads assembly program (Release-20130225) [29]. The resultant contigs were further joined into scaffolds using the paired-end reads and gap filling was carried out in order to complete the scaffolds. To obtain non-redundant unigenes, the scaffolds were assembled together and clustered using the Gene Indices Clustering Tools (TGICL, version 2.1) [30] and Phrap software (Release-23.0) [31], which finally produced the assembled consensus sequences of the *H. brasiliensis* latex transcriptome. The transcriptome data have been submitted to the SRA database with an accession number of SRR1648124.

### Functional Annotation and Classification of the Latex Transcriptome

All the assembled unigenes were used in a BLAST search and for annotation against the NCBI nr database with an E-value cut-off of 10^-5^. Functional categorization by Gene Ontology (GO; http://www.geneontology.org) was performed using Blast2GO software (http://www.blast2go.de/) with an E-value threshold of 10^-5^. Analyses of the Cluster of Orthologous Groups (COG) identifications and the Kyoto Encyclopedia of Genes and Genomes (KEGG) pathway annotations were conducted using Blastall software (http://www.ncbi.nlm.nih.gov/staff/tao/URLAPI/blastall/) against the COG database in the NCBI (COG; ftp://ftp.ncbi.nih.gov/pub/wolf/COGs/) and the KEGG database using the BLASX algorithm with an E-value threshold of 10^-5^.

### Identification of ABC Protein Genes in the Latex Transcriptome

A reciprocal best hits (two-direction) blast search was performed to identify the latex ABC protein genes. First, *A. thaliana* ABC protein sequences were retrieved from the *A. thaliana* Information Resource (TAIR) database. Putative ABC proteins obtained from the *H. brasiliensis* latex were searched by performing a tBLASTN analysis (http://www.ncbi.nlm.nih.gov/blast) against the generated latex transcriptome data using *A. thaliana* ABC protein sequences as queries. Second, the best hits were blasted again against the entire *A. thaliana* TAIR10 transcripts (http://www.arabidopsis.org/Blast/index.jsp). Non-complete latex ABC protein genes were manually assembled into complete unigenes using the CAP3 program (http://doua.prabi.fr/software/cap3). The open reading frames (ORFs) of the latex ABC protein unigenes were verified by searching the *H. brasiliensis* genome DNA sequences that have just been sequenced and assembled by the Rubber Research Institute of China using the Vector NTI Advance program (version 11.5.2). The polypeptide sequences corresponding to the latex ABC proteins were analyzed for the presence of ABC conserved domains using the Conserved Domain Database (CDD) at NCBI (http://www.ncbi.nlm.nih.gov/Structure/cdd/wrpsb.cgi) and the Pfam27.0 web server (http://pfam.xfam.org/) [32, 33]. The cDNA sequences of all the latex ABC protein genes have been deposited in the NCBI database with accession numbers ranging from KM035282–035324.

### Sequence Analysis and Phylogenetics

The deduced amino acid sequences of the latex ABC proteins, together with those of the *A. thaliana* ABC proteins, were aligned by a multiple sequence comparison using the log-expectation (MUSCLE) alignment tool (http://www.ebi.ac.uk/Tools/msa/muscle) with the default program options [34]. Then they were subjected to phylogenetic analysis using the neighbor-joining method and 1000 bootstrap replicates were employed in each analysis to maximize the statistical significance [35]. The phylogenetic trees were constructed and visualized by MEGA5.05 software [36].

### Treatment of Rubber Trees Used for Gene Expression Analysis

Field experiments were performed using mature, 7-year-old virgin rubber trees (Clone Reyan 7–33–97) that had never been tapped. Stimulation assays with exogenous methyl jasmonate (Me-JA) or Ethrel (an ethylene releaser) were carried out according to the method described in a previous paper [28]. Briefly, 0.3% (w/w) Me-JA (Sigma–Aldrich, USA) in lanoline or 0.5% (w/w) Ethrel (Sigma–Aldrich, USA) in water was applied to the bark below the half spiral of the tapping cut. Lanoline or water was used as the mock stimulation for the control samples. The trunks of the treated trees were wrapped with black plastic film around the tapping cut and stimulated for 0, 0.5, 1.5, 4.0, 8.0 or 24.0 h, after which they were tapped for latex early in the morning on the same day. For the tapping assays, the rubber trees were sequentially tapped seven times using an S/2 d/3 system and fresh latex was collected at each tapping time for analysis. Each sample included three independent biological replicates, and the latex collected from six rubber trees was pooled to make one biological replicate for each sample.

### Transcript Abundance Analysis of Latex ABC Protein Genes by RT-qPCR

Total RNA from *H. brasiliensis* latex was isolated using an RNeasy Plant Mini-Kit (Qiagen, Valencia, CA) according to the manufacturer’s instructions. Reverse transcription was performed with SuperScript III reverse transcriptase (Invitrogen, CA), followed by incubation with RNase H (Invitrogen, CA). RT-qPCR analysis was performed using the method described by Duan et al. [37]. Primers were designed for the selected genes by DNAMAN 7.02 software with a PCR product of ~200 bp. The forward and reverse primer sequences used to detect each mRNA and their efficiencies are shown in S1 Table. The cDNAs were synthesized by reverse transcriptase and quantitative gene expression analysis was carried out by RT-qPCR using a LightCycler 2.0 (Roche, Basel, Switzerland) and the following parameters: 30 s at 94°C for denaturation, followed by 45 cycles of 94°C for 5 s, 60°C for 20 s and 72°C for 20 s. Each RT-qPCR reaction was replicated three times. For normalization purposes, the *H. brasiliensis* 18S rRNA gene was taken as the internal reference for all RT-qPCR analyses.

## Results/Discussion

### Sequencing and *de novo* Assembly of the *H. brasiliensis* Latex Transcriptome

The *H. brasiliensis* latex transcriptome was generated using the latex collected from rubber trees that had been regularly tapped for 2 years in an S/2 d/3 system. These rubber trees had a sustainable and higher latex yield than newly tapped trees due to their regular bark tapping, which represents a mechanical stress for the rubber tree. Latex production is a natural response to mechanical wounding and studies have reported that it activates latex metabolism, which then leads to an increase in latex production [38]. The complicated biological process leading to latex production involves several biochemical reactions that occur in the laticifers and many genes or proteins may contribute to the final results. The complex biological mechanism underlying latex metabolism requires a global transcriptic survey to improve understanding of the process. Therefore, the latex transcriptome was first sequenced using the Illumina HiSeq-2000 platform. Approximately 6.17 Gb of total nucleotides were obtained, and the 6.85 Mb clean reads were assembled into 182,956 contigs and 60,909 unigenes, respectively.

### Identification of ABC Proteins in the *H. brasiliensis* Latex Transcriptome

A reciprocal best hits (two-direction) blast search was performed in order to identify the orthologs of the ABC protein genes in the *H. brasiliensis* latex transcriptome. The results demonstrated that the identified latex ABC protein genes from the two blasts closely matched each other (data available in S2 Table). A total of 46 putative latex ABC protein genes were finally identified via the reciprocal search approach. The details can be found in Table 1, but only the results obtained by using the *A. thaliana* ABC protein sequences as queries are shown. The complete ORFs of the 46 latex ABC proteins were further verified by searching the *H. brasiliensis* genome DNA sequences that have recently been completed by the Rubber Research Institute of China. The genome DNA sequences for each latex ABC protein gene are shown S3 Table, which also shows the corresponding exons and introns for each latex ABC protein gene. Aligning all the transcripts of the 46 latex ABC proteins to the *H. brasiliensis* genome did not reveal any alternative transcripts from the same locus, since each transcript of the 46 ABC proteins was definitively located on different scaffolds of the *H. brasiliensis* genome, as shown in S3 Table.

**Table 1:** Detailed inventory of the 46 ABC transporter genes identified in the *H. brasiliensis* latex.

**ABC subfamily**		**Gene name**	**NCBI accession no.**	**Length (aa)**	**Topology**	***A. thaliana* homolog**	**Common name**	**Identity (%)**
Subfamily A (ABCA*)	AOH; ABCA	HbABCA1	KM035282	1883	(TMD-NBD)_2_	At2g41700	AtABCA1	74
	ATH; ABCA	HbABCA2	KM035283	970	TMD-NBD	At3g47730	AtABCA2	71
		HbABCA7	KM035284	939	TMD-NBD	At3g47780	AtABCA7	67
Subfamily B (ABCB)	MDR; DPL(PGP)	HbABCB1	KM035285	1363	(TMD-NBD)_2_	At2g36910	AtABCB1	88
		HbABCB11	KM035286	1283	(TMD-NBD)_2_	At1g02520	AtABCB11	74
		HbABCB13	KM035287	1135	(TMD-NBD)_2_	At1g27940	AtABCB13	71
		HbABCB15	HQ917533	1250	(TMD-NBD)_2_	At3g28345	AtABCB15	77
		HbABCB19	KM035288	1259	(TMD-NBD)_2_	At3g28860	AtABCB19	89
		HbABCB20	KM035289	1404	(TMD-NBD)_2_	At3g55320	AtABCB20	86
	ATM; DPL(HMT)	HbABCB25	KM035290	739	TMD-NBD	At5g58270	AtABCB25	83
	TAP; DPL(TAP)	HbABCB26	KM035291	702	TMD-NBD	At1g70610	AtABCB26	75
		HbABCB28	KM035292	660	TMD-NBD	At4g25450	AtABCB28	70
	DPL(LLP)	HbABCB29	KM035293	650	TMD-NBD	At5g03910	AtABCB29	65
Subfamily C (ABCC)	MRP; OAD(MRP)	HbABCC2	KM035294	1624	(TMD-NBD)_2_	At2g34660	AtABCC2	79
		HbABCC5	KM035295	1499	(TMD-NBD)_2_	At1g04120	AtABCC5	81
		HbABCC13	KM035296	1480	(TMD-NBD)_2_	At2g07680	AtABCC13	66
Subfamily D (ABCD)	PMP; FAE	HbABCD1	KF701641	1337	(TMD-NBD)_2_	At4g39850	AtABCD1	78
		HbABCD2	KM035297	746	TMD-NBD	At1g54350	AtABCD2	69
Subfamily E (ABCE)	RLI	HbABCE2	KM035298	605	NBD-NBD	At4g19210	AtABCE2	93
Subfamily F (ABCF)	GCN; ART(REG)	HbABCF1	JX109943	605	NBD-NBD	At5g60790	AtABCF1	85
		HbABCF3	KM035299	715	NBD-NBD	At1g64550	AtABCF3	82
		HbABCF4	KM035300	726	NBD-NBD	At3g54540	AtABCF4	79
		HbABCF5	KM035301	709	NBD-NBD	At5g64840	AtABCF5	79
Subfamily G (ABCG)	WBC; EPD(WHITE)	HbABCG3	KM035302	723	NBD-TMD	At2g28070	AtABCG3	81
		HbABCG5	KM035303	626	NBD-TMD	At2g13610	AtABCG5	73
		HbABCG7	KM035304	720	NBD-TMD	At2g01320	AtABCG7	78
		HbABCG11	KM035305	649	NBD-TMD	At1g17840	AtABCG11	53
		HbABCG15	KM035306	714	NBD-TMD	At3g21090	AtABCG15	68
		HbABCG20	KM035307	715	NBD-TMD	At3g53510	AtABCG20	75
		HbABCG21	KM035308	680	NBD-TMD	At3g25620	AtABCG21	68
		HbABCG22	KM035309	738	NBD-TMD	At5g06530	AtABCG22	78
		HbABCG28	KM035310	980	NBD-TMD	At5g60740	AtABCG28	68
	PDR; EPD(PDR)	HbABCG40	KM035311	1438	(NBD-TMD)_2_	At1g15520	AtABCG40	72
Subfamily I (ABCI)	NAP; CCM	HbABCI1	KM035312	229	NBD	At1g63270	AtABCI1	86
	NAP; ISB	HbABCI6	KM035313	335	NBD	At3g10670	AtABCI6	82
		HbABCI7	KM035314	492	CYT	At1g32500	AtABCI7	62
		HbABCI8	KM035315	553	CYT	At4g04770	AtABCI8	81
	NAP; CBY(Y179)	HbABCI10	KM035316	261	NBD	At4g33460	AtABCI10	71
		HbABCI11	KM035317	277	NBD	At5g14100	AtABCI11	72
	NAP; MKL; TGD	HbABCI13	KM035318	343	NBD	At1g65410	AtABCI13	80
		HbABCI14	KM035319	325	TMD	At1g19800	AtABCI14	77
		HbABCI15	KM035320	385	SSA	At3g20320	AtABCI15	73
	NAP; NO	HbABCI17	KM035321	283	NBD	At1g67940	AtABCI17	73
		HbABCI18	KM035322	280	NBD	At1g03900	AtABCI18	76
	NAP; NO(ADT)	HbABCI19	KM035323	300	NBD	At1g03905	AtABCI19	80
		HbABCI20	KM035324	329	NBD	At5g02270	AtABCI20	87

*ABCA: Similar to human abca1 protein; NBD: Nucleotide binding domain (ATP binding cassette domain); TMD: transmembrane domain; SSA: substrate binding protein; CYT: conserved soluble protein that interacts with ABC domain; DPL: Drug, peptide and lipid exporters; PGP: Similar to p-glycoprotein; LLP: Lipid A-like exporters, putative; OAD: Organic anion and drug exporters; HMT: Similar to yeast heavy metal transporters; TAP: Similar to the human transporters associated with antigen presentation; WHITE: Similar to Drosophila white protein; CCM: cytochrome C biogenesis family; ISB: iron sulfur center biogenesis family; CBY: family similar to putative cobalt uptake systems; Y179: similar to the *M. janaschii* Y179 protein subfamily; MKL: similar to the *M. leprae* MKL protein family; TGD: trigalactosyldiacyl glycerol: *TGD1*, *2* and *3* are components of a chloroplast phospholipid translocator; NO: proteins of unknown function, apparently unrelated to existing families and ADT: proteins of unknown function.

**Figure 1:**
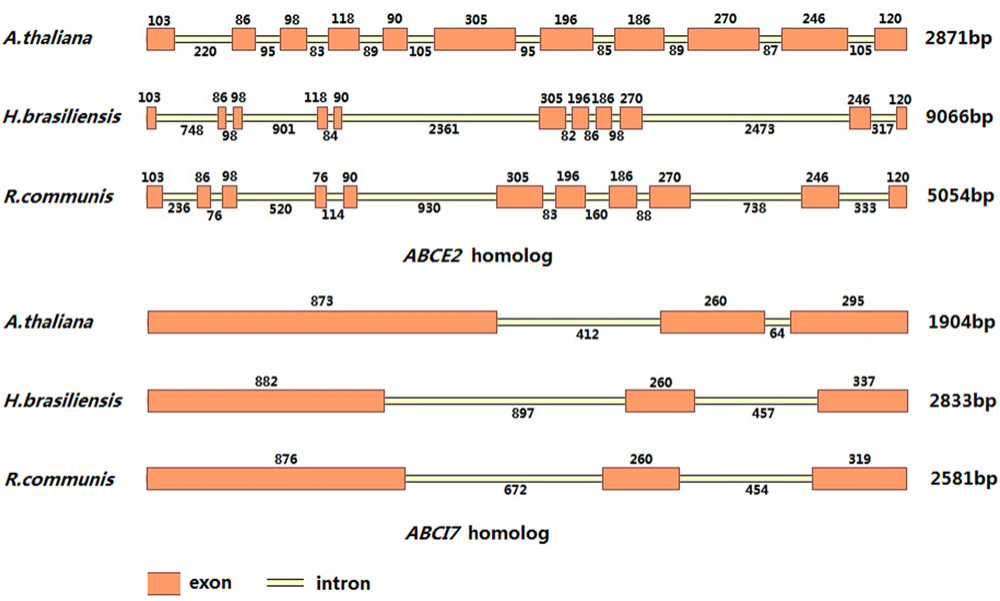
Linear representation of the exon–intron structures of the selected *ABCE2* and *ABCI7* genes in the genome sequences of *A. thaliana*, *H. brasiliensis* and *R. communis*. Exons and introns of the *ABCE2* and *ABCI7* genes were predicted and mapped by the Vector NTI Advance program (version 11.5.2). The accession numbers for the *ABCE2* homolog genome sequences are NC_003075.7 (10501200..10505217) for *A. thaliana* and NW_002994591.1 for *R. communis*. The accession numbers for the *ABCI7* homolog genome sequences are NC_003070.9 (11749823..11752106) for *A. thaliana* and NW_002995814.1 for *R. communis*.

Most subfamilies of plant ABC proteins have corresponding counterparts in the human genome, so that the latex ABC transporters were also denominated using the Human Genome Organization (HUGO) nomenclature for human ABC proteins [39] in order to obtain a unified nomenclature of plant ABC proteins, as suggested by Verrier et al. in 2008 [1]. According to the HUGO nomenclature, all the eight subfamilies of plant ABC transporters were identified in the *H. brasiliensis* latex, among which ABCB, ABCG and ABCI were the most abundant (Table 1). By comparing the mapped reads (reads per kilobase of exon model per million mapped reads), the top five latex ABC transporters were *HbABCF1*, *HbABCG21*, *HbABCI18*, *HbABCB15* and *HbABCG11*. One representative linear comparison of the exon–intron structures of two selected *ABCE2* and *ABCI7* genes in the genome sequences of *A. thaliana*, *H. brasiliensis* and *Ricinus communis* is shown in Fig. 1. The alignment analysis revealed that the *H. brasiliensis* latex ABC protein genes were highly conserved compared with those of a closely related species, *R. communis*, and a distantly related species, *A. thaliana*.

Of the 46 latex ABC transporters, 12 members were full-size transporters, containing two TMDs and two NBDs, 21 members were half-size transporters and contained one TMD and one NBD or two NBDs, and the other 13 members could be called quarter-size ABC molecules with only one NBD or TMD domain. All 12 full-size transporters, with the exception of one protein (HbABCG40), exhibited a forward topology orientation in which the TMDs preceded the NBDs, but most of the 21 half-size transporters had a reverse orientation where NBD preceded TMD. The cDNA sequences of the 46 latex putative ABC transporter genes were submitted to GenBank. Their accession numbers are shown in Table 1 and the predicted amino acid sequences of the putative ABC proteins are listed in S4 Table.

### Phylogeny of the *H. brasiliensis* Latex ABC Proteins

**Figure 2:**
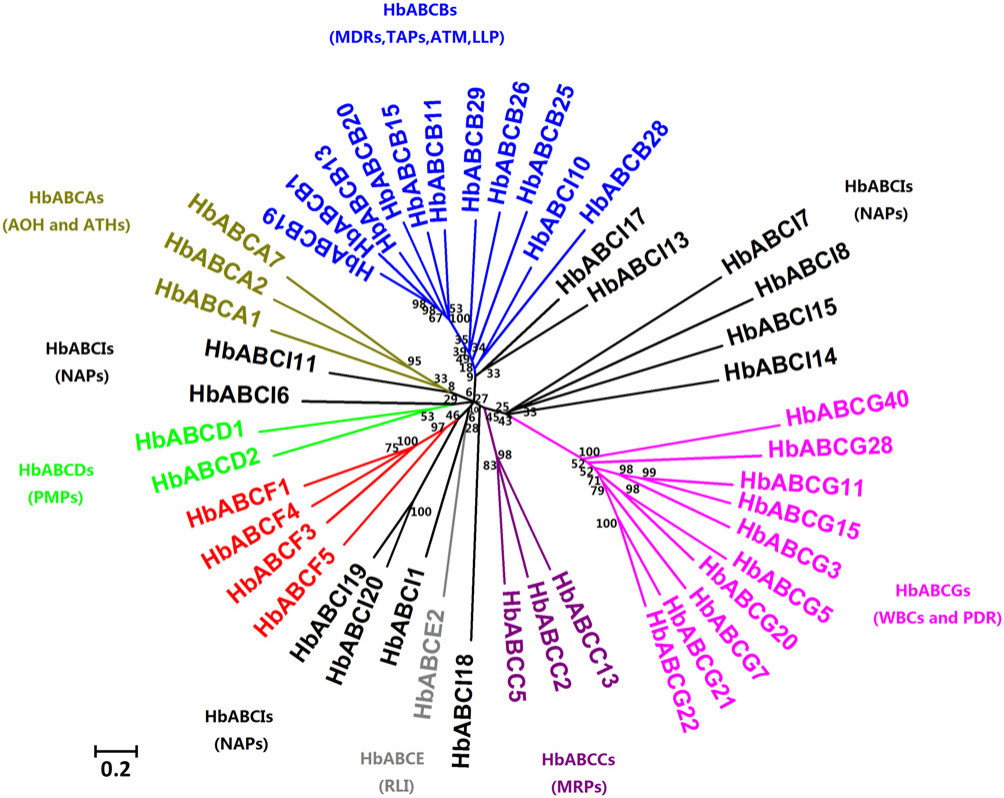
Phylogenetic tree for *H. brasiliensis* latex ABC proteins. The amino acid sequences of the 46 latex ABC proteins were aligned using the MUSCLE program and subjected to phylogenetic analysis by the distance with neighbor-joining method using the MEGA5.05 software. The numbers on the nodes indicate the bootstrap values after 1000 replicates. The scale bar indicates the estimated number of amino acid substitutions per site. The HUGO nomenclature was followed and the ABC protein abbreviations are as follows: AOH: ABC1 homolog; ATH: ABC-two homolog; MDR: multi-drug resistance; ATM: ABC transporter of mitochondria; TAP: transporter associated with antigen processing; LLP: Lipid A-like exporter; MRP: multi-drug resistance-associated protein; PMP: peroxisomal membrane protein; RLI: RNase L inhibitor; GCN: general control non-repressible; WBC: white-brown complex; PDR: pleiotropic drug resistance; NAP: non-intrinsic ABC protein. AOH and ATH, which belong to the ABCA subfamily; MDR, TAP, ATM and LLP, which belong to the ABCB subfamily; MRP, which belongs to the ABCC subfamily; PMP, which belongs to the ABCD subfamily; RLI, which belongs to the ABCE subfamily; GCN, which belongs to the ABCF subfamily; WBC, which belongs to the ABCG subfamily and NAP, which belongs to the ABCI subfamily.

The amino acid sequences of the 46 latex putative ABC transporters were aligned using the MUSCLE alignment tool and the phylogenetic tree was generated by MEGA5.05 software as outlined in the Materials and Methods section. The phylogenetic tree for all 46 latex ABC proteins is shown in Fig. 2. With the exceptions of the ABCI and ABCE subfamilies, which only have one HbABCE2 member that was grouped on its own, the other six subfamilies of the latex ABC proteins were clustered tightly within their respective subfamilies with bootstrap values of at least 90%. The HbABCI subfamily had 13 members and they were clustered into four different clades. HbABCI7, 8, 15, 17 and 18 were grouped within the same clade, whereas HbABCI1, 6 and 11 formed their own clade and HbABCI10, 13 and 14 were in the same clade. HbABCI19 and 20 were clustered together and were closely related to the HbABCG subfamily. The lack of a coherent phylogeny within the HbABCI subfamily was consistent with the clustering patterns of the ABCI subfamilies in *A. thaliana* [2] and *Vitis vinifera* [5]. Of the 46 latex ABC proteins, 42, excluding four HbABCI members (HbABCI14, 15, 17 and 18), contained at least one NBD. The NBD phylogeny of the 42 latex ABC transporters is shown in S1 Fig.

### Characteristics of the *H. brasiliensis* Latex ABC Family Transporters

**Figure 3:**
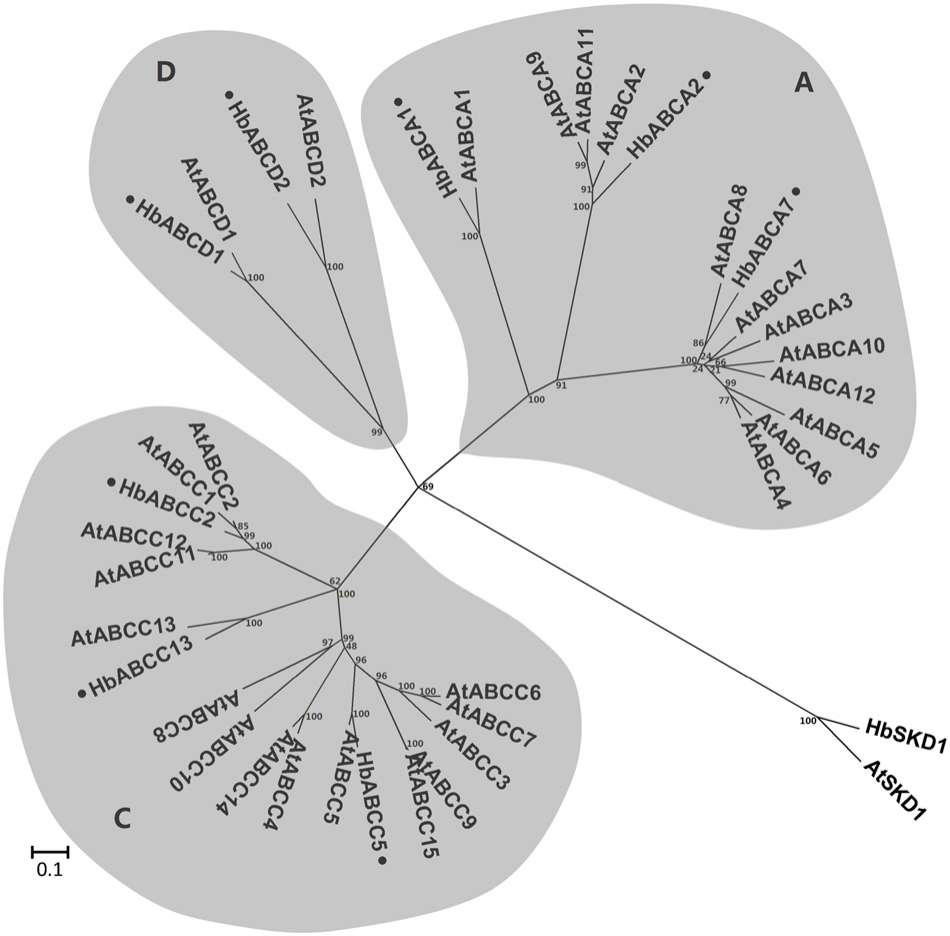
Phylogenetic relationship between *A. thaliana* and *H. brasiliensis* latex ABCA, ABCC and ABCD proteins. The amino acid sequences of all the *A. thaliana* ABCA, ABCC, ABCD and *H. brasiliensis* latex proteins were aligned using the MUSCLE program and subjected to phylogenetic analysis by the distance with neighbor-joining method using MEGA5.05 software. The numbers on the nodes indicate the bootstrap values after 1000 replicates. The scale bar indicates the estimated number of amino acid substitutions per site. Accession numbers for the *A. thaliana* sequences are AtABCA1 (NP_850354.2), AtABCA2 (NP_190357.2), AtABCA3 (NP_190358.3), AtABCA4 (NP_190359.5), AtABCA5 (NP_190360.2), AtABCA6 (NP_190361.2), AtABCA7 (NP_190362.2), AtABCA8 (NP_190363.3), AtABCA9 (NP_200981.1), AtABCA10 (NP_200982.1), AtABCA11 (NP_200977.2), AtABCA12 (NP_200978.1), AtABCC1 (NP_174329.1), AtABCC2 (NP_181013.1), AtABCC3 (NP_187915.1), AtABCC4 (NP_182301.1), AtABCC5 (NP_171908.1), AtABCC6 (NP_187916.3), AtABCC7 (NP_187917.3), AtABCC8 (NP_001189944.1), AtABCC9 (NP_191575.2), AtABCC10 (NP_191473.2), AtABCC11 (NP_174331.2), AtABCC12 (Q9C8H0.1), AtABCC13 (Q9SKX0.3), AtABCC14 (NP_191829.1), AtABCC15 (NP_191656.2), AtABCD1 (NP_568072.1) and AtABCD2 (NP_175837.2). AtSKD1 (AEC08019.1) and HbSKD1 (AIN75626.1) were used as outgroups. The *H. brasiliensis* latex ABC proteins are marked with a dot.

**HbABCA subfamily**. Three members were identified in the HbABCA subfamily. These were one full-size HbABCA1 member and two half-size members: HbABCA2 and HbABCA7. Until recently, only one full-size ABCA transporter gene has been identified in plants and was designated as AtABCA1 or AOH (the ABC one homolog) in the *A. thaliana* genome [2]. HbABCA1 was the largest latex ABC transporter, consisting of 1,883 amino acid residues, and shared 74% similarity with AtABCA1. The phylogenetic relationship between *A. thaliana* and *H. brasiliensis* ABCA proteins is shown in Fig. 3. The ABCA1 gene is conserved in humans and other animals. Its human counterpart (ABCA1) is involved in cellular transportation [40, 41], but the function and the localization of the plant ABCA1 transporter remains unclear.

**Figure 4:**
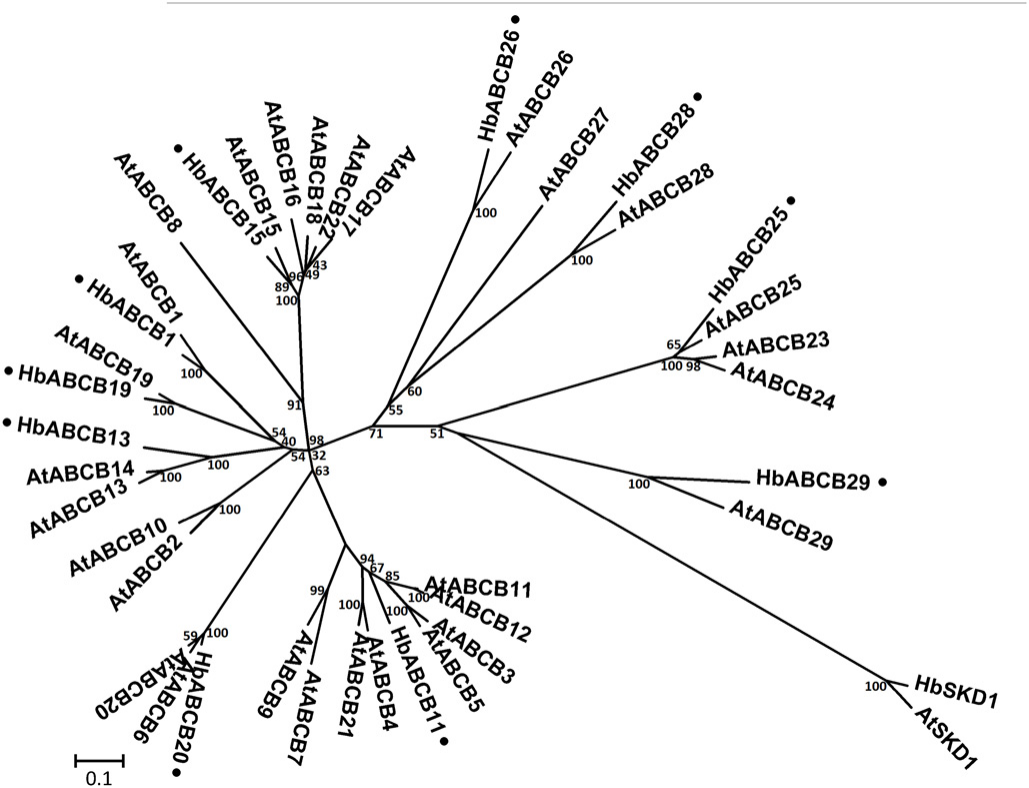
Phylogenetic tree for *A. thaliana* and *H. brasiliensis* latex ABCB protein sequences. The amino acid sequences of all *A. thaliana* ABCB and *H. brasiliensis* latex proteins were aligned using the MUSCLE program and subjected to phylogenetic analysis by the distance with neighbor-joining method using MEGA5.05 software. The numbers on the nodes indicate the bootstrap values after 1000 replicates. The scale bar indicates the estimated number of amino acid substitutions per site. Accession numbers for the *A. thaliana* sequences are AtABCB1 (NP_181228.1), AtABCB2 (NP_194326.2), AtABCB3 (NP_192091.1), AtABCB4 (NP_182223.1), AtABCB5 (NP_192092.1), AtABCB6 (NP_181480.1), AtABCB7 (NP_199466.1), AtABCB8 (Q9LHK4.1), AtABCB9 (NP_193539.6), AtABCB10 (NP_172538.1), AtABCB11 (NP_171753.1), AtABCB12 (NP_171754.1), AtABCB13 (NP_174115.1), AtABCB14 (NP_174122.1), AtABCB15 (NP_189475.1), AtABCB16 (NP_189477.4), AtABCB17 (NP_189479.1), AtABCB18 (NP_189480.1), AtABCB19 (NP_189528.1), AtABCB20 (NP_191092.1) AtABCB21 (NP_191774.2), AtABCB22 (NP_683599.1), AtABCB23 (NP_567813.1), AtABCB24 (NP_194591.1), AtABCB25 (NP_200635.1), AtABCB26 (NP_177218.3), AtABCB27 (NP_198720.2), AtABCB28 (NP_194275.2) and AtABCB29 (NP_196011.1). AtSKD1 (AEC08019.1) and HbSKD1 (AIN75626.1) were used as outgroups. The *H. brasiliensis* latex ABC proteins are marked with a dot.

**HbABCB subfamily**. The latex HbABCB subfamily contains ten members. Six are full-size molecules, conventionally named multi-drug resistance (MDRs) [2] or drug, peptide and lipid exporters (DPLs)/PGP (similar to p-glycoprotein) [3] and four are half-size proteins, consisting of one ABC transporter in mitochondria (ATM) [2], two TAPs (similar to the human transporter associated with antigen presentation) [2] and one LLP (lipid A-like exporters, putative) [3]. All 21 full-size ABCB transporters in the *A. thaliana* genome have been shown to be localized on the plasma membrane [6, 42, 43] and AtABCB1, AtABCB4, AtABCB14, AtABCB15 and AtABCB19, have been characterized as auxin transporters or are associated with polar auxin transport in *A. thaliana* [44–46]. Furthermore, ABCB14-mediated auxin transport was recently reported to be involved in Fe homeostasis in rice [47]. Orthologs of AtABCB1, AtABCB15 and AtABCB19 were also identified in *H. brasiliensis* latex (Fig. 4), but it remains to be determined whether they are implicated in auxin transport in the *H. brasiliensis* laticifers that are apoplastically isolated from the neighboring cells [25].

Less information is available about the function and the localization of the half-size members of the plant ABCB subfamily. Among the four half-size ABCB proteins in the *H. brasiliensis* latex, one member, HbABCB25, is a close homolog of *A. thaliana* ABCB25/ATM3, which is a mitochondrial ABC transporter involved in the biogenesis of iron-sulfur proteins and molybdenum-containing enzymes in plants [48, 49]. Proteomic data from previous studies have demonstrated that *A. thaliana* ABCB26/TAP1 is localized to the chloroplast [50] and the results of this study suggest that the latex HbABCB26, a homolog of At ABCB26/TAP1, might possibly be localized to the Frey-Wyssling particles, which are very specialized chromoplasts in rubber tree latex [51]. However, this needs to be verified.

**HbABCC subfamily**. In plants, ABCC subfamily transporters belong to the multidrug resistance-associated proteins (MRPs) that were originally identified in human drug-resistant cell lines [52]. They only have full-size ABC proteins that contain an additional N-terminal extension (NTE or TMD0) of around 220 amino acids [53]. Three members of the ABCC subfamily were identified in the *H. brasiliensis* latex transcriptome, whereas 15 ABCC protein genes were determined in the *A. thaliana* genome [2]. Their sequence similarity was confirmed by the phylogenetic analysis (as shown in Fig. 3). Until recently, only the full-size ABCC subfamily transporters have been shown to be localized on the vacuolar membrane in plants [54]. Furthermore, their localization to the tonoplasts gives ABCC subfamily transporters an important function in the general vacuolar sequestration of conjugated metabolites, such as anthocyanidin 3-O-glucosides [55], abscisic acid (ABA) glucosyl ester [56] and phenolic compounds [57], etc. In the case of the *H. brasiliensis* laticifers, no typical central vacuoles have been observed, although polydispersed microvacuoles called lutoids are present, and laticifers have been implicated in pH regulation and solute accumulation [58, 59]. It has not been confirmed whether the latex ABCC transporters are intrinsic lutoid membrane proteins that are involved in the maintenance of ion homeostasis between lutoids and cytosol, which is crucial for lutoid-mediated rubber particle aggregation and sequential latex coagulation.

**HbABCD subfamily**. The ABCD subfamily transporters are conventionally designated as PMPs (peroxisomal membrane protein), which are intrinsic membrane proteins of peroxisomes and are mainly involved in fatty acid β-oxidation through the importation of acyl-CoA esters into the peroxisome [60]. Only half-size ABCD proteins have been detected in mammals and fungi, whereas both half-size and full-size ABCD transporters have been detected in plants. The prototypical plant member of the ABCD subfamily is the *A. thaliana* protein, Comatose (CTS; also known as AtABCD1) [61, 62]. Until recently, only one half-size and one full-size member of the ABCD subfamily have been identified in the *A. thaliana* genome and their orthologs were also present in the *H. brasiliensis* latex transcriptome (as shown in Fig. 3). Loss-of-function *AtABCD1* mutants are significantly impaired in several important metabolic and developmental processes, such as germination, fertility, seedling establishment and root growth. These studies provided evidence that, besides the involvement in fatty acid β-oxidation, the plant ABCD transporters are also involved in auxin and jasmonic acid (JA) metabolism via the importation of the auxin precursor, indolbutyric acid (IBA), and the JA precursor, 12-oxophytodienoic acid (OPDA), into the peroxisome [63, 64]. Several attempts have now been made to elucidate the physiological and molecular characteristics of JA in *H. brasiliensis* since recent reports demonstrated that JA and its conjugate, methyl jasmonic acid (Me-JA), are key inducers of laticifer differentiation [65] and are regulators of rubber biosynthesis-related genes in rubber trees [28]. Further investigation is needed to ascertain whether the latex ABCD transporters mediate JA biosynthesis in the *H. brasiliensis* laticifers, which are the natural rubber-producing cells of rubber trees.

**Figure 5:**
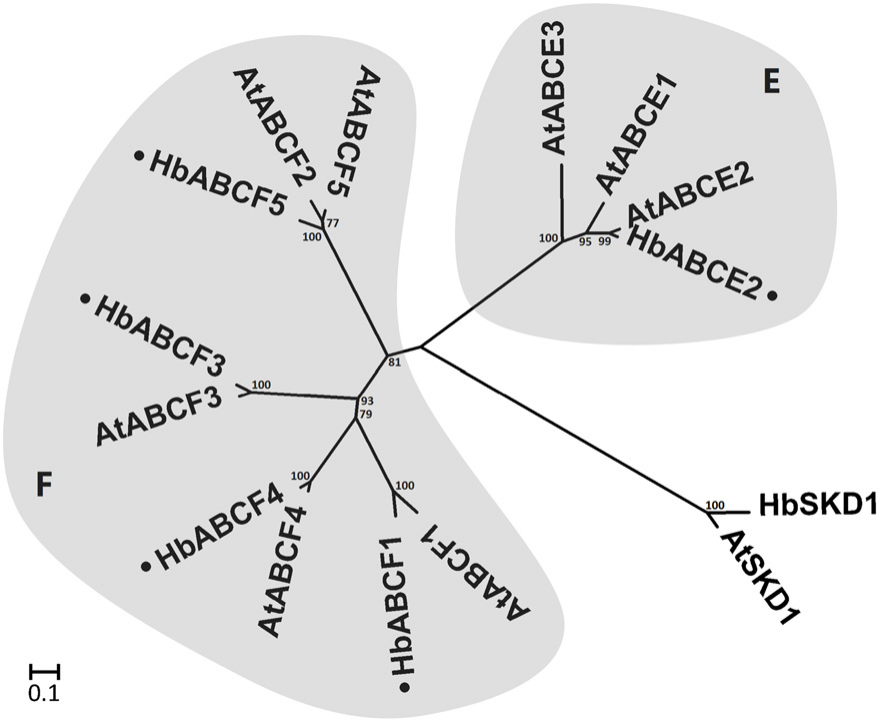
Phylogenetic relationship between *A. thaliana* and *H. brasiliensis* latex ABCE/ABCF proteins. The amino acid sequences of *A. thaliana* ABCE/ABCF proteins and *H. brasiliensis* latex proteins were aligned using the MUSCLE program and subjected to phylogenetic analysis by the distance with neighbor-joining method using MEGA5.05 software. The numbers on the nodes indicate the bootstrap values after 1000 replicates. The scale bar indicates the estimated number of amino acid substitutions per site. Accession numbers for the *A. thaliana* sequences are AtABCE1 (NP_187973.1), AtABCE2 (NP_193656.2), AtABCE3 (NP_194759.1), AtABCF1 (NP_200887.1), AtABCF2 (NP_196555.2), AtABCF3 (NP_176636.1), AtABCF4 (NP_567001.1) and AtABCF5 (NP_201289.1). AtSKD1 (AEC08019.1) and HbSKD1 (AIN75626.1) were used as outgroups. The *H. brasiliensis* latex ABC proteins are marked with a dot.

**HbABCE and HbABCF subfamilies**. The members of the ABCE and the ABCF subfamilies are thought to be soluble proteins that contain only two NBDs and no TMDs. The *A. thaliana* genome contains three ABCE and five ABCF members, whereas only one ABCE protein and four ABCF proteins were identified in the *H. brasiliensis* latex transcriptome (Fig. 5). However, information is relatively scarce about these soluble ABC proteins compared with other intrinsic membrane ABC transporters. They are probably involved in non-transport processes, as is the case for their yeast and human orthologs, which participate in ribosome recycling and translational control [54]. Only one ABC transporter, which is a member of the ABCF subfamily, was identified in rubber particles through proteome analysis [66], but it is unclear whether this protein is related to biogenesis or to the biosynthesis of rubber particles.

**Figure 6:**
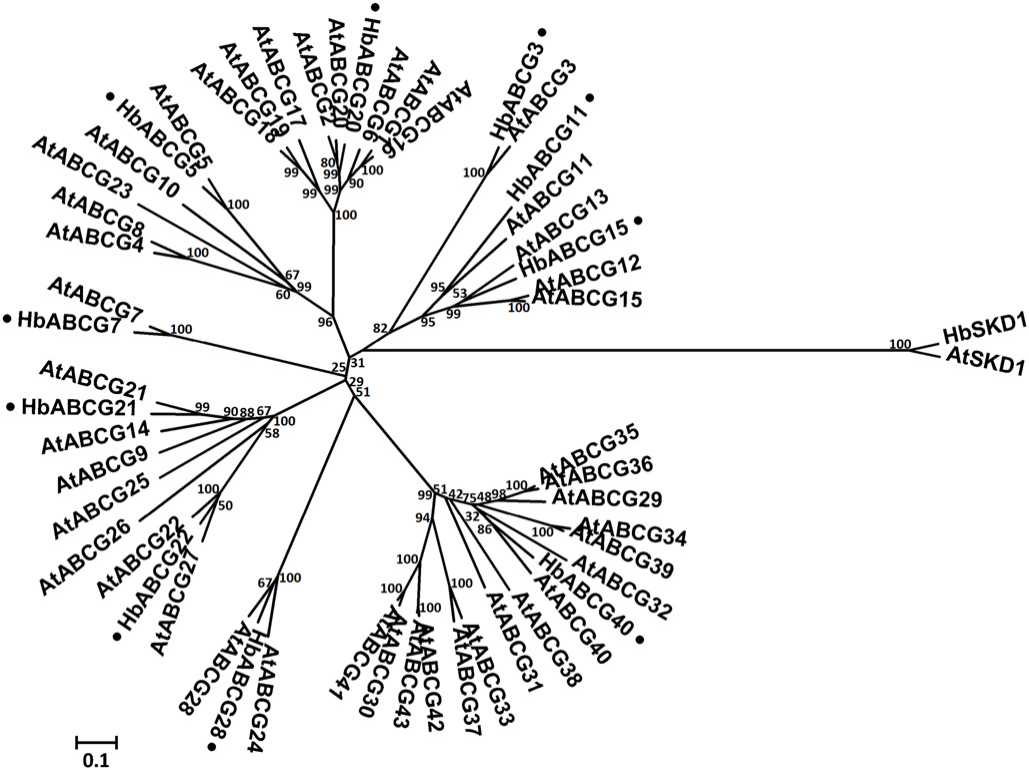
Phylogenetic relationship between *A. thaliana* and *H. brasiliensis* latex ABCG proteins. The amino acid sequences of all the *A. thaliana* ABCG proteins and *H. brasiliensis* latex proteins were aligned using the MUSCLE program and subjected to phylogenetic analysis by the distance with neighbor-joining method using MEGA 5.05 software. The numbers on the nodes indicate the bootstrap values after 1000 replicates. The scale bar indicates the estimated number of amino acid substitutions per site. Accession numbers for the *A. thaliana* sequences are AtABCG1 (NP_181467.1), AtABCG2 (NP_181272.1), AtABCG3 (NP_850111.1), AtABCG4 (NP_194305.1), AtABCG5 (NP_178984.1), AtABCG6 (NP_196862.1), AtABCG7 (NP_178241.1), AtABCG8 (NP_200098.1), AtABCG9 (NP_194472.3), AtABCG10 (NP_175734.1), AtABCG11 (NP_173226.2), AtABCG12 (NP_175561.1), AtABCG13 (NP_175557.1), AtABCG14 (NP_564383.1), AtABCG15 (NP_188746.2), AtABCG16 (NP_191069.2), AtABCG17 (NP_191070.1), AtABCG18(NP_191071.1), AtABCG19 (NP_191073.1), AtABCG20 (NP_190919.1), AtABCG21 (NP_189190.2), AtABCG22 (NP_568169.1), AtABCG23 (NP_197442.1), AtABCG24 (NP_175745.4), AtABCG25 (NP_565030.1), AtABCG26 (NP_187928.2), AtABCG27 (NP_190799.2), AtABCG28 (NP_200882.4), AtABCG29 (NP_566543.1), AtABCG30 (NP_193258.3), AtABCG31 (NP_180555.2), AtABCG32 (NP_180259.1), AtABCG33 (NP_181265.1), AtABCG34 (NP_181179.2), AtABCG35 (NP_172973.1), AtABCG36 (NP_176196.1), AtABCG37 (NP_190916.1), AtABCG38 (Q7PC85.1), AtABCG39 (NP_176867.2), AtABCG40 (NP_173005.1), AtABCG41 (NP_680692.1), AtABCG42 (NP_680693.5) and AtABCG43 (NP_680694.2). AtSKD1 (AEC08019.1) and HbSKD1 (AIN75626.1) were used as outgroups. The *H. brasiliensis* latex ABC proteins are marked with a dot.

**HbABCG subfamily**. The largest ABC subfamily is ABCG, which consists of 28 half-size (WBC) and 15 full-size (PDR) transporters in the *A. thaliana* genome. All the ABCG subfamily transporters have a unique reverse “NBD-TMD” orientation and are reported to be plasma membrane-localized proteins, except for AtABCG19, which has been localized to the vacuolar membrane and confers kanamycin-resistance in plants [67]. In the *H. brasiliensis* latex transcriptome, nine WBC-type ABCG proteins and one PDR-type ABCG protein were detected. The phylogenetic analyses of *A. thaliana* and *H. brasiliensis* latex ABCG proteins are shown in Fig. 6.

ABCG half-transporters have been implicated in several plant functions. *A. thaliana* AtABCG25 and AtABCG40 are involved in abscisic acid (ABA) transport and responses [68, 69]. Recent studies have indicated that ABCG9, ABCG11 and ABCG14 are involved in lipid/sterol homeostasis regulation, which is required for proper vascular development in *A. thaliana* [70]. Finally, some members of ABCG subfamily confer plant resistance to various biotic and abiotic stresses [71, 72].

**Figure 7:**
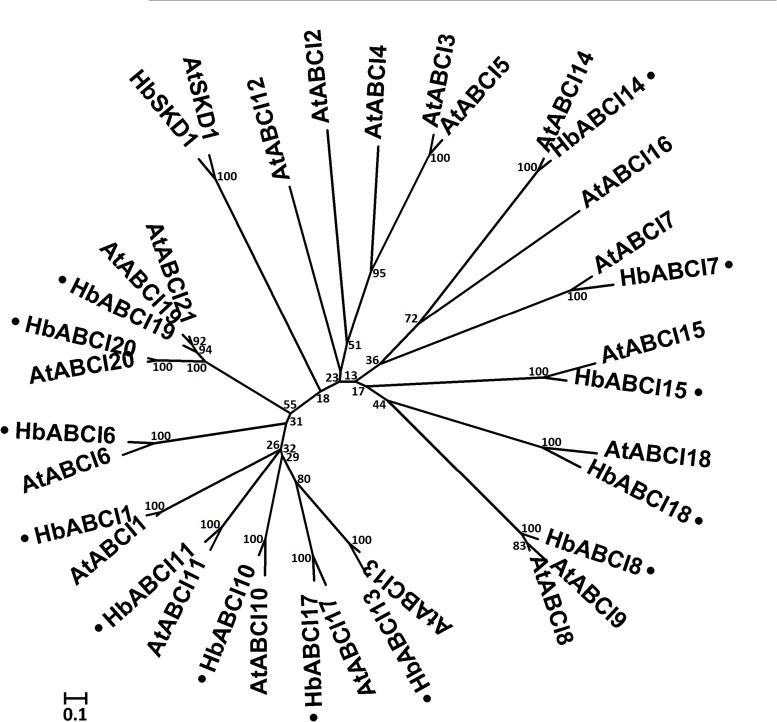
Phylogenetic relationship between *A. thaliana* and *H. brasiliensis* latex ABCI proteins. The amino acid sequences of all the *A. thaliana* ABCI proteins and *H. brasiliensis* latex proteins were aligned using the MUSCLE program and subjected to phylogenetic analysis by the distance with neighbor-joining method using MEGA 5.05 software. The numbers on the nodes indicate the bootstrap values after 1000 replicates. The scale bar indicates the estimated number of amino acid substitutions per site. Accession numbers for the *A. thaliana* sequences are AtABCI1 (NP_176516.1), AtABCI2 (NP_085482.1), AtABCI3 (NP_085546.2), AtABCI4 (NP_565344.2), AtABCI5 (NP_973435.1), AtABCI6 (NP_187678.1), AtABCI7 (NP_564404.1), AtABCI8 (NP_192386.1), AtABCI9 (NP_680390.1), AtABCI10 (NP_195072.2), AtABCI11 (NP_196914.1), AtABCI12 (NP_566688.4), AtABCI13 (NP_564850.1), AtABCI14 (NP_973869.1), AtABCI15 (NP_566659.1), AtABCI16 (NP_181270.1), AtABCI17 (NP_176961.1), AtABCI18 (NP_563693.1), AtABCI19 (NP_563694.1), AtABCI20 (NP_195847.1), and AtABCI21 (NP_199224.1). AtSKD1 (AEC08019.1) and HbSKD1 (AIN75626.1) were used as outgroups. The *H. brasiliensis* latex ABC proteins are marked with a dot.

**HbABCI subfamily**. The ABCI subfamily proteins consist of bacteria-type soluble or peripheral ABC components with a single NBD and are designated as non-intrinsic ABC proteins (NAPs) [2]. Genetic evidence has demonstrated that the ABCI subfamily protein subunits include not only NBD and TMD domains, but also homologues of established soluble cytosolic proteins that interact with NBDs and putative substrate-binding proteins and are similar to the periplasmic-binding proteins in bacteria [3, 73, 74]. A total of 13 ABCI proteins were identified in the *H. brasiliensis* latex transcriptome and there were 21 orthologs in the *A. thaliana* genome (Fig. 7). Three *A. thaliana* ABCI proteins: AtABCI19, 20 and 21 have been shown to translate into cytosolic proteins [75], whereas several other ABCI subfamily members are predicted to be localized to the chloroplast or mitochondria [1, 76].

The functional significance of the ABCI proteins remains to be determined. Previous studies have suggested that *A. thaliana* ABCI subfamily proteins can be assembled into multi-subunit ABC transporters, which is similar to the way that ABC proteins are formed in prokaryotes. Five members of the *A. thaliana* ABCI proteins: AtABCI1, 2, 3, 4 and 5, have been reported to be involved in cytochrome c biogenesis [77], AtABCI6, 7 and 8 were responsible for Fe-S cluster assembly and iron homeostasis regulation [78, 79] and AtABCI13, 14 and 15 have been shown to be involved in plastid lipid formation [80–83].

### Expression Profiling of the Latex ABC Protein Genes

Plant ABC family proteins have been shown to be involved in many physiological processes that allow the plant to adapt to changing environments and to cope with biotic and abiotic stresses. To investigate further the possible roles played by latex ABC transporters during latex metabolism in rubber trees, the gene expression patterns of all the latex ABC transporters were first ascertained using rubber trees, which were treated with Ethrel or Me-JA for 24 h or tapped at the seventh times. The results demonstrated that the most of the latex ABC transporter genes were controlled by Ethrel, Me-JA or bark tapping to varying degrees (S5 Table), among which *HbABCB15*, *HbABCB19*, *HbABCD1* and *HbABCG21* were significantly up-regulated by the three different stimulations.

**Figure 8:**
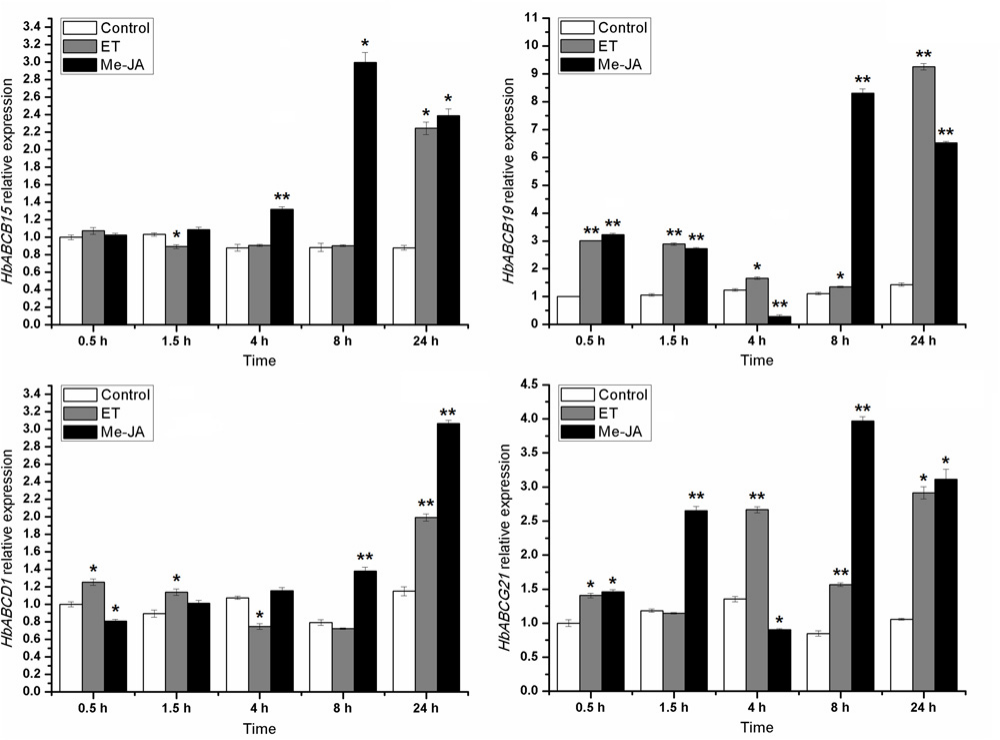
Regulatory effects of ethylene and JA on the gene expressions of the four HbABC transporters in the *H. brasiliensis* latex. Mature, virgin (untapped) rubber trees were treated with Ethrel (ET) or methyl-jasmonate (Me-JA) for 0.5, 1.5, 4.0, 8.0 or 24.0 h. The controls were rubber trees that had not been treated with stimulants. Three independent biological replicates were included in each sample. Fresh latex from each sample was collected and used to isolate the total RNA. The transcript abundance of each gene was detected by RT-qPCR and the values are shown as the mean ± S.D. Statistical significance was determined using Students *t*-test using SPSS 19.0 software (Chicago, U.S.A.). When compared with the control, one asterisk shows a significant difference with a *P*-value < 0.05 and two asterisks show a very significant difference with a *P*-value < 0.01.

**Figure 9:**
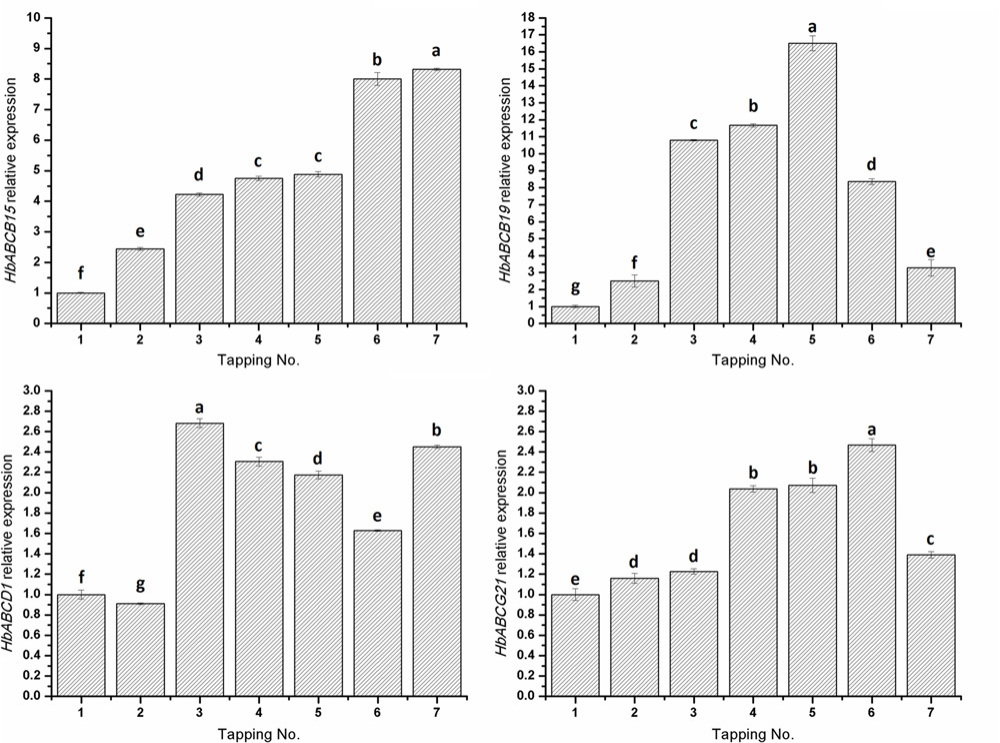
Effects of bark tapping on the gene expressions of the four HbABC transporters in the *H. brasiliensis* latex. Mature, virgin rubber trees were tapped sequentially seven times using an S/2 d/3 tapping system. Each sample contained three independent biological replicates. Fresh latex from each tapping was collected and used to isolate the total RNA. The transcript abundance of each gene was detected by RT-qPCR and the values are shown as the mean ± S.D. One-way ANOVA was performed using SPSS 19.0 software (Chicago, U.S.A.). The Student–Newman–Keuls test was used for multiple comparisons testing to investigate the significant differences between groups. Bars with different letters are significantly different at the *p* < 0.05 level. The values represent the mean ± SD of three biological replicates.

The gene expression time courses for *HbABCB15*, *HbABCB19*, *HbABCD1* and *HbABCG21* were further analyzed using rubber trees that had been treated with Ethrel or Me-JA at different time points. The data indicated that although the four HbABC genes were highly regulated by Ethrel or Me-JA, their individual expression profiling was different from each other (Fig. 8). Bark tapping, as a simulated wound stress, could also induce expression of the four selected ABC transporter genes, but in different ways (Fig. 9). Several studies reported that salicylic acid (SA), Me-JA/JA and ABA could modulate the gene expression of plant ABC transporters [84–86]. However, information about the responses of ABC protein genes to ethylene and bark tapping (a wound stress) is still relatively limited.

Ethrel, Me-JA and bark tapping are three efficient stimulants of latex production and laticifer differentiation in rubber trees, but the underlying molecular mechanisms remain poorly understood. The identification and expression analyses of the latex ABC transporter genes provide some valuable insights into how the related latex ABC transporters regulate latex metabolism/regeneration and rubber biosynthesis by *H. brasiliensis*.

### Concluding Remarks

The physiological functions of plant ABC transporters have only been marginally characterized compared to human ABC transporters. The whole latex transcriptome survey has produced an inventory of the laticifer-specific ABC transporters of *H. brasiliensis*. Laticiferous cells are where natural rubber is synthesized and stored. To date, the transportation of various substrates related to latex metabolism and rubber biosynthesis inside the laticiferous cells has been poorly understood. Several latex ABC transporters were transcriptionally induced by ethylene, JA and bark tapping (a wound stress), all of which activate latex metabolism or laticifer differentiation in rubber trees. The identification and expression analysis of the latex ABC transporters may facilitate further studies into their possible involvement in latex metabolism and rubber biosynthesis by both *H. brasiliensis* and other latex-producing plants.

## Supporting Information

S1 Fig NBD phylogeny of the *H. brasiliensis* latex ABC proteins containing a NBD domain. The conserved NBD and TMD domains in the *H. brasiliensis* latex ABC transporters were predicted by Pfam 27.0 (http://pfam.xfam.org/). The amino sequences of the latex ABC protein NBDs were aligned using the MUSCLE program and subjected to phylogenetic analysis by the distance with neighbor-joining method (1000 bootstrap replicates) using MEGA5.05 software. The scale bar indicates the estimated number of amino acid substitutions per site.
(TIF)

S1 Table Oligonucleotide primers used for RT-qPCR analysis of the ABC protein genes in the *H. brasiliensis* latex. (DOC)

S2 Table Identification of ABC protein genes in the *H. brasiliensis* latex through a reciprocal best hits blast search. (DOC)

S3 Table The genome DNA sequences of the 46 ABC protein genes identified in the *H. brasiliensis* latex. (DOC)

S4 Table The amino acid sequences of the 46 ABC proteins identified in the *H. brasiliensis* latex. (DOC)

S5 Table Gene expression analyses of the *H. brasiliensis* latex ABC transporters by RT-qPCR. (DOC)
