# Supplementary figures and images for: Whole-Transcriptome Survey of the Putative ATP-Binding Cassette (ABC) Transporter Family Genes in the Latex-Producing Laticifers of Hevea brasiliensis
Source: PLoS One. 2015 Jan 23;10(1):e0116857. doi: 10.1371/journal.pone.0116857 (PMC4304824; doi:10.1371/journal.pone.0116857)

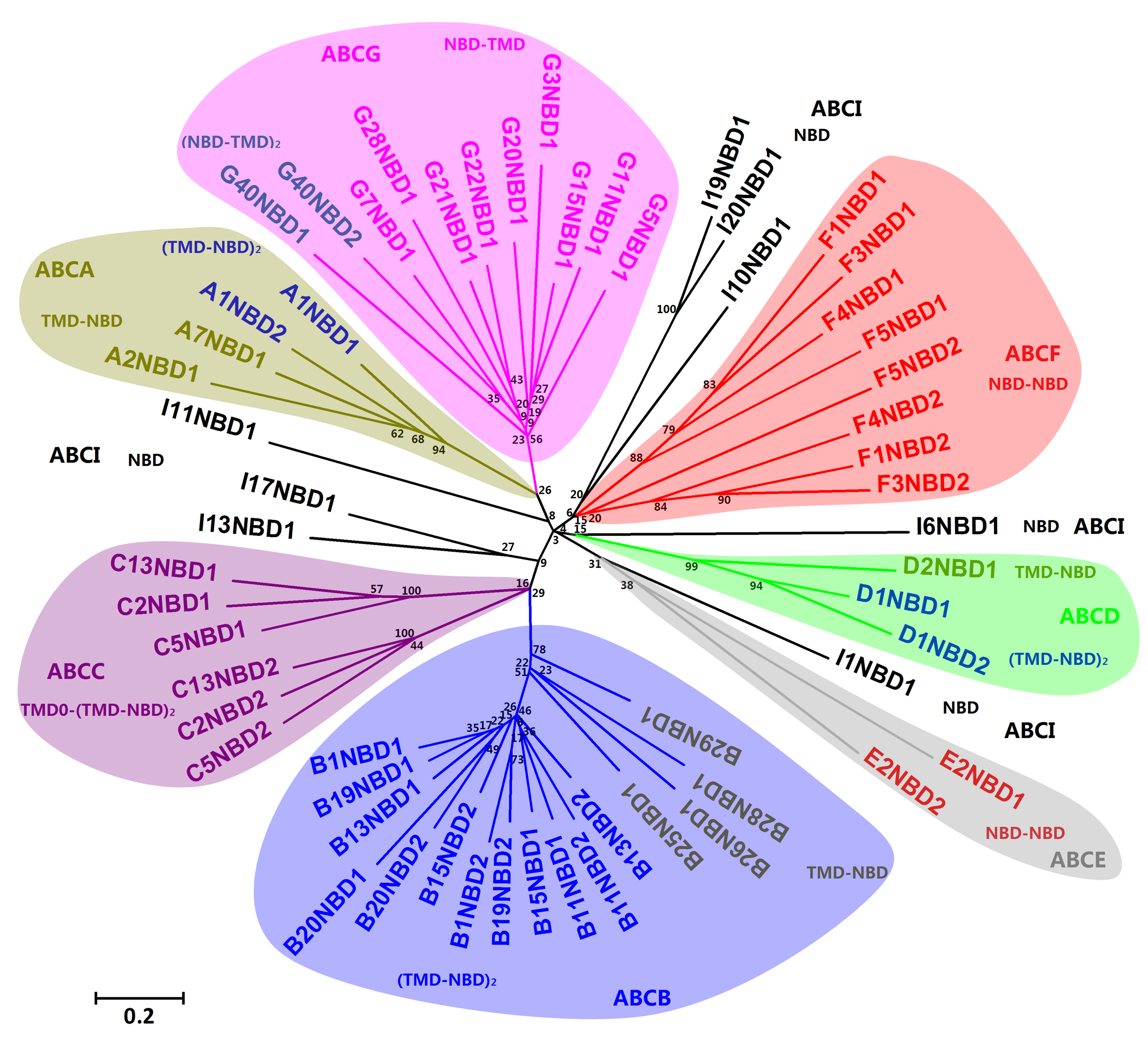

Supplement: S1 Fig — The conserved NBD and TMD domains in the H. brasiliensis latex ABC transporters were predicted by Pfam 27.0 (http://pfam.xfam.org/). The amino sequences of the latex ABC protein NBDs were aligned using the MUSCLE program and subjected to phylogenetic analysis by the distance with neighbor-joining method (1000 bootstrap replicates) using MEGA5.05 software. The scale bar indicates the estimated number of amino acid substitutions per site. (TIF) [file pone.0116857.s001.tif]
