# Supplementary material for: Whole-Transcriptome Survey of the Putative ATP-Binding Cassette (ABC) Transporter Family Genes in the Latex-Producing Laticifers of Hevea brasiliensis
Source: PLoS One. 2015 Jan 23;10(1):e0116857. doi: 10.1371/journal.pone.0116857 (PMC4304824; doi:10.1371/journal.pone.0116857)
Supplement: S1 Table — (DOC) [file pone.0116857.s002.doc]

Table S1. Oligonucleotide primers used for the RT-qPCR reactions of the *H. brasiliensis* latex ABC protein genes

| Primer name | Sequence (5’-3’) | Primer efficiency | PCR product (bp) |
| --- | --- | --- | --- |
| **HbABCA1_FW** | TACGGAATGGACTTGGTGTGT | 1.870 | 187 |
| **HbABCA1_RV** | GAAAGAGCCCTCACAACAGTG |
| **HbABCA2_FW** | GCCTCATTCCTCCAGTTGTT | 1.812 | 132 |
| **HbABCA2_RV** | CGAAACCAGAGGCTGAGGAT |
| **HbABCA7_FW** | GCTATTTCCTGTGGTGCTGAC | 1.832 | 106 |
| **HbABCA7_RV** | GGAAATCATCCAGTAAGGACCAT |
| **HbABCB1_FW** | CAGGAGATAAAGACGATTGAGCAG | 1.921 | 123 |
| **HbABCB1_RV** | TGTTAGAGTTGGAGTTGGAGGAG |
| **HbABCB11_FW** | AGCAGCCTTTCTTCAGGTGAC | 1.943 | 156 |
| **HbABCB11_RV** | TCACCAGACATTCTACCAACAAC |
| **HbABCB13_FW** | CAGAAACAAAGAGTGGCTATTGC | 1.884 | 146 |
| **HbABCB13_RV** | ACTGTTGTTCGTCCTTCCATAAG |
| **HbABCB15_FW** | ATGGGTTGTTTGTTTCGTAGAG | 1.997 | 200 |
| **HbABCB15_RV** | CCTTCTCACTTAGAACGTCTTGA |
| **HbABCB19_FW** | TCGCTCTCTATGCCTCTGAAG | 1.895 | 153 |
| **HbABCB19_RV** | TCTCTGGAGCAAGGCTAACAG |
| **HbABCB20_FW** | TGGAGGACGAATAGTTGAGGA | 1.835 | 138 |
| **HbABCB20_RV** | ATGCCCAAATGTCAACCAAC |
| **HbABCB25_FW** | CTGTTGATTGGTTGAGCACTG | 1.879 | 174 |
| **HbABCB25_RV** | TAGAGAACACGGCAGTCCTCAG |
| **HbABCB26_FW** | AGTTGGACATCAAGTGGTTCAG | 1.953 | 179 |
| **HbABCB26_RV** | ATTGGGCAGAGAGGAGATGA |
| **HbABCB28_FW** | GCTTATGGGCTTCCAGATGATA | 1.969 | 151 |
| **HbABCB28_RV** | GCCTCTGTCCTCCACTCAATAA |
| **HbABCB29_FW** | GCTCATCGGTTGGAAACAGT | 1.934 | 141 |
| **HbABCB29_RV** | ATGCCCTCAAACCACAAGTC |
| **HbABCC2_FW** | TTTGGAGAGAGCACACCTGAAG | 1.886 | 184 |
| **HbABCC2_RV** | TCTCACATCAACAGCAGCAGTAG |
| **HbABCC5_FW** | CGAGTGAAGTTGGAGGAGATG | 1.977 | 171 |
| **HbABCC5_RV** | AAGAACACCTCCAGCAGTGAG |
| **HbABCC13_FW** | GCTAACGTGGACACTCAAACAG | 1.885 | 160 |
| **HbABCC13_RV** | GGTTCCCTTGCTCAATCACA |
| **HbABCD1_FW** | TGGGAGAGCAACAAAGGTTAG | 1.954 | 131 |
| **HbABCD1_RV** | CATAGCAAGAACTTTCGCACA |
| **HbABCD2_FW** | TGAAGTCAATGAGGCACATCTG | 1.923 | 171 |
| **HbABCD2_RV** | CCAGGATTTATGGACTCAATGTC |
| **HbABCE2_FW** | TGGCAGGAAATCTTGACCTAC | 1.902 | 150 |
| **HbABCE2_RV** | GTCAAGCACCTGCCCTACAT |
| **HbABCF1_FW** | TCAAGCACAGAGCAAGGAGA | 2.002 | 137 |
| **HbABCF1_RV** | CCACAAACTGAAGCACAGGA |
| **HbABCF3_FW** | TGCGTCTCCGACTTCTCTGA | 1.795 | 170 |
| **HbABCF3_RV** | TCCATAACCTCAGGCTTCTTCA |
| **HbABCF4_FW** | GGCTCAGGCATCAAAGATTCT | 1.821 | 174 |
| **HbABCF4_RV** | GCTCTCAGGTCAAGGTGGTTA |
| **HbABCF5_FW** | CCTGAGGATTCGGATAGGTTG | 1.883 | 172 |
| **HbABCF5_RV** | CGTCTTGCTTCTGGAGATAACCT |
| **HbABCG3_FW** | TGGTGAGAGAAGGCGTGTTAG | 1.963 | 113 |
| **HbABCG3_RV** | CCATCATCAGAAGAGCAGAGACA |
| **HbABCG5_FW** | TGGGTGGTCTTGAGAGAGAAG | 2.021 | 135 |
| **HbABCG5_RV** | TGGGTGGAAATCTTGAAGGT |
| **HbABCG7_FW** | TGCTTTCCAGGCAGAGAGAGT | 1.936 | 159 |
| **HbABCG7_RV** | GCAGGACCAGCATAAACAAGTG |
| **HbABCG11_FW** | GAAGGAGAGAGCAGAGAGGACA | 1.942 | 169 |
| **HbABCG11_RV** | TGTGGGTTCATCAAGGAAGAG |
| **HbABCG15_FW** | CCTGGTGGCTGTTGTAGTCA | 1.955 | 167 |
| **HbABCG15_RV** | AAGAAGGGAAGGGTGAACTGT |
| **HbABCG20_FW** | CGGTGGTGACAACCTTCTGTA | 1.967 | 154 |
| **HbABCG20_RV** | CGGATAGGAGTGCTGCCAT |
| **HbABCG21_FW** | AACAGGCTTTGTCGCTCAAG | 1.896 | 181 |
| **HbABCG21_RV** | CCCACCCACTACACTGTTTCTA |
| **HbABCG22_FW** | GGTGGCAAGAACACACACAT | 1.865 | 131 |
| **HbABCG22_RV** | CCGGTGAAGGAGAAAGACAAG |
| **HbABCG28_FW** | GTCCAGCGAAGAAAGTTGAAG | 1.894 | 170 |
| **HbABCG28_RV** | CCCATTATGAAGCATCCATCTG |
| **HbABCG40_FW** | CGGGATACTCTTGAAGATGCAG | 1.909 | 168 |
| **HbABCG40_RV** | GGGAATTGAATATGACCATGCAG |
| **HbABCI1_FW** | GGCACAAATACTGGAAGCATC | 1.852 | 102 |
| **HbABCI1_RV** | ACCCAACCAACCAAGTCTCAG |
| **HbABCI6_FW** | CTTCCAATCGCCAGTTGAGA | 1.871 | 130 |
| **HbABCI6_RV** | CGGAAACAGGTAAGCATAGAAC |
| **HbABCI7_FW** | TGTCTTGGAAGTGATGATTGAAG | 1.808 | 145 |
| **HbABCI7_RV** | GCCAGTGCTTACCTCAACAAG |
| **HbABCI8_FW** | GGCAGTTTGAGAGGACTTTGATA | 1.861 | 178 |
| **HbABCI8_RV** | TCATCACCAGCATACCAGTTC |
| **HbABCI10_FW** | TGACTGAGGATGAAGTTAGGCA | 1.903 | 184 |
| **HbABCI10_RV** | TGGTCCAAGAATGTTGTGAGC |
| **HbABCI11_FW** | TTCTTGCTGGGCTAAGTGAAC | 1.926 | 128 |
| **HbABCI11_RV** | CGGAAACTGAAAGACAATACCA |
| **HbABCI13_FW** | GGGAGAGGAACAAATCTTAGAG | 1.851 | 106 |
| **HbABCI13_RV** | TTTCTTCATCCCACCAGACAG |
| **HbABCI14_FW** | GTCACCCTTCTGTCTCTCTGCTA | 1.877 | 136 |
| **HbABCI14_RV** | CCTGCCAAGACAAGAACTGATAA |
| **HbABCI15_FW** | TGAAGCAGTTGTTGAGGTTGAA | 1.891 | 201 |
| **HbABCI15_RV** | CCCTTTATCTTCTGCCTATCACA |
| **HbABCI17_FW** | CCGTAAGGTTGGAATGCTCTT | 1.924 | 150 |
| **HbABCI17_RV** | AAGGAAGAATCAAGGTCAGCAA |
| **HbABCI18_FW** | GACATCGGTAGAGACGGTGCTA | 1.793 | 139 |
| **HbABCI18_RV** | ATCCGATAACGCCACATTGA |
| **HbABCI19_FW** | TGTTGAAGATTCTGGCTGGTAAG | 1.946 | 174 |
| **HbABCI19_RV** | AGTCACCCTGGAGTGGTATCTC |
| **HbABCI20_FW** | AAAGGCAAGTGGTCTTCCAGA | 1.895 | 99 |
| **HbABCI20_RV** | CAACCATTGTTCATAGCACGA |
| **Hb18S rRNA_FW** | GCTCGAAGACGATCAGATACC* | 1.880 | 146 |
| **Hb18S rRNA_RV** | TTCAGCCTTGCGACCATAC* |

* *H.brasiliensis* 18S rRNA gene with GenBank acc. No.: AB268099 was used as internal reference.
