# Supplementary material for: Whole-Transcriptome Survey of the Putative ATP-Binding Cassette (ABC) Transporter Family Genes in the Latex-Producing Laticifers of Hevea brasiliensis
Source: PLoS One. 2015 Jan 23;10(1):e0116857. doi: 10.1371/journal.pone.0116857 (PMC4304824; doi:10.1371/journal.pone.0116857)
Supplement: S2 Table — (DOC) [file pone.0116857.s003.doc]

Table S2. Identification of the ABC protein genes in *H. brasiliensis* latex through a reciprocal best hits blast search (Blast 1 - *A. thaliana* ABC proteins blasted against *H. brasiliensis* latex transcriptome; Blast 2 - best hits from Blast 1 blasted against entire *A. thaliana* TAIR10 transcriptome).

| Gene name | NCBI accession no. | BLAST *A. thaliana* ABC transporter query on *H. brasiliensis* latex transcriptome sequences (Blast1) | | | BLAST *H. brasiliensis* latex ABC transporter query on TAIR10 Transcript sequences (Blast2) | | |
| --- | --- | --- | --- | --- | --- | --- | --- |
|  |  | *A. thaliana* homolog | Common name | tBlastn Identities(%) | *A. thaliana* homolog | | tBlastn Identities(%) |
| bABCA1 | KM035282 | At2g41700 | AtABCA1 | 73 | At2g41700 | 81 | |
| HbABCA2 | KM035283 | At3g47730 | AtABCA2 | 70 | At3g47730 | 79 | |
| HbABCA7 | KM035284 | At3g47780 | AtABCA7 | 67 | At3g47780 | 65 | |
| HbABCB1 | KM035285 | At2g36910 | AtABCB1 | 89 | At2g36910 | 81 | |
| HbABCB11 | KM035286 | At1g02520 | AtABCB11 | 74 | At1g02520 | 68 | |
| HbABCB13 | KM035287 | At1g27940 | AtABCB13 | 71 | At1g27940 | 66 | |
| HbABCB15 | HQ917533 | At3g28345 | AtABCB15 | 75 | At3g28345 | 73 | |
| HbABCB19 | KM035288 | At3g28860 | AtABCB19 | 89 | At3g28860 | 84 | |
| HbABCB20 | KM035289 | At3g55320 | AtABCB20 | 86 | At3g55320 | 79 | |
| HbABCB25 | KM035290 | At5g58270 | AtABCB25 | 77 | At5g58270 | 80 | |
| HbABCB26 | KM035291 | At1g70610 | AtABCB26 | 74 | At1g70610 | 72 | |
| HbABCB28 | KM035292 | At4g25450 | AtABCB28 | 70 | At4g25450 | 66 | |
| HbABCB29 | KM035293 | At5g03910 | AtABCB29 | 61 | At5g03910 | 61 | |
| HbABCC2 | KM035294 | At2g34660 | AtABCC2 | 78 | At2g34660 | 76 | |
| HbABCC5 | KM035295 | At1g04120 | AtABCC5 | 81 | At1g04120 | 76 | |
| HbABCC13 | KM035296 | At2g07680 | AtABCC13 | 65 | At2g07680 | 61 | |
| HbABCD1 | KF701641 | At4g39850 | AtABCD1 | 77 | At4g39850 | 74 | |
| HbABCD2 | KM035297 | At1g54350 | AtABCD2 | 68 | At1g54350 | 69 | |
| HbABCE2 | KM035298 | At4g19210 | AtABCE2 | 92 | At4g19210 | 92 | |
| HbABCF1 | JX109943 | At5g60790 | AtABCF1 | 83 | At5g60790 | 82 | |
| HbABCF3 | KM035299 | At1g64550 | AtABCF3 | 82 | At1g64550 | 76 | |
| HbABCF4 | KM035300 | At3g54540 | AtABCF4 | 80 | At3g54540 | 72 | |
| HbABCF5 | KM035301 | At5g64840 | AtABCF5 | 78 | At5g64840 | 70 | |
| HbABCG3 | KM035302 | At2g28070 | AtABCG3 | 80 | At2g28070 | 76 | |
| HbABCG5 | KM035303 | At2g13610 | AtABCG5 | 72 | At2g13610 | 70 | |
| HbABCG7 | KM035304 | At2g01320 | AtABCG7 | 76 | At2g01320 | 75 | |
| HbABCG11 | KM035305 | At1g17840 | AtABCG11 | 53 | At1g17840 | 53 | |
| HbABCG15 | KM035306 | At3g21090 | AtABCG15 | 68 | At3g21090 | 67 | |
| HbABCG20 | KM035307 | At3g53510 | AtABCG20 | 75 | At3g53510 | 68 | |
| HbABCG21 | KM035308 | At3g25620 | AtABCG21 | 68 | At3g25620 | 66 | |
| HbABCG22 | KM035309 | At5g06530 | AtABCG22 | 79 | At5g06530 | 73 | |
| HbABCG28 | KM035310 | At5g60740 | AtABCG28 | 68 | At5g60740 | 67 | |
| HbABCG40 | KM035311 | At1g15520 | AtABCG40 | 71 | At1g15520 | 67 | |
| HbABCI1 | KM035312 | At1g63270 | AtABCI1 | 85 | At1g63270 | 85 | |
| HbABCI6 | KM035313 | At3g10670 | AtABCI6 | 71 | At3g10670 | 82 | |
| HbABCI7 | KM035314 | At1g32500 | AtABCI7 | 61 | At1g32500 | 54 | |
| HbABCI8 | KM035315 | At4g04770 | AtABCI8 | 81 | At4g04770 | 78 | |
| HbABCI10 | KM035316 | At4g33460 | AtABCI10 | 71 | At4g33460 | 71 | |
| HbABCI11 | KM035317 | At5g14100 | AtABCI11 | 72 | At5g14100 | 72 | |
| HbABCI13 | KM035318 | At1g65410 | AtABCI13 | 74 | At1g65410 | 79 | |
| HbABCI14 | KM035319 | At1g19800 | AtABCI14 | 76 | At1g19800 | 76 | |
| HbABCI15 | KM035320 | At3g20320 | AtABCI15 | 73 | At3g20320 | 73 | |
| HbABCI17 | KM035321 | At1g67940 | AtABCI17 | 72 | At1g67940 | 67 | |
| HbABCI18 | KM035322 | At1g03900 | AtABCI18 | 75 | At1g03900 | 72 | |
| HbABCI19 | KM035323 | At1g03905 | AtABCI19 | 81 | At1g03905 | 69 | |
| HbABCI20 | KM035324 | At5g02270 | AtABCI20 | 88 | At5g02270 | 78 | |
