# Supplementary material for: Whole-Transcriptome Survey of the Putative ATP-Binding Cassette (ABC) Transporter Family Genes in the Latex-Producing Laticifers of Hevea brasiliensis
Source: PLoS One. 2015 Jan 23;10(1):e0116857. doi: 10.1371/journal.pone.0116857 (PMC4304824; doi:10.1371/journal.pone.0116857)
Supplement: S3 Table — (DOC) [file pone.0116857.s004.doc]

Table S3. The genome DNA sequences of the 46 ABC protein genes identified in the *H. brasiliensis* latex*.

(*The corresponding exons of each latex ABC protein gene are bolded)

>HbABCA1 scaffold1045(170114-201896)

**TCTTCCTTTCTCTGAAAGGATTTTAGGGTTTAACAGGCTTTGGGAGGAGATTGAAGGGGCTGTCACACACGTACGTCGGATATGGGGACTTGCCGGAGGCAGTTGAAGGCCATGCTTCGCAAGAATTGGCTTCTTAAAATTCGTCACCCTTTTGTTACAGCTGCTGAG**GTTCATTTTTGCATCGTTCTCTTCTTTGTTTATGTATTTGATTGTTTTAAATATAACCCATGTTGTAATTTCTTCAAAGTTTCATCCTTTAATTCTTGAATTCTGGTGTCTGTATGATTTTACTTTGAATATCTACCTTCAATGATTATTCTTTTCTGGATTCAATTTGTTTAAGCCCGTGGTTTATTTGTAATAGAGATTTTTTTTTCAGGGGCCTGTTACTGGCATCAAATGAACTATTAGTTTGTTTTTTTTTTTTTTTTTTTNNNNNNNNNNNNNNNNNNNNNNNNNNNNNNNNNNNNNNNNNNNNNNNNNNNNNNNNNNNNNNNNNNNNNNNNTTTTATGCTTGCTTAATTTATTAGTGAGGAATGTTGAACAAGATAAATAACAAATTAATGTTCTTTTCTTGTTTTGGCAG**ATTCTGCTTCCTACTATTGTAATGTTACTGTTAATAGCTATAAGGACACGAGTTGATACCCGAATTCATCCTGCACAGCC**GTAAGTCACCATTGCTCTTCACTATACTTCAGCTTCTTTTTTTTTTTTTTTTTTTTTTTTTNNNNNNNNNNNNNNNNNNNNNNNNNNNNNNNNNNNNNNNNNNNNNNNNNNNNNNNNNNNNNNNNNNCCGGAACCCTTTGGCTAACTGAGAATTTTGGGTCAAAAAGGGAAAGTTAAAGTTTGAACATTTATATTTACGTTGTTTTTTTGTTTGAACATACTTCAGCTTGTTTTGTGAAAGGACAACTCGTGCAATTAGTAATCAAAATGATTTTTGTGAAGGGAAAAAATGGTTTCATGATTGCTTAAGTTTATTGCAATTACTTGTTCATGATGTTGTGAGAAGTAAATGGCACTGTGATTTTATTGGATTTCTCTGCAG**GTATATTCGAGAAGATATGTTTGTGGAAGTGGGGAAAGGAATTTCCCCTAATTTCCAACTAGTTCTGGAATCATTGTTGGCAGAGGGGGAGTTTTTGGCTTTTGCACCAGATACAGAAGAAACAAGGATGATGATCCACTTCCTGTCGATGAAGTTTCCTTTAATTAGG**GTACCTATTGATCTTTTATTTTTTTAAATGAGATTTGAGTTATAATTTCTATTATATACTGGGTGTGTCAATTTTATGTTCTAGGCCATTTTAACTTCTTTAGTTCTTTTGATATGTATATTTTAGTGTGTGTGTGTGTGTGTGTGTGTTTCTTTTTTTTTCAAATGTTATGCTTTTGATTTAACCTCCTATTCTTTGCCCCTCAAATATGAGATGAATCAAACTAGAAACAGAGTGAATTACAATGAGCCTCTTAAACTAAACAGCTAGGAAAATGTTTGCAACATAGTCTTGTGGTAAATAACTATGGCCAAGGTTATCATGTGCAGCTTTTGCTTTGAATTCACTGTCTTTTATGAAATTACACAAATATCTCATAGCGTATAGCGGAAATGTCAATGCCTCCAATGGTCTAGGATTGCTTAATTTTATTGCAGATGCAGAGGCTCATGCAATGATTTCGTACTTTGGTGTGCTTTTATTATGTATAGTACATTATTTGTAATAGTCTATGCTTGCTGTTAACAACCTTATACCAAAGGGAATCATCCTCTAATGGAAATTGCCACAACCAATTTACATCCTACTGTCCCAACTAATGATCTTGCTTGTTTTCCTTTAATCCTGACTAAGGAAAGTCTACCATTTGTCAACATTTCATCCTGTTTTAAGTGCATTTGGTAGAATGATGCAAAGAATTAATATTTCTTAGGTGGGTTAACATTTGGCCCCAAGTTAAGTGGGTTGAAAGAAAGGGGGAAAAGGCTTTTTTTTTTTTTTTTGAACAACCATTTTTGCCACCTCTAGTGTCATCATGGTGGATGTGCACCTCAACTAGACCTTATGCATAAGTAGAAAAGGTTAAAATAATGCAAATTTCATTATTTCTTTAACCTAGTGCAAAACAGGGTTAACTATTGACATTGCAGTTATCATTCGTACCCTGGGGTAGACTTGGCTAGGGCATGTTTGGAACTAGAATGGGCTGGTTCGGTTTGGGGCCAAAACAAATTTTTTCTTTTAAAAAAATAAAAATTTTGAAAAAAATTTCAACAGTTGGATTCGATCAAAACTGGAATTGTAATTGGGGGGGACCCTGGGGGGCCGGTTCTGGTCTGCTGAATCTTGAACTGGAGCTGGCCGTTGGCCACCCTACCCTTTTGGGGTGGGGGGAGGACAGGAGAGTTGAATGATTGGATAGTCAACTGGATGATTTATTTTTTGTGATGCAGCCTCCAGTTAAGCAGTTCTCTCTCTCTCTCTCTCCATGATGAAATTTCTCTCATATCTTCTATCACTAAAGTTTTGCACTTCTTACACTATTAAAAAAAATGTAGAAGTACAAGCTAGCAATTCATATTATTCTACAATATTGTTATTTTGCAATTTGCATATGATTTGTGACCTTTATGGGTACATTTGTTTTCTTAG**GAAGTTTCTAGAGTCTACAAAGATGAAGTTGAGTTGGAAACCTATATATGCTCAGATCTTTATGGTGCCTGCAATGGAGTCAA**GTAAGTTGAATTCTTGATATTCATTCTAATGAAGTTCTTTCACCATGTGGCTGAATGATGATATATATAGCAATTTTCTGGGATTAAAGCATTAACCTTACCTTGCATTATATGCTGATTTAGTAATTGGGGAATTTTAAGAACTATTGTGAAAAGAACTAAGCTGTCTGTCAACTAGTGAAAAACTTGAGTTTTGAATCATTAAAATTTCTGTTAATAGAGTAAAGGAACTATATTTGAGTGCAATCATAAATTTGAACTTCATTATATAATGAACAAATGTTGAAAAGTGTATGCCTGGTGTTTTTCTCAAGATAATTGTCCATGATTAATATGATATTTCACATATTTTGGGTCCAGCTTGTTGGTGGAGAATGAGAGATAAGTGAACAAAATAAGGAAAACTAAAGATGAAGCAAGAAATTGAAAGGATGAAAGAAAATACGAAACCAAATGTTAAAGCATGAAAGTTGGAATTAGAGGTTTGGAATTTGGAAAAAAAAGAAAGATAGAAAAAAGAAAAGATATATGTAAATGTATATTTTCATTCACAAGGGCAACCTCTATTTTTAGGGAGATAAAATGGCTAGTATGCTCATTCAATAATGGTTTTTAAATAACAGGTGTTAGCTCGTTCCATTATGGAATGGCCGTTATTTAAAGGGATTTTGGCTTACTGTTAGTGTTACTACCGTTATTTAAAGGTTTTGTTTTTTTTTTTAAATAGTAAGTGTAAAGGCTGTTATGGTCGTTTTTTAAAGGTAACTGTTACGTATTATAGAAGAAGGAAGAAGAATGTTGATAAATAGTTGCTGTTAGAAGTTAGTTGGGATAATTGGAGGGAATTGCTGTAACATTTTTTTGGAGGGAATTACTTGTATTAGTTGAATCAGTTGCAGCTGACTGATTCTATTTTGTTATTAGGAGGCAGTTACTACTTTGTCTAGAAAATTGGTAGACATTTCTGTGATCAATTTATTCAAGAATGATATATCATCAGTGATACTTCTCTCATCTCTAGATTCTCTTCTCCTCTCATCTAGCTCTATTCTTTTCTCATCTAGCTCTATTCTTTTCACATCTCATCTCTCTTTCTGTTTCTTTCAAAACTCTGCTCAATCTCTATCCTAGGGATTGATTCAGGTCTGTTACCTTACAGTAATGTCCATTACCAACTGTTAAGTAATGACTATTTCAGCTGTAACGGCCATTACCTTTCCAGTTGAGAATCTTTGGCTTTTGTTTACCTCTTTCACGTGAAATGCAGCTGAGATAGCCTTTTTTTGGGTAGAAAACTATCTTGGTTCATTTATTTTAGCACCTTTTCATTTTCTCTCTAAGCAACTAAGCCATTTTCTTTCAACTTTCTCTCAATTTTCTCCACAACCAAGTGAAGTGAAATCGTTCATTTCCCTACAAATTTTTGAGCTCAAGAGTATAGATCATCAAATAGACAAGGTGTTTTGAAGTGAAGTAAAGCAAAGGTTGAATTATTGTTCTTTTTCTTTCCCCCCCCCTCCTCCTTAATTTTAGCTTTTTTTTTGAATCATTACATAAATAATTAAAAAAATAATATCAAAAAAGTTTAGGCATTAATAATATTTTGCAAGTATAAATTGATTAATACTATCTACTTAGTTACTTTGGTTCTAAATTGATTAATACTAATGATGTATGGGAATTATGGATTTAATGTTTTTATGTGATATATTTATGCTTATTATTTAGTTGTATATTTTGGTCTTAGTTATATCTTTGAATGTTTATTCATTTCATGAATTTTGCAAATTTTTCAAAAAAATTTGCGTAGCAACAAGCCGTTACTATTACGTTACGACTGTTACAGTACTATTTAGTGACCCGCGAACACGGCCACAACGACGATTTAAAAACCATGGATAGAAACCTATTCTGAATAGGAAGTGCATATGTAACAGGATTCCACAATGTCAAAAGTTCAAGCAAAATCCTCCTATTCTAGAATTCCTCGAATTGGACAGGGAATGTATATTATTGTTAGTATCCTAGACTTGCTTAGCAAGCTAGTGCTTTTGTTGTATGGTTGCAGGCCTATTAAAGCATAAGGCTGATTGATGCATGGTCAATTGAAATTATGTTTGTGGCCAGTTGATGCAGTACTGGAAGTTAAGGAATTATGGTCACTGTGAAATTAGGAAATTTAGGTTATCATTATTTAGGATTTTTTTCTTTGAAAATCAACAGTTTAGATGTTGAAAAAAACAGTTTAAAAAAAGTTATTTAGTTGTTTTGGATTTCTTATGATTAAACTAAGTTTTAATGCCCCTCTAATTAACATATTTAAGGAGGTGCTATTCCATTAGTAATACAATGCAAAAAATTTATTTATACATGAATATAAAATATTTACTTCTAGGTAATACTTCCTATTTAAGGAATCATAACTTTAAAAGGTGGCAAAAATTTCGTGTTTGTTACTGGTGCATGATATAGATATGTGGTAATAATTGAAATTAATTAAAATTGTCTAGTAAATTTGTTAAGCTTGTGGATATCCTGTATGTATCACCTAGAAAAAGGAACTGCATCTGAACTTTTGCTGGAAGCTTACCATGAGTAATGAGTTTAACTGAAGCTTGATTTAAATGTAAATAAACCAAGCTTGCAACTTGTTTTGCTTGAATTTGTTTCACATAATTATAAGCCTTATCTAATAGCAACTATCGCTGGAGTTTGAGAACAAAAATAGGAAAATCCATTTGTCGTTTTAGATAATAAGATGAGGGCAAATGCCATAACAGAGCCGATTAGCGTATATCTTTTATTAACGTAGCCTGGAATAAGTATATCTTAGTAAACAATAGATATCCATTATTCCTTTTTTTCTTCTTCATTTATATATTTTGAACTCTCCTCTTCCATTCTTTTGTACTTTTCAGCATGCTTTGAATGTGTTTCTATCTTTTGTGAGAACTTCAAAATATTGAACTATGCAAAGTATTTTTCCTCCATGAAATTTAAGTATGCCACACAATACTGTGATTTTCTAGAACTACTTAAAAGAGGTGTTGTCTCATTTTGTAGAGTTATTATTCTATTTTATCTTACTGAAGTTACATACAGATGCTACTAGATGAGAATCTTTGTTTTCATTATTAATATCATAATGTGTTGATTTTCATAAGAACTGGCAATATCGTTCTTATTTCACCCCATTGTTGAACTTAATGGATTTCTGTTCTCTTTGTTTAG**GAATTGTTCAAATCCCAAAATCAAAGGAGCTGTTATATTTCATGATCAAGGTCCTCAGTTATTTGATTATAGCATACGTCTTAATCACACGTGGGCTTTCTCAGGGTTTCCTGATGTTAAAACCATCATGGACGTAAATGGTCCTTACCTAAATGACTTGGAATTGGGTGTTAGTACCATACCAACAATGCAATACAGCTTCAGCGGATTTTTCACT**GTATGGGTGCCTATTTCTTTTCCCACTATTTTTGTCCCTTTTCATATTTTATTGTGGATTTTTAGTTTTTGATGAATAATTATATGTAAGTGCGTGTATTTGAGGTGGTTGTGTGTTTTGGATATGTACTGTTATTTTAATGACTGCATCATGCTATTGAAGGAGATTTTTGGCCTTGGTGATAATTGTGACTGATATGTAAATGGTAATCCATGCTTTATAGGAATGTATTTCCAAGAAGTGGCCGCGATTTTTAAGTTGGTGATTCCTTTGGTTGTATTCTCTTTCAGCCCTTTGTAGAAATTCTCTAATTTGGCTTCATTGTCAAATGTTTTTCTGTCCAAATAATAGTTGGAAGGTTGATAAGTTCAGTTATAATCCCCCCTTTCCCCAAAACTCGTTTAGATACACAATTATTATAGTTGATGCTTTCATGATTTCTGCAG**CTACAGCAGGTAGTGGATTCATTCATAATATTTTCTGCTCAGCAAACTGGGACTAAGGCAGCAGGTGGACATATAGAGCTGCCATCATCCAATTCATCTATCTCTTCGTTGCTCAAAGTGCCGTGGATGCAATATAGCCCTTCGAAAATTAGAATTGCTCCCTTTCCAACTCGTGAATACACTGACGATGAATTCCAATCAATTGTCAAGACTGTCATGGGAGTACT**GTATGTGTTTCTTTTTTCCCCTCAAATGTTTCTTTCTTTTCATCTGTTAGCTTCTATTTTTCACCCTTTTTGTAACAACTATCTTTGGTCTACTGACCTCTGAGCAG**CTACCTGTTGGGATTTCTCTACCCGATCTCTCGCCTTATCAGCTATTCAGTGTTCGAGAAG**GTTTGGTAATGACTGATAGTTGGCATAAAGCATAATATTATATCTGTGCACTTTTTCTTATTTCATCTTGTAAATCCCCCCTTTTATAGTTTAAATTTGATAATGTATCATCATGTGTTATTTATTTTTTTAAAAACTTTTATATAAATCCATTATTATTTTTGATATTTCAG**GAACAGAAGATAAGAGAAGGTCTCTACATGATGGGCTTGAAAGATGGAATATTTCATCTCTCTTGGTTTATTGCATATGCTTTACAG**GTGAATTTTCCATGTGTTAGTTACGTCTTTGAATCAAATGACCAATCTTGCATGAACTTGTTTTCTCTTCTCAAAGGGCTTTGGATGATAATTTAGGGATGAGGATTATGTGTTCTTGGTTATTACAAGATAAATGAACATAGATCTATGTGTGTATGATGCCTGCATGTATGTATGAGCTTTGTGCATGCATGTGTTATTTGTATTTGCAAATCTGGTGTTTCAGTTGGAAGGTTGAATTATGAAATGGGATTTATGTTTCCCTTGTCTATGGCTCTTTGATGGATGCTAGCTACATACTTTGATCAACCCCTGGAAATTTAACCATTCAAGACAAAATAGTTTTGTGGTATCCTGCAAATTTAAACATGAATGCTCTTCTGGATAGGCTTATGAATTATTCATCCAACTTCACCAAAGCTTGCATAAGTTCTTTGGATGTTTTGCTGGTGGTGTGATGGAGGCAGTGTTAATGGTGATTATTTTTATGGTATCTTGGTCCTTCCATATAAGCAATATTTAATCATTTACCAGGAAATGTGATTGATTTATTGTGTTTTAGACCCTTTGAAGAAAGGGATTTTGAATGATCTATCAACGAAAGTAACTTTTTTGACATGGTACAATACAATGAATGTTCTATTTCTATATTCATTAACTTGTGCCTAAGGTGTGGGATGCAGAGGTAATCCTAGACCTATGTTGCAGTTTGCACCACAAACCTTTTCCTAAAACAAATATGATATGTATACTTCTTTATCAAAGAAATTGGTCAGCCAGTTGAACTATGTTTAATTTGCAGTGTTTTTGGCTTTTGGCTCTTGTTTCTATGGGTTGGTATTCTGGTTTATTTGCTATCAGAAATTAGGAATGTATGAATAGTCCATCTTTAACAATGGTCTATCTTTTTTTTTTTTTTTTTTCTTTCCTCTCTTCTTCTTCTTCTGCTTTATTTTTTTTCTCCTTCTCCATTCTCTCTCTTTTTATATTGTCTGTTCCTCATTTGCTGGTTGAATCTCCTGCAG**TTTGCAATTTCATCTGGGATTATTACAGGTTGCACCATGAATAACCTTTTCCAGTATAGCGATAAGTCGGTGGTGTTTGTGTATTTTTTCTCCTTTGGACTCAGTGCAATTATGCTGTCATTTTTGATCTCTACATTCTTCACTCGAGCAAAAACAGCTGTTGCAGTTGGAACCCTTTCTTTTCTTGGCGCCTTCTTTCCCTATTATACTGTGAATGATGAGGCTGTCCCTAT**GTAAGCGCTCATGCTATGAATAATTTACTTTATGCTGATTGCACTTGTTGCAACTTCTCATATTTTAGAATGCAGACGGACGTTATGTAACATTTAGTTCTTATAG**GATACTGAAGGTCCTTGCTTCTTTGCTTTCACCTACGGCTTTTGCTCTAGGATCGATCAACTTTGCTGATTATGAGCGTGCTCATGTTGGGCTTCGTTGGAGCAACATATGGCGA**GTATGTTCTACATTTGTGGTTTTTGATGCCATATATATATATATATAAAATTTTGGTTCTTCATTTCTAAAACTCTAACCAACATTTGTGTAG**GGGTCATCTGGAGTAAATTTTTTGGTCTGTCTCTTGATGATGTGGCTTGACACGCTGCTATATTGTGCTGTTGGTCTATACCTTGACAAG**GTATTAACCTCTGTTTTGCAGATATCTTGGTACCTAAAATTGTCGTACATTGTGTTGTACTGTTTTTGTTAGTTCTATTTGAATATATTTTTCCCCTTTTTTTTTCTTTTGTTTTCCTATTTCCTCTTAAATAACTTTGGGCATCTGATAGGAGAAACCTTGTATCTGCACAAGGAGGTTTTAGACATTTATTCTGTGATTTGAACACACACACACACCCACACACAGAGACACACAGTGTCAGAGAGAAAGAAAAAGCCGGGGGGTGGGGGTGGGGGGTTGAGCCTTGGAGCAACATTAAGGTTGCTCCACTATGACTTGGAGGTGATGCGCTAAATTATGGGAACAGCCTTCTTATAATTCAAAAGGAGAGCTATGCATATTTGATCTTTCCTCAAGCTAAAAATGTGGCATGCCTTGTGAGCCAGAACTGCCTGTAATCTCTTGTCTGGTAGGCTTCTTGCTTAGTTACACGGAATGGATTGTGGCTGCATTATTTTTAAGTAATTATGGGAGCAGCTATCATGAACTCTATGTTTTCAGAAAAAAGCACCTCGGATTCAATTTATTTCCCTTCTATTTTAATTCTGCTTTTTGGCTATGCTAATTTTTGTTTGGCAAAACGTACTGATCTTTGTATGATCAG**GTTATTCCGAGGGAAAATGGAGTGCGTTACCCTTGGAACTTCTTATTCAAGAATTGCTTCTGGAGAAAGAAAAGTATGATCAAACATCATGTCCCAAGTTTGGAAGTTAAACTTAACGGCAAGCTTTCTAACTTGGGAAATGACACTGTTGAACCTGCTGTTGAGTCAATAAGCTTGGATATGAAACAACAAGAACTTGACAACAG**GTATATACGCTAAAATTTTAAGGTACTCTTTCAAATGTTAATATGTTTGTTCTCTTCATATTTATTTATTGGTTTGTAG**ATGCATCCAAATAAGGAACCTGCATAAGGTGTATGCTACTAAAGGTGGGAGTTGTGCTGCTGTTAATTCACTGCATCTCACCTTGTATGAAAATCAGATTCTTGCTCTTCTTG**GTTGGTTCTGCAACTCACTTCTTCACCTTCATTTCTATTTCATGCTTGTTTTTTCATAGTTATTTTGGACTTTGAGAATATGCAATAAATCAGATCAAAGATTTTCTCCCATTACATATATATCTTTTTGATATCTGCTGTCTTCTTCAGCAAAGGATGCATTTCTTACAAGTGGAGCCTTGTAAATACTTTAAAGTAGCTCTATAGTTCAGATTGCTTATATGTTCTTCTTTCGTTGTATATCCTGTTATGGAACAATATGTAAGAGCCCTTTTGTTTCATAAGCAAATGAAGAAGATAGAGAAGAGGAGGAAGGAGAGAGACAGAGAGATGGTGTGTTTTTTGGTCTATTTTTCTACCCTACCCCCCTTCTCTCTCTCTCTCTCTCTCTCTCTCTCTCTCTCTCTCTCTCTCTCTCTTTTTTCTTTTTTAAACCTTTGGTCCAATTTGTAGTAATAGCCATTGCTTTTTAGTTTGTTAATTATATACACAACCTTTCACGTTGGTGTCAGTTGTAACCAAATTCAGGAGAGCATGTGCCAAAAATCCCAAATAGTTGACGTGGCTTATATTCTATTCATATTTTTTTTTATAAGCATATTTATGGTGAGGGAGTGAGGGAGGGAAGAAGATGTAGAAGAGAAAATGTAGTGAAAAGAATCCACAATTTTCAATTCCACTTTTTTAAATTTTTTATTAAATGGTAATTAGTAATTTTTGACAGGTACTTACACTTAAATATACCATATCATGTGCTAATATTTTTTTTTTAATAAATTCATATTAGTAATTTGAGAGTTATAGAATCAGCTCTTCTAGGTGTGGCTAAATGGACATCAATGTTAAAAGTTTCATAAACAATTGACAAAATTAAAATGATTTTCTATAATTACAAACTGGATAAACAAATTTGGAATTTTTGGGTCACTAACCCTTTTCTAATTCTCCTTTTCCTTGACCATTATTTGAGCGTCTTTATAAAATCATAATGGTGAGCTTCTTTTGAAGTTCTTTTTAGACTATATTGTATCTTTAACATCTTTAAATGACAAAGGCATAGCCCATATATACAAAAAATAATGTTGTTTTACGGAATTATTTTAGAATTTTTTGATTTAAGATTTAATTGTATTACATTTTTCATTTTTGTAACATATACTTTTTTATTTTATTGAATCATGTAACATATACTTAATAGTAAATTTTTATACGTTGTTATTTGCACTAAAAAAATTGTTACCGCTATGAGTCAATTATGAGTTATGACTTGAAAATCATAATTTAGTTTGGTTGTTGAGAATATTGATATTTGTGTATCTAGTTAAATACAGGTTAAACTGAAAATTTGATTATTTCTGCTTATTTTTAAACTTCTTGGTTGTTCATATGCTGATACATTTCCATACTGACATATGGTCCGACACTACAACTTCAAAATTGCAACCTTAAAACTTCAAAAAGTTGTATTTCTGTATGCATGTCTGTTGATAACCAATGGTTGACCCATTTTTTATTCGTAAATTTAAGCCCGTTTTATCTCCTTTAAATTTGACCATTCTCTTTTCCTTGCATGATGGAATGAACCTCAGTGGATGTGTGCTATTGACTGTTGTCCAATAGTCATTCTTCTCGTTCTCTTGCCTGAGCTTGAACACATTTCCCGCCCTCTAATAACATATATAATTTCAATATCAGGCTAGGACAGGCACTTCTTACTTCCAAACATCACAAATACTAATTCTAGCTTTGATTGTCAAGATACTTGGTTATTTCTCACAAGCACTAACAAACCTCAGGCTCCTCCTATGCCTTACATATTAGAAACGGTATCTTCTTATTGTGAATATTAACTAACCTTAGATAATGTCAAAGAATTTGATAGTCCAGAAAAGAATATATCAGGAAGCTAAGGTTATTCATATATGCATATGCTATTTTGTATGACATATAGGTGCATATGTTTTGGTATTATAGCTGCCCCAGGGAAAATAAACGATCACTTATTTTCTTTTTGAAGAATAATTCTTTTAGGTGTGCTGATATAATGTTATTGTGTTCAAG**GACACAACGGAGCGGGCAAAAGCACAACTATTTCAATGCTTGTTGGTCTTCTTCCTCCTACTTCAGGGGATGCGTTGCTGTTTGGAAAGAATATTCTGACAGACATG**GTAAGTAATTTTTCTTTTCCTGAAACTATTCCTCCCCCCCTCCCTGATGCTTTGAATTCTGCTTAGAAATTTTATTCCATGTAAAATTAGGAAGATAAGTATTTCATTTCTTTAGGAAGTTTATTACTATTATTATTATTAGTAGTAGTAGTAGGAGTAGTACTAATAGTAGTAGTAGTAAGGAAGTATTTTATTGTAAGTTTACTAAGTTTAGTATTTCTAATTAGGGTTGAATTGGGGTTCTAGAATCCTAGTGTGATTGGGATTTTAAACTCTATAAATAGGGTTTAGTATTTATTTACAGACTATTTGTGATTTTGATTATTGATTTGAGCAATAAAAACTGAGAATTTGTTTCTCTGGAGAATTAGTTTCTCTTTCCGTCAGAAGTTGTTTCCTTTGATTTCTAATTTCCTGATTCTCCCCCTTTTCTTTTAATCCATTTTGCAAATGGTCTAACATTTATGTTATAGATGCTCTTATAGCAATTTCTGGCACGCTAATCACTTTTTGCCTGTGTGGTTCCTTGGTGGTTTGAATGGATTGTCTAATAATTGCGTTCCACTCTGTTCGGGGATCCCCTAAATTAAATTTGGCTGCCTAATATTTTTTCACTTGTATATTTATGGTTTTTGTTTACAACAAATTGTTTATCATGCATTTTTCCTCTTGGAGTGGGTGTGTGGATTGGGGATATTGTGAGCCTTCTTCCCCCAAAAAAAGTTGTTGCTCTTTGTTTTAGGCTATTGGGATTGTTTCAAGCAGGATGTGTTCACTTGGAAGCTTTCTTTCCTTTTTTTGCTCCTTCTGTCCTTCCCCACACCCCCTCCCCCAGCCCCTCCTCTCCTTCTGCCCTTTTGCAGTGTCGGGTTTTCAGTTTTTGGGGCTTAAGATGTAAATCATTTGTGAGCTTTTAAGCTATTTTTTTAAAAAAATTTTTAATTCTTCTATTTATTTTTTCTTGAATTTTTTTGATTAATATTCTGATCAATTTGTTATCTTTTGTAG**GATGAGATACGGAATGGACTTGGTGTGTGCCCCCAGCATGATATTCTTTTCCCAGAACTGACT**GTAAGTAAGAGGGAGTTTTCATTTTCGAACTTGTGTCTTCCAACACAGATTATGCTTATGTTCTTAAAAATGGTGATCAGCTGTGGTTTGAAGCTAGCTGGATATTTATACAGACAAGAAGTTTGGGACTTTTTATTGCATGCTACAGACTTAAATTTATTATTGTATTGCATGTTATATCTCTTTCTCATTAGCATGAATAGTCTTCCAATATAAAATCTAATAGAATATGGAAGAAGTACAAATCACTGTCAAAATGCTTTGACATGCAGTAAGAATAAGTGCAGGAAAAAAAAAGGAATGAGAATAACATGTGGCAGTTTTCACGAATTTAACTTAAATTCTTGTAATAAAATGTATGATCAGATGCGTTGTTATTGGTTTGTGCATTTGGAATATAATGGAATGGTTATGATGCATTTGTGATATGTGGTGTGCTTCCTCATCCTTGTGAAACTTGAGAAGGTGGGATAGGTGGGCAGTGAGTAACTGGTGAAAGTATGTTATACTGTGACTATTTTTTGAGAGATGACCAAATAATATCATAAAATTTTGAGATATGATGAGATATTAAAGAGGAATCTACTTTTTGTTCTCCAGTGGGTGGATTTTCTGAATATTCAGTTGTCAAGCATCAAATATTTTGATTGAGTGAAGTTCAAAGATTCAGATATGTGTTTGCTGACAGATCATGTAGATTTGATAAGAGCTGATTCATTAAAAAGTGACTCAATTTGGTATAGATATGAGCTTACTGCCAGATCGATTATTTGTTGTTTTTGGGAAAGTCATTTATCGTATATCTTTAGCCCTCCCACTGTCATTTTTCTTTTATTTAGGGTAGAATGTTAGGGTGGCTGGAAGGAGCTTTCTCTGTGTGTGCTTGGGGTCTGACAAGTATGTATGGATGTGAAATCTGAGTATGCTTTTTGGGTTGGGATTTTCATGCTATTTCGTGTTTGTTATCAACAAAGTTAGTTCACTAAAACGTACTCTTCTATGATGCTTTACCTTGAGAATCAGAATTGATGGAATTATTTCTTCAAATTAGGTTGGCTTTGCCATGATTTGTGAACTTTTGAATGAGAAATGTTAAGGTAGTTAGGTTGTAATTGTCCTGGATTGTAATGTGTGCCAATAGGTCAAGTGTAAATTTACTGGGGATTAGTTAAATGCTTTGAGCACCATGCTTCCTATTGCAACATAGAGAATGGAGTTTGGATTTAGAATGGAAGGGATACCAGAATTTTTATAAGCATTTTCATTCACTCAAAGTGTTGCATGGAATTCGTATGCTTTCTTTTGGCTGTCCTCCTTTCTTGTTCACTTGATTGTTATCATAACTTTCAGCAATATCAAGAGTGCTCTAGTTGTGTGGAGGATTTAATTGATATGTATTACTTTTTGATTAAGGAAAAGTTAATTTTCTTGGAGGGAGAAGAAAATGATGGAATTTCTGCTTGTCCTTATTTGGGTATTTTATTATTTGTTCCATAACATCTTTTGTGAATTTTGTTTTTCTGACAACTTTATTCAATAATACTCTGTCTAGAACTTGTGGTTCTCTGCATATACTTAATTAACTATTTGAGTAGTTATTCCTAGTAGATGAAACGTTGCTGCTGCTGCAATTTTATTGCTGATATTTTCTTCTTTGGAATGTTAATAAATCATCAATTTTTATTTTAGCTGTAGTTTCCTATTTAGATTGCATGGATTTGTTCACAACATATACATTGAATGTCTCTTTCTTGAGATGCTAAATAATGACTTTAGCTGATTTGTAGAAGCATCAATTAACCAGTTAATTAATCTATATAG**GTGAGGGAGCATTTGGAAATGTTTGCTACGCTGAAGGGTGTAAAGGAAGAGATTCTGGAGACCAGTGTAACTGATATGGTTGATGAA**GTGAGTTCTCATTCATTTAATTTGATTATTGAACACATATGTTACTAAATGTTTGAAGTTGTTTTTGTTTTTGAATTGTGATTATGACATTCTTTTGCAAATTTGGACCAGCACATATATCATTCGTCTTTTCTTTTTTGAACTAGGTCTTATATCTTTTTTGTCATTGGAAAACCTTATTCCTGCTTTTGTTTTGGAAGATTTTGGATTATGAAAGGATCAACACTTGCTAGCTTTCCTTGTAGTCTCCATTGCCGATGTTAGTGGTTCACGATATAGCTTATAAATTTTTTCTGTTTTTAGTTTGACCTTTCATTACTAAATGAGTTACAGAAAGAGCTTTTTGAAGATGGATTTCTTTCTACCTTTTCTTATTCATTTATAGCACATTGATTGAAATAGCAGTTGTGTGTAATAAAAAATTGGGCTTTCATGGCCATTATGTTACGGATGTGGTCCAATAGTGTGTTAAATTAATGGAATTGCTGAAGGATTTTACTAAATTTGATAAGTGCAATCAAATTTTTTGGGATAAAGGCTTAGTTGAGTTGAGTTGAGTTGAGTTGTGCAATCAACTGATGAGCATAAATACTTAAGAGACATTGAATGCATGATATATATTCTCAAGAAAATAGCACAAAACAAAGTGGAGAATAAAAAGCTAACCTTAACTCCATCAAAAGAGTATCTAAGAGGATTGAGTATTTGGCTGGTTTTGAGTGTTTTGGTCTCCAGAAATACTGCCATAATGCTTTCTAGTTACCGTGTGAGATGTTACATATTATAGAAAGAAGTATAAGAGTAAGGATAGGAATTAATGTACAAGATTCTAGAAGTTTTAGCAAGTGGGGGAAAAAGTTAAAACAAGTACTAACTGATTCTGTTGTATTGATTCTGTTATATCTGTAGCTAAGTTGTTAGGCAGTTATCAGCTGAAGTTGCTAGGGCAGTTACTACTGGAACTTAAAGCTCACTGTAGCTCTCTTGTAATTCTAATCCAAGAACTATCAAGAAATACAATATTTTTCTCTTCTTCTCTCTATCTTTCTCTCTTCTATCTCTATTCCATTTCTTTTCTCTGTTAGGATTCCAAAACCCTAACATGAGATCAAGTTAGAATTGTATGTAGTAAGAGTTCTGGAGGCTTTAGGTTAAAGAAATGGTCAAAATGGTGGCTGGGAAAATGGGGGGCCCTGGCTTATAAAGAATTAGCCTTTCTATTACAGTTAAAGGCTGTGGTGACTGTTATTCTATTGTTGATTGCCAGATAATGGCTGTTAGAATCACATGATGGCCATTACAGCTGTTACAACAGCAGAAAGTTTTTCAATTGAAACCATGATATGCCTTGTTCTATTTTGGTCAATTTAGTTAAATATCATCATCATCATCATCATTGTAATGATCATCATCATCATCATTTTCTTATTTTTGTTTTTTGTGGGACATATAAGCTTCATTCTTTGACACATTTGATATTGCTTTTTTGTTTTCTCCCCTTTTCTGTTGTGTTATGAGATTGGAGAGTGACTTATTTAATTTCAG**GTGGGTTTGGCTGACAAAGTGAACACTGTTGTGAGGGCTCTTTCCGGTGGCATGAAAAGGAAATTATCTCTTGGAATTGCCCTGATAGGAGACAGTAAG**GTGAGAAGCAAATTTGAAGGCATGGTATATCCTTATCTTTGTTGAAATTTGTATTTATTATTTCTCACCATCTGTCACAG**GTTATAATTCTTGATGAACCTACCAGTGGAATGGATCCATATTCGATGCGATTGACATGGCAATTAATCAAAAAAATAAAAAAGGGCAGGATAATTTTGCTAACAACCCACTCAATGGATGAAGCTGATGAACTAGGAGATCGGATAGCTATCATGGCTAATGGTTCTTTGAAATGCTGTGGAAG**GTAAATTCTTTTAAACTACTATTTCATTCTATTTTCAGCAATTGCTCTTGTTGTTCTTACTGAAAATTTTCTAGGAATGAAGTTAGATATGACTAGCTTTTTTATCATTAGATATGACTATAAATCACATATAATACCATGTGTTTCCTATTTCCCTTTTTTGGAAACTAGAATTTTACAAAAATTCAATTGATTTCTTTTTGGTCAATTTTCATGTTTAGAATAAAAGAATTCAATTTTTTTTTAACTTAAAAAAAAAATTCTTTTAAAAGAAATAGAAATCAAGCATGATTGTGTGAGAATTCAATTGAATTCTTTTTTTGCAAATGATTTATTCCAAAGGAAGTCATTCAGAATTCTAATTTGAAGGTTTGGTTGTTACTTCAGACACTAATTGCCTGTTGCATGTTTCTGTATTGAACAGTTTTCTTAATCATGAATTTTTAGCTGCATTTTTTTCAGATGTTCTTTTTTTACTTTTAACATTTGTATTGCGAATGATTTTTTTTCTTCATTATTACCCATTGCCTTCTCATTCTTTTTATCAATAAAGGTAGAGATGCTGAATGAACTTATATATAATCCCTCCGATTTGTAAATGAGTTTCATATTTTAATGTAAAATGAAGAGAATGAGACAAAACATAACATTTTCTTTAAGTTATTCAGATGACTAATAATTGTTTTTGAAGAAATTCTCTGACAACATAGGTAATGCTTTTTCTGGCATAGTTTCGGAGTAAAATTTAGTGAAATTGTTCAAGGATTCTTAGTTGAGAGGGGGTGAGCTGTGCTAAATGAGGTGATGGAGAATTGAATTCTATCACATTCTTGATAGTTGGGAACATGGTTGTTAGACCCGGACCGGTTTAATTGGGATTCGGACCTTTGGTCTGGTATGGTTCACTCACAAACCCTCTTTTTGGTAAACCTGCCTTGAAGTGGAATAAACCGGCCGATTAACTGGCGAACAGTAAACCCGGCCAGATTTAAACTAGTCAGTTGGGTGACTTGGGTCATTCATTAAAAAAAAGAAAGAAATTGAATCATGTATGCTGGACAGGGCTCTATCCATGGTTGATGCAGTGTTCATTGAGACTTCATAGCACTCCAACTACTACCAGAATTTATCATTTTGCTTCCCTTATATATATAGGGTGAGCAAAATTCAGTTTAAACTGAAATGACCAACTGAATTGAACTGAATAAGAAATTTGGTTCAGTTTATAAATTTAAAAAAAAAATCAGTTTATTCGGTTTGGTTCGGTTTTGAAAAAGAATTTGGTAAAACCGAACCAAACCAAATCTATAAAACAGTGTGTTTTAGGTGGAGTTACTTATGTCATTTCTTCTCCTTCAGCAACTAATCTGCTTCTCAAACAAATGACGCTGCCCTCATCAAATCCCTTTGCCTCTGCTCCTCACTTCCTCAAACCCGTTTGGCCATCTCCCTCACTCCCTTCCCTTGAAATCTGTGCCTCTCGAATTCTTAGAAATCTTCTTTTTCCTTTATCTCAGCAGTCCATATGTAATTCTTCTCCATTCCTCTTTGCTTCTTTGATCTTCTCAGTCCACTTGCACTTCCTCTCCAGAAATGACAGCAAAAACATTGAAGCTGATTGTTGGGACCTGTTTTAGAAATGCGCAAGAAAAGGAAAAAGCCTTTGATGGATCTTTATTAGATTTCCTCAGATCTTCTCTTCTAGAAATTAGACACCAAAATATGTCAAGCCAATGAGAAAGAGACTGATGAGGGATTTCAAGAGATTCTAGGACGATGGAGGTAGGTCATTATATATGTTTTTTCTCACATTGTGTTTTTCTTCTTGGGTTTGTTGACTTGATGATACTCTTGGGATTATTTTCATGATTATTATATTTTAAATAATTTACCAGAATGGAGGCGGGTAATTATGATTCTTCCTATCTCTACCTTAAATTATTTAGGTAGATTATTTTTATATATTTGATTGATTATGCTTGAAATTTGAAAATTAATTAAAATTTTCTCTCTTCTAGGTAGTTCTTATTCCTTCAAATAAAGAGGCTTTTATATATATTTTGACTGTATTTATTAAGCTATTATGGATTATATTTATTTTTTATTTCTTAAAAAGTAGGAGTCTGCAACATTTGACAAAATCTCAAGTGTTACTAGTAATTGATCCATTCTTCCAGTTTTGAGTTGCTGTAAGTGTATATTTGTTTATGGAATTACGGTTGTTGGGCTGTATTTATGGACTGAAACTTTGTTGGTTTCCATTTTATATTGCTTAGATTGAATTAGCGGAAAATTTTTCTTCTTTGTTGATGGTCTTTCTGTGAATTTGCTGTTTATTTCCTGTACTTTTGAAAACCAAATGACCAAACCGAACTAACCAAATTTATTCGGTTTGGTTCACTTTTTCAATAATTTGGTTTTTGGTTCCGTTCGGTTCCCAACCAAACCGACTGTATGCTTATATATATATATATATATATATATATTAACTTCAAACAATTGTAAATGAAAAGTAAAAGTTTCTCCGTTTCACTAGCTTCTAATTTGAATTATTAATCAGTGTATTAACATGAATTCTTTTTCTATTTTATTATTTTTTATATAACAAAAAATGTAAATTTTCTGTATTTATTATTATATGAAATTTTAATGTATTATAATTTATTTTTATGACAGAAATAAATTTATTATTTTGAAAGTTTTATTTTAAATTTTGTTATTATAAAATATTATCATTAACGTTTTAATTAAATATTAAATTTTTTAAAAAATTATTTTACATATTAAACTTTAACTAAATTTATTTTAATTATTAATTACAAAATATATCAAACTATTAATTATAATAGCACATTGTTTACATTATTATTTTTAAAAAAATTATTAATTTTATAATATATTTAATATTTTTAAATTTAGAGAGAGTGCTGGGCAAATTATGTATTAATTCATTTTAATCTGGGCTTATACAAGTGGTTGAGTTGCTTTACACATCAAAATGACTAGCCAAGACAATGTTGCAACCCTTCCACTTATATTCACTCTTTAATTTTTTTATTTTCCCGATGTGGGACACGTAATCCCAACACTCCCCCCTCAAGTGCAACCATATGGTTGTCACTTGACAATTAGTTAATATTTAACCCAATATGGGCTGAATTATACAACCTTATGAGCAGCTGAAACTCACAACCCAGTCCGGGCTCTGATAACATATTAAATTTGGAGAGAGTGCTAATCAAATTATGTTTTTAATTCATTTTAATTCGGACTCATGTAACTAATTGAGTTACTTTACGCATCAAAATGATTAGCTAAGTTAATGTTGCAACCCTTCCACTTATATTCACCCTTTAATTTTTCTATTTTCCTGATGTGGGATATATAATCCCAACAAATATTATACTGGTTAAACCCTGGTTTGACTCTGGACCCTTAACCCTTGTCTTCACCGGTTCACTAAGCCTTGATTGGGAATTCAAGTTTGATGGAGGTGAGTTCTCTTGAAGTGGTAGAGATCAAAGTGTCTCTTTGGGTATGTTGGGAGTTATTAGCCTTGAATATATTTAAATATTTTGTACATGATAGCCTTCTAACTACTTAAAACTTGTCTAAGTTGAGGAAGGGATGAATAAAGGAGGTGGAATCAATTCTCATTTTTTAACTAATTAAAATTCCTTGTCTATGTGCTTTGTGATGAAAAGGAAATAAACAAACCTTAAATAGTCTTTGCTTGTTGAAGATCATATGGCTTCATATGTCTGTTGTTGACATTCGACAGTTGGGAATAATAGCAGCATTTTATTTTAAATCTACTTTAACTATTTTCAGTGAACACTAAACAGAAATGTTTGGTTCTTCTCTCTCTCTCTCTCTCTCTCTCACTCTCTTTTTCACATTGTGTTATTGAATGTGATTCATTTCATGAATCGTATGTTTAAGAGGTATTTGAACATGTCTTTTTTTCTAAAAAGAAAATGATTGTGAGCATGATATGGCTAGCATTGCTCATCATTCACATTTTCAATGGTTATTTTGCAG**CTCTCTCTTCTTGAAGCATCAGTATGGGGTTGGTTATACTCTTACTTTAGTGAAG**GTTTGTCCTATTTGTGATGGTTTAGTTGTAAAGCTTCTTTTGTGACACAATGTTCTCTTTTTAATATCACTGAAAATGGCCTTATTGTTAACACTATATTCTTTCAG**TCTGCACCTACTGCCTCTGCAGCTGCTGATATTGTTTACCGCCATATTCCATCAGCAATATGTGTGAGTGAG**GTATCATTTAACTTTTATATCTCAAATTGGTATATTGCATAAATGGGTTTTATATGTGCAAGATATATATTTTATGTTGTCTAATCTGATGCCAAACACCTGTAATGATGATTGCTACTTCAAGAAGGGATCCTCATTTGTATTTACTGTTAGGTTTGGACATTTGATACTTCAAAGAATAGAATATTTGAATTTTAAATTCAATTAAAAATTTTAAGTTAATATATTATCTTGGATATCTTTATGCTATGGATGTTACATATTATATGAACATCTGAGTTCTTACCCATATGCGTTGTTGATTCCTTGTGTATAATATACAGTTGTACTGTTGAGCTTCCTTTTTAACTTTCATGTGCTGTTTGTTTTGCCTCTCCAG**GTTGGAACTGAGATTTCCTTTAAGCTTCCTTTGGCATCTTCCTTGTCCTTTGAGAGCATGTTCAGGGAAATTGAAAGTTGCATGAGAGTCTCGAAGTCAAAAATAAGTAGCAGTGAGGACAAAAATTATCTTGGCATTGAGAGTTATGGAATCTCAGTCACGACTCTGGAGGAGGTATTTCTGAGAGTTGCAGGATGCGACTATGATGGGACTGATGGTTTCGAGCAGAGGAGTAATATTCTTTCATCTGGTTCTGTGGTTCCTACAGCTTCTCACAATCATGGCTCGAAAAGAGTTTTTGGTTCCAAACTCCTAGGAAATTATAGAAAGTTTATCGGGTTCATTTCTGCATTAGTGGGGAAAGCATGTGGTTTAATGGTTGCGACAGTTTTAAGTTTCATTAATTTTATAGGCATGCAGTGCTGTAGCTGTTGTATCATTTCAAGATCAACATTTTGTCAACATACCAAGGCATTATTTATAAAGAGGGCAATATCTGCTAGGAGAGACCGGAAAACAATTGTTTTCCAGCTTCTAATTCCTGCTGTATTCTTGCTTTTTGGTCTTCTTTTTCTCAAAATTAAGCCACATCCAGATCAGCAATCTGTTAGCTTGACAACTTCACATTTTAATCCTCTCTTAAGTGGAGGTGGTGGTGGTGGTCCCATTCCTTTTGATCTGTCCCAGCCTATTGCAAGAGAG**GTCTGTTAATTATATTTACTTTCTTCTGATTTTGTTGTTCTTTTATGTTGTTCATGCAGCTGTTGAAGCTGTATATTATTCTTGGCAATCATATTCATTTCAAAAACATTCAAATTCTTAAATTTTAAGTCCAGCCTGTAATATAATAATGGAAAATGTAAAATCTTATTGAAGAAGCTTCATGAAAACTTACTTGAAGTAGTGCCCAAGTAAACTACAGTGGATGCATGAATTTGGCTAAGTTTTGACTGATATTCATGGCTATATAAGGAGCTTTGAGCTGGTGTTTTGGCTGGCCACATATTTGAGAGATTGTGAAGTGTGTGCCATGGGTTGAGCAAGCTTCTTGCTTGCATGTGAGAGTATTTATTTTGTATAAGAGTGTATTTTGTGAGGGTTCTAGCAAGAGTACACTTAGGGTCTGTTTAGCACTGTTGACTACTATTGAGAAAAACACCTCTTTAAATGTGCTAGTTAGAGGGTACTAAAAGTTGGTTTAAAGTAAAATTTGACATGGTTTAGTTGTAACAACTCCAAAATAAGTAAACATGTTAAATTTTGGGGAGAGTGCTGGGCATATTACAAATTAATTCATTTTAATACAACTTATATAAGTGATTGAATTACTTTTGCATCAAAATGATTAGCTAATTTAATGTTGTAACCCATTTATTTATATTTTTCCCTTTCATTTCATAGATTTCCCATGTGGGATTTCCAATTCTCAAACACTTCCCCTTCAAGTGCAACTACATGGTTGTTACTTGACAATTAATATTAACCCAATAGGGGCCTCAATGTATAGCCTTATGGAAGCTGAAACCTACGTCCCAATCCGGACAATGATACCATATTAAAATTTGGAGAGTACTAGGTATATTACACATAAATCCATTTTAATATGGGCTTGTAAAAGTGATTAAGTTGCTTTATACATCAAAATGACTAGCCAATTCAGCGTTGCAACTCAATCATTTATATTTTGCTCTTTCCTTCATAGATTTCCCATATGGGATTCCCAATCCCAAACAAAAACTTTTTCAATAACATCTAAATGGTGCTTTTCTGAAAAGAACCTTTTAAACCTCCCAATCTCAACCCTGAATGGAGCCTTAGTAAAGGTAAACATCATTGGGTGTAATTGGGTTAAAGGATTTGCAAGTGTGTGATTAATTAGTGGTTGTATTATTTTCTTTATTAGTGAATTGTTTAGTACAGTAGGCAAATTCCCAAACTGGTTAAACTTTGTGTTCCAATTATTTTGTGATTGCTAGTATATTTTTCACAACAATAAATTTTACTAAACTGCAG**GTTGTAAAATACATTAAAGGTGGTTGGATTCAAAGCTTTGAAAAGAGTGTGTATAAATTCCCTGATTCAGAGGGGGCATTAGCTAATGCCATCAAAGCTGCGGGGCCAACTTTGGGACCTGTTTTACTTTCAATGAGTGAATTCTTAATGTCTAGCTTTAATGAATCTTACCAGTCGAG**GTATGTTAACTGAATGCTGAATCACTTTATCTGTCAAAAGTTGGCATTCTCAAAACCGCATGTAAAAATATTGCATCATGTATTTCATTTTTAAATTTTAGTTCTTCGTGTTAGCTTCATTGTTTTGGTGCTTAGCTGCCATTACATTAGTTATTCCATGTTTTCTCCCTCATCATCAGAAATGACAAATGGTTGGATCCTTCTTTTCTATAATTGTTCTTTCATGAAGATATGCATGGTTTTAAATCATGGAACAGTTATTGTGTCCCTGAAGTAGGTTGTTGTGGACATAATGTGTAGTTGCTTTTTTTTTTTTTGTATGTTATGCATGTGATATGTAATTGTTGGAATTGATGAGATTTTACTTAAATTTATGGGATATGCTTATGAGAAAATCACGTGATTAGTTAACTTAATTAGTACTTATATTAAGGAGTGATTTAAGGAATGCCATATATCTTGTATCTTCTATTATAAATATGGACTCTTATGTATTATGTCAAGTACAAGTAACTGAAAATGTAATCCTTTCAATTCTCTCCCTTTCTCTCCCTTTTCTTCTTTCTTTCTAAAAAATCAAGATGGTATCAGAGCCTACAGAATTCTAGGGTTTCTACTTTCTTATTGTCCTCCTTCTTCACAAAGCAATGGGTTTTAGTCCCAAAAGCTATTTTCTTTCTTCAGGGTTTGAGTCCCAAGCTATTCATCTTCCTCTTCTTCTCTTCATGACTTGAGAGTCTAGGGTTGATGTCCCTACTCTTTTCTTCTCCTTCATCAAAGCCTAGGGTTTGAGTCTTGGCTATTTTTCTCCAATATTCTCATTCTTCTTCATTTTAGCCTAGGGTTTGAGTCCCAAAGCTGTTTTTCTCCCAAATTCTCATTCTTGATGATGCATCCATAACTTCAGCAAAAATCTCATCAAGGGGGAGTGTTGGAATTGATGAGATTTTACTTTAATTTATGGGATATGCTTATGGGAAAATCATGTGATTAGTTTACTTAATTAGTACTTATATTAAGGAGTGATTTAAGGAATGCCATATATCTTGTATCTTCTATTATAAATATGGACTCTTATGTATTATGTCAAGTACAAGTAAATGAAAATGTAATCCTTTCAATTCTCTCCCTTTCTCTCCCTTTTCTTCTTTCTTTCTAAAAAATCAAGAGTAATGACTACTTTCCTTTCTTTCTTTCTTTTTCTTTTTTTTCTTTTTTTTAATGCACTGTACGCTGCAATTGTCAAAAAGTACAATCTCTCTTCTGTTAGGACTTCGGATGCTTTAAAATTATAATTTACTAGATGACAAGGAACTTGAAGATTTGAAAGTATAAGGAGGAATTCTGATGAATTTGGCTCTTTCAACAAATTCAAGAATTTAATTAACTACTTGTGTAGTTCACATGATTGTCACCTTTTCATTGGTGTTATTATGAAAATATGCATTATGCTTACAAGGTGTTGTTGGGGTATTTACCCTAGTGGTTTTCTTTTGTTTTGCTGTTTTTCTCCTTTACATGTAAATGTGTTAGCAATGATGATAGAAAAAAATTGTTATCCTTGTGTCAAATTCTTTGTTTACAAGAAAAAAAAATGGTTAACAAAAATTTAGCTAAGAAAAAGAAAAAATGGAGAAAATGAACAGCAACAGCAGCTAAGCATTATTAACTTTTTTTCATTCCTTGTCATTTTCAAATAAATTTGTACAAAACTCAAGTAAGTGTTAATCCTAAGTCATTAACTAGTTGGAGTTGGTAATATGGTTCATTTCTATCACTCACTTGGTATAGGGATATATCTTCAGAGAATCTAGAGCACTGAATTCTTTTTTCCCCCACATCATTTTGACTAACCTCACTTTCTTATCTCCTATAATTTCACTGAACTCAACCTAATTTTCAGGCGTATTTGCTATTCTATGATACACACCTTCAAACCATGTTAGTCTCCCTTTTCTCAACTTTTTGTCAATCGGCGCTGTGAAATTCTTTAAGCCGGAGAGTGCGGATGATTATTGGCATAGGTTCATGTGTTGAGCTTAAGGTTGCCTTCACCAAAATGGTTAAGCCAATTTGGCTTGGTTCTCCTTGCTTTATTTTATATTCTCTTCTTTTTCCTTCTTTGTTGTCAATGTGGGACGCCAGCACTCCTCCCCTCTAGTGCGGATGATCATTGGCATGGGTTCATGTGTTGAGCTTAAGGTTGCCTTCACCAAAATGGTTAAGCCAATTTGGCTTGGTTCTCCTTGCTTTATTTTTATTTTATATTCTCTTCTTTTTCCTTCTTTGTTGTCAATGTGGGACACCAGCACTCCTCCCCTCTAGTGCAAGTAGACTGACCTTAAGTTTATAGGTTCATTTGTTACTTGCATTGTATGCGGTGGTCCATTCACTAGAGTCTAGCCTCTTTCGTTTTGGCTCAAGCCTATTGGTTTAGTTCTTTTTATTAGCCCATGAATCCACTTGGTTTTGGCTTGATGTGGTATTGCCCATCATGCTTTTCGGATCCCATAATATGACCTGCTTTGATATCATGTGAAATTCTTTGACCTGGAGAGTGCAGGTAGCTATTGGCATAGGTTCATGTGTTGAGTTTAAGGTTGCCTCTATCAAAATGGCTTAAGTTAATTTGGCTTGGCTTGCCCTTGCTATATATTCTATTGTTTTCCCTTGTTTGTTACTGGAAGATGCTACTCTCTCCTTCCCATGAATGCATTAATTTTTTTATCTCGTCCATCGTCATGTTTTGCACATACGTCCTACTTTATCCTATGATTGATGTCCTCATTTCACCCTATGTTGGAGAATAAATATTCAAAGATATAATTAAAACTATATGTGAGTAAAACAAAAAGATCAGGGCTCAAGGCCAATCCTTAATACAATCCAACTGTGATAGGACATTTTTTCGCATTCTCTTACCATCTGGACGCTGGTGACTGCTTCTTTATACATATCCTTGTAATGTCCCCAAAAGCCCACTTAGTTTTGTGCATGGTGATTTAATGTGCCTTGCCTAGTAAATTATACAGCCATGGTGGCTCACTCAACTATTCTGCGTCATCATTGTGTGGGTTGATATTTGATTAAAATAGATATTTTTATGCACTCACATTCACTGCTAAAATCTTCACATTTTGTGTTTGTCTTGATTGTGAGCTACTGTTTTTTTCAATCTGATAGAAGAGTCTTTTGATTTGAGTCCAAGTTGGTATGTCGCCTGCCATAGAATTGGAGGTCACTAGCACTAGACCTCGCCACAGTCTTGTTCAGACCTAGAACTAGACCGGTCTAGCCCTGGACCAGATTGAACTGGACATCTTTCTCTCCTCTTGAACTGGCTTAGAGAAAAAGAAAAAAATTGGAAAAAAAAAATTCAACCAATGGATTAAAATTAGAACTGAGCTGGAACCAAGACTGGGGCGACTGTGGTCCAGTCTGAGGTCCCTGGTTCCAAGAGTCACCTGAACCAGTGGCCGCATCTAACTAACCTTATTTGTCATCATTATTGTCAAACCAATATTACTTAAGCTTTAATTTTTCATATTTACATCAGAATCCTTTGTTGTTACAAAATATATGTAATTATGCTTGTTGAATGTGCTAGTTGAATGGCTTTGTGTTTACGTTACATGCCTTTTTCTTGGATGAGACTGATTTTAATCTGACATTAGGCTGTTAGGTTAGGCTAGAGCCAATTTCTGGATGTTACAATTTTTGCCAAGAGAAGCAGCTGGAACTACATGTTTATCACTTTGAACTTTTTAATAAATGATCTAGGGCTCTGTTCCATTTGATGTTAATCCTGTACAG**GTATGGAGCAATTGTGATGGATGATCAGAATGATGATGGAAGTTTGGGATATACTGTACTGCATAATAGTTCTTGTCAGCATGCTGCTCCAACTTATATCAATGTAATGAATGCAGCAATTCTTAGGCTTGCTACTGGTGACAAAAATATGACAATTAGAACTCGCAATCACCCTTTGCCAATGACAAAGAGCCAGCACTTGCAACGACAT**GTATGTTTCCCCTTTCATGTTATTGTTCTCAAGCATTTTACACAAACTGGTTATGCTAGAAGCCAAGATTGAGCCAGATAGTTACTCTTTTATATACAATTTTAAATGCCTAGTATTGAAAATATCATATATAAGATTGTGCAATTTTAAATACTTAGTATTGAAAGTAACATCCATAATATTGCACAAATTAATAGCCATAAGGAATGGCTGACTAGTTGCTAGTTAGATCCTCAAATAAATGCTTCTCAGTTCTCACTGAAATGGGGATACTTATCTGGCAGAAATTAATCTATTTACTTTAATGCAGGATACATTTTAGGTGCTCCTATTATTTTCCCATTGAGGGCCTAAATCATAGCTTGAGAAATTGTTGTCCGTACAATGATAAAGGATTGAATTGTTGAGAAGAAAAGAGCTGTTATGGCCCCCTTGTACCACCCAGATCCCATGAGTGAATAGAAAGTTGGGTCTACATGGGTCATACTTAAATTATCCTGTCTATTTTCCTAAGAGAAGCTTAATGCTACTTGGATTATCCACATCCCAAATTGGGTGCTGATGAGCTCATGGATGCTGGGGGAGAGCGTGGGTTTAGATTAAGAGGTGGCAGGTTCTAAGGAAGTGGCTTCTTCAATCTGGGAATGTCTTGCATTCATTCATTCTTTATGAATATGGGGCCATTCTGGAGAGTGTTGTGAAGCATGGTGCTCACAAATGGCATACCACATGAAATTAGATATACTTTTGTTAAAACGGTTTCATAGTGCCTATATCTGATGCTTTCATCATTATATGCTTATTATGTGTTGGAGATTCTTATACCTTAACAGGAATGCCTTCTGACATGCATGCTCGTCTGTTTTCCTGCTGGATCAGCATATTAGCAGCTTGTATTATGTTATGCATGGACTCGTAACATTGTATAAAAAGATCTATCTTTCTTGATTTAGCATTGCTTCTGACATACGTATCTGTGAATATTTTGCCAGTTCATTGAGACAATTAATGAATTTACAACTTGAATTATCATGTGAATTATGTAG**GACTTGGATGCCTTCTCTGCTGCTGTTATTATTAATATCGCCTTCTCATTTATTCCTGCTTCATTTGCTGTTGCCATTGTGAAG**GTATTGTTTCTGAATATTACTCTGAATTATTTATTTTAAGTAGTTTTCTTCATGCATTGCTTCTGACATACGTATCTGTGAATATTTTGCCAGTTCATTGAGACAATTAATGAATTTACAACTTGAATTATCATGTGAATTATGTAGGACTTGGATGCCTTCTCTGCTGCTGTTATTATTAATATCGCCTTCTCATTTATTCCTGCTTCATTTGCTGTTGCCATTGTGAAGGTATTGTTTCTGAATATTACTCTGAATTATTTATTTTAAGTAGTTTTCTTCATGCATTAAGCATTATCGCATAACTTATAACTGCTACTCATAAGGCATGTGGTAAATTCAGTAGCAAAACAATTATGAAAGTTTAAGCATTTGCATTGTTTGTAACATTTATCTTTGGCAATAGAGGTAATCTAAATAGGATTTTTTCGACGTAGATTTTTTTAATTTGTTGCCTTTATTCCTATTCCTTTTTCCTCTAAGCATGTTAGCTTTGTTTTCCAGTTTCCATTGTGTACATGTGACTGTCTACTCTTTGTACCTTGTGCATCTCCATGGGTCCTATATGAATTTTCTTTTCAAAGATGTTACCATGTCCCTTCAGTCCTGTTGCAATATTTTTACCTTTTTTTTCTGCTCTTATACATTCTTTTGCCTCATGTTCATTACATGTCAACTGCTCTACCACTCTACCTTTCTGCAGTGGTCCTCATACGTAACTTTGTTCTTGTCAATGTCAACTTGCTATCTGCCATGACAGCAGTAAAAAATTCTCAATTTCTCTTGTGAATTTATTTGCTTGGGCTGATGATATCTAAAACTTCTTCTCATAACCTGTTAGAATTTTATTTTTTCATGGGAGTCATTTTGTTCTTTGCCTGGCTGATGTCCTCATAAGAGACTTGTAACATTAAGTATTTCCATTTTTGTCATGGCATGACATTTCATATTATCTGTTCTATTTGATCCAG**GAACGTGAAGTGAAGGCCAAGCACCAACAGTTGATTAGTGGG**GTATGATTGCTCTGTACTCTTTAAGGCCAAATTAGCTTCTCAGTTTTTATTTTTTCATAACTTTGAAAATCTCCTTTCTCTATCTCTCTCTCTCTCGCTCTCTCTTTCTTTGAAAAGTAATGTTTAACAATGTGGTGTTGCTCTCAG**GTTTCTGTACTTTCATATTGGGCTTCTACATATATTTGGGACTTCATCAGCTTCTTATTTCCTTCATTTCTCGCAATAGTTCTTTTCTACATCTTTG**GTAAGTTTAAAATATAAGCAACATGCAAGCAGTTCAAATTTTTTGAGGGTCTTGATGAATGTAGAAATAGATGATTCTCCTTAATTTCAATTAATCTGTACATGGGTATATATATACAATTGATTCCTATAATTGTGTTCTACTAATTAGGAAGAAATCCTAAATAAGAAATCCTAAATAGGAATACAGAATACAAAATATACAGAGAAATAATATAGTGATTGACTTTCCATAACATAGTGATTGACTTTCCATAACACTCCCCCTCAAGTTGGAGCATAGATGTTAATCATGCCCAACTTGTTACAAATGTAGTCAATCCTAGCTCCATTCAGAGTTTTTNNNNNNNNNNNNNNNNNNNNNNNNNNNNNNNNNNNNNNNNNNNNNNNNNNNNNTTGTACCTGATTTACAGAACATATATATATATATATATATGTCCTTTTGGGTGTTTTAACCTTGTCAGAAGTTTGATTTGGGCCTGCAG**GTTTGGATCAGTTTATTGGAAGGGATTGTTTCTTTCCAACACTTCTGATATTTTTGGAGTATGGACTAGCAATTGCATCTTCAACATACTGCCTTACATTCTTGTTTTCTGATCACACCATGGCTCAG**GTAGCAAAACATTCTTTTCTCTTTATGTTTTTTTCGGACTTCCAAATCATTAAGCCGATACCATTTTCAATGAAACATTTACTATCTGCAG**AATGTGGTTCTTTTGGTCCACTTCTTCACCGGACTGATTCTTATGGTTATCTCATTCATAATGGGGCTTATAGAGACAACAAGAAGTGCAAATAACTTTCTCAAG**GTTTATTTCTTCCTTATGCTGTCATATATATTAATCAAGGAACTAAATGGCTTTATTATCAATGGTCATAATTTGAAACCAAATAAGTGTCAGATGTATAAATAGGCATATATATATAAGCAGTGTTGTGAAAGGTGTGCCTAAGGCGCAAGGTGCTCAGGGAGCTAGGGGCTGCACATCGCCCTAAGAAAGGGGCTTGACCTCGCCTAGGTGTAGTGAGGTGCAAGGCGAGCCCAAGCGCACTTGGGTGAGGTGGGTAAGAAGAAGACCAAAAAGAAAAGAAAAGATAAAAAAAGAAAAGAGGAGGAGGAGGAGAAGCAGCCTACCCATCAAAGACTGCCATTGAAAAGAAGAAGAAGAAGAAGAAGAAGAAGAAGAGGAGAATAAATAAAAGCAGATGTACCCGGCTGGCTCTGGCTACCTTTGCTTTCTATGATAAAACTTGGTTGTGTCTGTGTATTTGCTGTGTATCTCTAATGCGGCTATTGGTGATAAAATGGGCTAATGGTTAATGGGCTTAAAATCTAATTGAGAGTTTTTTAGTGATCTTTTGCATTTGCATGCTTTTTATTTTTTAAAATCCTATTTTACATAAGTTAAAAATAAATGTAACATACTTAATGAGTTTATAAAATTCAATTTAAATTATTTTAAGATATGATTAATAAGGCATTA

>HbABCA2 scaffold0189(1711459-1718552)

**GGGAAATTCGGACCCGATTATTTTCCTATCAGCGTTCCCAATCTATCCCCAGGTGTTAACCATGAACCAGAAACGAACCATGAACCGCGCTTTTTTAAGCAGCTGGCCGTCTCATTAAACCAACAAAGCGTTGAGAGCTATGGAATTACAGAGAGGATTTCCTCTGCTATACCAGCAATCTAAAGCTCTATTGAAGAAGAACCTGTTGCTTTCATGGAGGAACAAGAGCGCCTCATTCCTCCAGTTGTTTTCATCCTTGTTCTTCATTTTCCTCCTCTTCTGCATCGAAAAAGCCATGAACGCTGCCAACTCCGACACCACGGCGTATAAGTCGGTACTCGATCCTCAGCCTCTGGTTTCGCCGCCGATCCCTCCCTGCGAGGACAAGTTCTACATTAAGAAGCCTTGCTTCGACTTTGTATGGAGCGGAAACTATAGCGATAGACTGAATCGTATTGTCAGTTCAATCATGGCAAATAATCCTGGCAGGGTGATTACGTCCAATAAG**GTATGGCACAAGGAAATGAGAACTTGTTTGATAACAAATGAAGAAAAGAATCTTGTTTATAATTAAAGAAATTTCGTTTCATTGTTATTGCTGTCTATTGATGTGTTTTAGGAATGTCTAGTTTTGAATTTAAACAAATTCTAATGAGAAATGAGAAGCTTTACTGTGATGATGAATGATCATGGTGTAAGGTAGTACAATTTGACGGTTATTATTCGGAGTTTTGATTTGGTATGGAGCTTTACCTGTTTAGGTTTGTTAGTTTGCCATATTGATTTCTTTTTTCCTTTTATATTCGTTGGATTTTTTTTTATCAGAATCATCTCTCTCTCTCTCTCTCACACACACACACACACACACGCACACACACAATTAACAGTTAAACAAGCTATGAACAGTATTTATATGGCTGTAGATAGTAGCTTTTCGAATAAGTTGCAACTTTAAATTTTCTATTAATTTTCTGAGAGTGTAAATAAACAAAACAAATTTTGAGGGTTTCAATCTTTTTAAAGAGAAAGAAGTATTCTGCTAGCACCAAATTCATAGTGCTTTTTTTTTTTTTTTGGGTTAGGTTTTCTTTAGTTATTAGGTTAGTTATGATTAATCACGTCTTTGAGCAATATGTAGCTTTCTCTTCTTTTTCAAGTCCAAGTACCGATTTTATTTTCATGCCACTAAATTTCTTATCATTTAATAATTTCGTTTTGGTTGTTCCAAAAACTAGTTGGGAGAAAAAATCTGGCAAACTGAAAAACCAAACTTGATTTCTTAGACATAAACATCATCATGAAAACGCTCCTGTTTGCCTTTCAATGTACAG**GTTAAATCATTCAGAACAACAGACGATGTAGACTTTTGGCTTTTGAATAATCCTATGCATTGCCCTGGAGCTCTGCATTTCAAAGATATAAATGCCACTGTTATCAGCTATGGCGTACAGACTAATTCTACCCGAGTTATGAATCGGGGGTATTCTGAAGATCCTACATTCAAATTTCAAATCCCACTTCAGATTGCAGCAGAGCGTGAAATTGCTCGGTCTATCATTGGAG**GTGTGAAATGACACATCTACTTTTCTATATTTGATTTACTAATTATATATATTGTAACTCCACATATCCAGGCCCAAACTTTAGCTGGGTATTAAGGAATTTATTTGTGCTCTTTTGGTGAAATTTTGACATATATGAAGAGTCTTATTTATTCATTTGAATAAATATGTTTCTTATTTTGTTATTCTGGATAATCAG**ATCCAAACTTTAGCTGGGTAGTTGGACTTAAAGAATTTGCACACCCAGCAAAAAACAATTTCTCTGTGTTGGCTTCAATTGGACCAACTTTCTTTCTTGCGTTTGCCATGTTTGGGTTTGTGATGCAAATTGGATCTTTGGTGGTAGAGAAGGAACTCAAACTTCGCCAG**GTTTGACTCTATCTTTATTTGACTTGAATAGCACTAAAAATTATAGCTACATAGCAAGGGTATCTCCCACATCTTAAAGATCTCCATTCCCCATCTGAGTGTTCCAGACCTGAGACCTCCAAGTTGGAATCTCAAGCTTTAGCTATTAAGTTGTGCCCTCAGGGACCTGAATTTCTAATATTGTTGATGTGTATTATTTATTTATCTTTTTTCTGCTTCCACAG**GCAATGAGTATGACAGGTCTTTATGAATCTGCTTATTGGTTCTCTTGGATCACATGGGAAGGAATACTTTCACTCGTATCATCACTTCTCCTTGTTCTTTTTGGAATGATGTTTCAGTTTGACATGTTCAAGAAAAACAATTTTGCAGTTGTGTTTCTTGTGTTCTATCTTTTTCAGCTCAATATG**GTAAGACTACTCACTGTCTTCTGTGCAGTTTTCACTTATTTAATTTATGCTTATTCATACCAACTCTTTTCTGTTATTTTCTCACATTTGATGCCAG**GTTGGTTTTGCGTTTTTGTTGTCAAACTTCATTAGCAAGTCATCTTCAGCCACAACAGTCGGTTTCTCGGTATTTATTGTTGGCTTTTTTACACAG**GCAAGATTCCTCCTATGTATTATTAACCACTGTTTTAAAAACCAGACTGGACAACCCCATTGTATTGCGTGAGCCTTAAATCTGAGAGTATATTTGTCTGGCTGATATGTATCACCCATTTTAAGGGAAAAAAGGGGAGATACTCGTTTGACATGACAGTCAAACCTTCAACTAACTGATCAAGACTGGGTTTCTGAAAATTCTCCTTTTTTTTTTTTTTTTGGAGCTTGATTTATTATACAAGTCTTCTATTGGGCCCCTGCAGATGCATAGCACGAGTAAATAGAGTCCCCCCTCTACCCCCCTCTTTAAGGCCTAAAATAGAGTCAAAGTCAAATTCTTGATTTCATCTCTTTCCGCAGAATTTCAAATCAATCTCTTTGTCCTCACATGACTTACATTGATAATTAGCATTTAAATTGTTTCAATATTTTATTGGAGGTTGCTGTTTTATTTAAAAGAACAGGTTAAATTAAAGCCAGTTTGTCCTCGCAGTAGAAAATGAAAGGAGATGTCTATTAAATAAAAAATATAGAACAAATATGAGTCATTGTACTTGTTATTATTTTTATTTTCATATTTGTTATTTTATATTTAATATATTTTTAACTTCATAAAGTCATCCGTAATCAGTTCAACTGAAATAATTGTTCCCTTGTTGTTGCAG**ATTGTTACCATATTTGGATTCCCATATGGTATCCATGTCGCTAGTATTCTACGAACTCTCTGGTCATTATTCCCACCCAATCTTCTTGGTATAGCTGTGAATTTGCTTGCTCAGGCAACTGCCACTCCTGAAGATGTTGGGATCAGCTGGAGTAGACGGTCAGAGTGTGTACGAGATGATAGTGCAGCAGACAAGTGTGTAATATCAATT**GTATGTAAAAACTTTTAAGGCTCACTCATAAGAAGACACACACACATGCACAGACACTATCTTATTTTGAGCTCACTATTTTTTTTTTTTAAAAAAAAGGTAGATCAGGCCATTGACAAAATGATTTTCTACTTTTTCCCCTATAGTTTTCTTCTCTTTTCTATTGAAGTTAAGTTGAATTCGTCTCATTCAAGCTGATTGTAAATTGTATGTTGTTGACAG**AATGATGTTTACTTATGGCTTACATCTACGTTCTTTGTCTGGTTTGTTTTGGCAATCTACTTCGACAATATAATTCCAAATGCATATGGTGTCAGAAAACCTATATTTTATTTCTTAAAGCCTGGGTATTGGACTGGAAAAGGTGGAAATAGAGTGGAAG**GTTGGTGAGCTTTGAGTTCGGTCTGTTATCTGCATATCTCCTTAGCTAATATTCTGACTGTGTTTTCCCGTTTTTAATAG**AGGGTGGCATTTGTAGTTGCATGGGTTCAGTCCCACAACAAGAGCATATTACTCCAGAAGATGAGGATGTGCTTGAAGAGGAAAATATTGTTAAACAAGAAGCAAAAGATGGCTTAGTCAATCCAGAGGTTGCAGTTCAGGTACGTGGACTTGCAAAGGTATATACTGGGACTACGAAGATTGGTTGTTGTAAATGCAAGAAAACTTCACCTTACCATGCTCTCAAG**GTAAGCAACATCTTTTCAACTCAATTGTCTAGTTACTTTAAGAATGTACACAAACCTCCTTTTTTCCTTTGGCTCTTTTAATTATGTTGTTTGGACACAGCTCATTAAGAATTACCTGCAG**GGCTTATGGATGAACTTTGCAAAGGATCAGTTATTTTGTCTCCTTGGACCAAATGGCGCTGGAAAAACTACCGCAATCAATTGTTTGACAGGCTTAACACCTGTGACCAGTGGAGATG**GTATTATGCTCATTTGTTTCCTTGTTTATCAATCACAATTAAATTGGAAATGGGGATAAGGTCAACAGAAAAATAATAACTTCATGCACTGACTTGTTGACAG**CTTTGATTTATGGATATTCCATTCGGAGCCCTGTTGGCATGTCCAACATTCGAAGAATCATAGGAGTTTGTCCCCAG**GTGAAATATCTATGAGTATGCATGCACACACATTTGTCTGTGCAGATTTTGTCTTTCTTTATGTTTGTTATTGTTTCTATTTACAGACTAGTTTTTTCATCCTTTTACAG**TTTGACATCCTTTGGGATGCATTATCTGGTGCAGAGCATCTCCATCTACTGTGCAGATTTTGTCTTTCTTTATGTTTGTTATTGTGTCTATTTACAGACTAGTTTTTTCATCCTTTTACAGTTTGACATCCTTTGGGATGCATTATCTGGTGCAGAGCATCTCCATCTCTTTGCTAGCATTAAAGGCCTACCCCCAGATTCAATAAATTTG**GTATGTGCTAATTACTTAGTGAACTTGGAAAGATGTTGTCATAATGCTTTCTTTCAAATGATAGTTTGAAGATTAATTGTGCTTTGACCATATTTTGCTTTTGCGGTTATTTATACTTCTGTTGAGTGTAG**GTTGCTGAGGAATCATTAGCAGAGGTAAGACTCACTGAGGCAGCTAAAGTGAGAACCAGGAGTTACAGTGGAGGAATGAGACGCCGGCTCAGTGTTGCAATAGCACTTATTGGGAACCCAAAGTTGGTCATTCTAGACGAACCG**GTATGAAGAATGGAATATTTTGAATTGTGCTTGCATGTTTGATCTAATTGTAGATGCATGTTTGTTAATTTCAATTAGTAATCTGCAG**ACTACTGGTATGGATCCAATATCGAGAAGACATGTCTGGGATATAATACAGAATGCAAAGAAAGGTCGTTCCATTGTCCTGACAACACATTCAATGGAAGAAGCTGACATTCTAAGTGATCGCATAGGAATTATGGCCAAGGGTAGGCTCCGATGCATCGGAACATCAATCAGGTTGAAGTCGAGATTCGGTACCGGTTTCATCACTAATGTGAACTTTATTGAAAGCAATGCTGAACAATCTCCTCCAAATGCATCCGTGGATCTTTCCAATCAACACGAGGATGTAAAGCAGTTTTTCAAATAT**GTATGTTGATGACTAACATGGTTGAAAAGTCTCTATACTTTCAGAGTGCTCTATTTCTGACTCGGGTAATGTCTTGACAG**CATTTGGATGTAATGCCAGCAGAGGAGACCAAATCCTACCTGACTTTTGTCATTCCCCATGATAGGGAGAAGCTTTTAACG**GTAAGCATTTTTGAAATTCCTATTTTGTTTTCTTATTATTGTAACAGTGTCACTGTCACCTTCACGGCTTATATGTGCTATGTTGGCTTATATATGTCAAGTTGAAAGTAGGTTGATGGAAGAAATTGGTTATTTATATACATAATACGGTGGAAAATGGCAATTGCTGTGAACTAAAGATGCATTAGTGTGTATCATCTCAAAAAAAATGGCATTGGAGAAGAAAGAAGATACACTAGTGTATGGATTTAGCCTGCTAATTTAATAAAGCACACAGTTTATCCTTCCTGCAAATTACTTTGTCTTGCTGAAAAAAGATCACTTTGTTTTGGTTTGTGCAGAGACAAAGTATACACTTTGTTTTGGTTTGTGCAGAGACAAAGTATACTCTTTTTAAGGGAATTGCATTTTCTATAATTTAATCTGCTCGTTTCACTATTTACTTACAACAAAGGAGCAGGCCTGTTGGTCATTTTCTTTAATATACTCGCTCTTCAAATCTTCAATAAAAGCAATAAAATAGATATCCTGAATCTTCAATAATTGAAACAAATGTGGCTCTAAGAGTAACAGAGTCCTAGTAGCTGTAGAACTCTAGTTTGACCTTAAAGCCATTGAAGAGTACAGTCACAAACATTTTCAGCCCCCTTTTTTTTACAAGTTTTATTTGTCATTTAAATCTTAAAACAG**AGATTTTTTTCGGAGCTTCAAGATAGAGAAAGAGAATTTGCTATAGCCGATATCCAAATTGGCCTTGCAACGCTTGAAGAAGTTTTCTTAAATATTGCCAAGCAGGCAGAGCTAGAAAGTGCTGCAGCTGAGGGGAGGTTGGTTACTTTGACCTTAACATCTGGAGCCTCAGTTCAG**GTAGGCACGATTTATTTTGAAGTTATATATATATATATATATATAAAGCTACTTAGTAGGAAAAACAAAATCAAGTCTAAATTTGACTCTTTTATTTGCTGCCTAAAATGAAAATGTAG**ATACCTATAGGAGCTAGATTTGTTGGGATTCCGGGAACACAATCTCCAGAGAATCCTAGAGGCATAATGGTTGAAGTATACTGGGAGCAAGATGATTCCGGTTCTCTCTGCATTTCTGGTCACTCTGCTGAAATGCCTCTGCCTCTTAACGTTCAACAATTTATTTCTGCATCACAGCCACGTTCTAATATAAATTTCCTCCGCCGGGGTAAAGAACCAGTTTATGGAATCATGATCGATCCGAATCAGATTAGTCCTAGTAATTATTGACAATCATAAACTCTTATTTATTCTATTATTCTACACCTCTCTGATTTAGCGGTATTAGTCCATAGCTTTTTCTTTGGCTATTTGTATAGGACTTGATATTTGAATAGTTCAAAATAATATCTCTATTTCCCTTCTGGTATTTCATGGGAGTTGTAGAGAGAATTAAA**

>HbABCA7 scaffold0189(1694588-1703072)

**TTAGCACATCCATGGCGGACACATCACATGGCCCTTCAAGTTTCTGGACTCAAGCTGATGCTTTGCTTAGAAAGAACTTAACTTATCAG**GTTATTTCTCTCTCTATCTCTCTCTCACTCTCTGTCGTGGCATTTTTACGTGTGTGTGTGTGTGTGTGTGTGTGTGTGTGTGATATTTGCGAGTAGATTCTTTCTCTAATTTTGATTTCTGGTGAATTGTGAGCCTAGTGTTGGTTCTTTGATTGAAAAATCGGAGCTGATTTTATGATTGATTTTCTTTTTGGTTGTTTTTATGTTTTGATTCTTGCTTGCTGATTAAGTTTCCTGTATTTCTTTTTCCCTCTTCCTCTTTGTGTTTTGCTTTCTGGGTTCTGTGACGGATTGATTTTGTTGGGTTTACTGCTAGCCCGGTTCGCGTGCCTGCATGCGCTTTTCTTTTTTCATTTTTACCTACTGGCGTGAGTTCTGAGATGTGAGTGTAGTGGGGGTGTTTGGTCTGAGAAATTGAAGGGAGAGAGAGAGAGAGAGAGAGAGAGAGAGAGAGAGAGGGTACAAGGAGGGTTTTGGGTATCTATTCTCGGTTTTCGCTTTGGTATTTTCATACTTGAATTTTGTTGTTGATTGAGAGTTTGCTTTTCATTTTTCCTTTTATTTTTGATACTTTTATCCTTTCCCTCTCTAAAACCTATGTTAAGTTCATTGCTTAGTTGGTCCAATTTTCAAATTCATAGATAAACTTTCGTTTCCAGTTGATGGGCAGATTTTAATTGGAATGTGCTCTCCTCTTAACTCTTGATTTTGGTTGATTCTATTTTCGTTAATTAACAAATAGTAACTCTATTTTGTCTGTGTGCTGCAAACAATTATTTATATAATAAATGCTAATCTTTTCTAGGTAAACTTATTTATGATTTTCTGTCTGTCTCTTAAATTCAGTTACATGATGAAGATACTGAATTTGATTTATCTGCTTCTTTTTTCCCCAG**AAACGAAATGCTAAGACAAATTGCAGGCTCATTTTATTTCCATTTATCCTCTGCATACTACTTGTTATCACTCAAAGTTTGCTTGACCATGAATTGAACAAGGCTAGTAGGAAGTGTGGTTGTAAAGATGTTGATATAAATGGAAATGGACAACTGGAGAAAGTTTGTGGTTTGCAATACTCAGATGCATTTCAAGCTGCCACCTGTTCCATTCCGAGCCCCCCGCAATGGCCTCCTCTCTTACAAATTCCAGCTCCTCAGTATCGTGCAGTCAGATCTGAAGTTATTCCATTTACAGACTTGCCAAATGATTCATGCAGGAGTACAGGGTCCTGTCCTGTAACTATACTCTTCACTGGAAATAATCAATCACTTGGAGAAA**GTATCTCTCTCACTCTCTCCCTCCTTCCTTTTGTGTGGTGCGTGTGTGTACATGTGTCTGATGTCCACCTTCATAATCATTTATATGCATCTTATTATTTATTCTCCTTATCACGTTTGTCTTGCCTATTAGGAAATTGGAAATTAACAGTTGGGTTTTTATCTGAATCTTAATCTGGGATTTTGCTGTTCTACCCAG**ATTTGGCTGGGAATATGTTCCCAAGTTCTTTCACTATCAATTCTTCCAATTATATGGACAGTTTAGCCTACAATGCGCTG**GTAATTTTTTTCCTTCTCTTTCTATGCATTTTCTTTCACTTTTTATTCATTTATTGTAATTTTTTGCTAGTAGGTTTGGCTACTAATGTGAATTCTCAAAATGTTTCAG**GGTTCAGATACAGAGCCAAAAAGAGATAATTTTATTGATCCAGCTTTCATTGAGAATTCTACTCTCTATTATGTTCAGCATCAATGTGCATCAAACTCCACATTATCTATTTCTGTTCAGTCAGTCATTGAATTTCAAAAAG**GTATAGATTTATCACCTTATATACAATGCAAATTGTGCTGTTGGATTCACATCTTACTAGTTTGGCTTGTGCTACTTCACAAGGCACAATGGAAATTATTTGTGCCTTTAAATACCATGCTTACGGGCTTTACTTTTCTGAAGTCCCACAACCATGCCTTATAACTTATTCAGTCACCATGCCACTTGGTTCTCTAATCATGTAAGATCATTTGCTTGTAATATTCTGAATTCTTTTTCCATTTGTGTCTTAAATTCAG**AGGCAGCATGTGTTCAAGATCTAAAGTTGTGGCGCAATAGTTCTTCTGAGATAAATGAACAGCTATTCAAAGGATACCGTAAAGGGAATTCTGATGAGAAGATTAATGAGATACTTGCAG**GTTGCCCTTCCTAATTCTCGTGCAATTGTTTTGTGTGAGTGTAGTATCATGTTTGATGTATCCTTACAATGTCTCCTTCTGCAG**CCTATGATTTCTTAAACTCCAACGGTAATAATTTTAATGTGAGCATATGGTACAATTCAACCTATAAAGAGGGTGACATTCAAGGCCAATTTAACTATCTGCGAGTTCCTCGCTTTGTGAATCTG**GTAAATGTCAGAAACTTGTATGCATCATGAGACGTTGGTTGTATAGGTTTAAATATTTGTTAGGCCTTCTGGTTTGACTCTTGAGAACTATGGTTCTGAGAATTTTAATGGACTATGATTTTGTAAGATTAATGGATATAGAAAATAGATTTAAGATTACTGATTCTCTTGGGTGTCTAATACCTCTGCATTATGGAATATGGTCTTTCATTTGCGCACTCTCTTGAACAGTTGATTGTTCCTCCTCTCCCCCCTCTGTCCTCTTTTGTCTTTAATTTATACTTTGTTATTTCAGTATTTTGTGGAAATTTCTGCAAACTTTTTATTGTTTTATTCATTTTTTCTTAATGGTTTATTTTTCCATTTATTTCATGAAAACTTCCTGTTCTTGTGAATGAGAACTTTGATATATCTCATTTGTCAATTTAAGGGGAGGTATTGTAATATGTCCAATATTGTTGTAGCTGGGTTACATCTTTTTAGTATGGCTGCAGGGGGGGTATTGGGCAACCAGTTATGTGCCTGCAACTTATGTTCTAGGGAAGTCTTTTCTCTGTCAAAAGAACAAGGTTGCAATTTTGATTATAATATGTTAAAAAATTTGCATTCTTGTTTAATTTATTTGTTTGGTTTCTAGGATTTGTATCATTTACAATGACTATTGTAGATTTTCATTGTTTCTATTATGCCTTTCTTTGTTTTACTTGTTTTCTTGCTTTCTCAGCTATTCTTCATTTGAATTGAACTGTATAAACTGCTGAAATTTATTATCCTTGCCTGCAG**GTGTCAAATGCCTACCTACAGTTTTTCCAAGGGCCTGGTACAAAAATGCTATTTGAGTTTGTCAAAGAAATGCCTAAAGCTGCCAGCAAAATCAATGTGGATCTGGCATCTCTTCTTGGCACACTTTTCTTTACATGGGTTATTCTACAGCTATTTCCT**GTAAGCATGTGAACCATAGTTCATTAGGATTAAACTGTTTGCAAATTTTGTTTGAATCATGTACGCCATCACCAGTTTATTATAATTTTTTGCATCGGCTGGAAAGGTCAAACTATGGGTATTGAGGATTATACTATGTGACCACAACAAGTTTTTTTGTGGCAGAATTAGCATCTGTTGTCTTCTTTTATTTGGCAAATGAACTTTCATATCAAGTTTTATCTTGTCCATTGAGCTGAAACATGAAAAGGAATCTTCCATTGTGGGACATAAAGCATATCGAGTTAGAATTCTTCCTCAAGGGGACGTGTTTTTAGTCACTGGGGAAAAGTGTTCCACTGATGCTTAGTTCACATTCCAACAAAGCCTATATAGAAAAGTTCTCTTAATTTGGGGTGGTTTTTGCTAATTTCACTAGGAGTCCAATGGTTTAAATCTCATTTCCCTGATTTCATCCTTAATGCTAATTTCCTCTAATTGGGAGCTGTATGTTTACTCTTGCGATTACTTCCTTGTCTCTAGTTTCATTCAATAAGTTAAAGAATGGTAGCCATGCCACCTAATTTCATATTTTTTCTTTTATGTAG**GTGGTGCTGACATCATTGGTATATGAGAAACAACAAAAACTGAGAATCATGATGAAAATGCATGGACTTGGTGATGGTCCTTACTGGATGATTTCCTATACATATTTTCTTTCCATATCTTTGATGTACATGTTGGTTTTTGTGATATTTGGCTCAGTTATAG**GTAATATTCATGTCATTTGTGCTTTCTTCTTTTGCCTTGGCTTCAAGTTACTAATGCAGTTACATTGCAG**GGTTGAAATTCTTCACATTGAATGACTACGGCATTCAAATTGTGTTTTATTTTATATATATAAACCTGCAAATTTCCGTGGCCTTTCTAGTCGCTGCATTCTTTTCCAATGTTAAGACTGCTACAG**GTTTTCTGTTAGCTATTTGATATATTTTTCAGTCAATATTCATTTTGAAGATATTAGCTGGTTTTCTTATCTTATGTGGTTGAACTTTGATGGCTGCAG**TTGTAGGCTACATAGGCGTCTTTGGAACGGGGCTGTTGGGTGGTTTCCTTTTCGCAAACTTTGTTGAAGACTCATCATTTCCTA**GTAAGTAGGACACTTTGTGAAGCATCTGCAATTTGACTGGTCCTAGGGTACTCTTTAAATTAGGAAACCATTTTCATTTTTTTTCTCTTGTTCTGTTAAGCTAACTGATGGCATGTATGGGAATGGGATCATACATTTGGATGTTGTGCTATTACATCAATTGTTGTAATGGTTGTATAGTTATCCAGTATTCCAGCTTTTTAACAAATATGAGGTGTTAACAATCACTTCCTTGATTTTAAAAACCAGCAGCAAGCCGCTTCAAAGTATGCTATGATTATTACGTCATTGGTTTAAAAGGCACCTAATTTTCCAACAGTTGTTGCCTTAGATTCAGTGCTTAATGTTTTTGTATGCTTCATTTTTTTACCATATTAACTCATTATGGGACTTTACTGTTTGCTGCAG**GAGGCTGGATCATTGTGTTGGAGTTATATCCTGGTTTCTCTCTATATCGTGGTTTATACGAGTTTTCACAATACACTTTTACAGGGAATGCAATGGGGACACATGGGATGCGTTGGGGAAATTTGAGTGATAGCAAAAATGGAATGAGACAGGTCTTGATTATCATGTTTGTTGAGTGGTTAGTGCTACTTTTTGTTGCATATTATGTGGATCAAGTTTTATCCTCTGGAAGTGGAAAAAGTCCTCTATTTTTCTTGCAAAATTTTGGGAAGAAGCGTCCATCATCTTTTCGGAAGCCTAGTTTGCAAAGGCAGGGATCTAAAGTTTTTGTTGATATGGATAAACCTGATGTCATTCAGGAG**GTAATAATTTCCTTACATCTAGTGTTGTTACGTAGTATGCAGTTTACTGGTATAGAGCTGTTGTATCTGGGGCACCTCTAAGGGTAGTTCTTGCAATTCTAATAATTGTTTATCCAACAATAGATTTTCAAGTGCTGATGGTCTTGAGTTGTATTCTACCTCTGCCTTCAATAATAGCAGAGCTACATAATTAAAAGCCTTTGAAGGATGTTTTTGCTTCATGTCTTTTTCAGAATTTGCCATTTTATCTTTTTTCAAAAATTTTAGCATCAATTGAGCAACGTCATCATGTATAAATGTAGATCTCACAATATATTTTTATTCAATAATACAG**AGAGAGAAGGTTGAGCAGTTACTGCTGGAGCCAAGCACAACTCATGCAATTATCTGTGACAACCTACAAAAGGTGTATCCAGGAAGGGATGGAAACCCTGAAAAACTGGCAGTACGAGGGATCTCTCTTGCTTTGCCTCCAGGAGAGTGCTTTGGTATGCTCGGTCCCAATGGTGCTGGGAAGACCTCTTTTATTAGTATG**GTTAGTACAAAGTACTCTTTGTTTTCTAATCTGCATGAATATAGAGACTCCATAGCACTAAAAATGCCTTAATAGTCTTTTACAAGCTGGATTACTTTGGAAAGATGATGACATGATTATAATTACTTTTTTCAAGCTTTTATATTTTTCTTTCTCTAATGGAACTAATAAATGCTCAGGAGGGATGTTAGCTATATAAGCCTCATTCTGAATTGTTGATGCCAAAGAAAGTTCTTTGGAGGAGAGATTTGGATAATGGTTATTCCTTTCAGCATTTTCACATAAACTCTCCACTCCAAGTCACACACTTATATTTGGTCTCTACTTTGTCCTTTATAGTTGCTTCTCCATGACATTCTGAGCTTACTGTGAAAAGCTCTGCCTGATGTGGATGCAATTTTTAGGCTTCAATTTTCTGTCAAAATCAGATTCATTTGTGTTTCTGAAACTTTTCTTTTTTTTTATTTTTTTTTATTTTTTTTTATTCTACTGATTAG**ATGATTGGGCTCACAAAGCCAACATCGGGGACGGCTTATGTTCAGGGTTTGGACATACGGACTCATATGGATTGGATTTATACCAGTATGGGCGTGTGCCCTCAGCATGA**GTAAGCTTAAGTCTTCGTTATTTTCTTCAATGAACGCATTTTGGCCCTCCACTTCAATGCCAGTCCATTTGTAACATATGCTTTGGTTTTTCTGGACAAATTTTAG**CCTGCTTTGGGAAACTTTGACAGGAAGGGAACACTTGCTATTTTATGGCAGACTTAAAAACCTAAAAGGTTCTGCCTTGATCCAA**GTAAGCTATTATCTTCTTTACTTTTTTATTGTCATTTGAAAGGTGGTTTGTTTTCTTTAAACAATTGGTGGCTGTAGGAGCCTAGCAGGATTTTCGGATGGAAAATTGGCATGCATAGTTTCTATAAACAAAGAAGCTATAATAAACACACACACACACACACACATACACAAGGCCATAGCAATTAGATTTAGGTGACACATATGAGCAAGTAATTAGAAGTAAACCTGTGGAATGCATGGATGTACTACACAGTGATTGCTCAAATTTTTGGAGTGGAAAACAAACAAAGGATTTTCGGATGGAAAATTGGCATGCATAGTTTCTATAAACAAAGAAGCTATAATAAACACACACACACACACACACACATACACAAGGCCATAGCAATTAGATTTAGGTGACACATATGAGCACGTAATTAGAAGTAAACCTGTGGAATGCATGGATGTACTACACAGTGATTGCTCAAATTTTTGGAGTGGAAAACAAACAATGTGGTAAAGCAGCGGTGAGATGGATTATATTTGTGGTTTTCTAGCCCTGCTTAGTGGGATCAGACATTTGAAATTTACTTTTCTTTTACCTTCTTGTTACCATAG**GCAGTGGAAGAATCTCTCAGGAGTGTCAACTTGTTTAATGGTGGGGTTGCTGATAAACAAGCTGGGAAATATAGTGGAGGTATGAAGAGGAGGCTTAGTGTTGCAATATCACTGATTGGGGATCCCAAA**GTATGTTGTAGTCTCTCCTTAAAGTACTTTGCTTTATGAAACCTTTGGATATAACATCTTCAGAGCCACCTCACTCATAATGGTTGAAAATTTGCAAG**GTTGTCTACATGGATGAACCCAGTACTGGATTGGATCCAGCTTCGAGAAGCAATCTGTGGAATGTAGTGAAGCGTGCAAAGCAAGACCGGGCAATTATTCTCACCA**GTAAGCTGTCCATCTCTTTCTCTATCCCTCTCAATTCTTTCCTGGCATGCAACAGCATTTTAGGAACTTTGGCCCCTTGTCATTCACTATGCTTGTTTTCATGTTTTCAG**CACATTCAATGGAAGAGGCAGAAGCCTTATGTGATCGATTAGGAGTTTTTGTTGATGGCAGCTTGCAGTGCATAGGAAACCCAAAAGAG**GTAACTTCATATCAAAATGATTTTTTTGAGAAAAAAAAATAATATCCAAATGATTTCTTTTCTTTGAAATTTCGTTTGCTATATCTGCATTGAATGTCATACTGCAGAGCAGAGAACATGGTGCTTAACATTTCTTTTCTTCAAATCCTCTTGATTATTGTAGTCTGCCCGTCGATTTGTTACACAAATCTTAGTCTTTCCCCTTTTGAATGTGCTAAACCCACTACTGTATAATAAGGAATGGAAAAGGACATGTTTTAATTACAATTTTCATGACAG**CTGAAGGGTAGATATGGTGGTTCTTATGTGTTTACAATGACAACATCTTTGGATCATGAGCAAGAGGTGGTGATGATGGTGCAACAACTCTCCCCCAATGCTGAACGGACTTACCATACATCTGGAACACAGAAGTTTGAGATGCCAAAAAATGAGGTCAGAATTGCAGATGTATTTCATGCAGTTGAGATTGCTAAAAGCAGATTCCCAGTTTTTGCTTGGGGTTTGTCTGATACCACCTTAGAGGATGTCTTCATCAAGGTGGCTAATGGGGCCTAGCTATAGCCATTCAACATCTCTTTACTTTGTTGGAGATCATTTTCTTTTGCAAATAATCAGCTTTAGTTTTAGAAAATGTAATATGCATAGTAGACGCATACCTCTGCATGTAACTCAATATCATTTTCCAATCA**

>HbABCB1 scaffold0110(968429-975297)

**TGGGTGGTGGGTTTGTTGTTTCAATAAAGCTAAAATAAGCTAAGCGAAAGAGTAAAGAGAGAGAATTTTTGCAAATGTCACAAGAGTCTCAGGAGATAAAGACGATTGAGCAGTGGAAATGGTCCGAAATGCAAGGCCTCGAACTCGTGCCTCCTGCTCATGACCCCTTTATAAACAACACTGCTTCCGCTCCTCCAACTCCAACTCTAACAATCAACTCCAAAGAACACCAACAACAAGAGGAAAACCATCAAGAGACTGTACTAGAGAGAAGAGAAATGGATAACACTACCCCAAAGAAAGATGGTAGTGGTGCTGGTAGTAGTAGTAGTCCAAGTGGTAATGGAGAGAAGTCAGGGGATGTTGCTACTGTTGGGTTTGGAGAGCTGTTCAGATTTGCTGATGGGTTGGATTATGTGTTGATGGCTATTGGGTCTATTGGTGCACTTGTACATGGCTCTTCTTTGCCTCTCTTTCTCCGCTTCTTCGCCGATCTTGTTAACTCTTTCGGGTCTAACGCCAATGACATGGATAAGATGATGCAAGAAGTTTTGAAG**GTAAAGAAAAGAAAAGAAAAACAAATTCACAAGAATCCAATTTTGAGTTACCATTCTGATCCAATGAAATTAACTAGTTCTGGATTTTTGAAAATCGCAG**TACGCATTTTACTTTCTTATTGTTGGTGCTGCAATCTGGGCGTCTTCCTGGGCAG**GTTGTTCTTGACTCTGTTTCGATTTATTGTGTTCTGTTTCTGGTTTTCTTTATTTGCTTATATGTTAATGTGAAGTGAAACAGAGCTTTTGAATTGGGTACAG**AGATATCATGTTGGATGTGGACTGGAGAGAGGCAAACAACTAGGATGAGGATCAAGTACTTAGAAGCAGCTCTAAACCAGGACATTCAGTACTTTGACACCGAGGTTCGCACCTCGGACGTCGTTTTTGCCATTAACACCGATGCGGTGATGGTCCAAGATGCCATTAGTGAGAAG**GTAAAACAGAACAGAACGCAACTCCCCTGTTCCAGCTTTTGATGAGGTCTTGGACAAAAATGTGATCTTCTTGGTTTTGTTTTGTTTATATTTTTGAACAG**CTGGGCAATTTCCTTCACTACATGGCAACATTTGTGTCTGGATTTGTGGTGGGTTTCACAGCTGTGTGGCAATTAGCACTGGTCACTCTAGCAGTGGTTCCTCTTATTGCTGTAATTGCAGCAATCCACACCAACACGTTAGCTAAGTTATCTGGGAAGAGCCAAGAAGCTCTATCACAGGCAGGAAACATTGTAGAACAG**GTAAGAAAGGAATGCAAGGTTCAAATTGGTAATCTCTATTTTCTTAATTTCGGGTTGTGCAGGATAGGATTTAGAGTTACTTTTACGGCAAAAATTTTCTGCATTTTAGTATCTGGGTATGTGCTAAATTGAAGCCAAATTGCAG**ACGATAGTCCAAATTCGGGTTGTTTTGGCATTTGTTGGAGAATCAAGAGCATTGCAAGGCTATTCGTCAGCATTGAAAGTGGCTCAAAGAGTCGGTTACAAGAGTGGATTTGCTAAGGGAATGGGATTAGGTGCAACCTACTTTGTTGTTTTCTGCTGCTATGCTCTGCTTCTATGGTATGGAGGTTATCTTGTTAGGCATCACTACACCAATGGAGGACTAGCAATAGCCACCATGTTCGCTGTCATGATTGGAGGATT**GTAAGTGCTCTGTTTCCGTTACAAAATTTGACCGCTTTCTCTAATTCAATCCTTTTCCCTTTCATTTTTGGGTTTCTTAACTTTTGTTTTGCTTGTTTGGCAG**GGCTTTAGGCCAATCTGCCCCTAGTATGGGTGCATTTGCGAAAGCAAAAGTTGCTGCTACAAAAATTTTTCGTATAATTGATCATAAGCCTGCTGTTGATCGAAACAGTGAATCTGGGTTAGAGTTAGACTCGGTTACAGGGCTAGTAGAGCTCAAAAATGTAGATTTCTCCTACCCATCTAGGCCAGACGTGCGGATTCTAAACAATTTTACCCTCAATGTTTCTGCCGGCAAGACCATAGCTCTGGTTGGCAGCAGCGGCTCTGGCAAAAGCACTGTGGTCTCCCTCATTGAGAGATTCTATGATCCCAACTCAG**GTAAATTCTGCTATAGTTTTATTCTATATTCATAAAGGTGATATCTTTACTTGTACATCTGCTGATCTTTTGCTCCACAAAATTAATGGGTGTATGCTTACCAATTTGTAAACTTTGGTTCTTGAGTTCATGTACTCCAAGTGAGTATTGGTGTTTTAAAGAAATTCTATTAATAAAATCGATATTTTTTCTTCAG**GGCAAGTTCTGCTAGATGGGCATGACATCAAGACATTAAAATTGAGATGGTTGAGACAACAAATAGGACTTGTTAGCCAAGAGCCTGCCCTGTTTGCCACCACCATTAAAGAAAATATACTGTTGGGTAGACCTGATGCAGATCAAATCGAAATCGAAGAAGCTGCCAGAGTTGCCAATGCACATTCATTTATTGTCAAGCTACCTGAAGGCTTTGATACTCAG**GTGTGTTAGTTTTTTGACATTAAACTGTGAATACGACCTAAGAAATCCAATAGGGCAATGAAAGTTTTGTCTCTTGTGGTAATTTTCATCACAGTTGATCATGAACTTGTTCTGATAACCATCTATTTCACTCAAAAACTGGGAATCTTCTGAAATTGAAATTGAAATCTTCCAAGTAAATTGATTAAATGGCTAACTACTCTCCTTTGACATCCAACCATAATAATTGCCAAACTAAGTGCTATCCTATACAAGTTTGACATGTTTTCTTTATGTCAGATGAGGGAGACGGGGAGTGGGGTAAAGTGGGTTGTTCTAGATAGTGAAAGTGAAATGATGGATCTTGACCAGTAGGGATCATGGTTGGTTAGATTTTTTTTTTATGGCAAATAATTGCTACTTGCTACTTCAATATTTTGGGGGTATTAAATATTGAAATTACTTATTTGGTCAACAAACTTGGCCTAACTTTATTTAATTTGTTTTTTAGAAGCCAAATGTGAGGATGTGTTTTGGTCATTCATGCATTTGAAGGAAGGTAACATGCTTCAGTCTACAATCCATGTGCATAAATAAGGGGAAAATATAATTAGAAAGAGGATATAACTTATGAGACATGAAGAAGACAATATTATAAGAAAATGAAGAGCATCAAGTCTTCATTATATGTGTTAGTCTAACCCTTGGGAAAAATAAACAATTCAACATTAGAAGGAGAATAAGAATCTTTATGATGTTGACCAAGGAGGTGGATGATGACTTGAAGAGAACAGATGGGAAGAAATACCTGAAAGAATGATAATGAATGGACTATTCATTTAGGCACTAGTTAATCAGCAAGGATTGGAAGTTAAATCAACATCTTCCAATTCATGTATAGAAAACAAATAATAAAATCAGTAAGCAAGCCTTTAATTAAGATTCATTACTTGCATATTTAAATTTACAACTGACTGGCAGGAAAATCTGAAAAGGTGACTAAAATTAAAGACAGTTCAATGATAACAAAATTATACCAGGAAAAATTTTAATTAATTGTTTGAGTGGTTTGAAGAGGAAATAAACCAAATATCTTACCTCAACCAAAGCTGTTAACGTAATTCCATGTTTTTGTTTCTTTTTTTGTGTTGTACTGGAATCAACTATCATAAAATTTATGCGGAATTACCAGTGAGCATCAGTAAGAAATAATGTTGAAAATGACAG**GTAGGTGAGAGAGGACTGCAGTTGTCTGGAGGACAGAAGCAGAGAATAGCTATAGCAAGGGCAATGCTGAAAAACCCTGCTATTCTGCTTTTAGATGAGGCAACTAGTGCTTTGGACTCTGAATCAGAAAAACTGGTGCAGGAGGCCCTTGACAGATTCATGATTGGGAGGACAACTCTTGTCATTGCTCACAGGCTCTCCACCATTCGCAAGGCTGATCTTGTAGCTGTTCTCCAGCAGGGTAGTGTCACTGAAATCGGAACCCATGATGAGCTTATTGCCAAAGGAGAGAATGGTGTTTATGCCAAGCTCATCAGAATGCAGGAAATGGCTCATGAAACAGCTATGAACAATGCCAGAAAGAGTAGCGCAAG**GTGTTTCAACTTTCTAATTTCAATTTCACTTTAAAAGATCTGTTCCATAAATTTTCTGGTTTTCCAAAATGGCGCATATATTATAATAAAAAATGTCATTAGTTTGTTCACTAATGGGTTCTTCATCTGCAG**GCCCTCAAGTGCCAGGAACTCTGTGAGCTCACCAATAATTGCACGTAATTCTTCTTATGGTCGATCACCATACTCACGCAGGCTATCTGACTTCTCTACCTCAGATTTTAGTCTCTCCCTTGATGCCACACATTCCAATTACCGACTCGAAAAGCTTGCTTTCAAAGAGCAAGCGAGTTCATTCTGGCGCCTTGCAAAGATGAATTCTCCTGAATGGGTTTACGCACTAGTTGGTTCTGTAGGCTCAGTTATTTGTGGCTCCCTGAGTGCCTTCTTTGCCTATGTCCTCAGTGCTGTCCTCAGTGTCTACTATAATCAAAACCATGCTTATATGAGCCGAGAAATTGGAAAGTACTGCTATTTGTTGATTGGGCTCTCATCAGCTGCACTAATCTTCAACACATTGCAGCATTTCTTCTGGGACATTGTAGGAGAGAATCTTACAAAACGAGTGAGGGAGAAAATGCTGTCAGCAGTACTAAAAAATGAAATGGCATGGTTTGATCAGGAGGAAAATGAGAGTGCTAGGATTGCAGCAAGATTAGCTCTCGATGCAAACAATGTCAGATCAGCCATTGGAGACAGGATTTCAGTGATTGTACAGAATACAGCCTTGATGCTAGTTGCTTGCACCGCAGGGTTTGTTTTGCAGTGGCGCCTAGCCCTTGTCCTTATCTCTGTCTTCCCCTTGGTTGTTGCAGCCACTGTTCTACAG**GTTTGTTTCTCTAATTTGTTCATCAACATCAATTTTACCATGTTTGGAAACAAATGGTCAAATTATTAGAAGCTAGTGGTGAACCGTTAATTAAAATGTGCATTGAATTCAATAGATTATTTGAGTGGGACAAATGGAATATATTTTAAGAGGGAAATTTTGAGAAAATTGAATGGAGTCAATAATTATGAGCAACAGTTCTTATTTTCCTTGCCATTTGAAACTTTTGTCCTAATATCACCAAAAATGAATCATTTTTTTGTCACCATTTTTGTTGGGTTTCAACAG**AAAATGTTCATGACTGGTTTCTCCGGGGACTTAGAAGCTGCACATGCCAAAGCCACACAACTGGCAGGTGAGGCCATAGCCAATGTAAGGACTGTTGCTGCCTTCAATTCAGAGTCACAAATAGTTGGTCTTTTCGACACCAACCTTCAAATTCCCCTCCGACGCTGCTTTTGGAAGGGGCAGATAGCTGGAAGTGGATTTGGAATAGCCCAGTTTTCACTCTATGCTTCCTATGCTCTTGGTCTTTGGTATGCATCTTGGCTGGTGAAGCATGGGATTTCAGATTTCTCAAAGACAATTCGAGTTTTCATGGTCCTTATGGTTTCTGCCAATGGCGCAGCTGAAACATTAACCTTAGCTCCTGACTTTATCAAGGGCGGTCGAGCCATGCGCTCAGTCTTCGACCTGCTTGACCGCAAAACAGAAATTGAACCTGATGATCCAGATGCCACCGAA**GTTCCTGACCGTCTTCGTGGAGAAGTTGAACTAAAGCACGTAGACTTCTCTTATCCAACTCGCCCTGATGTCCATAAGGGCGGTCGAGCCATGCGCTCAGTCTTCGACCTGCTTGACCGCAAAACAGAAATTGAACCTGATGATCCAGATGCCACCGCAG**TTCCTGACCGTCTTCGTGGAGAAGTTGAACTAAAGCACGTAGACTTCTCTTATCCAACTCGCCCTGATGTCCCAATTTTCCGTGATCTCAATCTTCGTGCAAGAGCTGGAAAAACTCTAGCCCTTGTAGGTCCAAGTGGATGTGGTAAGAGCTCAGTCATTGCACTTATACAGCGTTTCTATGAGCCATCATCAGGAAGGGTTATGATTGATGGGAAGGACATTCGAAAATACAACCTCAAGTCCCTGAGAAAGCACATTGCCATGGTCCCTCAGGAGCCATGTCTCTTTGCAGCTACCATTTACGAAAACATTGCCTACGGTCATGAATCAGCAACAGAGGCAGAAATCATTGAAGCTGCTACTTTAGCCAATGCTCATAAATTCATATCAGGATTGCCAGATGGGTACAAAACATTTGTAGGGGAGAGGGGAGTGCAGTTATCCGGTGGACAGAAGCAGAGAATTGCCATAGCTCGGGCTCTTGTAAGAAGGGCAGAACTTATGCTACTTGATGAGGCAACAAGTGCGCTAGATGCTGAGTCTGAGAGGTCTGTCCAGGAGGCTCTAGACCGTGCTTGTTCGGGAAAGACCACGATTGTGGTTGCACACAGACTATCAACTATTAGAAATGCCCACGTCATTGCTGTGATCGACGATGGGAAAGTAGCAGAGCAAGGATCCCACTCTCATCTATTGAAAAATTATCCTGATGGATGTTATGCTCGTATGATACAGTTACAAAGGTTTACACATAGCCAAGTCATTGGAATGACTTCAGGATCAAGTTCCTCTGCTAGACAGAAGGACGACGAAGAGAGGGAAGGCTAATGAGATCAAGAGAAAGCAGCAGCAGTAAGAGGAGTATATTCCCACATACCCCTTGTGATTTTTGAGTATTTTTTTTTTTGTTTTAATGCCATGGTATAGTATAGTATTGTGTATTATCTAATTATATTTTTTTTTACCATATATTCTGATCCTATGAATCTTTCCAAGG**

>HbABCB11 scaffold0214(914859-921970)

**GTAATTAAGACTCGAAGCTTGAAGAGGCGGGAGCTAGCTTATGTACGGCAGGCGCCAACTGTATCAAGTTCACAAGCCAAACATATCAGAAACTGCTCGACATCATTATCCTTCAATTCTTCTG**GTAAGTGAAAAATTCTTTCTCAAGAGACCTTTTTTTTTTTCCCTGTAGTTCTATCTTGATCTTCGTTCTTTAACTGCTTGTTTGTTTGGAGGAAGTTTTTGGAGGTAAAAGAATGAGTTTTTAAAGTAGCACGCTTGATTGGAAGGAAGATTTTTAACAGAAGGAAAGGTTTTTTTAAGGTTTAAAAACCTCCAAAACTTTCCTTTTTTCAATCCACTAAATTATTGGGTTGAGAAAATTTTACTATTTACTTACTATTATTTTCCCTTACCTTACTTTATATAAACATTCTCTCGTTCCATCTAAACACACACTGTAAGATGTGCGTGAAAAAATGAGGAGAAACTTCTTTCGGTTTCAACTTTCAAGAAACTTGCTGTATGTTCTCACAGAATTCATTTGATATCCATATGAGCTAGAGCAGTTAAGGCTGCCAAAGGAGGAAGATTGAAGAGATTAGTGGCGTGCTCAAAATGTCAACCATTTTAATTAATGCTGTGCTTTGATTTCTTTTTTTATTTTTATTGAATTTAACGTTTATTAATTACTATTTTACTTTAATGGGTAATAGGAATCGTAACTTGTGAGATTCAGATCCTTGTTTTCATGCATCTAGAAGAAAGTTATAACTAAAAATCCTAAATTTTAGGGATAATACTAAGATAAAGATGGATCTTTTTTTATACTACATCCCTATATTTTAAAAACCCTAGTCCCTATAAGTTGATGTTGATTTGAGGCGGAGGATAACTTCGTCTGCTCTATCAATTACTCACCCACTTACCTATTAGTCAGTGAATTGCAGAAGGGGCATTTGTGTCCTTTTGATAGTAGTTTGTGCCCGTGAAGTGTTTGACATTAGATGAACTCCTTATGGATTTGGCTTTTCCCATTAAACAAAAATCATCACCAATTTTTCTTCTTTTTGTTTCCCAATTTCGTGTCTGCAGCGTAGTTTTCAAAAGTAATGAAGAAAAAAATAATTCAGATATCTCCGCAATTGCAGTGAAAGGTATTGATCCAACTACTGAATATCTATAGATATGAATGCATGTGCATAACAATGAATCATGGGTTAGTTAGGCATGGTTACTGTTTCTTTTTTTTTTTTTTTTTTTCTTAGTCATTCTTGCTTTAATTAAAGACAAAGAGGCAGGTCTCTTTTTATTTTTCTTGAACTGGTCATTGGTAATGGTTTGGAGTTTGTTGAAACGTTAAACCATCACTCTCATATACTATTTGTTTGTTTGTTTTCTATTGCCGGCCATGACCGGTTCTATTTTTAATGTAGTATTTTGTGCTGTTTTCTGTTACTTCCACTGCAAGTTAGTTTAATAAGTTTATTTGGATGTGTTCTGCAG**CCTCAGGCTAAATTCTCTGCAAAGTTCTGCGCCTTCTGATACTAATTCAATTTCTGAGTTTCATATCTTGGGATTAGAATTATGGCTGAGGAGAACGGGGATCCCAGGATGCATGAGGCCAACACATCAAATTCTCAGGAGCAGGAGAAACACTCCAGCTCCAACGGGAGCAAAGAGAATGACAAAGAGAAAGCTAAGACAGTTCCTTTTCTTAAGCTCTTCTCCTTTGCAGATTCCACTGATGTCTTGTTGATGATTACTGGCACGGTTGGTGCCATTGGGAATGGAGTGTCTATGCCTCTAATGTCGTTGCTATTGGGACAAATGATTGATTCCTTTGGAGGAAACCAGAGTGATAAAGATATAGTTAATATAGTTTCCAAG**GTAGAATGAAATTAATAGTCGGCGGCTGTAACATTTTCCATGGAATTCAGATTCTAAATTATGTTTTTTATCTTGGGAATTTTTGGAATTTTTCATGTGAAAGTGTTAATGATTTGATACTTGCAAGTTTTTCAG**GTCTCTCTAAAATATGTCTACTTGGCAGTAGGAGCTGGAGCAGCAGCCTTTCTTC**GTAAGTTCATCCCATACCTGATATGCCGACAAAATTTTCTAGAAGTACTGTGAATTAAAAGAAAATCCTGTGTTTGTTTGCCAG**AGGTGACCTGCTGGATGGTCACAGGGGAAAGACAGGCAGCACGAATCAGGAGTTACTATTTGAAAACTATATTGAGACAAGATATTGCTTTCTTTGATAAAGAAACAAATACGGGAGAGGTTGTTGGTAGAATGTCTGGTGATACTGTTCTTATACAAGATGCAATGGGTGAAAAG**GTACTATACTGCTGCAGTTTTTAAAATCCTCCAGCAGGGGAAGTTGCAGTGAACTTCATTAATCTTTTCCATCTCATCCTGGGACTAATTCAAGTCTGATTGTTTTGTGCAG**GTTGGAAAATTTCTGCAGCTGATGGCAACATTCATTGGGGGTTTTGTAATAGCATTTGTCAAAGGGTGGATGCTTGCCCTTGTCATGTTATCTGCTATTCCCCTGCTAGTTCTAGCTGGTGCAACTGTGTCCATTTTGATATCCAGGATGGCAACTCGTGGACAAAATGCTTATGCAGAAGCAGCTACTGTAGTAGAACAGACAATTGGCTCAATTAGAACT**GTATGCTATGTAACCAGAGCAAAAAGATGTTCGAGAAATATTAGAATCTTTCTTTTGATGAACTGAACCTATCGTGCAATGAACTATGTTTAATTTTGCTACTTATTTTTTGGTAAAGGTATGACATCTATTTGGCATTCCAG**GTTGCATCTTTTACTGGAGAGAAGCGGGCCATTAGTGTTTACAACAAGTATCTCCAAATTGCTTACAAATCAGGTGCACACGAAGGCTTTGCTTCTGGAGTAGGTATTGGCATAGTCATGTTAGTTGTATTCAGTAGCTATGCCATGGCTGTGTGGTTTGGCGCAAAGATGATATTAGAAAAAGGATATAGTGGAGGACAAGTTATTAATGTGATTGTTGCCGTGTTGACTGGTTCGAT**GTGAGTTCCTTTTACGAGTTCTGATGTGAATGCATACATATGCTAATTGATTTTATACAAATCTCCTTTCATGAATCTTTTTTGCTTGTTTCTTGGAATTATGCAG**GTCCCTGGGGCAGACATCCCCTTGTATGAGCGCATTTGCTTCTGGTCAAGCCGCAGCGTATAAAATGTTTGAGACTATTGATAGGAAGCCAGAAATAGATGCTTATGATACAAGTGGAAGAGTATTGGATGACATTCATGGAGATATAGAATTGAAAGATGTATATTTCAGTTATCCAGCTAGACCGGATGAGGAGATATTCAGTGGATTCTCTCTTTCCATCCCAAGTGGCACGACTGCAGCATTGGTTGGGCACAGTGGAAGTGGGAAATCAACAGTAATCAGCCTGATAGAGAGATTTTATGATCCAAAATCTGGTGAAATTCTTATAGATGGCATTAACCTCAAAGAATTTCAACTTAAATGGATTAGAGGAAAAATTGGTCTTGTCAGCCAGGAACCGGTGTTGTTTTCTTCTAGCATCAAGGACAATATTGCATATGGGAAGGATGGTGCTACTATTGAAGAGATAAGAGCTGCAGCTGAACTTGCCAATGCTGCAAAATTCATTGATAAACTACCTCAG**GTTCTAAGTTACTGCACATTTGTCAATCAAAATTTTGTTTTAGGATCATATAAATTGCTGATTTTGAATTTTGCATCCAG**GGACTTGATACCATGGTTGGTGAGCATGGAACTCAGCTATCTGGTGGTCAGAAGCAGAGAATTGCAATTGCAAGAGCAATCCTGAAGGACCCTCGAATTTTACTTTTAGATGAAGCTACCAGTGCACTTGATGCAGAATCTGAAAGGGTAGTTCAAGAGGCACTAGACAGGATTATGGTCAACCGAACTACTGTTATTGTTGCCCATCGTTTGACCACTGTGAGAAATGCTGATATGATTGCTGTCATTCATAGAGGAAAGATGGTCGAAAAAG**GTATCTCTAAAAATTATAGCCATAAAATAAACATTCTCAAGTTGAAATGTTGTGCATTATCCAGTATGCAAAGTCAACTTTTTTTTTCTTCATGGCATGTTTCCAGATATTTGCAAAGTTCTCAAATTATGCCATGTAGCTTTTCAGTAGTTTTCCAGAGTTGAATTCCTGAAGTTTCCAGTGCTAATTATAACATGGTCTAGGAATCCATAAATGATTTCTGACCATAAGAAAATAAATGTCTTCATACTTGAGCCTTCTAGAATTTTGAACTTTGTGATGTGATCGAGAGAGAGGAAGAAACATGTTGACACGCCTGTCAATTTTCAG**GCACGCACTCAGAACTGCTTGAGGATCCTGATGGAGCATACACTCAGCTTATACGCTTACAGGAAGTAAATAAAGAGACAGAACAAGCACCACAGGATTACAGCAGGTCAGAAATTTCTATGGAATCATTTAGACAGTCAAGTCAAAGAAGGTCATTGCGCCGTTCCATAAGTAGGGGATCATCAAGAAATAGCAGCCACCACTCGCTTTCACTCTCATTTGGTTTACCCACAGGATTCAATGGCCCTGAAAATGACCTTGCAGATGTAGAAGATTTTCCATCAAAAGAACAAATTCCAGAGGTCCCCATCCGCCGCCTTGCCTATCTCAACAAGCCAGAACTTCCAGTTCTTATTGTTGGCACTATTGCTGCAAGCATAAATGGTACAATACTTCCAATTTATGGTATACTGATCTCGAAAGCAATCAAAACATTTTTTGAACCACCTCATGAACTGAGAAAGGATTCAAAGTTCTGGGCACTGATGTTTACGACCCTTGGTCTTGCGTCATTTGTGGTACACCCATTCAGAACATACTTCTTTTCTGTAGCTGGGTCCAAGTTAATACAACGGATCAGGTCCATGTGTTTTGAGAAGGTGGTTCACATGGAGATTGGTTGGTTTGATGAGCCTGAGCACTCAAGTGGTGCAATTGGTGCCAGGCTCTCAACAGATGCAGCAACAGTGAGAGCCCTAGTCGGAGATGCATTAGCTCAGATGGTTCAAAACATTGCAACAGCAGTGGCTGCTATGGTCATTGCTTTCACTGCAAGTTGGCAACTAGCATTCATTATCCTTGCATTAATCCCTCTGATAGGTGTCAATGGAGTAGTCCAAGTAAAGTTCATGAAAGGATTCAGTGCAGATGCCAAG**GTATGCCCAAAAGCCAATCAGCCTGACTTGGCTGTGGCAAGCTCAACTCAATCCCGGGGGAAATAAATAAATATTTTGATGATTGAAGAAGCTGAATACTCATCCATGGAGTCTGTCAAACTGACATTTCCAGTCAGACATCTTCAGACTGCTATTTGACAGATTATTGGTATCGTATAAATTTATATATTTAATTACTAAGACATAAATCTAAATGGTTGCAG**ATGATGTACGAAGAAGCTAGCCAAGTTGCTAATGATGCAGTCGGGAGTATAAGAACGGTTGCTTCTTTCTGTGCTGAAGAGAAGGTGATGCAACTGTATGAAAAGAAATGTGAGGGGCCTATGTGGACTGGAGTAAGGCTAGGATTGATCAGCGGCATAGGATTTGGACTGTCCTCCTTCTTTTTGTTCTGTTTCTATGCAACTAGTTTCTATGCGGGAGCAAGACTGGTTGAGGGTGGCCACATTACATTTGCAGATGTCTTTCAA**GTTGGTTATCTTTCCTATCTATTAACTTTGTTCTCCAATTACAGCATATTGTTCAGTGATATTGAAACACGTTTTCATCTTGCAG**GTTTTCTTTGCTTTGACTATGGCAGCTGTAGGAATTTCTCAGTCAAGTTCCATCGGTACTGATTCCACCAAAGCGAAGGCTGCTGCTGCCTCTGTGTTTGCAATCATAGACAGGAAGTCAAAAATAGACCCTAGTGATGAGTCAGGAACAACAATAGAAAATGTAAGAGGAGAAATTGAGCTTCATCATGTAAGCTTTAAGTATCCATCTAGGCCAGATATTCAGATTTTCCGGGACCTCAGCTTAACTATTCGTTCTGGGAAG**GTAACTTTTGTAGATATTTTGAATCCCATTTGTTTCAGGCAACTAGTACATGACAAAAAGCAATTAGAAATGATAATACATTAGAAAAATATTGATTTTTTTCCTTGTTTCAG**ACTGTTGCCCTGGTTGGAGAAAGTGGGAGTGGTAAATCTACAGTAATTGCATTGTTGCAGAGATTTTATGATCCGGATTCAGGTCACATCACACTTGATGGAATTGAAATTCAGAAGTTGCAATTGAGGTGGTTAAGGCAGCAGATGGGGCTCGTCAGCCAAGAGCCTGTTTTATTCAATGACACAATCCGTGCCAACATTGCGTATGGAAAGGAAGGAGATGCAACCGAAGCAGAAATTATAGCTGCAGCAGAATTGGCCAATGCCCACAAATTCATAAGTGGTTTACAACAG**GTAAGAAAACAATATAATGAATGATAAAACCACACCATCAACGTAGTTGGCCTATGCAGGGGTAATGTAAGAGAGAATTTTCTGAAATAGATGTTAGATTAAAATAAAATGGCAACGCAAAATGCAG**GGGTATGAAGCCGCAGTAGGGGAGCGAGGAGTCCAGCTGTCTGGAGGACAGAAGCAAAGGGTAGCCATAGCTCGTGCCATAGTCAAGAGTCCAAAGATACTGCTATTAGATGAGGCCACCAGTGCTTTAGATGCTGAATCAGAGAGAGTAGTTCAAGATGCACTAGACCGAGTGATGGTGAACAGGACTACGGTGGTGGTAGCTCATCGGTTATCGACGATCAAGAATGCAGATGTTATTGCAGTGGTTAAGAATGGAGTTATTGTGGAGAAAGGAAGGCATGAGACTCTGATCAACATCAAAGATGGTTTCTACGCCTCCCTGGTAGCACTTCACATGAGTGCTCAAACTGCATAAGCTATAATTAGGTTTTCTATTTATGGTCAAAAGAGACTCGAATTCTACGAATAGAATGGAACTTGAAATGTAAATGTTTCCTTCTCTTTCTCTCTCTTTGATTGAGATTAATAGTTGTTAAGTGCAACATATTTCCTGATAAATTAATAAAAGC**

>HbABCB13 scaffold0563(296238-301733)

**GCTTCACTTTTGATATCAATTTTCGCCATGGAAGCGTTGGAGCTTGCTTCTGATCAGGAATTAGATCAGAATTCACGTTCAAAAATGGACCAAGAAAGGCCAAGTATTTCTTTCTTTGGTTTATTTTCTGCTGCTGATAGAATTGATTATTTCTTGATGTTTTTGGGAAGTGTCGGGTCATGCATCCATGGTGCTGCTCTTCCTGTATTCTTTATCTTTTTTGGTCGTATGATTGATTCTCTTGGAAATTTAGCTTTGGATCCTCACAAAATGTCTTCACAGGTTTCCAGGGTATGATCTGAACTAAATCACTCTGTGTTCTGTTACTTTAGTTTGTTAAAATTAAGGGATTCAAGCAGTTAATCTTCCAAATCCTAGCCTGTTAGTCCTTGCATTTCATTTTTCTGGATGAAAAGAAAGCATTTGAAGTCTATCATTTGTTGCAGCATGCGTTGTATTTAGTATATCTGGGGCTTGTCGTTTTTGCATCAGCTTGGATTG**GTATGTTTCTTGATTCCAGTCTTTTTCAGGAGAAATGTTTAGCTTTTTCTCAGGAATTAGCATCATTGTTAATATTCTCCATTCAG**GTGTAGCGTTTTGGATGCAAACTGGGGAAAGGCAGACAGCTCGTTTGCGACTCGAATACTTTCAATCTGTATTAAAAAAGGACATGAATTTTTTTGACACAGAAGCAGGAGATTCTAACATCATATATCACATATCCAGTGATGCAATACTAGTGCAAGATGCAATAGGTGACAAG**GTAATTCATAAACTCTCAGTTTGAATCCTGAGACAAACATTTTTACACGAATAATAACCATCACAAACTGTTTCTGTGTGTCCTGATACAG**ACAGGCCATGCCATACGCTACCTTTGTCAATTCATTGTCGGATTTGCTATTGGATTTGCATCTGTGTGGCAACTTACACTCCTCACCTTGGCTGTGGTTCCATTGATAGCTATTGCTGGGGGAGCTTATACTGTAATTATGTCTACCTTATCAGAAAAAGGTGAGGCGGCATATGCTGAAGCTGGGAAGGTTGCAGATGAG**GTATATCACTTGGCTTCTCGATTCAAATAAATTGAACTGCTTATGCTGAACCTAAAAGGTCAGAGAAGAGCTTATCCATGCCTGTTTTCACTTTCATATTTTCTCCCCCATTTTTATGAGTAG**GTTATTTCTCAAATCCGCACGGTATATTCATTTGTGGGAGAGCATAAAGCAATAGAAGCATACTCGAGGTCACTTACGAGCGCACTTAAGTTGGGGAAGAAAAGTGGGATTGCAAAAGGAGTTGGTGTTGGATTTACTTATGGACTATTGTTTTGTGCCTGGGCAATGCTTCTCTGGTATTCCAGCATACTTGTAAGGCATCACATCACAAATGGGGCAAAAGCATTCACAATGATTATCAATGTCATTTTTAGTGGATT**GTAAGTGTTATCTGAGTCATATTAGCAGTTAGTATTGAGGTTTTGTTACTGTTTATCCATTTTTATCCTGATGAAAACAGGCCTTTTGTATTTATAG**TGCTCTTGGTCAAGCTGCTCCAAACCTTGCTGCTATTGCTAAAGGTCGGGCTGCGGCTGCCACTATCATTAACATGATTGAAACAGGCTCTAACCCTTCTAAAAGGTCAGAACATGGATCAGAATTGCCAAAAGTAGAAGGGAAAATTGAATTTTCCAATGTCTGTTTTGCTTATCCCTCACGACCCAGTAAGGTCTTTGAAAATCTGAGCTTTACAATAAGTGCTGGGAAGACCTTTGCGGTTGTCGGCCCCAGTGGCTCAGGAAAAAGCACTGTTATATCCATGGTTCAACGTTTCTATGATCCCAATTCAG**GTATCTGTACTACATTACAGATTGCAGAATTTTCATTTTCCAGCTAGTGTTTTCCTTTTTTGAATGCTGATGGTCAGTTATAAATACTTGTGTGGCTTATGCACCAAAATTCTCTAGGTAAAATACTATTGGACTACTACATTACAGATTGCAGAATTTTCATTTTCCAGCTAGTGTTTTCCTTTTTTGAATGCTGATGGTCAGTTATAAATACTTGCACCAAAATTCTCTAG**GTAAAATACTATTGGATGGACATGATCTTAAGACTCTTCGGTTGAAATGGCTGAGAGAACAGATGGGATTGGTGAGCCAGGAACCTGCATTATTTGCCACAACCATAGCTGACAACATTCTGTTTGGTAAAGAAGATGCAAATATGGATAAGATTGTTCAAGCTGCCAAAGCTGCTAATGCACATTCTTTTATCCAACAATTACCTGATGGCTATCATACTCAG**GTGTGTCCCATGCAAAGTCTCTTGTAGATGTAGCATAAAACTGCAAATAGATTATATATTTTACCATGGCTGCTAATAAGGCTGGACATCAATGGTAGAATTTATCAGAAAACAATCTGTTTTATATGATTCCATCCTTTCTCTTCTGATATTTGATGGTTTCTTGAATCTAG**GTGGGGGAAGGAGGAACTCAGCTCTCTGGTGGACAAAAGCAAAGAATCGCTATAGCAAGAGCAGTGTTGAGAAACCCAAAGATATTACTTTTTGATGAGGCTACTAGTGCTCTTGATGCAGAATCAGAATTCATTGTTCAGCAGGCACTGAATAAAATCATGTCAAATAGGACTACAATTATTGTTGCACACCGCCTATCAACAATTCGAGATGTTGATACCATCATTGTCTTGAAGAATGGTCAAGTTGCTGAGAGTGGGAGCCATTTAGACTTGATAACAAAGGGAGGAGACTATGCAACTCTTGTGAGCTTGCAAGTTTCAGAACATCCAACACGGTCAAATTCAATAGGTGGTTCTGAAGCTTCTGGGAATTCTAGCTTTCGACAACTTCCCCACAGCCAAAACAACCAGCAGGATTTTAAGTCGATCTCAATAAGAGAGCTGCAATCAAAAGATGATGGTATGCCTTTACAAAAACATTCTCCCACTCCATCGATTTTGGAACTACTAAAACTAAATGCACCAGAGTGGCCTTGTGCTTTGCTTGGGTCACTAGGTGCAATTTTGGCTGGCATGGAAGCTCCTCTGTTCGCCCTGGGGATTACACATGTATTGACAGCATTTTATTCCCATGATGCGTCTGAAATGAGACATGAGATTCAACGGATATCTCTTATTTTTGTTGGATTGGCCGTTGTTACCGTTCCTATATATCTTCTGCAACACTACTTTTACACGTTGATGGGAGAGCGTCTCACCACTCGCGTTCGCTTGTCAATGTTCTCAG**GTTTCTTTTCTGCTTGCTTCTTCATTTTAATAATAAAATATATTTTTCAGAATTTACATGATTCTGAAACTTTTTCTTGTTTCAACTGCAG**CTATCCTTTGCAATGAGATTGGCTGGTTTGATTTAGAAGAGAATAACACTGGCTCGCTAACATCAGCTTTATCTGCTGATGCAACCTTAGTACGCAGTGCTCTTTCGGACCGACTCTCAACAATTGTGCAGAATGCAGCACTTACAGTAACAGCATGTGCTATTGCTTTCACATTGAGCTGGCGCATAGCAGCTGTTGTTGTTGCCTCCTTACCTCTGCTCGTGGGAGCTTCCATAGCTGAG**GTATGCCTTTGAGATCACCATGATAACATCAGGGTGATTCAATCATTTATTTCATTTCAAATATCCACACACTAAGTACTCTTATTTTGAAAATCTGCAG**CAACTATTTCTCAAGGGGTTTGGAGGAGACTACCATGCGTATTCTAGAGCAACTGCAGTGGCACGTGAAGCGCTCACCAATATTCGTACTGTTGCTGCATTTGGGGCTGAAGAAAGAATCTCTGTTCAATTTGCCTCTGAACTCAACAAACCAAACAAGCAAGCACTTTTAAGGGGACATATTTCGGGTTTTGGCTATGGCCTTACCCAGTTATTCGCTTTTGGTTCCTATGCGCTCGGCCTTTGGTATGCATCAGTCCTAATCAGACACAAAGAATCCAATTTTGGACATATCATAAAGTCCTTCATGGTTTTGATAATCACTGCATTGGCTATAGCAGAAACGCTTGCTCTTACACCAGACATTGTGAAGGGTTCACAAGCACTTGGATCCGTTTTCAATGTTCTCCACAGGAAAACTGCTATCGACACTAACGATCTCTCATCGAAGGTGGTAACTGATATCAAAGGGGATATAGAATTCAGAAATGTGAATTTCAAGTACCCTGCCAGGCTTGATATCACCATTTTTGAGCTTTTAAACCTCAAAGTTCCAGCAGGCAAAAGTCTGGCTGTGGTAGGCCCAAGTGGTTCGGGGAAGAGCACTATAATTTCCCTGATACTGAGATTCTATGACCCCATTTCTGGGACAGTCCTGATTGATGGATGTGATATTAAAACCTTGAATTTGAAATCACTGAGGTTAAAAATAGGTCTGGTTCAGCAGGAGCCAGCACTGTTCTCCACAACTATTTATGAAAATATCAAGTATGGAAACGAAAATGCCTCAGAAATTGAGATAATGAAAGCAGCCAAAGCAGCAAATGCTCATGGATTCATCAGCACAATGCCTGAAGGGTACAAGACTCATGTGGGTGATAGGGGACTGCAATTATCTGGGGGGCAGAAACAAAGAGTGGCTATTGCTAGAGCAATACTGAAGGACCCTTCCATTCTTCTATTGGATGAAGCTACTAGCGCATTGGATACTGCATCAGAGAAGCTGGTTCAAGAGGCTTTGGATAAGCTTATGGAAGGACGAACAACAGTTATGGTAGCCCACAGATTGTCCACCATTCGTGATGCAGATAGCATTGCTGTGCTACAACATGGGAGGGTGGCTGAAATTGGCAGCCATAAACAGCTCATGGGAAAACCTGGTAGTATTTATAAGCAATTAATTAGCCTGCAACAAGAAGAGAGCATACAGTCATAAGTAATTATCTTCAACAACTCAACTATTGAGTAATAAGGACTCCAAGCCTAACAAAATCTTAGTTGCCTGTAAATGAAGTTATGCATATAAGAACTTCCATTTAAAACTGAGCATATTCTTCAGGCACTAAGTTGTTTGCAAGAGATTTTTGTATACTTGAAATGCATCACTGAAAGGAATGTTATTGTAATCTGTTGATACTACAGGCGTTTGATGTCTTAGAATTTTGATACATTTTTCTCTGCAACTGAGTTCAGAAGTACCATTTATATTGTCCTTTAAATCAATGACAGAAGACGACAATCTGTAAAAAAATAGCTTGAGGCAAGCCTGCTGCCTGATCACTTGCCTGCCTGATTAAAAAACAATAAGTAAACTAGTTGGTGCTCATTGACCAGATTTAAATATGAATGCTTATGCTGAAGCTACAAGGTTGCAGAAGAATTTATCCATGTCCTCTTCCTGCTTCCTTTTTCCTAGCCTTCTTTTTCATGTATGAGGTTATTTGGCATTGTAGACCCATTTGTTAAAGAGGATAACAATGCAAGTGAAGCATACTCAATTTCATTTCAGAATCA**

>HbABCB15 scaffold0015(154054-160164)

**ACGACACGGCCCTTTTGGGAGCAAACTGCCAGTGACTTTCTCTATTTCTCTCGCTCTCGGAGAGTACTTGTAGCTCATAGCCAGTTAGTTATTGTTAGTTCAAATGGTGAAGAAGAAGAGTAGCCATGTTGGGTCCATAAGATCAATTTTCATGCATGCAGATGGTGTAGATTGGTTTTTAATGGTTCTTGGGGTCATTGGATCTGTTGGTGATGGCTTCTCCACCCCGCTTGTGTTGTTTGTTACAAGCAAATTGATGAATAATATTGGCGGTGCATCTTCTTTCCAGAGTGATTTCTCTCACAACATTAATAAG**GTTAATTAATCTATTTTCCCTTTCACTTCGTCTTTCTCTTTCATTTAAAGCTAACAAACTACTTTTGGATTCCTTTGGCAG**AATGCACTGGCGCTCTGTTACTTGGCTTGTGGACAATGGGTTGTTTGTTTCGTAG**GTGGGTTCAAATTTGATACTTTGATAATTACGATTCTTGTTGGCTTGTTTTGATGGATTAATTAAGGAGAGATTAATTATGGGCTTGGATTATTTGTTTGAATATCAG**AGGGATATTGTTGGACAAGGACAGGGGAGAGGCAAGCTACAAGAATGAGAGCAAGGTATTTGAAAGCGGTGCTGAGACAAGAAGTGGGTTATTTTGATTTGCATGTAACAAGTACGGCAGAGGTCATCACAAGTGTCTCTAATGATAGCTTCGTAATTCAAGACGTTCTAAGTGAGAAG**GTTTGTTTATTTCTGGTGTTTGATTGAAAAATTTTCACCTACCAAGGAAAATTGAGAAATGTCAATATTTTTTACTAATTAATTTTTTATCATTTATGTTAAATCAG**GTGCCAAATCTTTTGATGAACGCTTCCATGTTCTTTGGTTGCTATCTTGTTGGGTTCTTGCTGCTTTGGAGGCTAGCAATTGTGGGGTTTCCTTTTATTGTCATTTTGGTTATTCCTGGTCTGATGTATGGGAGGACTTTGATGGGTTTGGCAAGAAAGATCAAGGAAGAATACAACAAAGCAGGTACAATTGCAGAGCAGGCGTTATCTTCCATCAGGACTGTCTATGCCTTTGTTGGTGAATCCAAAACCGTAACTGCATATTCTGCAGCTCTTGACTTTTCAGTGAAATTGGGGCTCAAGCAGGGCCTGGCAAAGGGATTAGCCATTGGCAGTAATGGTGTTGTCTTTGCTATTTGGTCTTTTATGTCTTATTATGGTAGCAGATTGGTCATGTATCATAATGCTCGTGGAGGGACTGTTTTTGCTGTTGGTGCCTCAATTGCTGTGGGTGGATT**GTAAGTTTCTTCTCTCTTTTCGTATAATCTTTGAAAGGTTTCTTTCTTGCTTCGCATTATAGTAAAATAAAATAATATAAAATAAAAAGATATTACTGTAGGTTGCTTTATTATCATGGTGTTAGTTAATGCGTTGCATAACATACAGACAAATCATTTTTTTTTTTTTTATCTTTGTCTAATTTCATGTTTCTTAATTTGTTGAACGCTAG**GGCCCTTGGTGCTGGTTTATCCAATGTTAAGTACTTGTCAGAAGCATGTACAGCGGGAGAACGGATCATGGAGGTGATAAGAAGAATCCCCAGAATAGATCTAGAGAACTTGGAAGGTGAAATTTTGGAAAACGTAGGAGGTGAAGTAGAATTCAAGCATGTAGAATTTGCATATCCATCAAGGCCAGAAAGCATTATCTTCAAAGATTTTACCCTAAAAATTCCAGCAGGGAGAACTGTGGCCTTGGTGGGGGGTAGTGGCTCAGGAAAATCAACAGTAATAGCACTGTTGCAAAGATTTTATGACCCACTTGATGGGGAAATACTTCTTGATGGGGTGGCCATTGATAAGTTGCAGCTCAAGTGGCTAAGGTCACAGATGGGTTTGGTTAGCCAAGAGCCAGCATTATTTGCAACAAGCATTAAGGAGAACATACTTTTTGGCAAAGAAGATGCCACAATGGAAGAGGTTGTTGAAGCTGCCAAAGCTTCTAATGCTCATAATTTCATATGTCAGTTGCCCCAGGGGTATGACACCCAG**GTTAGTTTCTGCTTGTCGTTTAGCTAGCCACGGCCACAACCACTACACTAAAAACATTTGACTTGCAAAATTTTTATGCTTGCTAGGCATATTACAGAATTTGAGATCTCACATATTCTTTCAAAAAACGACAACCCTGCTTGTCCTATTTTATTTCTATGTCCTCTGTACCTAGTCATTATTTCTGTTTTATTGTCTGAGAATTGTAATTTTGTATTTTTTTTTAATATTTTACCTGATATATTTTTTTGCCAAATTCAAATCCAAACTGTGTGCTAGCTAGGGACATGGACCCCCACATAATTAAATGACCCCATACCTTCCTGCAAGGTGCTATTGCTTTCCCAAGTCCTAAAAATGGGCACCCATAGTGTTTCCATCATTTGGTAGCAGATTGTATGAATCCAGTGCCTATCAGTGTCACCGCTCTCAATTTTTTTTTCCTTTAATTAATACACCTTCTGTTGAAAATCTTGCCTAGAAGAAGGAAGGTAAAGCAAGGCAAGGGATACCTTTCAAAGTGGGAAAGTGCTTCGGGTCACCTCCACCCAAGATTCCATAAACGTAAATTGGAACAAGATAAGGACACGCTCCATGTTTCCTTGTTTTCTACCCATTTATAAACAAAGTTTCTTTATTTTTTTTTTATATATCTAATATTTTTAGAATAGGGTGTCAGAATTAGAAATTTTCATCTCTGGCATTATCTTTATTAGATATTATTTTCAGGACACTTTCTTAGCAAAGACACGAAAATTTCTATGTTTAACTTTATCAAGTTGAATGATAACTATTTCATAAAATGATTGGCAAGTTGATTGGCTCAAATTCTTTTTTTAATCTTTGTCTTGATGAATGAATAAACTACACATCATGCATATCTTAATCATTCATCTTTATTCTAAGATTTAGTTAGCAACCAATGAAAAATACTAATACATGATTCACAG**GTTGGCGAGCGAGGAGTTCAAATGTCTGGAGGACAAAAACAAAGAATTGCTATTGCAAGAGCAATAATCAAAGCACCAAGAATCCTCCTTCTTGACGAGGCAACAAGTGCATTAGACTCAGAATCTGAACGCATTGTCCAACAGGCACTGGACAAAGCTGCCATTGGCCGCACCACAATCATCATAGCTCACCGTCTCTCGACCATCCGAAATGTGGATGTAATTACCGTGGTCCAAAATGGCCAGGTGATGGAAACAGGTTCTCACGATGAATTAATGGAAATTGAAGATGGACTTTACACAACCTTAATACGTCTTCAACAAACAGAAAAAGAAAAATCTAATGAGGATGATCAGTACCATATACCCTCTTCATCTTTGATCTCAAAAATGGACATGAATAATACTAGCAGTCGCAGGTTATCAATGGTGAGTAGGACAAGCTCTGCCAATTCAATTGCTCCAAGTCGAGCTTCAGTGAATGCAGAAAATATACAATTGGAGGAGCAAAAATTTCCTGTACCTTCATTTCGTAGACTGCTAGCTTTGAACTTGCCAGAATGGAAACAAGCAAGCTTCGGGTGTTTAGGTGCAATTTTATTTGGCGGTGTTCAACCCTTATATGCATTTGCAATGGGGTCAATGATATCTGTATATTTTTACACGGATCATGATGAGATTAAGAAGCGAATAAGGATTTACTCACTATGCTTTCTGGGGCTCTCCATATTTACATTCATCGTCAACATCGTTCAGCATTACAATTTTGCATATATGGGTGAGTACTTGACCAAGAGGATAAGAGAGAAGATGCTTTCAAAGATGCTCACTTTTGAAGTTGGCTGGTTTGATCAAGATGAGAACTCAAGTGGTGCAATTTGCTCTAGACTGGCCAAAGATGCCAATGTG**GTGCGTTATTTTTTTAATTTATATTTGATTAGTTTAAAGTTAAATTTGATATATTTTAATCTTAAAATTTTTTAGAATAATTAAAGAATTTTTTTCAATGATATTTATATGATATTTTTTTAAAAAACAATTTTGTTTTTTTGTTTTTATTTTCCAATTGAACAAAGTCAACTTCTTGAGCTTCGGTTGCTAGGTGTGATTTTGACAAGCCTTTTGACTCTACTAAGAACCTCACAGTACCACTCATTGGGACTAAATTTGAATGGTTGAAAGCTACTTTGTACTTCCAATTCACATTAATATTTAGCTCCAAGATTACTCTATCATCATTGTAACACTAAACCCAGCTGCCTTTTTTTTAATATTTTAATTTTAAATTGTATATGTGGCACCTAACTAATCATTTGATGAGGAGATTCCTTGCCTTAACCTGACCAATCATGACAATTTTCTAGTATCAAATTGATATCTCCTTTATATGGCGAAGTCAAAGGCCAACCTATTGTTACTAGTTTTGTTCACCAAATAATGATAATATATTGGCCAAAATACAATTTTTCTTTTTTGAATTATTCAACCATATTAGATTTTGGACCCTTGTGGCCTGAGTATGTATTTTTGTTCATGGGCAG**GTGAGATCTCTAGTGGGTGACCGAATGGCTCTTGTAGTACAAACGGTCTCAGCAGTAGTCATAGCCTGCACAATGGGCCTTTTCATTGCTTGGAGGCTTGCAATTGTGATGATTGCAGTCCAACCTCTCATTATTGTCTGTTTTTATACCAGGCGTGTACTACTCAAAAGCATGTCCCATAAGGCCATCAAAGCCCAAGATGAAAGCAGCAAGCTCGCAGCAGAGGCCGTGTCCAACCTCCGAACCATCACTGCCTTCTCTTCCCAAGATCGAATCCTTAGAATGCTTGAAAAAGCCCAAGAAGGTCCACTGAGAGAAAGTATTAGACAATCATTGTTTGCCGGTATTGGGCTTGGCACCTCTCAAAGCCTAATGTCATGCACATGGGCCCTTGATTTTTGGTATGGTGGTAAGCTCATTTCCAAGGGGTACATCACAGCAAAAGACCTATTTGAGACGTTCATGATCTTGGTAAGCACAGGCCGTGTCATTGCTGATGCGGGAAGCATGACCACTGACCTAGCCAAGGGCTCAGATGCTGTTGGGTCTGTCTTTGCTGTATTAGATCGGTACACAAAGATTGAGCCCGAAGGTGCTGATGGGTTGAAGCCTGAAATGATAATGGGCCACGTAGAACTACGAGATGTAAACTTCGCATACCCAGCTAGGCCTGATGTGATCATCTTCGAAGGTTTCTCAATCAAAATTGAAGCGGGAAAGTCAACGGCATTGGTGGGACAAAGTGGGTCCGGTAAGTCAACCATCATTGGGTTAATTGAGAGATTTTATGATCCCATCAGGGGCATAGTGAAGATAGACGGTCGAGATATCAAGTCATATCACTTGAGATCATTGAGGAAGCATATTGCTTTAGTAAGCCAAGAACCCACATTATTTGCTGGGACTATAAGGGAAAACATTGCGTACGGTACATCTAAAAATGATGAATCAGAGATCATCGAGGCCGCTAAGGCAGCCAACGCTCATGATTTCATCGCAGGATTGAAAGATGGATATGACACGTGGTGTGGGGACAGGGGAGTGCAGCTCTCAGGTGGGCAGAAGCAACGTATTGCAATAGCCCGCGCAATACTGAAAAATCCTACAGTATTGCTATTGGATGAGGCGACCAGCGCGCTTGATAGTCAGTCAGAGAAGGTGGTGCAAGACGCGTTGGAGCGCGTGATGATTGGGAGGACAAGTGTGGTTGTGGCTCATAGGCTAAGTACTATACAGAACTGTGATCTTATTGCCGTTTTGGATAAAGGACAGGTGGTGGAGCAAGGGACCCACTCGTCCTTGTTGGCAAAGGGACCCACAGGAGCTTACTTCTCATTGGTAAGCCTCCAAAGGACGCCGCACAACAGCACCACTACTGCTAGCCATACATTCAATTGAGTGGAAGCCTAATAGAGTTGGTTTTTCTTGCCATCAAAATGGTGTAAAATGAGAACCATCTTATTTTAAGGCATTTTTCTTGTTTTGTAATAATAATGATAAGTTTATATGTAAGTTGTGTATATAGAAGAAAGAGATAATGCTAATAAAGCCGATCTTTCTAA**

>HbABCB19 scaffold0875(24453-31902)

**TTTTGGGGACCAAAATGGCAGCAGAGACTGTAGATACTACCACTACTACTAATACTAGTTCTAAAGCATCATTGCCAGAGGCAGAGAAGAAGAAAGAACAAAGCTTGCCATTTTACCATCTTTTCTCATTTGCTGATAAGTATGATTGGCTGCTTATGATCTCTGGTAGCTTAGGAGCCATTATTCATGGCTCTTCAATGCCATTCTTTTTCTTGTTGTTTGGTGAAATGGTTAATGGGTTTGGGAAGAACCAATCTGATTTGCCAAAAATGACCCATGAAGTCTCCAAG**GTCCGTTAAATTCCATTGCTTTTATATATATATATATATATATATATATATATCTTTTGAAGAGTTNNNNNNNNNNNNNNNNNNNNNNNNNNNNNNNNNNNNNNNNNNNNNNNNNCAAAGAGAATTAATATAATTTTAGATCTGAGAGATCTAACGCTTACTTTTCTTCCTGCCCCTTCTTGCGGTGATTGCAG**TATGCACTATATTTTGTATATCTTGGCCTTGTTGTGTGCATATCATCATACGCAG**GTATGTTTAGTTTTCTTCTTGGTTTTGTTGTTTTATGAACGGAGTTTGTGGTAAATGAATCACAGGGCCACACTTGGTTGGTGCACCACATGGTTCAGAACAGAACAAAATATGATTCGTGTAGCCTGTTCAAAACGGACCGGAGGGCTGCAAAGCTTAGCCCACGTGGTCCATGGACAGACCTTTCATCCTTTTTCTCCCTTCACAGCTTCACTTGTCAATTTGATCACTTTCCTTTTTCTTCCTATCTCTTAAAACGTTATCTCCACCTGTCTCTTTTCATGGGTTTAATTTGCATGTATACCCTTTTAATCCAACCTGATTGTAATTAAACTAATTCTTGTTCTTTGAAATGGTGGAATTAACCAG**AGATTGCTTGCTGGATGTACACTGGGGAGAGGCAAGTAGGCACACTGAGGAAGAAGTATCTAGAGGCTGTGTTGAAACAAGATGTTGGGTTCTTTGATACAGATGCAAGAACTGGAGATATTGTCTTTAGTGTCTCAACGGACACTCTTCTTGTCCAAGATGCCATCAGCGAGAAG**GTATTTTTTTAACAGACTTTTCTGCTATTCTCATCTTGATGATGTTTCTAGTTCATTAATTGCTTCTTGGTTTTGTACTGTTGCGTGTGGGGCAAAATCAAGTGTCCCGTTGCGGCTTGGCGTTAAATTGCAGAAAATGCTCATTCTGAATTGAATTAATGCGGGGCCCATGCTAAAATTTGACTAGCGCACGTTAGCAAGTCTGACTTAACGTGCGCCATACTCCCATGTTGGACTTTTGTGATATTGTAGTGGTGGAAATGAGAATTCTTGTGTTTATAATTCATGGTGGATTAGGTTCTCCCAAGAATTTCATGCAGTGTGATACTATTGTTGATGATAGTTTCTTTTTTTGTTCTTAATATACTAATTGTTCGTTAATTTTAG**GTGGGGAACTTTATACACTATTTGTCAACATTTCTAGCCGGGCTAGTGGTTGGTTTTGTATCAGCATGGAGGCTAGCACTGCTAAGTGTGGCAGTGATACCAGGAATAGCTTTCGCTGGTGGCTTATATGCTTATACTCTTACTGGTCTCACATCCAAGAGCCGCGAATCCTATGCACAAGCCGGCATAATTGCCGAGCAG**GTGAGGTTTAATTTAGAAAAGCTACTTCAATTTGGAAGCTTTGTTGAGGTGATGCTATATTGTTCTTTTTGGGTACTATATGCTTCCATGTATAGCTTGTCTTGATTCATTCTTTGGATTATGACAAGGAAAACACAGCTGTATTGATATCACGTTAGTTTTTATATTTTGTTAAGCATGGAAAGCTGCAAGCTATGCTGTTGTTGGAAGTGGCACTTTTCTAAGCTTTATCGATTTTCTTTTTATTATTTTTTGCTCCAATTTTGGCTTTTAGACCCTAAATTGTTGCCAAATTGTATCATACGACTGTAATTACAAAATAATCAAACATATGAACTGCACTAGTTTTCTATGTTCTGCATTCTTTTTGGCTATCTAAATTATCTATCTCGTTGCATAG**GCAATTGCGCAAGTTCGGGCAGTTTACTCATACGTTGGAGAGAGCAAAGCCTTAAATTCATATTCAGATGCAATACAAAATACTTTAAAGCTTGGATACAAGGCTGGGATGGCTAAAGGTTTGGGGCTGGGATGTACCTATGGAATAGCTTGCATGTCATGGGCCCTTGTGTTTTGGTATGCTGGTGTTTTTATCAGAAATGGACAGAGTGATGGAGGGAAGGCTTTCACTGCGATTTTCTCTGCCATTGTTGGTGGCAT**GTAAATATCAAAATACTGAAATGCCAGGATTTAGATTTTTTGATAAATTATGAAATAAGCTTGGTAATAATATAAATGATATTGTCTTTTACAG**GAGTTTGGGCCAGTCATTTTCTAATCTTGGGGCATTTAGCAAAGGTAAAGTGGCTGGATATAAGTTGATGGAAATTATTAAGCAAAAGCCTTCCATAATTCAAGACCCCTCTGATGGGAAGTGCTTGCCTGGGGTTAATGGGAACATAGAATTCAAGGATGTAACTTTTAGCTACCCATCAAGGCCAGATGTTATGATATTCCGTGATTTCTCAATCTTCTTTCCTGCTGGAAAGACTGTTGCTGTTGTTGGTGGTAGCGGGTCAGGGAAAAGTACTGTTGTCTCTCTGATAGAGAGGTTTTATGATCCTAATCAGG**GTAATTAAGAAGCTTGTCTAAATTGAAAGTTTGAAATTTCACAACAATAGCTGGATGATTTTGCTGATGTTTCTTACTGGTGAGCAG**GACAAGTTTTGCTTGATAATGTGGACTTAAAGACCCTGCAACTGAGGTGGTTACGTGATCAGATTGGGTTGGTGAATCAAGAACCTGCGCTCTTTGCAACCACCATACTTGAGAACATTTTGTATGGAAAGCCTGACGCCACAATGGATGGAGTGGAAGCTGCTGCTTCTGCTGCAAATGCACATAGCTTTATTACTTTGCTTCCTAATGGTTATAACACTCAG**GTTGGTATATGTAAGAAAAATTAGAGCAAATATTATTTCACTATGATAAGTCATGTAAATATTTTCATTTGATGCTTCACCACTTCAGTAATTCTGACCTTGATTTTTGTGAGAAAAAAAAAAACGGAGAGAATTCATGTTTTGGGTAAACATAATTGCACCTTTTACTGTATCTTGAGGCTACGAATACTTTATGCTGGCCAG**GTAGGGGAGCGAGGAGTCCAACTTTCTGGTGGCCAAAAACAAAGAATCGCAATTGCTAGAGCTATGTTGAAGAATCCAAAGATCCTACTTCTTGATGAAGCTACTAGTGCCCTTGATGCAGGCTCTGAAAGCATTGTTCAAGAAGCATTGGACCGTCTCATGGTTGGAAGAACAACCATTGTTGTTGCACATCGACTCTCCACCATTAGAAATGTTGATACAATTGCAGTTATTCAACAAGGGCAAGTTGTTGAGACAGGAACCCATGAAGAACTGATTGCCAAAGGAAGAGCATATGCTTCATTAATCCGATTCCAAGAAATGGCGAGAAATAGAGACTTTGCAAACCCATCAACTCGAAGATCACGCTCATCACGTCTAAGTCATTCACTTTCAACAAAATCTTTAAGTCTCCGGTCTGGCAGTTTACGGAACTTGAGCTATTCATACAGTACTGGTGCTGATGGCCGCATTGAGATGATCTCAAATGCTGAAACTGACCGTAAAAATCCAGCACCAGATGGCTATTTCTGCAGACTACTCAAGTTAAATGCTCCTGAATGGCCATATTCTGTAATGGGTGCTATAGGATCTGTGCTTTCTGGGTTTATTGGTCCAACCTTTGCCATAGTGATGAGCAACATGATTGAGGTTTTCTATTATAGAAATCCTGCCTCAATGGAAAGGAAGACAAAGGAGTATGTTTTCATTTACATTGGCGCTGGACTTTATGCTGTTGTTGCATATTTGATTCAGCATTACTTCTTCAGTATTATGGGAGAGAACCTTACTACAAGAGTGAGAAGAATGATGCTGGCAG**GTAAAATTAACTGTTATGTTTTTGCTGTTGTTGTCTCATTATTTTACATTCATTATACTTCAATCTAGGAGTAAATTTCCTGATTATGATTCTCTTTAATTTTTCCTTCAG**CAATTCTGAGGAATGAAGTCGGATGGTTCGATGAAGAAGAGCACAACTCGAGCCTTGTTGCAGCTCGATTAGCTACGGATGCTGCTGATGTAAAATCTGCAATAGCCGAGAGAATATCTGTGATACTACAAAACATGACCTCTCTCCTCACTTCATTCATAGTTGCTTTCATAGTGGAATGGAGGGTCTCCCTTCTCATTTTAGCTACCTTCCCTCTTCTTGTCCTTGCCAACTTTGCCCAG**GTAAGTTTAGAACCATGCTGTGCTGCAAAAATGCGCAAGCATTCTTTTCTTTTTCTTTTTTGCTTGTCATTCTGATAACCATGTTAGGTGGGGTAATGGGTTTGGTCCAAAACCAATACCCAGCCTTAAAGGGCAATTAATATTTTCTTGGGTTCTGCAAGGGCAAAAGGGTAAATAAGAAACTCCCTATTTCATTAATTTTTCTACAGCGGTGTCGGATCTCTGCATCTCCTTTTTTTTTTTTAATCTCTGGAATCTAACCTCCTACCATTAAACGACAAAAGCTAAGAAAATACGTCGTGTCACTCATGTATGAACTGCACATGCTCCCTACAACTCCTCAAAAACCCCAACATAAATATATGTTAATATATTTTAGAGCTCGTGAATAACAGTGAACTATTGTAAAATACAAGATTCCATGCCTAAACCCATCAATTTCCATGCCACTGAGAAAGTGTTCTAGTTTATGCCTTCTCGAGTCCATGACCTTTTCTTGGATTTTTAATCCCAGTCAAATCTCATGTCTTGTTTGTTGTTTCTTTCTCTGGACTTTATTTTCTCTCAATCTATGCAGTGTATATATGTATATCAGTTCTAGATATACGAGTTTTCAGTGTTTACCAATTACATTGTATGATACTCCTAGATGGATGCCACCACCGAGCGAGTGTTTCTAAGGGATTCTTGGCCCGTGAATCACATTATTTATGACACATTTATTGGCACAGATTATTATCTTCTGCAGCTTTTCTTTTTAAAAATTTTTCTGGGTATTTTGGTGTTTTCTTTGGAGTGTCGGATGGGTGACAGAGTCAATAGTAGCATGGCAGTGGCTGAGTCACACCCTTTTCTTGCATTATTGTCGTGTTGATCTCTGTGGTCTTGTCGTTTTGAGCGTGAGTCTTGAATTTATGGGGTTTGCAGAATCTGTATTAGTTTTTCAACTGTGTAGGTATGATAGGTCCACAGTTCTGTCGTTGTATGTAAATTTGCCCATGCGATATGCACAGTGGATAAATTTATTGCTCGAGGATGTACCTCAAATATGCAGTGGTCACAGTCCTATATGTGCCTTATATGTGTAGGAGTGGACTCTAGCATGATTGGCTCCACTCCTTGTGGGTCCTTGCAGTCTTTATAGAGTTAGCGTACGATCATTTTTTAGATGCTACCAACTATTGATCACTCAAGGACATCAAGGTTCCTTACGCGAATGATAGAAATAAATAAGAACCAAAATTCTAGATTCGGTGACAATCTTTACATGGATGCTTGTCTCTTTTAGGAAGAGACTTTTCAGTTGACCAGTGCAAAACTCGGAAGTTCTTGTGCCAATCATGCTTAAACCATCATTTCTTAATGGGTCTTATTGTTTTAACATCTTAATTTGACAGATCTAAGCTCCTTGGACAATACCTAGAAATGGAATGAGAATACATTATTGAAACTTTATGCAAGGTGCTCCACCAATTAGCATGTCTATGTACTTAATTGGCATAAAATTCTCTGATGCATTGCATTTTAATTAGCAAATTCATAGGTGATTCAATTTCCAGGACGATTGATTATGTTATTTTCTTTGTTACACAG**CAACTCTCTCTCAAAGGGTTTGCTGGAGACACAGCCAAGGCCCATGCAAAGACTAGCATGATTGCTGGTGAGGGAGTGAGCAATATTAGAACTGTTGCAGCTTTTAATGCCCAAGATAAAATCCTCTCTCTGTTCTGCTACGAGCTTCGTGTTCCACAGCTTCGCAGCCTACACAGGAGCCAAACATCTGGCCTCCTATTTGGCCTCTCTCAGCTTGCTCTCTATGCATCTGAAGCTCTCATCCTCTGGTATGGTGCTCACCTTGTGGGCAAAGGGGTCTCCACATTCTCAAAGGTTATTAAGGTTTTTGTAGTACTGGTGATCACAGCTAATTCAGTAGCAGAAACTGTTAGCCTTGCTCCAGAGATTATAAGAGGTGGTGAATCTGTCGGTTCTGTATTTTCAGTTTTGGACCGTTCCACCAGGATTGATCCAGATGATCCTGAGGCTGAGCCTGTTGAATCAATGCGTGGGGAGATTGAGCTTAGGCATGTTGATTTTGCATACCCATCACGACCTGATGTACCAGTGTTCAAAGACCTTAGCCTTAGAATACGTGCTGGCCAAAGCCAGGCCCTTGTGGGAGCTAGTGGGTGTGGAAAGAGTTCTGTCATTGCACTGATTGAGCGATTTTATGATCCAACTGCTGGAAAGGTTATGATTGATGGGAAGGATATTCGAAGATTGAATTTGAAGTCCCTGAGGCTTAAAATTGGATTAGTGCAACAAGAACCTGCCCTCTTTGCAGCAAGCATTTTTGATAACATTGCCTATGGAAAGGATGGTGCAACCGAAGCTGAAGTAATCGAGGCAGCACGTGCAGCAAATGTGCATGGGTTCGTTAGTGCATTGCCTGATGGGTACAAAACACCAGTTGGTGAAAGAGGAGTTCAGCTCTCTGGAGGACAAAAACAGAGAATTGCAATTGCAAGGGCTGTTCTTAAGGATCCAGCAATACTTTTACTAGATGAGGCTACAAGCGCTCTTGATGCTGAATCAGAATGTGTGCTACAAGAAGCACTTGAGAGGCTAATGAGAGGTCGCACCACAGTTCTTGTAGCTCACCGTTTATCAACCATTAGAGGTGTGGATAGTATTGGAGTAGTGCAAGACGGTCGGATTGTGGAGCAAGGCAGCCATTCCGAATTAATTAGCCGAGTCGATGGGGCATATTCTAGACTCTTGCAGCTACAGCACCATCAAATATGAGTGAGAGGGAAAGAGGGTTTTGATGGAAGCAACATGGCCTTCTTGGTTGGTAATTATGTTTGGGGTCCCATTAACCTTTATGTTTCCATTTTTTTTCTTCTTTTTTTTGAATGATGATGACAGTTGTAGATATGGAATGTGTAGACTATATCTTGAGAACTATCACCCCATTTCCCATGGGTAGCAAGTGTTATATTATTAAATATCTAATGGTTC**

>HbABCB20 scaffold1096(9145-23417)

**TTCACTAAATCTCTCAAGATGATGATCTCTAGGGGATTGTTTGGGTGGTCCCCACCTCATATACAGCCGTTGACGCCTGTTTCTGAGGTTTCTGAGCCTCCGGAATCGCCATCTCCTTACCTGGATACATCTGCTGAAGCGGCTGCCGCTGCTGCTGCCGCTGCTCAGGCTGAGGCGGAGGAGGAGATGGAGGAGCCTGAAGATTTGGATCCTCCTCCTGCTGCTGTTCCTTTCTCGCGTTTGTTCGCTTGTGCTGATCGACTTGACTGGTGTCTCATGATCGTTGGCTCGCTTGCTGCTGCTGCGCATGGTACTGCGCTTGTTGTGTACTTGCACTATTTTGCGAAGATTGTTCAGGTTATGGGGATTCCTCCTGATAGACCTGAGGATCGATTTGACAGGTTCAAAGAT**GTGAGTATGAATATTTCGATATTTGAAAATTTTGCTTGTATTTTTTGTAGTTTCTGTGTTTGCTTTGTCGATGGGGAAATGAGGGAAGTGGAAGGAAGCTGAGGTACTGAGTTACGGGTTTTTGGAGTTTGTTGAGTTCAGGTGAATTGAGCATATTTGGTACTTAGCTGAGTTTGGTTGGCGGGTTAATTAAAATTAGGGATTTAGGACTTAGAATTGGAAATTGATGAAGTTTGGAATGGGATGATGTAGTTTGATAGCACTTCTTTCCTTTGTCTGTTCATTGGAAGGCAATATGGTCTTTTCTAGTGACAATTGGATGAAGAAGCAGATTTACATTTTGAGTTGAGTGTTTGATGAATTTTATGATTCTGCAGTTGATCTTAAATCTTGCATATTTAATGTTTTCAAGTTTTTTTTTTTTCATTTGTTGGGTTTGGTAACTACTAATGTAGCTTCTGCCGAGAAGAACACTATTTTGAATACGTTAATTAAGGGAACTAGAAAATGTTTTTAAGGTATATTTGACATGTTTTGGTCCGGAGAACTCCACCTGAGCTAAAACTTGTTTCAAAATCTTGTAAAATGGTTTTCTTTCTTTTTCCTTTTTCCTTTTTCCTCCCCCTCTTTCTCCCTCAAAAAAAAAAAAAAAAAAAAAAAAAAAAACAAAAGAAAACCATTTCAACTGCAATGCCAAACAAATGCTAGATGGGAAATTTCATGATATGGATAAATTGTCACAGTGTGCGACTCTGTGTCTGTGTTGAATTATAATGTATTGAAAACATTGGATGTCTGCATTTTTTTAATCTCAAGGGGCTACTGATGTGAACCTCAAGAAGGATACCTTGAAACCTACGAACAAGTTTGGTTTATAATCCCAAGAAAGCCAAAATTAAGTTCAGACACCAAAGAATACCTTGACCTGTCAATTATATAAAATTGAAAACCAAGATAATTCAAAGTTAGAATTAAGGATTATTGACCCAAAGTTTATAATCAAACAAGAACTAAGATTATCAAAAATTGTTAAATCCTACAAGATTAATCACTCTTGAAGAATTTAATTTAGTTTAAGAAAGAACTTAGGAAAAATCTCTCTCGTGTTAATTTTATCAAAAGTAATGTTCCCTTGAAAAAGTATTAAGTTAGCCTTATATATACGGCTGAAACCCTAATTCTCTAAGCTAAAAAGAATAAGATAAAACCCTAAAAACTAATAAAAAATTGGATTGGGCTGCACTCATACCTAAGGCCCAAAAACACATAAATAAGGCCCAAAACACATATTTGACTAAAAATAATTAAATAAAAACATTCAAACTTNNNNNNNNNNNNNNNNNNNNNNNNNNNNNNNNNNNNNNNNNNNNNNNNNNNNNNNNNNNNNNNNNNNNNNNNNNNNNNNNNNNNNNNNNNNNNNNNNNNNNNNNNNGTAAGAGAGTATTCCTTGGTGCATAATGTTTGCGGATGATATTGTTCTAATAGATGAGACGCGAGAAGGAGTCAATAGAAAGCTAGAGTTTTGGAGAAGTACTCTAGAGTTAAAGGGCTTTAAGTTAAGTAGAACGAACACAGAATACATGCATTGCAAGTTCAATGAAGGCCAAACTGGTAATAGGGAAGGAGTTAGTTTGGATGGAGTGGTACTGTCCCAAAGTAATCACTTTAAATATCTCGGCTCAGTTCTTCAAGTAGATGGGGGATGTGAGGAGGATGTTAGCCATAAGATTAAAACCGGATGGTTGAAGTGGAGACGTGTCACGAGAGTTTTATGTGATCGCAAGTTTCCTAATAAGTTGAAAGGAAAATTTTACCGTACAGCCATACGATCGACTATGTTATATGGTAGTGAGTGTTGGGCACTGAAGGAGTCGTATGCATCTAAGATAAGAGTTGCAGAGATGAGAGTATTAAAGAAAGTCCGTAAGTTGAGAGTATTAAAGAAAAGGTAGGAGTGATGTCAATTGAGGATAAGTTGAGAGAAGGGAGATTGAGGTGGTTTGGTCATGTGAAGCGTAGGCATACGGAGGCTCCAGTTAGACAAGTAGAGCACATTAGGTTAGAAGATAGAAAGAAAAAAAGGTGTAGACCTAAATTGACTTTGAGGATAGTAGTACAACATGACCTAGAAGCATTACACATTTCTGAGGATTTAACCCAAAGTCATTTAGAGTGGAGAAAGCGAATCCATATAGCCGATCTCAAATTTTTGGGATAAAGGCTTAGTTGAGTTGAGTATACTATATGCACAAAACATGGAATTAAAGAACTCCAAATAGTTCTTGGAATCAACACACCAACAAGTGAGCAGGGAATGAACATCATGAAAGCTACGTATGGATGAGCAAACTAGTTCATTGCATTATTGGAGAAAAATAACCTTACAATGGAGAAGAGAACTAGGAAAAGAATTTCTAGTATAATCCCAGCATCATGTAACAGCATCTCTGCAGAATCCCACAAAGTAGCACATTGATGTTGAAAGGGATGACTGAGGTTGTGTGGCCCGCCATTATGGACTTCCCCATTATCTGTTAGCGGTGTAATTGAGCATATTGTAACCAATCCCTTCAATTTTCTTGTTATGAGTTGAGAAATTGTAGCAATAGAAAATGATGGCAACTCCTGTTGGATGAAATTGGTATTGGTGCTCTCCATTGGTTGCCATTCTGCCACGGCCTCCATTGTTTTGTAATTATTTAGTTGGGGCTGTGGTTTCAAGGTTCGTCGTTTAGGTAAGGGAACCTGAAATATTGTCGAAGGAGGTGAAAGAGTTATAGCTGGAAATGGTAGTGAAGGAAGGAGTAGGGAAATGGTTGGCTAGGTTATTAGACCTTGTTTGGTTTAAATGAACTACTTCCTGGGAACTCTACTTGTTAGGAAGTAAGTTATCTAGCTTAGAGTACGTAACATACTTATTGGAAAGTGGAGAAAAAATGGCCTTTCACTTCCTAGGAAGTGGACACTTACTCCCAATTAAACAAGTCAATTTTTTGCATTTCTTGGTAACTTGAAGTTCTCTAGCTCACTTACTCCCATCCAAACAAGGCTGATTAGGAGGCTTTGGAGGTGGTCTCAGTAAATATTGTGGCTTGGTGCATGTTGGGTTGCTGTGCAATGGGCTAACTACCTGATTTGTGCCATTGAAACCCTTTGCCCTTAACCCAACGATGAGAAATCTCAGTCCACCTTTAGCACCATCCTTCATCATCCCTACCCATTTCCCAAACAGAGGTCCTTCCAAGTCTCTCACCTGTGTCCCACTGTGAAGATTTTAGTCCTTGCCTCTGTTGAAATTCTTCGTACCTGTCATCTCTGTGAAGTCATGCGACTACAATTGTGTCTTTAAGGTTTCGCGCGATTCTGGCAAGGAGTATCGCATGCAATGGACATTGGAGAGCTCATTTCCTTGTGTCACGCTGGAGACAAGACCATCAACATGCCCAATTACCAAACCCAGCAGAAAGTTCTCACTTCTCAACAAGATTTGTAGTGTATGTGGGATTGTTGTCCCCACCAAATTGTTGCGACAACTTTGCTGTGAACAGGGGCCAAGTAAGTAAAGGATGTCGATGTTGAAGCTCAAGATACCAAAGGAGAGGGGCTCCCTTAAATTTGTCCCAAACAAAATAAATTTTAACTTCTTTGTTTGTCTTGTGGATGGCGAAATATTGCTCCACTTGAGCAATCCAACCTTGTGGATTAGTTCCATCGAAATGAGGCTTTTCATGTGTTTTGGCTGCCAAGGAAGTTTCTTTGAGAAACAGAGTGCGAGAGTTGGATATTATAGGAGGGGCGTCACTGGTAGATGCTAGCTAAAATTTTGGTTAGAGATTTGCGTGAGGGAGGTAATGATAATTTTCCAATTTGTTAAACTGCTGCTCTTGTTTGAATTTCAATGTTGTGAGTTTTTCCCCCATTTTGGTTGTGTTCCTCTCTATTTGAGTGAGTCGGCATTCCATCAATGATTGGGTCATGTTCTCCATTCTAGTATGGCATTCTAATGGCAGTTGGACTAATTGATGGAGTAAAGAAACTCAAAACCTTGAATTAATAACAAATAAGAACAAAGTAAAGAAATGTTAGTAGGAAGAAGAAAAGAATTTTATTCAAAATACCCAAATGGTTCCAGGTGTTTACAAGATAAGACTATCCCTCGCGCACACCAGCAGTAGATCACTGCCCCCGAGCCATGCTAGCTGGAAAATTGTAGTTTCAATTCATGCCTCCTAGTCTACCCTTTCTCTTCCTATATATAATTCTCCTAGCAGTTGCATCACACACATCCAGGAATCACTCTAATGGAATGGAGCTGAGTTGGACAGATCTTCCATAATTTTCTTGCTTCGCTAGAATACATCATCCATATCCCTATCAAATTCCCACTCAATATTTTCCACAATAATTTGTATATCCGTCACATAAAAATAAAGACTCTGCTATCTTCACCCAGTGTTTAAGCCACCCCCATTGAACCAATATTCCTTCTAAAACTCAGAGGAAAAATTGCCCGAGAGTAAATTCCACCCTCGAACTGGAGCTCTAGTATTTACCTGAGCAAAAACTGTTGCTGTTGCTCTCCCTAACTCTTTAGCTTTCTTAATCAAACGCCATAATTATGCCCAAATGTAAGTTATCACCAGCTAACCTACAAAAAGGTGATCTGCTTTACTTTTATGGGCATGTGCTAAAAAGTGTTTGGTTCAACCGGTGTTGCATGGGCAAGTTTCTATTATGCAGGCACATTTGTCACAAGGATAAAAAATCGAGGATCTTGTGCCCATCACCTATTTTGGCTAGGAGTTTACATATCTCAAAATTTGATTTTCAATGATTGAAACTTGGACTTGAATCTTCATTTGATATCAGCACACAAGTCAAGGACAGATAGATACCTTCATTTCTTCTTTAGGGTATTTTTGATGATGTTGTTAGCTATTTTCCTTCCCTCTGTTTTTCCTTTTTCTTTCTTTCAATCTTCTTATTCTTCCTTTCTCTCTATATTTCAAGTGGATTTGGCCTCTTTTTGTTAACCAAGATGTTTGTTTTCTGCAG**CTATCTTTAACCATTGTTTATATAGCTGTGGGTGTTTTTGCAGCTGGTTGGATTG**GTAAGTTTTACATATTTATCATAATTATTTCTGTTATCTTCTCATTTATTGTTCTTCTTTGTTGCCATTGATGCTAACTCGCTTTCTGAAATAG**AGGTATCGTGCTGGATTTTGACTGGGGAGCGTCAGACAGCTGTTATTAGGTCCAACTATGTTCACGTATTACTTAATCAGGACATGAGCTTTTTCGACACCTATGGGAACAATGGGGACATTGTAAGCCAAGTATTGAGTGATGTGTTGCTCATTCAGTCTGCTCTTAGTGAAAAG**GTATCCTAGAGATGTATTATTCTACATGTTCTGAATTTCACAAATATCTTATCTAACTTGTTGAGAGGTCGTTGATGGTGTAG**GTCGGAAACTATATTCATAACATGGCAACATTTATTGGTGGTCTTGTTATTGGATTTATCAACTGCTGGCAGATTGCCCTTATAACATTAGCCACTGGTCCATTCATTGTTGCTGCTGGAGGCATATCAAATATATTTCTTCACAGGCTTGCCGAGAGTATTCAAGATGCATATGCAGAAGCTGCTAGCGTTGCTGAACAG**GTTTATTCTTTTTTCATGTTTTACCTCGATAACTCTACAACCACCCGCGCTAACCTCCCAAAAAAAATAATGAAAAAGGAAAAAGAAAGGGCCCCAGTTCTCATAAAGGAAATGAAAGGATTCTTTTTTGTCAGTATTTTTAATATTTAATTATGACTATCTGTCTCTATCTCGCTACAAAAGTCATTCTTGCCTTGCATATGTTGAATTCTGTCTCAGTTTTTTCTTCTTCATACAACAATATTTTGTGATTAATGTCTTATAAATTGGGTTTAGTTTAGTACATTTCTCTTTCAAAAAACACTACAAGAGACGATCCTACAAACTAAATGGTTTAGTTTAATCCTGATGAATAATGGTGGGGGCATAGTTGTTAAAGGTATGCCTGAGGTGCAATGTGCTTAGTGTTATAAAAGGCGCCAAAAGGCGTTGGGCGGGGTGTGCCCCACTCCTAAGATGCAAGTGCCTCTCTTGCCTAGGTGCTGAGGAGTGCCTCTTTCAATTTTTTTTTTCTTTTTTTCCTTTAGCCTTCTCTCCTATTTCTCCCTAAACCTTGAACCCCAATAAAGTGGAGTTAATTAATCTTTTTTAAAATTTCATTTCATTTCTATAACCTTATTATTTTATGCGTGCAAATAATATGTTTTTTTTATTTATTAAACTAGTTAAGAGTATGATAATTTTATGGTAGAAGTGTGTGTATATGTATAGGCTATGGTGGTGCCTCACTTCAAAAAGGCCCTGTCCTTGCCTCCCACCTCTTGCCTAAGGCCATAGGGTACCCTATCGCCTTAGTGTCGCTCCTTGCCTTAATAACATTGAATGCGCTAGAGGAACTAGACCTAGAACCTCAACTTGCAGAAGTTGGGTGAGGTTCAACAGGCAATGTGTATACATTTTAATGGTGGCCCATGTAATGGAACCTGGGTACTTGGCTTCATAGCATTGGCAACTGTCTTTGCAATGTATATACTTTGTTTCTTACTATTTTCTTTGATGAATTTACTGTAGAATTTATCCAGCATGCTTCTTTTTAGTGAAATGAGCTACAATACATTCATTAATGATTTCTTACATTACGCAG**GCAGTCTCATATATTAGGACCTTGTATGCATTTACAAATGAAACATTGGCCAAGTATTCTTTTTAGTGATATGAGCTACAATACATTCATTAATGATTTCTTACATTACGCAGGCAGTCTCATATATTAGGACCTTGTATGCATTTACAAATGAAACATTGGCCAAGTATTCTTATGCAACCTCATTGCAAGCAACTCTGAGATATGGTATTTTGATTAGTCTTGTGCAAGGACTTGGACTTGGGTTTACATATGGGCTT**GCAATATGTTCTTGTGCCTTACAACTATGGGTAGGAAGGTTCCTGGTTACGCATAAGCAAGCTCATGGTGGTGAAATAATAACAGCATTGTTTGCTGTAATTTTGAGCGGCCTGTAAGTGTCTTGTTCTTCTTACTTGTCGGTGCTGTTATCTTCTGTAACATGTTCTTATCTGCCCTTTTTTGTGTTTCACAG**TGGGCTAAATCAAGCAGCGACAAACTTCTATTCATTTGACCAAGGACGAATTGCTGCTTATAGACTTTATGAGATGATAAGCAGATCTTCCTCCACAGTTAATCAAGATGGAAACACCCTAGTGTTGGTGCAAGGAAATATCGAGTTTCGGAATGTATATTTTAGCTATCTATCTCGTCCTGATATCCCCATCTTGAGTGGATTTTACCTCACTGTTCCTGCTAAAAAGGCTGTGGCACTTGTTGGCAGAAATGGCTCTGGGAAAAGCAGTATTATCCCCCTCATGGAGCGGTTTTATGATCCTAACTTAG**GTATTTCCTTGTGACTCATTATTTTTCACTTAAGGTTTCACTGAATTTCCTTAACAATGGCTTTTAATTCTTTATTATGGTGCAG**GAGAAGTTCTTTTAGATGGAGAGAATATTAAAAACCTGAAGTTGGAATGGCTCAGAAGCCTAATAGGACTAGTCACCCAGGAACCTGCCTTGCTGAGTTTGAGCATAAAAGATAATATTGCATATGGACGAGATGCTACTTTGGATCAGATTGAAGAAGCAGCCAAAATAGCACATGCGCATACATTTATCAGCTCACTTGAGAGGGGCTATGAGACACAG**GTGAAACTAACAAGAATAAATTTTTGCTTGAACCTGTGTCATCTGTTATTTATCCTTTGGCCAATTGATGTAAACCAAGAACACAAATGGCAGAGAAACAAATTCTCAAAACTTTATTAATTATCAATATCAATAATAATCATAATTATAGTCTATCAAAATAAGTAGAGAACATAGGTATTTAGTGGAATTATTAATCCTGCTACTATTGGGATCTGGAATCATAACTAAGAAAATACTAATTACGGATCCAAAAATCAACATAATTTTAAATAAAATAAAATACTATTTCCTAAATAATATAAATGTCCTTAATTGATAATGTTGTAATGTGGAGGATTCCAACATCAGCAAAGCAAAGAAAAAAAATGAAGGGCTTTAAGTACTGGCAAACCAAACCAAAATGGCTTAGGCCATTTTGATGATGGAAGCCACCCCAGCCAGCCGAATTCCAGCAGCAGGGCGGCCCATTTGTCCTTCTATGATAGATTGAGGACCTGCACTTAGCCAAACACAACAACCAGAGAATAGGAAGGAGGAAACAGGGTTGGGAAAGGAGGAATTGTTTTGAATAACAAGGAAATCAGTCTCAGTCTCAACACAAGAGTGAATTTCCCTGGCAGAATGTCTACTGCCCACCCAGCTTCAAAAGCTAGTAATTGCAAATAACTATTCAGCATACCCCTTACATGCTGCTACTACTCTTAATACAGATTTTCCCTCCCATTTACATGTTTGTTAGCCCCTTCACATGCATTCCCGAAATATCCTCCCCATTCTGTTGAGCGGTAGCTGTCATCCCATCTTCTAGAACTTTCTTGACTCTTCTAGAGTAGACCTTCCATTTTCTTTTGGCTCTTCCTCTTCCTTCACTAGGCTCTGGGTGTGTATCATTGCTGCCCTCTGCTGGAACAGCCTTGTCCTCAAGGCTGAAGTCCGGAAATTGGCCTCTGAAGTCACTCACATCAATCCAAGTAGCCTCTTCTATGGTACGCCCGTACCATTGAAACAGAACTTGAGGGACTGAAGTACCACCTTTTGCAAAAGTTCTGTGAGCCAAAATAGATTCGGGAAGAGTGGGAATTGGGTCATCCACAGCCATTTCACGGGGTAAGTCAGTCACTACAGAGTGGTCACCTATCACCTGTTTTAACTGTGAGACATGAAAAAGGGGGCGAAATTTGGCAGTGGCGGCCAGATAGAAACCTAAAATTTCTAATTTTACAATGAAAATTTAAATTGCAATAATATAAGAGTTTAGAATTCCTAATTTTACAATGAAAATTAAATTTCCTTGACTGCATCACCAATCTTTTGAGAATTTAATTCTTGTGCATTACAG**GTGGGTAGAGCTGGTTTAGCATTGACAGAGGAACAGAAAATTAAACTTTCTATTGCTAGAGCGGTGCTGTTGAATCCAACAATTCTTCTGCTTGATGAGGTTACTGGTGGACTTGATTTTGAAGCTGAGAGAACAGTTCAGGAGGCTCTAGATCTCCTCATGTTGGGACGGTCAACCATAATAATTGCTCGACGACTTAGTCTTATTAGGAATGCTGATTACATAGCTGTAATGGAGGAAGGTCAGCTTGTTGAAATGGGTACACATGATGAACTATTAAACCTGGATGGTCTATATGCTGAGCTTCTCAAATGTGAAGAAGCTGCAAAACTTCCAAGGAG**GTATAGCTTGAGAACAAACTGTCTTTATTGCAACAAAATTTCATGTGTGTGCAGCTTCTTTTTTGAAGTGTAGTTTAACTAGTAGTGAAAGTGTTTTCGTATGCTGAAACACATGATTTTGAGACATGTATCTTTCTCCAG**GATGCCAGCTAGAAATTATAGGGAGACTGCTGCTTTCCAAATTGAAACGGATTCTTCTGCCAGTCACAGCTTCCAAGAACCATCGTCTCCTAAAATGATGAAATCACCATCTCTTCAGAGAGTTCCAGGTGTAGTTCGGCCTATGGATGGCACCTTTAACTCGCAAGAATCACCAAAATCTCTGAGTCCACCACCAGAGAAAATGATGGAAAATGGTTGTCGCTTGGATGCAGCTGATAAAGAACCATCAATAAGGAGACAGGATAGTTTTGAAATGCGACTACCAGAGTTACCCAAGATTGATGTCCAGTCTGCAAATCGGCAAACATCAAATGGGTCAGATCCTGAATCACCTGTATCGCCCCTTTTGACTTCTGATCCTAAAAATGAACGTTCCCACTCACAGACTTTTAGTCGGCCTCATAGTCACTCGGATGATATTCCAACTACATGTAAGGATGCAAAGGATACAAAGCATCGGGAAGCACCATCTTTTTGGAGGCTGGCAGAACTTAGCTTTGCAGAGTGGCTTTATGCTGTTTTAGGAAGCATTGGAGCTGCTATCTTTGGTTCTCTTAATCCACTTCTTGCTTATGTTATTGCGCTGATAGTGACCGCTTATTATAGGCCTGAACATCCTCACTTGCGGCAGGATGTAAACAAGTGGTGCTTGATCATTGCCTGCATGGGTGTGGTGACAGTTGTTGCAAATTTTTTGCAGCACTTCTATTTTGGTATTATGGGTGAAAAAATGACTGAACGAATCCGGAGAATGATGTTTTCAG**GTAAATAGTCCTTAATCTTGTAACTAGGTGCTTGAGAATAGAAAATAACAAAATAGAAATTCCTTTATATTGATTTTACCTTGTTACTGCCCAATGCCCATTATGCAACAG**CAATGCTGCGCAATGAAGTTGGTTGGTTTGATAAAGAGGAGAATAGTGCCGACACATTGTCCATGCGGTTGGCTAATGATGCTACATTTGTGCGAGCAGCATTTAGCAACCGGCTTTCAATATTTATACAGGACAGTGCTGCTGTTGTTGTTGCTATAATCATTGGAATGCTGCTTCAATGGCGATTAGCTCTCGTGGCATTGGCAACCTTGCCAATTCTCATGGTTTCTGCAATTGCACAG**GTTTGCATATTTTCTTTCTATTTTTCAACTTGATATCATTTAATTGATTGTTTACTCATTACATTCATATTTCTTGTTTTTGGAATGAACCATATTGTGGATCATTATTCTTTTGATCTGTGTTACTGGCTCCATGCCAATGAAATAGATCATATGGTTGCTTATTCTGTAAATTTTGGTTCCAGGAGTTTTAATGTCATTGTCATCAGTTTTCAAGTCATTTTCCTCTAGTTCTTTTCTTTAAACAAGACATATCTCTTTGCTGCTTTTTACAAGATGGCAAATTCAGTTTTGCAATTATTTTCTTCTAGATGGTTCATTCCATTTTCTCCAGATGTTGATTCTTTTTAGTTTATTATTTTAATTTTGAATTTGAATAGACAAACAGGAATTTTCTGATAGGTCCTTGATAACAAAGTGTGAACTTGAACTTCTGAAACACCATCTTGGTCGCTTGGTGTGACCATGAAGTTCACTTTTTAGTGTGTTAAGGAATGAAGAAGAGGAAGCAAAGGAGTTGCCAGTAAGGTTGTTATTTTATTATTTTAATTTTGAATTTGAATAGACAAACAGGAATTTTCTGATAGGTCCTTGATAACAAAGTGTGAACTTGAACTTCTGAAACACCATCTTGGTTGCTTGGTGTGACCATGAAGTTCACTTTTTAGTGTGTTAAGGAATGAAGAAGAGGAAGCAAAGGAGTTGCCAGTAAGGTTGTTTGACAGGTCAGTTTGTCCATTGTAACTTGAGTGATTAGTGAGCTTGAGTTTATGAGGTGCGAGCTGGAATTATAATGCATTGTGATACTTCAAATGAAAAGTCAGAAACTCCCGAACTCTGTCTTAATTTTCTCTCTGGATTCTTCTTCTTCTTCTCTTTCTTTTCCAACTGCTCATTTTCTTTTCACTTTTTCCCCTCACTCTCTATTCCATTCTCTCCAAAGGTTTTTCTCTGATCTGAGCTTTACTCCAACCTAAATGAGTGGAGTTCCTTTACAGGATATCCCTAGATGTTATTCCTATCACAAAGTCGTACTTGTCATAACACTATGAGCAAATGGTGCGGATCATCCTATTTCTTATAATTGTCATGCTTTTCCCCCAGTTATGCATACACTGGAGTGAACATGTCATCCAAGCAAAATGATGTCGAATGCTTTTCAGATTGTTATGTTCTGTGTGATGTTGAATGCTATTGCAACCTTTCAGATTGTTATGTTCTTTGTCTTAGTGTTTCGTTTCCTTGCTTATGTTTTCAAGACTGATGAGTTCCTTTTTGCATGATCTGTTGGTCTGTGCCATCCTTGCAACAGATATTGTGATCATGCCAGTCTAACTGCCTTCATGTTTCTTGGATGTATAAGTTATTCAGAAACTAAACTTCAATTCTATCTGCATTTTTGGTTTTTGACAACTTGATAATGACACATTCTAATATCTTATTTTTCTGCTCAAATTTTGAATGGCTTCCAACTGAAATATTGCAG**AAATTGTGGCTTGCTGGATTTTCAAGGGGCATCCAGGAGATGCACAGGAAGGCATCATTGGTCCTTGAGGATGCTGTTAGAAACATTTACACTGTTGTAGCATTCTGTGCTGGTAACAAAGTAATGGAGCTCTACAGATTGCAACTGAAGAAAATATTCAGAGAGAGTTTTTTACACGGAATGGCTATTGGTTTTGCATTTGGCTTTTCACAGTTTCTTCTATTTGCCTGTAATGCACTTCTTCTCTGGTACACTGCTTATTCTGTAAAGAATCATTATATGGATCTGCCTACAGCCATCAAGGAGTACATGGTTTTCTCATTTGCAACATTTGCACTAGTTGAGCCTTTTGGATTGGCTCCATACATTCTTAAACGGCGGAAATCTCTCATTTCAGTATTTGAAATTATAGATCGAGTGCCCAAAATTGATCCAGATGATAACTCAGCACTGAAACCACCTAATGTCTATGGGAGCATCGAGTTGAAAAATATCGATTTCTGTTATCCTACTCGCCCAGAAGTGTTGGTATTGAGCAATTTCAGTCTCAAAGTTAATGGTGGTCAAACTGTAGCTGTGGTAGGAGTTTCAGGTTCTGGAAAGAGCACTATAATCTCTTTAATTGAGAGATTTTATGATCCAGTTGCTGGTCAGGTTCTGCTTGATGGGCGAGATTTGAAACTTTATAATTTGAGATGGTTAAGAAGCCACCTTGGTGTGGTTCAGCAGGAACCAATTATCTTCTCAACAACCATAAAAGAAAACATTATATATGCTAGACACAACGCTAGCGAAGCTGAGATGAAAGAGGCTGCAAGAATAGCAAATGCTCACCATTTCATCAGCAGCCTGCCTCATGGTTATGACACACATGTGGGAATGAGGGGTGTAGACCTGACACCAGGACAGAAGCAGAGAATTGCAATTGCTCGAGTGGTGCTGAAGAATGCACCCATCTTGTTATTGGATGAAGCTAGCTCCTCCATTGAATCTGAATCTAGTAGAGTGGTGCAAGAGGCACTAGATACACTGATTATGGGAAACAAAACAACCATTCTGATAGCACATAGGGCTGCAATGATGAGGCATGTTGACAATATCGTGGTTCTAAATGGAGGACGAATAGTTGAGGAAGGGACCCATGATTCTTTGATGGTGAAGAACGGCTTGTATGTCAGGCTGATGCAACCCCACTTTGGAAAGGGTTTACGACAGCATCGACTTGTTTAGGTTGGTTGACATTTGGGCATAGTTCTTTTGTATAGCTCTTATTGGCCATTTTTATATTGCTGGAACTTGTTGGCAATATGGAAGAATACATTCCGGAGTGGGGAAGTACAAACAAGAATTCTGAGGCTTGTAACAATTTTTGCATGGTTGGATGGGCGGGTTGCAGTGGTGACAGTGACAGTTATAGTGTTCGAATGTAGGGTTTAGGAATAGGGATTTTGGAGCTTGGTTGGCCATTGAGGACATGCTCTCGGGGCTAGATGCGTATTCTTTTTGGAGGCAAGGATTAGGTTGTTGCCTTGTCTCCTTGGTATATTGATTTGTTCATTAAAAAAAAATTGGGTTGGGGTGGAGATAATTCTGG**

>HbABCB25 scaffold0069(27075-64634)

**GTCCCATTTGAACTGAATCACTTAAATAGTAAAATTTGTGTACTAGCCGGAGGCGACACCATTTGTCCAAAGCAGGCAGCGAAGCTAGTCCCCCGGGCTCAATCTCAGCCGCACCAACACCACGGACAGCCGCCCATATCGATTTGGAATTGGATAATAGTTGCTATTCTATTGTTTATCTGCATTCAAGAACGAAGAATGTTGGGAGCATCAAAGTGGTTTAGGTTTAGTACGCGGGAGCTAATCTTACGCAATGGCTCTTGCAAGAAACCAGTTCTCTTAGCCCAGAACAATATTCTTCTGCGTGGGAGCATAAGCAGCAGCAGTAGTGTTATCAGTGGTTCCTACTCTGCGTACCTTCCATGGAAGAATCTCAGAAGCAGAACAAGCTCCGCTGGACCTCTCAATCTCAAAAATTTCCTATCCGATTCTGGGTTTTCATCTGCCCCTTCCAGGCCACCG**GTTTGTCTAGATTTCGTATTGTTCTTTCCGTTTAATTGATTTTGCCCAATTTGAATCTGTACTATAGAATTTTCTTTTAACAG**AGTGCAATGCTGAATGGGCGCGTATTGTTCTCGACCTCCGCTCCTAGTAATCCTAATGCTAATCAGAACCATGGAGCTAAATCATCGATTGCTACTACCAAATCAGAAGAGAAACATGTTGCAGACATGAAAATTCTTCGTACCCTTGCTAGTTACTTGTGGATGAAAGATAACTTCGAATTTCGCTTAAGGGTCATCACTGCTCTTGCCTTTCTAGTGGGTGCCAAGGTCTTGAATGTTCAGGTCCCTTTCCTGTTTAAGCTCGCTGTTGATTGGTTGAGCACTGCTACTGGCAATGCTACTGCCCTTGCTTCCTTCACTACTGCTAATTCTACTCTCATTGCTCTCTTTGCAACCCCTGCATCTGTTCTCATTGGTTATGGCATTGCCCGCTCTGGGGCTTCTGCTTTCAATG**GTATATTTTTTTTAATGTAAACTTGCTAATTTCAAGTTAAAGTTGGCATCTTCCATTCATATTTTGTTGAAATTCTGCTCTTGCAG**AACTGAGGACTGCCGTGTTCTCTAAGGTTGCTCTGCGAACCATCCGTCAAGTTTCTAGAAAG**GTAAATTATTCATTGGTTCTAATGTTTCGTTTTTTTTTTTGGCTTATGTCGGGGTTCATCCATGCTTCATATGCTTAAGATGTTTGTTCTTTTTCTGATGTGATGTTATGTTATGGTCCCTTCTATCTTACTTACATCATATGATTGAGTTTTCACTGCGCAG**GTGTTTTCACATTTGCATGACTTGGACCTCCGTTTTCATCTCAG**GTATTTGTGTACAATGATATAAGTTGCTTTGGTTAAATATAAGGTGCTTTTGGCAATTGACTTACATTTCTTCTGTTTGCACTTGGTCACTCTTTTTCCAG**TCGAGAAACTGGTGGATTAAATAGAATAATTGATCGTGGCAGCCGAGCAATCAATTTTATTCTCTCTTCAATGGTGTTCAATGTTGTACCAACTGTCTTGGAG**GTATGAATGCATCTTTTTCCAGTCGAGAAACTGGTGGATTAAATAGAATAATTGATCGTGGCAGCCGAGCAATCAATTTTATTCTCTCTTCAATGGTGTTCAATGTTGTACCAACTGTCTTGGAGGTATGAATGCCCCTCATCTTTTTTACCAAGGCCTTTAGTTCATTTTGCTTTCTGATAGTTATAGTAGGCATGACAATGTTCTGGGATTTGGACGATCAATATCTGTCCATGCTGGACCCTTCATTTCCTTGGTCATTACATTAACATATCAAAACTGCATGTCTTTATACAATATACATGCAATTTCTTGCTTCGAAACATTTTTTAATGGATAACAATGATATAAGTTGCTTTGGTTAAATATAAGGTGCTTTTGGCAATTGACTTACATTTCTTCTGTTTGCACTTGGTCACTCTTTTTCCAGTCGAGAAACTGGTGGATTAAATAGAATAATTGATCGTGGCAGCCGAGCAATCAATTTTATTCTCTCTTATTCTAATATGATCCTTTCAG**ATATCTATGGTATCAGGCATTTTGGCATACAAATTTGGAGCTCCTTTTGCATGGATCACGTCACTTTCAGTTGCTGCATATGTTGCTTTCACATTGTCTGTGACACAG**GTAACATTTTCTGCCGATTTTTTGTGACCACATTTGTGTAGGATCATAATAATAATTAGGTATGGGTGCTCTTTTGTTTTCTTTCATAACTTTGTGCTTTAGTGCTTCACAACTATGGTCTGTTAGGATCATCCAAATATGAAATGGGAATACAAATGACATAAAGGTTTAAATTACATATAGTGTAATGATAGTTTTATAAATTGTACTCAAAACCAGATTTTAATTTTTGTCTAAATTAAATAGTGTACAATGTAGAAATATCAGCAAAAAATTTTGGATTCCTTGTTTTCCTAACTATTGACTGAAGTCCCATTTTAGTATTTGCATTCAATTTCTAGAATTAACCAAACTGGCTTCAAATACTCATGTGTCATTTCCTTGGGTTAAATGGATTTAAATTGCGGTTGGAGCTGTGATAATGTTCACAATTGTGTGGAACGGAAATGGGGCTGTTGTGGCTGTAACATGACTGTAATGGGAATGGCCTATCACTGTGCAATAATGTTTACAAAATGCTATAAACTAACAGATATCAATAGAACCAACTAAATTAAGAATCCAACTTGCTACTGAGCATAAATACATAAAAAATAAACTCATGGAATCCATGTTTTATATTTATTAATTATTATGAACAACAAACTTAGTTAACTAGCACAAAATAGAGTAGAGGATGGAGAACTTACCTTATCGTCACCAAAAGGAGTGTCTAAGAGGGTATTGACTCTCTTTGTACCAAGTAAAATGTAAAATTAATTTCAAGTGGATTGAAGAAATGCACAAAACAGTCTAAGAGGGTATTGACTCTCTTTGTACCAAGTAAAATGTAAAATTAATTTCAAGTGGATTGAAGAAATGCACAAAACGAAAGAAAGGTCGCTGAAAAGAAGGTGGGGTGGGGGGTGGGTTTGGCAGTCTAAAAGTGTGGTAACCCTTACTTAAAGGGTAATGAGTAATGACCAAATAACAACCATTACTTATTACGTAATGCTGGTAATGGTTGCTACTTGAAAAGAATATATATGTTAGGTAATAATGACCAAATAACAACCATTACTTATTACGTAATGCTGGTAATGGTTGCTACTTGAAAAGAATATATATGTTAGGTAACAAGAGTAATGGTAAGGGGCCCTCCAAAAACCTTTATTGTCGTTACATCTGTTATTTAAATCCATGTAATGTAAAAGCCACCCATGCAATTTTTTTTGATATGTAAAAGCCACCATGCAATTTTTTTTGATATAGTTTTTAGGGGGTTGGTGATGAAGGATGGATTAGAATGGGCTTCCTTCATTTTCTTTTGCCTTCATTTCTTAAGCTAGTGAAACTTTATACGTTGATAACCATTGTGGCAACCCTTCCTTTTCATTTCTAG**TGGCGGACAAAGTTTAGGAAGGCCATGAATAAAGCGGACAATGATGCCAATACAAGAGCAATAGATTCTCTAATCAATTATGAG**GTGAGATTGTAATGTTTCATTTCTAATCTCTTTTTGGGTTATCTAGTTTTCATTTCCTTTGACTTCAATTTTTTTTTCTCCATTTTTTGGGTTATGTATTTTTCAGTTCCTTTTGAATATTTTTTAATGTTTTTTTTTTGTTATCCATTTTCAATTCCATTTCCTAG**ACTGTCAAATATTTCAACAACGAAGCTTTTGAAGCTGACAAATATGACGAGTTCTTAAAGA**GTAAGTAATTTAAGCATTCTAGTTTTTCCCCTTCTCTCCATAGATGTTAAACATGAACTGAACAAACAAATGGTTAAACTGAAAACTGATACAGGTTTGTCAGATTAACTTGATCATTCACAAAAAGGTGAAACTCATAATTTGATATCTGTGCCCACATTGGAAAGGAACTAAGGAAGGCTTGTAAATTCTTTGCAAAAGATTTTCTATTCTACCAACTTAACCTTAAATTAGGTTGAAACAAATTGGGGACTTTCAAAAACCAAAATAATTTTATGTAGATTTGCAATTACTTGCGTATTATTTATAGACAGGATTTTCCATAATGTTCATGGTTTTATATGCTATTATTGTCTTTGACAACATAGATGCACCCATCCAAGTATTCTTAGGTGAAAGCTTTCTATGCAAAATGTTTCATTCATTTCTTTTCAATCTTTTTTTTTTTAAATTTTTTTATGCTAATTATATTGTAATTATTGTTTTATTTCACTTCAACTTGCAAAATTAGTTTCTTGCTATCATTTCACGGGCAAGTGTCTATTTCCAACCAGTAATCTCTCTATAAAAGACTTAATTTTTTGAGTGCTTTTTTTTTTTTTGGGTGGATATTTTGCAGCCAAACAACAAGTTGTTTAACTCATTGATTGTCCATTGTGCTGCAATGTACTGTGGCAGACTCAAAATTTTCATGTTATGATACATGCGCACACACACAAAATTTTCTGCTAATTTATACTGTTTTAGTTTTACTTCTTTCTTCTCACTTGATAAGGACTATTTTTCAG**GGTATGAAGGTGCAGCGTTAAAAACACAACGAAGTCTTGCATTTCTTAATTTTGGCCAAAATGTGATATTTAGCTCAGCTTTATCAATAGCTATGGTTTTATGTTCCCATGGGATCATGAATGGCCAAATGACAGTTGGTGATTTG**GTAAAACTTCTTCATACAATCACTTCTTGTAGAGCTTTACACTCTGGTAAATTGGTTACTTGATGATAATACAAGTTCTTATTTATAAATGTCAAAATGCAG**GTTATGGTGAATGGGCTACTCTTCCAGCTATCCCTCCCCCTCAATTTCCTCGGCAGTGTTTATCGTGAGACCATACAGAGTCTTGTTGACATGAAGTCAATGTTTCAATTACTTGAG**GTAGTTTTCAAGGAACTAATTACAATTTTCTTTTAGTGCAGGAGTTGACAAATCTTTTGTTTCCTTTTGTCTTGTTTTTCTTAAACATCAAAGAATAAGAAGCACAAAAAATACATTAAGATCATTGCAACTTGCAACAAGTGATTGGTTTATTGATCTAAGGGATTTACAATTTTCTGTGTGTATTTTCTTGATTTACAATTTTCTGTGTGTATTTTCTTTGGCACTTGTTACATAATTTCTTTATAATGATTTATCCAG**GAGAGAGCTGATATTAGAGATAAAGATGTTGCAAAGCCTCTTAAGTTAAATCGGGGTAGCATACAGTTTGAGAACGTGCACTTCAG**GTAAAATGGTGCTCTTAGTTATTAAAATATTTTGGAAATCTAATAGTTTCACAAATCCATATACGAAGCTAAGGTCTTACTTGCAGGCCATTATCATAATTATTTTGTATCATCTGTTTAGCACTTGCATTTAGCTTTCCCAGAATCAATAACTGAAATTACTATTTGTGCATCTTCTAATATTGATTTTCAATTTTTCACTCTAGTTGTATTCTTAGGTTCTACTTGAAGTAATTATATTTCTAATATAGTCATATTAGTATGCGATCCAGTGCTAATCTATTCCTTTACTTGGACAAGAAAATTGTGAATAGTTTAGATACTGTGATCAGCTTAGATTTGACCCCAATCTGGTTTAGCACTATCTTTCCTAGTACTACCATAGTGCATGGAATATGCCAAGATATCAAATATTTGGAGTTTCTTTTTGTATCTTATATGGATGATTCGATTTGTAAGGTCCATGATTGGGAATTGTTTGATCTTCTTTCTCAAAGGAAGGGGTGAAACATATAAAAACAAAAGAATTCGGCACTCGGGAAGACTAACCAGGACATTGAAGAATGATTGACTACTTTATCCCTTACAGAGTACCAAAATCCTTCATAAACATAGCCCAATGGACGGGTTCGGATATTGGGTGTAGGACCACCCTTTCCGTTCGAGATTACTAGTTACAACTTATTGCAACTAAATCGGCCTCCACTTTATTATATAATCAATCCCTTTTAATATTTTGTTATTGATCCTTTATTTCATGTAGTTAATGATAGTTGTATCTTCACACATGAAAGCACATAAACGAGTAAACCATTCATTCATGGTCAAACTGAATTAAAGAGTCCTAATCGTGCTACGTTTCTACTTTTAGGGTTAGTCTCACTTGCAAGTAGCCTTATTAGCATGTTCTTAGGTTAATTGCACACAAAGTCGATTAGGATTTAATTCACATTGGAGTTCTAAATGAAACATACATTTAAACAAGATAGAGATCTAACCTAAGCATGTTTCTAACCTAAATAGATATTCAAGTAAAATAGAAGTTTAGCCTAAATAAATATTAAAGCAACATAGGAGTTGAACCTCAGCATGCATCTAAACAAAATAGAAATCTAAAGAATGAGGGGAGAAAAAAAGAGTTACAACCCTTTAAAACACTTAAACCTTCAACTAAAGAAAGAGGGAGGAGAATAAAATTACAACCAAAATAAAATACAAAGAAAATGCTAACTAATCCATTTGGCTTTCTTAGGGTTTCAAAACCCTAATTTCATTTGAAGGGGGCCGAGGCCCTTGTTTGGTCTCCTTTGGGCCAATGCCTCCATATGCCATCATCACTTCTCTTCAAACCTCCAAGGAACGCACAAACTTCATAGAATTGCCAAAGGGACACTTAATTGACACTTATACAAAGATTAGTACACAAAAGTAACAAATTATATTAAACCGGTAACTGCTCAATTCAAGATGTATAAAACATGCTTAAAACGACCAAAATATATAAAATTAATCTAAACCAATAGATTTAGATAAAAAATACACAAAGGAACATAGAATAGAACAAAGCAATGACCCTTTGAGTCAAGAATGCTTTGACCTCCACACCAAGGCTAGGGTTTTCGGTTTCTTTGATTCCTAATGTTTCTAATGCACTTTCTACTACTTTAAAATAAAGTTCAGTACCTTAAAAGTCAAACGTGCTATTGAAATGCATTAAATTAAAATGAATTAACATAGATATGAACATGAAATCAATTTTGAAGGAAACAATGTAAGGTGAATCAAAATCATGCATATGGACCCTAAATTGATTCCCATTAACATGGACTTGAAATTGAATGCCTAAACTAATATGAACATGAAGTCAATCTTTTAAACTAATCATGAGCATGAAAAATATCTCCTTTTTTTATATTTTTTTAACCCATAACCTTTAAATCTATGTTGAAAGAGAGATACAAATCCCTCGCTAAATAATATATGTGCATACTTGGTGAAATTCAAGTGCCCTTGAAAACATATCTTTTCGATATGAGGACTTTTATGAACACAAAATAGTTGCTTATGCGAACCAAAATCACTATTTAATAGGACTTTTGGTGATTTTAACCCCTTTAAAACCCTGGAAATCTGGAACTTGATTTTATCCTCTTAACTGTAGCTTGGTGATTTAGAACATTTAGGGACTAAAGCTATGATTCTAATTGATTAGAATCAGTTTTTGAATCCCTAGAACAAGAATTCAACAAGCTATGGAGCTTGCAACTTCAATTCCTTCTACCTCAAAAGCCCTAAAAGTGGAACTAGCACTTCCCAAAGAGCCCTAGCTCTCGGTTTGGTTCAACCCTTTGACTTCAATTTGTTTGTCCCTTTGCTTTGCGTTTTCAGTTAGCTTCTCTAATTTGGTGGCTTCCCCCTTTTGAGATGTGTTATGATATATTTATAGGCTCCAAGACCTTAAGGATAAACCTTAGGAGTTCGATTAGGTTTAGCAAACCCTATTTCAATTAGTAAGCTTCCCCTTGTAATTTCCAGCCACCAAGTCCCTTTCCCTTTAGATATGTTTTAGATATGTTTAAATACCTTAAGTTTATCCCTAGCCAACTAGGATTGACCCTAATCAACGTTAGGATAAGAAAAAGGACTTCAATTTTTTTTCCCAAAATCCTTTACTTTGGGATCAAGTAACCCTTGTCGTTATCCTTTTCTTTGGGACCAAGCAACCCCTAATTGTCTCCCAATTCAAAAATTTTATCCTTGTGAGAGCTTTTGATAAGACCAAAGCAAGCCTCAAGGATGGCCACGGGCCGAAATTTATCAAGGCCAAACCCTAATGGGCACACTTGTTAGTTGTTACCCTAATGAGACCATTAACCATCCTTTTAGCCCATTAGTCTTATTTCTCTATCTAGATAAGACTACAAACCCTAATGGATAAGTAATGGACTAGCATGGACCGAAATCTAGGGATTTGAGGGCCTAGTATGGTCCTAAGCTTGGCCCAATTACAAACCCTAGTAGGCCTTTTGATTCCTTTTGATTCTTCCAATCCTAACTTTCGTTTTTAAATCACTAAAACCCCTTTTGAATTAGTCATTTGCTTTGTAAGAGCTACATTTCATGTCCATACGTGTCAAATGCCACAGGTTTCATCATTGAGCTTCCATGCACACTCAAACTGTGCATATGCACATTGGTGCAAAAATAGGTGTCTACAGGCATAGGAGAATTTGGTTCATGTGATATAGTGGAAACTGAAATCATTTGGTTTTGCTGATAGCTGACCTCTTACATTTTTTGGTACTTGTCCCTATTTGAAACTTGGAGAAATTTTTTAGCAAAGTTACTGGGGTTGCATGGAAGGATCTAATTGCAGCAAACCTTTGGCTTTTGTATACTCTCTTTGTTGAAGGACTGAAGAAAAAGAAAACTTATATTTTACCATATTATTTTGAAAAATATGCTCACTCAAAATTTGATATTTAGGGTCGAAGTCATGTTAATAAGGGCCATAGTGATAGCGATGGAGGACATATTCCAATAAATCTGTCCGACCTGATTGTGATGAGCTAGTGCTTAAAAACTAAGTTTAAGGGCATAGGAATTGGGATGAGAATTTCATTTCTGTATTTACTACAAAGGAGTTTAAGGATAAAAGAAAATTGCTTGTTGGCTAAAACTGAAAAATCCGGTCACAGCAGCATCAAATTTAGCATTTTCGATGTTTGTTTTGATTGTTTATGTGGTGGAACAAATAGATAATATGCATGGGAGTTATGGCTTCAGGGTGCAAAGGAGGCATTTAATTTGTTAAAATAAATAAAGGTACCAAAAGTTTGTGGATTGGGGTTGTAATTAATTAACTAGAGAGAAAAACTTTCTGTTCACCGAGTAATAGTTGACATAAACGTGAATACTGTGGGCAGCCTTGGGAGATAGAAAAGAAGAAAAGGAAGAATAAAAGAGGAAAAAAGATCATGTTTTTCAACTAGCAATTCTTTCACAAACTGAAAATACTTATAATAACCAATTTTAAGAGATTGTGATTATTAAATTTATGGCTACTAACAAAAAAAGTGCCCATTTTTAATAGTGATAACTTCTTTTACAATTTTCAGTAATGGTCTTAATTATTAGTGGGCGGTAGCACCTCTAATCATGTTCCTTTGATGCAGCTCCTAATTGGAAGGTGATTTGAACTTGACTAATTTTTTATTTTTTATGCTAAGATTTCTAATTACATTGAAATATCTTAGACAAAATTAAATTTAATTTGAAAAATAAGGGAAAAGAAATGCCAATATTAAATTCTAATTATTTAAATATATAAAATTATTTTAAAAAGCCTCATCATTTTTAGTTTTTTCCTAGCCCATTATCAATGTCACTTGGTTTAAATGGAGAAACTAAAGGTTGGGTTCAAGTGTTAGCAATATTAAATTTAACCCATTAATAATTAAAAAAAGTGTAAACCAGTCTTTTAAGTTTATAGACTATTGGACTCTCCGCAATGAATTAATGTATGGTTACTATTTGAAACCTCCATATTTTTAAGACTCTTGATGTAATATGAAACAAAGGAATGTAAAACCTATCAAAAGTCTATTCTAGGAAAGCAAAAGATTAGCAAATAGCATTGCATAATAAAGTATGAAAACACAAGTAAATGCAGGATAACTGCAAATTAAAACCTGAACAGCTGCCACAAATGGCTTATCTTTGACCAAAATGCTTATTGGCATAGGGTTAATAGATTGAACTTTAAGTTTCAAATTGGATAATAGATACTGGGGTTACTAATTATTCAATGATATCAAATCAATGAATGTTTTCTTAAAATCCTAGGAGTTCAAGGTATGACAATTCTAAATTTTCCTAAATTTATTTCACTAGTAATCTTTTAATATTTTAACATATTTTAACATGTTCTATTAGCTTAGGTTCCAAAAAATTCTTTGCTTTCTATTACAGGTTGCATTGGGAACTAGGATTATTTAAAGTCTATAAAAGTGGCCGTGCATTTTCTTTCAAGAAGGAATGAGGTTTTGAATCTTTTAGGAGTTTTTGTTTATGCAATTCTAACCCTAGATGTGATCTCAACATGATGTTAGTGTAGTGTTAAAAGGCTATATGCAAGATTCCTGGCTACAATATTTAGAATTTTAGATGCCATTTGTAACATCATGAGCTGATCAACTAAAAATGTGGGCTAGAGAAGTTTTAGGGATCATGGAGTCTAGTGAGTTTACCAAGTATCTCTTCACTGAATTACTGGTGTGAACATGTGGGATATGGTGAAATAGAAAGAAACGTAAAACCCAAAAATGCTGTTCACAGTGCTTGTCAGATTGTGCTCACTTCTCAAAACAATATTATTCTATCAATTAGTTTTCGATTGGCAGCCTTTGGATCTAAGAAAGATCTCCAGAAGATCTTCTTCTTCATCTTCTTCTTCTTCTTTTTATTTATTTATTTATTTATTTTTTTCCTTTCCTGAAGAACCTCTAGAAGTATGTTTTTAATCATTGTTATTTTTTAAAATATGTTGCCAACTTTCAGTATCATACCATTATCGATCAGTGTACTGTCTAATAAGTTGGTTGGTTATTAATTGCTCATGGGCTGTAGTTGGAACTTGCAACAG**TTACTTGGTGGAAAGAAAGATTCTTGATGGCATATCTTTTGTTGTACCAGCTGGAAAAAGTGTGGCTATTGTTGGGACAAGTGGCAGTG**GTAAGGCATTGGTTTTTCAGAGTTCTGCATCATTTTTTTATTTCATCAGAACAATTATCTTGAAAGAAAATTTTATCTATTGGTTTTTATTCATATGCCTTCCAAGCCACAGTAAAAGGATATTTGCTGGAGTTGCCTGAATGTTTACATACCAATACCTAAATTAGGTTTACCATGTAAACAGTTTCTTGTTCTTCCGCACCTAATTTATGAAATACTATTAGGTGATGACGTGCAGTTAATTTGTCTATGATAAATTTTGGAGAGAGTGTTAGACAAATTACACTCTAATTCATTTTAATATGGGCTTGTACAAATGATTGAGTTACTTTACACGTCAAAAACGACTAGCCAATTCAATATTGCAATCCAACTATTTATATTTTGCTATTTCATTTCATAGATTTTCGGTGTGGGATGTGTAACACTCCCCTCTCAAGTGCAACCACATGGTTGTTTCTTGATAATTAATTAGTATTAACTCAATACACACTTAATTATACAGTCTTATGGGCAGCTGAAACCCATAATCCAAACCGGATTTTGATACCATTTTAAATTTACAATACACACTTAATTATACAGTCTTATGGGCAGCTGAAACCCATAATCCAAACCGGATTTTGATACCATTTTAAATTTTGGAGAGAGTGCTAGACATATTATACATTAATTCATGTTAATACGGGCTTGTGCAAATGATTGAGCTGCTTTACACATTAAAATGACTAGCCAATTCAATGTTGCCCCCAAACCATTTATATTTTGCAATTTCATTTGATATATTTCTGAGGTGGGATTCCCAACAGTCTAAATGGTCGGCAAGCTATTAGACTACCCTTTTCTGCTGGCTGGTGTGACAACATTTTGCTAATAAAGAGCAAAGACAAGTTAATATGTTCTTTCCAGTTTCCCTTTCCTGTGAAATCAGGCAATTGTTATTATGATGCCTAACAAAGTGAAATTTGTTTGTATGCACTTGTGTTAAATGATACTCTTGCATTGCGTTAAGGAATGCCTGTTCATTAGTTACCTTGCTGCACTATTAATTCATATAAATTGATATATGCTTGCAG**GCAAGTCAACTATTCTTAGATTGCTCTTCAGGTTTTTTGACACTGATTCTGGAAGT**GTAAGAACTAATACATTCATCATTAATACCTTAGGTTTCCAATTGATTGCAGCACTTCTTTTCTGTTCTTTTACTCTTAAATAATTTCTAATGGAATCTTATATATTGAAAAGAAAATTTCCTTGGTTTTTTTTTTATCACTCCCTTGCAACCAGTCTGGACTTTTATATTCCAATTTGGTCTGTTCCCAACTTCTGCCAAGCTTAGGAAGTGCTGTTTGCTGCTTGACAG**ATAAAAATTGATGGTCAAGATATACGAGATGTTACACTGGAGAGTCTGAGAAGGTCTATTGGTGTTGTCCCACAAGACACA**GTAAGTTCAGTTGAGCAACTCAGTTGTGATGTTGATGAGCTGAGCTGAGCTACTCTTTACTTAATGTGCAG**GTACTATTCAATGATACGATATTTCACAACATTCATTATGGTCGTCTTTCAACAACAGAAGAGGAA**GTATGTCTTTTGCCTTTTTACTCTGCTAAATATGAAATGTTGGTTACTGTTGATTGTATTTTGCTTTGTACTTATTCATATTTAG**GTGTATGATGCTGCTCGACGTGCTGCAATCCATGACACTATATTGAATTTCCCTGAGAAATATTCTACTGTGGTGGGGGAGCGAGGGCTCAAG**GTGAGAGGTCTACTGATTTACCAAAAATATGCATTTATTAGGTATGGTTGTAGGACACCTGTCTCAAGTCCTTGGCTGAGGCAATGTTCTATGTTTAAAATTTTTGCATGCCTGTCAAAATGATCACAGTAAGAAATGGATTAACTTTAACTGCTAATCATCTGTTCATACACCACAAGGAGAAAAGCTACTTTGGCTCAGACTGCAAAATAACATTGTGTTTTCCCTTAATGCGTACAAGTTGAAGGTGTAATCAGGCCTAACTTTCTAAAATTCTCATTCATTCTAACCTGCCTTATCTTGAGAATAAGATGGCCTAGGTTTCCATTTGTTGTGGTTGACATGTTCAGTTTTTGGCCATTGTCATTCCATTGCAGTTGCATTTCAGCTCATTTGGACTGTACACAATTCATGGACTGAAATTGTTGCTTAGAATGGTGGTTTTGCAATTGCAAGGGGCATTAACAACCACACAACCTGGAAAGTTCTCAAATGGATTATTCCTTGATGTGTGTTGGACTGCACTGCATACATTTATCATGGACTGAACTTTTGGAGTAAGTTACATGTATGATAACCAGACTTTGCACTGAATCTCATCATTGTCACTTCAATTGACTCATAAAAATCACTGAACTTATCATGTAGTTGCATTAAGGTCACGTGACCACTTTCTGATAAAAATATTGTTGGAACATTGACATGGCAATCAAACTTCTTCTTCATCTTCTTCTTTTTATTTTTGTTTATTTTTTATTTTTAATAATTCCATTTCATCCAAATGTTGGCCTTTTAAAGTTTAAATTAAATTCTCTCTCAAGTTTATTGCAAGTTTTGAAGGAACATATTTTGAGTCAAATTGGATAAAAGCAACAAATTTGACTGCCCCATCAGCATTTTGGCAACATTCTTGCAACATTTTGGCCTGAATGTGGTCTGAGTGATCTAATTGCAACGACATAAGTTCAATGAATTTTAAGAGTCAAGTTGAAGTAATAATAATGAGATTTAGTGCAAAGTCAAGTTTTTATGAATGTAATTCACCCATAAAATAAATATCATATAAACTCAACTAAGCTTTAATTCCAAATTAATTTGGACTCGTGAACTATTGTATGGCAAATTATTAATTTTTGACTGGTTCTCGTTAATTGATGGAAGCATAGTAACTTTATGTCTTCGTTGTTGGTAGAATGACTCCTACAAATTGTGATGACAGATATTAAAACACAAATGATGAGAATGTTGCAGGGTAGACTATTATATGTGTATGATTTGGGAATCTGTGTGGTAAATACAATGCACAATAAACAAAAGATATTTTGTAATAAATATTCCAATTAATAGGGCAAATATTTGCATGTCAGTGGACTCTTGAGCTTTGAATAATCAAGAAACTTTTCTTTTCCAG**TTAAGCGGTGGTGAGAAGCAACGTGTTGCGCTGGCACGTGCATTTTTGAAAGCCCCTCCTATTCT**GTAAGAACTTTATTCTTCTTCTTGGTTAAACTTGTGCACATATCTATTACATTGAGTTGACTATTTCCTTTCTCAAAATCATAGTCTGAAATTGATCAGTATTGGCAGTTTAATCTAGATTCCCTTTTCATCTCTGTTGTTTCTTGTGGTTCCTTAGCATTTGCAAATAACGGCCAGTCCCTAGAAAGGCTGAATATATATCTTTTCTCCTCGAATTCAGGTTTCTTCATTGGCCTAGCACCTAGTGGACGTGAAAATGAAGTATTAAACCATAATATAGAAGTTATTAAAAATTCACATGTTTTGAAGTTCTAGTTTGTGTTTATAATCATGATGGTGATTTATTGCCATTCTAAAATTTAAGCAGGACTTATGGAAATCTTCAAGCCTTTAGGAATATCAAAATTTTGATAATCACTTTATGTAGCATTTCTTTAAAGCAATATAACTTTACCATCTTGATGAGTTTTCAAATTAAAGGGATTTGAAAGTCTAAAATTGGCATTTCTTTGCAACATATTTGAGTACTGATATTTGAGTATTTAACCCAAAATCGTTCAGAGTGGAAAAAGCGAATCCATATAGCCGACCCCAAATTTTTGGGATAAAGGTTTAGTTGAGTTGAGTTGAGTTGTATATTTGAGTACTGATATCAAGTTAATAATTGTGGTTATTTTTATAGACCTATAAAAGTCAACTGGTAAATCTTCTGCAATACTGGGCATTTCCAATTTTAAATTCTGTTTTTAAACGTCTGGTTGACCTTGTCCCTGTACTAGTGTTGGTCTACTATACAAATCCGTATTCTTTATTCTCTCTATAGTTCCAAATCTTATGTAATATTATTTTCCATTTTCTTTAGCAGTGTCAGTTTGTGTTTTTCAGAGTCAAAACTTTGCCATGTGTATCCTATGCCAAGATGGCATAAACAGAAGTGAGCATGAAACATGTGTGAAATGGCATGGTTGAAATTTAGAAACAACAATTTTAAACTAGACAGATGGATAGCAGAAACCTTTCCTACTTCATCTAATTTTTGGTTTCTTTTTCTTTGCCACCATTCTTTGTTCGATCTGGTCTCGTTTGATGAGATCAATTGAAAACCAGTATTTTCACTGTAAATCATTATTCATATTCAAAAAGCCTTGATTTTGTCTGTTTCCTCACTGATTCTGCTATATTCATCTATGGGTTGTTGCCAATGATCTTGCCGCTACAAATACTGTACTCCTCTCTCTCCTTAATTTGTCCCAAACTTGTGCATCTGCTGCATTCTCTCCCTGTTATCATCATCATTATTATTATTATTATTATTATTATTATTATTATTATTATTATTATTATTTATTTTTTGTTTTGTTTTTTTTGTTATCTAATTTCCCTGCAAACTAGACAAAAAACAAAAGTGAAGCTGTGTAAAACCCACCAAGTGCAACCTAAGTTTGGCAATGTGTTGGACTTATCAATTTTGCTTTCAAATGGTCCTATAATCTAATGATATTGGGCCTTGTAGGCTCTATTTGTTTCAGGTTGCCTTTTTTCATTTTTTGTTATTATGGAAGCAAAATGGAAACCAATGTTTGGTTGCAATTTATGAAATGCATAATTTTGTCCTCTTTTTCTTATTTAGGATCACATCTTCAGAAGATCTAGGACCACTAAGTTCTTCCTCATTACTTCCCTCTTAACATAATCTTAAGTTTGCCCCTCCTTTTACCATTTTATTGTATTTGTACCGTTGCATTTATTGTTCTACATTGTGAGAAAATAGAACAGAAGAAGAATATTGAGAATGATTGAAGAGCTTGATTATTCCCTCAAGCCAATGGTGTCAATTTATAATCTGTACAGGACAACTAAATAGGAAATACAATCCTAATTAAATACGTAACTATAGCCACAATTCTAGCACCTAAATATATTCACACAATTACTACAGATACACATATCCTAGCTATTTATGGTACATATCTTAACACTTCCCCTCAAGCTGGAGTGCTCCAAGCTTGTTACAAATATATTCAATCCGAGAGCCTCTGAAAGATTTTGTTAGGATATCTGCTAATTGGTCATTTGAGCTAACAAAGCTAGTGGCAATACACCCAGATTCAATCTTTTGTCTGATGAAATGACAATGCACCTCTATATGTTTCGTTCTCTCATGAAACCCTGGATTAGAGGCAATGTGAAGTGCAGCTTGATTGTCACAGATTAGCTGCGTTTAAGTTCCCCATACTTCAACTCTTGTTTCAACCATATAAGTTCACAAGTTGCCATAGCCATAGCTCGATATTCTGCTTCTATACTTGACCTAGCAGCCACATCTTTCTTCTTACTTTTCCAAGAAATCAAATTACCTCCAATCATAATGCAATATCCTGAAGTAGATCGTCTGTCTGAAGGAGAACCTGCCCAATCTGCATCTGAGTAACCTATAATCTATGAATGACCCCTGTCTTCATATAATAGGCCTTGTCCTGAAGCTCCTTTAATGTATCTGAGTATCCGAATAACTGCATCCCAATGACTACTATATGGTGCTTGAAGGAACTGACTGACCACACTTACAGCAAATGAGATGTCTGGGCGTGTGATTGTAAGATAATTGAGTTTTCCAACCAATCTCCGATATCTACTAGAATCCTCCAATGGCTCCCCCTGTCCAGGAACAAGTTTAACATTTTGATCCATAAGAGTATCTGCGTGTCTACAAATTAACATGCTCATCTCTGTCAGTATATTCAAAACATATTTCCTTTGAGAAATGGCGATACTTGTCTTGGATTGTGCCACTTCAATCTCCAAGGAATACTTTAGCTTCCCAAGATCCATAGTCTGAAAATGACTGGACGAGTGTCGTTTGAGTTTTGAGATCCCAACATGATCATTTCTTGTAATAACAATGTCATCAACATAAACAACGAGATAAATGCACTTATCATGGCCATTATGGTGGAAAAATACTAAATGGTCAGCTTCACTCCGAGACATTCCAAATAGTTGGACTACAGTACTGAACCGTCCAAACCATGCTCTCGGAGATTGTTTCAAGCCATATAAAGAACGCCGTAGGCGACACACCAATCCAGACTCCCCCTGAGCAACAAACCCTGGTGGTTGCTCCAAATAAACTTCCTCAGCTAACTTGCCATGTAAAAAGGCATTTTTGATATCTAGTTGATGAAGTGGCTAGTGATGGATGGCAGTTGAGGAAATAAGAAGGCGAACAGAGGCAATCTTAGCCATAGGAGAGAAGGTATCACTGTAATCGAGGTCAAAGATCTGAGTATATCCCTCTGCAACAAGGTGAGCTTTAAGTCTATCAATCTGGCCATCAGGCCCCACCTTGACTGTGTAAACCCATCGACAGTCAACAGTAGATTTGCCCTTCTGTAGTGGCACCAAATCCCAAGTGCCATTGTTATGGAGACACTCATCTCTTCAACCATTGCATTACACCATCCTGGATGTTCTAAAGCTTCTCTAACAGTCTTAGGTATGGATACATTAGACAAAGTGGTGACAAAAGCATAGTAGGAAGGAGACAAACGATGATAACTCACAAAATTATGAATAGGATGAGGATTTCGAGACAAATGAATACCTTTTCGGATAGCAATGAGAAATAACATCGTCTGTTGAAGGTGAGACCGGAATAGGTGAAGATGAAGGAGGGCAAGAATCACTAGGAGCCGGTGTTGTACCTGTATTAAGCAAAGGAGCATCAATGGTGTTAGTGGGAGGATGAGGACGACGTGAGTAGACGTGTAGAGGTGGTGGACTGATCGGTGGTGGAGGAAGAGTAGGAACTGGTCAGGCGGTGGGAATAAAAACCTTGGATGCGGTGTTGGAGGAAAAATAGGGAGATGTTTGAGGATTTCGAGACAAATGAATACCTTTTCGGATAGCAATGAGAAATAACATCGTCTGTTGAAGGTGAGACCGGAATAGGTGAAGATGAAGGAGGGCAAGAATCACTAGGAGCCGGTGTTGTACCTATATTAAGCAAAGGAGCATCAATGGTGTTAGTGGGAGGATGAGGACGACGTGAGTAGACGTGTAGAGGTGGTGGACTGATCTGTGGTGGAGGAAGAGTAGGAACTGGTCAGGCGGTGGGAATAAAAACCTTGGATGCGGTGTTGGAGGAAAAATAGGGAGATGTTTCAAAGAAGGTTACATCAGCTGACACAAAGTATTTATTTGTAAGTGGATAATAGCATTTGTAACCTTTTTGAAGTCTAGAATATCCAAAAAAGACACATTTTATTGATTTGGGCTGAAGTTTGTCTTTGCCAGGAGTGTGATCATGAACAAAACAAATACAACCAAATACACGCAGGGACAACTGATGGGCTTCTTGGTCGGAAAACAAAATGGAATGAGGACTCTGATTGTGCAAAACAGAAGAAGGCATTCAGTTAATTAAGTAGCAAGCAGTAAAAATAGCATTTCCCCAAAAATGAAGAGGAACATTATGGTGAATGAGTAAAGTGCGGGCAGTCTCAACTAGATGACGATTCTTTCGTTCGGCAACCCATTTTGTTGAGGGGTATAAGCACATGAAGTTTGGTGAGTAATACTCTGAGAAGATAATAAATGAGTAAAGGGAGTAGACAAATATTCTTTTGCATTGTCACTACGAAGTATTTTAATAGAAATACCAAATTGATTACGTATTTTAGTAGAGAATTTTTGAAATATAGAGAATAATTCAGAACAAGTCTTTATTAAAAATAACTAAGTGCAACGAGAATAATCATCAACAAAAGTAACAAAATAATGAAATCCCAAAGTTGTATCGACACAACTTGGACCCCAAATGTCTGAATGGACAATTTCAAACATGGACGTAGCCATATTATTGACTCGCTTGGGAAAGGAAACATGACTTTCTTTCCCAAGTTGACAGGACTCGCATTCAAAAAAAGATAAATTAGAAAGACAAAGAACTAATTTTTGCAATTTCGCAAGACTCGGATGACCCAGACGATTGTGAAGAAGATTAGCGGAAGTGGTAGAGGCAAGAGCAACTGGAGAATTTGAAGTAGAAAGATGGTACAATCCTTGTGACTCACATCCTACTCTAATCATCTTCCCCGTACTCCAATCCTGCACAACAACAGAGTCAGCTGTAAAGGTGACAGAACAATTGAGATTTTTAGTCAATTTACTAATGGAGATTAAATTATATGGGCATTTTGAAGTGAACAAAACTGTGGTTAAAGGAATAGATGGAAAATTTTTTACTTCTCCTATGCCCTTAACTAAAGTTTGTGATCATTAGCCAAAGTAACTTTAGACAAAACCGGTGGAGAAACTAGAGATGAGAAATGACTTTTGTCACCGGATAAATGATCAGAAGCACCAGAGTCTAGAATCTATGGACTAATGAGCGAAGACTGTGTTAGACAAGCAAAGGAATTACCAGAATGAGCACTGCTAGAAGATTGTTGTTTGGCTGTTTGATATTGCAAATACTCCTTGTAATCAGCTCCAGTTAATAGGATAGAATCTGACACCTGATCCTTTCCATCAGGTAATGGAAGAATACCATTTTCACGGGATTGAGCCACATGAGCAGTTGATTGGCCCACATTATTTGACTGAATGGGGCGTGGTGATTGACCATGAATGGCCCAACAAGTGTCTCGAGTGTGATTACTCTTATCACAATAAGTGCAATGGAGCCTTTTACTCTTGCCTCTCTGATAAGTACCTTGTCTCTGTTGGTTTCCACTTTCTACAGTTAAAACTGAAGATTCAATTCCGGATAAATTATTCTTATTGAGTGAGATGCGTAGAAGCCTAGCAGACACATCCTCTAGAGTGGGAATAACCAGACTAGTTAATATCTGGTCTCTAACATAATCAAGGTTAGGTCTTAGCCCAATCAATGCCAAAACCATGAAGAGCCTGTCCCGTTGTTGCTTAGAAATATTATCAGTACATGGCATGATAGAATTAAATTTATCTTTCAGTGATTCTACCTGTCCCAAATAACTAGATATATCTTGTTGATTTTACTGCAGATGAACCATATCTGATATTACCTTATAAATACGCTGCACATCATTTGTATATAAGGTTTTTGCCTTAGTCCAGACTTTGCAACAGGTTTTACAAGACTGAAAAATATTAAGTAATTTTGGGTCTAGAGAATGCCACAAGAGACTACATAATTGAGCATCAATCTTAGTCCAATTAGCTCTATTTGTTAAAGCTATATCTATGGCATTTTTAACCGGGTGGTCATCATAATCCTGCCCCATAAACCATAATTTCACTCATGCAGCCCAAGACATATAATTTTGACTTCCTACTAGTTTAACAGTAGTAATAGCTGGTGAATTACCAATGGAAGAAAATATGGGTTTGAGAATCTCAGAACCTGTGTTAGATATGGGTGTGAGAATCTCAGAACCTGTGTTGGAGGGTGTGAAGGAGCGGAAGCATGTTGATTAGTTCATTAGAGCATTGAATTTCAAAATTTTCTTCTAGGGTTACATGCATCATACCACATCTCTTATGTGCCTAATTAATTTCAATGTTCAATTACATATTAAAACACTTTTAATATGTATTAGGATTTATTTTTGCCATTCAAGATTGTGAATTAACAAATTAATTCATTTAAACCCTAGTTTAAAAGAGAAGTTAGAGCACTAACCTTTTTGATGCACTATGGATGTAATTGGCACCTTTATAAAGCACCTAGGACCCCAAGTGTTGTCCCTCTAGCTTGTCCACACCAAGATCACCAATGGGCAGCCCCCAATTTGCTTCTCAAGCGTTTGCCAATCAATTATAAATTGGGTTTTTGCCTGTAGAGAGGTAGTATGTATGTATAGGACACTAGAAACAATTTCTAGTAATTTTATTCAAAGGATTTTGGGCAATTCTCTTTGAATTGATGAGACAAGATGAAGAGGAGAGAGAGAGAGGTGTGCCGCCACCTTGATGAAGAGAATAAGAGTGTTATGTTTCTTCTTTTCTTTTCCCTTTTATATAATTAGGTCATTAAGTCACTTAAACCCTATGCCACATGTTACCACCTTATTGTATCTTATTTTTAATTGACTCAATCACATTAAGCCAAGTGTCAAAGCTAGACTTAATCTTGACTTTGATCATCTTACATGATAGGAAGACAAATGGCAAGCTTATATGATGCCATGTGTCACCATCTCATGGTGCCACGTGTCACACTGCAAAATGACCAAAATGCCCCTGTGTCTTAATTTTGAGTTCTTAACCCAAAATAATTATTTTCTTCTTCTAATTAATTTATATCAAATATAAATTAATTAATTAATCTCTATTAATTAATTTCTCATTAATTAAATTCATATTTAAACACTTTAAATATAAATTTAACTTATATTATACATCCAATAACCTAGATTTGGTTTCAAGTCATGCTAGGGACTTTGCAATCTTATTGCAAACCAAACCTATTTAATTAATCAATTAAACTCTTTAATTAATCAATTAAATCACATTTTACTTGTTGATTATCTTGTGTATGTGTGTGACTCACTAGGCTCATCACTAATTGGCAATAAGATATGATATAAACTCTTAATGTCATCAGAACTCTTTCTTACAATAAATAATTTCTCTAAATCATTTTAGGCATCTCATAGACCATAGTTGTCACCTAGCATAGCGTGCCATGGCCACTCAATCAGTAACAAGGAATACCTTAAATGAACCTTTAATCATATGTTACCATGCACTAGAATCTCTCTGCTACAAAATCCCAATTCGAGCTGGAGTCATAGTGTATGTCAAACTCCATTTGCTATGAATATTATATTTTCCTTTAATTCCAGTTCTTGATTAATTAGATTTTCTTGTCAGAAACTTTTTTCTGATTAAATCTGTCTGTCCTGGCCAGGAACTTGAAACATCAAGAACTATTAAATGAACATAGGATTTTTATCCCTATTTACTTAGGGCAACAGATTCTATCTTGATCAACACCTATCTCCATATATAACTAGTAGGAGCTAACACATGCTCATATACCTATACACAGTACAAGTATGAAAGCAGTATCAAACTTAAACTACCTATATACAAGATAACTGTGTTATCTCAGGTCTAAAGATTATATGCACTGATATGATATATGACAATGCATTGACAAGAGTAAACTCCATGTGCTTGTCATATGCATCACTTGTTCGGCCTACTTATCATGTATAAGTGCCTATAATGTTTGTTATATGACATGAGACTCATTATTCCATCTTATTTATATCTTATATAAATACCTTGGGAACAAACATGAATACAATCTTTTTGGATAAGTCATGTTCTGTGTGAAGTATCCTCGATTATAAACCAATTTATGATATTTTGTGCTAGAAATACTGTCACTCATATTCTTAACAACTTAAGAATAGAATTTCTAACAAAATATCAATGGACCTTTTCTATTACACATAAATACATTATTTAAACGGAAAAGTGAAATTGCCTTTAATTAATAAAATATGTACAAGATACATACTGCATAATATGCTCTAGAGCCATTCATTACAATATCTTAGATCCATCTTCTCAAGATGTCGATCTAACTGAGCTTGTGACAAAGGCTTTGTGAATGGATCAACTGGATTTTCAGCTAGTGCTATTTTCTGCATTGTTACATCGCATTGCCTAACTCTCTCTCTGATAATGTGGTAGCGCCTTTCTATGTGTTTGGATTTTTGGTGAGACTGGGGTTCCTTATCCAAACGGCCTCTTTTGTAGCATCTGATGCAGTAATGTATTCGGTCTCTGTAGTGGAATCAGCAGTCTTACTCTTTTTGGAACTCTTTCAACTGACTGCACCTCCATTACAAATGAACACAAACCCTAAGGTAGACTTTCTATCATCAATATCTGATTAGAAATCAGAATCAGTATTACCATCCAATTGTAAGTCACCACCTTCATAAATCAAGAATAAATCTTTAGTTCTTCTCAAGTACTTAAGGATATTCTTGGCAGCCATCAAGTGTTCCAAACTTGGATTGGATTGAAACCTGCTAGTCAAACGAAGAGCATATGCGATATCCGGCCTAGTACACATTATTGCATACATCAAACTTCTAATAGCCGAAGCATATGGAATCCTGGCCATTTTATCTCTTTCTTCAGGTGTCTTTGGAGACATCTCTTTAAAAAGGTGGATACCATGTCTCACTGGTAATAATCCTCTCTTTGAATCAAGCATGTTAAACCTCTTTAACACCTTTTCCAAGTATAGACTTTGGGATAAACCAATTATTCTTTTCGCTCAATCTCTATAAATGCGAATTCCAAGAATATAGGTTGCCTCCCCTAAGTCTTTCATGGAGAATGTATTTGATAACCATATCTTGACAGTTGTCAACATACCTATGTCATTACCTATTAACAGAATGTCATCCACATATAAGACAAGGAAAGTGATAGCACTAGCACTAACCTTCTTATATACACATGGTTCATCCACATTTTTTATAAAACCAAATGACTTAATGGCTTCATCAAAACGGATGTTTCAACTCCTCGAAGCTTGTTTTAACCCATAAATAGATCACTTTAGCTTGCATATTTTGGAACCATCTTGGGATTCAAAAACCCTAGGTTGTTCCATGAAAATATTTTCTTCAATGTATCCATTGAGAAAAGCTGTTTTGACATCCATCTGCCATATCTCATAATCATAGTATGCAGCTATTGCTAATAGAATCCTGATTGTTTTAAGCATGGCAACAGGCGAGAAAGTCTCATCATAGTCGATTCCTTGCTTTTGGCGAAACCTTCTCGCTACTAGCCTTGCTTTATAGGTTTCTACCTTTCCATCAGAACAAATTTTTTCTTGAAAACCCATTTATTTCCTATAGGTACAATACCTTCAGGTGGATCAGCAAGGTCCCAAATTTGGTTCTTATACATGGAATCAATTTCGAATTTCATAGCTTCAATTCATTTTGAAGAGTCAGTATCTGATATAGCTTCTTCATAGGTAAGTGGATTATCCCCATGATCTACTTCTTCATGAGTAAAAAACTCTTGTTCATCTTTATGAAGAAAATCATATCTCACTGGTGGGTGAGATATCCTGGTTGATCTGCGAGGAATTACTGTAGATGTTTCATCAATAGGTGTAGGTTGGCTAGATGGATCTATATCCATTTGATCTGTTGGTTGGTCAGAATTCTCCAATTCTAACTCTATTTGCCTTCCTTTGCCATCTTCTTCAATGAACTGTTTTTCAAGAAATATGGCATCTCTGCTTACCACAACCTTTTGTGATGTAGGCAAAATAAAAATAATATTCAAAACTTTCTTTTGAATATCCAACAAATCGACATTTTTTCTAATCTGGTTTTCAATTTATCAGTGTTCAGCTTTTTGATATAAGCTGGACAACCTCAAATCTTAACATGCTTAAGACTCGGTTTTCTTCCATGCCATATCTCATAAGGTGTGGAAGAAACTGATTTTGATTGAATCCTATTTAGAATATGCAAAGCTGATTCTAATGCAAATCTCTAAAAAGAGATTGCCATATCAGTGTAGCTCATCATACTGCGTACCATATCTAATAGGGTATGATTTCTCCTTTCAGATACACCATTCAGCTGTGGCATTCCTGGAGGAGTCAGCTGGGAAACAATGCCATGCTTTTTCAAGTATTCATTAAATTCAGTACTTAAGTATTCACCTCCACGATCTGATCGAAGAGTTTTAATACTTTTTCTTGTTTAATTTTCTACTTCAGATTTAAATTCTTTAAATCTTTCAAAGGATTAATGTTTGTATTTCATCAAATACAAATACTCAAACTTTGATTTATCATCAGTAAAGGTAATAAAGTAATGAAAACGTCCTCTAGCCATTTCCTTAAGTGAGCAACTCCAAAATATTTTTAGCTCTTAACCCTTGTCCAACAAAAGGTGATCTAGTCATTTTGCCTTGAAGGCAGGATTCACAAGTTAGAGTAGGTCCAGAACCCAATGAAAATAAAATCTCCATTTTCTTCAGCTTTGTAATCCTATCTACTGCAACATGATCTAATCTTAAGTGCTAAATATATTTTGAACTTGAGTTGATTTTCATCATGGCATGACAAATTGCTTGCATTAGATTTGTATTTAACATTATTATCTAAATAATAAAGTTCATCATGCATATAATCCGAACCAATATATTTATTTTCAAAATAAATATTGCAAATATCATTTATGAACTGAAATTCATAACCATCTCTAGTCAAACTAGATATAGAAATGATGTTCTTAAAAGCATCAGGTACATGCAAAACCTTATGTAAATACAAAACGTATCCACACATGTATAAAGATTTAGATCCTATGGCTAAAGCTTCAACAGTTGAGCCATTGCCAATCCGGATTCTAATATCTCGTTACTACAAGCTGCTACTACTTGCTATACTCTGGGCAGTTCCTATTCCAGTGTCCATCCTTCTGGCAGTGGAAACACTTTCCTTTGCCTCCATCAGCTTTGGTCTTCCTTTTCTGTTTGGCTATCTTCTTGGAAGGTCCTGGAACTTGAGGTTTCTTATTCTTATTGCCCTTCTTCTTTTTTGACTTTCTAACATAAGAAGAAGATACAATCAAAGCTACTTCTTTTCCTTTATTGCCCGACATATTCTTTTGGGCAATAACAAGCATGTTTAGTAAACCGGCCAAGGTGTATTCCTGTTTAGTCATATGAAAATTTGTCACAAAATTCCCAAATGATTCAGGTAGGAACTGAAGGATCAAATCCGTCTGCAGTTGGAAATTCATATTAAAGTCAAGATGTTCTAGCTACTCCATAAGCCGAATCATCTTGTGGACATGATCTCTAACATTCTGTCCCTCAGACATCTTCATGCGGAATAGCTGTCTAGATATCGCATTCCTGCTGTGCTTACCATATAACTCTTGCAGGTGAAGAAAGATCTCACTTGCACTTTGCATATTTTCATGCTGCTTCTGTAACTCATTACTCATAGAAGCAAGCATGTAACACTTGGCTCTCATATTATGCTTCTTCCACTTGTCCAAAGTATCATGTTCCTCTTGAGTGGCCTCTGGAGGTAAGGGACCATGAACCTTTGAGTCTAGAACATATCCAATACGTTCTAAGTTCAGGACAAGTTTTAAATTTCTTAGCCAATCAGAAAGATTAGGTTCCGTCAACCTATTGTGATCAAGTATGCTCGCAAGGATATTGGATGGTCTTTTAACTGCTCATTATAATAAGAAAATTAACTGTAAAAAATAACTAGATTAATTAGTAAATGTATCAAGTAATGAACCAAAATTGTAACACCCCTCACTCGACTACAGTGTAGCCGAGTGAAGTGTGCTAACTCCTACTAACTTGGCGAATCCTACACTTCCAAAGTAGGAAACGGAAATCCTAGTTGGATGGATTTCTAGTGGGTGATTGAATTCTTATAGTCTTATTGATCATCCTTAGGCACATCTATTATTGGAATTACAATAAACTATAAGTGAGCAACTCTTTGCCCATCACATCTCATGTGAGGTTCAATCATTCATCTAACCTCTAATGCTTAAAATCTCAGGTACATCCATTATTGATTTATTTTACATTATTTAAGTTGATCCCATTGAGCCAGTAAACATGCAAATAAATTTAATGTCCTCAGGCACATCCATTATTGGCCATCAACTTATTTATATATTTACAACATCTCATGCTTAACAATTATTCTTAAGAAAATCTCTTAAATTAAATGCATCACATGCAAGTATTTAAAATTTCTTAAAATAATTGCCTCAATGGAGGGTCTATGATATAATTACTTTAATTATACCATTTCAAACTTAATCATTTGTTTGGAAAATTTAGTGGTCGGCTTAATTACTATTATGGTCTCACTTTGCATATTATCCAATTAGCATGCATATATCATATACTTGCATACATTCCCATGCATCTCATGCATACATGAATAAACAATAAATATGGTATAATCATGGACTTTCTAAAGGATTCAATTCTGAGCCATCAAGAATTGAATCAGGGTATTCCTAGGTGCATTTCATTCATTAATTTTACAAGAGTTGCTGAAGGAGTACATAATCAACACTTGATCTTGATTTCCTCCCATTGGTCCCACCAATGCTCTTGATCTTCTTGCAATTCAATTATATTGGAATCCTTGACATACCAAGGCGAATTTACAAGATCATGGACTTTAATATCCTCAAATAAATGAAATTACAACCCAAATTATTACAACACTTATAATACATACTACTAAAATAAATTAATTATTTTACAATCCAAAGAAAATAAAAGAAATAAATCCAATCACATTGGTCTTTTATTGTCCATGATCATTCATCATGCATATTACCATTTAACAATTAAATAAAACCTACATACTAAAATTAAATTGAATATCTCATATTCAACTAAAAAATTCAGATTTGAATCTCATTCAATCAAATTTAAAAATTCAGATTTGAATCTTATTCAAGCAAATCAAAAAATTTAGATTTGAATATCATTCAAACAAATTTAAAAATTCAGATTTGAATCTCATTTAAACAAATTTAAAAATTCAGATTTGAATCTCATTCAAACAAATTAAATTTAGAAACTCTTTTCAAATTTAATACCTTGAAAACAATTTTCAATAAATTAATTGTGATTAAAATTCCTAATTAAACAACTTAATTAGGTGTGGGCCTAATTATGGGCCTTAAGATATACAACAATTGCATAAAGAAGCTCAAAACCAAAACACACCCTTAGGGTGTGATTCACAACCAAGCCGCCACACATGAGGAGCATCATCTTGATGCCGCCACCTTTGGAATAACCAACCAGCTTTGAATCAATCAACATTTGATTCAATCACACAATTAAATCTCATATTTAATCCTAATGGCAAATATAGTGGCTCTGATACCAATTGAAGGAGCGGGACCATGTTGATTATTTCATTAGAGCATTGAATTTTAAAATTTTCTTCTAGGGTTATATGCATCATATTACATCTCTTATGTGCCTAATTAATTTCAATGCTCAATTACATATTAAAACACTTTTAATATGTATTAGGATCTATATTTGCCATTCAAGATTGTGAATTAACAAATTAATTCATTTAAACCCTAATTTAAAAGAGAAGTTAGAGCACTAACCTCTTTGATGCACTATGGATGTAATTGGCACCTTTAGGAAGCACTTAGGACCCCAAGTGTTGTCCCTCTAGCTTGTCCACACCAAGATCACCAATGGGCAGCCCTCAATTTGCTTCTCAAGTGTTTGCCAACTAATTATAAATTGAGTTTTTACCTTTAGAGAAGTAGTATGTATGTATAGGACACTAGAAACAATTTCTAGCAATTTTAATTCAAAGGATTTTGGGCAATTCTCTTTGAATTGATGAGACAAGATGAAGAGGAGAGGGAGAGAGGTGTGCCGCCACCTTGAGGAAGAGAATAAAATTGTTATGTTTCTTTTTTCTTTTTCCTTTTATATAATTAGGTCCTTAAGTCACTTAAACCCTATGCCACATAACACCACCTCATTGCATCTTATTTTTAATTGACTCAATCATATTAAGCCAAGTGTCAAAGCTAGACTTAATCTTGACTTTGATCATCTTACATGATAGGAAGATAAATGGCAAGCTTATATGATGCCATGTGCTACCATCTCATGGTGCCACATGTCACCATCTCATGGTGCCACATGTCACCATGTGAAATGACCAAATTACCCTTATGTTGTAATTTTGAGTTCTCAACCCAAAATCATTATTTCTCCTCTTCAAATCAATTTATATCAAATATAAATTAATTAATTAATCTCTATTAATTAATTTCTCACAATTAAATTCATATTTAAACACTTTAAATATAAATTTAACTTATGCTATACATCAAATAACCCAAATTTGGTTTCAAGTCATGCTAGGGATTTTGCAATTTTATTGCAAATCAAACCTATTTAATTAATCAATTAAATCACATTTTATTTGGTGATTATTTTGTGTATGTGTGTGACTCACTAGGCTCATCACTAATCGACAATGAGATATGATATTAACTATTAATATCATCAGAACTCTTTCTTACAATAAATGATTTCTCTAAATTATTTTAGGCATCTCATAGACCATGGTTGACACCTAGCATAGCGTGCCATGGCCACCCAATCAGTAACAAGGAATACCTTAAATGAACCTTTAATCATATGTTACCATGCACTAGAATCTCTCTATTACAAAATCCCAATTTGAGCTGGAGTCATGGTTTATGTTAAACCCCATTTGCTATGAATATTATGTTCTACTTTAATTCCAGTTTTTGATTAATTAGATTTTCTTGTTAGAAACTCTTTTCTGACTAAATCTATTTGTCCTGGCCAAGAACTTGAAACATCAAGAACTATTAAATGAACATAGGATTTTATCTCTATTTACTTAGGGCAACATATTCCATCTTGATCAACACCTATCTCCATATATAACTAGTAGGCGCCAACACATGCCCATATACCCATACACAGTACAAGTATGAAAGCAGTATCAAACTCAAACCACCTATATACAAGATAACTGTGTTATCTCAGGTCTAAAGATTATATGCACTGATATGATTTATGACAAAACATTGACAAGAGTAAACTCCATGTGCTTGTCATAAGTGTCACTGGTTCGGCCTACTTATCATTTATAAGTGCCTATCATGTTTGTTATATGGCATGAGACTCACCATTCCATCTTATTTATATCTCAAATAAATACCTTGGGAACAAACATGAATACAATCTTTCTGGATAAGTCATGTCCTGTGTGAAGTATCCTCGATTATGAATCAATTTATGATACTTTGTGCTAGAAATACTGTCACTTATATTCTTAACAACTTAAGAATAGAATTTTTAACAAAATATCAATAGACTTTTTTTATTACACATAAATACATTATGTAAACGGAAAAGTGAAATTGCCTTTTACTAATAAAATATGTACAAGATACATACTAAATGATATGCTCTAGGGCATACTACTAACAGGCTGTATTGAAGGACTCCTCAATCTCAGACATGTTGGCAACAGAAGCACACTGAAATTTTGCTTAGAAATACGTCAGAAAAGCCTACTGTATGAAAAGGTAGTGACAAAAAAGTGAGAAACAGGTGACGGGACCAGTGTGGTTGAGGCTGATCCTGGGCTGGGTAATCCACTACGCTAGGCTGTGACAGCCACAGACTCCCAGAAAGTGACCGGAAAAAGAGGGAAAGTTCCTCCCTATGAGGACTTTGTTTCAGATTTCAAATCAGACCCATCGAGGGCTTTGATACCATGTGAGAAAATAGAACAGAAGAAGAATATTGAGAATGATTGAAGAGCTTGATTATTCCCTCAAGCCAATGGTGTCAATTTATAACTAAATATGAAATACAATCCTAATTAAATACGTAATTATAGCCACGATTCTAGCACCTAAATATATTCACACAATCACTACAGATACACATATCCTAGCTATTTATGGTACATATCTTAACATACATTGCACATGTCAAACCATATTACTCCCCATTATCTAAACTTAGCCTCAATCGGTGCTATTTCCACCATACTATGAATGTTTTTAATCCTTATCTGATCCAACTTTGTAGTTCTACATTTATTTTGGCATCCTCATTTCTTCAACTTTGAGCTTATGCATCTGTTATTTCTTCCCAACCCAACATTCACCAACATATATCTTAATGGGTCAAATCAAGATTCTATAAAATTTCCCTTTCAACTTAGCTGATACTTTACAATCACGTCACGTGGAACTCATTCTACACTTTGCTGTTTCATCCATCCCGTCATAATCCTATAGTTGAAATCTTGCCGCTGCTTCATCTTTTTGTATGCTATAGTCTAGTTATATTAAATTTGTTCTTTATGTGACATAAAACACAAATTTAAGCTTGCCCCACTCAAATTGCATTTGCATCTATTTCTATCACAAAAAAAATCTCTTTCTATCACAAAAAAGCAATGTATATACTGTGTTGTAGTTCTACTTAATTTGAAGCTTTTTGGACTTTAGAATGCTCCTCCATAACTTTTACTTTCTGTTAACTCCAGCTTAGGTTTCATCCATTAAAATTATCTTATCTGCAAATACCATGTATACCAAGGGATACCCTTTATGGTTATGGTTTGTCAGCTCATCCATGACCAAAACAAAATGAAAAAGGCTCTAGGTTGATCCTTAATGGAGTTCAACTATGATAGGGAAATATTTTGTATCACTGGCTACCTCTTCATGCTTGTCCTTCAACAATTTTGTATCAAAAAGATAATCCTGCCTCTTCATGCTTCTCCTTCAACAATTTTGTATCAAAAAGATAATCCTGCCTCTTCATGCTTCTCCTTCAACAATTTTGCATCAAAAAAATAATCCTTTCTGTTTCAAAACCCTTGATAGAACACTTTTTAGGAGTTGCAAGCTTTGTTTAAATTGATAAAACCATATGGAGATCATCTTTCTAAATTTCTCCATTGCTTTCATAGTTGATCTTCTTAACATGAAACCAAAGTTCTCCATCAATCCTCTTATTAAAATTTTTTCTTAGAAAAATAAAATTTTGTAAAAAGTGTTTATAATTTTATGTAGAAAAATAAATATGAATCTCACTCTCAATATTATCGTCCAAAATAATAACAATGTTGTGTTTTTTCCAACGTTCATGTAACTTATACCAATTATACATTTTCTGCATCCAATGTGTATTGTGCCCTTGTAATCTTTCTGCTTGAACTAAATGATGAATGATGTCTTAAATCCATATAAATTGTAAACAACAATTTATAGTCAAAATAAATGAATAACAGTTCTCTTTCTGTTTATTCATATATGTTGGTTGATTTTGATATCCTCAG**GCTGTGTGATGAAGCTACAAGTGCACTTGACAGCACAACAGAAGCAGAGATATTAAGTGCATTGAAGTCATTAGCCAATAATCGAACTTCAATTTTCATTGCTCACAGGCTTACAACTGCAATGCAGTGTGATGAG**GTGAGCATTTTTTATATCATGTACTTTTGTTAAGATGCTGAAATTTTTAGGGTGTTATTGAGAAGAATTATTTCAAAGTCTGTAAAGGAAACAAGAAAGAGCAATTTATTAATCATGAACTTTGATTTGGTGGGATGATCTTGTGGTGGATGTCATATGGCAATTAGATTAACCATTAGTTCAGATTACTGTTCACAATACAGTAGCATGAATACATTTGTAAGCACGTGTATTTCTGTAGTTTCTAATTCTCCTAGGATAGGCCAAGCTATAATTTTTTTAGGGTCTTAAATTATGTCAAATTTCTATAGGATTAGGAGCACTAGTAGTCTATATGATGTAGAACACAATAAAGCACTCAAAAGGGGATGAGAAATAATTCTCAAAATTTGTGTTAATTCTTAAAACCAATTCTTATGTTGTTCATAATAAAATTTCTAGATAACTTGGGTATTTATAGTATAAGATTAATCCTAATGCTATTAGGATTATGAATCCCAAAATAAGAAAAATATTAAACCAATACTAACTAAGAATGTAAAACAAACACTAACTAGGAAAATATTAAATCTAATAGAACTCTTTAGATACTCCTAAATAAAACTAGAATTCCTAAATAATGAAAATCTAAACTTCTTGATTATACAATGAAAAATCAAATCAACATAATTTCTAAATTGAATCAGACTTGACTGCATCATTCTCCTCTGGTTGAAGAAAACTTGACCTTGAGTTTTGTAGTGATAACGAATTAAAAATTAAAATTGGAGAACACAACCTTATCTTCCTGATGGTAGCCATAAGGTTGAAATTGAAAAAACTTCATGCTTTTCTTCACATCCCTAGAAATATATGAATTTATAAAATCAGTCTCGGTTATAGTTGAAAGGCACTTAAGTTTGATACTTCAATAGGAATAAGAGATTTAGATAAATGTTCAATTTCTGTTGTCGTGTCTATGGTTTGTTTTTCTTCTTTTCTTCTTTTTGTAGGATATATTCCTTAGGCTTTTCTTGCTCATTCTCATCAGCTTCTTTTTTCTAGAACGTGTTTCTCGAAATTTTCACACTCAGTCTTACCATCTTAGATTTCAACATGTGAAGGCTCAACAATTAAAGTTTTGGATTCTTCAAGTTGCTCCTTATTCTTCATTATGAATTTCTTGTCTTGCATCCTTTTAATTTTTAAATGCATCTTCAAGAACTCTTGATATTGCTTCCTGCTTTGTTTTCAGCTTATTCAAAGTTACTTCAAAGTGCTTCTCATATTTTCCTTGAGATTTTTCTTCTTGAACATTTGAGCAAGCGTCATAAGAGGGGATTGTAACAAGTTGGGTATTTTTGGGTACCTGTTACTCGAACCAATTTATATAAATAATTAATTAAATATAAAATATACATTTTTATAATAATATTAGTAAATTTTTTGATATATTTTAAATTTAGAAATTTGAATATTTTTTAGAATTATTAGAATTTTAAATATAATTACTAATAAAAAATATTTTATGTAGATTATTAATTAAAATATATAAAGTTAAATGGATTTAGGTATTATTTGGGCAATAATAATTGGGTTTGAAATGAGTTCAGGTATTGAAAATACTAAGCTAGTTTGGTTTGGCAGGGTGATTTTTGTTGGTACCCTACCTTATCCATGATCAACTTATTTTTGGTTGAAATAAAGTTCTCCAATATGCTTTATATTCCTTCTTCCTTCAAGTTCTCTCTCCTTGACTCATGGAAGTTGTTTGATGATTCGATATTCGTAAAATAAGTTACGAGGACAATATACTGCATCGCCAATAGTCATTGATTTCAGCTCGTAGTGTCTTAATTCCAATTCACAGTCTCACTAAAAACAAAATTAAAGCTTTACAAGCTTTTAAATACCCCTTTCTGCTCAAACAAATTTCAAAATTGAAACCAGGATGTTGAAATTAGCGGATAAGGTTTGCTAAAATTTGGTGATGTGGCAAGTGTCAGATGGAGTGGCGTTGGCATGCTAGGCAGTGTGGCACTATCATAGCAGGTGGTAGGGTGCTGATGTGGTGAGGCAGATGGTGTGGGGCAGGTGCACAGCAGTCAGCATGAGCTCGAGTGTGTTTCTGAGTGGGTCGGACCCGACCCAATATGGGAAGGCATTTGAGAGGTTTGAAGAGCAACCCGACAATGGTGACTCATTAATCAGTGTGCGACGAGTCTTTGGCAAGGTCTGTAACACTAGGGTTGGGTCAAATAGTTGTATGGGTTGAAACTTGACTCGTATAGATCTTGTGGTAATGACGTCGGACGTGAGTTCCGATAGTGGGAATGACGTTGCCAGTGGATGGCAAGATGCGGGAGAGGTACAGGCAATGAAATATATTGAGTTTTGGTCCTTTTTTTTTATTGGTTGAAATCTGACCCAATTTGATCTTAGTTTTCGGGCAAAGTCTTGCGGCCTAATTTGCACCAGAAGAGTCTCATGGCTGGAGAAAAGGGCTTGATGGTGGTGGCATGGCTACTTAATAAAGGCAGAGTGATCGGTTGTGTATGTTAGTAGCTAGGGCTTTGGGAAGAAGAGGAAAAACGTGATAATGGCAGTGGCAAACTGTTTTTTTCTTTTAAATTTGCACCAGAAGAGTCTCATGGCTGGAGAAAAGGGCTTGATGGTGGTGGCATGGCTACTTAATAAAGGCAGAGTGATCGGTTGTGTATGTTAGTAGCTAGGGCTTTGGGAAGAAGAGGAAAAACGTGATAATGGCAGTGGCAAACTGTTTTTTTCTTTTTTTTTTTTTTTTTTTTTTTTTACACAAAGGAAAAAATTTAAAACTTAAGGGAACAAAATTTTTCGAAGGAAGCTAAAACTCAAATTGGGTGATAATTGTTCATGGTCAACAGATCAAAGTGGATCTGATATCAATTTGATGTAGAACACAATAGAACACTCAAAAGACAATGAGAAACAATTCTCAAAATTTATATTAATTCTCAAAAACAATTTTCAAGTTTTGAATAATAGAAGTGTCTAATAAAATGCTTAGATGACATAGGTGACACATGTTTATAGTATGATATTGATCATACTACTATTAGGATTAGGATCCACTAGCGAGAAATATAAAACCAATGGTAATTAGGAAAATATTAAGTCTAATAGAACTCTTTAAATATTCCTAAATAATAATAGAATTCCTAAATATTAGAAATCTAAACTTCTTAATTTTGCAATGAAAAATTAAATCAACATAATTCCTAAATTGAATCTTCGTTGATTGCATCACTATATCGATGTAATGCGTGTTGATTGAAAAGGTCATCCTTATATTCAGTTGTGTGGTGCAATTATAATCCTATAAAATGGAAAATTTCTTGATATTAATGAGTCCTATATTGTGTTTCAAAAAATGGACAACATGTCCTTTATTGGGATCACAAGTTCACAACAGAATTTTGGCTTGCTGATACAGGATTTCCCAATACTTTTCCATGAATTTCATATTTACAATTATTTTGTTCATAATTGAATGATAAGGACAACGCCTGATACTTTCCACCTTTTTTTTCCCTATGAACCAAGAAGATGAGGAGGAGAGGGGTGGTGGCATACCTGTGTTATGCAACCTTTGGTCTTTTTTATTTTTAATTTTTTTCCCTCCTTTTAAAAACCATAAGTATTACTTTGGCTTCTTTGTATAGCCATTCCAATCGGTCTTATTTTTAATTCATATTTAGGCAAGGTTTTACTTTTAATATTTTTAAACAGAAATATATATATATATATATATACTGATTTCATTTTTCTATTTTTATTGTCAATAAGTTTGTCTTTCTATTTTTATTGTCAATCAGTTTGTCAATCTTAAAAAAAAAAAAATATTTCCACTAAAAATATTGCTATATATATAATCAATTATGATTGGAAAGTCATAATTTGTTTTAGATGCTGAGATTGTATTTTTGTTAACTACAAATTTCGTTAAATGACATTGACTTGGTTATATTTAAAACATAAATTAAACTTTTGATTGGAGTATTTTCTGTACCAAAAAGAAAATAAATTTGAGTTTTTTTTTTTTCTTTTTAAAAAAAGAATCATGGTATGCTAGTTGATTTAGTACAATCGCGTTCTAATTTTGTTATCCTAAGAAACCCTAACCCTGACTTTAAATCAGTCTATCTGATCCCCTTGATGTCCTACATCAATCATCTCTTACTCCATATTTATGTATGTCAATATGAATATCTTCACCCACTTTCAGACGCAATGCTTCCAAACTACATCACTTTTCAGTTGGCATATCTCTCTGCTCCTAGTTTTTTCTGTCGGAATAATGTTGCATATATTGTTATTGGCAAGATTATGAGATTTGCCTTCTGCTCATACCATTTGATTGTGGGTATAGCAAAATAGGCCAGCCAGCAGTCACACCTTAGGGCAAAATTGAGTTTTGCAACCATATATTTGAAATAATAAGAAAATGTCTGTATAAAGTTAGATATTTTTGCTGCATAGTGTAGGACTGTTCACCAACTAGTTTTTAAGTTTTTTGTTCGATGCTCCAACCTAAAACTGTCTATTCAATTGAACAATGTATTTTATTGCCATCTTTGCACTGTAGCATCTATCTGTCTTTTGTTTGTTCTTGTACCTGCAATTCTACATTTGACATTCTTCCTTCCCTCCGAATATGGTTTCAG**ATAATAGTTCTGGAGAATGGGAAGGTGGTTGAGCAGGGACCCCATGAAGTTCTATTAACCAAGGCAGGGAGATATGCACAGTTATGGGCACAGCAAAATAATTCGGTGGATTCCCTTGATGCAGCTATTAAATTGGAGGCATAACTGTTATCTTCCTCTCTATTTAACCCTTCGTCCTGGTACCATACAAATAGAATGTTTACTCAGCTTTTTATTTTTGTGAGTAAGCCCATGTAATGGGACTTGGGCACTAGGGCACTTGATGGGTGACTCTTGCGGGGTTGCATCCTCAGAATGAGTGTGCAAAGGGGTAACATTTGCCATATCTTGGAAGAAAAGAAGCAACAATATGCTACATAAGAAGCTATTTGGAACTGAAATTATGGTGAGATGTACATAAGCCTTCACAACTCACTCAATAGCATAGATCAACAAAGACTTCAGATCTAATTTTTCTCACATATCTATTTTCTGGTCCTTAGATTGGATGAGATTGAATATAGTTGAGATGAAC**

>HbABCB26 scaffold2073(10341-17918)

**ATTTTCTCTGGAGCTCTTTCGTCCCTGAAAAAGCAAATTTCTTACGCCCCCTCTAAATGCCCCCAAAATCATCTGCACCACAGGGACTAGAATAGTTGAAAACTGAGCAGAATCCATGGCTCTGCTGCTGTGCAACCCTTTAGCACAGCGTTGCTTACTCTCTTCGCTTCACTGCAAGAATCAACGACCTCTCAGCATCCGCCTAGCTGCCAATACTAAACTCCGATTCTCTCCCTCTGAATTTTCCCTCAGCAGTGGACGACGTTGCTTCTGTCCTCTGAAGTCTTCTTCAATTAACGGATTTTCCATCGCTAAAAAGGATCATGTAGAGCAGTTTGAAGGAGAGCAGCGAGAAGAAAATTTTGAGCTGCATGGGAGAATTAGGAAGTTTTTTGAGTTTCTTCCATCAATTTTGCCTGGCGGAAATTGGTGGAGCTTCTCTGAGGACGTGGAGATTAAATTTTTAGCCAAACCGGTGACCATGTGGCGGGCGCTTGGTCGGATGTGGCAATTGGTTGCCCATGATCGTTGGGTTATTTTCGCCGCTTTCTCTGCTCTAATTGTTGCAGCG**GTGAGTATTCTGAATATTTCTTTTATCTTTTTTTCCCCACTTATGGAACCCTTTATGTGATCTGCTGTACGGACAAGNNNNNNNNNNNNNNNNNNNNNNNNNNNNNNNNNNNNNNNNNNNNNNNNNNNNNNNNNNNNNNNNNNNNNNNNNNNNNNNNNNNNNNNNNNNNNNNNNNNNNNNNNNNNNNNNNNNNNNNNNNNNNNNNNNNNNNNNNNNNNNNNNNNNNNNNNNNNNNNNNNNNNNNNNNNNNNNNNNNNNNNNNNNNNNCATGATTATTTTTTGGTTGGAGGTTGGATTTTTTTGGTCTTTTCTTTGTGCTGTGGATTCAGTTTTTACCAAGGCTGCAACTTAATATGTCGTGGAATTCAATACTGGCATAATTTCATTTTCTTGAAACATAGGCAACATAATTAGTTGAAGTAAAACCTCCAGTTACATAAGATTCCCTTATAGGTTATATTTTTGGCTGCGGGTGCTGGTCTTGCATTGGGGTTTATACTGCTGAACTTTGAAGTAACTTGTGTGTCTGATTTTATTTGAAGATAAGCTGTCATAATATAGTTTTCTTAATTTTTTTTTTTTACTAAATTCGTCATGCGCTTGGCTCAG**GTTTCGGAGATCTCAATACCACATTTCCTGACAGCGTCGATCTTTTCTGCCCAGAGTACTCAAATTGCATTATTCCATCAAAACGTGCGACTCTTGGTGTTGCTATGTGTTATAGCAGGAATATGCAG**GTCAAAATCTTGTATGGTCGAAAGTCCCTTCTTCTTTAAGCTGTAAACTACAACACGCAACCCTTTTTCTACACCTGCTCATGTATAGGACTGATTGGAAGTGAACAAAATTTACAATCCGATCATTTTATATGAGATTTTTTTTTTGTTTGCATTTTCATAAATAAAAAATTGTTCACTGTTTCATATTATTGATGTGCCATTAATTTGTTATCTTACTGAAAATGTAGAACCAATTCAGTTTTATATGCAATATCATATCATTTTCTGTTCACTGTATTGAACTTGTGCCTCAATGAGACCTTCTATACTCTGTGTATGGAACGTGATCCTTCTTAATGAACTTTATGACTGCCCTACTGAACTCGTTTTCTGTTTATATGAACTTCAG**TGGTCTACGAGGTTGCTTTTTCGGCATTGCCAATATGATTCTT**GTGAGTTGCTCTCTCATTGATTACTTCTTCAATTAATAGAATTATCATATGCTATTTTTTGCTTCCTGGTTGCTGCCATTGTCTGTATTATTTTCAATCTTGCTTTACACAAACATCATGTCTGAGTTAAAGAAAATCAGGTTGACTCAATTTTGTACATATGGTAAATAAGGGAATGTTATTTGAAACAATATTTCCCCTTTGGAAGGATGACTGCGATGCTGACGTGTTCCTGTAATATTTATAG**GTAAAGCGAATGAGGGAAACACTATATTCTGCTCTTCTTCTTCAG**GTTCATTTTACTCTGATCATTCTATTAATCTGTTGCTATTGGCATTTGAAAATGCTACTCCAAGCTATAGTTGCCTTAATATTATGATGGATGGATTAACTTATATTGGTGTCTGTTGTAG**GATATATCTTTTTTTGACAATGAAACAGTTGGTGATTTGACAAGTAGGCTTGGATCAGATTGTCAGCAAGTGTCTCGGGTTATTGGAAATGATCTTAATTTGATTCTACGCAATGCTGTACAG**GTTGTTTGCTGATAAAATTATCAATGCCACTTGTTTGCTTGCAATATGAGAAATTAACTTCTTGTTTTATGCCTCTTCTAG**GGTACAGGTGCCTTGATCTACTTGTTAATTCTGTCCTTGCCACTTGGTCTGTGTACATTGATAATATGCTCAACTTTAGCAGCTGTAATGCTGATATATGGCAT**GTAAGATTTTTTTACTTTTTTCCTTGCTGTTCCTCCATTACACTTGAGGGAGCGTTATTGAAAGGGCACCATCATAATTGGGTTTGATGGGAATGGGGATGTTTGTTAGGTTCCTTGCAAAACAAGGAAGGACACATTGGAAACAAAAATGTTCATGTAGGAAATAATGCTTGATTTGATTAAGTGTGATGTAGTTTAAGTAAATTTCAACTGAAGAAAGGACTATTTAAGTAATAGATTTCTGAATCTCATTAAAAAAGTGCTTTAACTTTGATGTTTGGAGAAAACATTGTAGAATAGAATTGTGTGGACTTTGACTTGTTGCTGCATATTTGCATATACAGTTATACGCAAATTTAAAATTTATTGCTTTGATGTCAACTTATGCATCCTATTTGTGAACTTATTTGGGATAAAGGCTTAGTTGTTGTTCTATTGGGTCCTTATTTAATTATGTTTTTGTTGTTACTTTTCAG**GTACCAGAAGAAGGCCGCAAAGTTAACTCAAGAGTTCACTGCTTCTGCTAATCAA**GTAGAATAAGATGATTTATCTCTAAGTTGTTGGATAAAATACCATTTTTTTATTCAGTGATTTTGGAAAATAAACAAAATTTCATCATTTTTATTTAG**GTGGCACAAGAGGCATTTTCTTTGATGAGAACAGTCCGTATTTATGGTACAGAAAATTTAGAACTTGAAAG**GTAAATGTTATTTAGAACCGTAAATTTTCTCAATAATAATCTTTATAAGAGCACTTCTTAGCAAATGCCAACAGGCATATATGCTAACCTAAATTAGTTCTGACAGAAAGACATTATCTTCGGTTCATAG**GTACAAACTGTGGTTGGAGAAATTAGCTAGTATAAGTTTGCGACAAAGTGCAGCATATGGATTTTGGAATTTGAGTTTCAACACACTTTATCATTCTACTCAG**GTTCAGTTCTTTGTTTTTTAATTTTTTTTTAATTCTAAAAATGTTAAAAGTTATTGAGTATTCCAGACTGAGGTAGCTACTGTTTGCTGCATTTGGTTAGCTATTTCACAGTAGTTATTAGAAGTGTATTTCATGATAGTAATTTTATAATATATATTGTTTGTCAGCAAACTAAATATTGTGGTATAGTCTAACAGCTTTTATGATACTTTTATTTGGTTGGTTCTCTTCCTTTCGTCTTTTTTTTTTTCTGTTATCTTTTCTTTTTCAGTTAGTGCATCCTTCATTGAACCAAGAAATTTTTTCTGGTGTATCTGTATTAGTATGCTAGCTTATGAAATTTATTCCAGAGATAGATTTGCCCATACTAGTTGATTCAACTGACCATTCTGTCCTAGCTCGATGCTTTTGGAGCACCTCAGGGTTTTTTTTTTTTTGGGCAGCAAGTTCTATCCTGTATGTTATGCCTTGAACTGGCCTTCAGCCTGACCTGTCCTGCTATATTTGTAGTCTGATCTCTGAAGCTGCAGATGCTAATTAACCATTTAATTGTGAATTGTGATACAATCCTTTATTTCTTATTGATATATTGTAATATGTTGCATTTGGATTAATGAGTCAACTGTCATGCGTGTATCTTTTTGAATTCATTGACATACAATTCTAGAATTAGACACTTTAGTTTATATCATACTGAGCATAAGAAAAGACTTATTTATTTGAAGTCTAGGTTTTAGATAGTGGTTCTTTGGTCTTGTTTGAGTTATGCAACATCATATTGCTTATATTGGCACATTTAATTTTTTTTTTTCAG**GTCATTGCTGTGCTAGTCGGAGGAACGTTTATTCTGGGTGGTCATATTACAGCTGAGAAACTTACAAAGTTTATATTATATAGTGAGTGGCTGATATATTCTACATGGTGGGTGGGGGATAATTTATCTTCTCTTATGCAATCTGTTGGGGCAAGTGAGAAGGTCTTCCAATTGATGGATCTCTTGCCAAGTGACCAATTCATATCAAAAG**GCGAGTATTTCACTAACTCTACAGATGTTTTAGATTATTAGATTCTTTTATTTTGAGGAAGTCCTTTTCATGCTTTTTGATTAATGGCTTAATTAAATCTCTTTAGAGATTGCAACATTAAAATGCACATGCATATTCATTTCCTCCTATGAGTTTTTAGAAGGTCTTCCAAATTAGTGAACACAATTTCGAAGACAGTGCATCTGTTCAATTTTGTTCTTAATGAATGCCTGCAAACATTTTATGATGGTTTCAAGTTTTTCCAGCTGTGCACTTTGGTTTGGCTGCTTGAACCTTAGTTGAAAGCTGGAATGGTTGATTATAAAACTGTCACCAACTTAGCTGTTTGTCAAGAAAATGCAATAGCATTTGCTTTGGTTTTGTGAAACAGTTGATTTTTTTTTTCCTAATGATTTTTAAAGCAAGTTTATGTTATAGTGATGATAAAAGGAAAATTTGGCTTCCTAGAGGATGAATTCTGGGTTTTACTTTCTGCACATCTTATTCTTCATACTAGTTTATTTTTTTGGATTCTCCACATGCACAACTTCAGGAATTTCCCCTCGTACTGGTTTAGTAGTTTGGATTTTGGGTTTTACTGCTTTATAG**GTTTGAAGTTGCAGAGGCTGGTGGGACAGATAGAATTTGTAAATGTATCCTTTTATTATCCATCAAGGGCGGCG**GTACGTTTCTCATCTTCATCATCTGGTTCCTGATATCGTACCCCTCTTCTTTTGCTAATTTACGCTCTTTTGCTACCTTTGGATGGGGTTAAAGTAGTTCTGGACCCATCAATGTGTGTGTGTGCATGCAGCAATGGTTTCTTCCCAATCTTTTTAATTCCATTTTGGCAAACTAAAGTGTGCTTTTTCCATCTATTTCCTGTCCCTTATTGCAATGAACAATTTAGTTAAGGGGTAAACATTATAACTTTGGGACTGATAAAATCAGCACAGAGCAGTTGTAGCTTGCTTTTCACTTCTCAGTTCCTCTGTGAATAGCTTGGTTACTTATGGTGTGGACATTATTCATGATGCCGTTTTCTGTTTTTAAAATCTGTAATTGTATGACATTTGGACAG**ATACCTGTACTGCAACATGTAAACCTTTCAGTGCATCCAGGTCAAGTGGTTGCAATT**GTAAGTGTGTTAGTTCTCTTATACAATATCTTGCTTCAACAGTGATCATTGGAAATTACTTTCAGCTTTGCTTTGATTTTAATTTTCAG**GTTGGTCTCAGTGGCAGTGGGAAAAGCACATTGGTGAATCTTTTGCTTCGTCTTTATGAACCAACTAACGGTCAG**GTAAAATTTTTCGACCTTTGATTGGACCTTAACAACTTCAATTAATGCCTCAGATGGAAGGACTTGATGTCAAGTTAGAAAAAGTTGTTTAGAAAAAATTTGTTACTGTCATTCTAATTTTAATTGTTGATTGATGGCTTCCCTCTTGGAGAGTGCCTGTGGATGCCTACTATTTGAATTTTTCTAAGTATTTGTTGTTTATTGCAG**ATTTTAATTGATGGCTTCCCTCTTAGGGAGTTGGACATCAAGTGGTTCAGGGAAAGAATTGGATATGTGGGACAG**GTCCTTCATTAACTTCTAATATTAATAGGTTTCTGTAAATTCACAGATTTCCTCTCTAAAAAAGAAGAATTTTGAACAG**GAACCAAAACTTTTCCGGATGGATATCAGTTCAAATATTAGATATGGGTGTACAAGTGATATAAGCCAGAAAGATGTTGAGTGGGCTGCCAAGCAGGCATATGCTCATGATTTCATCTCCTCTCTGCCCAATGGTTATGAAACAGTTGTGGATGATGATTTGCTCAGTGGGGGTCAGAAGCAGCGAATTGCCATTGCCCGGGCTATTCTTAGGGACCCTCCAATTTTGATCCTTGACGAAGCTACTAGTGCTCTAGATGCAGAGAGTGAGCACAATATTAAG**GTATCTACATCAGCAAATATTTACATCACCCTTGCTCCTTTTCTCTGCATACAAAAAGGATGGAAAGGGAGGAAAAAAAAAGAAATAATTTAAGTAATATGACTTGCTGCCTTCATTGTGCGATTTGCCACCATGTCTAG**GGTGTTCTTCGAGCCATTGGAAGTGACTTCACGACAAAGAGAACTGTCATAGTAATTGCACACAG**GTAGGTCTCTGCTACCCTCCTTCGGGTATCTGTTGTGTGAGTTGAAATTTGACACGTTCATTTTCTGCAG**GCTTTCTACAATACAAGCTGCTGATAGGATAGTGGTGATGAATGGTGGTCAAATCATTGAG**GTTGGTACTCTTCTCTTTGGAACTCTTAAACATATATTCTATCCTATATTCTGTTGTTGTATTTTCTGCTCTCAAAGCAATACTTGCTCACTCAAAATGCTTGCTCAAATAAGCATCACACTTTGCCCACTTGGTTAAAAGAACCTTGAAGGCTTTTATCCCGCAATATCTATTACAATACAGTGGAGATTTTATTTTTTTCTACACAGAAATTGTTGATGTAGGGCCTCGTGGACCCTGTCCCATTGAAAGTGTAATGCACCTTAGGGGAAGCGTCTGAACATTGAACACCTTAACCCACTTGCAGTTACCTTCAACTGTACCAACTGAGCCAAGTAGCTGGTTAGATAATGGGTTTTGCGTTATGACTTGTAACACTGAAATAGTGTAACAAGTCAACTTTCATAGTTCATATGTTGAATCTGGGCCGATGAATTACTTGTTGAGCATACTTACTAATTTTAGCCTGAATTACTATGAACATAATTTTCTTTTTTCCCCACACAG**ATGGGCAATCACAGGGAACTACTACATCAAGATGGCCTGTACGCACGATTGACTAGAAGACAGGCTGATGCTGTGGCATGATCATATACTGCTTTCACATGTTCGATATTATTTCCTTTAAATCATACTTCATATATCGTATTGGGGCTGAAGGAGCCTTTCAACTGCTGACTCGTGCATTCCTCACATTCTACCAAATATGAAATGAAATGTCTTGGGATGAGAAAGCTGACAACAAAGGAATCACTAAAGCAATTTCTTCATTCTCTATCAGTGTGATGTGGAGTATTCAACGCAGCACAAAACAAGAATGTTCACCTTTTTTCGTTCTTTCTTCCCACCCTAATGCCTGAAATGTAATATTAAAC**

>HbABCB28 scaffold0534(716568-723811)

**GTTGGTGGTCGAGTAATGAAAATTTGCCAAGCAACTTGGGAGTGTGAGACTATATCTTGCTGGGTGTCTCTCGTCCTCCGGTCTACAGCGAGAAAATCTATGGCGTCTGCAACTGGCGTCCTCCTACAAATCAATCCCACTCGCTTCCCAATACCTAAGCTTCACGTGCGTCCCATTAATAAACATAAACTTAGGCAGAGTCAACTGTCGATCTCCTCTGCTTTCTGCCATTTCCCGCCATTTTCACAATCTTATGTGAAGCGATGGAGCACCAAAATCTCCACAATTTCGTGCGCTTACGTGTCGGGGCCTCCAACTTTGAGCGAGCCCGACCCGAAGGTCGACGCATCGGAGTCCACGACTGAGGAGGTGCAGTCAACGAAACTGATAAGTTGGGGACTTTTGTGGAGTCTGTTGCTTAAGCATAAGCTGAGGCTTGGGGTCTCGGTTCTGGCTCTCGTTGGTTGCACTACTTGTACCCTTTCGATGCCCTTATTTTCAG**GTACGACTACGAGTCATGAGAATTCTTCTGGGTTTGGTTCCTGTGAAAATTAAAAGGGAACGAAAGGGGAAGAAATTATTTGAAAATCGTGTTTTAGTTATTATTATTTTCCTGGTATCCATTATTATCGTACTCAGCGGATTAGCTAGCATGTATTGTTCTTAATCAGTTTAGTTGTAATTTAATTATGACTATGAAAAATTATACTTTTGGTCTGTTGATTTATTTTAGTCCTTGTTTGGTGCTAG**GCCGATTTTTTGAAGTGCTTATAGGCGCAAGGCCAGAGCCTTTGTGGAGGCTGCTTAGTAAGGTTGGACTTTTATACTCATTGGAACCAATTTGCACTGTGATCTTTGTTGTGAACATGAACGCTGTTTGGGAGAAGGTAATGTCAAGGCTAAGAGCTCATATTTTCAGAAGAGTTCTAATACAGAAG**GTAAAAGCTGTTTGATGAATTAATTAACTTTTTGAACATGTTAATTGTAGAGTTAAATGCGATTTTCACAAAATTACTTTCTTTTGTTAATGCAG**GTGGAGTTTTTTGATCGATACAAG**GTTTGTATAATTAATTTCGAACTTAGTAAAATTAGTTAGGAACTTGTGTAACAGTACATTATTGTAATGGAATTGAAGGACTTAGGGTTCGTAACTTTATTTGCAG**ATTTGGTGAACTCAGTGCATTGTTAACATTTGATTTGGGGTCTCTTAAAGATATTGTCAATGAGAACATTTCAAGGGATCGTGGATTTAGGGCACTTTCCGAG**GCAAGCCATTCTATACTAGAGTAAAAGTGAGCTTTATGTTACCCAGTCATATTGCTTGTCGTCTCTATTTAGTTGGGCTTCGTACACCTGAAAATATGTTGCCTATTAATTTTTCTACAATAGGGTTGCTTTTCATATTGCCTTTTCTAAAATATAAGCCTCTGCCCATTCTTATTTTTATGAGATTCCCATCTATATTTCTGCTGCCAGAAGCTGCTGGTGTCTTACATATCCTGTATTCTTTGTTTTTCTGCAG**GTAATTGGGACTATATGCATATTATTTGCTCTGGCCCCCCAACTGGCACCAATTCTGGGTATATTGATGCTTAGTGTGTCTGTTTTAGTCG**GTAAGAATTTTGTCTTTGGAATTATCAGTTTATCTAATGCCTTTATTTTCCATCTAATAAAGACTGCACATGATAAATTGTCTCATACATGACGTAGAAGTATGGTTTTTCCTTGATAATTTTATCATTCAAATTTTACTAAATATTTGATATAGCATTGTACATATTCTAGAGCCTGTAAATTTCTGGAGTAGCAAGGATTAGAGTTTATATTTATTATTAATTCTCCTATTGCCGTTGTTTCACAACATTTGTTGACATCAATAATTGCCTAAACTATTGTCTCTTTTGCATTCTACTTCTGTAAATATGGTTCCCCTTGTAAATCTAGATTATGCTTTTGGTTCTTTTTTATCTACTTTTGTTTTGTTACCTAATGCTAACTAGAATTATGCTAACTAGAATTACTATTCTCAAGCCTATTGCCGTTGTTTCACAACATTTGTTGACATCAATAATTGCCTAAACTATTGTCTCTTTTGCATTCTACTTCTGTAAATGTGGTTCCCCTTGTAAATCTAGATTATGCTTCTGGTTCTTTTTTATCTACTTTTGTTTTGTTACCTAATGCTAACTAGAATTATGCTAACTAGAATTACTATTCTCAAG**CCACATACAAGCGGTCAACTATACCTGTTTTCAAAGCTCATGGAATGGCCCAAGCATCCATATCTGATTGTGTGACAGAAACTTTTTCTGCTATTCGCACTGTAAG**TTTTCAAAATGCTTTACCGTAAATGTTCTGAGTAAATTACTAGAAGTCTTCAAATTCGTGCATGATAATTGAAATGGTGATTTTTGTTTCCTTAGGTAAG**ATCCTTCAGTGGTGAAAAGCGCCAAATGTTGATGTTTGGTAGCCAG**GTAAATATGGACCTTGGTTGTGTCTTGAAATATTTTAGTCGCTAGCACTACTGTTTCCACTACATAGCTGTAGTCAGTGTTATGCACTTAGCATTTACATGAATGAGCTTTGCAG**GTTCTTGCTTATCAGGGTAGTGGCATAAAGCTTGGGACCTTCAAATCTGTGAATGAATCATTGACAAGAATAGCTGTTTATATTTCTTTGATGGCTTTGTATTGTCTTGGAGGAAGCAAAGTGAAGGCA**GTAAGTACTTCTACTCTGTAATACACTTGTTCATATCATCCATATTCTTGATTCCTTTTTTAGTGATATTTACAATAGCACTCATAAAAAGGCTATTATCCTGTTATGAGCTTACCAAATTGGCTCTTGGGGGGGAAGAATTGGGAGACAGTTCCACTTTCAGTGATTTGGCCTGTATTTGTTGATGTTTGGATATTAATGTATAATTGGTTTCAAGTAGAGTTAAATGATGAAAAATGATTCATATAGCTAACTCCAACTAGTTTGGGAGTAAGGCTTTGTTGTTGTTTGGAGAAATTCATTCATTTTGTTACATTTCAAGTCTAGAAATTTGTAGAATTACCTTAAAAATGAAAGGGAGCTCATATAATACTCTACTTCGTGAATTTTCTTCAAGATAGATCTTTTTTTGTTGTTTCTTCCTGCCTTTTTTCCTTTTTCATGTTCACTTTGTTGTGCATTTTTTCTAATTATTTGAGCAAGTTTTATAATTTTGAATAACTCTGTAACTATATATAAGACAACAGAAACATGGAGTGACTACTGCCTAAACTTTCAATTCCCTCTCCTCCCCTTCCCTTCACCCTCTTTCCCTTTTTACATTGTTTTTCTGAAGGGGTAAAATAATAAACTATTAACAATGCGTTGAAAAGCTGACTTGTAAAATTGGAGCACCCCCTATGCCCCCATCATCTCTCTTTTCTTCAACTGTCTTCCCCCGATTCTTCTTATGCCAAAGTTATGCAACTCTGTCTGGCTCTCTACAATTCATTCCAAATTGTGCTTGCAG**GGCGAACTCTCTGTTGGAACTGTTGCTTCTTTTATCGGATACACTTTCACATTAACATTTGCT**GTGAGTCCTTTTTCATTCTTTTTGCATTATAATGATTTTTCTCTCTTTAATATGCTTGATAATATAACAAAAACTTTCAG**GTTCAAGGACTAGTCAATACATTTGGAGACCTTCGTGGAACATTTGCTGCTGTTGAGAGAATTAATTCAGTTTTGTCTGAAGTAGAAATTGATGAAGCCCTTGCCAATGGTTTGGAAAGAGAAATCCAGGAAAAAGAAAATCATGATGAGATAACTAAATTGTTCTTTGTTAATGGTTACAGTGGGAAGAATAGATATTTAAATGCACATTACATGTCAGCCTTGAAATCAGCTAGCAACCTGAGCACCTATGCTTGGTCTGGTGATGTTTGTCTTGAAG**GTATCATTAATCCTGTCTTATTTAACTTTCAGATGGATTGATGTAAATTTTACTACATCTTTGCATCTTATGATTTTAGTTATTAAATCATATTGATAATCTTCTCATGTTCTTACAGTATGAGTTAATTGTCTCTTGCGAAACAAAATATAATATTTATTTCAAATTAAGTGTGCAAATCTTATAACTTGATACAGTTTTGTGCTTTATAAGATGCGCTCTCTATGTGGCTGAAACTGTCTTCCCCTACATAAATTTTCTTGGCTGTGTAGTTTTGGGATATATAGAACAAAGAATAAATCCAAATTGCTAATGGTTTCTCTAAATTCATGTACCTTTCGTTTGAATGGAGAAATTTGAAATGTGAAATTTGAATGTCATATTGATAATACATAGATATTGGTTCAATTGATTTTGTCATTTAAAATCCATTTCAAATGAGTTCTCAACCACATTTCATTTTTATAAATTTTTTTTTAAGGCTTCACAGATGGGTGCCACATGGAAGGGGTTGTCAAAAGAGTGGTTTGAAGGAATGGTCTAACTCTTGAGCTTCCACTTTAACTCTAGCTACAAAACAAACAAGGGTTAGAGGGCGAAGAGGGTCTCCTTGCTTGGCACTCCGATGATCAAGTTAGAAAACAGTTTTTCCAGAGGTAGATAAGAGCATTAAGGCGAAATAAATGAGCTCAAGAGTGTTTTTTCTAGAATTGAAGACTTACCTAGTACTAACCGAAATACATACCACACCTGTCCAATAATATAGTGCCACATGTAATTTACATAACGGATAGATTATGTATGGAAGAACCTTATTTTACCAATGTATTGTCAATCAATGTTTACAGTAATAAGACCTTATTAAGAATTAGAGTAGTGCGCTAAAAAGCCTCAATACCAGGATGTGGGGGGTTCAAAGTGTGAAAAGGATAAGGTTGTGTGCGAGAGTCATTTAGCTGTTAAATGCAATGGTGGGTTTATTTAGCTCCATCTTTTAGTAGGTTTATCGCGCTTGATGCTCATATTTTAGGTTTCAAAAGCATTAAATGCAGTAGGTCAGGGTCGTAGATGAGCGATTTCCTTCTAGAGGCCGTTTTCCTCCTTCAAGGGATAGGAGCATGTGTGAAACTACCCATCTGCCACTGGAGGACGTTTTTAAAGGTTTTCCCTCCGTCAGGTATTTTAAATATCAGAAAGAGACACGTGGCGCCATCAAGATGGGTTAGGAATTTGAAGTGTATCACATTTAAACAATTTAAATTAAAATCAAATCCAAATCTTTTACTTCAAATAATTGCATCCAAAAATATTAATGGACCAATATTTTATGCCTACTGATGTCCTCAATTTTTTCTGCAG**ATGTACATTTTTCTTATCCTTTGAGACCTGATGTTGAAATCTTAAATGGTCTTAATTTGAAACTAAAATGTGGAACTGTGACTGCTTTAGTGGGCCCAAGTGGTGCAGGCAAGAGTACCATAGTTCAGCTATTGGCACGCTTTTATGAG**GTTTATTTTCTTTTTAAAATCTTAATTTTCAATTTTGAATTTCTACTTGTTGCTGTTATTATTATTTTTCCATCAAGAAATAGTTGTAATTCGTAAGTGTATCTTTTTCCTACAG**CCGACTAGAGGCAAGATAACTGTTGCAGGAGAGGATGTCCGGACATTTGACAAGACCGAATGGGCTCGAGTTGTCTCCATAGTGAATCAA**GTATATATTTGGATCTTCATACTTCTCAATGTCCACTTAAATGCAGTAATGCAAAACCTGTTAATATGTTTAGCTAGTTGGCCATCAATTACTACTGACATACTGTTTGCTTTAGTTCTAAACCAAGAGGTGGAATACTTGAATACCTAACAAATGAACTTATCTTGAAATTTCATTAGCAAGCGCTGTTTGATATTAACCTGGCATTTGGTTGAGAGAATGGATAACCATTTATTGCCAATTGTAGATACCTTAGATGTCCTAGTTGAATAATCCAAATACCTTTGGATGCTATCTATCCCTGTCTGAAGAATATGTGATGTGTGTGCTGAGGGAGTTACTTCAATTGCCTGATATAAGTCTTCTGTATTTCTCAAACTTAAGCATGTAACATACACAG**GAGCCTGTACTTTTTTCGGTTTCTGTTGGAGAAAATATTGCTTATGGGCTTCCAGATGATAATGTATCCAAGGATGACATCATAAAGGCTGCCAAAGCTGCAAATGCTCATGAATTTATAATTTCACTTCCACAG**GTTCATTATCTTGTAGCTTTGCTTTTGTTGAAAGTTTTGCTTGCTCTCATTACTGTTTGCTGAACCTTTACTTGGATTTCAAAG**GGTTATGACACTCTAGTTGGTGAGCGTGGTGGCTTATTGAGTGGAGGACAGAGGCAGGTATGCAACAAGCTTTTCAGAGAGATTGAGAGATAGGTGATTGGTTTGTGTTTCTGATCTTTAGGTGCTTGTGAAAGAGAGAGGAGGAGGGGGGGCAGGTATTTGAAAGATAAAGGATTGAATTTTATTTTCTGACCTTCATTCTCCAGAGAGTTGCTATTGCGAGAGCCTTGCTCAAAAATGCTCCAATCCTTATTCTTGATGAG**GTAGGCTTCTTGTTTGTTTCTCTATGCATGTGTGATTTCTTCTACCTTGTGTCAACATCATAATATGTTCTTTGTTAAAAGCTCTGTTGAGGCATTAGTTGTTAGAATATTGTTATTATCTTGTTTCAAGCCTGCATAACATATATGTTCTTTACTGCAG**GCCACCAGTGCTTTGGATGCAGTCAGTGAACGCCTGGTCCAGGATGCTCTTAATCATTTGATGAAGGGCAGAACTACATTGGTGATTGCTCATCGATTAAGCACAGTTCAGAATGCCCATCAGATCGCACTTTGCTCGGGTGGGAGGGTTGCAGAACTGGGGACCCATTTTGAGTTGTTGGCGAAGAAGGGTCAATATGCATCATTGGTTGGCACTCAAAGATTGGCATTTGAGTGACATTGAATTGCTATATATTGTCAATAGTTGAACACAGAAGTGCTAAAATTGGTACTTATTTTTCTAATTGAAATTCATATTGCTTCTTAGTTCATACAGCACTACAAGCATAGTTTTGCAATTTTGGTTCTGCTTTCTCTGTGCAGATATGCCAAATGGTTCAATTTCACAAATGGAAATTGGCCTTCAAGTCACAAGAGGAGGTGTGCTGAGTTGAGGGCTTCTTCTTCTTGCTAATTACAGGTCATAATATTTTCACACACACACACAACCCTTAAAAAAAAAAAAAAAAAAAAAAAAAAAAAAAAAAAAAAAAAAAA**

>HbABCB29 scaffold0924(249280-254488)

**CGCTCCAACTCCAATATGAAAGCATTGTCCCTTCAGATTCAACCGAAAACTCCTTTCCCTCGTCTTCCTCTCTTCCACCTCAAACCAAAGCCCCTTTCCTCTCCTCCCATTTCCAGAACCCATAACCCTACTCTCAAACTCCCTTCAAACACCCAACTCAAACCCCTCAATTCCTCCAACCTTCCCTCTCTCCTCCCTTTCACCCAACAAAATCCCAAACCGAATCCAATTTCCCACACATTCCACTCTCTTTCCACAATCAGACCCTATGTTCTTTCCCAGCACAAGCTCATCCTTCTTGGCTGGCTATGCAGCTTCGTCTCTGTTCTCTCCCTTACCAACCTCGTCCCAAAGTTTGGGAAATTCTCCGCAACCATTGGGAAAGTCGATGTCGTTGCACTCAGGAATGAGGGGCTGGTGCTCGCTGCTCTCTTATTGGCCAAGCTGATTGCCACGTACTGGCAGCATGCGCTTTTGTGGGAGGCAGCGCTTAATGCTGGTTATAAGATCAGGGTTCATGCTTTCGAGAGGGTTTTGCATAGGGAATTGGGGTTTTTTGAGGGAGGGAGTGGGGTTTCAACTGGTGATATTGCTTACAGAATTACTGCTGAAGCTGCGGATGTTGCTGACACTTTGTATGCGCTTCTCAAT**GTGAGTAATTTACTTGTGTATATGGATGCAGGAGCAAGTGTTTGTTTTTGGGTTTTTGGAAGGGTTAGGATTATTTTAGGAACAAGCTTATGCCCCCAAATATTGTATGTGTTTATTGACGGCCAAAAACTACAAATCTAATCGTTAAACGGGTATAGAAGATGTTTGTATAAACCCCGTACATAAGTGGAATCTGTTGTTACTAATTGCTATTTTTTTGGACAAGTAATTCAATTTTGGTGATTGTGGCTTGGTTAAAGCTTGTCCCAACAAAGTGAATATGCTTTTGTTCTAACAGTTAATATTCATCACAG**ACCACTGTGCCTAGCGCTCTGCAGTTATCAGCTATGGCAACACAGATGTGGGCTATCAGCCCTGTCCTATCCCTGATTTCAGCCACG**GTAAGGGATTAATTTTGCAGCTACTATGTATGTGCGTGTCTATATATATGATCTTGGGTCTTGAATTTTGCCTGCTTGCTGTAG**GTAATACCATGTATGGCTCTTGTTATTGCATATCTTGGTGAAAGGCTTCGTAAGATATCAAAGAAGGCACATCTTAGCATTGCAACTCTTTCAGCTCACCTGAATGAG**GTTCATTCTATAACCCATTTCATCACCCAAAATCTCTATTTGATATGGTTCTTGGATTGCTTAACTCATACCCTATTCTCAAATACATTCGTTATTAGTGTGAACTTCTTTGTTTGTGTTATTTCTGGTATCCTATTATTTAATATAGGTGGAAATCTCTCACATATGCATGCACAGACACACAACCAGTGGATATACTCTATAAATTTATTATGCATGTTTCTTCTTGTTAACACCTCTTTTGTCAATTCTCGTTGGCCAGTACATTTTTTCATTTGTGTTTTCTATGTTGTAG**GTCCTCCCAGCCATTGTTTTTGTGAAAGCAAACAATGCAGAGCTATGTGAGAGTGCTAGGTTTCAAAGGCTTGCTTATGCTGACCTATCTGAACATCTTAAAAAGAAGAAAATGAAGGCGCTTATCCCCCAGATCATACAGATTATTTATTTTGGAGCATTATTTACTCTTTGTTGTGGATCACTGGTGATTTCGCGTGGTTGTTTTGATGGCTGTAGCATGGTTTCTTTTGTAACATCTTTGGTTTTCTTGATTGAGCCAATCCAG**GTAACATCTATTCAGAAGATTATTCAAAGAGGAATTTATTGTTCTCTTCTTATATGGATTTTTCCAATGACTAGATGAAAAAGTTCATTTATAATTTTTAATAAGATGCTGATAACTTTCTGGTAATTTTTGGTTATGAGTTATTGTGCTCAGTCTATTTTAGATCTGTCTTAAAGTAATATATCTGAATTTGAAGAAATGTGCAG**GATGTTGGAAAAGCATACAATGAGTGGAAGCAAGGGGAACCAGCTATTGAACGGTTGTTTGATTTGATCAGTTTTAAATCTAAG**GTAAATCGCTGCTTTATCAAAGGAAAATTTTCCTTTGAATTTCTGTTTTTCACTTTAATTTTTATAGTTTATTTCTTTTTCCCCTGAAGTAAACTTCCTGCCATCTCCAACTGAATTGAGTGCCTGTTACATACCTGCTTGCATGAGATATTTAAGCCTTCTTTTATGCCTAATTAATTGTGTTATTTGTTTTATTTCGACATCTTTGTGTTTTCAGAAATTTTATGTGGTTGAAGTATATTTAAACTGATGGCTTCTTACTGCAACCAAAAAAAAAAAAAATTGTGTCGGTATGCATAATACTCAAACAATTTTACTCATTATTTTGTGTGTGTGTGTGTGTGTGTGTGTGTGTGTGTGTGTGTGTGTGTTCTTACTTTTGTTTTCGCTTCCGTTTTACGAGTTCTTGAACGTCCATGAAGCTCTCATGCTTTACATGGCCATGCTGATACTGCACATTCTAAAAACTGCATCATGTGTTGTCAAGATACTAATACATTTATTTAGCAGTTGGCAGTTGCTGATGAAATATGTAGTTCAGTTAGTCATTTTTAAATGTACAATAAATGCCGGAACATCTTGGTGCAAGTTGCCACTTTTGTTTATTTATGTTATGGTTGGTCATTATTTCTGTAAGTTATTATTTGTTTGGTTGTGATCTCAAATTAATTCACTCCTTAATATAG**GTGATTGAGAAACCAGATGCTGTTGATCTGGCCAATGTTACAGGAGATATCAAATTTTGTGATATCTCATTTAAGTATGGTAACAATAGGCCTTTTGTTTTGAACTCATTGAACCTTCATATTAAAGCTGGAGAGACAGTAGCTCTGGTTGGCCCATCTGGAGGAGGAAAGACAACCCTTGTTAAATTGCTTCTTCGACTTTATGATCCTTTATCTG**GTGAGCCATAATTTACAATGTCACTCATTTCTAAACCAATATAATCATTTTACCATTTAATGTTTGGCTGTAAAACTCTCTCTTTTCACAGTAGTACAGTATGCAATTGTCTGATCCTCCTATGTCAACAG**GTTGTATACTTGTTGATGACCAAAATATCCAAAACATCCGGTTGGAAAGTTTGAGGAGGCATGTTGGTTTGGTTTCTCAAGATATA**GTGAGCAACTTTTGAACTTTTCTGAATATTTTTTTTCAAATACCCCTTTTTCTTCTCCCAAAAGAGAAAAATAAATTTAAAAAAAAAAAGGATGGGAGCTTTGATGCATGGATTTGAAGTCAATATCTTTTATTCAACATGATGATGCCAATTTTAATGAGAAACAGGTAGTTCTTTTGTTTCAAGTGGGAATTGCTCAGAGTTCCTTTCTTTCTTTGTTTTTTCCTTATCATTTTTAATCTTTCATATGTAAGAAGTAAAAAAAACTTCTGCACCTAGAGTAGCTGGATATTTGTTGTCAATCACACACATGGTGCTGCTGGTTCTGTTGACTTTGAAAGCTTCCAGCTTTGACTTGCAATTTTTGAAGTTCTACTGAAATTTTTTGTGTATTTCGTTGTAATGATAGCTCGGGGTGGTTGGTAATGAAAAATGTTTTTGAAAATGCAGTGGTTGAACACACATGGTAAATTGGTAATTCTTTGAATTAGCTCCAAGTGGTTCATTTAACCAACTTTTTGTTTTTGCAG**TCACTTTTTTCTGGAACGGTTGCTGAAAACATTGGATATAGGGATCTGATGACAGAAATAGACACGGAGAAGGTCGAGGCGGCAGCAAGAATTGCAAATGCTGATGAATTTATTAGAATGCTTCCCAAAGGGTACAAAACATATATTGGACCAAGGGGGTCAAGTTTAAGCGGTGGCCAAAAACAAAG**GTGAAACTTCTAATCCAAACTCTTGATTCTATCTCTGATACATATGAATTGCCAGTTGGTCTAATGCAACCAGTTGACTAGTTGACTTTCAGAATAACGTTATTCCTTTTTTTTTCGTTTACTTAAATACACAG**AGTAGCTATTGCAAGGGCACTCTATCAGGATTCTTCCATACTGATTTTGGATGAGGCAACTTCTGCTCTAGATAGCAGGTCTGAGTTATTAGTGAGGCAAGCTGTGCAGCATTTAATGGAAAATCATACT**GTAAGGATCACTGCTCATTCTATCTGCTTAACATATAGTTTTCTTCCTTGGTACTTCTTCATTGCTGAGGAATAGATGTAGCAACCACTGTAAAGACTCTGATTCAGTTTTGTGGTATACTTTTATGGCAGTATTATCATAAGTAATTAAGTACTAAGCCAAGTCTAAGTTAGATCATGACACACAGATGTCTATGCATGTTGTATCTGCTAACTCCGGTTTTGAGCGATGCTTCTTCCAAAATCAACCCTTCTATATTATAGCCAAAATGACCTGTCCATGCATGTGCAAACATTCTAGTATCAACAATCTGCTACCTACCTACAGTTTGTTTATCTGATGAGAAACTCGTAATCATAATGGATGTCTATAGTGGAATGAAACCAATTCTCTAATGCATCATTTGAAACTTAATCTTGAATTTGAATCCTAGTTGATTTCTAGCGTCTGAGAGGAAGGGGGAGGCATGAAGACAATAGAAACTGTGAATTTCACATTAAAATGTCATTATGTAGTTTATCTGATGAGATCACCATAGCAGTCGTTACATTCAGAGCCATTCATTAGCTTGAATTCGGTGCTGCACCTGACGCAAGGGTAAATTGTTTATTTAAAACCTTAAGCATGATTTTTCATGCTTTGTGTTGAAGCATTAATGTTTTCGCTGCCCTTTTTTTTTTTTTTTGGCTTGAACATGTATTTAACTAGGTTTATATGTGAATAG**GTGCTTGTGATTGCTCATCGGTTGGAAACAGTTATGATGGCTAAACGAGTATTCATTTTAGATGGTGGCAAACTTGAGGAGCTGACTAGCTCAAATCTTTTGGGTGGTCACAGCAATTCTGGGTCATCGACTGGACTTGTGGTTTGAGGGCATGGAAATATGTGGCACGGGCAGTAAAGAGTACAGACATAGATATAAGCTTTTCTTTTTTCTTTTTTTAACTAGATAGGCATAGGCATTTTCAGCATACTGGAAGTTAAAAGTTTTGCTCAACATTTTCAGTTGT**

>HbABCC2 scaffold1385(44136-77992)

**GGGAAAGACAATAATACCCTTTCTGAGCTGCCGATTACTGGAGAGATCCGAGACGCCCGCCTCTGTGAAGTCTCATTTCTGGGCCCTCAATTTAGATACAATCAGTCATTTCTGTTTGGCCTCTTTCTCCAGAGAAACCAGAACTCCTTGTTTCTTGTTTGGTAGTTTTTCTCTGATTTCTCCACGCTAAGACTTTGCTTTTTGATTCCCATTCACACCTCTCATAGCGAATACG**AGGTAATGGAACGGAGACTCTCTTTCTCTAGGTGACCATTTTCAGTTCACCACTTCGCTTTCTCAGCTACAGACATCGTTTCTCTTTTTTCTCTTGGCCTTTTCTTTTTTCCCTTTTTACTTTTCTCGTCTTCTTGTTTATAATCACGATGGTTGTATGTAATTTTATTTATGTATTTTTTAAATTTCTGGGTTATGTTTAATGATATGTTTGTTAGTGATTTTAGCCTTAAATGTAATTAAGGTGAGACGAGAAGAAATTTGTTTTTGTTTTTTGGTTTTGGTTGATGAGAAAAGACTGCTATCTAATCTCAAAGAATGTATCTCTGTTTGTCTGTTTGATCATAATTGTTGGAACTTTTGAGCTCCTTCGTTTTTCCTGCTAAAACTTTCCCAGAAAGCAATTTCTGCTTAACCAATCGGAGTAAATTAATTGGAAGTATGACATCAATTATATTTACGAACTTGCTTGTTATTCAAATAAATAAAGGTAGCTTTCTGTTGTTGC**AGAATTAATTTGCTGTTGATTATGCATTTACGCGGTGAAGAATAGTGGTCGGAAAGTGATAAAAAGATGGCCTTTGAGCCTTTGGTTTGGTATTGCCGGCCAGTGGCTAATGGACTCTGGACAAGGGCAGTGGAAAATGCCTTTGGCGCCTATACACCTTGTGCCACAGACACTCTGGTGGTGGTTATTTCTCACTTGGTTCTCATGGCTCTTTGCTTTTATCGAATATGGCTCACTAAGAAAGATTTCAAGATCCAGCGATTCTGTTTGAGATCGAAACGGTATAACTATTTCTTGGGGCTACTAGCTGGTTATTCTACCGCAGAGCCTTTGTTCAGATTGATCATGGGAATTTCAACTCTGAACATAGATGGGCAGAAGGAACTTGCTCCTTATGAG**GTGGGTTTCTTTTGATTTCATTATTTACTTTCGTTCCATTTTGCATTTCTTTATTAGCAGAGTTAATCTGATTCGTCAGAACTCAGACACCACTAGTCAATGTTTAAATCTGACTTCTTACTTGTCAGCTCAATGCCTCAGTTTATGCATGAAAAAGAAGTAGATGCATGTCAGTCTTCATTTCTCTTTAGAGGAAAGCCCATGTTGTTTGAAATTTTCTTTCTTGAATAAAATTATGCACATTTTCTTGGCTTAATTTTGGGAATGCCAGTTTATATTTTTATCTTTTAAGCAGGCCAGTTTGCTATGCTTGTTTTATTTGTTCCTTGTCATTCAAACTCAACAATTATGCTATTTAGCCATGAGGTCTACACTCTTTCATGCCGTGGTAAGCTAATGATGATTTTATTAGCAG**ATTGTTTCCTTGATTATTGAGGCTCTTGCTTGGTGCTCTGTGCTTGTCATGATCAGTGTGGAAACCAAAGTCTACATTCGTGAATTTCGATGGTTTGTTAGATTTGGGGTTCTTTACACTTTGGTGGGAGATGCAGTAATGTTCAATCTTATTCTTGCAGTGAAAGAGTTCTACAATAG**GTTTGCATTTCTTTAGTTACTATAACTTGCATATTTTGGGCTAAACATGCTATCACTCATCTTTTCCACTTTAAAGCATGATAAATGATTTCTTTCATTCAAGTTCTGAACACATTTTCTTAAAAGGCAAACTCATGTTTGTGTATTGTTTGTGCAG**TTCCGTTCTGTATTTATACATCAGTGAGGTCTTTGTTCAG**GTTTGTTGCTTCTTTCTTCCTTTTTCTTCCTTGAGGTGATTTTTGTTTTATTTCATTCTATTTCTACGGTGCTTTTATTTGTAAGAGTCAGTTAATCTTGGGAGTTAGGCTGGTAATTTGCTCTCATAAGTTGGCCTTTTATACTTGTGATGTGTGTTGTGCGTGTGTATTTATTCAAACTCCATTTTTTTTAATTTATTTTTTTCCACAGCCCTCAGGCCTGTTAAATGGCCTCTGGCCCCACTGACAAAGCGTGCCATATTCCAAAAAGTTGATCTTCTCAATAGTATTTATTGTTGATGGTTTTCAGTTACATGGTGTACAAGACAGAGAATTTAGAGAATTTCTTTCATAATTTTTTATACAAAAATGATTTATGAGAATTCTAATTTATATACAAAGGCTAGGACTAAATAAGAAACTAATAAATACAATGATTCCTAATTATATTCTAATTTATAATTAATATACACTAATCAAGGGATTTACACTAATTAAAGGATTCTAACACTTCTCCTCAAGTTGGTTCATGTATGTTGCACATGCCCAACTTGCAAACTAGGGTATGAAACCCTTTACAACTTAATCCTTTAGTGAACATATCAGCTAACTGGTCTTTCGACCCTCCGTAAGAGATACTTAAGGAATCATTGACAACTTTTTCTTTTATGAAGTGTTTTCAATCTCTATATGCTTGGTCCAATCATGCTAAACTGGATTGTGAACTATACTGATGACAGCTTTGTTATCACAGAACAAGGACAAACCACTAGCTTTCAGCAATTTTAATTCCCCCATCAATTTTCGTAACCACAATCGTTCACAAATACCTTGAGTTATTGCTCTAAACTCATCACAAATACCTTGAGTCATTGCTCTAAACTCATCACAAATACCTTGAGTCATTGCTTTAAACTCATCACAAATACCTTGAGTCATTGCTCTAAACTCAGCCTTTGCACTAGATCTAGCTGTCACATTTTGCTTCTTGCTTCTCCAAGTGACTAGATTACCACCAATAAAAGTACAGTAACCAGATGTTGATCTCCTATCATCAAGAGATCCAGCCCAATCTGTATCTATAAAGGCCTTAATCTGAAGATGACCATACTTTGAGAAAAGAAGTCATTTCCCAAGTGCAGATTTTAAGTATCGTAGGATGCGAAAAACAACCTCCAAATGAGGTTCATGAGGATCATGCACATACTCACTCACTAGACTCACTGCATATGCTATATCTGGTCTGGTATGCGAAAGATAAATCAGTCTGCTAACCAACCTCTGATATCTCCCTATATCCACTGATTCCCCACCTACAGCTTGTAATTTGTGATTTGCCTCAATAGGAGACTCTGCTGGTTTACAACCTAGCATTCCTGTTTTCTCCAACAGATCCAGTATGTACTTTGAGAGATAAAGATTTCTTTATCTGATCTGGCAACCTCTATTCTAAGAAAGTACTTTAGCCTTCCCAAATCTTTGATTTCAAACTCCTGTGCTAGTCGCTCCTTTAAATGAGCCATTTCTTCCCTATCATCACCAGTTGCCACAATATCATCCATATAGACAATAAGCAGAGTGATCTTACCCTTATAGTGTTTTATAAACAATGTGTGATCAACATTGCTTTGACAGTATCCAAAGGAAACCATAACCTTACTAAACCTGTCAAGCTATGCCCTAGGTGACTGTTTTAAGCCATACAGTGCTTTCTTCAATCTATAAACATTTTCTCTGGTCTTCTCATCCTCAAATCTTATAGGAATTTTCATATAAACTTCTTCTTCCAAATCATCATGTAGAACAGTATTTTTTACATCAAACTGTTGTAAATCCCACTCAAGATTTATTGCACATGATAGTAAAATTTTAATGGTATTCATTTTAGCAACAGGAACAAAGATTTCCTGGTAATTTACCCCATACGTCTGTGTGAAGCCTTTTGCTACTAGCCTAGTCTTATACCTTTCAATTGAACCATCTGCTCTATGTTTCACAGTAAACACCCACTTGCATTCAACTGGTTTTTTTTCCAGTGGAAGAGTAACAAGTTCCCATATCTCATTCTTTGCCAGAGCTTTCATCTCCTCCACCATGGTTGCCTTCCATTTTGGATCAAGACATGATTTCTTCCAATCTGTGTAATAGAAATAGAGGAAACAGACAATAGAAAGGGTAAATAGGATGGAGACAAAGAATCATAGGAAATGAAGTTAGAGATGGGATGTTTAGTACAGATTCTGACACCTTTTCTGAGAGCAATAGGAATATCAAGATCATTATCAGAAGAAGTAAAAGTAGACTGAGAATTAAAATTACTCTTCAGGAATCAAAGGAAACTCGTCATATACCTCAGGATGTTCAGGAATTGAATCCAGAGATTCCGATTGATTAGCAGTCTCTTGCTCGATGGCTATATCTATCTTGTTTCGCTGAGTATAGATTCTCAAATTTGATCTATTTAGTCTCTTTCGAGATTTAGGTGACTCCCCTTGAGTATCATTATTATGTATAGGCTTTAAACCCAGATTTTCCCTTTGAAGTTGAAAGTTGGGAGAAGACAAAGAATATGGGAAAGATACCTCTTTTTCCTTCCTATGCTCCCTCTGAAGAGGTGGATGGAAATAAGATTCAGGTTCCCTAAAGGTAGCATCCATGCTCATAAAATATTTCCGTGTAGGAGGGTGGTAACACCTATACGCCTTCTGAGTACTGGAATAACCAACAAAGACACACTTGAGGGCTCGAGGTTCTAACTTTCCCCTATCAGGCTGATGAATAAAGCAAACACAACTAAAGACCTTTGGAGGAATAATATATTAATTTTTACCTTGGAAAACCTCTAAAGAACTCTTAAACTCTAAAGTCCGGAGTGACATCCTGTTAATAAGATATGCAACAGAAAGAATAGCATCTCCCCAGTAAGGTTTGGGAATATTCATTGTAAACATAAGAGACCTAGCAACTTTAAGTGAGTTTCTATTTTTTCTCTCAGACACTCCATTTTGAGCACTAGTGTTAACACAACTAGTCTGATAGAAAATTCCAAGTGACTTCAAATATTCCTAAAAACTCCATTCATATACTCTGTGCCATTATCAGTTCTCAATATTTTAACATGAGTATCAAACTGGGTGCTAATCATTTTGTAAAACTGCTAAAAATATGAGAATACTTCATTTTTCCCTTTCATCAGATATACCCAAGTTAATCTACTGCAACAATCAATAAAGGTTACAAACCATCTATAACCAGATAAAGACACAGTTTGAGTAGGCCCCCCTACACATCAGAATGGATAGTCATAAAAGGAATTGAAGCCTTATTATTTATTGCAGGATAAGATTGTCTAGTATGTTTGGCAAACTCACAAGCATCACATGCTAACAATTCAGTTTTGCATTGCTTAAACAAAAGAGGATAAAGTTTCTCTAAAACAGTAAATGAAGGATGTCCAAGTCTCCTATATCACTGTATAATTTCTTTATCAGCACTCATAGACTGTCCCAACATGGCCTGATCAACAAAATCATTCAATAAATATAGCCCATCTTGCAGTCTACCATTGCCAATCGTTCTCCTTGTTATCAATTCCTGAAATACACAGTGAGTGGGAAAAAATTCAATTTTGCAGTTGAGAGCTTTTGTGATAGAACTGACAGAAAGAAGATTAATAGGAAAGCTAGGCACATGTAAGACAGAATTAAAACTTATAGTAGAAGTGCATTTAATAGAACTTGTTCCAGAAATATTAGATAAGGATCCATCTGCAGTGCGAACTTTTTCTTTGCCAGAACATGGAGATAGGATATGAATTTATTCGAGGAGCCTGTTGTATGTTTGTTTGCTCCAGAATCTATAATTCAAAATAAATTATTATGATTGGCAAGAAAAGCATTACCTGAGTTGAGGAAGTTAGAAGAGGCAACTGTAGTGGATTGTGATTCAAGCTGTGACAGGAATCGCCTTAGAGTCTGTACTTCTTCATTGGAAAACATCCCAGTGATAGCAGTATCTTCAGAAACACTCACAGCCTCATATACATTTGCTTGTGGTCTAGTGGAGCCTATCCTTTTTCCTCCATGCCCTTTAGTTGGGTGGCTATGCAGCTTTCAACATTGTTCTTTGGTGTGCCGGGATTTCCCACAGTAGTCACAATGCAAGTGATCCTTATCTGATGGACTATGTGACTGTTCTCGTAAGAAGTTAGCAGTCAGCCTAGCCTTATCAACAGTTGTAGAATTAATCATGACATTCCTTCTGCTTTTCTCCTGCTGAGCATAAAAATATGTCTGCTTTAAGGTGGGCAAATGATTCTTATCAAGTACTTGGACCCGAATCTGATCATACTCCACATTTAGCACAACAAGAAAATCATATATGTGTTCCTTCTCAATCAATTTCTGAAACTTAACTGCATCAACCGGACATGAAGCCTGAAAGTCCTGATAGAAATCCAACTCTTGCCATAGACCACTCAATTCCGCATAATGCTGAGCAACGGTCAACTCACCTTATTTCGTTCCATGAACCTTGTTCCTAAGCTCATAAACTTGTGCATCATTCCTAATTTGAGAATAAGTCTGAGAAACAGCACTCCAAATGGTAGCTGCACTGTCTAACAATAAATACGTACGAGCTAGATGAGGTTGCATAGAATTGATGAGCCATGACATAATAAGAGAGTTCTCCGACTCCCACTGATTGTTGGTAGAACTAGCACTTTTTGGCTTTTTCTTATCTCAAGTGGTATACCCTTGCAGTTCTCTAGCTTGAATGAACTGTAGACAGGATCTAGACCATTCCAAATAATTAGTGTCATCCAACTTTACAGGACCAATTTGTAAAGAGGGATTATCACCGACAAATCCAGTCTTTGTTTCAGAGGTTTTCTTCTTTCCATCGATCATTTTTCAATTAAAGGAATAGATACTGAGGCAAAAAGCAAGAAACTGGACAACGGAAGGTACCAAAAAATAAATGTGCAAACCAGGTGTGGAAACACGTTGGTCAAAGGCGAGCCAGACAGGGAAGGTCGTCGGCAAGAGGCACGGCCGGAGAAATCACCGGAAAAAGGGTGCACGAGCCGGCGCGTGAAGAATGACGTGAGGCTTCTGGAGGTGCGTGAGCTCACGCGCCTCACCGAACGGCGGCGGGACTCCGGGCGTAGGCCGATCGGCTGGTGTCCTTCCTCCTCAGGTGGGTGGTGACAGTCACCTCCCTTCTTAGAACAACATAGAAGCTTGACCCACAAAAAAAACTACTCAAAACTACACTGCTCACGCAAAAAAATTTTGGCACAGCTCTGATACCATGTAGAAGACAAAGAATTTAGAGAATTTTTTTTATATTTTTTCATACAAAAATGATTTACAAGAATTCTAATTTATATACAAAAGCTAGGACTAAATAGGAAACTAATAAATATAATGATTCTTAATTATATTCTAATTTACAGCTAATTTACATTAATTAAGGGATTTACACTAATTAAGGGATTCTAATACATGGTTATCCAATTTCTGTTGGTTGGAAGTCATTGTAGCCCAGTTTTGCTAGGGAATAGGATCATGTTGGATGTTAAAAGTGGAACTTGTCCTCTAGAGCTGAAATTGTTTTCCTCTAATAAAATTGGGCTTATCTTTAGTGCTTAGGTTTATTGACACAGATTGATACTGCTGAATTAATGGCCTAACATGATTAGAATCGCCAAGGGCGTTGCACAGTTTATATTAATTCCGCTGGCCAAACATGACCAGATATTGCCTCTGAACAGGATCTGGAATAGAGTTTTTTTTTTATATATATTTGTTGAAAAGATTTAAACACTCTGTTTTTCATCTTATTTATATTAAAAAAATTACAATTTAGTCCTGATATAATTGTTGTATTTATTTAG**GTTTTATTTGGAATACTTCTGCTTGTGTACGTTCCTGACTTGGATTCTTACCCTGATTACACTCCCTTACGGTCTGAATATGTTGATGATGTTGACTATCAAGAACTTCCTGGAGGAGAGTACGTATGTCCTGAGCAGCATGTCAACATATTTTCCA**GTATGTATACTCTAATATATTTGTCCTAAATATCTGGTAGCTGGAAACTTTCAAATTGCCTAGCATGCTGACTGATGTATAAATCATTATTTTTAAGATGAGAAAATAATAATTATGTTAACTTTATTATGACTGGTCAACCAATTAATCATTTTAGCTTCCTAATTGTTTTGCATTCCATTTTTCTTCCGCTAG**AAACTATTTTTGCATGGATGAATCCTATAATGAAGCTAGGATACAAAAGGCCTCTCACTGAGAAGGATATTTGGAAACTAGACATGTGGGATCGGACTGAAACACTGAACGACAGGTT**TGTGTGCTTGTTTTACCTTACATTTGTTTCTAATATAGTTTTGTGCAATCAAAGTTTGTGTGATACGTTTTGCTGACCCTATAGGTT**CCAGAAATGTTGGGCTGAGGAATCACGAAGGCCTAATCCATGGCTTTTAAGAGCATTAAATAGTAGCCTCGGAGGAAG**GTAAAACTTGTGACAATCTTTTAAAGTAATTTTTTTTCTTATGCCTGTACGGTTCAGTTATATGAGTAAATCTCATATACGTTTGTTGCTGTCATGGTTAACATTTTATTTATGGTTTTGAAAGAGAAGAGGTGGAGTTGGAGATAAATAACCAAAGTGCATAATGTATAATATTCAAAATTAATAAAGTTCTATTTAGGAGATATTACTTGGAGAACATATGATAGGGTCAAACGTAAAACTTGCTGACAATCTTGATTACTATTATGATGTTTATGTTTTCTTTAGCTGCTATTTATTTGTTAATTTATGTTTTCTTTAGCTGCTATTTATTTGTTAATGCAATAGCAATAGTTAGTTTGCAATAGGACTCTCATTGCAATAGGACTCTCACTATGAACTCAAACTTAGTCCAGTCTGGGTACAGACTCAGCAAATGCTGATTGGTTCTTTTGTGATTATGTTGATGTTTTTGTGTTTATTTTGTTTCTGATGAGTAGTAATAGGAGTGGTTTAATGAGTATGTATGTGTTATTAAGGAATCATTCTACTATTATCAATATTCTTGTATCTTTGTGTGTTAATAAGCAATTGAGCGAGATTTCAACTTTTTTGCTGCTCATTCATTAGTGAGGAACTGTATTGTTCTGCAGTTGACTAGGCAACATTTATTTGGTCTGTCTTTTGCAAAATCATCTCTGGGTCCAGCCTGGTTTGACCTGTATTGACCTAGTGCTGTTGATTTACTCTTCCTTGACTAGTGGATGTGTTGATGAGTTGATTTTCAGTTCTGGGATTGATTTTTGCAATGGCATGATTTTACGGAATTAATCTGAGATATAAAAAGATGCTAACTTGAACAAATGCTGTGCACATTCCTGCAGAAATTCTATGCTTCTATCTGTTATATTAAGTTTGATAAATGTTTTTCAATTCTCCCATCTGTCTAGAAGTGTGCTTGTTTGCGATTACATGGACATGTATCCGACTGATGTTCCCTTTTTGCTTTTTGTAG**GTTTTGGTGGGGAGGCTTTTGGAAG**GTAATGTGCTTATCTTTCTAAAATCAAATTTAGATTTTATTTTGGTTATTGAACTGGAAGTATCATATTTATTAATGGACACATGATGTTATGAGAATTGATTTAGGCCTATATTGATCAGAGTGGATTTTAATATGTCCAAGCTTAAAAAATTGGTGTTTTTTAGTATCTTCTATGAGAAGTGATTCCCGTTAAATCATTATGGCATTACTGAAATGTATTATATTTAGAAGAGTTTGAATTGTACAATGATTATAACAGAAAACACATAATCGACAAGAGTTAAATCTGCATAACATATTTTATATGAACATAATCTCTGTTATCTTCTTCTTTTCTTTTTCTTAGGTCCAAACTGAAGTTCAATTTATGCAATACTTTTTTTATAAAAATAATAATTTATCTGCATTAAACTTATTATACCTTGAAAATGGTATTTTTACAATTCAATTCTTTGAGAAAAATCTCCTTATTTTTATTATCTTATATATGTTGATTTCACATGATTTATTTTTTTTTCTTCTAGTGAACTTATGTGAAATGTGTTGGCTTTTAAGGATATATTGCTGTAGTATTACCATCAATAAGCTTTACATATCTGATATATCTGTTCCTATTTTTTTAACTCATAG**ATTGGCAATGATGTTTCCCAATTTGTGGGGCCACTTCTATTGAACCAACTCCTACAG**GTTAGCTACTCTTTTGTCCTTGTTTTTCTGGATAAATGATTAACCTTCTTTCTTTTTTTTTGCCCCATAAGGTTGAACTTTAGATTTCCATGGTCTGGTTTTGAAGTTAATATTTACTTTATTTTTAATTATTTATTTTTTTGGTGGATAAAATTGGTTGAAGGTGGCATGCAATTATCTTTTATTTTAGTACATGACATTGATTAAAAATATTTATCAATAATGCTTATATGATCCTTTAATGTTCTTTTGATGCGAAGAACAAATAATAGCAAATGCAAATTTGTTGTTCTAATCTTGTATATTTCTATCTCCATGTCATACTTTATCACATCATGTGCCAGTTCATTTATAATTGGTAAGATTTATGGTATTTCATAGTATCACTTTGCTTGTTCTTATAGATTAAGGATTGGATATTTTATGGTGGAGATCTGATACCTGATTCATTTTTAAATTGTGAAGATTTTTTTCTTTTTAGGTTGAGGGGGTGTGAGCATGGATTAGATGTTTACATTTGGCTGATATAAACTGTGTGGTTCTTTGACTAAATGTTTTCCTGGACTTTTTTTTAATTGAAGTTTTCTGAGACTATTTCACTTACTTTTTTTGGTGGACTACATGCAG**TCTATGCAAGAAGGTGATCCAGCGTGGATTGGATACATCTATGCTTTCTCAATTTTTGCTGGGGTG**GTATGATATATATCCTCTATCCATCTAAGTGTTAAGCTTGCCTTCTGATTTAGCTTCTTATGTGATGTCTTTTTGACAACATGAAATGCTGCATTTTGTTGTGCCTTGTGTATTGATTATTTGAGTTCCATGCCTTCCTGAGGAAAATGGTTAGAGGGGTGACCATGTAACTATTATTCGTCTATCAGAAGCAAAATGATGAAATAGTATAATAAATGCCTTATTTTAAAAATGTTCTGCGTGGTGATTCTGGCTCTTGTGCATTGGGCATTTGCAGTTAACCGATGCCCCATTGTTGTTTATTTTGTTCTATGTTTTTCGTAGAAACTAGTTTTGTTAGTTTAGGCTGTTCCTTCTACTTGCAGGTCCTTCTTCAAGTGCACTGTGCACTTAATTTCATAATTGAATAATATATAAGCACTTGGGATTTCTTACTTGCTGTAATATTTGTCAG**GTATTGGGTGTGTTATTTGAAGCTCAGTATTTTCAGAATGTCATGCGTATTGGTTACCGGCTGAGATCAACTTTG**GTAACATCGCCTTGTGGGCCTTTTTTTTTTGTATTCAGTTTCTATTGTGATTTTGTGAATCAGCTTTACTAAAATAAATTTGCCATTGAAGAATTACCTACTAGAAAATTGAATTAATTTTACTACACAGCAGTATAAAAACAAATGCCCATGAGTGTCACTATGTAAACATAAAAGATAAAACACAGAAATTGGGGGATAAGATATTATGTGGCCTCAGGAATCATTCTTATTCTTATTGAAACCTAAAATCAGCATTCTTTTTTTTTTTAAAAAAAATATTTCTATAATGCTGAACTCAAGGGGTAATTGGCTTTGGGTAAAATAGCAAAATGTTTTATGCATTATTATTTTGTTGGGAGGAAGAGGCAACCTCAGTGGGTTTGTTGTTGGTTTAAGGGAGGAAATTGGAAGTTATCTGAATGGTTTAGTGGTAAATTCAAATGTTGAACTTAAATGTTCTGCATCTCTCATGCTTTTCAG**ATTGCTGCTGTATTTCGGAAATCTCTAAGGCTAACTCATGAGAGTCGGCGAAAGTTTGCATCTGGAAAAATAACGAACTTGATGACCACTGATGCTGAAGCACTTCAG**GCATGTTCTTTCATTTCAATCTTTTATACAAATTGTTGTGTTCTTGATTGCTTATCTCACTGAATGTGTTTTCTACTCTGTAAAATAGGTGAAGTGTGTAGGCTAGATTTATATACTGATTGCTCTAAAAAGATTCAAAACAGTGTGCAATGTGCCATTTGTGTTTTATGAGCTCATGTGTGCAAACACTCGGTTGGCATGTATCCATGAAGAAAAATGCCATCACCAATTATAACTCCTGCAAAGCATGGTGCTCGGAAGTAATTCTGGAGAGTCCTTTCTTTCAGAATTTTGTATGAATTGATTGTTTATAGCCCCACATAACCACCACACATTCATAATGTATTTCCTAGTTCGTACACCATTTTGGTATTGTAACCATATTAGAATTATTGTAATTATTCTAGGTCAATATACCTTTAATAGAAATAATAGCTTGGGCTTGCACCTTCTGTATGATCATGATTTTCTGTTTACTAGAATTGAGAAATGAATAGAAACACCAATATAATTGCCTTTCCTGCTATTTTCTAAAAACTATCAAGATGTTACTTTAATGAAGATGCATTATATGAGCTTCCTTTCTGTGTTGCTCTTTTGCAAATATAATGGGAGATCCAAGGACTGAGCCTTGTATAAATATGTAACTAGATCATGTTATGCCACCTTAACTTCCAAGTATTGTTTCTGACTAGATGTGGTTTTCATTTTTATGAAAATTTCATATTCTGTTTAGTTAACTTGGTTATTATTAATGACTGATTGCTGATTTCTATCAACAG**CAAGTATGCCAATCCCTTCACACTCTGTGGTCAGCTCCGTTTCGTATTATCATTGCAATGGTTTTGCTTTTTCAGCAGTTGGGCGTTGCATCACTTCTTGGTGCAGTAATACTAGTACTCCTCTTTCCCATACAG**GTACATGAAAGTTCCTTCTCTTTTTGATCATTTGATCTATAGTAATTTTTGCTAGATGTTGTTTTCTCATTTTTTTTCGCTTGGTCAACTTTCATTTTCATTCTCAAATATGTGATTTTTTTAGAATCAATTTTTTTTTTTAAATAATTTTTCTCTGTTTAATAAAAGGAATGTTCTACCTTTCAGCTTTCACAGATTTCATTTTCCTGAGTACTTTACAGTTTGATGAATTTTCAGTTTTTCCTTTTTCATTCATCTCCTTCCAGAAATTTTTGCTTCTATTAAACTGAGTAGTGGAATCAAGTGGGGGTTATTTTGATCTGTTTCTCGTATGTTATTTTTATTATTTTCCCTAGGTTCAACTTATGTATAGTTGCATCTGATTTGTGAATCTGTTAATCTAATTGCTTATGAGAAAAGGGTCATCAAATTTTTTGAAGAACTAGAGTCTAAGAAAAAAAATGATGAAATATGATTTGAAGTAATACTAATACATCAAAAGAAAAAGTGAAGATTTGTGATGAGCAGAAACCTGCTTTGAATTTTCTATAACTTTGTTTGAACTTTTGTGAGAAGGGTGAGGAAGTATGAATATGATGAATGAATAAAGGCATATTTGAATCTTTGTAAATGCTCCAAAAAGAAGATTTCAGTATTCTATAACATTACCTGGTGCACATTATATTAGTGCAAACTGGGCTTTACATGCTATTCAGTATAGACAGTGGATAGAAATCCAGGTTTTTCATATCATCCCTTTCTAGCTTTCTCTACATTATTTTGGCATTGTGTGAAGGCTCAATGAAGTGATTTTCAAGAGTAGTATTAACAAATTGAAATCTGGTTTTGCTTTTGAGGAACGTACATCATCAGAAGAGCAAAGAAGGTTTTGAATAAAATTTTTAATCGATATATTGTATAATTGTACTTTTTGTTCATGTTGAGGTAGTCAAAATAGTTAGTTGTAGTCCCTATAGAAGGATATCACATGATAAGAAAAATTGAAAATGCAAGAAGCTTGAAAGAACTTATCCAAGAAGTTAATTAGCATTGAGAGTTGCAAAGAAGTTTGTAACCTTCAAATTAAAGCACTAATGACGTCATGTTTTAAAAAGCTCAATAATGAAGTTTTTCTATCATTCGACCAAAATTTCACCAGCTTGCAATAATGTGCTTTTGGAGGACTCTTTTTAGTTTTTCCTGTTTCCTTATGTGTGTTCATATGCATTCATGCGTACAAAGAGTTTGAATTGATCAAAAGTTTCATGGTTCCCATATTACACAGTTGAGGTGCAAAAACTATTGGAATGTCCTCCAAATGAACTTTAACTCTAAGCGACTGACACATTGAATATTAAAAAAAAAAATTGAAATTAAAAATGATTTGTTATTTTTTCATAAAATATTTAAATGAGTACTTTGCTGTGACATGCTCATGTTTATATGTAAAACTTATAAGGGATTCATCCTTTGAAAGGATTTATAAGTAAGCCTATAGTTAATAGCTGCAAGGATCTGATTATACAACTGCAAACTAAATATTATGAGGGATTGTATTTCATTGCATTATGGTTATTTTGAAAATGGAAAAGGACTGTATAATCCAACTGGTGTAATTAAAATAGCAGGATCAAAGGTTTATGTTTATATGGGTGTTCTCCAAAGGATGCACTTTTAGCCTTACCATTTAATGTAAATCACATTTTTGGTTCAGTATGGTGAAAATTTTTTACGGCCTTTTCTTCTCTCTCTCTGTATGTGTGTGTGTGTATTTGTTTTGTGCATTATCAAAATGTCTTAGCATATGTATCTGGACTCTTGAAAAAAAAAAATAAAGTAAAAGATTACACAGGCTTCCAAAATTAAAAGAATGCTCTAAATGACTTTTGTCATTTTTCCTGTAACTAACCAAATAGCATTTTGAACATTTAAAGTCCGATTTAAGGTAGTGCAAATAGCTTATATCAAGCCCATCCAATCAGGTTGCTTTTTGCATGTGGAACTAGAACTCCAGATAGAGGGTAGGTTTTCGGATGTAATTAGAACTAGCCTATGTCAAACTCATCCAATAAGGTTTCATATGCTGCAAGTTATTAATTTGTTGATGTACTTGTACGTTGTTTATTCCTTTAATTAGATTGTCCCTAATTCTTGCTCTCTCATGCATGTTGGAACAG**ACATTTGTTATAAGCAGAATGCAGAAATTGTCAAAGGAGGGCTTGCAGCGCACTGACAAGAGGATTGGCCTTATGAATGAAATTTTGGCTGCAATGGACGCTGTGAA**GTATGATACATTTTTTAAAGTAATTTCCTTTATTTTCTTAAAGGGTATCTACTGATTTTTTTTACCATGTTGAAATGGCATAG**GTGTTATGCATGGGAGAACAGTTTCCAGGCTAAAGTTCAAACTGTTCGTGATGATGAATTGTCATGGTTCCGAAAAGCATCACTACTGGGAGCG**GTATGGCCTGGAACATATTTGATGGAGATTCCCTTTTTTTTTCACCTATAGTTTAGAAAGTCTAAAATATTGATGATATCATGCAGTTTTTTGAGTTTTAACAGCTATTTTGGTAATCTTGTTTAGTAATCTTGAATTAAAGCTCAAATAAACTCAAGTGAGCTTGAGTTCATAAATTATTATTTAGATAAAATATAATATATATTTAAAAATTATGTATAAAAATAAATAGTCAAATAAGCTTGCCAGCCAGTCAAGCTAAACATTGTAATACTTGAACTTGGTTTGTCTTGCTACTCAAGTTTGAGCTCAACTCAACTAAAAACCAATTTGAATTTGAATATTTTGCAAGCCAAGTCTGAGTCGTGCACGAGTAACTCGACTTATTTGCAGCTCTACTTCTGTTGCAGCCTATAATTCAGGCTGAAACTCGCATGTCTAGAAACTGAAGGCTCAGGCTTAGGAAAACTTAATTGATTAATGATTTTTTTGAGGATAATTTGTTTATACCTGAAACAAATACATTGCTTGAATCTGAATGTTGGCCAATTGATGTTATACACGGATGTTTAGTGCGAGGACTATTAAGTTTTATGGTTTTTGTTTCTTGAGATGATGCATATTCTTTGCTCATTGCTTGCTTTGCAG**TGTAATGGTTTTATACTAAACAGCATCCCTGTGGTGGTCACTGTGATTTCATTTGGTATGTTCACTTTGCTTGGGGGAGATCTGACACCTGCAAGGGCATTTACATCACTTTCCCTGTTTGCCGTGCTTCGCTTTCCCCTCTTTATGCTTCCTAATATTATTACCCAG**GTTTGTTTACTTGCCTCTTTATTCTATATCTTGCATCTTGCTTACTGCTGAGATACTGCATGCAATATTATTGTGGAAAGAAAATGCAAAAACAATTGGGATTATTTTCCTGTTCTAATTGGTTTCCCTGCTGCAGCTTGTCTGCTAATTTTAATAGAATATTTAGTTTAGATTGTTTTGATTAATTTAATCACTAAATTTGACTATCAATTTTGATTTTGAAATGGAACATGGAACAGTCAAAATAACTTGATTCAGAGCTGATATATTTGAAGGTGAACTTGAAATGAATAAAATAAAAATGTCTATTAGAAATATTTTTTCAAGTACACTGCACATGAAATTTACCATTTTTAATAATGTTAGAGTCTCATGAATTTAAAAAACAATACTTTTTGTTGTAGAATAGGATCCTCGTCTGTTGTTTTCTTTCTTCCAGTATTATTTCTTATTTTCTTTTTTATAATAAATTTTCATTTCTCTGCGTGATTGATATACGTTATATGCTTGCATGTCTCCAAGTTGCTTGTGTTGTTAGGTTATACTGATGTTGTAATTGGACAG**GTAGTGAATGCAAATGTATCCCTAAAGCGTTTGGAGGAACTACTTCTGGCTGAAGAGAGGATCCTTCTACCAAACCCTCCTCTTGAGACAGGACAACCCGCCATCTCAATAAAAAATGGATACTTCTCTTGGGATTCAAAG**GTTCCATTCATCAACTTTGTTACTTTAGGTCACATGTGCATTTGTGTTTCAACTGTTTGTGATTATTCATGCAATTACTTTCTAGTACATCAATTATTACTTTTTAGTACATCAATTATGTCTGAGACTCTGAGGTGAACTTTAATATAAATGATAGTTTATTTGTTCAAATATCTATGCAAAATTTTAAACCATATCTTTTACCCTTTGCTCAATGCTGATTGATGAAACTTTATTAGATGAATGCTTCAAGATGAATGAACTGAAGCGACCACAATAGCATAGAAAAATTTTGACTTGCGTGCTGTTGTGCCCATATTCTGTAACATGTATAAAAATGAACATATTCCTTCTCAGTAATAGATAATATACTCATTCAGACATAAAAATGCACACATCCCTGGACCTGAGACATGTAATCTTTTGAGTAGCAAGGGAAATTCAACGTAAATGAAGTGTCTTGTTGTTACAACTGCATTTGAGTTATTGAATGAAGAGAATTTTTTCTGTGTATTTTCTTGAATGAGATACAAGGTATTTATATATAAAGAATCTTATAATTAAGTATTCTAATTGGGAAGAGACCCAATAATTATGCTAACTAATTAATAAAGAATATTATGTACATAATTATAAGATTGACTTTTTATAACACTCCCCCTCAAGTTGGAGCATAGATATTAATCATGCCCAACTTGTTACAAATATAGTCAACCTAAGCCCTATTCAAAACTTTTGTGAAGATATTTCCTAATTGCTCCCCGGTTTTGACATGCCTTGTCGAGATGATCTTCTGTTGAATCTTTTCTCGAAGGAAATGACAATCAATTTCAATATGCTTAGTACGCTTATGAAACACTGGATTTGAGGCGATATGAAGAGCGGCTTGATTGTCACACCATAGTTTGGTAGGAGATGAGGTCTCAAGATCCATTTCTTCCAATAACTGACGTGTCCACATAACTTCACACACAGCTTGGGCCATAGCTTGATATTCTGATTCAACACTCGAACGGGAAACTACAGTCTGTTTCTTACTTCTCCAAGATAGTAAGTTTCCTCCAACAAAGATACAATAACCTGTAATGGATCTTCTATCAATCTTTGAACCTGCCCAATCTGCATTTGAAAAGCGCTCAATATTTAAGTGCCCATGATTGTTATATAAGAGACCACGACCTGGAGCACCCTTCAAGTAACATAAAGTTTGCTCCAAAGCTTTCCAATGGGTAACAGTAGGAGAGGACATACATTGACTTACATTACTAACTGAATATGCAATATCTGAACGAGTTACTGTAAGATAATTAAGTTTGCCAACCAATTTTCTATACATCTCAGGGTTTTCAAATAGTTCACTATCTCCTGCTGTGAGCTGAAGATTGGGAGTCATTGGTGCACTACATGGTTTGGCACCTAATTTACCTGTTTCCACCAATAAATCAAGGACATACTTCCTCTGAGATAAGAAGATGCCTTTCTTACTCCTTGAAACTTCAATGCCTAAAAAATATTTCAATAAACTCAAATCTTTTGTTTGAAACTGAGTCTGAAGGAATGACTTGAGTGAAGAAATGCCTGCATAGTCGTTTCCAGTAATGACAATATCATCAACATATACTATAAGTAGGATTAGTCCAGCTTTAGAATGTCTAAAAAACACCGAATGATCAGACTTATTCTTTTGCATACCAAACTGCTGAACTACCTCACTGAACCTGCCAAACCAAGCTCGAGGACTCTGTTTCAGGCCATAAAGGGGTTTTCGAAGTCTACAAACTTTGCCTAACTCCCCCCGAGCAACAAATCCTAGTGGTTGCTCCATATAAACTTTCTTCTGGAGATCACCATGAAGGAAAGCATTGTTGATATCCAACTGATGTAAAGGCCAATCATATCTAGCTGCCAAGGAAACAAATAAGCGAACAGAAGCTAGTTTAGCAACAAGTGAAAAAGTTTTAGAGTAGTCAGTCCCATAAGTCTGAGCATATCCTTTAACTACAAGGTGAACGTTGAGACGAGCCACAGTACGATCGAGGTTCATTTTCACTGTAAAAGTCCATTTACATCCAATAGCTTTCTTATCTGGAGGCAGATGCATCAATTTCCAAGTACCATTAGTATTTAAAGCCACCTTTTCCTCTTCCATTGCGGCACGCCAGCCAGAATGGGACAATGCCTTAGCAACAGTTTTAGGAATGGAAATAGTGTCTAGAGAACTAATAAAACAACGAGATGAAGGAGACACTTGATCATAACATATAAAGGAAGAGTTAGGATAAGTACATTGACGCTTACCTTTGCGAAGAGCAATGGGAAGATCTAAGTTAGACTGAGAAGCAGTAGATGAAACTGGATCTTCTGACGAAGAAGCTGGTGGAGGATCTGAGTCGAGTCTCCAATCTCCTGGAATAAACATGAACAACAGGTGGGCGATGAGGTCGAGCAGGTGCTGGTGCAGGAGGATTCTAAGGACTTGGCTGAGGACTTGACACTGGATGGACAAAATAGACTAAGAGATCATCTTCCTCCTCTTGACTCTCATAACTAGGAGAGGGAGGAAAGAATGGAGTAGATTCAAAAAAGGTAACATCAGCAGACACTATAACGATTAAGGGTTGGAGAAAAACATTTGTACCCTTTTTGAAGTCGAGAATAGCCAAGGAAGACACACTTAAGAGACTTGGGATCCAGTTTAGTAACCTGTGGACAAACATCTCTCACAAAACACGTACTACCAAAGATACGAGGTTCAACAGGAAACAAAGATTTAGAAGGAAACAAAATGTTATAAGAAATGTCACCATTAAGCACAGATGATGGCATACGATTAATTAGGAAACAAGCTATAGAAACTGCATCATCCCAAAATTGTTTAGGGACCTTCATTTGAAAAAGGAGTGCCCGTGCTACCTCAAGAAGATGTCTATTTTTTCTTTCAGCAACCCCATTATGGATGGAATATCAACACAAGATGTTTGATGAAGATGTCCTTTTGTGACATATAGGCCTAAAAATTGTCAAAAATATATTCCTTAGCATTATCACTTCTCAATATACGCATAGAAGTTTTAAATTGAATTTGAATCTCAGCACAAAAGGTACAAAAAATAGAAAACAATTCTGAACGTTCTTCATTAAAAACAACCAAGTAACACGGGAATAATCATCAACAAATGTAACAAAATACTTGAAACTAGTTTTAGAAGTAATAGAACAAGGACCCCAAATATCAGAATGAACCAACTCAAAAGGGGACGAAGCCTGTTTATTAACTCTAGGTGTAGAAGGTGAACGATGGTGCTTAGCAAACTGACATGACTCACACTCTAAGATGGACAAAGACTAAAATTGAGGACATAATTTCTTCAAGGTAGCCTAGGAGGGATGGCCCAAACGGCAATGAATTTCGAGCGGAGTTAAGGTGCTGGAGCAAGTAAGAGATTGCGGTACATATTTATTCAGGATGTAGAGACCACCGGACTCATATCCTCTATCAATAATCTGCTTCGTCCTAAGATCCTGAAATAAACAATAATCAGGAAAAAAGGAAACCGAACAATGTAAGGCACGAGTAAGTTTACTAACCGAAAGCAAATTAAATGAAAATTTAGGTAGACACAAAACAGATGATAAAGAAATAGATGATGTTGGGTTTGCAGTATCAGAACCCATGACACAAGAAGTAAAACCATCAGCTAAAGTAACTGTAGAGGAGGTAGGATGAGACTGAAAAGATGATAGAATACTAGAATTACCTGTCATATGATCTGTAGCACCAGAATCAATGACCCATTTGGATGAGGAAGATACAAGGCATGTAGTGGATTTACCTGACTCAGCGATAGCAGTGACAGGGGAATAAGAGGCTTTTAAAGATGCATGATATTGGGAAAATTGTGCATATTCCTCTGCAGATATTAAGACATTTTTCTCAGATGAGGGTGCAACTGTAGACTCATCTATTGCCATGTTTGCCATCTAAGACTGCTGATTCTTCCTCTGAAGCTTTAGACAATTGTATTTTGTATGACTAGGCTTATGACAATAATAACAAATAACTCTTTTTGAGTCCAGATTAGAATTGGTTTCCCCATTACGCTGATTACTTCTATTGCTTGAATTTCCTCCTTTATTTCTCTGTTGTCCATTCATATTTTGACTAACAAGAGCACCACTAGTAGTTTTTGGAAGGTGGGTACTTTTTGTATGAAGGACACGTGTGAATGTATCATGTAGAGAGGAAATTTCAGAACTAGATAGGATTTGAGATTTAACAGTCTCATATTTTGAAGGAAGACCTGCAAGAAAACTGATAACAGCCATCTGCTCTCGTTGAGCCTGTTGGACTTTCATATCAGGGTTAAAAGGCAACAAGACATTAAGTTCTTCATATACTCTCTTAAATTCTATAAAATAAGCCGCGAGAGACTTATCCTGTTTCTCAGCACGATAGAATGCTTTGCACACATCATAAATACGAGAAATATTTCCTTTACCAGAATACAGGAAATCTAAATAATCCATCAATTCCTTAACAAATTCACAGTAATTAGTTAAACTAATTACCTCATTATGAATCGAATTTCAAAGTTGCAAAAACAACCCCGCATCTTCCCTAAGCCAAGCTTGCTTTGTATCATCAGTGGGTGGATCTTGAGTCAGATGATCATCCTTGTCAATACTTCGTAAATAGACCCTGACGGTCTTACTCCACTCTAGGTAATTTGAATAATTAAGTTTGTGTTATGTGATCTTAGTCATCATTGGAATCACATCAGAAATAATAGCCTTATTGTCCGCCATTAGATGAAATTAACAAAATTCTAACACAGATTAATAACCAACTCTGTCCCAATCTTCAAACAAACTTAATAAAACTTAGAAACAAAAGAATTCCGTGAAAACAGAACCTGAAATTGCTCAACAGTGAACTGCCCGAAGGAGAGAAATATTTGAGATCCATAAGAAATGCCCCAAACTGAACAATGAACCGTGGAACTAGAATCACAAAGTTGAGGCGACCCTGGGATAAGAGGCGTGACTGTCGGACGTCACCGGAGGAGGGCACACGCGCGTCCAAGCGCCGGCGCGTGGAATAGGGATTAGAACTCAAGAATGGGGCATGAAGGCTACGCGCTTCAAGTCCGGTCACCGGACTTCCAAGGGTGGCCGATCGGCACCTAGGCCTTCTCTTGAGATGGGTGGTGAGACACTGTGATAACTCTATTGAGTTCTCCTAGAAGACAGCACTTGTAGGAGGAGAAGAACCAGAGAAAAAATATATATATATTTCAAAAAAAAAAAATTTTGGGCTAGGGAACCTAGGCTTTGATACCATGGAGAATATTTTCTGTGTATTTTCTTGAATGAGATACAAGGTATTTATATATAAAGAATCTTATAATTAAGTATTCTAATTGGGAAGAGATCCCAATAATTATGCTAACTAATTAATAAAGAATATTATGTACATAATTATAGGATTGACTTTCTATAACATTGAAGCAATTGAAGAATAGTAGTACTGAATCATTACTCTGTAGTCATTACTACTTTAGCAATGCTACACAAGCTGGCAACAATATTTCTAATTATGGACAATTTAATTTTTTTTTACTTCAG**GCAGAGAGGCCCACGTTATCAAATGTCAACGTGGATATACCTATTGGTAGCCTAGTGGCAATTGTTGGTAGTACTGGAGAGGGAAAGACCTCATTAATTTCAGCAATGCTTGGGGAGCTTCCTGCGATATCAGATGCAAGTGCTGTTATTAGGGGAACAGTTGCTTATGTTCCACAAGTTTCATGGATTTTTAATGCTACT**GTAAGTTATGGTTTTCTTATGCTTTGTAATATATAAATTATTTTGTTAAGTAATTTATCCCAAAAAAAAAAAAGGAAAAGAGTGGATGGTTGTATTTATGTTTGTGTGTGAGTTCAATTCACTCCTATGTTAATGTTCGTGGCTTTTGGGATAATTGTAAATTTCATATTGCTGATTGCAATGATGTGAAAGCCTTTAAGTTACTCTTTATTGGATTTAAATAAGTTCCGTAATTGAAATTACTAGTAGTTGACTTGTGACTAAATTGCTTGGTACAACTTTAAGTGACTGCTGGAGTCTATTATTGCAG**GTACGAGACAACATACTTTTTGGATCTCCCTTTGATTCAGCAAGATATGAGAAGGCAATAGATGTGACGTCATTGCAGCATGACCTTGACTTATTGCCT**GTAAGTTGTTCGAGAAAGTTATCATATGTAGCAGTACTTGTGGATATGCAAATATCAAATCTTTTAAGAAGTTCATTCCAGAATTTCTTCTCTCTCATGTTCTTGATTGCTAG**GGTGGTGATCTCACTGAGATTGGCGAAAGAGGGGTGAATATTAGTGGTGGGCAAAAACAAAGAGTTTCCATGGCTAGGGCTGTGTATTCCAATTCAGATGTATACATTTTTGATGATCCCTTAAGTGCTCTCGATGCTCATGTAGCTCGACAG**GTAAGATAGTCTGCATTATTACTCGAAACACAATTGTTATTTTATTATTGTAGCATCTATAGTTTCTGTTTGTGGTTGTGACAATTAAGATTCTTGGTTGGGTCTTTAAGATCACAGATAGGATCAAATAATAGGACAGACTTGACTGCTCTACTTGATATGAAACAAAAAACGTTGTCCACACATTGTCATGTATAGTTTTTTGCAAACTTAGTAGAGGGCTATTAATGGACAAGGCAGTTCCCAACCATCTCCAGCCAAAGTAAGGTAGCATTTTGAGACATTCCACTCTTTCAAAATTATGCAAGAATGGCTGCTTACAGCCTCCTCTTTGTTTCTTACCCAAGATTCTTACCATTGCAATTTGGTTTGGTTTGGTACTTTTTATATTGCTATTGGACAAGAAATATACATTTTTTCTTGTAATGACTTTAATTTGTAAATTCCTGTACTTTGAAGAGCTTGTATACAATGAGTGTTATTTTGCTTTGATAGTGCCTCAGGGCATTGCTTCTCTCATGTCTTGAAACGAGTTCTTCTTAAAACTTCCTTTTAACATATGTTGTTTATTTTCATATTTTCTTCTCTTTGCTATCCATTGCACTCAGGGTCTCAAAAAAAGTCATGTTGTCTTGTATTGGCCAGAGTGCTAAGGAAAAATGGCTGGCTCATATAGGGATTGGGTGTTATCTGTTTGGTTTATTTGGGAACACTGGGGTTTTGCCTATAATGGTTTTGATTGCACAAAGCATTTGACACACACCATTATGTGAGAGAAATAAGCATGTCAAACAGAAAAAAAAAAATGGAGGAAAAGAAGAGGAGGGTTGAGGCGGAGAGATAGTGTCTCTTTCTTTCCATCGTCTTTTCCTTTTTTTTTTATTTTTTTTATTTTGACACTATTTCTAAAAGCCTTAGCTCTTATATGGGCTTTCATAAACAATGCAAGTGCTTCTTGTAAAGCCTATTTAGGCTAAGTTTGACCTTTTATAGTTTTGTAGGTGTTTTTATTTAGAGAGACAGAGAGAGTTCTACAAAATTGTTTTACAATTACTTTTTTCTTTCTTTAGAATTCCTTTTTTTTCCCCTCCATTTTGCACTGTAGACTGTAGTCTACTTTTAAACGTTGTTATTTCCATTTAAAAAAAGTTACCCCTTTTCATCAATTGCAGCTAGAAAATTATAGTATATTGTTGATGTTTTCTTAATATGTATATGCTTGGTTAGGTACATGCTAAACTATGAATATGATTTTTTTTTTTTTTTTAAATTATGGTTACACAGGTTGATAAGATTGTAATACTGTGACAGTCAATCTTGACTGACACCATTAATGAAGCCAAGGTCCCCAGGCTCAAATGATTAACAAATGTTAACCTTCCATAAATTATAAAAGAACTTTAATATGTGGTACTTATTTTAGCTGTGATTGACTGTGTTCATGATGAAGCCATTACAATTTTATTTTTATTTTTATTTTTATTTTCTGGACTAATGTTCACTATTATCTTTAG**GTTTTTGATAAATGTATCAAGGGAGAATTGAGTGCGAAAACGAGAGTTCTTGTCACAAACCAGCTCCATTTTCTTTCTCAAGTTGATAGAATTATGCTGGTCCATGAGGGTATGGTGAAAGAGGAGGGAACCTTTGAGGAGCTCTCCAACAATGGCATGCTGTTCCAAAAGCTGATGGAAAATGCAGGAAAAATGGAAGAGTATGTGGAAGAAAAGGAAAATGGTGAAACTTTTGATCTTAAAACCTCTTCAAAACCTGTTGCTAATGGGGCAATGAATGACTTGCGTAAGAATGCAACGGAGACCAAAAAACGAAAGGAAGGCAAGTCTATTCTTATCAAGCAGGAAGAACGGGAAACAGGTGTTGTCAGTTGGAATGTTTTGATGAG**GTAATAAAATGTTTACTGTAGTGCTTCAGTTATCTATCGGTAAAATGCAATGTATCTCTTTTACATAAAATATGCTTTGAGGAAACAAACCGGACATCCCCAGCAATAAATAAAGTGGAACCTTCATGCTAATTTGAGACCAGATTGTCTCTTACCTCATTTATGAATTGAGTATCTCAATTTAGAATACGAGTTTCATTGTTTCAGCCATTATAAACTCCCCTTGTCCTATATTTGAAATTAGAATTTCATTGCCTTTTAAGTGTTGGTGATTGTTACAGGATGAAAAGTTCTTGTGCGAACCTTTTGTTTGGAACTAGAATTTGATGGTAATGGGCATTGATGATGGTCCCTGCTGTGTTCTGTCTTGCCAAATGAATGCTGAAATTGATTTTCTTAGCTGCTTATTGTTTCTTCCTTTGATAACCCATACATTTTATCCTTTTTAATTTCTTGTTGGTCATCTACTTTGTCAGGAAAGATTGGAAAGCTTATTGTGTGGCAATAATCATATATTTATATTCGCTCAACATTGTGTACATGTTGAATCACTGATTAAGAGAGGGATCTTAGAGATTAATTGATGTGTTGGAGAGGAATGTTTTAATTACCTATTGATGTGAATACTCTGGTGATTTCATTACAGTATTTATAACAGTATAATGACGAATAATGGAATTCATTGAGGAGCACCACAAAGACAGTGCAAACCAAATGCAGATAAGAATGTGTAAAAGATAATCATGTTGGCTTAGCAGCCACAGACAATTCAGAAATTTCGTTGTAGAAGGAAAGAGTACGAAGTTAAGAATGATAGTAATAAACACTAAACATAACATATAATGCAGAAATTTGTGCAAGTGAGAAAGGGAAAATTAAAAGAGAAAGAGGGAGAGGAGGGGAGGAGGGGGGCATCCAGAGGCTGCTTGCTGATAGGAGTTTCTGTTTTGAGAACATCAATTTTAGTGATCCTTATTTGACATGTGATGTAATTATCTGTTAGTTTCAATTTATACAGTTTTTATCTTTGTTTTTTCTCCTTAAATTGTAGAGAGTATGATAATAATCTCTTTCCAG**GTACAAGAATGCATTAGGAGGTGCCTGGGTGGTATTGATACTCTTTATGTGCTATGTCCTTACAGAAGTTTTGCGAGTTTCCAGTAGTACTTGGTTAAGTAATTGGACAGATCAAGGCACTACAAAGATCCATGGGCCCCTTTACTACAATCTTATATACTCCCTTCTATCATTTGGTCAG**GTATGAATCAACTGGATGATAACTGAATGCAAGTTCTACTGCAGATATATCTATTTACTCATTGTTTATCCGTCATTCTGTCCAGTCTAATTTAGCCGCTAAGGGGCCTTCATTGCCTACTAAGTGAAAGAATCTTTACCATCCAAAATAAATAATTTTTGTTTTAATAACTGATTCCTCTTTCCTTACTCAATTTGCAG**GTTATGGTGACATTATTAAATTCATATTGGTTGATCATTTCAAGTCTTTATGCTGCCAGAAGGTTGCATGATGCTATGCTTAATTCCATATTGAGAGCTCCAATGGTCTTCTTTCACACCAATCCACTTGGACGTATTATCAATAGGTTTGCAAAGGATCTTGGTGACATTGATAGGAATGTTGCCCCATTTGTGAATATGTTTCTGGGTCAAGTGTCACAGCTCCTTTCTACATTTATCCTCATTGGAATTGTGAGCACTATGTCATTGTGGGCCATAATGCCGCTTTTAGTTCTGTTTTATGGAGCCTATCTATATTATCAG**GTATGTGAAACTTCTTATCGTTTGTTATACATGTTTTCATCCGACATGTAAATATGGTGCTGTTATTTTTTTCAGTTATCTCCCTTTCCCCTCAATTATCTACTATTTTTATTTATATGATAAGTGGCTACTCATGGTAGATTGGGAAATTGAATGTTGTGGGTTTTATCCAATGTAGTTCTTGCTAAGCATTTAAATTATTGAGATTATTTTGGCCTTATAGATGTTAAAGCGGACTGTAATTGAATAGACACTCAAGTAATTTAACGTAATTATGCACTATAATGTCTTGATCTTTAGTAAATTCCAAGATCTTGTTCAACTGTAGGTAAATTGTTGTTGGATGGAATTTGATGGGTGTGCAATATCATTTTTAACATGAGATTTATATGTGTGTGGCAAAGCCTTACCGCAACAATTTGGTTTGGCCGGCTTTAAAGGCCACTGTATTTTATGGCTTTTATGCTATGTTCTTCCCTTCTGGTTCTATTAGGCTTCCTTTTTGGCTTGTAAGGCTTCAATATGAATAAATACCACTTAGGCTGCATCTTAGTCTGAATTTCTTCATGTCGTCCTTAGTTATTAATTGACCTGAGTGCTCATAAATGCTATTGCTTGTAATTGTCTCTACCTGCATAGCATTTTGGTTAAAATCAAGCTATAACAGTAACTTTCTAGATTTGTGGCAATGATTGTATTCTCTTAGAGAACATTTTTGATGCTGTTAGGAATGGCAAAACCCGATCAGCCCCAAAAGCCCCACCCTGCCAATGGTAATGGCACAGAGATGGGATTAAGCTGTCCCAATCCCACATTGATAACTTAATAATATATGAAAGTTTAGGAACAATTCAAGTAAGGTCAGAAGAAATAGTGTAGCTCGAACAAAGTGGTGGGAGTTCAAAGGAGTAAAGCAAGTGAAGTTCAAAAATGAGCTTCTCGAGTCCGAAGTATGGAAGCTAGATATGGAGGCCAATGATATGTGGATACAGATGGCATCAAAGATTAGAGAAGTAGCTAGAAAAGTACTTGGAGAGTCTAAAGGACATGGACCACCCTCAAAAGAGAGATGGTGGTGGAATGAGGAAGTACAAAAGGCAGTGAAGAGAAAAAGGGAATGGTATAAGAAATTACCTAAATGTGATAATAATGAGGCATGTGAACAGTACAAGATAGCAAAAAAAGAGGCAAAAAAGGCAGTTAGCCAAGCAAGAGCACAGGCCTTTGAAAAGTTATATGAGAAACTTGGAACTAAAGAAGGGGAGAAAGATATTTATAGATTAGCAAGGAGTAGAGAAAGGAAATGTCAAGATCTCAATCAAGTTAGGTGCATTAAGGATAAAGAAGGAAAAGTGTTGGTGAAAGATGAGGACATTAAAGAAAGATGGAGAAATTATTTTAATGATCTCTTTAATAATAGTCAAAATGGAAATAGCGTGAATATAGATTATAGAACAATAGAAAAGAATGTAAATTATACTAGAAGGATTAGATCTTTAGAAGTAAAGGAAGCACTTAAGAGAATGAAAGTAGGTAAAGCCTGTGGACCCGATGAAATACCAATTGAAGTGTGGAAGTATTTGGGAGATATGGGAGTGGCATGGTTAACTAAATTATTTAATAAGATTCTAAACTCAAAGAAAATGCCTGATGAATGGAGGAAGAGTATTTTAGTACCTATTTTTAAAAATAAGGGAGACATACAGAGTTGCTCAAACTATAGGGGAATTAAACTCATGAGCCATACTATGAAGTTGTGGGAGAGAGTTGTGGAGCATCGACTACGTCATGATACTTCTATCTCTCTCAATCAATTTGGTTTCATGCCTGGTCGTTCAACTATGGAAGCGATCTTTCTCATTAGAAGCTTGATGGAGAAATATAGAGATGGGAAGAAAGATCTACACATGGTTTTTATTGATTTGGAGAAGGCTTATGATAGTGTTCCAAGAGAGGTCTTATGGAATGCGTTAGAACAAAAGAGGGTATCTATTAGGTACATACAAGTATTGAAAGATATGTATGAAGGAGCAACTACTATTGTGCGCACAGTGGGAGGGGACACAAGAGATTTTCCGATCTCAATTGGATTACACCAAGGATCAGCCATAAGCCCTTACCTTTTTACATTAGTTTTAGATGAACTGACGAAACATATACAAGAGAGTATTCCTTGGTGCATGATGTTTGCGGATGATATTGTTCTGATAGATGAGACACGAGAAGGAGTCAATAGGAAGCTAGAACTTTGGAGAAGTACTCTAGAGTCAAAGGGTTTTAAGTTAAGTAGAACGAAGACAGAATACATGCATTGCAAGTTCAGTGAAGGCCAAACTGGTGATAGGGAAGGAGTTAGTTTGAATGGAGTGGCACTGTCCCAAAGTAATCACTTTAAATATCTAGGCTCAGTCCTTCAAGTAGATGGGGGATGTGAGGAGGATGTTAGTCATAGGATTAAAGCCGGATGGTTGAAGTGGAGACGTGCCACGGGAGTTTTATGTGATCGTAAGATTCCCAATAAATTAAAAGGAAAATTTTACCGTACAGCCATACGACCGGCTATGCTATATGGTAGTGAGTGTTGGGCACTGAAAGAGTCGTATGCATCTAAGATAAGAGTTGCAGAGATGAGAATGTTAAGGTGGATGAGTGGTCATACTAGACTAGATAAAGTCCGTAATGAAAGTATTAGAGAAAAGGTAGGAGTGGTGCCAATTGAAGATAAGTTGAGAGAAGGGAGATTGAGGTGGTTTGGTCATGTGAAGCGTAGACATACGGAGGCTCCAGTTAGACAAGTAGAGCACATTAGGCTAGAGGATAGAAAGAAAAAAAGGGGTAGACCTAAATTGACTTGGAGGAGAGTAGTACAGCATGACTTAGAAGCATTACACATTTTTGAGGATTTAACCCAAAATCGTTCAGAGTGGAAAAAGCGAATCCATATAGCCGACCCCAAATTTTTGGGATAAAGGCTTAGTTGAGTTGAGTTGAGTTGTACTATTTTATGATAATATATAGAAAAGTACATATTTTTAATTGTTATAGAATTAAAAAAAAATTAAAAATGTGGAATCCGCCCCAGCCCTAATAATTTTTTGCCCCTCTCTATACCTACAGGCACAAAACCTGGACTCACCCTGCCCCATTGCCACCCCTAGATGTACCCTGAAATAGCTTTTATAGTATCATTCTTTATTACTGCAACTGTATATAGGATAAAGCAACAAATATTTTTAGGTTGCAGAGCCATTTTGGCAGATAAATGCTTTCTATTTGTAATCACTCGTTTATTTTTGAGAGTCAGTTTCATTTAAAAACTACTTTGTTCCATAG**AGCACAGCCCGCGAAGTAAAGCGCTTGGATTCTATAAGTAGATCACCTGTTTATGCACAATTTGGAGAAGCTCTAAACGGTCTGTCAACTATTCGTGCATACAAAGCTTATGATCGGATGGCCGACATCAATGGTAGATCTATGGACAACAGTATCAGATTCACCCTTGTCAACATGAGTGCAAATCGTTGGCTTGCTATCCGTCTGGAAACATTGGGAGGCATTATGATTTGGCTTACAGCAACCTTTGCTGTGATGCAGAATGGAAGGGCAGAGAACCAACAGGCATTTGCATCTACAATGGGTCTACTTCTCAGCTATGCCTTAAATATTACTGGTTTACTGACCGGTGTACTAAGACTTGCAAGTTTGGCTGAGAATAGTTTAAATGCTGTAGAGCGGATTGGCACATATATAGATTTGCCATCAGAGGCCCCTCCAATTATTGAGGGCAACCGCCCCCCTCCAGGTTGGCCTTCATCAGGATCAATCAAGTTTGAGGGTGTTGTCCTGCGTTACAGACCTGAACTTCCACCTGTCTTACATGGACTGTCCTTTATGGTTTCTCCAAGTGACAAAGTTGGGATAGTTGGAAGGACTGGAGCAGGCAAATCTAGCATGCTCAACACTTTATTTCGAATTGTGGAACTAGAAAGAGGAAGAATCTTGATTGACGGTTGTGATATTGCAAAGTTTGGTTTGATGGATCTACGCAAAGTACTTGGTATTATACCACAATCACCGGTTCTTTTTTCAG**GTACACTCTCTCTATAATCTTAGTTTCAATCATGATCATAAGACTATGATTAGTTAACTTCCATTTTTTTTTATTTTGTCTAG**GAACTGTGAGGTTTAATCTTGATCCTTTTAATGAACACAATGATGCTGATCTTTGGGAGGCTTTGGAGAGAGCACACCTGAAGGATGTTATTAGAAGGAATTCTTTAGGTTTGAATGCTGAG**GTACATGCTCAAAATTTCTTTTGTTAGATTTCTATTATTTTGAGTTAGTGAGGCAAACAATTCTGCTTTATGAAATTTCTGTGCAAGGGTTTATTTTAACATCATCAAACTTATCCAATGCAGGAGGGGGTTCCATGTTATAAAGCTTGTCATATAGACCTCTTGAACCATATTACTATGAATTATTCTGGATTAAAAATATTTACTCTTAAATAGTTTTCTGTTGCCATCACAGCTCTTCTGTCTAACTTGAACAGTTGAGAAACTTTTTCCTAAAAAGAGAAAGAAAGAAAAAAAAGGGAAAGAAAAAAAAAAAAAAAAACCTCTTTTTGGGGATTGGGGGTGGGGGGAGGATTTGCATGCATATCTGCTTCAGAAGTTAGCAAAACTTGGCCAAAGTTGAGCCAAAAGATCTTGATAGATTCATGAGGAGTGACACCACATTTATTGTTGTGCTATCATTAACCTTACAGATTCTTTTCTCTATTCTATGGCAATTATCTGTTGTGGTCATAGATTTAGGTGGCTTGTATGGGAGCCACATCCCACTGACTTCCATGGTGTGGTAAAAATTGATCCTACACTGCACTGAGTTTCACATTTACAGCTGGGGATTGACTGATACCATTGTTATGAAATGAATGGTGGTTTTGATTTTTTAAGTAGATGTGCCTCTACCTAACTTCTAAAGTTAAACAATGGAGTTTTGATTTTGAAGAACAGTTAGAACAAGGAACACGTCATAATTGGTGTCTTGTTGTGATGTGTTCTTGGCATTTTAGTAGTGAACTGATCTATAGTAACTCTGTTTTGAATTGTTACTACTGCCACTTCCACTACTACTATTATACTACTGCTTTGAACTATAATCTTGCTCACAATTTGTCAAG**GTTTCGGAAGCTGGGGAGAATTTCAGTGTTGGACAGAGACAACTTTTGAGTCTTGCTCGAGCATTGTTGCGGAGATCAAAGATACTTGTTCTTGATGAAGCTACTGCTGCTGTTGATGTGAGAACAGACGCTCTTATTCAGAAAACCATTCGAGAAGAATTCAAATCATGTACAATGCTAATCATTGCTCATCGACTTAATACGATCATTGACTGTGACCGGATCCTTCTGCTTGATTCTGGCCAG**GTATGCCTCCAATTTGAAGCTTTATTCTGGCTAGGCGTGCATGAATATCTGTAGGTCCGCAGATCCTGCCCCGGCATGCACTGGCAGATTTCTTATCTGATGGAAAATTTTCATCTTCTATCTCTATGGTGTTTGTTTATTCACTGAATGTGTCATGAATCTTCTTCAG**GTACTGGAATATGATACACCAGAGGAACTGTTATCAAATGAAGATAGTGCTTTCTCTAAGATGGTTCAAAGTACAGGGGCTGCAAATGCTCAGTACTTGCGCAGTTTAGTACTAGGAGGGGAAGGAGAGAGTAGGTTTAGAACACGAGAGAACAAACAGTTAGATGGGCAGAGGAAATGGCTGGCCTCTTCTCGTTGGGCTGCTGCTGCACAGTTTGCCATTGCTGTTAGCCTTACTTCATCACATAATGACCTTCAGCGGTTGGAAGTTGCGGATGAGGACAGTATCCTGAAGAAAACAAAGGATGCAGTGGTAACTTTGCAAGGAGTGTTGGAAGGGAAGCATGACAAAGTAATTGATGAATCATTAAATCAATACCAAATTTCCAGAGAGGGCTGGTGGTCAGCTCTTTACAAGATGGTTGAAG**GTACAAAATGATTGCCCCTTGTAATACATGAGCCCCATATAACCTTCAATGATTGCAGACATTCATATTTGTTTGTATTTTATGAATTTATCTTTTTTATCAATTGATGCATACTTCTCTCTAATATGTATTTTTCTACTGTAAATCTGCAAATCAATATCCTGATCACTCAGGTTGAAAAATGGATATCACATCACTTGTACCATTCAGTTGCTAGTGTAACTACAATATAATCTGTTTACTGATAGTGGGCGTGATGAACGTTTTTGGATATCATTCTTTGAAGCAGCAGCAGACGCTGCACTGGCATTTGTGTTCCATCTCGTAGTGGGGAGCAACTTAGCATTTGTCTCATATTTTAGTTCTTCCTTCATTTATGCATGGTCTCTTTTTATTCATATTTTTTTCCTTTGCAG**GTCTTGCCATGATGAGCAGACTAGGTCGAAACAGACTTCACCAATCAGAAGGCTTTGAAGATAGATCAATTGACTGGGACCATGTTGAAATGTAGGAAGCAGTATTTCATAAGATTTCAATAACAGGGTTTATAACTTTGCTTCAAATTGTATCCACATGAGGAAGTAACTTACCAGGCACCTGATATTTTTGTACGTAAGTCTCTGGAATTCTGTCCATATGCTTCAGATTGTCATTTCAGGACAATCCTTTTTGTCCATTTTTACATTTGACTCAGAATCAGGGGTGGTACCCTTAAGTGGGTTCCAGGTATATTCAG**

>HbABCC5 scaffold0097(1869496-1879433)

**ATAATGGGTATCACCCTTTTGCTTAATAACATCGTCACGCAATCGACACACCCCGTTTTGAAGGCAATTCGGGGCCTGCCCGTATTGGAGCTAGCCTCAATTTGCATCAATTTGACGCTTTTCCTTGTCTTTCTCTTCATAATATCTGCAAGGCAAATATTTGTGTGTGTTGGTAGAATTAGATTGCTTAAGGATGATACAGCAGTTGCCAGTTCAAGCCCAATTAGACGAAGTACTGTTGATGGAGATATTCGAGTTGTTACGATCGGTACCGGGTTTAAGTTGGTCCTATTTTGTTGTTTCTATGTTTTGTTTTTGCAATTTTTAGTGTTGGGGTTTGATGGGGTTTCTTTGATTAGAGAGGCTGTTAATGGGAAAGTTGTGGATTGGTCTATAATTGCTTTCCCTGCTGCGCAAGGTTTAGCTTGGTTTGTGTTGAGCTTTTCAGCTCTTCATTGCAAATTTAAGGCGTCTGAGAAATTCCCACTGTTGTTGAGGGTTTGGTGGTTTTTCTCATTTTTTATGTGTTTGTGTACTTTGTATGTGGATGGGAAGAGTTTTCTGATTGAAGGTGTGAAGCATTTGAGTTCTCATGTTATGGTGAATTTTGCTACAACTCCAGCTCTTGCTTTTCTATGTTTTGTGGCAATTAGAGGCATTACTGGAATACAAGTTTGTAGGAATTCTGATCTTCAAGAGCCACTGCTTCTTGAAGAAGAATCAGGGTGTCTCAAGGTTACTCCTTATAGTAATGCTGGATTGTTTAGCTTGGCTACTCTTTCATGGTTAAACCCACTTCTGTCTATTGGTGCAAAGAGACCGCTTGAGCTCAAGGACATTCCTCTTCTTGCTCCAAAAGATCGAGCTAAGTCCAATTATAAGGTTTTGAATTTGAATTGGGAGAAATTGAAGGCAGAAAATCCTTCAAAGCAGCCTTCTTTAGCTTGGGCAATTCTCAATTCATTCTGGAAGGACGCGGCTTGCAATGCCATATTTGCCTTGGTAAATACACTTGTTTCATATGTAGGTCCGTATATGATGAGCTACTTTGTTGAATATTTAGGGGGGAAGGAGACTTTCCCTCATGAAGGATATATTCTTGCTGGGATATTCTTCTCGGCAAAGCTTGTGGAGACCTTAACAACCCGGCAATGGTATCTTGGTGTTGATATTTTGGGTATGCATGTGAGGTCGGCTCTGACAGCAATGGTGTACCATAAGGGACTCAGGCTCTCAAGCTTGGCCAAGCAAAGTCACACTAGTGGAGAAATTGTTAATTACATGGCAGTTGATGTCCAGAGAGTGGGGGACTACTCTTGGTATCTCCATGACATATGGATGCTTCCCTTGCAAATAATTCTTGCACTTGCAATTTTGTATAAGAATGTTGGAATTGCTTCTATTGCAACTTTAATTTCCACTATCATATCCATCATTGTTACTGTGCCCTTGGCTAAGATACAAGAAGATTATCAAGACAAATTAATGGCTGCAAAGGATGACAGGATGAGGAAAACTTCTGAGTGTCTGAGGAATATGAGGATTCTGAAGCTGCAAGCTTGGGAGGACAGGTATCGAGTGAAGTTGGAGGAGATGCGGGATGTGGAGTTCAGGTGGCTCCGTAAGGCCCTCTACTCACAAGCTTTTATTACATTCATTTTCTGGAGTTCTCCCATATTTGTTGCAGTTGTTACTTTTGGTACTTCAATATTGTTGGGTGGTCAGCTCACTGCTGGAGGTGTTCTTTCTGCTCTGGCCACCTTCAGAATCCTACAAGAACCACTTAGGAATTTCCCTGACTTAGTGTCAATGATGGCCCAGACAAAAGTTTCCCTTGATCGAATTTCTGGATTCCTACAGGAAGAAGAGTTGCAGGCAGATGCAACCCTTGTTTTGCCACGAGGTATGACAAACATGGCCATAGAAGTTAATGATGGTGAGTTCTGTTGGGACCCTTCTTCTTCTTCGAGGACCACTTTATCAGGCATTCATGTGAAGGTGCAGAGAGGGATGCGTGTAGCTGTTTGTGGCATGGTTGGCTCAGGAAAATCAAGCTTTCTCTCTTGCATCCTTGGGGAGATTCCCAAAATCTCTGGTGAAGTA**GTTAATTTAGACTTTGATGACCTTTCTTTTAATTTATGATTAATGTTATTTTTTCTGTCAACTATGATTTCTTCTTTATCCTAGTAATTGAGGACGAAATTGCCTAAATTTGTTCTCTCCTTTTTTCTCAGGTAAG**AGTTTGTGGTACTGCTGCTTATGTTTCCCAGTCAGCATGGATACAATCTGGAAATATTGAAGAAAACATTCTTTTTGGCAGCCCAATGGATAAAGCAAAGTACAAAAACGCTATCCATGCTTGTTCACTGAAAAAAGATTTGGAACTTTTTTCACATGGCGATCAGACCATCATTGGTGATAGAGGTATAAATTTGAGTGGTGGCCAGAAGCAGCGGGTACAGCTTGCAAGGGCACTATATCAAGATGCAGATATTTATTTACTTGATGATCCCTTCAGTGCTGTTGATGCACACACTGGTTCTGAGTTGTTCAAG**GTTAGATTTGTTCAAAGCATCATGAATATTGCTGTTCAAATAAATGTATTTGCTGAACATATGATATGGAACTGCAG**GAATACATATTAACAGCACTAGCAACTAAGACAGTTATTTTTGTAACCCATCAAGTTGAATTTTTGCCAGCGGCTGATCTAATACTG**GTATGGAATTGCTTTCTCATTTTACTTTTTTATGTTTGTGCAGTGACAGGTCACCTTGGGGCTTTATCTTGCATCTGAATGAGATTGCTCTTTTTCTTATTTTATTTTTTTTTTCATAACCCATCAACTTATTACAATATTGCGTTCATTGTTCATTTCGATAAGATCTTTTCCACATTTTGGTTGATTTCCCTTTTTAGATACTCATTGTTGTTTTTGTTGTTGTACTATTGAGAGAGTTGATCCTCAACGCTGGAGTTATTAGTGGGCATCCATATATAGGAAAAATAAACATTATTTTCTATTAACTGAAGGACTGATGCGGCCAAGGAATTCAAGAAAATTCATGGTCAAATACTAAGAAACTTAGGATTATGGTTTAGTTTTAATGTAGAGTTTGTTGGGAAAAATTATTTTGTAATTCTAGTTCTTTTAGAGTTTTGTGTCTATTTAAATATATTAGTTGTTGGTCCTTTATTCAAGTCACATTTTGTAATTTCAAATTTTCTAGAAGGGTCTAGATTTTAATAGGAGTCTTTATTTAGAGTCCTTGTTAGTTAAGGATTACTTTTTGCGTACTTAAGGTTAATGATTACTTTTTGGACCCAAATCCTTCCAGATTTTGGATTACTTTTTGAGTCTACGCTTGTAATGTTGTTTTGAAGTTTTTGATCAATACGAATTCTAGCAATATTGCTTTGAGTTGTTTGATGCAATTCAACCCTAGATATGATGCTTATGGAACATTAGGTGTGATATTCTTAAGGAAGTTTAAGTGTGAAGCCTAAACTTTTTATTTTATTATTCTTTAATCTTGTATTATTATGTCATATAGCGTAGCTATTTATGGTTTATTATGAATTTTATGCAAGGATTTTATGTAATACTTTGATGTTAAAGATTTTTTTTATAATTTTATTAATAGTATTGAACTCATAATTATTTTTCAACTATAAATATTATATATTATTTTTATAAATAAAAATTATTGTAAATATATTTTCATTAATAATTACTAAATAGTAAAAAATATATATTATATTTTTCTGTTAGTTTGGTTTTAACAGATAATCAATCGAACATTTTATGTTTCGATTAAGTTTGGTTTTCTTTCAATTTCGGTTCGATTATATTAGTATTTTAGAGTTGGTTAAAAAATCAATTAGTAAAAAAATTCGATTTTGTTTAGTTCAATTAATCGAATGTACACTTAATTAGCTCCTTGAATGAAAGTTTTCTGGTTCTGCCCCTGAGTGGCAGCATGCCGATTGCTGGCTATTATGAAAAGCCAAGCATTCAAAGCCAAACAGCAATCGGAACTACACAAGAATTTGGCTCTAATCTTTCAATTTCCGGGGTTCATAATAATACAACCTCTCAAAACCAAGAGCAGAAGCAACAATTACATGCAGGAATTTATGGACAACATTCAATGGATGAACAAGATGAGCCCCATCAGCAACTTATAAAGGTGGAACAAAAGAGGCAACAACTAGAAGAGAGCCTATGCAACATGATGATAAGTTAGAACAAGAAACCAAAAAGTTAGAATTGTCGTCTCAAAAGATTCAACAACAAAACTTGCTATCAAATTTGAATCAAATGAAGATTGAGCATTAATGCAAGGAATTTTTTGTCAAGAATGCTATTGAACAAAAAATAGAAGGAATAAAAGAGTTTGGATTCAGTGATCAAGATGTTGAAGATGATGGATTTGTATTGCCTGCAGTTGATATTCAAGAGATGAAGATGAATTTCATTATTCCAACAAAGTCCATTAATAAATCCAAATGCGGCTTTCAGGTGTTTCTATTGTTGTTGACAACTAAGGAGGTGACATCATTGTTCAAGAAATTGTCATTTTTGGTGCTTAAGAATTTCAAAACTCAAGGTCGAGTTTTCTTCAACAAAGAAAGAATGATGCAGCTAAGGAATTGAAGAAGATTCAAAGTCAAATACTCCATAAACTTAGGATTATGGTTCTGTTTTAATGTAGAGTTTATTGGGGAAATTATAGAGTACTTTCTGGGTCTTTTAGAGTTATACTTATCTGTCTATTTAAATAAATTAGTTATTGGTCCTTTATTTAATCTTAGGTATACTTTTGCAATTTCAGATTTTGTAGAAAGATCTAGATTTCTATAGGAATCTTTATTTGGAGTCTTCATTGGTTAAGGATTACTTTTTGGGTTACTTAAAGTTGAGGATTATTTTTTGGAGTCTAAATCCTTCAAGGTATAGGATTACTTTTTGAGTCTATAAATACTTATGCTTGTAATGTTGTTTTGAGTATTTAGAGTCTTCATTGGTTAAGGATTACTTTTTGAGATTACTTAAAGTTAAGGATTATTTTTGAGAGACTTAAAGTTAAGGATTATTTTTGAGAGTCTAAATACTTAAAAGTTTCTGGAGTCTAAATCCTTCAAGGTATAGGATTACTTTTTGTCTATGAATACTTATGCTTGTAATGTTTTGAATTGTTTGATCAATATGAATTTTAGTTGCACAAATTTAAGCAATTACGCATCGAGTTATGTGATGCAATTCAATCCTAGGTGTGATGCCTATGGAACTTTGGGTGTGATGCCTATTGAAGTTTTTAGGTGTGAAGCCTAAACCTATTACTGACACAACACACAAATTTTCTTGTTCATGTTTAAATTCGCTTTTCAATTCCATCCTTTCATAAACCCTAATTCTTCACCTAAATTCCTACTTATTTTAGCCTTCACAAAGCCTCAAATTTCAATATTATCTAATACCTGCAGGCTCTCCTGCATCTAAGAGCCAGGTTTTTCTTGAAGTCAAAATATGAATGTCGATGGGTGGGTTAAATGTGTATGGAATCTCCATGCTCTGAAGATATCTAATAAATGTTACCTTCCATCTATTCAG**GTTCTCAAGGAAGGGCGCATTATACAGGCGGGGAAATATGATGATCTTCTGCAAGCAGGAACTGATTTTAGAACTTTGGTCTCAGCTCACCATGAAGCAATTGTAGCTATGGATATCCCATCTCACTCATCTGATGATTCAGACGAGAGTTTGTCTTTGGATGGGTCTGTCATATTTAATAAAAAATGTGATGCAACTGGAAGTAATGTTGATATTTTGGCAAAGGAAGTGCAAGAAAGTGCATCACCATCAGATCAGAAAGCTATTAAAGAGAAAAAGAAAGCAAAACGCTCAAGGAAAAAGCAGCTTGTTCAGGAAGAGGAAAGGGTGAGAGGGAGAGTCAGTATGAAGGTTTACTTGTCATACATGGCTGCAGCATATAAGGGCTTATTAATTCCACTAATTATCCTTGCACAAGCATTATTTCAATTTCTACAAATAGCTAGTAATTGGTGGATGGCTTGGGCAAATCCCCAAACTGAAGGAGGCCAATCTAGGATGAGTCCTATGGTCCTTCTTGGTGTTTATATGGCCCTTGCTTTTGGGAGTTCATGGTTTATATTTGTGAGGGCTGTTCTAGTTGCTACATTTGGCTTGGCAGCTGCACAGAAGTTGTTTTTGAAGATGCTTAGAAGTGTGTTTCGAGCGCCAATGTCTTTCTTTGACTCTACTCCAGCTGGACGGGTCTTGAATCGT**GTAAGTTCATCTGTTTTTGCTAGAAATATATATCATCTGCTGGAATTATTTATATAAAGAACGAAGAATATTTTCTTTTTCTTTTACCCTCTCACTCCTTTCTTATCTTTATATAGTAGTGAAATTTATGACCTTACAG**GTATCAATTGATCAAAGTGTCGTGGATCTTGATATTCCTTTTAGGCTGGGTGGGTTTGCTTCAACAACAATACAACTTCTTGGAATTGTTGGTGTAATGACCAAGGTTACTTGGCAAGTTTTGCTTCTTGTTGTTCCTATGGCTGTTGCTTGCTTATGGATGCAG**GTAATTGATTGATTTTTCCTGTTTCACTTAGAAAATGAAAGGATATTGATTTTTTGCAAGTTGCTTCTGGACTTTACTAATTTTCCTGCATGTATGTGTCATTGTGAGTGACAGTGAGCACCACATTACTGCTTTAGATTTGGGTTGCAGCATTTGATATTACTTTTATTTTGTGCTGTGTATTTGCACAAGCGTCTTTATGGGAAGACAATAAAATCCATTAAGTGGAGATGTTGGAAGTTGCTTACGGTGACTAATAACACTGTTTCTCTGGGGGTTCACTCAATGTAGTCTTTACCCCTTAAAGTGTGATATTTTATTTATTGTGGAAATTATCATTCCTGAGGCCTTTTGCATGTCTTGATCATAATATAAATTTCGTTGATATTGTTGTGTGAAGATGAAGTTTTGGTATCAATGTTTCATTATTTGAAACCATATTATTTATGTCCATATAGCTTGAACTGTTTCAACCATGATTTGTGAAGTTTATTGCTGCTTGCTTGTTAGAGAAGAGCTAGTATGATTTTGAACTTAAACTTCTAATTTAAGGACAATTTGATAATTTGGCTAACTAATATTGAAAGATATGTCAACTTATTATCTGTATGCCCATATGCAG**AAATACTACATGGCTTCATCAAGGGAACTGGTCCGCATTGTTAGTATCCAGAAATCTCCTATCATCCATCTTTTTGGTGAGTCAATTGCTGGAGCAGCCACAATAAGAGGCTTTGGGCAAGAAAAAAGGTTCATGAAGAGAAATCTTTATCTTCTTGACTGCTTTGCTCGTCCATTCTTCTGCAGTCTTGCTGCTATTGAATGGCTGTGCCTACGCATGGAGTTGCTCTCAACCTTTGTATTTGCTTTCTGCATGATTTTACTTGTTAGCTTTCCTCATGGAAGTATTGATCCAA**GTAAGTAAATCATTCTGCGCTACTTTATAAACACACACACTCTTGTTTGCTTTGGGAACTTTATGTGCAGCGAGAGTTTTTTTAGTGTTGTAAAGTTCTATTTTAGTCTTAGGATGGCTTAGCTTGTGATGGAAATTGATTGAAGTTGGATACAAGATAAATAGTAAACCCTTTTAATTTGTAAGTTTATGCGGTTATCAATCATATTTGATTGTTCTTTACAG**GCATGGCAGGCCTTGCAGTGACATATGGCCTTAATTTAAATGCACGCCTATCGCGGTGGATTCTTAGCTTCTGTAAACTAGAAAACAAAATTATTTCCATAGAAAGGATTTATCAGTACAGCCAAATTCCAAGTGAAGCTCCATCTGTTATCGAGGGTTTCCGCCCTCCATCCTCATGGCCAGAGAATGGGACAATTGATCTGATTGATTTGAAG**GTAGAAGCTTAGCTTTATTCCACTATGCATGTGTATTATCCAGCCTTGAGGAAATGTGTACTTTCTTTCGTTTTGTTCTGATTGTTATGTTGGTTTGTCCACAGATGATGTCTGTTGGCATTCTTGGGCCTTCTGTAACATTCTTGCTGATTTACTCTGGATCAGGTTAAATGCTAGTTATAGGATATATCATGCCAACATATGTCAATATTCTGGTTTCAAACTTAAAAAATTAGCACCCACATTCAGAGTTATCAATTTTGTAACATGGAGTTTGAGAGTCATGATGTCTTTGTAGGAATCATGCATCTCTTTCTATTCTGGAGTTGATTCAATTATCTTTTGACCATGAGCTTATTGTGCTTGTTTCCATGACTGTGTTTGATTCTCTAGCTTGAGAAGAAAAGTGAACAGGAAAGTTGGATTTCTGTTATGTATATGCATATATGTAACAATATTTTTGGAGAAAAACGATGGAAGCAAGATTGCCTGGCTGATTCTTAATTGTTGGAATTGGTTTGAAAATGCTGACTCTTTCTTTTTCTGAAG**GTTCGTTATGCTGAGAATCTTCCTACGGTGCTTCATGGGATAACATGCACCTTTCCCGGTGGAAAGAAAATTGGAATAGTTGGGCGAACAGGAAGTGGTAAATCTACATTGATCCAAGCACTATTTCGATTAATTGAACCTGCTGAAGGGAGAATCATTATAGACAACATTGATATTTCTATGATTGGCCTACATGATCTTCGTAGCCGTCTGAGTATCATACCCCAGGATCCAACTTTATTGGAAGGGACAATTAGGGGAAATCTTGATCCCCTTGAAGAGCATTCAGATCAGGAAATTTGGCAG**GTTTTATCCCGCTTCCTTCCCCCCTCCCTCCTTATTTATGTAATTACATACAAGTTTACCTGGATTTTGAATGTTAAAAAGAAAGTCTTGATTTTAGTCATCCTTTGAACAAAAGTTCATATATCTGCATATTAAATTTTTGACCAATCTATAAAATAGCTAAGCATGTAGTTTGTTATAAATGGATCATTGCCTTCTGATAATTGAGATGCGAAGGTAAATATTTTTTATAAATCAATTACATAATCAATTTTCAAAAATTTCCAGATTTAGATGTTTTAAGTGATTGCCAGTATTAGTACAAGTACTTAGTTTCCGCATGGGAACACTATATTGCCAATTTATGCCTAAAACAATTTTTTATGTATGAAATCTTTTCTGATTATATGCTGAGAATTCAGCTATTTGTCACAG**GCTCTTGACAAGTCTCAGCTTGGGGAGATAGTCCGTAGGAAAGATCAAAAGCTTGATACACCAG**GTATGCAGCTAGCAGATTGGAAAATATGGTATTAATGTTTTCTTCTCCCTCATAATCCCCAGAGTTTGGGTGGTTTGGATGGCGTTTTGTTTCTGATTGATTAGTTTGTATTGGAATGTATAATTTGTAG**TGGTAGAAAATGGGGATAATTGGAGTGTAGGGCAACGGCAACTGGTTTCCCTTGGCCGGGCTTTGCTCAAGCAGGCAAGAATACTCGTGCTAGATGAAGCAACAGCATCAGTTGATACAGCTACAGACAATCTTATTCAGAAGATTATTCGTACGGAGTTTAAGGATTGCACAGTTTGCACAATTGCACATCGCATTCCAACTGTTATCGACAGTGATCTTGTTTTGGTACTCAGTGATGGTGTGCACTAATGATTTCAAAACTCCAACTTCTGTCATAGAGTCAAGTTGTGTCTCAGTGTGACATAGTTGACATATAGGACTTGTTGAGTGGATTTTTTCATTTTGGTAGACTTGTGAAGTGAAAAGTTCTCATGAAACTAATTTCACTTTGGTATACTTTTGCAGGTCGAGTAGCAGAATTCGATACTCCAACACGGTTGTTAGAGGATAAATCATCCATGTTCCTAAAGCTGGTAACAGAGTACTCATCAAGGTCAAGTGG**

>HbABCC13 scaffold1216(36083-77173)

**GGGTGCTTATATTAATCAGGCATTTTCTCTCAAATTTAAGTTTCGAAGTTATGGGAAAATTCCCCTTTTTGTTGTCTCATATGATTGCTCCCTTTGCAACCAAAGCAATGGAGTGGAAGAATCTCATTTGCCCTAATTCTCCATTT**GTATGCTTCTCTTCTCCACCCTTTCCCTCTTCTCTCTTTAAGCCAATTTGCTGTGGCTGATTTAACATGAAAAATTATGTAACTGGAATTCCCAATACTTATTTTTTCTTTTTTTAAATTAGAAATTGGACCCGTGTTTTTTTGTGATTTTAATGAGGAGAGCCTTCAAGTCCGTTTCTATGTAGAATTAGTTCAACTTCTGGTTAGTTGCTGGGGAAGTATAGGAATGCTCAATAAAGTGCAATTCTTGTTCCTTTGCATAGCAACTGTGTGAAGAAATACTGTACAATGTACTCAAAGACCTGTGAAGAAGCTTAACTTGATATTTTGTCAAAATTAATTTGGTTTATCATATTAAAAGAGAACAAATAAGAATTTTGCTCTTTGGCATAATATTCCAGAATTGTGAACAAACACACCACAAAACCAAACACCACCCCCACCCCCCTCTTCTCTCCTCCTTTTCGCTTTCCCCCTATTTGAGTATTTTAATTCGCATTCTAAGGTTTAATTTTCTGCTTTTCTTTTTTCCTTTTTTTTCAG**GTATGGGATGGAAATAAAATCTCAGAATGTTTTGATAATAT**ATATCCTTTTGTGTATGTTGAGGAGGTTTCCTTTTCAGCTCTATAAGATATTAATAGGTGTTGTTAATTACTTTTCCTTGACAATGCATAC**AGTTTTAGGATTTGGTGCAAATGTAGTGACTGTTCTTATGATTTCAATACTTGCAATTACACTGAGGAACGCCAGAGGAAGCCACAGG**GTAGATTCTTTATTCTTTTTTATACTTATGGTCTACTTACTTGCTGAAAACTGTAAGGAAAGGGTATTGGAAAGCAACATTGGGATAATCATCTTAAGCTGTGTCTGTGTTTCCTTCAGGATGCATTTGGTTAAGGAGGATAGTTTTTTTTTTAAAAAAAAAAAGGAAAAAGCAAGGGTTAGATGAATAATCATATGATGCATGCAGCTGCACTCTGTATTAAATTTTTTATCTGCTATAACTTAGTGACAATATCTGTCACAAATAAACTCCATGTGTCTAATTTAATGTGATTCTGATGGCTTTGACTGTTTATCTTTTCCATTTTGAGATGCATAGTTTATTTAGTTTCAAAATTTCCTATCAGATACCACCTTTGAGATGCATGGTTTATAAATTCTGCCCTTTTTAATTCAAGTAGACTGTTAAGCTGACTGTTAGTTCTTTCTATATCCTTAAACTTTGCATGAAGTTACAAGATGAATTCTGACAAAAACGTTAGTTCATTATAACTAGATGTCCTCTCGCCATTCAAAAATTATTACATGGTGGACTAATCCTTTAGAAGTTATATGCTCAATTAATATAGATTTAGTTAATATTTCTCTGTTGTATTACAAGTTCTAGACTAAACAAGTCAGGAGTCCTTGTTCCCGGTACAGGAATGTACTTGCAGAGTGAAATTATGCTTTGATTGTTTAATCATCACTTTTGGTAAACCTAGTTTGCCATGACCTTGAATGTATCTCATCTTGAAATTGATCTTTCAACTTATTCAATATCCATTCATTGCTCATAATAGCTTGAGCTTCTATTTATCTGTTGTATGTTATGAAATACTCTCACTAGCATTTAAGTCTCTGATAAAAAATCTCTGCCTATGTGGATTTTCTGAAAAATACACTTGATTAGTATTTGTGCCTTTTTGGCCAAACAGTTTACGTTGTTCCTCCTAGTATTAGAACTGATAGACTTGTGCATAGAAGCTTCTCTCTACTGGAGAGGTGCAGCTGCTACTTAGGCTGTGCTGTGACCTTATGTTGAACTTGAACTAGAAGTTTTCCAAACCTCTATTGTGATCAGTAACTTTATGTGTTTTGCTGCTGGATTTCCTTATTTGACTATCAGTTCTTCATGCTGGTGGGTGTGCACTAAATGTTACCCTTATCTACCTACCTGACTTCTTCTCCAAATTATATATCCAAGATTAATGTCAATTCCTTTCTTTCTTTCTGGATTAGGCATCTCTAATTTTCTTTTAAAACAATTTTTAGAATAATTCTAATGCTTTTATAAGAATTACACAG**ATGAACTTCAGAGAAAAGGTAGTGTTTCATTTTCTACCTGCACTTGGAGCATGTTTGTCATTTGTGGACATGGTTTTTCTTTTGAAGAAAGAACTTAATGGAGACTTTATTGTATATCATGAATGGCTTTTCAAAAGTTCTCAATTGATACTCTGG**GTAAGCTGTTTCTAACTTAAGATGTTTGCCTTGTTGTCATGAGCTTTTTATGCTGGCTCAAGATTTTAATTCAATATTCTGTTCCTGCAAATGTGCAG**ACAACCATCATAATTTCTGTAAAGTGGGCCTGTTTCCATGACTTATTTTGCAATTGGCTTCTTTGTATCTGGTGGATCATGAAAGCACTACTGGAAATTCTTCATCTGCATAAAACATTTTCTTCATTGGAG**GTACTTTTTTTTTCCCTGTTAGGTTCCTTATCTCTGCATTGCATGGTCATAAGTGATTTTCTGTGCTGTTATTTGTTTCAGTGGATTAAGCCGTGGATGTAACATTTTTGTCTCTACGGACTAGCCAAAATATAAATTTGCTTTGTTATAATTATACTTTAAGACAAGTAGAGCACATTAGGTTAGAGGATAGAAAGAAAAGAAGGGGTAGACCTAAACTGACTTGGAGAAGAGTAGTACAACATTACTTAGAAGCATTACACATTTCCGAGGATTTAACCCAAAATCGTTTAGAATGGAGAAAGAGAATCCATATAACCGACCCAAATTCTTGGGATAAAGGCTTAATTGAGTTGAGTTGAGTTGAGTTGTATAATTATACTTCAAAGAAATTTATGACAGGTTAAACATATAAAATTGTTTTTTATATTTTACATGCAAAAATATAAAATTTGTGTAAATAATTATATAATATTTAAATAAGTGTGAAAGGTTATAAACAATATGTTATTTTCATAATTAATTGATTATGTGCAAAGCATGTCAATTCCAGGCCAACCCTACATGGCATAATTGTGTCAACATGTTATCAAATAGAACATTATTGTCAAACTTGAACCCTCCAATTTTGAGTCATGATGAATTCATGTTTGCATATCATTTCTAATATTGTTGTTATGATCGACATTGTATAAGTTGCAGATCATACTCATGTGGTGGAAAACCTTCTAGGTTTTGCTTGATATATCTACTCTCCGAAGGCTATTCTTAAACTCCTTGTCATACTTTTTGGAGACCTTCATTCAATTGTTAGTGGCATTTGTGTTGTTTAGGTACTAAAGAATATTATGCACATGGGCATTTTATTATGTCTGGAAAACATAAATGGTATTTCTTTTAAATTGTCTTGGGTAATCTTTTATTGTATATACGTTTGTTTTGCGGTATTTCATTTTTTTCTTCCCTGATAAGATGAAATCTTAATTCTTTGAAAATTTTATGAGGGCAG**GCTTTGGAATGTCTGAAAGAAAGTTCAGTTGTTTTGTTGGATATCATGTTTGGCATAACCATCAATATCATCAGGATAAAACAGTCATCTTCCAAGGCCAG**GTATAACTATAACCTCTCAATTTTTTGCTTGCAATGTCTTTATTTTTTGATAGTAACAATATTGCACTGGTGTCACTATAACAAGCTTATAGATAATTCAAGTGCAAAATCTCTTAATGCTAGCAAAGGAATTGTGGTTATATTGTTTATGACTTGATGTTATGGTTGGCTTGTTAGAATGGGTCTGTGTAATTCCAGTGGTTATATTTTGAAAATGATTTGGTTCCTCATTGTTTTTACTGACTTCTAGTTCATTATATGTGATTGTCTGGGTGGATCTTGTTCAACATGAACTTTTGTTCATTTTTCATAACTTTATTTCAGATTCCCCCCCTCTTAAAAATGTACCTTTCCATCACCTCATATGATACTGCCAAAGATATTTGTGACAGTTCAATTTTTTTTTGCATAGGAGTTGTAGTAAAATATGCCATTGCAGATGCAGCTGAGTAATATAAGTTGCCAAACAGAAAGATGTTCAATTATTATTGGAACGAATATTGAGAAACATGTTGAATAATGGGATGATTTGGTTTAAAATGCTTAAATAAATTATTATGAAGACAGTGTTTTTTCATGGAGGAATTAGATGTGAAAACAGAAAACTTAATATGGAAAGTCCTGTAAGGTGCATGTTATTGCTATCCTTAATCACCATGGCTTCTAATCCTTCATTGTTATAGCATTTTATGATAACTGAAGGGGCTTCTGATAATCTGATTTAAAAGAGTTTGTTTCTCAATTCCATTAAGCTAGCATCATTTACTCTGTTGAATGGCCATTTTCTTTCTCAAACACATTCACAGAGACACTTGTACATGTGTGCATGTATGCATGTACTCATGCACATAATGAAACATCAGCTCTGAGTTATATCTGTTTGTTAATAATCGCTACATACCCTGTTATATTTGTTCACTAACAGTTAAGTACAATGATTGAATTTGTTTTCCCATTGATCATTTGTTACTGTAAGTTTTCATGTCAATATACCACATCATCTTGTCACTTATTTGTTCGTTTGAGATTTGTTTTTCTGTGAATGAATAACTGTCATGCTACATCCTTGTTATTTTTCTAATATTGGGATTCATTGACGATATTGCTTTTTGCTGAATTGTAG**TTCAATGGAAGATCCGCTTCTTTCTGTTAATATGGATATTGAGGGAGGTTTTCCTGGGGATTCT**GTAAGTGATTTGTGGCCATTATAATTTTTTTTCAGGAAGATTTACACTTTCTTCCTTATAAAAGTATAGATTATTTGATTTTCATATTTTGTAGTCAGAAGTTTTTAATATATATTTTCCTGCAGGAGCATTAAAGTTTGTAGTTAAACAAGTTTTTCAAAACTTTGGAATTCTTGCACTATCATCAGTCTATTTTATTATTTTATACTTCAGCAACCAAAGATCCATTGATTTTAATCTCAGTTAATTGCATATTGATGCGTGAACCACGAGAGAGGATGTCTGTATTTGTATTTTATTTTTTTTCTTCCCTAATTGAGCAGTTTATAGAGAATATCTGGATTAGATTAGATTTTCCTCATTTTTTTGTGCTTTATGGAGCTGAACTGAAGTTGACTTGAATACTGTAGTTTGACAAGCCCCATACCATAAATATAAAGGTCCTCCTTAATTGATGCATCACGTTTATTGTTGTTGAACAGTTAAACAGTACAAAAACTTATTCTATTGGATTATTCTGTTTATAGTTGGCTTTCTTGACTTCTGGCTGAGCCAGGAATATCTGTTAGTGGGGTCAAAAAATTTTAATTTACTTAATTTAGAGAAAAAATATTATATAATAATAAACTATCTATTAAGAGAAAAAAATATAATAATGCACGTAAATTTTCAATATATAACAATAATTTAGTTAAAAAAAATAATTTTATAAAATAATAAATTTTCGCTACAATCTTAAATTTACAATTGTCCTTAAAGAGTTTTCATTTTCTGGAAGGTATTGCGTGATTTCTTATAATTATCAATGTTATCAAAAACATCTTTCTCAATATATATATAAGCAAGGATCATTCAATAACTGATCTCCCATTGAGTTTCCACAATTTTCATGGTAGAAAATGCCTTTTCAACACTTTGAGTTGTAAGTTGTAATAGGTAATATCAACAGTAATTTTACAAGAAAGTGAAATAAAAAAAATAAAAATTTAACTTATGAAATTGAGTTTGTCCACTAGTTTAGTTAACATAAAAAGGAAAGGAAAAAGAAAATAAAACCTAATACCATTAATAAGAGACACAGAGAGAGAGAGAGAGAGAGAGAGAGTTTTTTTTTTTTTAAGCTTTATAATTTATTTAAAAAAATTAAAATTGTAGACTATGATAAAGTGTCAAATAAGTCTAAATATATTAAAAATAATAATATTTTGAAGATTTTAAAGGGATAAAAAAATATTTTAGTGGTGTCGTATAAATAATTTAATATATAGCATATATTAAAGTTTTTTTAATTTTTAAAAATTGAGTGGGGTCAATTGAACACACTTACCAACATGTAGCACAGTTCTGCCACACCTTCTTCTTCTTTCAATGTGTTATAGTGCTATCACCTATGAAACAGTTGACCACTGTCTCCCTCTTCTGGTTCATGAGTAGTGCAATGTCAATTTTTTGGTTGAGGTGAGCTTATGCCCATAATGAGTTCATCGATCTTTTTTGTTATTTATTTTTCTTGTTTTTTGTTTGAAGTCAATAAAAATCACAATAAATTATTGCATTTCATGTTACTATCAAAG**GGAAACACATGGAGCAGTTGGGATCTCATGACATTCAAAGCTATCACTTCTGTGATGAAGCGTGGCGTCATAAAGCAGCTTGGCTTTGAAGATTTGCTTTGGCTACCTAATGACATGGAACCCTCAACCTGTCATGATAGGTTGCTAAGCTTCTGGCGAGCTCAGCAGGGTAGCTCTAACCCCTTCTTGTTTAAAGCAATCTGTTACGCATATGGATGGCCATATTTTTGTATTGGTTTATTAAAG**GTAATTTATTCTTTCAGGCTGAATTTCTCACTATATAATAAAATTTATGTGATTATATTTATTATCAAGATAGGGGGCTGAACCTAATTTAAGTTCTTCATGTAGTAAATTGTCCTTTTGAATTGTTGGTTACTGATGCAGTTCCTCATTTTGCTTTCTCTTTTTAAATTAGTACCTTTGTTATGACAGTGCTAATGCTTATTTTATTTCATTTGCACGCTGAAGGAGTTGCTGAGCTAAAATTATGTCACATATTGCTCTTGTAAACATTGTTAAAATTAAGCAAATAAGTAAGGCGTATTTATCTTTTGTGATAGGTGTTTGGTTTTCATGAGAAAAGTTTCCCTTTTACTGAAATCTCAGACCACAATATTAAAAAGGGGAATAAAAACAAAAAATATTGGATTGCATAAAACATATCACAGAGAAAGCCATTCCAATTGATGATATTAGTGTCTCTAGCCAATGCTTATTGCTTAATAAAATATATCTATGATGAATAATATTTTCATTGACTAATTTCTTCTAGTTCTTTCTCAAAGGGAAATAGCTTTACAGTGAGAAATTTTACTTTTACAG**TTGTTGAATGATTGTATTGGTTTTGCGGGACCACTGCTTCTCAATAAACTCATTCGGTTTCTTCAACAAG**GTATGGAGCATGGATCAGTTTTTATTTTTGGTTCTTTAGATAGCATCTACTAGTTTTGGGGTGACACTGTAAATTTAAGATAATTGTCATGTCATTTTTGTTTTGGCCTTTCCCAGTTGTGGGTTTTCTGTCTGCTTCATTGTTCAATAAGCTCCTCTCTTCACGGTTCTGTAATGTAACCAATTTTATTGCCAACTGGGTGTATTGGAACAATAAATATGTGTTCTTTCATTTAATGTTTTAGCTAATGATAGATCTGTAGAAAGAGATGATTCTCCTTAATTTCAATTAATCTGTACATGGGTGTATATATATACAATTGATCCGTATAATTGTGTTCTACTAATTAGGAAGAAATCTTAAATAAGAAATCTTAAATAGGAATATAGAATACAGAATATACAGAAAAATAATATAGTGATTGACTTTCCATAACACTCCCCCTCAAGTTGGCGCATAGATGTTAATCATGCCCAACTTGTTACAAATGTAGTGAATCCTAGCTCCATTCAGAGCTTTTGTGAAAATATCTCCTAACTGCTCTCCAGTTTTGATGTGTCCTGTTGAGATGATCTGTTGTTGAATCTTTTCATGAACAAAGTCACAATCAATCTCAATATGTTTGGTCCGCTCATGAAACACCGGATTAGAAGCACTATGGAGAGCAACTTGATTATCACACTACAATTTCGCAGGCAGGGAGGTCTTAAAACCTGTCTCATCTAGTAATTGAAGTATCCACATTACTTTACATACTGATTGTACCATGGCTCTGTATTCTGATTCAGCACTAGATCGAGAAACTACACTCTGTTTCTTGCTTCTCCAAGACATCGAATTTCCTTCAATAAAAACGCAATATCCAGTAGTTGACCTCCTGTCAACCTTAGATCCAGCCCAGTCGGCATCTGAAAAACATTCAACATTCAAATGCCCATGATTACCATATAACAAACCTCTTCCTGGAGCGTCCTTCAGATAACACAAGATTTGTCCTAAGGCTTCCCAATGAGCAACAGTTGGGGAAGACATAAACTGACTTGCCACACTAACGACATAAGCAATGTCAGGATGAGTGACTTTAAGGTAGTTCAATTTTCCTACCAATCTCCTGTATCTCTCTGGATCTTCAAACAACTCACTATCCCCGGCTAACGGTTGTAAATTTGGAGTCATTGGTGCACTGCAAGGCTTAGCACCTAATTTTCCTGTCTCTGTCAATAGATCGATGATATATTTTATTTGAGACAGTAAGGTAGTTCAATTTTCCTACCAATCTCCTGTATCTCTCTGGATCTTCAAACAACTCACTATCCCCTGCTAACAGTTGTAAATTTGGAGTCATTGGTGCACTACAAGGCTTAGCACCTAATTTTCCTGTCTCTGTCAATAGATCGATGACATATTTTCTTTGAGACAAGAAAATACCCTTCTTACTTCTCATAACTTCAATACCCAAGAAATACTTTAACAATCCCAAGTCTTTTGTCTGAAACTGGGTTTGGAGGAAGGTTTTAAGAGATGAAATACCTGCAGAGTCACTCCCAGTAATGACAATGTCATCCACATAGACTACCAGGAGAATTAGACCAGCCTTAGATTGCCTATAAAATACTGAGTGATCACACTTACTTTTTGGCATACCAAATTCTTGTATTGCTTCACTGAATCTCCCAAACCAGGCCCTAGGACTTTGTTTCAAGCCATAAAGAGACTTCCGAAGCCTACAAACTTTACCCAACTCTCCCTGAGCAACAAACCTAGGTGGTTGCTCCATATACATCTCCTCCTGAAGATCACCATGAAGGAAAGCATTCTTGATATCCAATTGGTGCAGGGGCCAATCATATGTAGCTGCTAAAGAGATAAACAAGCGAACAGAAGTAAGTTTAGCTACAGGAGAAAAAGTGGCAAAGTAATCAATCTCATATGTCTGAGCATATCCTTTTGCCATAAGACGTGCTTTTAACCTAGTCACAGAACCATCAAGATTTACCTTTACTGTAAATACCCATTTGCAACCAATAGCTTTCTTACCAGTGGGTAAAGGCAACAGTTCCCATATACCATTAGCATCTAAAGCCTTTGAGTAATCAACCCCATATGTCTGAGCATATCCTTTTGCTACAAGGCGTGCTTTTAACCTAGCCACAGAACCATCAGGATTTACCTTTACTGTAAATACCCATTTGCAACCAATAGCTTTCTTACCAGTGGGCAAAGGCAATAGTTCCCATGTACCATTAGCATCTAAAGCCTCCATTTCCTCTTTCATAGCAGCACACCAGCCAGAATGAGACAGTGCCTCACCAACAGTATTAGGGATAGGAACAGAGTCTAAAGAAGTAACAAAACACCGAGAACAAAAAGACAATTGATTATAAGAAACAAAAGAAGAGATAGGGTAAGTGCATGAGCGCTTACCTTCACGCAGAGCAATGGGTAAGTCTAGGTCAGAATCATGATCAGTATGAGGTACAGGATCTCTCAACGAAGTAGCTGGTGGAGAATCTGAGTCAGGAATCTCCAATCTCCTGGAATAAACATGAACAACTGGAGGTCGAGGAGGTCGAGTAGGTCTAGAGACAGAAGGAACAGGCTGTGGGAGAGGACTAGACATTGGTTGGACAGTATATATTAAGAGATCATTCTCCTCCCCCTGACTCTCATACACAGATGATTGAGGAAAGAATGGAGTAGACTCAAAAAATGTGACATCTGCAGAAACAAGATAACGATTAAGAGTAGGAGAAAAACAGCAGTACTCTTTTTGGAGCCGGGAGTACCCAAGGAAGACACATTTGAGAGACTTCGGATCCAATTTAGTAACTTGTGGACGAACATCACGCACAAAACAGGTATAACAAAAAATACGGGGTTCAACAGGGAACAAAGTTTTTGTAGGAAACAAAGCAGTATAAGGAATATTCCCATTAAGGACAGAAGACGGCATACGATTGATCAAAAAACATGTCGTAGAAACTGTATCCGCCCAAAAGTGTTTAGGAACTTTCATATGAAAAAGAAGAGCACGAGTTACCTCAAGAAGATGCCGATTTTTTCTTTCGGCCACGCCATTTTGGGATGGGGTATCAATACAGGAAGACTAATGAAGAATGCCATTTTGTGTCATATAAGACTGAAATTGTGCTGAAAGATATTCTTTGGCATTGTCACTTCTTAATATGCGCACAGAAATATTAAATTAAGTTTTGATTTCATTACAAATGGCACAAAAGATAGAAAACAACTCAGAACAATTCTTCATTAAATATAACCATGTAACACGAGAGTAATCATCAATAAAAGTAACAAAATAACGAAATCTAGTTTTAGAAGTGTAGAAAGAGATGATTCTCATTGATTTCAATTAATCTGTACATGGGTATATATATACAATTGATTCCTATAATTGTGTTCTACTAATTAGGAAGAAATCCTAAATAAGAAATCCTAAATAGGAATACAGAATACAGAATATACAGAGAAATAATATAGTNNNNNNNNNNNNNNNNNNNNNNNNTCAAAAGGGGATGAAGCCCGTTTATTGACTCTAGACACAGAAGGCAAACGATGATGTTTTGCAAACTGACACGACTCATATTCTTGTACTGATAAAGACTGAAACTGGGGACACAGCTTCTTCATGGTAGACAAAGAAGGATGGCCCAATCTACAATGAGCTTCAAGAGGTGTTAAGGTACTGGAGTAAACAAGTGACCACGATAAATGATTTTCTAGAATGTAGAGACCACCTGACTCACGTCCTCTACTAATAATCTGCTTCGTCGCAAGATCCTGAAACAAACATTGGTCAGGAAAAAGGAAACATAACAATTTAAGGTACGAGTAAGTTTTCTAACATAAAGTAGATTAAAAGAGAATTTTGGTAGACACAAAACAGATAACAAAGAAATTGACGAAGTCGGGTTCGCAGTTCCAGAACCCATGACACAAGAAGTAGAATTATCAGCTAAAGTAACAGTAGAGGAAGTGAAATTAGACTGAAAAATAGATAGAATACTAGAATTACCTGTCATGTGATCTGTCGTACCAGAATCAATAACCCATTTGGATGAGGAAGACACAAGGCATGTAGTGGATTTACCTGACTCAGCGATCCCAGTGATAGGGGAACTGGTAGGCTTTTGAGATGCCTAATACTGGAAAAACTGTGCAAAATCTTCTGCAGATACCAAAACAGTTTTCTCAGAAGAAGATACTGTAGAATCCTCTGCTGCTATATTTGCCATCTGTGATCGTTGATTTTTCCTCTGAAGTTGCGGACAATTATATTTTCTATGGCCAGGCTCATGGCAATAATAACAAATGACTCCTCTTGAGTCCTGATTAGAACTAGCCTCCCCATTACGCTGATTACCTCTGTTGCCTGTAATTCCACCTCTACTTCCTCTTCTATTACCCTGTTGTCCATTTGGATTACGGCTAATAAGAGCACTATTGGCAGGCTGTGAAGATTGAGTACTCTCTGTACGAAGGACCCGTGTGAACGTTTCATGCAAAGAGAAAATCTCAGAACTGGAGAGAATCTGAGATTTAGCAGTCTCATACTCTGAACGAAGGCCTGCAAGAAAACTCATAACAGCCAATTGCTCCCGTTGGGCCTGCTGAACTTTCATATTAGGACTAAAAGACAACAATATATTAAGTTCCTCATATACCTGTTTAAAATCCATAAAATAAGTCGTGAGAGACTTATCATCTTTCTCAGCATGGTAGAATGCGTTACAAACATCATAAATACGGGAGATATTCCCTTTACCAGAATACAGAAAATCTAAGTAATCCATTAATTCCTTAACAAATTCACAGTGATTAATTAAACTAACTACCTCACTGTGAATCAAGTTCCGAAGCTGCAAAAACAACCGAGCATCCTCCCTTAGTCAAGTTTGCCGTGTATCATCAGTAGGTGGGTTCTTAGTAAGATGATCATCCTTATCAATGCTACGCAAATAGACCCTAACAGTCTTACTCCACTCTAGGTAATTCGAACCATTAAGTTTGTGTTCCGTGATCTTAGTCATCACCAGAATCACATCAGAAATAACATTCTTATTGTCTGCCATTTGTTGAGACAAAGAAAACTAACTGAAACACTAAGCCAAAGTGCTTACAACCGCAAAATAATCCAAAATCACAAATCAAGCAAATACCAAAATAGGATCTAAGAGCCAAACCTCAGAAGTCCTTTAATGGTGTACTGGATCAGGCAACAGCACACAGTGGTGGAATGAGGGGGTTGAGGCGACGCCGGCGATGCTGATCGGAGTCGAAACGGCCAGCGGACGCACCTCCACTAGCCGGCGAGTGAAGAAACGGCAAGAACCTGAGTTGGGCGCGTGAGCCTCACGCGCTTGGATTCCAGCTACCGGATCAGAGGGGACGGCCAGTCAGCAGTCAAGGTCTCTCTTGAGCTGGGTGGTGAGAACAAATAGTCACCCTAGATAGATGATCTAGGGTAACAGTGGGTTTCAGTCCAGAAACAAACGGGAAAGAAATTCCAGATTTAGATTCCTTAATGAATCTGCTCTGATACCACGTAGAAAGAGATGATTTTCCTTAATTTCAATTAATCTGTACATGGGTATACATATACAATTGATTCCTATAATTGTGTTCTACTAATTAGGAAGAAATCCTAAACAAGAAATCCTAAATAGGAATACAGAATACAGAATATGCAGAGAAATAATATAATAATTGACTTTCCATAACATCCTTTTTGCAGAATTCTGTTATTCAATTTAGCTTTCTGGATTTTGAAGCATTTGCATGCAAACTGCATGTATTCAGCCGTTTTTGTTTTATAGTTGCTGCTTTAGTTATCATATCGTTTTGAAGTTTGATGATGATAAATTTATAGTTGTAAAATTTATTGTGGTGTTGTAG**GTTCTGCGCATTGGACTGGCTATGTTCTTGCATTATCCTTGGGCCTTACTTCTATCTTGAA**GTAAATATCAATCCTAGAGTCTAATATCTTCTATTAATTGTCTTTTGTGCTTCTTTTTTTCCTCAGTTCTTTCCTTTTATTTTTTCTTGTATTGCTTTTTAAGCAGTGGTTGTCTTGAACTTTTTGGTTCACAGTTTTACTAGAAATTCTACCAAAATAGGATTAACATGTGATCCAAAAAAAGTTTGGGAGACAATGAAAGGCATCCATAACCATGTTTTTGCTTACCATTTTTTGAAAAAATAATAGGCATTGATTGGGACCTTTCTTGTGTATTTTGTTCACAACTTCAGGATTCTAAACCTCAATTCCTGGTGCTGTTTGTCTTTCAATATGTTGGGATAACTTTTTCTGTTAAGCTTCTCATTTTTATATTCATTTGAACTAATTATTGTATAACATGAAGGCTTAACAGAAAAAAGAAAGGGATAAAGAAATTCTAGATTTTCATTGATTATGTTCCTATTTTTCTATCTGATTGAGTCATAAATAGCCATGCCTTTAAGATGTAAAGCGATTGCAATTCTACTTTGTTGATGTTCATAGGTTCAATTTTCACACACTCAAATGCATATATGCAGATACACTTACCCTACTTGTCATTTAGAGATTATTTCCCAATCTGCAGTTATCTCATTTCAGTTGGTGACATTTTAACCATGATAGTTGGTTGATGGCTTGATGCCCTTTCTAATTTTGCAG**ATCTTTCTTAGATACACAATATTCATTTCATCTTGCAAAGTTGAAGCTAAAGCTACGATCTGGTATTATGACTGTAATTTATCAGAAG**GTACATATATCATCTGTTTCTCTTAGGGTTTTAAACAGGAATGCATATAAGCCAATTATTTGTACTATTATTTCACTGTCTTTGGACCAATGTGTTGACAAGTTTACTTGGAAGCTAATTAAATTAATGCCTGTTGAATTAAGTAGGGTATTTTCAGTCACAAACTTAGTTTGCAAAGACAAGCTGAATCAAAATGTTTGGGTAGTTAAATTTTGGAAACGTGAATTCTGCACTATGTGTGATGTTAAGGACTAGTTTTTATGCTGCTGCTGTAACAAAATATTTCTGAAATTCTTTAATGGTCTCAAGTTTCTTGTCAATCAAAATCTATATTATGTTTATATCATAGTTTTTTATAAGAACTATGAATATACGGTAAAGCATGCTAGCAATGAAAAGACAAATGTTCATAAAAGGAAATAAATTTTATTACATTCTATTTCACTCCACCATGACCTGACAAATACGGTAGGATTACATTATCTTTAATAATCCAAATTGTTCTTAAATCTTCTGTGGAAATTCAATAATTCTTTAATGATAACAAACTGATCATTGATTGGTTGGGTTGATTCTTACTGTCAATTTCGGATTGACGTGGCACCAAGTTATGTTCTTGTTAAATTTGACATCTTGTTATTCTATTACTTAATAATTGCTTAGTAATCTTATCTTGTTAAAAATTGTTCACCATATAGCACTTTATGCCTTTTGCTAAGCATTGAATATCCACAATTCCATATTGGCACGAGGTTTGAAAATTTTGTCGTAATTTATAGCTGGAGATATATTAAATTTAATTAACTAGTAGCATTGTAATTTGATGCTTTTCATTTGGCTCATGTTTAAGTTTGCTTGCTGTCTTACTTTGAGGAATGTATATTTTAACTTTATTCTTTGTTTACATATGATGCTCTGGGACCTCTCATCTTCTGGTTTGATAAATGATAACCTGTTTCTGGTTTTTCTTTAGTGACGCAGGTCTTCATTTTGCTTTGCCCTTTATGACTTGCCACTGCTTTATCATCAGTATTACATTCATTAATACTTTTTTTTTTAG**TGCCTGTGTGTTACCCTAGCAGAGCGATCCAAATTTTCTGAGGGGGAAATACAGACATTTATGTCTGTAGATGCTGATCGAACTGTCAACTTGTGTAACAGTTTCCATGAGATTTGGGG**GTAAAGACGCAATATTTTATTACATATGAAAGAAATTTGGAGCTACCAACTTTCAAGTCTGCAATTTAATTAGTTGTTTTAAAAGATCATCATCCAGTTTCCCTTTGCTTTTATAAAAAGTTCTATAAGGTTCAATGGTGGAGCAAGAAATTACTGTTGCATTTGAAAGTTTGTACTGTTACTAAAGTTATTGCTTAATTTTATTAAGTTCATCATTGCAATGCTCTGACATTGTTATTGATTTTATTATTGCCACTAATGTTGTTCCATTAATGTTGCTGCAGTACTAAATGGTGACAAACTTTCTCTATCTATTCTATTATTTTCTTTTTCTTGTTGTGTAAATATTTTCAGCTTTTGTTGTATTCTAGAAATTGATCATATTGCATCATTTTGTGAAAATTAATTATAAACATCAATCGATCATATGCAACTGCTTCTCAATCAAATGGCATAGAAGCTGGAAAGAATCTATAGCTGCATATTAAGGCACCAAAGGAAGAATGCCTAGTAGGGTTTCCAAAATATGTTTTTCCATGGAAAACATGTGCTTGCTGAATGATTGTGCAGCACTACAACACAATAACAATAACAACTAAACCTTAATACCAAACTTGTTGGGATCTACTATATGGACCATGGTTTCAAATAACAACCATTATTTAAAAGTTTTTTAGCCTCTTGTTACCGTTACACTTGTTATTTGAAGGTAACAGAAAAAATACACTTGTAATGGTTGGAACGGTCTTTACGTGTTACGTAACGGTGATAACTGTTATTTACCATTAAGTAACGGTAAGGGTCCGTTATCTCACTGGTTTTTTTTTTTTTTTGCTCCAAATGGGGGTAGTAATTCTTCCTCATGATTTGTTTTAGGTTTAGGGAGTCTTAAGTTGCTATGTCTCACTTTCAACTTTGCATATAAAATTTTCTATCTTTCTACAAATCTTTCACTTTTTATCTATTGAGCTAAGATCATAAACTATCTTTCTAGTTTTACATATCCATATCTTGCAAAAGATGAGTTAGTTATTTATTTTTTGGTTTTTTATTTATTTGTTAAAGAAAATTATGTTTGATGCTAGGTTTTTATTAAATTTATAGCATGAAGCATTAGTTTGCCAGATCTATACGTTATTTTTTTTTTCCTAATTTTGAACTATTTTCAAAATTAATTTTAGTAAAATATATAAGTAATTAGAAAATAACAGCAAGAAACTTTTAGGCTTCATTAATGTTTGTTAAGTACAAGTATAATGTTAGTTCTTTACTATCTACATTTTATTTTAGTTTTAAATTGATTAATGCTAATGATGTCTTAGAATGATAGATTTAGCATCTTTATTTGATATATTTATACTTATTTAGTTGTATATCTTAGTTTTACTTATATTTTTAAATATCTATTCATTTTGTGAACTTTGCAAATTTTCTATAAAAAAAAAAAAATTGTGTAGTGATAAGCCATTACCATTATGTTACGTCCATTACAGGCCTATTTCTGTTACATGCAAACGCAACCATGAATGCAAACGCTACTGCTACTGTGATTTAAATCCATGATATGGATTCTTTTTTGTCCTTTAGCTTTGTTTGATACCAATTCTGCATCAATATTCAAAAATTATAGATCTTTTACACTATTGCCCTTCACATCATTTTAGGTCTCACCTTTCTCCTCTCACTCTTTCCACCACTAATTTATCAGGCCTCTGTACTAGAGCATTTGATTATCTACGTTAGACATGATCAAACCATTTTAATCTACACTCTCATTTTTATTCTCAATGTTTGCATTCATTCTCTAGCAGATGAGGTTGTTCCTACTCTTATCGGCTATCGTAATACTAGACATTCATCTTAACATTCGCATCTTTGCCACACTCATCTTGTGGATATATTATACCTTAGATGCTCAATATTCACTCATACAACATAGTTGGCTTGATCGTTGTTCTATAGAACTTGCCTTTCACTTTATCAAGGATCTTATGATTGCATAATACACCAGTCACACTCCTCCATTTCATCTTGTGTAGTGTTAAAAACTTTTAAAATTATAGCCCACTAAAGGCTTTAACAAAGGTGACAATAATCTATTTTATCAGACTTGTATGCCTCTTAAGACCATAATTTTTTTTCTCTTTCTCTTTCTCTTGCTATATTTTGTTGTGGTTAACTTTTTCTTGTTAAATGAAACATTTTCATGAAAATGAGTGAAGATAGTTTCAAACAGCTAAGTAACTTACTCATTTACTGGAAATGACTTAATTGACAACTTAAATAGGTGAATGCAGACTAGCTTCTTAATGAGCTCATACTTAGTAGGACGGATTTTAATTGTTTTTCTTTTTATGTAGCATTGACTGAGAAAATGGGGCAAACTATTTCTGTCAGAGAAAAAGTGTGAATATTTTCTTTTTATACAAATCTATAGATTTATCAATAGTTTTTGTCAAAGCTATGGTATTTTTTTTAATTTTATTTTCTCCATATTTTGGGAGCTATGGTAATTTTCTGGTAGCATCAAATTTGAATTTTTAGTGCCTATATGAAGAGAGCTTTGTTAAATACTATGAACAAATCAAAATCTTGTGATTTTTTCAG**CTTGCCTTTACAAATTGGCGTGGCACTGTACCTTTTGTATACACAAGTCAAATTTGCTTTTCTTTCTGGATTGGCAATAACCATCTTACTGGTACCAG**GTATTTCTTAATGTCTCTTATTGTCTAACCTTTTACATGAGATCTACAATATACACTTTTGTATTGAAGTGAATATTTTCTGTGTATTTTCTTGAATGAGATACAAGGTATTTATATACAAAGAGTCCTATAATTAAGTATTCAAATTGGGAAGAGATCCCAATAATTATGTTAACTAATTAATAAAGAATATTATGTACATAATTATAGGATTGACTTTCTATAACACTCCCCCTCAAGTTGGAGCATAGATATTAATTATGCCCAACTTGTTACAAATGTAGTCAACTTGTAGAAAGAGATGATTCCCCTTAATTTCAATTAATCTGTACATGAGTATATATATCCAATTGATTCCTATAATTGTGTTCTACTAATTAGGAAGAAATCCTAAATAAGAAATCCTAAATAGGAATACATAATACAGAATATACAGAGAAATAATATAGTGATTGACTTTCCATAACACTCCCCCTCAAGTTGGAGCATAGATGTTAATCATGCCCAACTTGTTACAAATGTAGTCAATCCTAGCTCCATTCAGAGCTTTTGTGAAAATATCTCCTAACTGCTCTCCAGTTTTGATGTGTCCTGTTGAGATGATCTGTTGTTGAATCTTTTCACGAACAAAGTGACAATCAATCTCAATATGTTTGGTCCGCTCATGAAACACCGAATTAGAAGCAATATGGAGAGCAGCTTGATTATCACACCAGAATTTCGAAGGCAGGGAGGTCTTAAAACCTGTCTCATCTAGTAATTGAAGTATCCACATTACCTCACAAACTGATTGTGTCATGGCTCTGTATTCCGATTTAGCACTAGATCGAGAAACTACACTCTGCTTCTTGCTTCTCCAAGACACCAAATTTCCTCCAACAAAAATGCAATATCTAGTAGTTGACCTCCTGTCAACCTTAGATCCAGCCCAGTCGGCATCTGAAAAATATTCAACATTCAAATGCCCAAGATTACCATATAACAAACCTCTTCCTGGAGCGCCCTTCAGATAACACAAGATTTGTCCCAAAGCTTCCCAATAAGCAACAGTTAGGGAAGACATAAACTGACTTACCACACTAACGGCATAAGCAATGTCAGGACGAGTGACTGTAAGGTAGTTCAATTTTCCTACCAATCTCCTGTATCTCTCTGGATCTTCAAACAACTCATTATCCCCTGCTAACAGTTGTAAATTTGGAGTCATTGGTGCACTACAAGGCTTAGCACCTAATTTTCCTGTCTCTGTCAATAGATCGATGACATATTTTCTTTGAGACAAGAAAATACCCTTCTTACTTCTCATAACTTCAATACCCAAAAAATACTTTAATAATCCCAAGTCTTTGGTCTGAAACTGGGTTTGGAGGAAGGTTTTAAGAGATGAAATACCTGCAGAGTCACTCCCAGTGATGATAATGTCATCCACATAGACTACCAGGAGAATTAGACCAGCCTCAGATTGCTTATAAAATACTGAGTGATCACACTTACTCTTTTGCATACCAAATTCCTGTACTGCTTCACTGAATCTCCCAAACCAGGCCCTAGGACTTTGTTTCAAGCCGTAAAGAGACTTTCGAAGCCTACAAACTTTACCCAACTCCCCCTGAGCAACAAAACCAAATGGTTGCTCCATATACACCTCCTCCTGAAGATCACCATGAAGGAAAGCATTCTTGATATCCAATTGGTGCAGGGGCCAATCATATGTAGCTGCTAAAGAGATAAACGAGCGAACAGAAGTAAGTTTAGCTACAGGAGAGAAAGTGTCAGAGTAATCAACCCCATATGTCTGAGCATATCCTTTTGCTACAAGGCGTGCTTTTAACCTAGCTACAGAACCATCAGGATTTACCTTTACTGTAAATACCCATTTGCAACCAATAGCTTTCTTACCAGTGGGCAAAGGCAACAGTTCCCATGTACCATTAGCATCTAAAGCCTCCATTTCCTCTTTCATAGCAGCACACCAGCCAGAATGAGACAATGCCTCACCAACAGTATTAGGGATAGGAACAGAGTCTAAAGAAGTAACAACACCGAGAACAAGAAGACAATTGATTATAAGAAACAAAAGAAGAGATAGGGTAAGTACATGAATGTTTACCTTTACGAAGAGCAATGGGTAAGTCTAGATCAGAATCATAATCAGTATGAGGTACAGGATCTCCCAACAAAGTAGCTGGTAGAGGATCTGAGTCAGGAATCTCTAATCTCCTGGAATAAACATGAACAACAGGAGGTCGAGTAGGTCTAGAGACAGAAGTAACAGGCTGTGGGAGAGGACTAGACATTGGTTGGACAATATATATTAAGAGATCATTCTCCTCCCCCTGACTCTCATACAAAGATGATTGAGGAAAAAATGGAGTGGACTCAAAAAATGTGACATCTGCAGAAACAAGATAACGATTAAGAGTAGGAGAGAAACAGCAGTACCCTTTTTGCAGCCGGGAGTACCCAAGGAAGACACATTTGAGAGACTTTGGATCCAATTTAGTAACCTGTGGACAAACATCACGCATAAAACAAGTACAATAAAAAATACGGGGTTCAACAGGTAACAAAGATTTTGTAGGAAACAAAGCAGTATAAGGAATATCCCCATTAAGGACAGAAGACGGCATACGATTGATAAAAAAACATGCCGTAGAAACTGTATCCGCCCAAAAGTATTTAGGAACTTTCATTTGAAAAAGAAGAGCACGAGTTACCTCAAGAAGATGCCAATTTTTTCTTTCGGCCACGCCATGTTGGGATGGGGTATCAACTATCAACACAGGAACACTGATGAAGAATGCCATTTTGTGTCATATAAGACTGAAATTGTGCTGAAAAGTATTTTTTAGCATTGTTACTTCTTAATATGCGCACAGAAATATTAAATTGAGTTTTGATTTCATTACAAAAGGCACAAAAGATAGAAAACAACTCAGAACGATTCTTTATTAAATATAACCAGGTAACTTGAGAGTAATCATCAACAAAAGTAACAAAATAACGAAATCCAGTTTTAGAAGTAACAAAACAAGGACCCCAAACATCAGAATAAACTAACTCAAAAGGAGATGAAGCCCGTTTATTGACTCTAGACACAGAAGGCAAACAATGATGTTTTTGCAAACTAACACGACTCACATTCTAGTACTGATAAAGACTGAAACTGAGGACACAACTTCTTCATGGTAGACAAAAAGGATGGCCCAATCTACAATGAGCTTCAAGAGGTGTTAAGGTACTGGAGCAAACAAGCGACCGCGGTACATGATTTTCCAGAATGTAGAGACCACCTGACTCGCGTCCTCTACCAATAATCTGCTTCGTCGTAAGATCCTGAAACAAACACTGGTCAGGAAAAAAGGAAACAGAACAATTTAAGGTACGAGTAAGTTTACTAACAGAAAGTAGATTAAAAGAGAATTTTGGTAGACACAAAACAGAAGACAAAGAAATTGACGAAGTCGGGTTCGCAGTTCCAGAACCCATGACACAAGAAGTAGAACCATCAGCTAAAGTAACAGTAGAGGAAGTGAGATTAGACTGAAAAGCAGATAGAAGACTAGAATTACCTGTCATGTGATCTGTCGCACCAGAATAATAACCCATTTGGATGAGTAAGACACAAGGCATGTAGTGGATTTACCTGGCTCAGCGATCGCAGTGACAGGGGAACTGGTAGGCTTTAGAAATGCCTGATACTGGAAAAATTATGCAAAATTCTCTGCAGATACCAAAATAGTTTTCTCAGAGGAAGATACTGTAGAATCCTCTGCTGCCATATTTGTCATCTGTTATCGCTGATTTTTCCTCTGAAGTTGCGGACAATTATATTTTGTATGGCCAGGCTCATGGCAATAGTAACAAATGACTCCTCTTGAGTCCTGATTAGAACTAGCCTCTCCATTACGCTGATTACTTCTGTTGCCTGTAATTCCTCCTCTATTTTCTTTTCTATTACCCTCTTGTCCATTTGGATTACGACTAATAAGAGCACTACTGGCAGGCTGTGAAGATTGGGTATTCTCTGTACGAAGGACCCGTGTGAACGTTTCATGCAAAGAGAAAATCTCTGAACTGGAGAGAATCTGAGATTTAGCAGTCTCATACTCTGAAGGAAGGCCTGCAATAGAACTCATAACGGCCAGTTGCTCCCGTTAGGCCTGCTGAACTTTCACATCAGGACTAAAAGGCAACAATACATTAAGTTTCTCATATACCCGTTTAAAATCCATAAAATAAGCCGTGAGAGACTTATCCTCTTTCTCAGCACGGTATAATGCCTTACAAACATCATAAATATGGGAGATATTCCCTTTACCAGAACACAGAAAATCTAAGTAATCCATCAATTCCTTAACAAATTCACAGTGATTAATTAAACTAATTATCTCACTGTGAATCGAGTTCCGAAGCTGCAAAAACAACCGAGCATCCTCCCTTAGCCAAGTTTGTCGTGTATCATCAGTGGGTGGATCTTTAGTAAGGTGATCATCCTTATCAATGCTACGCAAATAGACCCTAACAGTCTTACTCCACTCCAGGTAATTCGAACCATTAAGTTTGTGTTCCGTGATCTTAGTCATCATCGGAATCACATCAGAAATAACATTCTTATTGTCTGCCATTTGTTGAGACAAAGAAAACTAACCGAAACGCTAATCCAAAGTGCTGACAGCAACAAAATAATCCAAAATCACAAATCAAGTAAATACCGAAATAGGATCTGAGAGCCAAACCTCAGAAGTCCTTTAATGGTGTACTGGAATACTCCAGATAATTCGAACCATTAAGTTTATATTCCGTGATCTTAGTTTTCACCGGAATCACATCAGAAATAACATTCTTATTGTCTGCCATTTGTTAAGACAAAGAAAACTAACCAAAACGCTAATCCAAAGTGCTTACAGCAGCAAAATAACCCAAAATCGCAAATCAAGCAAATACCAAAATAGGATCTGAGAGCCAAACCTCAGATGTCCTTTAATGGTGTACTGGATCAGGCAACAGCACACATTGGTGGAATGAGGGGGTTGAGGCGACGCCGGCGATGTTGATCGGAGTCGAAACGTCCGGTCGGCGGCCAAGGTCTCTCCTGAGCTGGGTGGTGAGAACAAATAGTCACCCTAGGTAGATGACCTGCTCTGATACCATGTAGACAGAGATGATTCTTCTTAATTTTAATTAATCTGTACATGGGTATATATATACAATTGATTCCTATAATTGTGTTCTACTAATTAGGAAGAAATCCTAAATAAGAAATCCTAAATAGGAATACAGAATATAGAATATATAGAGAAATAATATAGTGATTGACTTTCCATAACACAACTAGAGCCCTATTCAAAGCTTTTGTGAAGATATCTCCTAATTGCTCCTCGGTTTTGACATGCCCTATTGAGATGATCTTCTGTTGAATCTTTTCTCGAAGGAAATGACAATCAATTTCAATATGCTTAGTACGCTCATGAAATACTGGATTTGAGGCGATATGAAGAGCGGCTTGATTGTCACACCATAGTTTGGCAGAAGATAAGGTCTCAAGACCCACTTCTTCCAGTAACTGACGTGTCCACATAAATTCACACACAGCTTGGGCCATAGCTCGATATTCTGATTCAGCACTCGAACGAGAAATTACAGTCTGTTTCTTACTTCTCCAAGATACTAAGTTTCCTCCAACAAAGATACAATATCCTATAATGGATCTTCTATCAATCTTTGAACCTGCCCAATCTGCATCTGTAAAGCACTCAATATTTAAGTGCCCATGATTGTTATATAAGAGACCCCTACCTGGAGCACCCTTCAAGTAACATAAAATTTGCCCCAAAGCTTTCCAATGGGTAACAGTAGGAGAGGACATAAATTAACTTACAATACTAACTGAATATGCAATATCTGGATGAGTTACTGTGAGATAATTAAGTTTGCCAACCAATCTTCTGTACATCTCAGTGCCTTCAAATAGTTCACCATCTCCTGCTGTGAGCTAAAGATTGGGAGTCATTGGTGCACTATATGGTTTGGCACCTAATTTACCTGTTTCTGCCAATAAATTAAGGACATACTTCCTCTGAGATAAGAAGATGCCTTTCTTATACCTTGAAACTTCAATGCCTAAAAAATATTTCAATAAACCCAAATCTTTTAGCTGAAACTGAGTTTGAAGGAATGACTTGAGTGAAGAAATGCCTGCAGAGTCATTTCTAGTAATGACAATATCATCAACATATACTATAAGTAGGATTAGTCCAGCTTTAGAATGTCTAAAAAACACCGAATGATCAGACTTGCTCTTTTGCATACCAAACTGCTGAACTACCTCACTGAACCTGTCAAACCAAGCTCGAGGACTCTGTTTCAAGCCATAAAGGGATTTTCGAAGTCTACAAACTTTGCCTAACTCCCCCTGAGCAACAAATCCTGGTGGTTGCTCCATATAAACTTCCTCCTGGAGATCACCATGAAGGAAAGCATTTTTGATATCCAATTGATGTAAAGGCCAATCATATCTAGCTGCCAAGAAAACAAATAAGCGAATAGAAGCTAGTTTAGCAACAGGTGAAAAAGTGTCAGAGTAGTCAGTCCCATAAGTCTGAGCATATCCTTTAGCTATAAAGCTAGCCTTGAGACAAGCCACAGTACCATTGGGGTTCATTTTCACTGTAAAACCCTCTTTTACATCCAATAGCTTTCTTATCTGGAGGCAAATGCATCAATTCCCAAGTACCATTAGTATCTAAAGCCACCATTTCCTCTTCCATTGCGGCACGGCATCCAGAATGGGACAATGCTTCAGCAACAGTTTTAGGAATGGAAATAGTGTCTAGAGAACTAACAAAACAACGAGATGAAGGAGACAATTGATCATAACATACAAAGGAAGAGATAGGATAAGTACATTGACGCTTACCTTTGCGAAAAGCAATGGGAAGATCTAATTCAGACGGAGGAGCAGTGAATGGAACTGGATCTTCTGACTAAGAAGCAAGTGGAGGATCTTAGTCAAGAGTCTCCAATCTCCTGGAATAAACATGAACAACAGGTGGGCGATGAAGTTGACCAGGTGCTGGTGTAGGAGAATTCTGAGGACTTGGCTGAGGACTTGACACTGGATGGACAGAATAGACTAAGAGATCATCTTCCTCCCCCTGACTCTCATAACTAGGAGAGGGAGGAAAGAATTGAGTAGATTCAAAAAAGGTAACATCAGCAGACACAATATAACGATTAAGGGTTGGAGGAAAAGATTTGTACGCTTTTTGAAGTCGAGAATAGCCAAGGAATAGACACTTAAGAGACTTGGAATCCAGTTTAGTAACCTGTGGACGAACATCTCTCACAAAACAAGTACTACCAAAGATACGAGGTTCAATAGGAAATAAAGATTTAGAAGGAAACAAAATGGTATAAGGAATGTCACCATTAAGCATAGATGATGGCATACGATTAATCAGGAAACAAGCTGTAGAAACTGCATCATATCAAAATTGTTTAAGGACTTTCATTTGAAAAAGGAGTTGCCGTGCTACCTCAAGAAGATGTCTATTTTTTCTTTCCTCAATCCCATTCTGCGATGGAGTATCAACACAAGATGTTTGATAAAGAATGCCCTTTTGTGACATATAGGCTTGAAAATTGCCAGAAATATATTCCTCAGCATTATCACTTCTCAATATACGCACAGAAGTATTAAATTGAGTTTGAATCTCAGCACAAAAGGCACAAAAAATAGAAAATAATTTCGAACGATTCTTCATTAGAAACAACCAAGTAACACGGGAATAATCATCAACAAATGTAACAAAATACTTGAAACCAGTTTTAGAAGTAATAGAATAAGGACCCCAAATATCAGAATGAACTAACTCAAAAGGGGACGAAGCCCGTTTATTGACTCTAGGTGTAGAAGGTAAATGATGGTGCTTAGCAAACTGAAATGACTCACACTTTAAGACGGACAAAGACTGAAATTGAGGACATATTTTCTTCAAGGTAGCCAAGGAGGGATGGCTCAAACAACAATAAATTTCGAGCGGAGTTAAGGTGCTGGAGCAAGTAAGAGATCGCGGTACATATTTATTCAGGATGTAGAGACCACCGGACTCATATCCTCTACCAATAATTTGTTTCGTCGTAAGATCCTGAAACAAACAATAATCAGGAAAAAAGAAAACCGAATAATGTAAGGCACGAGTAAGTTTACTAACTGAAAGCAAATTAAATGAAAATTTAGGTAGACATAAAATAGATGATAAAGAAATAGATGACGATGGATTTGCAGTACCAGAACCCATGACACAAGAAATAGAACCATCAGCTAAAGTAACTGTAGAGGGGTAGGATGAGACTGAAAAGATGATAGAAGACTAGAATTACCTGTCATATGATCTGTAGCACCAGAATCAATGACCCATTTGGATGAGGAAGATACAAGGCATGTAGTGGATTTACCTGACTCAGCAATAGCAGTGACAGGAGAATTAGAGGCTTTAGAGATGCCTGATATTGAGAAAATTGTGCATATTCCTCTGCAGTTATTAAGACGGTTTTCTCAAATGAGGGTGCAACTGTAGACTCATCTATTGCCATGTTTGCCATCTGAGACCGCTGATTCTTCCTTTGAAACTTTAGACAATTGTATTTTGTATGACCAGGCTTATGACAATAATAACAAATAACTCCTTTTAAGTCCAGATTAGAATTGGTTTCCTCATTACGCTGATTAATTCTATTGCTTGAATTTCCTCCTTTATTTCTCTGTTGTCCATTCATATTTCGACTAACAAGAGCACCACTAGTAGTTTGTGGAAGTTGGGTACTTTCTGTACGAAGGACACGTGTGAATGTATCATGTAGAGAGAAAATTTCAGAGTTAGATAGGATTTGAGATTTAGCAGTCTCATATTCTGAAGGAAGACCTGCAAGAAAACTCATAACAGTCATCTGCTCTCGTTGAGCCTGCTGGACTTTCACATCAAGGTTAAATGGCAACAAGACATTAAGTTCTTCATATACTTTCTTAAATTCCATAAAATAAGCCGTGAGAGGCTTATTCTCCTTCTCAGCATGATAAAATGCTTTGCACACATCATAAATATGAGAAATATTTTCTTTACTAGAATACAAGAAATCTAAGTAATCCATCAATTCCTTAACAAATTCACAGTGATTAATTAAACTAATTACCTCACTATGAATTGAATTCCGAAGTTGCAAAAACAACCGAGCATCTTTCCTGAGCCAAGCTTGCTTTTGTATCATCAGTGGGTGGATCTTTAGTCAGATGATCATCCTTGTCAATACTTCGTAAATAGACCCTGACGGTCTTACTCCACTCCAGGTAATTTGAACCATTAAGTTTGTGTTCCGTGATTTTAGTCATCAGTGGAATCACATCATAAATAATAGCCTTATTGTCCGCCATTAGATGAAATTAACAAAATTCTAACACAGATTAATAACCAATTCTGTCCCAATCTTCAAACAAATTCAATAAAACTTAGAAACAAAAGAATTCCATGAAAACAGAACCTGAAATTGCTCAACAGTGAACTGCACGAAGGAGAGAAATATTTGAGATCCAAAAGAAATGCCCCAAACTGAACAATGAACCACGGAACTGGAGTCACAAAGTTGAGGTGACCCTGGGACAAGAGGCGCCACTATCGGACGTCACCGGAGAAGAACCAGAGAAAAAAAAATATATTTCAAAAAAAAAAAATTTCAGGCTAAGGAACCTAGGCTCTGATACCATGAAGTGAATATTTTCTGTGTATTTTCTTGAATGAGATACAAGGTATTTATATACAAAGAGTCCTATAATTAAGTATTCTAATTGGGAAGAGATTCCAATAATTATGCTAACTAATTAATAAAGAATATTATGTACATAATTATAAGATTGACTTTCTATAACATGCATCATTGGGTTTAATGCATGTTGGCCTCTCATCTGCATACACCTCCGTGTCAAGAGTGTGTGAGCATGGATTACTCAACCTCAAATTTTGTTTTTCTTTGCATTATGGATACTCATGCAGTACCACAACTTTAATGATGTTGCAGCTTATTATGCAAAAAGTAGATACCTTTCTCCTACTATTGAAATATTTTTTATGCAG**TGAATAAATGGATTTCTGAATTAATTGCAAGTGCCACTGAGAAAATGATGAAGCAGAAAGATGAGAG**GTACATTTTTGTGTTTAATCATTTTCTATAACAACAAAAAGATTGAAAATTTCTGAGATTCAAAAGTGCAGTTGTTATATATGCATGATGTCTTTGAAGCTATGGCATGACATTGACTAGTCTTCAAGCAAATGTAAAGTACTTAAGTGGCCTCTCCTTTTGATGAGTCCCAATGCTTGGTGACTGGTGAGACAGTCACAATGGTCCACCCACATATTTTCCCTGCATTGCAGTTATCTTCTTTACAATAAACCAGAAATGTTCTCTCATCTCCAAGATTTTTACTAGTTTTGAGTGGCATCAAACAGTTCCATATGTACTCTTTTGATTGGCATTTACACTCAAATAGATATGTTCCTTCACAG**GATTAGAAGGACAGGAGAAATTTTGACGCATATTCGCACTTTAAAGATGTATGGTTGGGAGCATCTTTTCTCTAGTTGGCTGATGGACACAAGATCCTCAGAAGTGAAGCACTTAGCC**GTATGGCATTGTACTTAATTCCTGGGTGGTCTAAGAGTGATGCAGTTTATAGGCATTATTCTTTTACTTCAAATGCTTGAACAAATGTCAATCATTATGTTTGCTCTTTCATGTCAG**ACACGTAAATATTTGGATGCGTGGTGTGTTTTCTTTTGGGCCACAACACCAACTCTCTTCTCTTTGTTCACTTTTGGACTTTTTACTTTGATGGGACATCAGCTTGAGGCTGCTACG**GTATTTTGATCATAACCCATGTGATATATTAATAATAAACTTCTTTTTGTTTCTCTATTCAATGTTTATTATTTGTTTTGGTCCATTTCTTAGCAACAACACTATAGCTACAAAAAATATTGATTTCTTTGAGAATCTTGGTTGCAG**GTCTTCACTTGTGTTGCCTTGTTTAATAATTTAATCTCTCCTCTCAATTCATTCCCATGGGTCATCAATGGATTGATTGAT**GTAAGTTGTATGTGTACTGCAATGTTTGAACAAATATTCCTAATGTGGTGATTTCTGATACCCGCATTTTCTCTTTGAACTAG**GCCTTTATATCAACCAGGAGGTTGAGCAGGTTTCTTTGCTGTTCAGAGTACAGACATGAACTTGAACAAAGAGCTGAATCTCCTTCAGTTTTAAAAAATTACCAGTCTGACATTATCTCTGAAGACATGGCTATTATAATGCATGATGCATGTTGTGCTTGGTCAAGCAGCGATGAACAGCAACAGAATTTGGTGTTAAATCATGTGACTTTGTCTGTTCCAAAGGGGTCTTTCATTGCAATAATTGGAGAG**GTTAAAATTCAAACCCATGCCAATTTTTTTATAATTCTATATGCCTTTTACAATTACAGCTCTGAACAGGAAAAAGAAAAGGGGGGAAACCTAGATATAGCATTACTACAGCTTCGAGATAACTTAATACCTAGTTGAACAAAAGATAATGTTATGAATATTAATAAGGGCTGTAATTGTATGGTGAAGGGAGTAGAATAAGTAGATGTAGTTGAGATGAGTTGATAGCTGTATAGGAGTTGTTATAGTAGTTGAAGTAGTTGTTCTAATTGACGGGAACAGTTAGTTAGTGGAAGAGATGATGTATAAATAATTGAAGAGATGTAACGGAGGACTATCTAAGAAATATCAATATTACAATCAATTTTTCTCTCTAATTCTCGCTATCTTCCTTCTTCTCCTATTTCTCTTTCCCTTTCTTCCTCTATCTCTTCCTAATTTCTGTTCTTCTCTGGAATTGAGAAATACTGCTTTGTGACAGATTATAAGAAGATATACATTTTCATTCTAAGTTCTGATGATAACTCAATGAAATACTCTATTTCTACAAGTCCTGTTCTTTGTTCTTCCTTCTTCTTGCTAACATCAACTATCCAG**GTTGGTTCAGGTAAATCATCATTACTGAGTGCAATTCTGGGGGAAATGTGGCTCATCCATGGGTCAGTACATTCAAATGGATCTCTAGCATATGTACCACAG**GTTTATACTCTTATTCTTGGTAGGACTTGCATAGCTTACACCTTTTGATGCAAAATCTTTTCATTGGCCTTTGTTGAAATTGCAG**GTTCCATGGATTTTGTCTGGAACAGTACGTGACAATGTTTTATTTGGGAAGAGTTATGAATCAAAAAG**GTATAATTCTATCATTTTGAAAAGCTTTTTACTTTTCTTGTGCTGAAAATCCTTAATATGTTGCTTTTTTTTGTTGATTGCGAAGATTTTTCTGGCTTTATATGCCAATCTTGTTAGGAAGGAATGAGATAATTGTGGTTCTAGGTGACAATTGTGGCATATTATGCCAAGAATCCAGAAAGGCATTTTCCTGCTTTACAATGGTGGTTTTACTGATTTCTTTGATTTTTTTTTCCGCTACTTGTCAG**ATATTCAGATACTTTGAAGGCTTGTGCACTAGATGTTGACATTTCATTAATGGCTGGAGGGGACATGGCTTATATTGAAGAAAAAGGAGTCAACTTATCTGGTGGACAGAGAACTCGTCTTGCTTTGGCAAG**GTTATATCCACAATATATTTACTTTTTCTGCCTTCTTGATAGAGAGAAGCTTGAACTTTTCTGTTTATTGTGGCTGAGCTTCCTATATTTGTGCAAAATTGGTTTTATGTGTCTTGTTTTTAGTATTTTTATCAATGATCAGAGCATATCTTATCATCTAATGTTATTTTCTTTCTTGTTTTATAATATTTGTTTATCTGATCTGAAAATGATATGCAACAG**GGCTATTTATCAGGGATCAGATGTATATATGCTTGATGATGTCCTCAGTGCAGTTGATGCAGAAGTTGCAAGGTTGATTTTACACAATGCTATTTTAGGCCCTCTTATGAATCAAAAGACTCGTGTTCTTTGTACTCATAATGTCCAG**GTAGTTGCCTTGATTCCTGTTCTTTTGCTTGAATGTTCTCATTTGCTTTTTAAGACATACATTGGAAGAAAAATGATCTCTCTGTCTTTCTTTCTTCTTTTTTTTTTTGGGAAGTTGTTTTATTGTAATCATGTTTTGAATTTTTTCAGTGTTTGGAGTACATAGGAGAATGAGTCAAATGAAAAAAAAAAAAATCCTGGTCAAAGATAAAGCAGAGCCATTTTCCAGGAATGTGAATTCTTGTTTGGAAAAGGAATTTTTATCCAGGCTTTTACAAACTTATTAATCACATAGTAGTTCCTTATTTATACAAGCATTATTATGGCTTTCTTTATTGAAAGAATATTAAGCTTTTCTGAAGTATTTCAGACACCAGATGACCAGAATGATTCCCAAATGATCTTCAATGTTGCAACCAAATTAATAAGCTAATTTATTATTTCAAAAACTTTTGAGTGACAGGATTAGGAAAGCATCTTGCATTTTCTTTTTCTAATAATTTGTAGGAAGAAAAAGATATTGAGAAAAGAGAGAGTTATTGGCTTCTCTATTTCATATTTTCTTTTTATTAAATCCGAGGCTGTATATACAAGACTTCTAAAATAGGAAGATAACTAAAAAGGAATAAAACCTATAAAGTAGGCTAATAATACAGCCCTAATTAAGTGATATACAGCAAATAAAAAGAGAAAAATATTACAGCAAATAAATCTCTAAAATTATGGTAAATGAATCTCTAAAAATTTGCTAATCCCTAAAATCATGCTAACCCAAATCAACTCCCATTTTTCAGCATAATGCTTACCTGAAAAGACATTGTTATGTAAAGATTAGAATATTTGATAGTAAAAATTACTAACTGTATGAAGCAGCTTATGTTGTTGCTAAAAAAAAAAAAGGGCAACCTAATCTGATAATTGAAGTAAAACATGAATTGCAAATACATTGGCTGCTAGAGGAAAAGTTCAACATGGTGAAGATTTGTGTCACCAATCATATATTGTGCTTCTTGCATTTTCTGTGTTTTAAGTATAAACCTCTTCACTGTTGAAAAGGTAATAGCATGAACTTCCATCCACTTTGTACTTGCTAGCACATCTACCCTGAGAAAATGGACAGAAAAAAAAAAAGATGCATTAGCATGACAGGAAAGAGGGAGTGAATGGTAGAAAACAACATAGATATGGGTTTTTGAGTGTGGAAACAAGTTACCGTGTGAAGTAAGGAGAATATCAATGACTGGAGGTAGAAGCTAATCTGCCACAGAATCATCTCTTTCATTTTATCTACCTTCATTTGATTGTCCCTAATGATTATTTCCATTGATATCTGAGGTAGCAAATAAAACTAAGGCTTTAGCTATGGTGTTACTAGATCCAATGGAGTAGTGGTTGTTCTATAACATGTCCAGTTGACATTTGATGCATTGGTTTTGACAATAATAAAATCAGATCAACACAAGAGGGAAGCAAAAATTTAGGTTATTCTAATTCTCACCTCTTTTCGCCTTTTTTTTTTTCCCTCTCTGGGAAAGTCAAAGCATTTTGATTTTTTAAAGATAGTCCTAATGGAGAATGCTATGTTTTTCTCCAACCACAATAACAGCGAGATATCCCAACTGGTTACATCATCAATTTTTCACAAAACTTAAAGGAGCTGAATTTTCAGTTGACACTTGGCTTGCAATTTTAATGATGCTTATTATTAACCTTTGCATGCATAGTGTTAACTTAGGCATCCATCTGGTATCCCTTTTTGTTCCTCTGTGTCTTCTGATCACTGATGCTATCTACCTTCCTATCATGTGGTTTTATGTCATTCTGATGAGCACTTAATGCAGCTTTTGGGTAGCTGCATTGCTACTGGTTTGCTTCCTAACCTATTACTTGATGAGAGGAGGGAAGAACTAGGTTCCTCATTATGAGGGAAAAAGATCTGGAAAGGAAGTATAAGAAGGTAGATTGGAGTGTAACTATATGGTTTCACTTTTATCTCTTTTTCTTTATAATACAATAGCAAGAAGTCCCTACATGAAAACAACTAGAGGAAGGCAAAGTGAATCAGTTCTTTCATTTGATGAGCACAGTGATAAGCACCAGCTTAAAGTTTGGTGCTTCTTAAGAAGACTCTGCTATTTAATTGGCTTGAAGAAAAAGATTCTTAATTATGAAAATGTCTTTCACAGTGTTAATGGTTCTAGAATGATGAGCCTGCCTGAATGGTACGCTGGAACATCTTACATTATTTGTAATAGATAGAAAAAAGGCGATGAAAGACTTTGTTATGCCTTCTCTTTCTTGAATATCCTGTGATAATAGTATAATTGGAACTTGGATTTATTATTTGTTCTTTTCTGGATATCAAAAAAGGCTAGTTATATTAGATATAGTTTCTACAATGGCTGATGGACCTGATGGTTTTGGTTTCTTACCTTGAATGAGTTCTGTGGTAAATTCCAATATCTGGATAACTTCTAATTTATTGATAACCCATCTGAAGTTCCTTAAATGAGCTGATATTGAAAAATGAATTTATTCTCTGTGGCTGTTGTGTCACAACAGACATATTGCTTGGGAAGTGGAAAACTGAGCATGATTGTTGCATGCTATCTAATTGAAAATAGTTTTGTTTCATGTATGAAAAATGTTGTGAACTCAACTTGAAATTAGAATGTGCCTTGTGGATTTGAGTTGGCTGTCATGACCCTTTCAG**GCAATATCTTCTGCTGACATGATTGTTGTAATGGACAAAGGACATGTGAAATGGGTTGGAAGTTCAGCTGACCTGTCAGTATCATCATTCTCAGCATTCTCTCCACAGAATGACTTTGATATATTACCAAATCTTCAAGGACAAGAGCTCAGCAAAAATACTTCTATCGAAGGCAGAAAAAGTTTCAGTTTGGAGGAAGAATTCATACACATTTCAGAGGAAGCACAAGAGATTGTTGAAGTTGAGCAGAGGAAAGAAGGCAAAGTTGAACTTGCTGTATACAA**GTAATAATTGCCGCTTAATATTTCCTAGCTTGGAAGAAATTCCAATATTTTCTAGGCAGGATGGTTAGAGATTTTCCATTTAACTTTCTTGCCGGTTGACAAAAGAAATTTAGTTTGTCATCTAACCTTTTGTATTGTTAACATTCGCAATTAATATTTTTTCCCACAG**GAATTATGCAGCATTTTGTGGCTGGTTCATCACTGTTGTAATATGCCTTTCAGCTATTTTAATGCAAGCTTCTCGCAATGGAAATGATTTGTGGCTGTCATATTGGGTGGATGCAACAGGAAGTAGTCAGGCAGACTACTCGACATCCTTTTATCTG**GTAATTCTTTATCCTACAGGAAAATACTACCATTAGTTATGCAGTTTGGCTGTATTAATTTTATGGGCCCTTTGCTTCTTATCCTATTTTTATTCTGCAAATAATAATCCAAGGAATGATTCTTGCTGCTGATAATATGAAGAACAATTAAGCATGTAACCTGTGACGTATTAAGTTGCCATTGACCAAGTCATAAGCAACAATCTTAAGAAAATTTAATCAACATTTGCGCAAGAACATTTCATAATGGGTATCCCTTTCATCGAATATTGAATTGGAAAGTTTCAATAATATTAATATATGTGTATTTGATTTCCAAACTTCGTATTAGAATTTTTTTTTCAAAGTATTGTGGATGGCCTATAGAGAAGTCTAAGTCTTCTACATTGTTTCTTTTGATTGGTGGGAATACAATTATTGATTGTGATTAAGACTTATTAAGTTTCTACATGATATCAGCACTTTGTTGTAAGATGTTTGAAAAGGCATGGGAAGTGTTAATTAGTTTTAGTGTTATTTAAATTTGCTAATTGGAATTTTTTGCTAGACAATAATGGATTTGCAATTCCGCAATTCAATAAGACCTAAGATTCCTAGATTAAACCAAGTGACTTTATGTAGATGATGTTTTGGCTGTGCAGATGCTTGTTCCAATATTCTTAAACAAATCAAAGAATTAGATTCTTAGTAGAAAGTTACCATGCAATTCTTAAAATGACAGGTACACCATAGTCCATTTTAGTCAACTCCCGACTAAGTGCAGTGTATGAAAAATATTGCTCTTTATGATATTTTAGGCATGTATTTTGTGGTTTTTTTTTTTAAACTCAAGTTTAGATATAAAGTTGTATATTGCAAATTGCCTTTATCTATTCAGTGTGGGATCAGTTCTATTGTTTAAGTGAACATAGAGGGGGGAAAGAGGTAAGGGAGCTTGTCAACGGGAGGTTTATTTCATTCTTCTTTGACTTGGCAATCAATGTTGCAG**GTTGTACTCTGCATCTTTTGTATTGTAAATTCGTCTCTAACACTGGTTAGGGCGTTTTCATTTGCATTTGGTGGCTTACATGCTGCAGTTCAGGTGCACAATACATTGCTCAATAAGATAATTGATGCACCTGTGCAGTTCTTTGATCAAACACCTGCTGGAAGAATACTTAACAG**GTGGGAATAAGATGCTATTTTATGATCAAGTCATTATTGTTTTTCTGATCTTCAACCATCAACATATTTGTTATGCTTTCTAG**GTTCTCTTCAGATCTCTATACAATAGACGATTCTCTTCCTTTCATTCTCAATAGTCTCCTTGCCCATTTTGTTGGCCTGTTGGGAATAGCAATAGTCTTGTCGTATGTACAG**GTAATAACAAAATTCTTTACTTTGCCCATCAGTGAAAAATCCTTTTTTTTATTTCTATTTTTTTTTTACTGGGCTTATATGTATGCAG**GTTGTCTTCTTGCTATTATTATTGCCATTTTGGTTTATTTACAGCAAGCTGCAG**GTATATGTTTGATTTCAGATCGCCAGAGATATATTTCAATTATAGCATCAACTTTAAACTTTTGAAACAAAATGAATATATGCAAAAAAATTATTGTACTTTATTTACTTCTATTATTATATACAAATCAGGAATGATCCCATCTTTGGATTAAGTCAGAAATGTTTAAAATTTATAAAGTTACAACCTAAATTGGACAGATAAAAACAATTTGCATTTTTTCTGTATATGGATTCCAAAATTTAGCAACCTAATATCTTTGCTTCTGCATTATCACTCTCTCCCCATTCCCATTGACGGATACACTTTCAAAAAAGAGGGGTCAACTGACCCCCCTTTTTATAAAAATATTCCATATACATATAAGTATGTGTTTAAATTGACCCCTCATAAATTTAATTATTGAACCCTCTTTGTGATAAACATTCAAATAGATTATTCATTTGTATGACTTACTTGAATCTAGTAGCTCTTCTTTTCACATGAAAATGTTTGGTTAGATTAATTTAATTTTATCCTACTAAATTTTTATATGATAAAATCTTAATACTTGAAAATAAATTTGAAAATTTTATTTTTTATATGTTGATCCCAGGAAAATTTTTTTCTAGATCCACAACTGCTAATATGTTACATGCTGTGTAAACCTCTTAGAACATGCTAAAGACATTAGGGAATCACCAGTTTACCAGGATTAATCCTTCAGTAAGGAAATATAGAATCTTATACATGACGTAGTATTGTATTTTATCTCATTTTCTATGAAATAGATTATATATTCTTCAGTACATGCATAACAATGTTCTTTGTTTGTTAGATTATGACTATAGCTGATTCCTTCCTCAATATTTTTTTTTTTCATTTCTGTAATTTTTCACTGTTCCCTTTATATCTAAAATGGAGGGAAATATGTGAAGTTTAAAGCTTTCTACTCAGTCAGAAGTTCTTCAG**TTCTTCTACAGATCAACATCAAGGGAACTGCGAAGGCTGGACAGTGTTTCTCGTTCTCCTATTTATGCAACCTTTACAGAGACACTTGATGGATCATCTACTATAAGGGCATTTAAGTCCGAG**GTAGACATTTGAATGTATCTCTTTAGAATGATTTGTTGACACTGACAGCCAGTTACATCTATTTCTCTTCAGCTGTCATAAATTAGAAACACAATCATTTGGGGAAAACAAACATTAGAAAGGAAAAATGATGTCCATCACAAGTCTCAAGAAGCTAAGGAAAAGATTTTTAAAGTGATTTTATATGTGTGTCAGGAATATCAATATCAGATGAGCAGTTAGTGCTGTGCAATGAAACCATTTCAAAGTTGTATAGTTTGTGTTTAAGTGATGTCATACTGTCCCATAAAATTTGAATGGGGGGAGTTTATTATCTTCTCAATAAATTTAAGATTTGCTATCCATTTCTAGGTAAATTAATTGCTGCGTGTATGAAACATTTTTTCACTATTTTCAGCATTTAATTGTGTTTGGAGGGGTAAGAGAAATTTCATACCTGAAATAATTTACCATGAATGATTAAATCTTTGTTTTGGAAGTACGTCAAAGTAAACTTAAGATATCAAATTATCGGTGATTACATTTTTATGCAGATGTAGATGAAAGCATCTGCAGGATTTTCTTGTGGCAAACACTAGGGTATTAGACCATAGACTTCAGACAATATAGGATTAGTGCTGGCACTAGAGATTGAATCCATATATTCTCTTCCATTAGAACATATTCGTACTGATATACTGGATAAATATTAATATATATAATTAAATGAAAAATTACATAGGCACTGACTACAGATGGTTGTGAACAG**GACTGTTTCTTGGTCAAATTTATAGAGCTTGTGGCCTTATATCAACGAACTTCTTACTCGGAGATCATAGCAAGTTTGTGGCTTTCCCTGCGTCTCCAG**GTATATATCAGAGGTGAAAGGACTGCCTCAATTTGACTATAAACTATACATTTTCTTAAACACAGGATAATGAAAATGTTCATACAAAAAGCAATCTAAAAGCAATTTAATGTGTGAAATTTCTGTTTTCAATGGGAAACTTTCAATTACTATTTGCACTTGGAAAAAACAGGATCTTATATGTGAACTCTTCTGGTTACTGAAAATGACCAGTAGATTTCTGTTTTTTTAG**TTGTTGGCAGCGTTTATCATCTCATTTGTTGCTATGATGGCCGTTGTTGGATCCCGTGGCTATTTGCCAATTAGTTTTGGTACACCTGGGCTG**GTACTTTCTTCAAACTTCTCTTTTGTATTCAATTTCAGCCCATCACATTTTAGCTTTCACTTTCAGATACCAGCTGCTTAAAGTAATGAGATTGTATTAATTTCGCTTATTCTTTTGAATCCGGATGCATAATTTGATTATTTATTTTCTTTTTTCAG**GTAGGTTTGGCTCTCTCATATGCAACTCCAATAGTGTCATCGTTAGGAAGTTTTTTAACAAGTTTTACTGAAACAGAGAAGGAAATGGTTTCTGTTGAGAGGGCTCTTCAG**GTATCAAGACCACAAGATGCACTCTGTTTTACTTCTCTATTAATTACTTATGCGATTTACTCTTTATAG**TATATGGACATTCCCCAAGAAGAGCTCAGAGGATCCCAATCTCTGAATCTTGATTGGCCATTTCAAGGATTGATTGAGTTTCAGAATGTTACAATGAGATACATGCCATCCTTGCCGCCTGCACTAAATGGTGTCACTTTTACCATTCTGGGAGGGACACAG**GTCAGTGCACATTTTGACCGAACTTTGCATTTTATGACTTTGTTTTGGAGTATAATCTTTCTAGTGGCCAGGTTTTCATGTTGTAAAATTAAATTCAAGTAG**GTTGGGATCGTTGGAAGAACAGGTGCTGGAAAATCCAGTGTCTTGAATGCTCTTTTCCGCCTCACTCCAATATGTAGTGGATGTATTTTGGTTGATGACCTTAACATAACTCACGTTCCTGTTAGAGATCTCCGTGCACACTTTTCTGTTGTTCCCCAGAGTCCGTTCCTATTTGAAGGATCATTAAG**GTACACCTGTGCAATGTAATTTGGTGATCATTTGTCTGGCTAGTGGCTACCATGAACTTTTGGATGTCATACTTCCTTTGATGTTAAAGTAGATTTCACATTGCTGTTCGAAATCATTTGATAATACCAACTTTAAATAATTAGATTTTGCATTGGTTTTGCATGCATTGTGAATTTCTAG**GGATAACCTAGATCCACTCCGGATGAGCAATGACCTGGAAATCTGGAATATTCTGGAGAAATGTCATGTCAAGGAGGAAGTAGAAATGGCTGGCGGACTGGATATTCATGTAAAGCAATCTGGATCATCATTTTCTGTTGGGCAAAGGCAGCTTCTATGCCTTGCACGTGCTCTTCTCAAGTCTTCTAAG**GTAAAAGAATAATATTTTCTGTTGTAATAAGTTGATACTATCTGGTCCAGAAATGGAACCAAGTACTTCTAATTAATTTGCTTGTAAATGTGTTGCAAGTAAGTATGTTTGTTGATATTTTTTGATAGAGCAACTTCTTCTTTTGGGTAAATGAAAATATAATAAATTATTTCAAGTGTTGATTTAATGCTCACATGACTAGGAATTAAACCTAATGATTCACTACAAAAACATAATCTGTTTAATGATAATGATGTCAATTTATTCATTAAACATCAGTGATCAGTCATAGTGGAGAGAGAAGACATTTCTGAGTCTGTATGTGACTATGAGCTGTATGGCTGATTAAGTAGGTTATGAAATCCCTTCTAAGATGTGCATATCTTCACCATCTATAGAAAGATTGCAGTTAGGGCTTGTGCCATATGGAATTTGATAGGTGATATCTCTTTGGTTTTTTTTCCCTTGAATTTCTGCTTATGTTGGTTATTTGGTATGCCATATAGTCTCTTAACTGGTCCAAGTGGAAGTAAAAGTGTTAGAACTTTCTTTTTTTTTTTTTTATTAAATGGTGAAATGAGGAAGAATCTGCTAATACACACACATAAAAGGATGGAAAAAGGATTGCTCATAATGTATAACCAGAGTAGTTCTTCTGTAGACTTCATGACTCTGGAAATGGACTACAGATTTTCCGGAATTTTTAATGAATTTTAGGAAGAGAATATGTCTTATGCAACTGCATGTGTGTTTTGAGATGGCGAATGAGATTACTAGTAAATTCTTGGGTTGTTTTGGATGAATCAACGACGAATGCTAACTGTCAAAATAATTAGGTGCTGCAGAAAGTAAACTTACAGGGAAGAATGCATTTATATTTTCAGGGGAGGGTATGTGTGTTCATTTGAGACATGATTAAAGATTACTAATGCATTATATATTCTCAG**GTACTTTGTTTGGACGAATGTACAGCTAACGTGGACACTCAAACAGCTTCAGTACTACAGAATGCAATATCTACTGAATGCAAAGGCATGACGGTGATCACAATTGCTCATCGAATTTCCACTGTTATGAATATGGACAACATCCTTGTTCTTGACCATGGGAATGTG**GTACGCTTATTTTCTGTCTTTATTTTTTCTTCTTTAATTATATTTATTAAGTAGCGGGCATAATCACTTAATAGTGCCAAGTTTTCCCATGAAGTAATGTGCTATTTCCAGACAGGCCTACGGCACACTGGAGCTGGCTTCCCCTAAGCTTGTTTTAAGACAGTTAAACTTCAAATCAACATATAATGTTGTATGCTATTCTAAGTTCAGTGGAACTGATGGGTTAAAACTTTCAAAAATACCATTTTATTTGTTTACTATTGCCGTTCCTTCTATTATTTATAAAAGTATCTTTCTTGCTACATAGGGATGTTTCCTTGTTACTATAACTGTAGTCATTGTAGTTCAAGAGTTCTATTGCTACTTGGGACTGACTAGTTCACATTGCATGAAAAGCTGAAACTGTTTTCCTGCCTGTGCAG**ATTGAGCAAGGGAACCCACAAACTCTTCTACAAGATGAATTCTCCAGATTTTCAAGATTAGCTAAAGCTTCTACCATGTAATTGCCCAACTACTTGCATAGGTCATTTAGGACATATTAGAATTCAACACGTTTCAGGATGCGAGGCTCTTTGTTAATTGTCAATTATCTTATCCCAAGTTATCAAAGCCAGTTATCATTCTAAACATGGCCAGCTAGCTAGCCATCC**

>HbABCD1 scaffold0412(96357-136726)

**GGGAAGGCTTATTTTCTGCCATGCCTTCTCTACAATTGTTGCAACTAACTGAGCATGGTCGAAGCCTCTTGGCTTCAAGAAG**GTATTAACTTTTCGTCCTTGTTTGCATTTATCGTAATTGGAAGAAAAAAGTTGTCTTCATAAAAATATTGCTTTTTATCAACTTGAATGTGCTGCCACCATATTAGGTACCTATTGTATAATTTGGCTGTTCAACGGTCAATGATTTTATTCTAAACAATTGCTATTATTCATCAGAAATTCATTTGAGATTTGTAAAATGAATGCTTCTGCCATCTCTCATTCAGTTCTTCTACAGTCACTTGTATTTCATTATTCTTGCTGTTGGCTAGTAAAATAACACAGGAGATGAGCTAATGATGTAGTTTAAGACGTTTAAGCAAGTAGTTGAAAATTATGGAAAGGGGAGGGAGGGTTTTATGAGATGCTCATAGGAAAGGCTGAGCACTCTGAAAACCGAATTGGACTGATTTACTTTGCCTTTTTGGTTCAGTAATTTGGTAAAACAGTTTGGTTTTGGTTTGTATTTTAAAAAATTTTCAGTTTTACATTTCGGTTCAGTTTGGTTAGGAAGGTCAAAAACCCAAATAACTGAACCGAACTGATTTTTCCCCTTGTAAAGCTTTCTACACCATTTTGTCCCTTCCAAAAACCATACCTATTTAAATTCCTCTTCTTCTTCTGTGAGACAACCAATAATCTCAACTCCCAACCCTTCCTCCTCAGTTCATGAACCCCTTCCATCTTCTTCTTCTTCTTTCTCATTTCCTCTCCTATCGACTGAGTCATTGCCTTTGCCTATGCTGTTGCATCATGCAACTGACCAAGTCCTCTGGTCCTCTATGATTCAGTTCCTTGTTGCATGTTGATGTTTCAACATACTCTCTTTCATTCTGTATCAAGTTTCCTCGGTGCGTATTCAAGTTTTCCTCGTCTGGTTTCAAGATTCCAAGTCTTCATGCAACCTTGCTACATATTCAAGTTGCAGATTTGTGAAATGTAAATGGTAGATTTGCAAATTTGTAAAATGTATATTGTAGATTTGTTAATTGACGCAATTTTAATTTGAGTTAGTCTCTTAGTTTTCCTGAATTGAGTTTTAATTTGGGTGATTTTGTGTTGTGCTTGATGAATTGAGGAATTTTCGGTTAATTTTGGTGAGGATTTGGGAGGGAGGGTTTGGCTTTGTAATCGTGGACTGGAGTGTTCTGGAAACTGATCGACATCGATTTTTTCGGTTAGGTTTGATTCAGTTTTACTATTAGTTTGGTTTGGTTCACAACAATATGCTTTTCGGTTGTTTGGTTTCTACGGTTCGGTTCAGTTTGGATCCAAACCGACCGATTGCACTTCCCTACTTATAGGATGGTCATGATTAAACATTATATATAATCCAGTAAGTTATTTCAATTAGATTGTGGAAAGGCAATTTTTTATATACTTGTATGCCATAACAAATGGAAGTTACCGTATGTGGATAGTCTTATGTGTTTAGAATGTTGTGAAGCGTAGATATACGGAGGCTCTAGTTAGACAAGTAGAGCACATTGGGTTGGAGGATAGAAAGAAAAGAAGGGGTAGACCTAAACTGACTTGGAGGAGAGTAATACAACATGACCTAGAATTATTACACATTTTTTTGGGTTGGAGGATAGAAAGAAAAGAAGGGGTAGACCTAAACTGACTTGGAGGAGAGTAATACAACATGACCTAGAATTATTACCCATTTTTTAGGATTTAACCCAAAATTTTTTAGAGTAGAGAAAGAGAATCCATATAGCTAACCCTAATAAATTTTTGGGATAAAGGTTTGGTTGAGTTGAGTTGAAATTGTTAGATTTTCATGTAGTGATCACTTTGTAATGACTTTGAGTTAACTGAATAATGGATTAAAGAAAGTATTTAGTAGTGTTTGACAACATTTTTGAAGGTAATCTATACAGTGGGGATTATGGGGACTCCTTTTTTGGTTGCTTTTTTTGTACTTTTTGAGTAAAAGATGGAAAATTACGAAAATGAAGGAGATCTCAAACCATTTTTACTTTCTAACTTGGGCTTTTGGAAATCCCACTTTGTGTTTTATTAGTAATGCGGGCCACTTGCGTTAATGGAATTGTTGGTTGACTCATTAGTAAATTATTATTTGTTCTAATAAAGTAGACATTTTAATATCTCCACTCCCCTGGTTCCCCTCCTCCGGTTCCCCTCCCCCCTATGGAAATCACAAAGGAACATACTTGGTCCTCTTATTTAGTCTTCAAGTTTTGGCTTTGAAAAGGAAAGGCAAATTAATCGAGCCATGCTTTAGTTGCTTTAGTTAATACGAGCAACCATATGCAAGGGTTCATTCTGTATGTTTAGGCCCCACTTGGGTCAATTTTTTACTTTTCAAAAGTACTTTTTAACCAGTTCAGATTTTGTCAAAAAAGAAAATTTAATGGTGAAAAAAGATAACTGGTAAATCTATGAATTTTAGTTTACATGAGGAGCAAAAAAAGGAATATAATTTTTATTTTTTGAGGAGAGTAATGTCTAGTCTATTTATTAGAGTATAGATCCTAATTATACTTGGACAAGTTTACTAACATAAGAATCCTAATTATATTTGGAGAAGTTTATTAAAATAAGAATCCTAGAAACTATAGGAATACTTTTATATGAGGAAATAAAACCTAAATTTTGTTACAAATAATATTACCTTTATAATAGGTATGTAGGAACCTCCACATCAACTACAGCCACCTCACCATCATTATTAGTCATTGGCATTGCCACCATCATTGTTGGCTTATTTATCTCCCTCCTACCAACCAAATAATTATTCATGTGGGCATTGTGGCTGTCTTCATCCTCCACATCCAAATTTGTTGCCATTAGTCAAATTTGTTGCCATTAGTCACTGTGCCCACCTTGCCAATTCTATTGCTGCCACTCAACATCGTCATCTCACTGTGACCATTACTATTATATAAGGAGTAATTAATTCAATCACTATCCAATTTATGTACAATATGTTAGTAATAGAAGGTAGTAAAAATTAGTGATCAAACTGAAAGTGTTTTCCTTTTGGAAATAAATTATAAAACTTGAAATGGAAAACAGAAAACAAAAAGCTGAAAATTTTTTTTGTGCGATTAAACAGACCCTCTTTTTATTAGTTTGCAAACTTCGGTTATTTTGATGTTACTCTTCTTAATTAAAAAAAATCTCAATATTGACATTTTGATTATCATCCATCTGTCATCCACTTGGTTAGCAAACTTTTTTAAAGAATGAATGATACAATTGTTGCTTTTAGTCCTTCCAATTTGGTTGTGCCTATGGCAGATGATTTTCGTTTTTTTCTCCTCAGTAGTTTTTATATTTAGCTGCTCTATTAGATGTCCTTATGGTAGAAGATCATTTATTTACTATTGTGTGCTGCATGCAACTTTCAAAAGCAGTCCGAGTCCTCTGTTTCAGTGCTGAACTGTGGTATATTGGTTATCCAAGCAG**GAAATCTCTATTGTTTGCTGGTGGTATCTTAGTTTTTGGCGGGACTGCTGCATATGTGAAGTCACGGCATGGCTGTAAAAAGTTTGATTCTATTGATCACTACAATGGGCTTAGGGGTGATAATGACAAATCAGACAAGCAGGTTGCGAAAGAAGCTAAGAAAATTATTCAGAAAAAAGGAAGTTTGAAATCACTCCATGTTCTAGCTTCTGTTCTTTTGTCTGAGATGGGCAAAAGGGGCACAAGGGATCTTTTGGCTATGATAGCTATAGCA**GTAAGTCTTTGTTCTATGTGCACTCAAATATTTTGTTTTCATCATCTATTCCTTAAGCTTTTGTGGTAGTAGTACATAAACTGCATTCATCAATTGTCCTTCATAGCTGCATAGGTCATAATTGAAATAAAATAGATAAATACTGGCAAAATTTTCTTACACTTCACCTATGGTTTTAAAATTTTGTCTTTTTCCTTTTTTAGGGTCAGTTCAGAAGTTCCTTTTGCTAGTTCTTTATTAGAAAGAGATATAAAGCTGTACATGTGTTTTCTGGCTCTACCCAGTATCAAGAGTTGGCTTACTTCTATGAATTCAAGTTAATAATAGAAATCCCACTTTGTGAAACTCTACTCGCTGTTTATTGCGGCAACAAATATCTAACAAGTCTCAAATATATAACTAGCTTGTGTCGGATATTATGATTTGGAGCATGTATTACTGGCAAGAAACACATACAATCGTAACTGGTCTCATGTCCAACATTCATATTGTGCAGTGATGCTAGGTTGAGGCATAAAAATGACTTGGCATCACTGTAAATGTTAGTTGGTGACCAGATGCACAAAACCTGATTGGTTGCATATCATGTGCAGACAAGGCAGTGCATTAGTGCTCTCAAGTCTCAATCTTATTCTTCTATCCAAGCTATTAATATGTAAAAAATTGGTAATTGCATTCAACTAAAGGTAAAGTTATTGTTACATTTTGAATATATAGGTTAGGGATCAAAAGATGGCGTAATATCAAAACTGAATATAGAAGATGGTGAAGAGATGAGATGCAGTAGTAGCAATAGAGTACATCTTATTCATGTTGATGGGGCTCCCAATAACATATTTCCATGGGTTGTTTAGATGATGAGGGGTTCATGAGGTTTCATTACTTTTCCTCTTTGGTTGAAGAGGCATAAGAACCCATGTACGATTGGTCGTAAAAATTAGATTTCTAAATTTTGACTGCTCTTTTATGGCCCTACAATAATTCTGAGATTTTGTGGCTTTTTACTTCATAAGCAATGTTTTGCAAGTTATTTGCAGAAGAGTTTTTGGCATCCTAATGAACCATACCATTAATACATTTTTAACCTCAGACAAATCATGTATCTTACTGCTAATGGGTGGAAAAAGAACCTTTATAAGCCCTTTGCCTCCTGACTTTCTTTGCTTTTGCTGCTTAGTTGTTACAAATTCTATTACTGCATCAGGCCATTTTATCCATAATCTGGTTAGTTGATGGACTTTTTGTCTGCAGTGCAGTCCTTTTAAGTTACAACAGTCTGATGGAGCATTGGCTCTTTATTCTCATGTTAGCAACTCAGTGATTGGATGAGCAAACTTAATTGATGATAGTGCAACTTTGATTTCTTTTGGTCTTCTCTTTTGCTGAGAAAGACATGATGATTTCTTTTTTGTTATAGAATGATCAGACTGTCCTTTTACTTCTCCAGATGGAAACCATTATTATATTATTGCTATGTGCTGAACTGTTCCCTTGAAACAAATAAATAGTTAAATAAGTGAAAGATTGGAAATTTGGGTGCTCTTAATTGGGAAATAAAATTTATTAAACAACAAAAGTAACCCAACAGAAGTCGGATTGTGTACCTAGGAGTTGGTTGTTGGTGATAGCAAATGCTGTTTTCAGTGGATTTTCATACCTGTAAGTGCGAGTGTCAGCATTTGTAACTGATGCAAAAGGATTAATTTAGCATATGAGAACCAACATGATCCTCTGTCCTTCAAATTTGTTCATTGATTTATTCATTGCTTGAGTAGAAATGTGCTTTTTCACTTTTTTTCAG**GTGTTGAGAACTGCTTTGAGCAACAGATTAGCAAAAGTACAGGGATTTCTATTCCGTGCTGCTTTTCTCCGACGCGTGCCATTATTTTTCCGGTTGATATCTGAAAATATCTTATTATGTTTTCTTCTATCCACTATTCATTCTACTTCGAAGTATGTAACGGGGACCTTAAGTCTATGTTTCAGAAAAATATTGACTAAACGTATCCATGCACATTATTTTGAG**GTAATCATCATAACTTTTTTATGTTTGTTGGAAAATAACTATTTCTGATAATTATTATTTCTGTGTTCTAGTTTAATTCATTTGGGCTTCATTACATGAAATCTTTTTCTTCCCCCTCACTTTTTGCTATAATTTTATTGCATTGATTAACTTTATTGTTCGCAATTCTCAAATTTGTATCCTTTGAATCTATATTAAATTACTAAATTGCAAGTGCTGATGGATGGCTGTTTAATCAAGGACATGGTTTTGTTATTCTCTTTGGGGAACAATTGTATGTTTAGTAGTGGTACCATAAAACATTTTTATCTAGGTGGACCTTGATTTTGTCATGATTCAGCTTTCAGGTGATTGGAAAAATGATATTGATGATAGTCATAATTTTGTTGTATGAGTTCTCATTCTGTTTGGGCATGTGAAACTTGTCTAAGATTACCTTGGGTGGAAATGTTCTGTAATTCTAGTTTGCCCTCTATTTAATGTAGGAACTAGCATGTGGGAACTCTATGATTTGGTTTCATGTAACAATTTTGCATGTTTTGTGTTTTTTCACAGCCTCATAAGTGTGTGTGAGTGCATGTGTCAACACTTGCATGCCTTTTTGGGTGGTGATGTGACATGCCTAGATGCTAATCTCCAAGATCCTATTCTGGTGAATAACAATGGAACTTGGAATTTATTACAGGACATATGATTTTTATCTCCATGCTTTAGTTTAGAATATGCAGCTTTTACTTTATTGGTGTAGGAAAAAGCTTTTTTTTTTGTTGACATCCTAATGATGCATTGATGCCTTTGTTCAGTGCTTGCTTTGTTTTCTCTGAGAGATTAATCATCCTTTTATTTGGTTCCAG**AACATGGCATACTATAAGATATCACATGTTGATGGTCGGATTACTAACCCTGAACAACGAATTGCAAGTGATGTACCAAGATTCTGTTCAGAGTTGAGTGAACTGGTACAGGATGACTTGACAGCAGTTACTGATGGTCTTCTCTATACTTGGCGCCTGTGTTCTTATGCTAGCCCAAAATATCTTTTCTGGATATTG**GTAATTTTTGGTCAAATCTATTATATTGATCTGAATTCTTGATATTCTTAATGTCTTATATTCTTTTGAATGGCTGCTGATAGCGTTTCAGCTTAGTATGATGACATTGGAGTGTTAACAAATTTCCACTTGACACAATAATGTTCTCAG**GGCTATGTGTTGGGAGCAGGAACCATGATTAGAAACTTTTCCCCTGCTTTTGGGAAACTGATGTCAAAAGAACAACAGTTGGAAGGTGAATATCGGCGGCTTCATTCACGTTTAAGGACCCATGCAGAAAGTATAGCATTTTATGGTGGAGAACGTAGAGAAGAATCACATATTCAGCAGAAGTTTAAGGATCTTGTTAGACACATGAGAGTTGTCCTTTATGACCATTGGTGGTTTGGAATGATTCAAGATTTTTTGTTGAAGTATCTTGGTGCTACAGTTGCAGTTGTATTGATTATAGAGCCCTTTTTTGCTGGCCATCTAAGACCTGATGCCTCTACTTTGGGAAGGGCAACAATGTTGAGCAACTTAAGATATCACACCAGTGTCATAATATCACTATTTCAGTCCCTGGGAACTCTTTCTATAAGTTCAAGACGACTCAATCGTCTCAG**GTAGTTCACAATCAAATTATTCATACCATTGTAGACCTCAAAGGAGGCCTTATTTATTTGTTTACCCTCTCATTTTTGGCAGTTTTTTTTACCCTTTGCCTCCCTTCCCCTTCCTCCCTCTTAAAACTGCTCTTACTTCCTGATGTGGGACCTTCTTGTACCAGGGATTTTAGGTCTATGTATTTTAGGAAGAAAGTGACAAAATTTAATTAGGAAGTAATATTTAATTATTTATGAATTCTACTGTTTAGAATTTCCTTGTTAGTGTTGCTTTGGTATTCCTGGTTACCATTGGCTTAGTATTGTCTTATTTTGGGATTCCTAGTATTGTCTACAAGTACCTGAAATCTTTATTTTATGAAGAAATTTGGGAAATATAATTAGGAAGTAATATTTAATTATTTGGGAATTCTACATGTTCCTAATATTGGCTATCTATTCCTAATTAAATTTAAATATTAGTCTACAGGTACCTGAGGTCTACTTATTCCTAATATTGGCTACCAGGGATTTTAGGCCTACTATGGTAGGAAGAATTTGGGAAATTAAATTAGGAAGTAATAGTTAATTATTTAGGAATCAATTGTTAAGAATTTTCTAATTAGTGTTGGTTTGATTTCCTAGTTAGTAGTGGCTTAGTATTTTCTTATTGTGGGATTCCTGATGTTAGTCTACATATACCTATGCAAATTAGGTATTTTGGTGGAGTTATTATTATTAACAACTGGGAATTAATAGAAATTTGTGAGTTTTGTATCTTTCCTTTGAGTGATCTATTGGTTCTACATCACTTCCATGTTCCACTTTTCTCCTGTGAAAAAAATTTATGGAATGGGTGGTGAAAATTTTTTGAGAGAAAAAAGAGACTAAGGTGCAAAATAACAAATAGAATGTTGTCATTTCACTGATTAGTTAGAAACTTGATTGATTTGTCCTTGAGCATGCCTTGCACATGATTCCAGTTATCCTCTAAGCCTAATGTTTGTGTTATTGCAG**TGGTTATGCTGATCGCATTCATGAGTTAATAGTAATATCAAGAGAGCTAAACTGCGATGATAAAACTTCTCTGCAAAGAAGTGGAAGTAGGAATTACTTTAGTGAAGCTGATTATGTTGAGTTTTCTGGTGTCAAG**GTACTTGATACTTACAGATTTTATTTCACCATGTTGTTATATATGTACACATTTTCTGTTCTTATGGTAGTAATGCCTCCTAG**GTTGTCACCCCAACTGGTAATGTTTTGGTGGAAGATCTGACTCTTAAGGTTGAATCAGGATCTAATCTGTTAATTACAG**GTATCTTTTCAGCTTAAAAATCAATTATCTGCATTTTTAATTTCTTTCGATTTGTTTTGTTTTCTTGGTCTTTTAATATGGTTCTTTACGCCTATACTTTCAGTTTCTTTGTTATTAACAAGAGTATGTTCTTATTTAACTTTGTTTTGGTGGAAGATCTGACTCTTAAGGTTGAATCAGGATCTAATCTGTTAATTACAGGTATCTTTTCAGCTTAAAAATCAACTATCTGCATTTTTAATTTCTTTCGATTTGTTTTGTTTTCTTGGTCTTTTAATATGGTTCTTTACGCCTATACTTTCAGTTTTTTTGTTATTAACAAGAGTATGTTCTTATTTAACTTGGGCGCCTGTGTCTCAAATTTTGCATAATACCTATAGAATGGCTCTTTCAGGGGACTTTTTATTTATTTATCTGCTACCCCTTGTTGATATGAAGTACTTGATCATGACAAACATTTAATATTTGATTGGATTATCTTTTGGATCCCAGTGGAAAAATGCTGGCCAATATTGGATTAGGTACTACGGTTAGTGAATTTAGGATTCATGGACGTTTCATTTCTAATTGTGTGTCTTCATGGAATGTCTGTTGTGTATCGTTGCATTGTGAGAAGCAATGTGGCTTGTTTGCAGAGCACAAGAAGAAAAGGAGAGGATAGGGGGAGAGAGCAGCATACCTGTGTGGCTCCTTCTTTCTGTCTTCTTTTTCATTTCTTGTTTTTTCTTTTTATGTGTGTATACAACTGTAACTCCTGTGTCCAACTTGTAGAAAACCCAACTCTTATTTGTGCTTCTTTCTAAAAAACATTATAGCGGATCTTCTGATAAAATTCCTATTTAGGCTAAGTTTCTCTGATTATATTTTAAATGTAAATGGCCACCAGCCCATTTTTTTTTTTTTAAATTGTGCTCTACAAAATTTCTAATGAAATATATGTATTTTTTTTTTTTAATTTTAAAGATCCTTGTATTTTTCACTTTTATAATGTCTCCATTTAAAATATCATTACCTCAATTTATTCAATTATGAATAGAAAACAATTATTTATTTTAGATTTGAAGATTATTGTTGTGTATGTTTCGTTAAATATCATGGTTTTCAAACTTGTACCAAAAAACCTGGGAACTGGACAGTGATTGGTCCGGGTTATATTTTAGACCCTTCTCATAAGAAGCTGCTTGTACCCGGAAAAATCGACTGTGGTGAACCGCTGAACCGGGTGACCAGGTTGCAGTTCAACTGGTTTGATTTGCAGTTATGTGGCATGCTAATTAACTGCCATGTCAAGGTTCACTTGAAAAACTAGTGCTTTATTTTAGCTACCTAGTTTCAAACCTTGCTTTTCAAAGTTTCCACGAGCTTGTCCATCCGCTAAGCTACATGACCAGTTTGGTTAAATTCTCTCCACGTTATTTATTTCATACATCTTAAAATCAGTATGGTTGTAGCCTCATTTTTCTTTTAAAGGTCAAAAGTTAACTTTTATGTAAACATAAAACAAGTAATATATCAAAACTGCTTTGATTTTATTATAAATTTTATGGAAATATTTGTTGTCATTTAAAATTTTATCTTTTAATTGTTTCTTTTTATCTTAAAATTTTATTATTTAAAATTTCGGATTATTTGTTAAAGATGTTTATTATATTAAAAATTTGATTGTATTCACTCTAGTAGTTTACTCATAATTGTTATATTGTTGTTTATATATTTTAATTTACTTGTTATTTTTTTAACGGTTTAAACAGCTGGCTGAGGTTTGAAAACCTTGGTAAATGTATATGTTGAACTATGAAATTGATAATTTATTTATTTTCAAAAATTTCATAGTGCTTGTGGACACAGTCCAACATGGACCAATATATTCCTATACTGTTACATTATGACTAACAATTATCATTGTTGAAAACTCGGGTGATTCTGCATTCCATTTGGTGTTAATAATTAAAAGGTTATCCATTCCAAACAGAGCTTTATGGGAAAAAAAAGGATATGATCAGTCATGGTAGAGGAGAATTGTTGGTGGATTTTTATCAGGCAAGACATGGGTAAGGGAAGTTACGTAATGTGATTCTTTTCAACAAATAGGGGGATTGTACCTTACACCCATATCAAGGTTTTCTGGCATGGAATAGCTTCCTAATATGCTGAAAGGAAATAAATGGTGACAGAACGATGAAATAACAAAAAATGGTTAAATGAGCATGCTCTCAGGGTCTATTTTGATGATATGGGGAAAGGGGAAAGTATCATAGATTTCACTTTAGTGTATGGTTTCTCTAAAGTTGCTGAACTCTTTAATTTTAGTTGCTAATAATGCTTTAAAGAAGCTGGCAATTTTGTATTGGCATGGAGTTGGAATTTAACAGATGAACTAGGTGGTAGTTTTAAACATATTAGTTCATAGGCAAATTGTTTAAAAATTTGCATGTCATCTCAGTAGTGTTTATGTTAACAATATAATGTACAGTCTCTATTTGTTTCCATATTGATAGCAGTTTAGCGGGAACTTGATCTTGTATTTGTTGTGATGGTGACCTATGCCTATATAGCTTCATTTAGTTTACTAGGACAAAGATGGAGTTCTCTTTGGTGTGCTTCTCATTGTTGTTCATCTTGGATTTGATCTGCAAATAATGTCAATTACTGGCTGCTTGTATCTGTTAGGTAGTAGACAAGGGGTTATTGTATGGTGTCACAGTGGACGCATAGTTTTGCCTGTGCAAGGGTGCATGTTTGAGTTTTTGACATTTCCCATCCTGAGACATTGCTTTATTGGGATCCTATCTGATTGGCAGCCTTTATCTTTTGTTAAGGCCTTGGTGCTGACCTATTATGTTTTTTAGAAACAAATGTAGCTTGTAAGAAATCTTATATTATTTATCCTTTTTTCTTTTGTGATATCTTGTTTGCATGCATAAGTAGCTTATATTAGTTAGTTTTGATGTTTTCTGATGTTATGCTTTTTTTTGTCATTTAATGAAAATGCTTATTTTAGTATGACTGGGTATCAGAACATGTTCTATTTGTACGTTAGCTTTGTTAATATGCTTCCATCTAAGTTGTATTTGTTAATTTTTGCTGAAG**GTCCAAATGGTAGTGGTAAGAGCTCACTTTTTCGAGTTCTAGGTGGCCTTTGGCCGTTGGTGTCTGGCCATATTGTGAAACCTGGTGTTGGTTCTGATCTTAATAAAGAGATTTTCTATGTGCCGCAAAGGCCATATACAGCTGTAGGCACACTTCGTGATCAGTTAATTTATCCTCTTACTGTGGACCAGGAGGTTGAACCTCTCACACGGAGTGGAATGGTGGAGCTCTTGAAAAAT**GTAAGTATCTTGATTCTTGTCTTATCCCTCTTGTCCTTGGTCTTTTACTGAAAATGCAGACTTTTGCTCGTTTACCTGTAACATTAATACATTATTCTCGTCAAATTTTATAATGCTAGAAATTTGGTTGTTCACTGTGTAG**GTTGATCTTGAGTATCTATTAGATCGTTATCCACCTGAGCAGGAGGTAAATTGGGGTGAGGAATTGTCTCTGGGAGAGCAACAAAGGTTAGGCATGGCCAGACTGTTCTACCACAAGCCTAAATTTGCAATTCTTGATGAGTGCACCAGTGCAGTGACTACTGACATGGAGGAACGATTTTGTGCGAAAGTTCTTGCTATGGGAACTTCATGCATAACAATATCGCACCGTCCAGCTCTAGTTGCGTTTCATGATGTTGTTTTGTCCTTGGATGGAGAAGGGGGCTGGCGAGTTAGTTACAAAAG**GTTTGATTTTGAGTTGGTAAATCTTTATATATATATAATTTTGGAAGGAAGATGGGTATATGCGTTCTAAAATCCTTGAGACTTCAG**GAGGGATTCTGCTGACCTGAAGGAACCTGGGACCAATGACACAAGGGCTTCTAAGACGGAACGCAAAAGTGATGCGATGTTAGTTCAACGTGCATTTGCCACGTCTGATAAG**GTGATATTTATCCTAAGAACTTCTTCTTCTGCCTTTTTTATTTCATTTTTTTAATAGATTGTTGTGTTTCATTGGATGTAATTCACTAACTCTGTAAACTTTATCTAG**GATTCTACATTCTCAAATTCAAAGTCGCAATCATATATTTCAGAGGTGATAGTGGCATGTCCTAGTGCAGATCCTGGTCTTCCTTTACCAATTGTCCCACAGCTCCAAAGGGATCCAAGGGTATTGGCATTGAGAGTGGCTGCCATGTTTAAAATACTG**GTGTGTCTTTTGATCCTTCATTCAGAATGAATTTATGCAAATCAGCTAATGCTATAGATTCTTACCTTGATTATTATGTTAGGCTGTATAATGTGCTATGAAGTTGATCTTTGTGTTGCATTCTATGGTGTCTTTAATCTCCACTGTTGGTCTCCTTTCATCCTGTTTGGCTATTTAATATTTTCTCTGGAACTTTAAATCCTTTGCATCATAATCACGATTGACAAAACTCGGCATTCCATTTATTTCTGAACAAAACTGATCACTGCTGGTAGACAATTGTCACTAGAAACCCTAATTCTTGGGTTTCTACCTCTTAAAATAGAATTAAGAGTTTGAGAGAGAGAGAGAGAGAGAGAGAGAGAGAGAGATTAATTTCTGTAATTGATTCAGAATGATCATCTACAAGTATGAGTCAGCTATTTATTGACTTTCTCTAACAGATTCCATAACTGCTCCAGCTACACAACAACCTAACTCATTTTTCCTCCAAAACATTCTCTTCCCTCCTATTTCTAAACTTCTCTCTAGTATGTTACAATTCCCTCCCCTCAATTACCTTCTTGTTCCCAAGAAGGTTGAAAAGTAGGAAACTGAGCCTGCAGAAAGGAAGAGTCTTCCCAAGTTGCATCTTCTTCAGTGAAGTGTGACCGTTTAATTAGGCCTTAAGTATTGGAAGACCATTCCTGAGGACTGTTCTTGCCTGTAGCAGATTTTCTGGAACCACTTCTACTATCTGATCATCATTCAAGGCAGGAAGATCCAGAATTGGAGTGATATTAGTGCCTATTTTCTTCTTTAGTGGTGAAACATGAAAACTCTGTTGCAGCAATAGAACAATGGAACCAACTTGGAGAGTCATTTGGCAAACAAATAACTGTAGCAAGGGCCTAATTTTCTTCTTCTTACTTCGCCTCATTTCAAACTCATCCTCTTTGAGCAGTAAATGATGACAATATTTGAAGGAATACATCTTCTTCCTTTGCTTTCGCCTAATTTCAAATTCAGTCTCTGAGCATTAAATGATTGCAATTTCTCAATGCTAAGACACAGCTTCCAATCTTTACTTCATCACGCATGAGATAGTCATCTTTAACACGCATGAGATAGTCATCTTCAACATGGACAAATGATGAAAAATATTCCTCAACTTCCATTTTTGTAGCTTGTTTGTTCAACAATCCCAACAACAAATTGTACTCAACTGCACCAACATATCTTACTTAGAATGTTAATTGTGCAAAGCTCTGATGGCCATTGCATCTTCTACAAAATTTCTGTATTTAGCAACACAACACTCTTACATTTTCTAAAATTTAAATACTTTCATGAGGGAGCTTGATATCCATGTCACCATGAATTCCAATTGGATTGTTTTCTTGACTTAATTGCATGAGTTCCTCAACTATATCCTCCTTTCCTTCATCCCCAAACCTAATGCATAATTCTTTCTTAAATTCCTCCCACAACAGATCTACCTTCTCCTTGCTCCAGTTATGAAACCAAGAATTTGCCCTATCAATTAAGAAAAGAGCAGCAATTCCAACCCTTGGATCCCTCGGAACCTTATAGATCTCAAAATACTTCTCACATTTTTTAAGCAAAATCCTAGGTTCACTGCCATCACAAGTAACCAACTCAATTTTTGGTAACAACCCTGATGCCTCCCCATTTCCCATAAAAGAAGATACAGTTACATGCTCTTTTGAATTTCCTTATGCTGCCACCTTAGGAATTGGCAAGATTCCCTTGCTTCCTTGCCCTTCTTGCATCACATTCCCTTCAACTTCCTCATTAACAGTTCCATGCCCTTTTTCAGAAAAAATCCACTCATAATGTTTTGCATCTCATCCAGTAACACTATCTTCATTTCCTCCATCTTTTGCTTAATCTTTTGTTCAATCTTCTTATCAGTAGCTTCCTGCAAGATTCGGAATTTCTCCTCCAATTCATCCATTCTAACAGCTTCTTGACTTATCACTACACTCTTAGACATCTTGCAAACCTTTGGAATCAAAGGCCTCACGTGAAGAACTTCAAGAAAGCAAAGAAAATAAAGAAAGTTAGAACTCTGATTGTTGGAAAGCAGAATAGAAACTCAGGGAAAGGAAATTTTGAGGGATAATCAAGAAAATAGGAAGAACTTCAAGAACAGGGGAAAATTTCAAGAATTTCAAGAAAGAAAAAGAAGAAGAAAGAACAGGGTCTGCAGAAATGAAAATAAGGAATATAGAGGGAAAGAAGGAAAAAATATCTAGAAAAAGAAGGAAGAATTAGAGGGAAAGAAGAGAGTAAGAGAGAAAAAGAAGAGAGAAACCTGAATTTGAATGTGTCTCAGATGAACTAGGAAGCAAGCAGTGCCCACCCCTTGAAAGCTTTGATACCAATTTATCAGTAGAAACCCTAATTCTGGGGTTTCTACCTCTCAGAACAGAATTAAGAGTGAGAGAGAGAGAGAGAGAGAGAGAGAGAGAGAGAAGAGAGGATTAGAGAGAGAGAGAGAGAGAGAGAGAGAGATTAATTTCTTATTTGATTCAGAATGATCGTCTGCAAGTATGAGTCAGCTATTTATTGACTTTTCCTAACGGATTCCATAAATGACTCCAGCTAAACATTCTCTTCCCTCATATTTCTAAACTTCTTTCTCTAGTATGTTACAACAATACATTCGACTTGTTTATGCTTCAAGAAAGGCACTACCTTAGTAACTTCTTGAATAGCGTCTGCAAATTCTACCTTTGTTGAAAACAGAGAGAGGGAGGGAGAGAGAGAGAGAGATCTAANNNNNNNNNNNNNNNNNNNNNNNNNNNNNNNNNNTACTGGGGGGTGCTATTCTTTCTGCTGCCAGTGTTATCAAGGCGAAAGGCGACACTAACACTACAGAGTACCCTATGGCCTTAAGCGAGAGGCGAGAGGCAAGACAAGGGCCTTTTTGAAGCAAGGCGTCATAACCTTAATTGCCTAAATTATATATATATGTGTATGTACTTATAACATAAAAATTCATACTTTTAAATAGTTGATAAGTAAAAAAGCATATTAAAAAGAAAAAAAAAATTATTTACATTATGTCTATATCTAAAATAAGAGAAATTGAAAATAGTGCATACCTGTATTTCCAGCTTAAGTATAAGATACAAGTGAGAGTGTGAGACTGTAAAATTGAAATATATAGCATAAGTAACAACACAAATAATAATAAGAGATGTTAGAAAAAGAAATAGTTCATATCTGCATTTTCAGCTTATGTATATAATAAAAGTGGTGCTATAAAATTATACATAATAAATAAAAAATTAGAAATAAGTCATAAGTCATATAATAGAAAATATAAATTATAGAAGTTCAGACTTTAATAAATAAAAATAAGTTATAATAAACAAAATGCAAGAAGTACATAAAAAACTTATAACTAAAACTACTCGTCATCAAGATTTCCAAAATCATCTTCATTTTCAACAGCGGCCATAACAAATTCTTCCTCCTTTTCGTCATTATCATCAACTTGCGAAGATGATGCACCTCTCATTGAACGAGGCCTTCGAAATGATGGAGTTTCAACTGGTTTTGATCTTGTAGCCGATCTCGATTCAAATTATACATAATAAATAAAAAATTAGAAATAAGTCATAAGTCATATAATAGAAAATATAAATTATAGAAGTTCAGACTTTAATAAATAAAAATAAGTTATAATAAACAAAATGCAAGAAGTACATAAAAAACTTATAACTAAAACTACTCGTCATCAAGATTTCCAAAATCATCTTCATTTTCAACAGCGGCCATAACAAATTCTTCCTCCTTTTCATCATTATCATCAACTTGCGAAGAGGATGCACCTCTCATTGAACGAGGCCTTCGAAATGATGGAGTTTCAACTGGTTTTGATCTTGTAGCCGATCTCGATTCAAAACGAGATTCGGAAACTCTAGCTCCTCTACCAACATCACTCAGGTCAACACGTCATCCATGAAAACAAGAGAATCATCATCATCATCATCCCCATCGCCTTTTTCTAATTCACCAAGAAACCACTCATTACTTTCATAAATATTTGCCAAATTAATCGGTGTTGCGATATCCCCAAAAGTGTGTCACATATACCAAATTGTCTAAGTGTTCTTGAAATAGCCGATTTCTTTTCTTATTATGAAGCTGTCATTTATTTTACAGTAACAAATAAGTTAAACTTAAAACTCAAAAGGCTCAAGATAAAATAACAAATTATATATATATATATATATATGTAGAAAGAGATGATTCTCATTGATTTCAATTAATTTGTACATGGGTATTTATATACAATTGATTCCTATAATTGTGTTATACTAATTAGGAAGAAATCCTAAATAAGAAATCCTAAATAGGAATACAGAATACAAAATATACACAGNNNNNNNNNNNNNNNNNNNNNNNNNNNNNNNNNNNNNNNNNNNNNNNNNNNNNNNNNNNNNNNNNNNNNNNNNNNNNNAGGAATACAGAATATACACAGAAATAATATAATGATTGACTTTCCATAACACTCCCCCTCAAGTTGGAGCATAGATGTTAATCATGCCCAACTTGTTACAAATGTAGTCAATCCTAGCTCCATTCAGAGCTTTTGTGAAAATATCTCCTAACTGCTCTCCAGTTTTGATATGTCCTGTTGAGATGATCTGTTGTTGAATCTTTTCACGAACAAAGTGACAATCAATCTCAATATGTTTGGTCCGCTCATGAAATACTGGATTAGAAGCAATATGGAGAGCAGCTTGATTATCACACCACAATTTCGCAGGTTGGGGGATCTTAAAACCTGTCTCATCTAGTAATTGAAATATCCATATTACCTCACATACTGATTGTGCCATGGCTCTGTATTCGGATTCAGCACTAGATCGAGAAACTGCACTCTGCTTCTTGCTTTTCCAAGACACCAAATTTCCTCCAACAAAAACGCAATATCCAGTAGTTGACCTCCTATCAACCTTAGATCCAGCCCAGTCGGCATCTGAAAAACATTCAACATTCAAATGCCCATGATTACCATATAACAAACCTCTTCCTGGAGCTCCCTTCAGATAGCACAAGATTTGTCCCAAGGCTTCCCAATGAGCAACAGTTGGGGAAGACATAAACTGACTTACCACACTAACGGCATAAGCAATGTCAGGACGAGTGACAGTAAGGTAGTTCAATTTTTCTACCAATCTCCTGTATCTCTCTGGATCTTCAAACAACTCACTATCCCCTGCTAACATTTGTAAATTTGGAGTCATTGGTGCACTACAAGGCTTAGCACCTAATTTTCCTGTCTCTGTCAATAGATCGATGACATATTTTCTTTGAGACAAGAAAATACCCTTCTTACTTCTCATAACTTCAATACCCAAAAAATACTTTAATAATCCCAAGTCTTTGGTCTGAAACTGGGTTTGGAGGAAGGTTTTAAGAGATGAAATACCTGCAGAGTCACTCCCAGTGATGATAATGTCATCCACATAGACTACCAAGAGAATTAGACCAGCCTCAGATTGCTTATAAAATACTGAGTGACCACACTTACTCTTTTGCATACCAAATTCCTGTACTGCTTCACTGAATCTCCCAAACCAGGCCCTAGGACTTTGTTTCAAGCCATAAAGAGACTTCCGAAGCCTACAAACTTTACCCAACTCCCCCTGAGCAACAAACCCAGGTGGTTGCTCCATATACACCTCCTCCTGAAGATCACCATGAAGGAAAGCATTCTTGATATCCAATTGGTGCAGGGGCCAATCATATGTAGCTGCTAAAGAGATAAACAAGCGAACAGAAGTAAGTTTAGCTACAGGAGAAAAAGTGTCAGAGTAATCAACCCCATATGTCTGAGCATATCCTTTTGCTACAAGGCGTGCTTTTAACCTAGCCACAGAACCATCAGGATTTACCTTTACTGTAAATACCCATTTGCAACCAATAGCTTTCTTACCAGTGGGCAAAGGCAACAGTTCCCATGTACCATTAGCATCTAAAGCCTCCATTTNNNNNNNNNNNNNNNNNNNNNNNNNNNNNNNNNNNNNNNNNNNNNNNNNNNNNNNNNNNNNNNNNNNNNNNNNNNNNNNNNNNNNNNNNNNNNNNNNNNNNNNNNNNNNNNNNNNNNNNNNNNNNNNNNNNNNNNNNNNNNNNNNNNNNNNNNNNNNNNNNNNNNNNNNNNNNNNNNNNNNNNNNNNNNNNNNNNNNNNNNNNNNNNNNNNNNNNNNNNNNNNNNNNNNNNNNNNNNNNNNNNNNNNNNNNNNNNNNNNNNNNNNNNNNNNNNNNNNNNNNNNNNNNNNNNNNNNNNNNNNNNNNNNNNNNNNNNNNNNNNNNNNNNNNNNNNNNNNNNNNNNNNNNNNNNNNNNNNNNNNNNNNNNNNNNNNNNNNNNNNNNNNNNNNNNNNNNNNNNNNNNNNNNNNNNNNNNNNNNNNNNNNNNNNNNNNNNNNNNNNNNNNNNNNNNNNNNNNNNNNNNNNNNNNNNNNNNNNNNNNNNNNNNNNNNNNNNNNNNNNNNNNNNNNNNNNNNNNNNNNNNNNNNNNNNNNNNNNNNNNNNNNNNNNNNNNNNNNNNNNNNNNNNNNNNNNNNNNNNNNNNNNNNNNNNNNNNNNNNNNNNNNNNNNNNNNNNNNNNNNNNNNNNNNNNNNNNNNNNNNNNNNNNNNNNNNNNNNNNNNNNNNNNNNNNNNNNNNNNNNNNNNNNNNNNNNNNNNNNNNNNNNNNNNNNNNNNNNNNNNNNNNNNNNNNNNNNNNNNNNNNNNNNNNNNNNNNNNNNNNNNNNNNNNNNNNNNNNNNNNNNNNNNNNNNNNNNNNNNNNNNNNNNNNNNNNNNNNNNNNNNNNNNNNNNNNNNNNNNNNNNNNNNNNNNNNNNNNNNNNNNNNNNNNNNNNNNNNNNNNNNNNNNNNNNNNNNNNNNNNNNNNNNNNNNNNNNNNNNNNNNNNNNNNNNNNNNNNNNNNNNNNNNNNNNNNNNNNNNNNNNNNNNNNNNNNNNNNNNNNNNNNNNNNNNNNNNNNNNNNNNNNNNNNNNNNNNNNNNNNNNNNNNNNNNNNNNNNNNNNNNNNNNNNNNNNNNNNNNNNNNNNNNNNNNNNNNNNNNNNNNNNNNNNNNNNNNNNNNNNNNNNNNNNNNNNNNNNNNNNNNNNNNNNNNNNNNNNNNNNNNNNNNNNNNNNNNNNNNNNNNNNNNNNNNNNNNNNNNNNNNNNNNNNNNNNNNNNNNNNNNNNNNNNNNNNNNNNNNNNNNNNNNNNNNNNNNNNNNNNNNNNNNNNNNNNNNNNNNNNNNNNNNNNNNNNNNNNNNNNNNNNNNNNNNNNNNNNNNNNNNNNNNNNNNNNNNNNNNNNNNNNNNNNNNNNNNNNNNNNNNNNNNNNNNNNNNNNNNNNNNNNNNNNNNNNNNNNNNNNNNNNNNNNNNNNNNNNNNNNNNNNNNNNNNNNNNNNNNNNNNNNNNNNNNNNNNNNNNNNNNNNNNNNNNNNNNNNNNNNNNNNNNNNNNNNNNNNNNNNNNNNNNNNNNNNNNNNNNNNNNNNNNNNNNNNNNNNNNNNNNNNNNNNNNNNNNNNNNNNNNNNNNNNNNNNNNNNNNNNNNNNNNNNNNNNNNNNNNNNNNNNNNNNNNNNNNNNNNNNNNNNNNNNNNNNNNNNNNNNNNNNNNNNNNNNNNNNNNNNNNNNNNNNNNNNNNNNNNNNNNNNNNNNNNNNNNNNNNNNNNNNNNNNNNNNNNNNNNNNNNNNNNNNNNNNNNNNNNNNNNNNNNNNNNNNNNNNNNNNNNNNNNNNNNNNNNNNNNNNNNNNNNNNNNNNNNNNNNNNNNNNNNNNNNNNNNNNNNNNNNNNNNNNNNNNNNNNNNNNNNNNNNNNNNNNNNNNNNNNNNNNNNNNNNNNNNNNNNNNNNNNNNNNNNNNNNNNNNNNNNNNNNNNNNNNNNNNAATCTAGATATAGCTATCGATGAGAAACTCATCGGTGACCATAAAAAAAAAAGATCTGAAAGAGAAAAGATGAAAACGAGAGGAGAGGAGGAGAAAATGAATAATAGATTTGACTACATAGTCATATAATGGGAGCTTGCCATTAACGTGAGAAGACTATTTTTTTTTTAAATATTATATCTAAACAGAATACTATTTAGATTAAGGAGCTTGCCTTCTTGTTAATTACATCTTATTTAATATTATAATATTTTCAAATATTAAAAATAATAAAAAAAAAGTTTAGCAAGAGTTGAAGTTATAATCAAACACAGACATTTAAACACATTCCAATAGCATACAATCCATTTATGTGATTTGCTAATCCATATGATTATATAATTGTAGACATAATGCTGGTAAAAATAAGTGAAATTAATAATCGATATAATCCATCAAAATTATTTATTGCTTTGAAAAATTCTTCCGTAGATGAGTATTCTTTGATTTAACTAATCCAATTTTCAATAAATTTGCAGTTCCATTTCAAGAAGTCTGTTCAACTGTATTTTGTTGTAAGATTTAGCTATGAATGCAAATGGCCTGACAAGTATGATATCTTAATTATCATTTTTAAAAGTACTTCCATGTTTGCAGTAATTATTTTGATCATAAATCTATCGATAGTCAAGGAAATAGGAAGGGAAATCAAAGATGAATTCTTTATTGAATGTAAGCTTGAAAATGTTTTAATTTATAGGTGAATGATGAAACTAGTTTCATGATATTAATCAGTGGGTCATTATTTTCCCTTTCCTGTTTATAGGAAAATGTAGTTTTAATTAGTCTAAAAATTGTTTATAAAAATATCTATTAATTTTTATGGTTTATTAGTTGATTAGCATGAATTATTTTCAAAAAAATTATATATAAAATAAAAATCAAATAATGAGTACAGACAACGAGCACATAGAATGTGCAACGCATATTAGATAAAAACTCTACTCAAACTCAACTCAACTAAGCCTTTATCCCAAAAATTTGGAGTCGGCTATATGGATTCACTTTCTCCACTTTGAACGATTTTGGGTTAAATCCTCAGAAATGTGTAATGCTTCTAGGTCATGTTGTACTACTCTCCTCCAAGTCAATTTAGGTCTACCTCTTTTTTTCTTTCTATCTTCTAACCTAATGTGTTCTACTTGTTTAACTGGAGCCTCCGTATGTCTACGCTTCACATGACCAAACAACCTCAATCTCCCTTCTCTCAACTTATCTTCAATTGGCACCACTCCTACCTTTTCTCTAATACTCTCATTACGGATTTTATCTAGTCTAGTATAGCCACTCATTCACCTTAACATTCTCATCTCTGCAACTCTTATCTTAGATGCATACGACTCTTTCAGTGCTCAACACTCACTACCATATAACATAGTAGGTCGTATGGCTGTACGGTAAAATTTTCCTTTCAATTTATTGGGAATCTTACGATCACATAAAACTCCCGTAGCACGTCTCCACTTCAACCATCCGGCTTTAATCATATGACTAACATCCTCCTCACATCCCCCATCTACTTGAAGGACTGAGGCTAGATATTTGAAGTGATTACTTTGGGGCAGTATCACTCCATTCAAACTAACTCCTTCCCTATCACCAGTTTGGCCTTCACTGAACTTGCAATGCATGCATTCTGTTTTCGTTCTACTTAACTTAAAACCCTTTGACTCTAGAGTACTTCTCCAAAGTTCTAGCTTTCTATTGACTCCTTCTCATGTCTCATCTATCAGAACAATATCATCCGCAAACGTCATGCTCCAAGGAATACTATCTTGTATATGTTTCGTCAGTTCATCTAAAACTAATGTAAAAAGGTAAGGGCTTATGGCTGATCCTTAGTGTAATCCAATTGAGATCGGAAAATCTCGTGTCTCCTCTCACTGTGCGCACAATAGTAGTTGCTCCTTCATACATATCTTTCAATACTTGTATGTACCTAATAGATACCCTCTTTTATTCTAACACATTCCATAAGACCTCTATTGGAACACTATGATAAGCCTTCTCCAAACCAATAAAAACCATGTGTAGATCTTTCTTCACATCTCTATATTTCTTCATCAAGCTTATAATGAGAAAGATCGCTTCCATAGTTGAACGACCGGGCATGAAACCAAATTGATTGAGAGAGATAGAAGTATCATGACGTAGTGGATGCTCCACAACTCTCTCCCACAACTTCATAGTATGGCTCATGAGTTTAATTCCCCTATAGTTTGAGCAACTCCGTATGTCTCCCTTATATTTAAAAATAGGTACTAAAATACTCTTCCTCCATTCATCAGGCATTTTTTTTGGGTTTAGAATCTTATTAAATAATTTAGTTAACCATGCCACTCCCATATCTCCCAAATACTTCCACACTTCAATTAGTATTTCATCGGGTCCACAGGCTTTACCCACTTTCATTCTCTTAAGTGCTTCCTTTACTTCTAAAGATCTAATCCTTCTAGTATAATTTACATTCTTTTCTATTGTTCTATAATCTATATTCACGCTATTTCCATTTTGACTATTATTAAAGAGATCATTAAAATAATTTCTCCATCTTTCTTTAATGTCCTCATCTTTCACCAACACTTTTCCTTCTTTATCCTTAATGCACCTAACTTGATTGAGATCTTGACATTTCCTTTCTCTACTCCTTGCTAATCTATAAATATCTTTCTCCCCTTCTTTAGTTCCAAGTTTCTCATATAACTTTTCAAAGGCCTGTGCTCTTGCTTGGCTAACTGCCTTTTTTGCCTCTTTCTTTGCTATCTTGTACTGTTCATATGCCTCATTATTATCACATTTAGGTAATTTCTTATACCATTCCCTTTTTCTCTTCACTGCCTTTTGTACTTCCTCATTCCACCACCATCTCTCTTTTGAGGGTGGTCCATGTCCTTTAGACTCTCCAAGTACTTTTCTAGCTACTTCTCTAATCTTTGATGCCATCTGTATCCACATATCATTGGCCTCCATATCTAGCTTCCATACTTCGGACTCGAGAAGCTCATTTTTGAACTTCACTTGCTTTACTCCTTTGAACTCCCACCACTTTGTTCGAGCTACACTATTTCTTCTGACCTTACTTGAATTGTTCCTAAACTTGACATCCAAGACCACCAACCTATGTTGACTTGTTAAAGCCTCTCCTGGAATGACCTTGCAATCCTTGCATAGAGCTCTATTTGTCTTCCTGGTTAAGAGGAAGTCGATTTGGCTTCTATGTTGCCCACTTTTGAAAGTCACTAAATGTGACTCTCTTTTTATAAAGTAGGTATTTGCTAGTATTAGGTCGTATGCCATAGCAAAATCCAGGATGCTTTTTCCCTCCTCATTTCGACTGCCAAAACCAAAACCTCCATGAACATTCTCATAACCTTGCCTATCACTTCCTACATGTCCATTCAAATCTCCACCAATGAAAACATTCTCTTCATTCGGTATGCTTTGCATTAAATCATCCATATCTTCCCAAAACCTTTGTTTACTCTCACTGTCTAGTCCTATTTGTGGGGCATAAGCACTAACTATATTTATTGTTTCTCCTTCTAGTACTAGCTTTACTAGTATAATTCTATCTCCTACTCTTTTCACAGCTACTACTGCGTCTTTCAATGTTCTGTCTATGATTATACCCACTCCGTTCTTGTTTCTCTCCTTTCCGGTAAACCACAATTTGTACCCTGAATTACCCACTTCCTTACTTTTCTCTCCTACCCATTTAGTCTCCTGAATGCAAGCAATATTCACCCTTCTCCTTTCCAAGGTATCCACAAGTTCCATTAACTGATCCAACATTCCAAGTACCAACCCTGATCCACCTCCTATCCTGCTCCTTCCTAATTGGTCTCCTTCTATGATATCTTCTATTGTTTTCTATGTCTATCTTATGTTCTGTTCCACTATTTATTCTACTATCTGTCCTATGGACTAACTTCTTTATCCACACCCGTCCATGATGTGGGAACCCTTGCTCACTTAACACCACACCCGGGCGCCGGCATGGCGCGTCGCTTTCGGTGAACGCCCTACACCCTTGCATATTTCTCACTACACCCGGGCTCCGATGTAGCGCGTCGTTAGTAGAGGACGCCCCAACGTTTATATCATTTGAAATAAATTAATAATACCTATATAAAATAAATAATTAATTAACAACTAATGTCTAATTAACAAGGCAAAAAGTAAAACTAATCAAATTAACAAGAATAAAAAATAAAAATTAAACTAATTACCAACAAATCAACAATACCCATGTTAATTAATCAACTAATTAAACAAGAATACAAAGTAAAATAATCAATTTAGTAATACCCATGAAATTTAAATAATTAATTAACAAGAATAAAAAATAAAATAATTAAATTAACAACAAATAAATAAATAATTAATAATAACTAATTAATAATGAGGAGATGGGGAAAAAAAAAGGGACTGCTGAAAGAAGAAGGAGAGGTGAGGTATGCAGTAGGAAAGGGACTGCTGAAAGAAGAAGAAGAAGAGGAGAGGGGAAAAAAAAATAAAAACAGTGTTTTACGCAATAGGAAAGGAGATAAGAAGAAATCAAAGAAGAAAAAGACGAGAGAGAGAGAGAGAGATCGGAGATCAGAAAGAAGAAGAAGAAGAAGAAGATAAGAGAGAGAGAGAGAGAGAGGGTACCTATCTTTGTTGCGAGGAAGAGGACCCTGCGATCTGTCCCTGTCATCGTGAGGTCGAGGAAGAGAGGTGGGGCTCTGCTGTCTTAGTGAGGAAGTGGTGCGGTGCAGCTCTTTTGTTGGCATCAAGTGGCTTTACTGGTTTTTTCAGTTAAAAAAAAAAAAAAACTTACCGTTGCTTTGATGAGGTGTCGCCTCTCGCCTCACTGGCCTGAGGCGTTGCCTCTCCAAAGCCCAGCCAGGCGTTCCAGTCGAGGAGACAGAGGGGCGCCTCGCCTGGCCTGGGCGCTTTCCTCGCTTTTGATAACACTGTTTTAGGCTTCCTAGTTTGAAGCAGAGAGAGAGAGAGAGAGAGGTGTTTTTTTCTTTTTTTTTATTAAATCATCCATATCTTCCCAAAACCTTTGTTTACTCTCACTGTCTAGTCCTATTTGTGGGGCATAAGCACTAACTATATTTATTGTTTCTCCTTCTAGTACTAGCTTTACTAGTATAATTCTATCTCCTACTCTTTTCACAGCTATTACTGTGTCTTTCAATGTCCTGTCTATGATTATGCCCACTCCGTTCTTGTTTCTCTCCTTTCCTGTAAACCACATTTTGTATCCTGAATTACCCACTTCCTTACTTTTCTCTCCTACCCATTTAGTCTCCTGAATGCAAGCAATATTCACCCTTCTCCTTTCCAAGGTATCCACAAGCTCCATTAATTTTCCACATTCNNNNNNNNNNNNNNNNNNNNNNNNNNNNNNNNNNCTCCTATCCTGCTCCTTCCTAATTGGTCTCCTTCTATAATATCTTCTATTATTTTCTATGTCTATCTTGTGTTCTGTTCCACTATTTGTTCTATTATCTGTCCTATGGACTAACTTGCTCCTTCCTAATTGGTCTCCTTCTATGATATCTTCTATTGTTTTCTATGTCTATCTTGTGTTCTGTTCCACTATTTGTTCTACTATCTGTCCTATGGACTAACTTCTTTACCCACACTTCCAAGGTATCCACAAGTTCCATTAACTGATCCAACATTCCAAGTACCAACCCTGATCCACCTCCTATCCTGCTCCTTCCTAATTGGTCTCCTTCTATGATATCTTCTATTGTTTTCTATGTCTATCTTATGTTCTGTTCCACTATTTATTCTACTATCTGTCCTATGGACTAACTTCTTTATCCACACCCGTCCATGATGTGGGAACCCTTGCTCACTTAACACCACACCCGGGCGCTGGGATGGTACGTCGCTTTCGGTGAATGCCCTACACCCTTGCATATTTCTTGCTACACCCGGGCTCCGATGTAGCGCGTCGTTAGTAGAGGACGCCCCAACGTTTATATTATTTGAATCCATATCATAGGGTGTGACGAAATTTTTACGCTGATTGTCACCTACCGCAACCCTCCTCCTTTATCCGGGCTTGGGACCGGCTAAGCGCAAACTATTTAGGCGGAGTTAATTACTTATCAAGAATAAAAAGAAAATAATCAAATTAACAATAAATTAATAATACCCATATAAAATAAACAACTAATATCTAATTAACAAGAGAAAAGCAAAATTAATCAAACTAAAACTAATTAACAAGAATAACAAGTAAAAGGTAAACTAATTAACAAAAAATTAACAATAACCATATAAATTAAACAATTAATTAACAATAATAAAAAAATAAAAATATTAAATTAACAATAAATTAATAATACCTATATAAAATAAATAATTAATTAACAACTAATGTCTAATTAACAAGGCAAAAAGTAAAACTAATCAAATTAACAAGAATAAAAAATAANNNNNNNNNNNNNNNNNNNNNNNNNNNNNNNNNNNNNNNNNNNNNNNNNNNNNNNNNNNNNNNNNNNNNNNNNNNNNNNNNNNNNNNNNNNNNNNNNNNNNNNNNNNNNNNNNNNNNNNNNNNNNNNNNNNNNNNNNNNNNNNNNNNNNNNNNNNNNNNNNNNNNNNNNNNNNNNNNNNNNNNNNNNNNNNNNNNNNNNNNNNNNNNNNNNNNNNNNNNNNNNNNNNNNNNNNNNNNNNNNNNNNNNNNNNNNNNNNNNNNNNNNNNNNNNNNNNNNNNNNNNNNNNNNNNNNNNNNNNNNNNNNNNNNNNNNNNNNNNNNNNNNNNNNNNNNNNNNNNNNNNNNNNNNNNNNNNNNNNNNNNNNNNNNNNNNNNNNNNNNNNNNNNNNNNNNNNNNNNNNNNNNNNNNNNNNNNNNNNNNNNNNNNNNNNNNNNNNNNNNNNNNNNNNNNNNNNNNNNNNNNNNNNNNNNNNNNNNNNNNNNNNNNNNNNNNNNNNNNNNNNNNNNNNNNNNNNNNNNNNNNNNNNNNNNNNNNNNNNNNNNNNNNNNNNNNNNNNNNNNNNNNNNNNNNNNNNNNNNNNNNNNNNNNNNNNNNNNNNNNNNNNNNNNNNNNNNNNNNNNNNNNNNNNNNNNNNNNNNNNNNNNNNNNNNNNNNNNNNNNNNNNNNNNNNNNNNNGGGGGGGGGTGTTGGGGGCGAGAAGATGGAAGGAAAGTGTGCTGTATTATACAGCTATTGTTTTATTTTCATGACTAGTGTACATGTGTTGGATATGCACACTAACATGGAATGAAAGGATGATGTATAGTGGGTTGAAATAAAAGTATCAATGGTCTTTTGTTGGAAAGCTCAGGAGCAAATTATTATTAGGCTTCTCCTTCAATTTGCAAATTGCATTTAGAAGAATGGCTACACAGTGTTGTAGAAATTCAACTCAGATCATGAAGTTAATTTGTGTCATCCATTGATGTAGTCAAGTCTTCAAGGAATGTGGAAATTGATATTGAGACCATACAGAAGATTGAAGTGTTAGGATACTTGGCACAAATCTCAGTATATTGTCCTTGTTTCTAGAAGGAATTAGGGTACGCTCAGAAATATGAGTCTTCCTTTTGCTAATTCTTTCAAGCACTTTTGCATTCATTATCCATTCTTAGGTCTTTAGCTAAAAATGTTACATGACTGATAAGATTTTGTCAGCTCTAGATTGTGTGGCTAATTAATGGGTTTTGATAAATTTGTAATGCTGTATTAGTGAATTTACACGTTCTGTTGGTATTATCACTTTGGACTCATCTTATACACAAGACGTGCCAACATATCGTGATTGCTGTATTTATTGTTTTGCCAGCCTGCTATACAAAATCTGGGGAGTTTGTTGGCTTGAAAATTTCCTGTAACTGCTTTATGGGTGAAATTCGAGGTCTTGTTAGAGTCTAAAGTGGGCAGGAATTGGAAAGTTCAGGTTTAGAAGGGTGTAGGCTTGGGAAGTAAAATTATTACAGGAAGCTTGGTTTTAGGGATGTTGTAGGACTGAATTAGGCAAGCAAGCAATAGAATAGTTGGGCGTCACACCTAAACATCCTTAGGCATCACACTCGTGTAGAATTGTATCAAAAACTCATGAAGCAAAACTGCAGGAATTCTTATTGATAAAAACACTATCAAACAACATTACAAATGTAGGCATTTATAGACTCAAAAAGTAAATTCTTATTCTTTTTAAGGATTTGGATTCCAAAGTAAAACTTAACCATAATCAATCTAAAAAGTGATCCCAAAATGTAATCCTTAATCAACAAGGACTCTAAAATAAAGACTCCTATAGAAATCTAGAACTTCCCATAGAATGTAAAATTACAAAAACAATCATCTTCTAGAGAGTAAAAAATCCTGCAAATTTCCTAGGCAAGAGCATCAAAACAAGACTGGTGGAACTCTAATTTAGTTCCCCACAATGATCCTTGTCATCAATGCGCCAAATAATTGGAGCTCAAAACAAGACTAATGGAGCTCTAAGTAAGTTCCCAACAATGTTTTTTGTCATCAAAGTGCCAAATAAGTGCTTGGGCATTAGAATTAGCTTTTTCATGTCTTTTTTACGTATGTTTACCATTACATTCCTTCAATATTTGTGGTCTTATGCTTGGTTGCTTTGGTATTTTCCCACAACCTACTGAATGGCACACTATGGCTTATACAATGCATCTGTCTCCGAAATTTGTCAACTCTTGGCTGGCTGTTTTATGCTTTCTATTTATATACTTTCTTTTTCAATGCTTTCATTTCAG**GTACCAACTTTACTTGATAAACAAGGGGCACAATTACTTGCAGTTGCTGTTCTTGTTGTCTCAAGAACATGGGTCTCCGATCGGATTGCCTCATTGAATG**GTACTAGTATCCATACTTGACTGTATGCCTTCAATAATCCAAGGAATAGTTTTCTTCTTATCGAGTGTATTAATTTTCACAG**GCACCACTGTAAAGTTTGTCTTGGAGCAGGATAAAACATCGTTTATTCGATTAATTGGTGTTAGTATTCTTCAAAGTGCTGCATCTTCTTTCATTGCACCTTCATTGAG**GTAATTCTGGTGTCAAATTGGTTTGTATTGAAGCTCTAACCATAGTCACTTTTAAAATCATTACTCCCATAAGTGGTGATTCTGTGTATTATTTACAACTGGACATGTGATTAATGCAGTTGTGAAGAGGCTGTTTACTTGTGAAAAATATTTCTATTTTTCATTTTTCACGTTGGAATTGATGAGATTTTTCCTTAGTCCATGGGATATGTCTCATTGAAGAATCCCATGAATATGAGAAAATCATGAGATTGATTAGCAAAATCTTAGCAGTGATTATATTGACAGCAGTCCATATTATGTAAGACATGCAACACAAGTGAATAAAATGTGTCTGTGGATGTAGGCTTGCCGTAGTTCTCTTGCTACTTTTCTTCTATGTTTTCTATCCTTCTAAATTTCAACATTTCATTTTCTATGAAGACTCCAGTTTTCATTTTCCACAGAAAACATGTGAAAACATGAAAAATGCATACATTTGTCAATAATAGAAGATAATTTTTTTAATTCAAAAATAAAAAAAATTATTCATATCTTTTATAATTAAAGAAAGTCATTATAATATATGTTTAGAAACTTATCTTCTAAATTTTTTTTAATGTGTTATTAATTTATTTTTGATGATATGATTAAGCTTTTTTTATCATTGTAAATACATATTTTCGTTAGTTATGGAAAATTTATAGTATAATTTTGGTATAGAAAAGTCATTGTTTTTCATTTAGAAAACAAGATTTTTGTTGCTGTTTTCTAAATCTTAGTTTAATTTTGGAAAACTAAAAAACCAGTTTTCTTTGAAAAAATTTCTAGAGAACTACCTCTATTTTCTACAAGTGAACAGGTCTCAAGTCTTTTGAAATGCTCTCTGTTCGATATGCTTGAAAGGACACCATTTAATTCAACAAATAGTTTGTTATGGGTTTGGTAACTAACGGCCTTCAAGATTACTTTATTATAGTGACCAGTGCCACTTCTTAATATTCTGACGTGATTCTAGGTGTAACTAGCTGAGCAATGACCCTATTTTTCTCGTTTTCCTTTCTATTCATGGCTTAGGGAGTGGGGTTGGTTTTCTTATGGGAAATTAAGCTTAATTATCACCTTCTGGATTGAGTATGACCTCCTTAAGAAATGGGTTGGTGGGTTCAAAGCCATTTGCCCCATTTATTCCTTTATGACAAACTTTTTAGCCCTAAATTTGGCGCATGATTCTTTTGCAAATAAACTTTCTTCTCATCACTCATCTTGAAAAAGGACATGTAATCTACACTGGTCTTGCTTTGTAAATTCTTCCTTCCTTCACAG**GCACTTGACAGCCAGGTTGGCCCTTGGATGGAGGATTCATTTGACTCAGCATCTACTAAGTAACTATTTGAGAAACAATGCATTTTACAAG**GTAATTAGGTTTGGAGTATTTAACATCTATTTTGTAGTTTGATTCACTGTTGAAATGCATTAACAGTTTCTGTTAGTAGCTGGCATTTACTAATTAAATGGAAAACTTCTTGGGTATGCATTTTTTAAAGTTTTGTGCTTTTTTATTGTGCAAAAAGGTTTTTAATTAGTTGAAGCTGTATATTTATATATTTGTAGATAGTATTTCACATAATTCTTGTGTCAGCCATCAAAAGCCATAGCTTCATGTCCAATATTTGTTCTTGCAG**GTCTTTCATATGTCAAGCAAAAATATTGATGCAGATCAAAGAATAACTGATGATCTTGAAAAGCTGACCAGAGACTTGTCCGGACTGGTAACTGGAATGGTAAAGCCATTAGTAGACATCTTGTG**GTGAGTACTTGGCTCAACTTTATTTAATTATTATGGTATAATACTAATTTCTTATTTGTAGAATGTAGAAAACTGATTTACTTAAGTGCTTTGTTCAATTCTACCACAAATTTTGAAATTCATTTGAAATGAATTCCACTATTTGGTACGATACAACTTAAAATGCAGAATTTAATTGAATTCCATATAATTAATGTTTGATATAATTCCAGAATTGAAATTCATTTGTCTTTATTTCCTAAACTATCCTCAAGAGTAAAATTAAAAAAAATATAATTTTCTGCAGGATTCAAAAAAAATCTTTTACCTAATAAATTTATAACAATTAACAAACATATAAACCAATTAATAATATAGTAATAAATATAAAATAAAATAACATAAGAGATTTGTATAACATAAATAGTGTCATTGCTAAGTGCATGATAAACTTAAATATTAAAAAGTAGGACAAATGTAAAATATTTTATATAGTAATAAATGTAAAAAGGAGACTTTTTTAGGAAGAATTGTGTAAGGTGACAAATTGCTTTAAGGAAGAATTTAAGGATGATATTAATGGTACTTGGGGCTATTTTTCTTTGAAGATTCGTACCTTTTTTAATTTTTTAATTAATTAATGGTGCTTATTGGGTGTAGTAAATTCTATTGATAACATTAGTGATCAAAGTTTTAAAGTGAATAATTTCAACATGGAGAGTGTATAAAAAGATGAATAATTGAGAGGAAATAAAAATAAGAGCATTTTAGTGCAAAAAATTTTATGTAAGTTCTGTGGAATTCATTTCAAATTCCTTCAAGTTTGATAGGAATTGGCTTTATTTATAACCAATTTTTTGAATTTAATTTTTTTTTTCTTTTCAAATCAAACAAACAAATCATGAAATTCAATTGAATTTCACAAAATTGTGTTTTGGAATTGAACCAAGCAAGTTCTTAACATTCTGATAAGTACTCTCCCTATTTCTATGTGCCAATAATGATTCCACTGCTCAATTTGTAAG**GTTTACCTGGAGAATGAAGCTGTTAACAGGTCAGAGGGGAGTTGCCATATTGTATACTTATATGCTGCTTGGTCTGGGTTTTTTGAGAACTGTAACTCCTGACTTTGGTGATCTTGCAAGTCGAGAGCAACAACTTGAAGGAACTTTTAG**GTAATTTAATCTCATATGTGCCTTGTATATTTAGTGTTGTTATATATTTTACATGTTGGAGAAAGATTTAAGTTTGAATATATATATGACGATTGTAACAACCTTTGGTGCTGCCTTTTTCTGTAAAAATTGTTCTCACTCTGTCATGCATATATTAG**GTTCATGCACGAAAGACTATGTACACATGCCGAGTCTGTTGCCTTCTTTGGAGGTGGTGCAAGAGAAAAAGCT**GTAAGCTACTTGTGTTTTATCATTTTACTTAATTTTACACTAGAACTGGTCATATGCCTGTGCAATTCTTGGATTTATTATTATGATATAATTTTATATATAATATGAAAATATTATTTATACATTGGTGCATGTAATAGTGTGTTTTCTTAAATAAATAACTTTTGTAGAAATAGAGTTTTATTATGAATATATTAAGGCATGTAATAATTATATCTTAAATAGTGACCCATCTTGTAATGTCAACTGTATGGAAAATTAGTCTTACATGGAAAAATACTAATTAATTAAGAAGCCACTTGTCAAGAAAAAAGACATGACACATGGCAAACTGTGGTGTAATATGAACTAAGCTCAACTTTATATATATATATATATATATATACCTCGCTCCTTATACAGGAGCAACTCTGGCTGAATATTTTATGTATTGTGAATGGCATACTTTAATCATATATGATCTCTCCAAACAAGCGCAGACTTATTTTATATATATATATATATATATATATGTTGGAGTGTAACGTTGCTTAATGTATTGGGCATTGATCATTAGACATAATCACTCATTATAGCTTTGAATACAGGATTTTACACATGGGAATTTTTAAAAATGTATGGAATATGTTACAATAATATCGGCTTTTATTCCAGATCAATACTCATATGGGAGTAACTTTTAGTATAATTCAAATTATTATTTATAAGTATTTACCTTTTGCCTATATGTTGTTTCAACCAGTTGAAATTTTAGTAGATGAAAATCATCGTTTCTTACCATGTTCCCCATCAGCATACATGAAAATTGAGAGGCATATAAGTATAAGCAAACAAACTTGGTGATGGGGAGTCTAGCTTAGTGTGTAACTTGCAAGTTTGATATTCATGCTCCTAAGTCCATAATTTTCAACATGGTATCAGGGTCGATCATTATCTCTATATTTGGTCACCAGTCATGCAAACTTCTCCCAAGATGTTCATGCCTGGGCACAAGGGGTTGTATTGTGTTACATTGACCAGGTATAAGACAAGAGTGTTAAATAAAAAGATTTGAGCTTTTGGGGTTGAGTTAGATCTGGTCCACTTTATTGGTATTGGAGTAGTGAGGCCTAAAGGCAGTAGTGATGTAGAACAGCTAAGAACAAAGAACAAAATAAGGACCAATCCTTGAAAAAGAGAACTAATTCTCAATAATTTTATTTAATCTTAATAATAATCATAATCCAAAGCTACTGAATAATAGAAAAATAGCTCTATTTATAGAGCTTACAACCCTAATCAAATTAGGAATAGAAATCCTAATTCAATTCTAATTAGGAATACCAAATAAGATAAATTAGAAAATAATAGACCTAATAATAATAAAATTTCTAAACAATAAAAACTCTTTAAATTTCTTAATTCTATAATAATTAAAATTCCTAAATAAAATTCTAAACTTCCTATTTTGCAATAAGTAGATTTAGGGCCTAAAAACCTGGTGGGCTAAGCCCCAGTGAGCCAGACCTATGAGCTAAGTCCCAATGAGCAAAACACAGGTGGATCTATGAGCTAACCCAATGAGTTAGAACTTGAGTGCAGAGCCAAGAGAAACTAGACCCAAGTTAGATGGGCCAGCACATGCAATGGTAAGTGACAACTAGGCCTGTGAGCCTTGAACTGAATCTAGCTACACTTGAGGGGAGAGTATTGGAATATCACTTGTCAGAACAAGTTCCACATTAGCAGTGTACATGAAAATTGAAAGGCATACAAGTATTAGCAAACCAAACCAAAATGGCTTAGCCATTTTGGTGATGGGGATCCTAGTTTCAAGCATAGTTTAGCATATAGCTTACCAGTTTGATATTCACACTCTTAGGTCCATAATTCTCAACAATTTCTAAAACCTATTAAGCAGCTTGAAAATAGTGACCTGCAGATATTTAAACTGGAGAACTATTTAACGAGGTAGATTTCAGTTGACATGTTTGTGATGTTGCTATGATGCCAGTGATTTTTGGCATTATGACATGTATTAAAGTTGATTTTTGGTTAGAGAAATTTTTTGTAGCAGCTTTACTACCAATCACAGTCCAGTTTGTGTCAACCTTTTGTTTTGCAATATGCTTTAGGAAATATTTTGACTCCCTTTCTCAACTCTAATATTATGCCCTTTTTTAAGAATATATTTTATCATCCTTAATTTATAAGTTGTGTTGAGCAG**ATGATTGAGTCAAGATTCAGTGAACTGCTGGACCATTCTTTGTTACTTCTAAAGAAGAAATGGCTATATGGTATCCTAGATGATTTTGTCACAAAGCAACTCCCACATAATGTAACTTGGGGTTTGAGCTTGTTGTATGCAATGGAACACAAGGGAGACCGAGCTCAGGTTTCAACTCAAG**GTGAGATTAGATTGTAATTAACTTGAATTGCAACTTTCTTTTTATCACTCTCCCACATATTTGTTACATATTTTTCAG**GTGAGCTGGCACATGCATTGCGGTTCTTAGCCTCTGTAGTCTCTCAGAGCTTTTTAGCATTTGGAGACATTCTTGAACTGCATAAGAAGTTCCTTGAGCTCTCTGGCAGTATAAATAGAATTTTTGAGCTGGAAGAGCTTCTAGATACTGCTCAGTCTG**GTAAATTCTTCATTCTATTGATTGTATTCTTTATATTAAAAAAAAAGGTTCCTTATTAATCCAATTTCGTGGGCCTTAGAGGGGGGAAAAAAAGGAGATTGGGACTTTCTTTTAATGTCATTCCAGAAGCAGGAAGAATTAACAGCTCTGCATATCATTGACAAATTGCAGTAAAAAAGACTTGAATTTGAGAAATACTGACAAGGAAAACATAAGTGCAGGATCAAATGAAACTAAATGGCTGTGTATTGCCAAAACAGAACAATTTACAGAAATTATCTAGCCCTAATACAAGTTAAAAGTAAAGGTGTGGAGATATTTATCGAGTAGGATCAGATAAAACACTTTGATTTGGTATATGTCAAGATGACATACAATTGTAACTAACATTAGTTAACTATCCTACATGTATCCAAGTTAAAATTGTCGTTATGTAGGAGTAGGTTGTTTTGTTTGTATAAGTATGCATTCAATATTCTGATAAAGGAGCTGATATGTTCTGAAGACAAAATCTGGTAATTGCTTGGTTTTGTGCACTTGAGAAGGTTTTGCCTTCATATTTCTTTGGAAAGGTCTGACTACTAAAAATGCTTTTATGCAACTCTGGTCTTTTACATTTTTGCTAAGCTTGCTTTGTGCACTCTAGTGCTTAAAAAAAAATCCAATAATAATCATCCTTTTATCTGGACTTCTCAGTGAGATGAAAAGGATGGATATTGGTTTGGAATTTTGAAGGCCTTTGTCCATGAGATGCATGGCTGCATATTTATCAAGAAATTTTTTGGTATCACTGAATGAAGCAACCATGTGTAGAAGGACCATTAATCTCTGAACATAATGAAGGAAATGATCCATATAGCTAACCCGAACAAGCCTAGGATTGAAGCTGAGTAGAGTTCAGTATTTTAACAAGGCAAAATAGGGCTAAAAAAAGGGATGGACTAGCCACTAGGTGCAATGAAACGGAGCATAGAATAACTAGAACTAATTCAACAGAATCAAAGGCCATATAGACAAGCTAGGACAAGTGACAACCTCAACTCCTAAATCAACTCCCTAGTAATGCTGTTCTCTTTCTTCCAGTAGTTTACTAATTGTTATTCCTTTTCCTATTTTCATTCGATGCCAGTGTCCTCAACTCCTAAATCAACTTCCTAGTAATGCTGTTCTCTTTTTTCCAGTAATTTACTACTAGCTATTCCTTTTCCTATTTTCATTAGATACCAGTGTTTTTCTTATTTTTTGTCTTAGAAATGAATTGGAAAATATAGATGAAAAATTTAGAAGAAAAAATTACTCATCTTCAAGAGTCAGGAGTTAAAAGAATCTAAATATCCGAGACAACTTGCCTCAGCAATGCAATGCCACTGAGAGGCATGAAACTGGTTTCGATGGAGTAGATCAATTTCATATATAAACTTGTATGTATACATTGAAGTAGAGTTATTCCTTCTGTAAAATATTGAGCAATTATAATTCCAATGTGGTATAACCAGCTTTATCTTTATCCACTTGCATATTATCGCATCAGTTATTATCTATGGATGCTCAAAATAATTCATTACCTTCGTTTTTATTTGATTTTATCCTATCAG**GGGATTGGTTGGTTGATAAACTATCTACATCTATGGAGAGTGATAGTAATGTGAAAGATGCCATTTCCTTTGTGGAGGTTGATATCATTACACCTGCGCAGAAATTGTTGGCAAGGCGGTTGACATGTGACATAGTACGAGGAAAAAGCTTGCTTGTTACAG**GTAGTGAATTAATTTCTTTATCTACCTTTTTATGCAAATTTACATCTGATGCACTTGCATTATTTTTATCATTGTACAAACTTAAAGCTGACATCATATCAAATGATTAAG**GTCCAAATGGAAGTGGGAAAAGTTCTATTTTCAGAGTCCTTAGAGGTCTTTGGCCTATTGTGAGTGGCAGACTTGCCAAAGCATCTCAACTTAATAATGAAGATAGTGAATCTGGTTGTGGCATCTTCTATGTTCCTCAACGACCATACACATGCTTAGGAACCTTGAGAGATCAAATTGTATATCCTCTCTCTCACGATGAAGCAGCACTCATGACATTAAAGTTGCATGGCGAAG**GTAAAGTACTTGAATACGTTGTAAATCCTTGATGTTTTCAAATGTCTCCTGTATACATGGTTGAAAGTCTAAATTGATGCATCTGGTAAGGCATGCATACGTTTTACACTCCTAACATTTTGGTTAATCAACAAATGAGGTTTCACTATTACATATATACGTATGAATGTCGAACGTAAAGTATATATGTTTATTTATTAAGGACAAACAAATATATGGATGTTGAAAATTTTGGTAAAAGTATGTTAATGATAAAAGTAGTTCTTTTTTCATCTTTGGTGCAATTATTATATATGTATATTTTTTTTGTTGAACATGAGTTGAGTGATTTCATTAACAAGGGAGAGAGAGAATCTAGCATTACAATACCGTAACTCAAAACACAACCCATAGGAGGCCTGAGGGTACAATCCATGAAGTGTTACAGTACAATGGAATTATGGGAATGGAATTTCAGCTCCTAATTGTGGGAGGGATGCGCACCAAATCTCTTCATCCACATCAGGCAGGAAGTGAGCTGGACTTGAATCTCTCATTTTGAAGAGAAACATGGGCTTCTCCATGCTTTGGGTTCCAAATCTAGATTCTTCACACACAAAAGGTTGGAGAAGTCTTGAAAGTTTTCTACTTTGGCTCATTCTGAAGATCTAATCCAAGAAACCAGCAAACAGACCTCAGGTAGGGAAAGACCAAAATTGAAGAAAAGCTCAGATTTGGACACAAAAAAGGCAAACCTCTACAGAAAAACTCTCCAAATCCAACCAAGAAACGATTAATCTAAAGTGAAAAGCATTAAAAAACTAAAGAAAGAAGGGGGATGGGAGGTATCTCATGAGGAAGAAACTTGAGAGCGAAAATAGAGAGAAAAATGCAGTGAGAACAAATATCTTTTGATACATTTTAAAATGTTGTGAAACTGAATTTGTTGGGGAAAACAAATGTTTGCTGCTCCTCTGCTCCATTTTTTCAGGGAACCTTGGAATTCTTGCTCTTGCCCTCCCTCTTTCTCTCTTACTTTCAACTGTGGTCATTCAGGGAGATGCTAAACATTGGCTAAATGCCAGATTTAAGTTTTGATTAGTTTTCAATGTTCAAACATCTTCATCTAAAGATGCCAGCTCCTTTCATTGCATACAGCTATTTAGGAGCCTTTTTTTTTTTTGGTTAAGAAGTACTGAATCGTCTCTTTTTTATTGTTCAAGTAAAGTTTGTAGAGTGATTTTGTAATTTGGATGCTCAGTGCTCATAGTGGTGTTATGATTTTCTGAGATGTAATTTCTCCGTTCTGTTGCCCCTCTTGTATTCTTATTGTGCTAAATTTCAG**ATAAAATATCTGGTGACACCACAAAGATTCTCGATGCACGCTTAAAAGCTATTCTGGAGAATGTTCGATTAAATTATCTTCTGGAAAGGGAGGAAGGTGGTTGGGATGCTAATTTGAATTGGGAAGATATTCTCTCTCTTGGAGAACAACAGAGATTAGGCATG**GTGAGTAAAGCTAATCTGTGCTATCTTCATATTTAAATGGAAGGGACAGGATTGCACAATTGGATTCCTAAACTTTTTGACTTGATGCTTTTGTTTTACAAG**GCACGCCTATTCTTTCACAAGCCCAAATTTGGCATTCTTGACGAGTGCACCAA**GTATGTTATTTTTCTTAACCTCACCTGAAATTTTATCAACTACAAAAATAATGGTATGTTAATCACGTGTATTTCTTTTTTCTTAG**TGCCACAAGTGTTGATGTTGAGGAACAACTTTATAGGCTTGCCAAGGATATGAACATCACAGTTGTTACATCCTCACAG**GCAAGTGGCATGTTTATTTTAATGGAAAATGAAACCATGGTGTATGTGATCTGATGAGATCAAGTTCTCTAGCAGTTGCATCGTGGTCATGTTTAGAACATCTTGTTTGTTTTGATTTTATTTTGAAAAGCACAAACAATTTTCATTGCAATACTTACGATAAATATATTAATTTACAGAAATGTGACATGGTCATCCTCCAACTAGGTGTTTTAGCTTTTGTATTTTTACTAGAATTTAATGCTCTTCCCTCAACTGTTCCTTTTTCTTTTCAG**CGTCCTGCTCTAATACCATTCCATTCTGTGGAATTACGGCTTATTGACGGTGAGGGTAACTGGGAGCTTCGTACAATAAGGCAATAACTTGACGGTAATATCAGCATTTCTTGATTTAAGCTGTGTGATTAATGGCATTAGATCTCATATGCAGATTACCATTCAGAATAGAATATATTTATACAGATTTCAGTAAGTTGTTTTGCTTAATGCATGAGTATGGCCGAGGGAACCTGGCAACCATGTTTTGAGTACTCCGTGAAATATGATCTCATCGTTTTTCATATAAATCATCAGAGCATAGGATGAAAAGAAAAGCCGATCGCATAAGCAGTATCTCCAGAATCATTAAAATTAAGGATAATCATACAGGGATGTCATTTAGCATAGTGATTCTGTACAGTTT**

>HbABCD2 scaffold0173(1295260-1320534)

**AGAAGAAATCTTCTTATGTGAAGATGATAGTTGAAACGCAAGCTCATAGATTGGTGCTTCTCTCTGCCTCTACTTGTATTTACACTCCCAGTTCCCAATCTCATAAAAGCTGTCATGTTCAAGCTGGCCTCTTTACTCCAAGATTTCCATTACCCATTTCTACTGGGCTTCGCACATGGACATATCGCTTCAAATTAGCTGTCACTGACTCTTCCCTCTCCGCTCCTCCTACTCCACCACCGCCTGACAAG**GTTTAGACTCCTGTTATGAAAATTTTTCGTTGTTTTGGAAATTTCCTGTTTTGGTATTGATTGGTAAAAGTTTTTTCAAG**GATGATGCACAAAGGAAGGTGCCTGAGCTACAAACGCTATTCAGAAGATTTTGGAAAGTGGCTGCTCCGTATTGGTTCTCAGACGACAAGGTCCAAGCCAGATTGCAGTTGGGAGCTGTCTTTGCTCTCACGTTGGCCACCACTGGTATCAGCGTTGGATTCAATTTCCTCGGCCGCGACTTCTATAATGCCCTTGCTA**GTAAGGGTTTTTTTTTTTTTCGATGCTAAGCTAATTCATTTTCTTTTTTTTATTTTTTATTCTTTTTAAGAAAAGGATTAATTGCATAATATCTCTGAGATTTAGCTTGAAAAGCACTTTGATCCTGTTGTTTTAAATTAACAATTTTAGACCAATAAGCAATATAATGGAGCTTTCTTTAGGGTTGATATGGTTTTTTTCATTGCAATTCAGGGCTTGTAGTTTCACCCAATTAGCACTTAAGGATCCGCATGCTAACATGTCAAACAAAAAAATAAATTTTTAATGTTCCAAGTGATCATTTTTAATGCCATTAGGTGTTTTGCTACTCAAGTGCCAATAGCACGAACCCACATACCCTGAATTGCAACGAAAAGACCATAGGCTTTGAATGCTTAAAGTGCGAAACAACAAAGTATTGAAGTGTACCTTTCCCTTTATTATGCTAAAAGAATAATGGCTAGATGATAATATTTTTGTTTGTAATAGTATATTTTTGAATTGATGTAATAATTTAATTTTTTGAGTGATGATAATTTTTTTTTTCTAAAAAAAGTATAGTAGACTATATTTATTCATTAGATTTTGTATGATACATATTAGAATAGAAGGCTAAAAAGAATGAGAAATTATAGATAATTTAGGAAATCTAAATAAATATAATACTTATAAAGAAGAAAGTGGAATAAAAAGGGAAAAAAAAAAGATAAATTGCCACATGGATAAATAAAGCATTTCTAAGAGATGTGTAATTTAATGCAATCTTCCTTAACTTTAGATATAATGTATAGATAAACAGCATTAAATGAACATAATAATTAAGGAAGTAATAGTTAAAAAAAATTAAAGAAATGGGTTGAAAACTAAATAGATTTTGCAAAAATATAATATAATATAGTAATATAACTTAATAAATTATATTTATAATTGAATTTATATAATTATGTATAAGTTAAAATTATGAATATATTATTTAGCATTTAAGTGTATTTGGTATGTGGTTCAAGGAATTGGATAAGAAATAATAAGTGATAAAATTTATGCATGACCATAATATTAGATAATATATAATACGTAAATTCAACTCAACTCAACTTGACTAAGTCTTTATCCCAAAAATTTGGGGTCGGCTATATGGATTCGCTTTCTCCACTCTGAACGATTTTGGGTTAAATTCTCAGAAATGTGTAATGCTTCTAGGTCATGTTGTACTACTCTCCTCCAAGTCAATTTAGGTCTACCTCTTTTTTTCTTTCTATTCTCTAACCTAATGTGCTCTACTTGTCTAACTGGAGCCTCCGTATGTCTACGCTTCACATGACCAAACCACCTCAATCTCCCTTCTCTCAACTTATCTTCAATTGGCACTACTCCTACCTTTTCTCTAATACTTTCATTACGGACTTTATCTAGTCTAGTATGGCCACTCATCCACCTTAACATTCTCATCTCTGCAACTCTTATCTTAGATGCATACGACTCTTTCAGTGCCCAACACTCACTACCATATAACATAGCCGGTCGTATGGCTGTACGGTAAAATTTTCCTTTTAATTTATTGGGAATCTTACGATCACATAAAACTCCCGTGGCACGTCTCCACTTCAACCATCCGGCTTTAATCCTATGACTAACATCCTCCTCACATCCCCCATCTACTTGAAGGACTGAGCCTAGATATTTAAAGTGATTACTTTGGGACAGTGCCACTCCATTCAAACTAACTCCTTCCCTATCACCAGTTTGGCCTTCACTGAACTTGCAATGCATGTATTCTGTCTTCGTTCTACTTAACTTAAAACCCTTTGACTCTAGAGTACTTCTCCAAAGTTCTAGCTTCCTATTGACTCCTTCTCGTGTCTCATCTATCAGAACAATATCATCCGCAAACATCATGCACCAAGGAATACTCTCTTGTATATGTTTCGTCAGTTCATCTAAAACTAATGTAAAAAGGTAAGGGCTTATGGCTGATCCTTGGTGTAATCCAATTGAGATCGGAAAATCTCTTGTGTCCCCTCCCACTGTGCGCACAATAGTAGTTGCTCCTTCATACATATCTTTCAATACTTGTATGTACCTAATAGATACCCTCTTTTGTTCTAACACATTCCATAAGACCTCTCTTGGAACACTATCATAAGCCTTCTCCAAATCAATAAAAACCATGTGTAGATCTTTCTTCACATCTCTATATTTCTCCATCAAGCTTCTAATGAGAAAGATCGCTTCCATAGTTGAACGACCAGGCATGAAACCAAATTGATTGAGAGAGATAGAAGTATCATGACGTAGTCGATGCTCCACAACTCTCTCCCACAACTTCATAGTATGGCTCATGAGTTTAATTCCCCTATAGTTTGAGCAACTCTGTATGTCTCCCTTATTTTTAAAAATAGGTACTAAAATACTCTTCCTCCATTCATCAGGCATTTTCTTTGAGTTTAGAATCTTATTAAATAATTTAGTTAACCATGCCACTCCCATATCTCCCAAATACTTCCACACTTCAATTGGTATTTCATCGGGTCCACAGGCTTTACCCACTTTCATTCTCTTAAGTGCTTCCTTTACTTCTAAAGATCTAATCCTTCTAGTATAATTTACATTCTTTTCTATTGTTCTATAATCTATATTCACGCTATTTCCATTTTGACTATTATTAAAGAGATCATTAAAATAATTTCTCCATCTTTCTTTAATGTCCTCATCTTTCACCAACACTTTTCCTTCTTTATCCTTAATGCACCTAACTTGATTGAGATCTTGACATTTCCTTTCTCTACTCCTTGCTAATCTATAAATATCTTTCTCCCCTTCTTTAGTTTCAAGTTTCTCATATAACTTTTCAAAGGCCTGTGCTCTTGCTTGACTTCTGCCTTTTTTTGCCTCTTTCTTTGCTATCTTGTACTGTTCATATGCTTCATTATTATCACATTTAGGTAATTTCTTATACCATTCCCTTTTTCTCTTCACTGCCTTTTGTACTTCCTTATTCCACCACCATCTCTCTTTTGAGAGTGGTTCCTGTCTTTTAGACTCTCCAAGTACTTTTCTAGCTACTTGTCTAATCTTTGATGCCATCTGTATCCACATATCATTAGCCTCCATATCCAGCTTCCATACTTCGGACTCGAGAAGCTCATTTTTGAACTTCACTTGCTTTACTCCTTTGAACTCCCACCACTTTGTTCGAGCTACACTATTTCTTCTGACCTTACTTGAATTGTTCCTAAACTTGACATCCAAGACTACCAACCGATGTTGACTTGTTAAAGCCTCTCCTGGAATGACCTTGCAATCCTTCTATAGAGCTCTATTTGTCTTCCTAGTTAAGAGGAAGTCGATTTGGCTTCTATGTTGCCCACTTATGTGATTCTCTTTTTATAAAGTAGGTATTTGCTAGTATTAGGTCGTATGTTATAGCAAAATCCATGATGCTTTTTCCCTCCTTATTTCGACTGCCAAAACCAAAACCTCCATGAACATTCTCATAACCTTGTCTATCACTTCCTACATGCCCATTCAAATCTTCACCAATAAAAACATTATCTTCATTCGGTATGCTTTGCATTAAATCATCCATATCTTCCCAAAACCTTTGTTTACTCTCACTGTCTAGTCCTATTTGTGGGGCATAAGCACTAACTATATTTATTGTTTCTCCTTCTAGTACTAGCTTTACTAGTATAATTCTATCTCCTACTCTTTTCACAGCTACTACTGCGTCTTTCAATGTCCTGTCTATGATTATACCCACTCCGTTCTTGTTTCTCTCCTTTCCGGTAAACCACAATTTGTACCCTGAATTACCCACTTCCTTACTTTTCTCTCCTACCCATTTAGTCTCCTGAATGCAAGCAATATTCACCCTTCTCCTTTCCAAGGTATCCACAAGCTCCATTAATTTTCCTGTAAGTGATCCAACATTCCAAGTACCAACCCTGATCCTCCTCCTATCCTGCTCCTTCCTAATTGGTCTCCTTCTATGATATCTTCTATTATTTTCTATGTCTATCTTGTGTTCTGTTCCACTATTTGTTCTATTATCTGTCCTATGGACTAACTTCTTTACCCACACCCGTCCATGATGTGGGAACCCTTGCTCACTTAACACCACACCCGGGCGCCGGCATGGCGCGTCGCTTTCGGTGAACGCCCTACACCCTTGCATATTTATCACTACACCCGGGCTCCGATGTAGCGCGTCGTTAGTAGAATTTTTTTTTTGGGCCTCTCCTTCCTCTCTCCCCCCTTCTCCCTTCTTTACCCAAGAGAAAGGGTTCTTGTTTAACCAAAATTTGAGGATTAAATATTAACACCTTGAATTTTTTACCCTCTCTTAACTTTTACCAAAAGGGGTAAAGAAGGTAAAAGTTTCAATGTTGAATTCTTAACCACCTTTACCCCTCTGCCAAACACAATGGTTAGTATATTCTTTTTTGGTTAAGCCCCTCCCTGTAAGTTAATTGATGGGGGGGAAAAGTTATTTAATTACTTTTATTAGTATATATCACAATTAAAATTACTAATATAATATTAGACTATTATAAATAAATAATTATAACATTCACCTTTACTAACATAATCAATTGTTACCATTACAACTCTACTCTCGATCGTTACCTCCTTATTTATTACACTATTAAGCATTACACTTGTAAATTTTTAAACTCTATCCTTTAACCTGAAAACAAAATGCAACTAGGTGAAGAAGTGAAATAAAAAGTTACTTGTGAAACAACTAAATGTATTTGGTCCAAGGTTACAAAAGTACAAGGTGAATGCCATGTATACTTATTTATTATATATATATTTTTTTATTATGTTAATAATTTTAATCGAGGTAAATGCTAATGAAAATTAAATGTGCATGTGTGTGTGTGCCCTCCCCCCAACCCCATTCCTGGAGGGGAGAAGTTAACAAAATTACACTAAAAGGGTTATCTGCCTTGATTCTTACCTTCCTTTTATCTCCATTGCTAATTTTAGAACAATAACAATGAAAATGTATGTTATTGAGAACTTCAGGGGCTTTTATAACTATTTCAAAGTAAAACTAATGCTGTAATTTTAAATGCAAG**ACAAGGATCAAGAACAGTTCACCAAGCAACTGCTATACTACCTGGGAGGCTTTGCTGGAGGAATTCCG**GTGAGCACTCAAAAAAAAAAAAAAAAAAAAAAAAATTGCTTCCTGCAATTCCTTAGGTATTACTCTGCTAGTCTTCTCTCAAGTCTAGTCACATCTTTGGGTACCATCTTTCATACTTTTGAGTTTTTGTTCTGATACTATTTGTATTCTTCCCTTTTACTAACTTTTGTACCCATCTTTATCATTATTTTTTCTTTTTTGTAG**TTCTTTGTGCTAAGAGATTATGCCAGAGAAATCCTTGCTTTGAGATGGAGATCTTGGATGACAAAATATTACATGGAACGCTATCTGAAGAATCAAGCATTTTACAAAATTCAATCCCAATCAATCATTGATAATCCCGATCAGCGAATTGTTGATGACCTAAGTTCCTTCACAGGGACAGCCCTTTCTTTCTCATTGATACTTTTCAATGCTGCTGTAGACTTGATATCATTTAGTAACATCTTGTATGGCATTTATCCCCCATTGTTTGTTGTCCTTCTTTTATATTCTGTGGGTGGAACTGCAATTAGTGTTTATCTTGGAAGG**GTAATTTTCTTATCTTCAGAACAATCATCTTTCCACTAAATGATTAGTTTTTGGTTTGAAATATGAAAATTCTTATATGGAACTGAAGCCATTTTACTTTCAATTTCAG**GGATTGGTGACTCTGAATTTCTTGCAAGAGAAAAAAGAAGCAGACTTCCGCTATGGACTTGTACGTGTTCGGGAAAATGCTGAATCAATTGCTTTCTATGGCGGTGAAGAAAATGAAATGCAACTTCTGCTGCAGCGCTTCAGAAGTGCTTTTGAAAATTTAACA**GTTAAGAATATTTGTTTTGATCCTAGATTGTTTACTTATTTCCATTTCCTTGTTATTTCAATCTCGGGCAATTGAATTTTGAACTTAATTCTAGTTTGAACCTAGATTGGGAGGGAAGGAATTGGGAGTCCACTATTAGACTAGAAAGAAATAGCCATGGCACATAGAAGAGGAGGTGTTTATATTAGTCAATCCGTTGCCCTCCCTCCCTTCATTTCTTTTCAGTACTAGAAATTCACATTAAATACTTGTGCATGAGTGCAATGGCAGATGTACTCTGTAGGTGAGAGTTCGATTAATGAAAAGACAAGTGATGCATATGATTATCAAGCAACGGAAAATCAGTGATGGTATAATAGAATATGCAAGCAGCCATTAGCTACTTAGATAACACGTGAAAGGTGATAAAGCAATAACAAGTGTAGTGCTATGGAAAGTCAATATTTATGTTTAGTTATATTCCTAATTAGCAGATTCTTAATTAGTAGACTTTATGTATTCCTATTTAGATTCCTAAATAGGTAGGAGTTTTATTCCTAATTAAATAGAGTAATTATAGGAGGTGTGTGTGTGTGTGTGTGTGTGTGTACATATGACCTCCTATTTGAGAGAATGAATGAGAATAATAAAGATGTAGTCTTTTATGGTTCTCTCTCCCTTCTCTTCCTTTTCTTTCCTTTCTTTCTTTTTCTTCTTTTTATTCTTAAATCTTAACATAACATGTAGTTTTGGCTCCATGGACAATCTAACAGGGAGTGAATTTATTTGACAATAAAAATTATTTATCTTTGTTAATTTATTTGTCTTTCTATGCATAGTGCAATGATGCAAGCAATATGGTGAAGACCCCATTTAGATTAGTTGCTAGCAGTTAGCTATTAAAGGTTGATCCCCAAATTATTTGGTTCCTAGGGCAGGGCTATAATTGTATGCAAAATGACAAATCAGGACATAATTTGCTTTTTTCTTTGATTATTGGACAAATAATAGTTTCATGCTCAAATAAATTTCTATCTTTAACTTTTGTGCTATCACAACTATTATTGCATTTCCACTGAACCTTCATATACACATAATTATGTATGTATAATTTACTTAGTTGCTAATTTATGCATTTTTCATTTTTTTTTCCCTGAATCTTACACAGGCACACAGTAGGCAATACATGGACTACACCTGTATTAGTAAATATGCGCTTTCATGCACACATACACAAATACACACATATGCATATAAACTTACAGGGCTGTGCATGATTTGTGGATTGAAAAATCCCTGGAACCAAGCCGAACCTTGGTTCTTTTTTCTTTCCTGGAACCAAAGTCTTCTATTATTCAGTTCCAATTATTGGTTTGGGTCTTTAAAATTTAGTTTTTTTTTTTAAAAAAAAAAAAACAAAGGAGGGAGAATATCAGAAAGTTCAGCTTTGAACAAAGGTTCATTGTCACAAACTATGTCAATGTTGCAGCACACTCTCAAAAATTGATGGCGTTGAGAAAATTGTGTGAGAAGGACAAATTTAAGGGTACGTATGAGCTCCTTTTTTGGTAATAAAATGAAAAGCAAATGGCATCTAATGAGAAGATAACAAGACTAAATATGTAGGCTAAAAGTGAGGAGGAGGTAATAGGCTGTTATCCAATGACTTGAAAAAGCGTTGAGGAACCTAAGAAGAGCATGATCCATTGTTGCTAATAGGCTGTTGTGGAAGCCAATATAAAGATACTACAATACAATACCTTGGGCTATAATTTGCTAGTGATACGATATTTCTATCAGTGGCAAAAACAGTCATGGATGAAGTTTCACAATTGTGACCCATTATGAACCTCTAAATGTAATCAAGCATACACAATAAATGCTGTGGGCCCGTGGCCATGATATCATAGTGGTAACTATATCCCATTCTAAAGGGTGCAGTTCTATTAAATACCATGGAGATGCATCTGTGGTTGAGGTAGATGTAGAACCCTATGTTTTAGCACCTAAATAATTTTTACCCAATGGAAAATAAATGTGCCATAGAGTTTACATCCTTATATGACTAAAATAAGTTTCAATTCATTATAGTTGGGCATCAAAAATAAAAATTGGCTTACAACACCCTCCTAAAATATAATAACTTGTTGAACTTCAGATTCCTTAATTGGCTAGAAGAAGAATAAGAATTGTTATTAGGTTCCTCCCTATGGGACAGATTTGGGTCAATTAGGGCTCTAGCTTCTCATGTGAGAAGGTAAGTATACTGCTGCATCAGAAGTGCTCCCAGCAATAATAAATGGCATGACAAGCTGCTATAATCATTTAAATATTTGTTCTCTGTTGTTGCCTTTGGTTTTCTGATTTATTATTGCTACTTTTCTATGCAAATCTGTTCTCGGCATACAATTTCACTTCCTGACATTTATTTATTAAATTTCCAG**CAATTGTTGATATCTTCTAGAAATCTAGAGTTCTTCACCAATGGCTACCGGTACTTAATTCAAATTCTTCCCGCTGCTGTTGTTGCTCCTATGTATTTCTCAGGCAAAATTGAGTTTGGTGTTATTAATCAGTCAGTATCTGCTTTTAACCATATCCTTGGAGATTTTTCTCTCATTGTTTACCAGTTTCAATCCATCAGTGCTTTTTCAGCCGTCATTGATCGATTAG**GTACTATGCTTGTTGTGCACCTCCCCCTCTCTCTCTTTCACACGCACACGCACACGCACACGCACACACACATGTAGTCATTCATTCACTCAGGCTTGGTTACAATCCATGAAGTAAGTGAAGTAAGACATTATTTGATCAGTTTATAAGGGAATTTTCGTGGGAATTAGATTACATATTTGCAGTAAGCATGCACTTGTGTTTCACTTTATAATGAAGGGATTGGTTTTTAAGATTTATAATGCAGGGATTGGTGTTTAAGAGCAATTATTTGAAACTTGTGTTCCTCTAGTTATTTTGCGTCCCTCCTGAAACTGGAATGTATAATAAAATGGTTGTAATATTTTCAATGATCTGAGATATCCATGCATCTATTTTTCTTTTGGAGAGACTTTATCTTCCTGCCATTCTTCATTGTTATTATTCCATGTGAAACTTCTTGTCGTTAGTGAAGTTTTTTCTTGGGAGAAAAGAGTTCTAATACTGAACTGATGCAGTAAAAGAATCCAGTTACTTTATCATTTTATGTACTTTGTTTTTATTTTTTGCCCTTTGATTGTGCTGTTTTTCATTAGTCAAATACTTTGGTATTTGCTCCATCTTTTATTGTGTTTCTTTTCTATTAAACATTTGTCGTGCTTGATGTCCTATACTCTCTCTCTCTCTCTCTCTCTCTCTCTCTCTCGCCACTTATCATGTTTCCATATGATGTATTTCATGCTTACAATACAGAAACTGGACAAGGAGATGCTTAATGTGATAGTTGGTCTTGCTTATGAATCGTTGATTTTAACATTGTCACTTTCTATATGTAG**GTGAATTTGATGATGTCTTGGATAGCAGCAGCTCTAAGCAACTGCCTGAGTTATCAGAAGAGATTTCTCTTTCATACTGCAATTATAGAAGTTCACTCATTTTGGAGTTTAATGGGTCTATTCCAGTTGACAGTCGCCAAAAATTGCTTAGTATAGAGAATTTGACTCTACAGACACCAACAAGTAAAGCTACACTGATTAGGGACCTGTCATTGGTCATCAATGAGAAAGATCATTTGCTG**GTCAGTGAATCAGTTTGTATTTTGATTTTTTTAACATGGCAAGCAAGTAAGTTTGCAAGGTATGAAATTATTCAGTGTTTCATGGGTATCTTAACAG**GTAATGGGACCTAGTGGGAGTGGTAAAACATCACTGTTAAGAGCTTTATCTGGTCTTTGGAATGTTGGAAGAGGAAAAATAACTTTCTATCTTAATGATGGGGATGATCCTCAATTGCCCACGTCTTCAGAACTGCCTGCTAATGAAATAAATACTTCACATGAAAATGCTGGGGAACTTGAAGGGCCAATTAATAGAAACTCTAGAGGCATATTTTTTCTTCCTCAAAGACCATATATGGTTTTGGGAACACTTCGTCAACAATTGCTTTACCCTACATGGGCTGACGATAAAACTCCAATGTCAGATGGTACTAAACCAGTAG**GTATGTTTTCTAAATTATTATTATTCTTATTATTTTCTTTTTGTGACACAATGCATTATTCTTTATATAGAAGTCCATGACTAATGTTTCTGAGAAACCACACAATTATATAACTCATATGAGAGCTTAAATTCATGATATGTGTTCATTATGTAACAGACCTATAATGCCCTCTGACACAGGGAAATAGGGAGAGAAAGAGATAGAAATGGAGTAGGTTGAGAAAAGAAACAGAAAGGGGAGAATATTTAGAGAGAATTAGAAAAGAAAGAAAAATAATTCTTTTTATTGATTCTTGGAATGAATCTCCTCTAGTACATTTCTCTGATTTATACAGCAAGGACTCCCTCTATACTAACTGTTCCCGCCCTTTCCTGTCAAACTACTTTCAGTTGACTCTCCTCTAACTACTCAGACAACTCTAACTACCTCAGTTACACTTCAGCTACTCTACTCTCTTCCTCATACAACTATAGCTCCATTCTTATTTTTTTCCTCTCCAATATCAACCTCTGCTATATGTAACAGTGTTTTTGTGATTAAATAAGCTAACATTGTTTTACAACCAATTTGATTTGAAATTTTTTTTCTCAAGGCTTTATTTGGGCTGATGAGAATTTGAAATGAAGTGGTATTTGAATTTGACAATATATTGATATTCTGTGTTTTCACTTGAAGTTGAAACACAAATCCAAGTCCATATCAGTCTTTATTCAATTGCATGCTTATCTAAATGCAAGGTTAGTTTAGAATACATAGAATTCATTATGTTTAGCATATCATGTATAATTACAATTCATTTTGAAGTATATAGTATGTATGTGTTAGGGCTGAGCACAGTTTGATCTAATCTGAGCATTCGAACATGCTTGAAACTATTTCAAAAGTCAGGGGTATCTAAAATGATATCTCCAAAACATTACTGTGACATCAGAACTCCATCCTGTATTGTCCTGAAAATTCTAGATATTTCAGTTCGGGGATTTGAAGATTCGGATGTTGTAGGTTAATAAGAAATTATTTATTGTCCTAAATAATCCATTTTTAATAAAATCTATCAAGATTTGAAATATGCAATAAAAATCTAAAATATTTGATAACATACACATTAAACTTTAATTTCTTCACTATAACAATTCAACAATAATCTAAAGTTCCAATATTATAAATTTGCAATGTGCTAAAACTAAAGAGAAAAAAACTAAATCATTACTACAGCCAACATTTTAATAAATTATACTAATACCATTAAAAGCATTTAACTAATGCTTGTTTCATTAAATCAAAGCATTCAAAACAAATTGGCCTATTGGCTAAATAAATCCAAACTTTTACGTTAGTTGCTAATTATATTCATGTTATGGAAAGTCAATCACTATATTATTTCTCTGTATATTCTGTATTCTGTATTCCTATTTAGGACTTCTTATTTAGGATTTCTTCCTAATTAGTAGAACACAATTATAGGAATCAATTGTATATATATACCCATGTACAGATTAATTGAAATCAATGAGAATCATCACTTTCTACATGGTATCAGAGCAGGTCATTAATCTAGGGTGACTATTTGTTCTCACTACCCAGCTCAGGAGAGACCTTGGTCGCTGACCGGCCGTTTCAACCCTGATCAACATTGCCGTCGTCGCCTCAACCCCCCTCATTCCACCACTGTGTGCTGTTGCCTGATCCAGTATACCATTAAATGACTTTTGAGGTTTGGCTCTCAGATCCTATTTCAGTATTTGCTTGATTTGTGATTTTAGGTTATTTTGCTGCTATAAGCACTTTGGATTAGCGTTTCGGTTAGTTTTCTTTGTCTCAACAAATGGCAGACAATAAGAATGTTATTTCTGATGTGATTCCGGTGATGACTAAGATCACGGAACACAAACTTAATGGTTCGAATTACCTGGAGTGGAGTAAGACTGTTAGGGTCTATTTGCGTAGCATTGATAAGGATGATCACCTTACTAAAGATCCACCCACTGATGATACACGACAAACTTGGCTAAGGGAGGATGCTCGGTTGTTTTTGCAGCTTCGGAACTCGATTCATAGTGAGGTAATTAGTTTAATTAATCACTGTGAATTTGTTAAGGAATTGATGGATTACTTAGATTTTCTGTATTCTGGTAAAGGGAATATCTCCCGTATTTATGATGTTTGTAAGGCATTCTACCGTGCTGAGAAAGAGGATAAGTCTCTCACGGCTTATTTTATGGATTTTCTGTATTTATGATGTTTGTAAGGCATTCTACCGTGCTGAGAAAGAGGATAAGTCTCACACGGCTTATTTTATGGATTTTAAACGGGTATATGAGGAACTTAATGTATTGTTGCCTTTTAGTCCTGATGTGAAAGTTCAGCAGGCCCAACGGGAGCAACTGGCTGTTATGAGTTTTCTTGCAGGCCTTCCTTCAGAGTATGAGACTGCTAAATCTCAGATTCTTTCCAGTTCTGAGATTTCCTCTTTGCATGAAACATTCACACGGGTCCTTCGTACAGAGAGTACCCAATCTTCACAGCCTGCCAGTAGTGCTCTTATTAGCCGTAATCCAAATGGACAACAGGGTAATAGAAGAGGAAGTAGAGGAGGAATTACAGGCAACAGAAGTAATCAGCGTAATGGAGAGGCTAGTTCTAATCAGGACTCAAGAGGAGTCATTTGTTATTATTGCCATGAGCCTGGCCATACAAAATATAATTGTCCGCAACTTCAGAGGAAAAATCAGCGATCACAGATGGCAAATATGGCAGCAGAGAATTCTACAGTATCTTCCTTTGAGAAAACTATTTTGGTATCTGCAGAGGATTTTGCACAATTTTTCCAGTATCAGGCATCTCTAAAGCCTGCCAGTTCCCCTGTCACTGCGATCGCTGAGTCAGGTAAATCCACTACATGCCTTGTGTCTTCTTCATCTAAATGGGTTATTGATTCTGGTGCGACAGATGACATGACAGGTAATTCTAGTCTTCTATTTGCTTTTCATTCTAATCTCACTTCCTCTACTGTTACTTTAGCTGATGTTCTACTTCTTGTGTTATGGGTTCTGGAACTGCGAACCCGACTTCGTCAATTTCTTTGTCTTCTGTTTTGTGTCTACTAAAATTCTCTTTTAATCTACTTTCTGTTAGTAAACTTACTCGTACCTTAAATTGTTTTGTTTCCTTTTTTTCTTGACCAGTGTTTGTTTCAGGATCTTACGACGAAACAAATTATTGGTAGAGGACGCGAGTCAGGTGGTCTCTACATTTTGGAAAATCATGTACCGCGGTCGCTTGTTTGCTCCAGTACCTTAACACCTCTTGAAGCTCATTGTAGATTGGGTCATCCTTCTTTGTCTACCATGAAGAAGCTGTGTCCTCAATTTCAGTCTTTATCAGTACTAGAATGTGAGTCGTGTCAGTTTGCAAAACATCATCGTTTGCCTTCTGTGTCTAGAGTCAATAAACGGGCTTCATCCCCTTTTGAGTTAGTTCATTCTGATGTTTGGGGTCCTTGTTCTGTTACATCTAAAACTGGATTTCGTTATTTTGTTACTTTTGTTGATGATTACTCTCGTGTTACCTGGTTATATTTAATGAAGAATCGTTCTGAGTTGTTTTCTATCTTTTGTGCCTTTTGTAATGAAATCAAAACTCAATTTAATATTTCTGTGCGCATATTAAGAAGTGACAATGCTAAAGAATATTTTTCAGCACAATTTCAGTCTTACATGACACAAAATGGCATTCTTCATCAGTCTTCCTGTGTGGATACCCCATCCCAAAATGGCGTGGCCGAAAGAAAAAATCGGCATCTTCTTGAGGTAACTCGTGCTCTTCTTTTTCAGATGAAAGTTCCTAAACACTTTTGGGCGGATGCAGTTTCTACGGCATGTTTTTTGATCAATCGTATGCCGTCTTCTGTCCTTAATGGGGATATTCCTTATACTACTTTGTTTCCTACAAAATCTTTGTTCCCTATTGAACCCCGTATTTTTTGTTGTACCTGTTTTGTGCGTGATGTTCGTCCACAGGTTACTAAATTGGATCCAAAATCTCTCAAATGTGTCTTCCTTGGGTACTCCCGGCTCCAAAAAGGGTACCGTTGTTTCTCTCCTACTCTTAATCGTTATCTTGTTTCTGCAGATGTCACATTTTTTGAGTCCACTCCATTTTTGCCTCCATCATCTGTGTATGAGAGTCAGGGGGAGGAGGATGATCTCTTAATATATACTGTCCAACCAATGTCTAGTCCTCTCCCATAGCCTGTTCCTTCTGTTTCTAGAGCTACTCGACCTCCCGTTGTTCATGTTTATTTCAGGAGATTGGAGATTCCTGACTCAGATCCTCTACTAGCTACTTCGTTGGGAGATCCTGTACCTCATACTGATCATGATTCTGATCTAGACTTACCCATTGCTCTTCGTAAAGGTAAACGTTCATGTACTTACCTTATCTCTTCTTTTGTTTCTTATAATCAATTGTCTTCTTGTTCTCGGTGTTTTGTTACTTCTTTAGACTCTGTTCCTATCCCTAATATTGTTGATGAAGCACTGTCTCATCCTGGCTGGTGTGATGCTATGAAAGAGGAAATGGAGGCTTTAGATGCTAATGGTACATGGGAACTATTGCCTTTGCCCACTGGTAAGAAAGCTATTGGTTGCAAATGGGTATTTACAGTAAAGGTAAATCCTGATGGTTCTGTGGCTAGGTTAAAAGCACGCCTTGTAGCAAAAGGATATGCTCAGACATATGGGGTTGATTACTCTGATACTTTTTCTCCTGTAGCTAAACTTACTTCTATTCGCTTGTTTATCTCTTTAGCAGCTACATATGATTGGCCCCTGCATCAATTGGATATCAAGAATGCTTTCCTTCATGGTGATCTTCAGGAGGAGGTGTATATGGAGCAACCACCTGGGTTTGTTGCTCAGGGGGAGTTGGGTAAAGTTTGTAGGCTTCGGAAGTCTCTTTATGGCTTGAAACAAAGTCCTAGGGCATGGTTTGGGAGATTCAGTGAAGCAGTACAGGAATTTGGTATGCAAAAGAGTAAGTGTGATCACTCAGTATTTTATAGGCAATCTGAGGCTGGTCTAATTCTCTTAGTAGTCTATGTGGATGACATTGTCATCACTGGGAGTGACTCTGCAGGTATTTCATCTCTTAAAACCTTCCTCCAAACTCAGTTTCAGACCAAAGACTTGGGATTGTTAAAGTATTTCTTGGGTATCGAAGTTATGAGAAGTAAGAAGGGTATTTTCTTGTCTCAAAGAAAATATGTCCTCGATCTATTGACAGAGACAGGAAAATTAGGTGCTAAGCCTTGTAGCGCACCAATGACTCCAACTTTACAACTGTTAGCAGGGGATAGTGAGTTGTTTGAAGATCCAGAGAGATACAGGAGATTGGTAGGAAAATTGAACTACCTTACAGTCACTCGTCCTGACATTGCTTATGCCGTTAGTGTGGTAAGTCAGTTTATGTCTTCCCCAACTGTTGCTCATTGGGAAGCCTTGGAACAAATCTTGTGTTATCTGAAGGGCGCTCCAGGAAGAGGTTTGCTATATGGTAATCATGGGCATTTGAATGTTGAATGTTTTTCAGATGCCGACTGGGCTGGATCTAAGGTTGACAGGAGGTCAACTACTGGATATTGCGTTTTTATTGGAGGAAATTTGGTGTCTTGGAGAAGCAAGAAGCAGAGTGTAGTTTCTCGATCTAGTGCTGAATCCGAATACAGAGCCATGGCACAATCAGTATGTGAGGTAATGTGGATATTTCAATTACTAGATGAGACAGGTTTTAAGACCCCCCAGCCTGCGAAATTGTGGTGTGATAATCAAGCTGCTCTCCATATTGCTTCTAATCCGGTATTTCATGAGCGGACCAAACATATTGAGATTGATTGTCACTTTATTCGTGAAAAGATTCAAAAACAGATTATCTCAACAAGGCACATCAAAACTGGAGAGCAGTTAGGAGATATTTTCACAAAAGCTCTGAATGGAGCTAGGATTGACTACATTTGTAACAAGTTGGGCATGATTAACATCTATGCTCCAACTTGAGGGGGAGTGTTATGGAAAGTCAATCACTATGTTATGGAAAGTCAATCACTATATTATTTTTCTGTATATTCTGTATTCCTATTTAGGACTTCTTATTTAGGATTTCTTCCTAATTAGTAAAACACAATTATATGAATCAATTGTATATATATACCCATGTACAGATTAATTGAAATCAATGAGAATTATCACTTTCTACAATTCAAAACTTTTAATTTTGGACAATTTAATCATAACTTTCAAAGTTGAAGAAATTTTATCCAAAACCTATAATCTAATTTGGGGATTTTCACTTTTGACACATGGGATGCTAAGTGACTTGACAAGTTATAATTTTTAATTTTTTTATTTGATTCATATGTCTCTCAAAAATTTAATTATTTTTCTAAAAGAAATAAATACATAATTATTAAATTATTAGCAATCAGTTCTTGCCTACCTCTTGGTCACTTAAAATTTCAAAATAAAGCTTGATTTTAACAGATTTTAAAGGCTTGAAAAAGTGAAACTAATTAATCATATTCTATAAGCATAGTCAACAAAGTAATTTTTTGTTATTTAAAAAAAAATCCTGATTCAGCGAAAATATACACATATTATCTTATAATTATTTTTATTTTAAAAATTACATGAAATTTATATAAAAAAATGTGTATACTTGTACTTTTTGATAATTGATCAATGGCATATGATTTGCCCAATTTCCTTTTTCACTTAAATATGACATGGTATATTTAGCGGAAAGATTGATGAGTGTTTTGATTGCTACCCACTATTCAGATATTTTTAAAATATAATTATTACAATGAGTAATATCATATGCTAGTTTTCGAAGATCATATTAAATTTATCTTGCTGGTAAGATAAAAAAAAATTTCTTTGCTTAGGTGAATTTTGTGTTGCAGGTTGCAAAAAGAGCTCTTTTTTTGCGGAACAATGGACTCATTGTGACCTTGATTGCTTAGAAGAGAGAGAGAGAGAGATAAATCAAATAAAAAAAAATTGTGACTTGTCAAGCCAATTGACATCCCATGTAGCAAAAGTCAAAATTTCCAAATTAAATTATGAGTTTTGGATAAAATTTATTCAATTTTGAAAGTTACAATTAAATTGTCCAGAATTAAAAATTTTGGAAATACTCAGCAATGTGCCAAATAAATTTGATAATCTTCTTTGATTCCATTAGGCTGCATCTATCTTCAAATCCAAAATTGCTTTGATTTAACTACATATGATGATGGTATTTTGATTTTTGACTCTTGAGCACATAAGTATTAAAAAAATTAAATTTAATTTTCATATATTGCTAGTTTATTTGTATGTCTATTTTTACAAATTACATTGGATGTAAATTAATATATTGCGAGTTTATTTGTACATATATTTTTTTCAAATGTATTCATTTATAATTTCATTTGTTATACTATTTCATTTATGAAATAATAAATTGCTTGTGTATTATCTTCTTATAAATATCACACAATCTTTTTACTATTTCAATTTGTTATAACTAGATTAAGGAAATATGTGATATAGGTATAGCGTATAATGTACAAAACTTAGTATATCATATTAATTTGTTAATTGAATGTGAATGCAATTAAGATAAAGTTTGATTTACTTTTATATTAATATGACAATTTATAGAGTACACATAATTACAAAGTACTCATTTAGGGTTTAGCATTTTTATAATTCATTGTAAATGTAGCCCAAAATGCATAAATGGTGGAGGAAAAGGGTATACGTATCCTAGTCCCACTAGATAGGGTTGAGGCAGAGTTGAGTACACCTTTTCAACAATGTATGCTCCATCCAGGCTTACTACCTTTTAACAATTTTCGACTATTGAAAAAGCCAAATCGGACAACTTCTTGGCCATTACAATCCTAAAGTAGCACATTATTTTAGCATTGCTTTAACATAGACATTAATCCGTTGATTTGATCAAATATCTTTCCTGTCTTATTAAAATTTTCTGTTATTGAAATCCCTTTAATGTAGCCTTGCAAACATAAAATTCAACCTTTCATGGTGTGTGTTTTTACCAATTCCCTTCCATGGGGAATTTGTTTTACCTTCATCTATGATCTCTTTAATCGTGGGCCCATAACAAAATCAGTTTAAAACAGCCTTCATTACTTTGTTCTTGTTTCTATAGCTTCTAGGAAAGATATGTGTTTCCCATATTTGAAGTGGGGTTTTTCACACTAGTGGACATCTCTTACTCGTATGCTTGTTTTGGTTAATACTTTATCAGAAGAACTAAGGTATATGTTAACAAGGATGGGGAAAGAATAACAGGAGAATGGACTACATGAATAGTAAATAATTAATTCAACACTATGATGTTTCTATGTCACAGCATTGCCACATTCCACTCATTTGTGTGCATTATTGCCTATTGTATGTGAGTTGCTAGTGGAAGCCGTATACTCACAGAGTTGACTTCTACTTAAATAACATTCAACTTTCACTAATTTCTTGGAGTTCTTAGTGTTATAGATCCAAGTTGGATTGTAACACCCCTCACCCGTCTACATTATAGCCAAGCAAGGCATGCTACACGGCGTGCCGGAACACCTAGTCTGTGATTATCTCATTTCTTGAACTCAATTTGATTTTAAGAAGTCATTTATGTAATATTCATATCATGTTGATACTATTGTCATACTTTGTTAAAATCAGTGTTTCAAGACTGGTAATAAAAATTTGGCAAGGTGCCGTCTATATTTAGGACAAACTGTCCTTCCAAACCTGTTGAAAACAGTTTAATATTATGATATCAAAATCTCAAATATTTCACAGTCTCTATTTCTCAGTAAAGTCAATAAACTTTTACAGTCATTAAACAGTTCCATAATACAGTCCATAATAATTTAAATATATACAGAAAATCTCCATGTTATTTAATATGTACAAAATATTACAAACTTTAGAGCTTTCATAACATTACAAAATACAGGGCTGAATTTCAAATATACAACAAAAGTACAAAATATAAGATATCCTAAGTCCTACCATGTATGCAATGCAGTGCAGATGACTCTGGACTCCTGTGCAGATCTGATGTCTCACCCGGTCGTAGGTCTGCTGGGCTCCCCAGCTGTATCTCCAATACCTACGCGTTACAAAAGCAACGCGCTAAGCAATTTTGCTTAGTGGTGCCAAATATAAAGAAATATACACTAAAATAAAGTAAATAAAGTGTTTACAAATTTTTGGTGGTGCTGTATGTCAGTAGACTGGATTTAGGTATTTGAATTTAATAGTACTTGAATTACTGCCCGTGTAGCCTATATACTGACCAGACTGGATAAACGGATATACTGGCACTGGGTACCTAGTACCTCGGGCCGTCACACCATCGGTCACAAAGTGTCTCCCGGTGTGCAAATAGCGTGGCTAAAAAGCCATATAGTCAATCTGGCGATAAGCCAAAAATAAATACACAATATGTATAGCCGTAGGCTATTAAAGTCACAGTGCGGCATAATAAGCCGTAAAAACACAGTATGGCATAAAGCCATTTACAGAACAGCTGTCAGAATCCTATTGGCATGCCAACCTATCCATACTAGTCAACTAGGCAAACTAGGGCACATTATTAATTAATTGTTTAATTCTTCAATTTTTGGAGTTTACTAACTATTATATAATTCACAAGTCAATGCATATGTTGACCTTTTTAGGTAATATGGATAAGTTGTTTCTAGCATCAATATGTCACAATTTATATCTCAATATGCTGCCAATATAGTGCAATTTACCATTTTTACAAGTTGGCATTCATTGCCAAAATGCTTCAAGCATCATTGTATGCAAAATGTCAAATTCTCAATTTTTGTGTGCTAGATTGGTCTAGCTTAAAGTCCTATTTCCCTTGGTTTTTAGCTTCTGGTCAAAGAAGCAAAATTGTAGCTCTATGTCTTATTGCACGTGGGGCAAAATTTCAGGTCATTCCGAGTTGTGTAGACCAAGATATGGTCAATTTGCTAAAGCTGGACAGATTGCACTTTTAGTGCACAATTTGATCAATTTTTTTGGTCACTTTTCGTTCGGCAGTTTTTGTGCCCGAACTTGTGCAGGCCATTTGACTTGGTTCTGGTCATTTCTGGGCTTTGGTGTCTTCATAAGACTTGTAGATATAGGTCTTAACTATTCATGGTCAAAATTTCAGGTCAATTGGACCTGTTTTGAGTGAGTTATGGCCTAAACACTAACTTCTGCCCAAATGGTCAGTTTTCAGGCCTCAAATGCACTAATCCGGATTTGGTCACTTTTTAAGGTCAGTTTCTAGGCAGAATTTTGGCAACCTTTCTACATGAAAGTTGGCCTATTTGGTGTCTAGTTTCACCCTATATTGGCCTCATACCAATTTGGTTCATAGTTTGACACTTATGGCCTCATTTAGGTGCTGCCTTCAATACACAACCTGCACAAAGCTCATACACTTCCAATTTAATGTTCCTCCTCCTCCTCATTACTACAATATTCAATCAACAACACTTCTATACATATATTGTACACAATTTCAGCAACAATTCTGGGCAGAATGTTCAAGTGCTCAATGCACACAATCCATCATTAAGTTCAACATTCACTTCCACCAAAATTCAACATACTTCAATGTTAATATTACACATATACTTCAACATATTCATACTTCAATATCACTTACACTTGCTGCCCACAATTTAACATTAACATATATCATTAATAACCACATGTTCACACACAAATTCATGCTATTAGGGGCTGCCAAACATGGCAGTTTGCTCAAGGCATCAAGGTTCCATTTCACACCTTTAATCTAAACACTACATGCACATACTTATGCACAAACTTACTACAACTTTCATTTGAATTAATCAACCTTCATTTTCATTCACTAGAAGTAAAAATAACTCAAGCTCAACAAGGGTTCATGGCTGCCGAAAATGGACAAGTCCATTTCTCACTATTTCTTTCATTTCTCTTCACCAAAACACTCACCTCAACCTAAACACAAGCTTTACTAAAGCAAGGGAGAGGTTTTGGACACTTACAACTTTTGGAGCTTATCAAAACTTTTGAAGCTTATCAAAACTTCACCAAATCTCTTCCTTTTTGTTCTCTATAATCTGCCACCATCGGTCACAAAGTGTCTCCCGGTGTGCAAATAACGTGGCTAAAAAGNNNNNNNNNNNNNNNNNNNNNNNNNNNNNNNNNNNNNNNNNNNNNNNNNNNNNNNNNNNNNNNNNNNNNNNNNNNNNNNNNNNNNNNNNNNNNNNNNNNNNNNNNNNNNNNNNNNNNNNNNNNNNNNNNNNNNNNNNNNNNNNNNNNNNNNNNNNNNNNNNNNNNNNNNNNNNNNNNNNNNNNNNNNNNNNNNNNNNNNNNNNNNNNNNNNNNNNNNNNNNNNNNNNNNNNNNNNNNNNNNNNNNNNNNNNNNNNNNNNNNNNNNNNNNNNNNNNNNNNNNNNNNNNNNNGGGGAGGGATTTTGGACACTTACCACTTTTGGAGCTTGTTAAAACTTCACCAAATCTCTTCCTTTTTGTTCTCTATAATCTGCCCAAGAGGTGTGGACCAAGTTTAATGAAAGAATTTAAGTGGAAACATGGCGTGAAGGTGCTTGCATGAGAGATTAGGAAGCTTTGGCCATGGTGTTTTCATGGTGGTCCATTTCGGCTGGAGAAGATGATGCAGAAAACTGAAGTGTTTAGTGGACATACACATGTCCTTTAGGTGTTTAAATACCCCTTAGTGCTCCACTCACTTTTAATTAAGTAATTTTATATGTTAAGATTTTAATTATTCACTTTATACCCCATTTCTTGCACTATTAATATAATGTACCACAATTTTAATTTTTAAGACATTTATAAAGCGTAATATCATTTATTTTTAATGGACATTGAGGTCAAAAGGCAATTCTAGGTGTCAAATGACCAAAATACCTCACTTCGGGTTATATTCCCGATTTTTTCGGTAATACCGATTTTTGTCTGTTTTTCGATTTTTCGTTTTTCTTTGTACTAATTTATTAATTTTTCTTTAATATTTCTAGTTTCAATTACATTTCAATAAATCTTTATTTATGTCTCAAAATTAAATTCCGAGGGTTTCCTGCGGTCCTGGGGTCGGCAACGGCCTTCCCGGTGCGGTCACCCATCGCTGTGGTGCCGGCTCGTTTAACTTAGTTTCGTTTTCTCTCTTTCATTTTTGTTTGATTTTTCTTGTATTTTCTTTTTATTTATTTCATCATTATATGTCTGTTCACCATCACCGAAGTGTAGTTCCAGACATCCTGACTTGCCTGGACTGTTATTCGACTACTGGAGCAACAGAACGTACAGAACACGTGCAATGGGGATGTTACATGGATGGCCTTTCATGTAAGTGGACATCAATGCTGAATAAGAAGCGAGAAAAATGAGTGTAATGAGCTCCTAGAGCTAATTAAAGGATGTGGCCTAGAAAGGCCATGAAGATACCAATGAACAAATGACTTCGACAAGTTTTTAAGTCAGCAGAAGAGAGTTTTGGTTTTCAAGTTTTTGGTGATTTTCCAATATTATCTATTTTCCAATATTTCTGAATAATTTTGGGAAGAAAAGAAAAAACCGCAGGGCGGGCGTCGGTGGGTGGGTGGTGGGGTGGGTGGGGTTTGTGTTGCAGGGTGGAGGAATGTGGGATAATTTCGGAGTAGATTTGGCTTATGTTCAACTCAAAGTGCGACCCTGATGATGCAAATTTTAGTTATGTTATATGGTAGTGAATGTAGGGCATTGAAGGAGTCGTATGCGTCTAAGATAAAAGTTGCAGAGATGAGAATGTTAAGGTGGATGAGTGGCCATACTAGACTAGATAAAGTCCGTAATGAGAGTATTAGAGAAAAAGTAGGAGTGGTACCAATTGAGGATAAGTTGAGATAAGGGAGATTGAGGTGGTTTGGTCATGTAAAGCGTAGACATACGGAGGTTCCAGTTAGACAAGTAGAGCACATTAGGTTAGAGGGTAGAAAGAAAAAAAGGGGTAGACCTAAATTGACTTGGAGGAGAGTAGTACAACATGACCTAGAAACATTACAAATTTCTGTGGATTTAACCCAAAATCGTTTAGAGTGGAGAAAGCGAATCCATATAGCCGACCCCAAATTTTTGGGATAAAAGCTTAGTTAAGCTGAGTTGTGATTGAATTTAAACATGGACAATTACTAGATACGGACAATTACTAGATAATGTTGTGTGTATAGTTCTTGCTACTTTGCCATTGTTATCAGTATTTTTAGGATTCTCATCATGCAATAATATTGTACATTTCATACATTGATGTATCGTATTACAAATTATTATGGCAAAAGAATGTGAAGAAGTTTTATATTACCTCTGTTGACAAATAAGGGTTAAAAAAAAAATATTTAACAG**GTTCAGTGTCTTTCTTGATGGGGAAAACAAACTCAGAAAATGTGAGAGGAAAGCCCGGCAAGCCCACAACAGATGATTTAATACAGGTTTTAGAGAATGTTCGTCTTGGCTACATATTATCACAATTTGGTAGTCTGGATTCAACATATGAGTGGTCCAGTGTTCTTTCCCTTGGAGAGCAGCAACGCCTTGCTTTTGCGCGACTATTGCTTTCAAAACCAAAGCTGGTTCTATTAGATGAATCTACCAGTGCTTTAGATGAAGTCAATGAG**GTACTTACGGTTTCCAGCTCTTCATTTCTCTTATAATGCAAATTAGTAAAACCAATGTGCCAACACTGGAAAACACTAGACATTTTGGATTGTTAGCTCAAAAAGAATTCATTGTTATGTAATAAAAACTCTTTGCAGCAAACCCCAATCTGAATACACCCATTATACAAAGACAATATTTTTAATCAGTTCCTAAATACAGGAAAAGAGTATACAAGTTAGATGCTTCTCTCAGATTGTCATATGTGAACTTTTAGACAATGAATGAGCCATATCCTATGTGGTTCATGGAAGGTGCGGCGGAAAACAAGAAAACAAGAAGCAAGTGGATTTAAGCAAGTGGATTCTAGTCTATATCAAGTTGCTTGTCCTGCAATCATGGATCCAAAGCAGTTGCTCATAAGTTGATTTGGAGATATAAGATTAGAGTCGTTAACAAACTTTAGGAACCCAAAGTAACTCATTTTAAAACAACAGGATTGAATGCAGATATTATGCAAAACAGAAGGGGTTCATAGTAATATACCCATATATGATGCGATTATCACAGGCTACTTGCTTAATTTCAGGTGCTAGGGCTGAGTCTTCGATCTTAATTTTGTTTATGGTTGTGTTTTATTATTGCAG**GCACATCTGTACCGGCAAATTGAAGCAGCAGGCATAACCTATGTTAGCGTTGGCCACAGACGTACTCTATATAAACATCACAACATGGTCTTACGCATATCCACAGCAGATCTCAACCGTAACAAACGGAACTGGGACATTGAGTCCATAAATCCTGGAGCCTTGTATAATTTGTCTAGTCAGTAACTAGTGTGCATGGTAGCTGCATTAGTATCTATGCCATGGTTTATATAAAATTGAAACCTGTAATTTCCTCCAAATAGAAGGGCACAAATGCAAAGGAGCAGTAATATTT**

>HbABCE2 scaffold2554(16420-26277)

**CTCTTTGATCTTATACAGTTATACTACATCGTTTTATGTGAAACTAAAACTGTCGTTCAATCAAAACAGGGTCTCTCTGCTCCATTTCACAGCTCCTCCTACAAACCCTAAGGCCTTCACTTGCCACAGCCGCACCGATTTGAATCCGCCACGACCATTGTTGTTGGCCTTTGCCGCTCTCCATAACTGCCGTGGTAGCTGTCGTTCACCTCTTCGAAGCCATCGCCGTGGCCGTTGCAATTGAACACAAGGAGGAGGCAAGTAGCCCTATATAGCTGTCTACCCCTCTGCCTAATTGTCTTATCCACATCGCCGGCGCTGTTGTGTGGTTGAG**GTGAGCTTTGCAATTAAAACTTTGTATTTTTTTCCCATGGCTGTTCCTTTCATTTATTTATGGATGCTGGGATCAAAATTGAAGAAAATTGAAGCCATAGATTGTGATGCTATTTGTTGTTTGAATCTCTCATAAAATTCATGATTTCTTCTTTAGATTTTCTGATTAGCTCATTAGATTTTCTTATTAGCTTTTTGTTAGATATTTCTTTCAAAACTTTGTGCTTGGAGGTAGTTGGCCAGCTTATAAATGAGCCGGATTTTATTGAGCTGAGCTCCGATTAGTTCATCCTATTTGGAGACTATTATTAACAATAATCATTCCCTTCAACATGTTATTTTTTATTTTAATTTTAGATGGTTGTGTGTGCATGAATATATTCGGCAG**GTAAAAGAAAGAAAGGAGAGGGAGAGATGGCAGACAGGTTGACGCGTATAGCAATTGTGAGCTCTGATAGATGCAAGCCCAAGAAGTGCCGTCAAGAATGCAAAAAGAGTTGCCCTGTTGTCAAAACTG**GTAACTTTTTTTTTTAATGATTTTATTCTTACATTTTGAATTTAAATATCAATGATCTATTTCTTTTTTTTTTTTTTTTTTTTTTTTTTTTTTTTTTAATCTTTTCATTTTGCTAGGCTTAGTCAATCAGAGTCTGTCCTATTATCTCTTGTTCATTTTTTAAAATCATTATGCTAGATCTTTTCACCATAATTGTTGTGTTTACTGCTCAATGCAATACTAGAATACTTTAGGCCCTTTGGTTGATGATTAGCAATGCCAATTCTTGGTTTTGCGTTCAGAAAAGAATTGAACATAAATTCACTAAAGCTCCTTTGCAGTAGGAGTGTTCTGCACCTGATTCTTTCCTTATATAGGTAACTTGTTTGTTTTGGTTTAAAAGTGTCTCTTTACTTCTACTACAGAGTTAGATGGTAGCATCCTAATTTCTATTTTGACAACTAGCCTTGTACTCAGTTCAACTAAACTTTTAGGTAACTAGGTTTGGGCTGGTATATTCGTTATAAGTTTAAATGAGATAGAGTTTTCTGGAGTGGGGAAGACTGTAAGAATCAGAATGATTTGGGTTAGGGTGGATCTCACCTTGATAACCTTATCCATTGTGCCCCTGTTTGGTATATGTTGGAGCTCAAAGGTAGAACTGATTGTCAACTTTCTATTGGTTTGAATTGTCTTTGGTAATGGTCTTTGCTATAGATTAGTAATCCATATATCTTTCTTCTTACCCTTTTGTACGATTTATCCGCAG**GGAAATTGTGTATTGAGGTTACTCCTGCATCTAAGATAGCATTCATCTCTGAGGAGTTGTGCATTGGATGTGGCATTTGTGTTAAG**GTTGGTAAATTCAAGTAGGGAAGGGATCCTTCTGTGTTTGTTTTCAGTTTGCTTATGGCTTCGTGCAAAACTGACTATTAATAGATCTGTTAATGCAG**AAATGCCCATTTGAAGCAATTCAGATCATCAACTTGCCAAAGGATTTGGATAAAGATACAACTCACCGTTATGGTCCCAACACATTTAAATTGCACAG**GTTTGTTAATGATTGCATGGTTACTGGGTTAATAATTGAAAGAAGATGTTCATCTTTTTAGCCACATTTAGAGTTGGAGATGCACACATAAGTCGAAGGCAGCCAGGAAATATCATTGATCTTTCCTGGATGATAATTGCTTGTGGGTTTGGAAGGCATAATTATATACTTCCGCACATACAAAGATAGAATTACTCTTTTGATTCCTTTATAATTTTCCTGTTAGTTTGGAGGTATGAAATAATAGGGAAGAAAAAAAGGACCCTCTCTTATAATTATCTTGGATTAGCTAGCTGCAAGGGCCATGGTTTTAAATCGCGGTCGCGGCCACATTCGTGAGTAATGGAAACAAGCCTGTGACGGCATGGTAATGGCTTGGCGCTACACAAATTTTTTTGAAAAATTTGCAAAGTTTATGAAATGAGTAGATATTGGTAGATACAAGTAAAATTAAGATATACAATTATATAATAGGCATAAATACATAAAATTAGATGTTATTTTGTAATTAATAATGTATTTTGGTAAAATTAATTCTAAAAAATAGTTCAAATTAGAAAGTATGATTGGAGTAACGATCGTTACCATTACTTAACGGTAAATAACGGCTGTTACCATTACGTAACAGTGGTAATGGCCGTTACCCATGCAAATCAAGATATACCTTTAAATAACAGTCTAACGGGTAACGTAATAGTCCCTTTAAAAACCTGTAAATAACGGCTGTTATGTTACATAATATGACCGTTATTTAAAACCACGGCAAGGACCCATTTGTCTTGGTGTAGTGTAGTGTCTTTTAAAGCCTTTCAGTGTCTTGCAAACAATGGCCTTGTTCTTGTATTGGACATCAAAAAGTGATGCTTATACCCTTTGACGTATCAGAAAATTACTATTGCAG**GTTACCCGTCCCTAGGCCTGGGCAAGTTCTTGGATTGGTTGGAACAAATGGCATTGGGAAGTCCACTGCCCTCAAAGTTTTGGCTGGCAAGCTGAAGCCAAATTTGGGCCGTTTCAAT**GTACTACCTTTGTGTATAATTCCTATTATTGCTACTTTTTTTTTTTTTTTTTTTTTTTTTTTTTTTTTTTTTTTTCTATTTCAG**AATCCTCCAGATTGGCAGGAAATCTTGACCTACTTTCGAGGATCGGAATTGCAGAATTATTTTACCCGTATCCTGGAAGATAATTTGAAG**GTACTTTCCCGTTTTGACATGGTCTTAAACTTGTATTAACTAATAACTACTTATGAAAGAATTAAGTTTCTGTGTCATAAGTTCTTGCATGTTTCTCTTCCATTCAACCTTCTGGTTATCTTTTCAAATTGTTCAATGGCTATAGTAAATTTTGACCATAATTGTGTAACTATACTGTGCAGATTTGAGTGTGGAAATGTTGAATGTTTAGTGTCATGACCAAAAATTGATTTGCATGCATTATCTGTGATGCATAAATGAGACAATTATTCAGTCAATAAAAAAGGATGATCTAGTGCACGAAGCATCACACTTGTGGGCTCCGAGGAGGGTCGATGTATGTAACCTTACCCCTACTTTGCAGAAAAGCTGTTTCCGCGGCTCAAATCTGGAACCTCCAGGTTACAAATGGAGTAACTTTACCATTACACCAAACTCACCCAATGATGATCACTTTTCTTTTTTTTTTTAATTCCTTATCTACATACAATTGTTTATTTGAATGCAGTCATTTCACACTGGGCATAAGATAAGTCCTTTTATTTGTTCAATTGTATGCCTTTATTGGTATTATATGTTCGGGGCTCTGGTCCATGAATTACTTTGCTTTGTGATCTATGAGTATCACTTGCTGTAGTCCTTTTTAATTTCAGTTCCCATTGAAGATAATCTTGAATGGGACAATTGTTAACACTATATTTGGGAAGGGATGAAATTGGGGGAGAGGAAGAGGGGAAGGAGAATATGAGAACTTGTTTGGAAGAGAGCGAAAAATGGATGAGGAAAAGTTTTAGTCTTTAAATACTCGTGTGGGGTCCACAAAACTTTTTACCCCAAATTTGGGTGAAATTGAAGAGAAATTAAACATAACTACTAAATCATCCTCTCTTTAGATGTTATTTGTTAAAAGAAAGGGATGTTATTGTGATTTGGCTCTAAAAAGAATTCTTTTCTTTCTTCTCCTCTATAGCAACGAGTAGTGTTATTAAGGCGAGAGCCGAGAGGTGACATTAAGGTGATAGGGTACCCTATGGCCTTAAGGTGAGAGGCGAGGGGAGGTGAGAGCGTTTTTGAAGTGAGGTGCAACACACACACACACACACACACACACACGCACAGAGAGACACTTTTAATATAAAAATATCATACTTTTAACTAGTGTAATAAGTAAAAAAACATATTGAAAAAAAATATATTATTTGCACATGTAAAACAAGAGAAATAAAAAGCAAAAAACAATAAGGTTATACAAAGGAAATGAAATTTAAAGAAAGGTTAATTAACTCCATTTTATTAGGGCTTAGGGTTTAGGGAGAAATAGGAGAGAAGAGAAGAGGAAGGGAGGAGTGAAGAGGAGAGGGAGAGGAGGGAAACCCTAAAAATAAAATAAAAAATTATTTTGAAAGAGGTGCACCTCGCCTCGCCTAGCCTAGTGCCTTAGTGCCTAGGCGAGAGAGGCGCTCACCTTGCCTGGCTCCTTTAATAACATTGGCTGTGAGCATTTGCTTGACTCGGTTTGAAATTATCAAAACCGAATAAACTGAAATTTCTTAGAAGGGAAACCAAACTGAACCTAAAATTGAAATATTTTGGCTTAGTTTGGTTTTTATTACATAATATAATTTCAGTTGGTTCGATTGATTTGTTTTTTTTAATTGTACAAACATAACCAAATGGAATTAGCTGAAATAATTGAAAAAGAAAAATAAAAAAGAACAACAATAACAACAACAACAACTAAGCTTTAATGGTCGGCTACTGAACTGAAAAACCAAATAGCATAAGTTTGGTTCAATTTGTTGGTTTAAGCCAAATAATGCTCACCCCTACTTCTCATCCAAACAAGAGAGAAACTCATTCTGTCTTATCCCATTTTCTATCTCCCTTCAGTTTCTTTCCCCTCATATTTTCCCTAGCAAAAAATAGTTCCTTCTGAATCTAATTCATAGCTTCTAATGTAAGTAATCTTACATGTCAAGTTGGATTTTTTACCTCATGTTTTTTTTTTTTTCCTCCTGTTCCTGAAGTGATGTGATGCATTATCTATTTGGATATGATTGAAGTATTATTTCCGTTCATTGTAATTTTTATTCTCATCTTCAACTTTTACCCTGATATCTTTATTCCTTACCGTTTTCTTCTTTTTAAAAAAAAATGTGAGTTTGCATAAGAAGACCTTTTGTTTTGGCTTGCTATGCTAAAGTCTGCGTTCCTTATGCTCCCTTTTTGTAGTGTATTTTTCTGCCTACCAAAGTAGGATAAAAAGGAGAAATGCTAAACTATGTAATCTTGTGAACAATGCTTGATTCATTTAAATTCATCTATTTCTTTCTTGCTAAAATACAAGTTCATATTCAACAG**GCCATCATAAAACCTCAGTATGTTGATCACATTCCTAAAGCAGTCCAAGGCAATGTAGGGCAGGTGCTTGACCAAAAAGATGAGAGAGAGATGAAAGCAGAACTTTGTGATGATCTTGAGCTGAACCAGGTTATAGATCGTAATGTGGGGGATTTGTCGGGTGGAGAGCTACAGCGTTTTGCTATTGCTGTCGTTGCAATACAGAATGCAGAGATATATATGTTTGATGAACCTTCAAGTTATCTTGATGTCAAACAGAGGCTTAAAGCTGCCCAAGTTATCAGATCTTTGCTCAGACCTAATAG**GTAACTTCTTTCTTTTGTTCTTGAAAATTTTTTTCATTCTTGTACTTTGAACTTAATTGCATTGGCTTCATCTTTGTTTCAG**CTATGTAATTGTAGTGGAGCATGACCTTAGTGTCCTCGATTATTTATCAGACTTCATTTGCTGCTTATACGGGAAACCTGGTGCATATGGAGTTGTAACCCTTCCCTTCTCTGTTAGGGAAGGAATTAATATATTTTTGGCTGGATTTGTTCCTACAGAAAACCTTCGATTCCGGGATGAGTCGTTAACCTTCAAG**GTAATTTTGCTATATTTTTGTATCTACACACAGATGCACACTTTGCCTCCCTTCCTCCTTGACGCAATATTAGTTGCCTTTTCTAG**GTTGCTGAGACTCCACAGGAGAGTGCTGAGGAGATTGAAACATATGCACGATACAAATATCCAACCATGACTAAAACTCAGGGCAACTTCAAGCTTCGTGTGATTGAGGGTGAATTTACAGATTCTCAGATAATTGTGATGTTGGGCGAGAATGGGACCGGAAAGACGACATTTATTCGTATGCTG**GTACTTGTTTCTAAACATAATTTTTTTTTGTAAAACATTCTTTTTACCATGAAATCTGGCATTTGTTGAACAATTTATTTTCTGGGCCAAATATGCAG**GCTGGCTTATTAAAACCTGATAGTGTCGAAGATTCCGATGTGGAGATACCTGAGTTTAATGTTTCTTACAAGCCCCAGAAGATCAGTCCTAAGTTTCAATCTACTGTTAGACACTTGCTACATCAAAAAATTCGTGATTCTTATACACATCCTCAGTTTGTGTCAGATGTCATGAAACCGCTTCTTATAGAACAACTAATGGATCAAGAAGTAGTGAATCTGTCTGGTGGGGAGTTGCAAAGAGTTGCTTTGTGCCTCTGCCTTGGGAAG**GTGAACACTTTTAACTTATTTTCGTAATTCAATAATTCATTTTGGTGAAATGAAAATACAACTGACCAGTTGGTGATGTTTTAATTCAATTTACTGTATTCTAGAATATGTCTTTTTTGTGGTTGATTCTTTTGGTCTTGAGTGAGTGGGCTCATGGATGGAACAGGTTTTGCAAAAATTATTGAAGAATTACATGAAGGGTGGCTTGAGGGTTTCGTGTGAGGTTGATGAGTTCAGTTATATAAAATTGCTTGGAATGCTTTACTTTGTTGAGATGGACTTTTAGTTTTATGTAGGGTGATGTAAAGTATTTCTTGATTTGTACATGGATTTTCTACTTGATCAAAAAATTGTGAAATCCTCTCTTCAAATTATGGAAGGGATGCATGGGAGCATTGTTCAAACTTTTTTTTTTTTTGTACCCATTTGGATTTCTATGCTATGCCAGCTTCTAGTCATATGAAGCATGGATACTTAGCTGTAGGGGGCTTGTTTAGTGTGTGACACTGTGCTAGACGTGGAATATGTTTGTTTGACAGTGTTAGGACATGGATAAATTTTCTGAGGCCTATAGGATTTGTTTTCTATTTTTGGTCCTTTTGTCGTTAGTGTTGATTATATTTAGTTACAATTGCATGATTATATTTAGTTACAATTGCATGAGTTTTATCACTTTCGACTATTTGCGCAGTTTTCAAGTGTTTAATTGTGATCATCAATTACTGCCAGTTATGTAAGAGTGTTATATTTAAGCGGTCTCCATAGCACACCTTAGTAAGTGCCTTTCACCAGTTGCTCTCCATTGGTGAATCTGCATCGCCATATACCATGTAAAATCATTGTTGAAGTACAAAAAGTTCCTCCCTTCCAAAGCCCCTTTTAGTTAGATGCATTGTTCTATTTGGAAACATTATTCATGCCTAAAGATCTCTCTAGAGTTGTTTTAATCTATATATGCCACGCTTCAGAATAGCAGAAAAGGATTTAAGTGAGTAGGGAGACTGGATAAAGTGTTCTTTATAATTGTAATCTAAATTTTTTAAGATTCCCAACTTTCTTAATTATTTCCTTTCTGCAAATCCAATCACTCTAATGCTCACCCTTGTTATCACTTTATTGCTCACACCTTTGGAATCACTTTATTGCTGACACCTTTGGATTTGAAGGCAACACCCAAAGGTTACTAAGTGAAAGGGAACTTGCCAAGTTTACAAGGTGGAATCAATATTTTTGTATTGTTGTCCACCTTGATTATTCTTATCAGGCCAAATTAATGTTCAACCCCAAAACCACTTGAAAGCGTATAAGAATGCCTCTTAAATGGAACAATTATTCCTCTTTTTCAACATAAATGGAATGGTATTATTGGCACCTTACATGGGAAATAATCTAGTATCCAATACCAATGCTGTGCTAACTTGGAATCCACTCATGTGATCATGTCTGTCTCTCCTGAACTCTGTAGTGTAATTAAGCTAGGCACCTTCATATGATGAAGTTTAATGTCCAACTCTGCTTGAAGCACCAGAAAAACCACAAGGAGATTTCATTAAGCAAAACCTAAAATTTTACTGTATCCATCCAAGAATCCCCATCAAATGCTTTTGTGCCTGAACCTCATTTTCTTATTGCTCAAAACGAAAGGATTGGCTGGATCCTTATCATTCATGTTTTAAGTTTTTATTGGTTCAAGGCAGCGGGATTCAGTGCAAATAGATTATTTATTTATTTTAAAAATGCATGGATCCTTGTGTTTATGCTGCGAGATGAAAATTTCTTTGTTGAACAGATCTTGTTAATGTCCATACCTGTGTGACTTCAGGAATTTTATGTTGACATTATATCCATCAAAATGCATGATTGTGGTTTTCTTTGCCCTAACCTGAGCCCTAGAGTAACCTTAGAAGACCTTCCCAAGTTTGAAAGCTTCCTAAAACCCACAAGAGGCAAGCCTGAGGGAACACTCAAGACCTAAGACAGGAAACCTTGTCCTCATTTGACTATATGGGGGGAAGACTGCAGGTCTATACCTGTGTGACTCCAGGAATGCATGTTTAAACATGCGGTGAAGACCCTAAGCTGCAAACAGTCCCTGAAACTTCTTTCGGCAGTTTTACTTTGCTCCTTTCCTGTTTCTGTAGTTTGTAATTGCTTGTACTTGTGATTCTTGATTTATGCAGTGAATGTCAGTTGTTTGAATATAAAATGGTAGCTATAGCTGATCGGCGTGTGAATTGAAACTAGTCTTGTCTATAACATTGTTCTTATTCACTTGGTGTGTGTGGTTCAGTTCTTTTTGTGGGTTCATAAAAAATCAGTGACATGAGCATGTTTGCAGCATATCCTGTAATTGTGACTAGAAGCTAGCCGATGGATCGTCTTACTTTCACTAATTTTTAATATTAATATTGAAAGATTTGAGGCTTCTTCTATTTAATGGGGTCTGATGTTTGGTTTACTGCACGTGATGCAG**CCTGCAGATATTTATCTAATCGATGAGCCAAGTGCTTATCTAGATTCTGAGCAGCGAATTGTTGCTTCAAAAGTCATAAAGAGGTTTATCCTTCACGCGAAGAAAACAGCATTCGTGGTGGAGCATGATTTTATAATGGCAACTTACCTTGCTGATAGAGTTATTGTATATGAGGGGAGGCCATCTGTGGATTGTGCAGCAAATTCGCCTCAGTCATTGTTGACTGGAATGAATCTCTTCTTATCA**GTAAGTACTCATTGTTCATGTTTTAAAGATAGTTAAGAAAGATGAAGTAATGAACGCAAGAAAATTCCAACTGAATTTTTCTATTTTCATTAGTCTAGTTTCTGACATAAGCTTTTGAAAATTTCACCTTGCATGGAGGCTCTCTGCTTCTTTTTGAACCATGTACTAGTTTTGCTTATTGTGAGCTTCACTAAGGCTCATCTCAATAGCTCACTTTTGCACAATTAACATTTGTCATAATTGTACGATTTAAATTTTACTCCTGGTATTTGTGGTCTCTTTAGAATGACAGTTTGATTTTGCTTCACTTGATGCAG**CATCTAGATATCACTTTTAGGCGGGACCCAACCAATTATCGACCAAGAATCAATAAATTGGACTCTACCAAGGATAGAGAGCAGAAGGCGGCTGGGTCATATTATTACCTGGATGATTGATGTAATTATTGGCAGGTTTACATAGTTTTCTTCATGGCCAAACA**

>HbABCF1 scaffold0291(635004-639836)

**CCCTAATCGTCGGGTTTTGCCCTCAAACCAATTCATCAGCAAGAGAAACACTAGATCCACAGATCTGTCCTCTCCTTTTCTATCTAAG**GTTTGTCTTCTGACTTAATTTTGAATATGGATTTTTCTCTGCATTTTAGTAGCTAATTTTTGGTTTTTAAATGTTTCAAG**GATGGTTTCCGACGCCAGCAAAAAGAAGGCCGCACAGAAGAAGGCGGCGGCGGCGGCTAAGAGGGGCGGAAAGGCAGCTGCCGCAGCTGCATCGTCCAAAGCTACTGCTGCTGCTGCTGCTGCTTCTTCAGCTGATAATGGAAGTGTTGACAATTTGTCGAATGGGGTTGGGGCTATTCAGATATCGGATCGGACTTGTACTGGCGTCCTATGTTCTCATCCTCTTTCCAGAGATATTCGC**GTAAGTTGATTGCCATCGATCTCTCTGTATTTATTACAAATGGTCTTGTGGAAATGTTAGGTTTTATTTATTTGTTTAGGTTTTTAGGTGATTTTTATAGAATTTTTTTCGATTTGACATGTTTTTTTTTTCACCTTTTCATTTGATTTTTACAGTTTTTGGTTAATCTTTTGTTATAACAGTGTCTTATTAAATACTGAAAGAAGATTAAACTTTTTTAGTTACGGAGATGGTTCTTTTTTTTTTTTTTCCCAAATTATAGTTGTTGGAATCAAGTGCTGATAAGATTATACGTTGGATGTGAAAATCATTTACAGGAATGGATGAAACTCTGCAAATAAAAAGGGGTAATTAGCTGGCGTAGTGTTCAGTTCAGGACGGGGATTTCCTGTTTCTCTAATGCTAGTCCTAAAATAGGATGCTTCTGTACTGGATACATGTTTCAGATGGTCAATGATAAATTAAATAAAATGTTGTGAGAACTTATTCTCATAAATCGATGTCTTCCTACATTCATATCATAGGTATATACCAGAAAAAAAATAATGTCAGCATGATATATTAGGTTTTTAAAATAGTATGAATAATGTTGGCATAATGAGTCTCATTACTAGCTTATTGTTGCTAGCTTGGTGTACCTAGACAAGTGGCTAGGCGAGCAGCTGAGTCTGTCACCAGGGCATTCTGACAACTCTTGATGCCACCTATAATTACTAGAAATTTTCATTGTGAATATAAGCAATTGTTCATCCTTGTGGGCACTGCTTAGAGGAGGCAATATTGAGTGATAAGTATTTTTGTACTTTTATATGGAGTTGAATTGTTGCTCTGAAAATGATGTTTTGGTGATTAACTAACCTATCTGCTAATTGCAG**ATTGAGTCTTTATCAGTTACTTTCCATGGACATGATCTCATTGTCGATTCTTTGCTTGAGCTTAACTATGGCAG**GTTTGTTATCCTACCTTCTCACTCAACTGTATACCATTCCTTATGGAACAATTGCTTGATGGATGTTTTGTGTGTTAATATTTCAG**ACGATATGGATTGCTGGGATTAAATGGATGTGGGAAATCAACACTCTTAACTGCAATAGGTTGCCGTGAGCTTCCAATTCCAGAACACATGGATATATATCACCTCACCAGAGAAATTGAAGCTTCTGACATGTCTGCTCTTCAGGCTGTCATTAGCTGTGATGAGGAGAGGGTGAAATTGGAGAAGGAAGCTGAAATCTTAGGTACACAG**GTATGTCAATGTACCCAATAACTTGGGTGTTTCAGATCTTAGGGGCTGTTCAGTTAGGCAAAAAGTAGGTTGAAATTGGAATTAGAATGAGCTATACCATAAAATGGGTGGGAATCAAAATGTGATTATCCCAATGGGAAATGGGAGTTGAACTTGCGAAAAATTGGTGTGTGGCTTTTAAAATTTATAAATAAAATATATAGAATTAGAAGTATCAAAATAAATAAATACAAATTAATAATTTAAAAATTTTTTATCAACATGAGCTTGCCACTTGAAATAAATAAAATAACAAAAATAATTGAAAATTTTAGAAATAAAATTATTTAATTAAATAAATAATAATTCAATATAAAATATATATTCAATGTGTGTGTGTCTGTGTGTGTATTCAATGAATTTTATATTATGCTAGTGTTAGTGTGTGTGTTAGCGTGTGCATGTGTGTGTGTGTGTGTGTGTGTGTGTGTGTGTTAGCATGCATGTGTGTATGCATGTGTGTGATTTTGAAAAATATGTATTCAATCTAAAATATAAATGATATAGCAAATTTTATTAATTATTTATGTTTATATTTTCTATTTGAATTTTTAGGAAAATTTTGTATTGATTTCGATTCAAGCATTAACAGCAACCAAACAACCTTTTAAGTAATTGAATTTCTGGTCGTTGTTTAACCAAACACTGAAGTGGGAAAAACCCATTCCTTTTTCCAACTCCGCTCCATTCCATTTCTGATTTTCACATTTCAACTAAACGGGCCTTTCGTTGCTGAGTATTGTAATGACTTCTGAATGAATTGTATTTGTCTTGGCTTTAG**GAGGATGGAGGTGGAGAGACTCTTGAACGTATATATGAACGTTTGGAGGCTATAGATGCATCTACAGCTGAAAAACGTGCTGCTGAGATATTGTATGGTCTGGGTTTCAACAAGCAGATGCAGTCAAAGAAAACCCGGGATTTTTCTGGTGGCTGGAGGATGAGGATTGCATTAGCTCGGGCTCTGTTCATGAACCCTACAATCCTTTTGCTTGATGAACCTACCAATCATCTTG**GTAAGCTTGTTGGGTGCCTGTGTTGCTTGGGATGTTAAAACATTTCCTGGCTGTTGCATGCTTTTTACATGTTTGTCTAGGTTTGAAATTTTTATTCCCAGATAAGGTTGCTCTACTAATTTATGACATCTGTTATTTTTATATATGAGCATGTGGGCCAGAATCTTTATGGCTTTTTAATTATTTTCTTTTTTTTTTCCTTAAACACCTTTAATGTATAACATTCTGGTACTAGCGGGGATAAACATGTTAATGATGGTTTATCTGTTGAAATATTACAATTGTACAATTCTGAGGTGTTATAAACATTTTTAGAATGCCTTGAGTTGTTGGTTATTGACATGAAAAGTTTATCTGCTTTTCAGAGTTGCATTTTATTTTGCCTGAAATAAAATATGGGAAACTATGGCATACTGTTTTTGTTTCCCTTGGGATGGATATTTAATTGTTGGTACCGCATCGATGTGCATTTAACCCTTGAAATTTCATTTACCATCTGAAGGGAAGTTTTAAGTTGTCATATTAACAATTAATTTTTTTTTTTCCATGATGACAGATTTAGAAGCCTGTGTCTGGTTGGAGGAAACTTTGAAGAGATTTGATCGCATTCTAGTTGTGGTGTCTCACTCCCAGGATTTCTTGAATGGTGTAAAGGGAAGTTTTAAGTTGTCATATTAACAATTAATTTTTTTTTCCATGATGACAG**ATTTAGAAGCCTGTGTCTGGTTGGAGGAAACTTTGAAGAGATTTGATCGCATTCTAGTTGTGGTGTCTCACTCCCAGGATTTCTTGAATGGTGTCTGCACAAATATTATACACATGCAAAACAAGAAATTGAAGATCTACACTGGGAACTATGATCAATATGTTCAGACACGTGCTGAACTGGAGGAGAATCAGATGAAACAGTACAAGTGGGAGCAGGAGCAGATTGCTTCAATGAAGGAGTACATTGCCAGATTTGGGCATGGTTCAGCAAAATTAGCCCGTCAAGCACAGAGCAAGGAGAAAACCCTTGCAAAAATGGAGCGGGGTGGGCTTACTGAGAAGGTTGTCAGAGACAAGGTTCTGGTCTTTCGTTTTGTTGATGTTGGGAAGCTGCCACCTCCTGTGCTTCAGTTTGTGGAAGTTACATTTGGCTATACTCCTGATAATCTCATCTACAAGAACCTTGACTTTGGAGTAGATCTGGACTCTAGGATTGCTCTAGTTGGGCCCAATGGTGCTGGGAAGAGCACACTATTGAAGCTTATGACTGGAGATTTGGTCCCTACTGATGGCATGGTTCGGCGGCACAATCACTTGAGGATTGCACAATTTCATCAGCATTTGGCTGAGAAACTTGACTTAGATATGTCCGCCTTACAATTTATGATAAAAGAATATCCAGGGAACGAGGAAGAAAGAATGAGAGCAGCAATTGGGAAATTTGGGTTGACTGGCAAAGCCCAGGTGATGCCAATGAAGAATTTGTCAGATGGGCAGAGGAGCAGAGTGATTTTTGCTTGGTTAGCTTATAGGCAGCCCCACTTGCTGCTGTTGGATGAGCCAACAAATCATTTGGATATTGAGACTATCGACTCTCTGGCCGAGGCATTAAATGAATGGGATGGTGGTCTGGTTCTCGTTAGCCATGATTTCAGGCTTATAAACCAGGTGGCAGAGGAGATATGGGTGTGTGAAAATCAAGCTGTCACGCGATGGGAGGGCGATATCATGGACTTCAAGGAGCACCTCAAGAAGAAGGCCGGCTTATCTGATTGAGTCAGATGATATGATGGATGTGATGATTATTATCCTATTATCAGCGTACCTTTATCTATGGTTGAGAATCACAGCACACGGCTACTTACACTTGTGAATCACAACACAGGCAGGTGATTCCTGTGGCTGGAAGATGCTTACCTAGGGTTGAATTATAATGAAAGCCAAGAGCAGAGATGGGTGACAGAGGCTTCGTTATTGTTTTGAGCTGAAAAGCACACTGTACTCTGCTCATTTACTAGGTGATTATTTTTGTTTTCATTATTCTTATTTAGTATACTTGGTTATGCACCAAGGAGATTGATCAACAAAATTTGCGGAGGTTTAGTTCATTACTAATATGAACCTCATTATAGCT**

>HbABCF3 scaffold1143(154673-162701)

**GAGCTCTTCCTGTATTTGTAACCTTACCTGCTTTCCGTCTTTGCAAAACCTAAGTACCATTTTCTTCTTTGATTCGAGTATTCGGCCATGACTGAAGTGGCCAGCTCAGCGGTGCACGAGGTGCTCGGCCGCCGAGTCCAGGATGTGGATCAGCCAATCATCGATTACATCATCAACGTCCTCGCCGATGATGATTTCGATTTCGGCGAGGAAGGTGAGGGTGCCTTTGAAGCTATAGGTGAACTCCTCGTTGGTGCCGGCTGCGTCTCCGACTTCTCTGAATGCCGTTTG**GTAAAGAGCTTTCAATTATTGCTTGCTTGCGTTTTGTTTGGTTTTGCTACTCAATTTCCTTTTATTCTAGAAGCAGCGATTTCAGCTACTGTACGATTGATTTCATTAGTCTTTTAATTGGCGGATAGCCGCCAGGTTATGTAGAGAAAGCTAAGATATTCCAAATTGTGTACTTCGTGTTGGTTAGCTTATTAGAGATCTAGCTGCTGTGTCTTTGGCCTTAGCTAAGCTAAATAACGAAGTATTATCTCTTTCTTTATTTGGTGGCTGAGAAGATGTGGAGAAATGAAAACCGTTGGGACTGGGGAGTAAAACTTTAGATAGTAATAGGAATTCCGTGCTTCATTTGCTTCTTATTGTAGGAATTGGGTATCAAACCTTTATTAAGCTATACAGTTGTCGATAAGCACATTTCAATATAGGCCTTTGTTTTTTTTTTTTATAGATGTGGATACACAAATTTAATTTATGTGGTGACTGCCCGGTTTATTCTTCATCTCCCCTTTTCATCGTTTTTGGAATTTATGATAG**GTTTGTTGTAAGCTATCTGAAAAATTTGGAAAGCATGGGTTAGTAAAAGCAAAACCAACTGTACGGAGCCTTACGACGCCCCTAAGAATGAATGATGGAATGGATGAGGAGGTTCCAGTGAAGAAGCCTGAGGTTATGGATGGTCCTGTTCTGTCTGAGCGTGACCGTGCAAAGCTAGAGAGGAGAAAGAGAAAGGAGGAGCGGCAAAGAGAG**GTAAAAATTGGGCCTTATCCTGTAATTGTGCTACTTCAATAGCGAGCAAGTCAGTGGCACATGACCATGCTCATCTTTTATTTTAGTATTTTTAAGTGCTGCTTCCAAACTTATGCTTCACATAATTTGGTTTTGTTTTATTTTAACTTTTGGGAACTTGGTTTTAAATCGCGGTCGCAGTCGCAGTCGCAGGTAACGGAAATGGGCCTATAACGGCTGTAACGTAACGATAACTGCTTGGCGCTACGCAAATTTTTTTGAAAAATTTACAAAATTCATAAAATGAGTAGATATTGTTAGAAATAAATAAAATTAAGGTATACAATTATATACTAAGCATTTATATATCAAATAAAGACGTTATTATCTATCATATTTGGACGTCATTAACTAATTTAGACCTGAAATAATTATATAAATTGTAAATAACTAACTTTATTACCACCAAAATGAATGTTTAGGAGGTTCTTGATAGTCTTGACTTTGGTTCTGACTTTGATCCTCACTTTGTTGATATTGACTCAATTTATAATATTGTGATGGATTGAAACTAAATAAGGAATCAACTGGATTAACGGGTTGTGGAAAGTATGATTGCTCTGAATATATAGGAGGGGGGTAATTATATGGATATGGATTTGAGTGTGCTTGTCCATGTCCATATCCATAATCACTAGTAGACGTAGATGATGAGCCTCCATGTATTATATTTCCACCATGACTATACCCATAGCTAGATTCAGAATCAAAAGAACACTCTCTATGTTGAACACCTTTGCCCTTTGATGACGTTAATTCAGCACCCCCAAAACCCCATGATCTACGATGTCGATATGTCTATGGCCATTCTGGAAATGGAGCTTCGCCACCAGGCACTCCACCACCTTCGCCACCACCTCCACCACCTTCTTGTAATTGTGAAGTTTGAACTAAATTATCATTCTGAATTATTCTCTGCTATTTTGGACTGAAATTAGGTGGTTTTTGAATTGTTTTGGACTGATAAAATCATGTGATTTCTGTCCCCCACCAGTCCACAAATTCTGCCACATTGGTAACGGCCGTTACCGTTATTTAAAGGCCGTTACCATAATGGTAACAACCATTATTCTGCAAATTAAAATAGGCCCGTAAATAACAGCCTAACGGGTAACGGCCTCCTTTAAAACCTGTAAATAACGTTACGTTATTTGATTCCATGTTTGGGAATTTCTACTGATTATGTATAATATATGATCTTGATTCTTGAATGTTTACTTTGGTTTTCACAATTTGTTTCCATTTAATTTTTTCTAATTCAACAGGCACAATATCAAATGCATCTAGCAATTCTACTGATTATGTATAATATATGATCTTGATTCTTGAATGTTTACTTTGGTTTTCACAATTTGTTTCCATTTAATTTTTTCTAATTCAACAG**GCACAATATCAAATGCATCTAGCAGAAATGGAAGCAGTTAGAGCTGGCATGCCAGTTGTATGTGTGAATCATGATATTGGTAGTGGACCAACCGTCAAGGATATTCATATGGAGAACTTCAGTATCTCAGTGGGTGGCCGTGATCTCATTGTTGATGGTTCAGTCACACTTTCTTTTGGAAGGCATTATG**GTTTGTTTTCTTGCATTTCCCACATAAAATATCTTGCATTAGTGTCAAGACATTAAAGAAAATAAGAAGGAATATAATATCTGTTAAATAGGGTTTCTCATAATTGTTATTTAGTTGATAATATTTTATACTTTCTTGTATTTTGCTTGCAG**GCCTTGTTGGAAGAAATGGTACTGGGAAAACAACTTTCCTCAGGCACATGGCTATGCATGCTATTGATGGTATTCCTGCAAACTGCCAAATCTTACATGTTGAGCAAGAAGTGGTTGGTGATGATACATCAGCCTTGCAATGTGTTCTGAACACTGATATTGAGAGAACCCAGCTTTTGCAAGAAGAAGCTCGTCTACTTGCACAACAG**GTGACATTGTATCTTTTGTTGCGGAGGAATCTATGGTCACAATTTATTTATTTGTTGTGCTTGATGTGATCAGTCCTTTTAATCTTGCTGACATATTGATGATTTCCTGAAG**AGAGAATTGGAATTTGAGGGAGAAAATGGAGATCTCAAAGGGGATCACAATGGGGCAATTGACAAAGATGGTATTGCACCAAGGCTTGAGGAAATATACAAAAGGCTTGAGTTTATTGACGCATACTCTGCAGAAGCACGTGCTGCTTCCATTCTTGCG**GTCAGTTCTCTGCTCTTTTTTTTTTTTTTTTTTTTTTTTTTTTTTTTTTTTTTTTTTTCTGGCGGTTGTGGTGCTCAGTTGACTTAGACCTCTAACTAATACTGGAATGCTTGGATCTAAAAGAATAGTAGAAACAATAGTCTAAAAAAGACTTTGGATAGCTGATAATGAAAATAAGGGAAATTTTAGGATTATCTGATTTTTTTTTTTTTAAGTGACATTATGGATCATTTGGCAAAAAAAGATACTACTGTACAAAAAGCCTGTAATTGTCTGGTTTGAACAGTTAACCTCATCTTTATATATTCTCACCATTTCTTGAATATTTGCAG**GGCCTCAGTTTCTCTCCAGAAATGCAGAAAAAGGCAACCAAAACTTTTTCTGGAGGATGGCGAATGAGAATAGCTCTTGCTCGTGCCCTTTTTATAGAGCCTGATTTATTGCTACTCGATGAACCTACA**GTGCGGCATATATTTATGTGCTATTTGAGATATTTGTTCTATTTATAATGACTTGGTTTATAATTTTGTTATCTTTTTTCTGCAG**AACCATCTTGATCTTCATGCTGTCCTATGGCTGGAATCTTACCTGATGAAGTGGCCAAAAACATTTATAGTTGTTTCTCATGCTAGAGAATTCTTGAACACG**GTAACATTCATACTGCTTATTTAGTATTAATGGATTGTCATGATAACCTTTGCCTTCTCTTTCTATAACTTTGGTTTGGTTTCTCAG**GTCGTCACAGACATTCTCCATCTACATGCGCAAAAGTTGAGTGCTTACAAAGGTAATTATGATACATTTGAGAAGACACGGGAGGAACAAATTAAGAACCAACAGAAAGCATTTGAAGCAAATGAACGATCAAGAGCCCATATGCAG**GTCTTTGTGTCAAGCCAGCAAAAGAAGCAATATATTTCATTTGTTGTTTATGGTTTCACTTTTCTTTTCTGTGTGCCTTTTCATGTTACTTTGTATTTGTATGGCAATAAAGTATTGAGGAATAAAAGAAGTTTGACAGCATTTGTTGTTGAACGCATGTAATCATATTTACTTTCATCAAGTGCATTTTCCATAAATTGTTTCTGCATAAAGCTTTACATGATGCTTTCTCCCTTTTCTCCATTTTCCATACCTGTATGTGGGCATTCATTAGGTCCATTCACTGACTCCTCAGTTATCAGGTCCTAAAAACTAAGAGTGCTGTGAATGTCAGACTCATGATATCATATCTACATTATACTGATAATCTACTGTCTTATGATCTCCCCGACATAACCTTCGCTTTTTTTTTTTTGTTGCTTTCCCTATACTTAATGCTCTTTTGAAATTGGCATTCAATATTATTTCAGGATTTCTGAATTATAAATCTTGTGTTTAACAAGCAAATGTTATTTTTTCTTCCTGCAG**TCCTTTATTGACAAATTTCGTTATAATGCAAAGAGGGCATCTCTTGTCCAGTCACGAATCAAG**GTGCTTAATGTATTATGGCAGATGATCTTAAATTGAATATTGTAAACCTTTTACTGCTTCATTTGCTTGATATACCTGTGTTCATTCTTTCCCTGGACTTAAACAG**GCATTGGAGCGAATGGGTCATGTAGATGAAATTGTTAATGACCCAGA**GTATGTATTGCTCTCCTTTATCACTCTGGTCAACTCATACGGGTTTTAGATTTGACATAATTTTTCTTTTTATTTACCCTCTCTCTACTTAGTTGTTTTTTTTTTTTCGTTGCACAG**CTACAAATTTGAGTTTCCTACTCCAGATGATAGACCAGGTCCCCCTATAATAAGTTTCAG**GTTAGATTTGCAACTTGTGGAATTTTTATGGTAAAGATGGAGTGCGGTGCCTTGCTTACAATTTGTTTTTTTATCACTGCAAACTTGAAATCCTATGCTTCATGTTTCATTTATATAAAGTAATGGCCTTAGTAAATTATTTCAGTTGATTCAAAATTTAGAAATGTTACTTTTATTTCTACTTACTAGTACTGTGTTTGACTAGGATCAAGAGCTCACATTTACTGTGATTGTGATTACAG**TGATGCATCATTTGGTTATCCAGGGGGGCCCATGTTATTTAAGAACTTGAATTTTGGGATAGATCTGGACAGCCGCATTGCAA**GTAAGCATGTTTTTCAAGATAGAATATTCCTTTTTCCAGCCTGTTCAGTGTTCATATATGTATTAATGTCTACCATATAAGAATTAGAGCAATCTACCATATAAGAATTTGGGCAATTAATCTATTAACTTATATTAGGCATATTTGCAATAATCTGACAGGTTCTTTTCCTCATTTATGTAG**TGGTTGGGCCAAATGGCATTGGGAAATCAACGATCCTTAAATTAATAGCTGGAGAACTACAACCAAGCTCTGGCACCATCTTTCGTTCTGCTAAG**GTGTCTTGCTTTTTTCTTATGATTCCACTTTTTGCATACCAATTTATAATTAATTATAAAACCTCCATCATACATCTATAAGTGTTTGTTGTTCTACCAGATAACGTCTCAACTGTAAAATTTGTCTCTTTCCAAGGGGGGAAATTGAAAACAGAAAAGCAGCCTGTTTACACTGTTTGGTGCTGGTCGAATGGAGATTTTCAAAATTTTTCTTTGTTTAACCATTTTCTCTATTAAAATTGTGTTATGCTAGTTATTGTGCTATTGATGACTTAAAGTCTGCTTTCTATATATGAATCTGATGGATACTTGTATTGTCCTGTTTGAAG**GTTCGAATTGCCGTGTTTAGTCAGCACCATGTTGATGGACTTGACCTGTCTTCCAATCCTCTTTTATATATGATGCGCTGCTTCCCA**GTGAGCTCTTGCTTTGACCAATTACATTTTTATTATGATTTTCCTCTGAACATTTATTTCCTAAATTTTTCTTTCAATCCATGGTTTAATGATGATATTGAGAGGAGTTCTTTGAGCTTTTGATAGAATTATAGTATTCATGTATTTGGACGTCTTTTTTGTTTGTTTTCTGCTTTGCAAGCCATTTTATTTGAAGATTCCTTCTTGTAG**GGGGTGCCTGAACAAAAGCTTCGTGCTCACTTGGGTTCTTTTGGGGTAACTGGAAATCTGGCACTTCAACCAATGTACACACTATCTG**GTACATTTATCATCTCATTGTCCTTCATTATTTATATTGTAGACTGAAGATCAAATTTATCCTCTTGAATTTTGAAGAAGCTTTCCTTATTGGCTGTGTGATTTGAGTTAAAGGCTTACCATGATGAATATGCAAATCACATGCTATTTGAGTGCTATGATAGAGCCAATCTCAGTTATGACTTCTAATAGCTCTTGCATGAAGCTTTTCTCAGATACACTGTTTGTATGTGTTTGTAATTGCTCTATACATTAG**GTGGTCAGAAAAGCAGGGTTGCATTTGCAAAGATAACTTTCAGGAAACCACATATAATATTGTTGGATGAGCCATCCAACCATCTT**GTAAGTCATTTTCTCCTCTTCCTTTCTCAATTTTTTTTTTTTTTCACTAGATGTTGTTGGGATTTTGGAACCTTTGCAATGAGCTTTTGCAGGGGAAAGACTGCTAAGGTTCCCAAATTTGCTGTTCTGAAATTATTTAGTTTTCATTTTATTATTTGATGAGCATTTTTTTTTTTTTAATTTGATGGGATGAATCTCAAGGAAGAGCACGTTTTATTTAATGCAAATTGCATTATTCTGCGACTTTCAATAATTATACTAGAGAAAAAAGGTTATGAAACATGGCATATAATCATGCTTGGAGAACATTTGAGCGTGAGCTTTACCAAATTATATATTTGTTTAGTGAAGCTTTCTTTTGATAGAGAGTTAGAAATTTTGCATATGGTTTTTGTAGGGATAAATACAACTGTTAACATATTGGCTTGCGCATTGCAAATGCTTATTTAGCAAAATCCCACATTGTCGATGTTAAACCTCATAGAGCAGACATATTCCTGTGTTTTTAGTTGATACAATTGCAGCCTTTGAAGCAACCTTTTTCTGGAATGCTGCATCAATACGACATTGATAACATTTTCTACTGCTTTTGACATGCTTATACATGTTAAAATGTAG**GATTTGGACGCAGTGGAGGCACTAATCCAAGGCCTTGTCTTGTTCCAAGGAGGGATTCTCATG**GTATTGTGCCTGTTGTCCTGATGGTTTTCATTTTCTGATTCAGTTTCTCAAAATAAATGATCAAATCAAGCAATTGTTATCATTCTTGCTTGCAG**GTTAGTCACGATGAGCATCTAATATCTGGAAGCGTAGAGGAGCTATGGGTGGTTTCACAAGGAAGAGTGACACCTTTCCATGGAACTTTCCAGGATTACAAGAAGATACTCCAGTCATCTTAAAACACATTTTGTTGACTTCATAGCTATTCCTGCTGCAGTTGGCAAGGAGCAACATAGAGATTGATTGCTGCTGCAAATTACATAAGATGATACAAGTACCTGCAACGGAACGGTTTTGAATTTGTGTTTATCGTCATAATTGGGAGTGGGTTAATTTTTTCATGCGAGAATGAGCTTCAATAGAGTCATTTTGCAAATGTTTTTAGCAACACAATATGATTGTGGTTTTTCTTTTTAAAAAAAAGAAAACAAAAAAGAAAAAAACTAAATTTCAACTCAAAAGGTTTCTTAAGCTTGCTTTTTACTGCAGTATATCGCAAATGATTAGAAATTTAATACCATCTCCAAATCAGAAATGTCTGGAAGATTCGGTCGGTGTGGAAGCAGA**

>HbABCF4 scaffold0583(88229-90611)

**CAGAGTAACTTCTACAATGGGAAAAAAGAAAACAGAAGAGACTGGTCTAGCCACTAGATCAAAGGCAAGCAGCAAAGATGGAAAGAAAGAGAAAATATCTGTTGCGGCCTTGCTTGCCAACATGGACCAGAAACCTGATAAACCTAAAAGGGGATCAACATCTTCCTTGAGCACTGCCAAGGCAAGGGCTCCAAAAGTTTCATCTTATACTGATGGGATTGATCTCCCTCCTGATGAAGAGGATGATTATGCCTCTGAGGATGAGCAACAGCATGCTGGGGCCAAAAGGCAATCGAGCAGGCAGCAAAGGGGTGAACCTAAGCTACTTGATATCTCTGTAACAGACAAAGAACTTAAGAAGCGAGAAAAGAAGGAATTACTTGCTGCTTATACTGCAGAGCAGGCCAAAAAAGAGGCCCTTAAGGATGACCATGATGCTTTCACTGTTGTTATTGGTAGCCGAGCTTCAGTACTCGAAGGTGAAGATGAAGCAGATGCTAATGTCAAAGATATAACAATAGATAATTTTTCCGTGTCAGCACGGGGGAAAGAACTCCTAAAGAATGCATCAGTGAAGATATCTCACGGAAAGAGATATGGTTTGGTTGGGCCCAATGGAATGGGCAAGTCCACCTTGTTAAAGCTTATTGCTTGGAGAAAGATTCCTGTACCTAAAAACATAGATGTGCTTCTGGTTGAACAAGAGGTGGTTGGTGACGATAAAACTGCTCTAGAAGCAGTTGTTGCAGCTAATGAAGAGCTTCTCAAGATTCGACAAGAAGTTGCTTCTTTGCAAAATGCAACGTCTGCTACTGGTAATGAGGATGGAGATGATGATATAAATGGAAATGATGCAGGAGAGAAGCTTGCTGAATTGTATGAGAAATTGCAGATCTTGGGGTCAGATGCTGCTGAGGCTCAGGCATCAAAGATTCTTGCTGGGTTGGGTGTCACCAGGGAAATGCAGGGCCGTCCTACTCAGTCATTTAGCGGTGGCTGGAGGATGAGAATTTCATTAGCAAGGGCACTTTTTGTGCAGCCAACTCTATTATTGTTGGATGAACCCACTAACCACCTTGACCTGAGAGCTGTTCTCTGGTTGGAGGAGTACCTATGCAGGTGGAAGAAAACTCTTGTTGTTGTTTCTCATGATAGGGATTTCCTTAACACAGTGTGCAATGAAATTATCCATCTTCATGACTTGAAGCTTCATGTCTACCGAGGGAATTTTGATGATTTTGAAAGTGGATATGAGCAGCGTCGCAAAGAGATGAACAAGAAATTTGAGATTTATGATAAGCAGGTGAAAGCTGCGAAGAGATCAGGAAACCGTGTACAGCAGGAAAAGGTTAAAGATCGTGCAAAGTTTGCTGCTGCCAAGGAAGCATCCAAGAGCAAGGCAAAGGGCAAAGTGGATGAAGATGAACCCTTGTCAGAGGCTCCAAAAAAGTGGAAAGATTACAGTGTGGAGTTCCACTTCCCTGAACCTACAGAGCTGACACCACCACTTTTGCAGCTGATTGAAGTCAGCTTCAGTTATCCCAACCGGGAGGATTTCAGGCTCTCAAATGTTGATGTGGGCATTGATATGGGTACCCGTGTTGCCATTGTTGGGCCCAATGGGGCTGGAAAATCTACTCTACTTAATCTTCTTGCTGGCGATTTGGTTGCAACTGAGGGTGAAGTAAGAAGAAGTCAGAAGTTGCGGATTGGGAGGTATTCACAACACTTCGTGGACCTATTGACAATGGATGAAACACCTGTTCAGTATCTTCTTCGACTTCATCCTGAACAAGAGGGACTTAGTAAGCAGGAGGCCGTTCGTGCCAAGCTTGGGAAATTTGGGCTCCCTAGTCATAATCACCTCACTCCAATTGCAAAACTATCAGGAGGGCAGAAAGCTCGGGTTGTATTCACATTGATTTCCATGTCTAGGCCGCACATATTATTGTTGGATGAGCCAACAAATCATTTAGACATGCAGAGTATTGATGCATTGGCTGATGCACTGGATGAATTTACTGGCGGAGTTGTCTTGGTTAGTCATGATTCCAGGCTTGTATCTTGTGTTTGTGAGGATGAAGAGAGAAGTGAAATTTGGGTAGTAGAAAATGGAACCGTGAATGCTTACCCTGGCACATTTGAGGAGTACAAGGAAGAGCTACAGAGGGAAATTAAAGCAGAGGTGGATGACTAAATGATTAAGTGCTTATTGTTCCAATTGCATAAAATAGTTAGCCAAGCTGTTATTATCTTGTATTTTCATGTAAGTGCATCAAATCAGGTACTGTTTTCCCCTTTCCTCGCTATCGATGTTCAGTGTATAATCTAATATGTTGCTGTATGTCAGAAACAAACAATAATTAATTTATCAATTTTGCC**

>HbABCF5 scaffold2098(6066-11421)

**GGTTGAGAAGTTCTTCTCTGAGTTCACTCTGTTCAATTCGCAGAGTTTCCCTGCGAGGATCTCAGCCCCTTTACCCAATTTCCCATTTGCCAAACATGGACTTATCCATCAAGTTCCACCGTCTAGACCTCCACTCTTCTTTCGTCACCGGTTCGCATCTGTTTGGTGCCAGTAAATCTCTTCGACTTCCCCGTTTTAGGCATAGCTCAAAACCCATTAAAAACGATCACAGTTCGCTCAAAATTGCGGCTCCTTTTGTATGCAGAAGAGGGAATTCAAAAATCACTGCTCAGTTATCCACCGCCACCGTCGAAACATCGGTTGCCGAGCCCGAGACCGATATTGAGTCCTTGTTTTCGAGCAGTTCTTCTGATGAATTCGGCAGGAAAGGTGCACATAAGCATTCGCATACTGGGGCTTCGGGTATTTCATCGGGTATCAAGCTCGAAAACATAAGCAAGAGCTACAAAGGAGTCACCGTGTTGAAAGATGTGACTTGGGAAGTGAAAAAAGGTGAGAAAGTAGGACTGGTAGGTGTGAATGGGGCGGGCAAAACGACCCAGTTGAGAATTATTACCGGACAGGAAGAACCTGATTCTGGGAACGTAATCAAAGCGAAATCCAACATGAAAATCGCGTTCTTGAGTCAAGAATTCGAGGTTTCTTTGAGTAGGACTGTTAAGGAGGAGTTCATGAGTGCGTTTGAAGAAGAAATGGAGATTGCTGGGAGGCTGGAGAAGGTGCAAAAGGCGATAGAGGGAGCTGTGGAGGACTTGGAGCTGATGGGGAGATTATTGGATGAGTTTGATTTGTTGCAGAGGAGAGCACAGGCTGTGGACTTGGATGAAGTTGATGCCAAGATCAGTAAGTTGATGCCCGAACTTGGGTTTGCCCCTGAGGATTCGGATAGGTTGGTTGCCTCCTTCAGTAGTGGGTGGCAGATGAGGATGTCGCTTGGGAAGATTTTGCTGCAG**GTATATTACAATCTTTATTTTTTTGATGATAACTTTAGATGCTGATTTTTGAATGAAAATGCAAGAAATATGTTTGCATTTTTATGTTGCGGAATTTTTGTTTTTTAATTGGGTCTAGATTTATGCTATTCCACCTCAATTATTATTGATGTTTAATATGAATACTTTGATTTTGGAAGAATTGTTAGCTTTGTGGTGAAGCAGAGTGATAACTGAGTGGCTAAAGAAAGGATGGCTCTTGGTTGTTTATTACTTGATGGTTGACTTAAATTTTCAACTATTCATTTGCAG**GACCCGGATTTGTTGCTTTTGGATGAACCCACAAATCACCTTGACCTTGACACAATTGAGTGGCTTGAAGGTTATCTCCAGAAGCAAGACGTGCCGATGGTCATCATATCTCATGACAGAGCTTTTCTTGATCAACTTTGTACAAAAATTGTGGAGACTGAAATGGGTGTGGCTAGGACGTATGAGGGAAATTATTCTCAGTTCCTTGTGTCCAAGGCAGCATGGATTGAAGCTCAGTATGCAGCCTGGGAGAAGCAGCAAAAGGAAATTGAGCAAACCAGGGGCTTAATAAGCAGGTTGGGTGCAGGAGCAAATTCTGGCCGTGCTTCTTCTGCTGAGAAG**GTAACAAATAGATTTATGGTTTCATTTTGTTGAAGAACAGGAAATATTTTCGTCTTGGATATTATTCAAACGTATAATTGATATTTAGTGAGCTAGCATATAGAAAGATCAGTTTATTCATTGATGTTTCAGAATGTTTCCTACTTTGTTACATTGAATCTGGTGGATTTTTTGAAGCTACTTGAACAATGTTATTATGATAATAATATTGTAGTATATTTGTGAAATAGTATATCTTCTATAGGTTTTGCAGCATTAATCTTAATGAAATTTGCAACAAATCCTTTGCTGAACCTTAAATTTACAACCTTCAGGATACCAAAAATGAGTCCAGAAATTCAAGTATGATGAGGGATGAGGCTTCAGTTGTGGCTGTTGCTTTTGTACTTGTAGAATATTTTTCTAAACAAATATAATTATGCTTGCAAATATCCTTGCATACATACATAAAATGGGGGATTCTTTAGTTGCTTTTGGTTCTCCCCTCCCCCTCGCCCCCTCCCCTCCCTCTCCCTCTCCCTCTCCCTCTCCCAGTTACTTTAGCCTTCTAGTTGGTGGGCGTATTGTCAATTTTTCGATTCAAATCCTTTGGTCTGTCTGACTATCTCCATAAGTAGGCCCTATTATTACTTTTTTCTATTGAAAATCATTTGACAGAAGTATGAATGAGTTTTAACTAAGTTAATGAAATTTAATACTGAATGCTTTCTCTATCTTTCTTTTGGCCTTGTCAACTATAGCTATTTAATATTGAGAATGATTCTTTCATCTTCATGATGTGAAAACTGCATTTTTTTCCCTAGCTGCTTCAGTTCTTAACCTTGTTTTTAAATTTATTTTTTATAATTTAACAG**AAACTGGAGAGACTTCGAGAAGAGGATCAAATAGAGAAGCCATTTCAACGCAAACAAATGAAGATCTGCTTCCCAGAGCGTGGAAGAAGTGGAAGAACTGTTGTAATGATTAAGAATTTGGAATTCAGTTATGAGGATAAG**GTGAGGTGCATCGCAGGAGAGGCTGCATTGAAGTTTGAATGTTGAGTTTTCGAATGATCTTCTACACAATATTTACATTTTTGGTGTGCTTTTCGTACCAG**GTGCTGTTTAATAGGACTAATCTTACAATAGAAAGAGGTGAGAAAATTGCCATTATTGGCCCAAATGGATGTGGGAAAAGCACTTTACTGAAGCTGATAATGGGCTTAGTGAAGCAGAAAGCAGGTGAAATTGTACTTGGGGAGCATAATGTACTACCCAACTATTTTGAGCAGAACCAG**GTGTGCTACTTTCATCAATCAGATACCATTTAATTTAAACTTTTTCACTTTTTATTGTCAATTTCCATTGTTATGCATATAAACTGTTTTGACCAACTGAGTTTGATCTTCCTATTTAAG**GCGGAGGCACTTGATTTAGATAAAACGGTGCTTCAAACTGTGGAAGAAGTTGCAGAGGACTGGAGAACTGATGATATAAAGGGACTGCTTGGGCGTTGTAATTTCAAAGCTGATATGCTTGATAGAAAGGTTTCCCTTTTGAGTGGTGGTGAGAAG**GTAAACTCAACTGGCCTCTCAATCTCATATCTCTATGTTCAGTTGCATAAGAAATTCTGGTTTACAATGAATAAATTTGTTTTGCTTTTCGAATCATTTATTGAAGCTTTCATCTATGCTCTTCTGGATCTTTTTGTAGCTTGTAACAATGGATTAGTTGATACAGTATTGGAAGATGATCAAACTTAGGAATTAGTCATTGTAGAATTATGAAGTTTAAGAATTTTATTAGGTTTAGAATTTATTATATAAGAATTCTATTAGGTTTAGTATTTTATTTAGGAAGTTTGAGTTTGTTTAGTTTTCCTAATTTATTTTGGTTTAGTATCCCTAATTAGTTTCAATTAGTAGTAGTCGATACACTATAAATACCTATGTGTAGGTATTTTATTATTAGTTTTGATTATTATGATATTATTAGCAATTGAGAATTAATATGAGAATTTGTTTCTCTTGATCCTGTTGTTCTCTTTGTTCTACACCTCTGATACAACTTAAATTAACCGATCCGGCAGAACAACTCAAAAGATAAAAAAGTATCATTAATTTCTTCATGCTCGTTCCAAGTTCGAAGTACCATTTGTACAAATAAGAATCCCCTTCGTGGGATCTATGGGGCAATTAATTAGAAATAAATTTTGTGCAACAGCCCGTGGACAGTTTTTTGAAACTTAAGGACCAGTATATGATGATTTTGCAGTCAGTGATAGCTGATATCACAAAAGGAACCAAAATATTATCTTCAATTCCTATCTAGTTTGAATTTTTTTTCTTGAATCCTTGTCAATCAGGTGGACAATTGAGTTTCTTAATATTATCTACTATCACTGATTCTTCTCACTATATGCTGCTGAGCTTCAATGATTAGCCTAAAAAATAATGAAGTTTTGGATAATAGTAATGCATGATCAAATTCAAGAACTAATACTACTACAAGATGAAGGTAGGTTATAGTTCCTCTTTCTGATGTATCTTTAATAATCTAGGCTCAAAGCATGAGGTTATCGATTATATGCTTTGAAACCTGAGTAGCTTAATGGCCACCTTGTTGCTATAGGTTGAATAGGTTCAATTCTCTATGATTTTTTCCTATTATTGTTTTCCCCCTTTTAACAAGGCATGGACTTTGAATGTTGCCTTCCTGCTGTATTCAG**GCACGTCTAGCATTTTGCAAATTCATGGTGAAACCTTCAACTTTGCTGGTTCTGGATGAACCAACTAATCATTTGGATATACCTTCAAAAGAGATGCTTGAG**GTTTGGATTGTTTGTTAATTGCTTTTACTCATTTTACATTTTATTTATTTTAAATGCTGTGGCCAATCTGTGTCCGATTGTCACTTCAG**GAGGCCATAACAGAATATAAGGGTACAATCATCACAGTTTCTCATGACAGATACTTCATAAAGCAAATAGTTAATAGAGTAATTGAAGTTAAAGATGGCCAGCTACAAGATTATGCAGGCGATTACAAT**GTAAACTTGCCCTTCTTTCATTTTGTTTTATTAATTCTGCTGCCTCTCTTATTATATGATGCATCCCCTGCCACTTTATTCATCTTGAGCACTCAAAGTTTTCATATCCATAACAGAAGGATATGTGTCTTTGGAGGTCTGATATCGCATCTTTGTCTGGAGCATTAGTTTAGTGATGAATCAAGAAATAATAAATGAAAAATTTTATTTGTCCTGCATGGAATGCATGACTAGGAGTGTGAGTTTCTAGTCTTAGGTTGCTTGTGGTTTTTGACCCTAGATTTCTCAATTCACAG**TATTATCTGGAGAAAAACCTTGATGCAAGGGCAAGAGAACTTGAACGTGAGGCAGAGCTCGAGGAAAGAGCTCCCAAAGTGAAGGCCAAATCAAAGATGTCAAAG**GTAAGTGTTATCCATTTTATTGCTTAGCCTTATCATTTGATTTTTTTTTTTTTTTTTTTAATGAAGTGCATTATTTTATTCTTTGATAG**GCTGAGAAGGAAGCTCGGAAGAAACAAAAAATGCAGGCCTTTCAGGCTGCAAAACAAAAGTCTAAAGGACTGAAGAATTCCAAGAGATGGAAATGAAGAGGGACATACACTATAAATTGATTTAGTTTCACACATGATTGAACTAATTTTACCATGTAAAGAGCCTAGGAGGTGATGATGTCCATTTTCACCAACGTACTCGACCATTGTAAATAATATTTGATAGAAATGC**

>HbABCG3 scaffold1413(27548-35627)

**TCTTAACTCCCATAATTAAAGCTCCCTCTCTCCGCTGGTTTCACTCTTTTCACACTAATTCGTCCTGCCAGCTCTTCACCCAGATCCAG**GTTTGTCTCTTTACACTCTTCTCTTTTATCTTTCTGTTAGTTTATCTTGTTCTCTATTTATTGGAGTGGATTTTTCATTGTTGTAGTAGCTGTTATTGGATTCTATTTGGTCACTGATGTAAAGGAGAGGAAAGGTGGAGTTGGGTGCTTTGTGTTTGTTTGTTTTGGAAAAGCAAAGGGGAAACTATGCTTAAATGAGCTAAAGAGTAGTGTTGAAGCTGAAACCGTTAATAAAGTTCTAATCTTTTGAATTTTTTCCTTCATTTTCTTCGCAACTAAACAGCAGGTCACCAGGTATTTGGTTTTTTAGCAAGTTGAGTTAATGTTATTTTAGTTCTGAAAAGGAGAGCAAGTGGGCTTAACTTGTTTTCTCTTGTTACGTGATCTTGGATTAGTTGGATCAAAGGGAAGTAGCGAAAGCAGGTGTTTAGTGGTGGTTTGCTAGTTTAGTTGAGGCTGGGGTTTTAGCCCTTTTGGGTTTCTGCCCTTCTGTTTATTTCACTTTTGTTCTAGTTGTGTTCTATGGAAGTTAAGGCATAGAAAAGTCGGTTGCGCCCTTACTGTCTGTTTGGTTGCTGAGAAAATGAGATAACTTAAGGGGAAGAAAACTTGAATGCAATGGTGCTATATTTTTTAGGTTTCCCTGATTTTATCTTGTTGATTTTTGGGGTTAACAGAATTTAGTATTGGTATTTATTTTTATATCTCGGGTTTACTTTTTTATTAAGAGCTGCCAATCCAAATGGGTTGTCATTTGACTGATTCCGTTTACAGTATGACTATGGTCTATATTTAACTGATTTGCACATTATATGTTTTGATATTGCAATTTTATTAAGTAACAATTTTACATTTCTTTTTGATGGTTTCTTTTCTGTTAGTATACAATGTATAATGGTACAGGAATCAAAGTGTATGCTTTATTATTTGGGAATTGTATTCCTTTGTTTTCATTATCCTTGACTTGTTATGCAG**GAGATGCTTTTTCATTGATGATGATAACTGACAGTCACTTTTTAGTTGGAATGTGTGAATAATAGTGCTGAAGACTTTTAAGTTGGAAAAGCAGGTTAAGCATCCCTTCAGTTGTGCAATGTTGCTTTTGACAGTTTGAGGTCTCAAAAAGTTGAGAGTAGAAGATGGAGGAGATACAGTCTCAATCAGATAATTATAGATCTTCTTCGTCTTCAGCCAGTAGTCCTGCAAGCAGGGTGCCTTCAAGTAACTTTTTTTACTTGCGGAAGCCTGGTTCACTTAGACAACCAATCTCATTTGAGGATTCACCAGAATGGGAGGATACAGATGTCGATGTTAGGGTGGAGGAAGGAGGTGACACTATCAATGTTGCTGCAACACCAGCGTCCCCATGTCTATCAAAGCTTAATAGTGGGTCATTGCCATCACCGCCATTACCAGAGAATGCAGTTGTTGCGAGAAAGATTGCGGGATCTTCCGTTGTCTGGAAAGACTTGACTGTTACAATCAAGGGTAAAAGGAAGTACTCTGATAAAGTTGTAAAGAGCTCAAGTGGTTATGCACTGCCAGGGACGATGACAGTAATCATGGGTCCTGCAAAGTCAGGGAAATCTACATTGTTGCGGGCTATTGCAGGTTTATTAGCTTGCTATTCATTGTGATCAGCTGCTGAACATTGAATATAATGATTTTAAATATCTTTTTGGGTGTTTGGAATTGAACAAGCATGTCTCTGAATTTCAGGAAGATTACATCATTCAGCCAAAATGTATGGTGAAGTATTTGTGAACGGCACAAAATCACACTTGCCTTATGGCTCATAT**GTAAGTTCTAAGTCTTTCAAGAGCATTTTTTTTTTCAAATCTGGATTTCCTTTATTGATGCACTCTTGAATCTTGATGTTCTTCCTCCTATGATTTTTTTTTCTTCGTACAAGAAGAAAGATTCTCACTTGCTTTAGAACATTTGGTGTACTACATCCATGTTCTCAAGGAGCTGAGATCAGTTTGTTTCTTACATGCCAAAAGCTGGTCCAGTTATTATGAGAATAAGCTTTTTCAACTCTAATCCTGTGCATTTACATAGCTTTGACTAACTAGTCGCTTAGTAATCTATATAAAAAAAAAAAAAGAAAAGAAAAGAAAGAAAGAAAAAAAATATAAATAAAAAGGAAGATCAGTCTAGATTCTGCAATATCAACAGTGCTTAAATTTTGACTGTTATTTCCATGATAGTTGACATGCATTCTTGATTATGTGTTTGATATAAGTTTAGACATGTGTCTAGCTTGAAAAGAGCAATGGTCCTTGACATTTATTGGATGCTGTTCCTGTTTCAATTCTCAAATCAAAATGTGATGAGGCCTCTGGCTGTAATCATTTCCTTTAATCTTTATTATCTCATCCTGTTGACATCTCTTGTGAGAGCATTGTTTAAAATCAATATGGACAATCCCACTTCTGAGTTCTGACAGTCTGTTCTAGGTCCGTTTCAGAACTGGAACTAGTTTGCTTTCTCTCTCCTCTTGAACTGGACCGGAATCGACTAGTCCGGTTTAGGCCAAGCCAGCTTTTATTATTATTATTATAAAATTTAAAAAAATCAACCATTGGATTGGATCAAAACTAGAATGAACTGGGTTTGGACCTTGTGGCCCTGTCCCAGGTCCTTGGTTCTAGAAAAAGGAACCAGTGGCCATCTATAGTTTTAGGCATTGAGTTTGTCGGCTGTAATGAAGATCATGAGTTAAAAGTCCATAGATTTTGTGCCCTTGAACTAGTTTTTAAGCTTAGATCCATCCCTCTCATAACTTGACAAGTTTTTTCTCACATTGGAAGAATTTTATGGTAATCTTAGCCACTTTTATCTTGGAAATTAATTTCTGTTGCAAGAAAAGTTTGTTATGCAGTGGAAGAACAAAAAAGTTTTGTTTGTGTTTCTCAATGAAGTTGGGTTTTTGACTTTTGGATTGCCTTTCGATTTATAG**GGTTTTGTTGAAAGGGAAACTACTCTGATTGGGTCCCTCACCGTCCGAGAACACCTATACTACTCTGCACTGCTTCAACTTCCTGGTTTCTTCTGTCAGAAAAAGACTGTGGTAGAGGATGCCATCCATGCCATGTCCTTGAGTGATTATGCAAATAAATTGATTGGAGGCCACTGTTATATGAAGGGCATTCCTAATGGTGAGAGAAGGCGTGTTAGCATTGCCCGGGAGCTGGTGATGAGGCCCCATATCTTATTTATAGATGAGCCTCTTTATCATCTTGACAG**GTTATTATATTTTCTCCTTTCTTCCTCATTTTCATTTCAAAGTGTCTTATTTAGAATTCCACACATTCATTCATGGCCTACTGTGCTTAATTGTATGAGTATTCTGCTAACTGCATTATTGAATTGTAGTTCTTGTTGATGGATAGGAACAACCACTTGTGTACCTCGGAAATAATCTAGATTATATGATACTGGAAGTTTGGAGTGTTTCAAACATACTTGGTTTCTCATGTATCATGTATGTATCTGAGAATGACTATGGACTATCGCATCCGCTGTGTGTTGTGCAAATTCACCAGAATATTACAACTAGCGGGACAATACTTGCCATGGCAGGAAAATTTAACTGTTCAAATTTTAGGATCGAACAGTATCATCCTTTTTTTTTTTCTTATCCATAGTAACTGTTCTGGTAACAACCCCTGCATGGTTGTAATGATCTGTTTGTTATTAGGTAGCATTTACTTTTTGGCCCCCTTTTAGCTAATTTTTGGTCTTTGTCTGATCAG**TGTCTCTGCTCTTCTGATGATGGTTACATTAAAGAAACTTGCCAGCACTGGTTTCACTCTTATCTTTACCATTTACCAGAGCAGTACTGAAGTATTTGGCCTCTTTGATCGGATCTGCCTGCTATCAAATGGAAATACGTTATTTTTTGGAGAAACTTTGGCTTGCTTGCAG**GTAAGATACTGAAATTTTGATCTTATCCTTTGTTCCAGTTGTGGATAAAAGAACATATTTTTGGAATTGGTGAAGGCCAGTTCAAGTGGACCATTTATGCATAAAACAGTAATTGAGAATTTAAGCAACTACCATGGTTGTCTTTTTTGTTCATTTGTTTGGTAAGAGTACTAGTCTTTCTATATTCTATTATAACAATAATTCTTTACTTTCATTTTTTGGGCAG**CACTTCTCAAATGCCGGATTTCCTTGCCCAATTATGCAAAGTCCTTCTGATCACTTCTTACGTGCAATAAACACGGATTTTGACAGGATAATTGCAATGTGCAAAAATTGGCAG**GTATTATTTTACTATTGCAAATATAGTGATTCTCATGCCTGAATTGCACGATTAAGATGTCTTTAAAACAGAAAAACAGTGCAAATAATAATGGGACAAATGTTTGTTGGATCTTATTGGCAATAGTTATTTTTTCAGTCTATCACATATTATTTTGTCCACCCGTGTGATAATATTATGTAATATCCTATTAATTTAGGTCAACTTTAGACATGTAAATTTAATATGCAGTCGTGGTAACAG**GATGACCATGGAGACTTTTCATCAGTGAATATGGATACTGCTGTTGCCATTCGTACCTTAGAAGCAACATATAAATCATCAGCAGATGCTGCTGCAGTTGAAACAATGATATTGAAACTCACAGAAAAG**GTATGAATGAAATTGCAAACCCCTCTTATTAGACTATTTTTTGGCCTATACACATGCTGAATCTTAGTTGTGCACATCATTATTGACTCACATGGGGCACTTGGAGTGTCTTGTTGTTTGGATGTTGTTCATTCTTTGATTGGTTGCAGCTGGAATTGATGTATCAATCTTTGCACTTTTGTTCCTATATTGTTGTTGTTGTTTCATCTCAAGCTAAGCAGTACATCGTATCTGGACCAAAATTGTCTGACTTAATACCATATTGCCTTGTTGGTCATTTTGAGTTTTAAATGGTAGAGGCCTAGAGTGGACTGGGTTTGGGTGGGTTATGGGTCCATATTGTAACAATCAAAAGAGAGAAAGGGGCATGGTTTATGATGAATTAGAATGTATGACTATCGTGAAGTCATCAGCCACAACTAACCTGTTCTGTGTGAGGCATTTGCATGCATGTTAGTTTTCTTGTCTGTTCCTGGTTTCAATAAAGATGCAACTAATAACTTTCATTCTGAAGAAAATTTTGAATCTGAGATTTGCCATAAACAGTCGATTTGCCTGCATTAGATTTTAATCCTTTCATTTTGCTCTTGTTTGCATTGAATTTGAATGTAAACTGAAGTGAAAGAATTTTTATTCTTCAATAACTCCCTTGTCATGTGTGATTACTTATGCAG**GAGGGTCCATATCTTAAAAGCAAGGGAAAAGCTAGCAGTGCTACAAGAATTGCAGTTTTGACTTGGAGATCATTGTTAATTATGTCAAGGGAATGGAAATACTACTGGCTTCGTCTTATTATTTGTATGCTTCTCACGCTCTGTATTGGAACAGTATTTTCTGGCCTGGGGCATTCTTTGTCTTCAGTTGTG**GTGAGTTTCTTAAGATAAAACTTTAAATAATGCATCTGGTTTTCTGGCTTATCCCTGCTTATTGAATGTGTTCACCATAAATATGCATCCACAATCCTGTAACACTGATAAGGTTCATGAGTTGGTTACTTGTCTCTTTCCCTTCAAATTTCAG**ATGAGAGTTGCAGCAATATTCGCATTTATATCATTTACTTCACTTCTAAGCATTGCAGGAGCACCTTCGCTTCTTAAGGAAATCAAG**GTATTGTGTTGCTATTTTTGTTTGGTTTTGATCAAGAAATCTGGCAACTTATGTTTATGTATGAGATAAAAGATGAGTCAAATCGGGTTCAAGAAAATAATGTAGTTCTGTTAATGATCTTAATGATGAGTTATGGCTCATATTAGTTAGAGGGAAATTTACTGGTCAATAAGGCCCTGGGCAAAGGGGAGATGCTCTGTGACGAGGGGCAGAATGCCCTTCTTGAAAAGGCAGTATGGTGTTTTGTTGGGGGTGATTATGTAAATCATATTTGTATTCGGAAACCTGGGTGCAAGGATCAGGTAATTATAAATAGGGCAATAATATTATCCTTATAGTAGTGTAGAAGCAAGTGGTCTCGGATGTAGGCACACTTGCCGAATAATGTAAATGTGTTTGCTTTCTCTCACTATTTTTCTCATCTCTGTGTATGTGTGTTCATGGGAATTTTCGCTAACACTTAGATGCCCAAAATGAGAAGTTTCGAATGCCCAATAAGACTTCCATAATGACCTGTGAAATTGTCACATGAAATGAGCTTCAATTGTTTATTTCAATATCTCTTGTTGAATACCAGTGAAAGCAATATGTTTATATACCATTTGCAGTTTGAATCTGTTCACTTATGCCATCACTTTTTTTAGGAAATTGAAAAAAAAAGTTGGATGGTTATGAATGGCATATAATATCTTGAAAAATATTGAAAAACCATGTTCTCTCTCTCTTGCTCTTCTTTTTGATCTTCTTCTTCTTTTTTTTTTTATTTTTTTTTTTTCTCTTTTCATGATTGATTTCATCAATCTGGTTCCCAATTTTGCAG**ATATATGCCAGTGAAGAATCAAATGGGCATTCTGGGGCATTAGTCTTTTTACTTGGGCAGCTTCTCTCCAGCATCCCATTTATTTTTCTCATCTCCATTTCATCAAGTCTAGTCTTCTATTTCCTTATAGGGTTGCGAGACGAATTCAGCTTGTTAATGTACTTCGTTTTGAATTTCTTCATATGTCTCTTGGTAAATGAAGGACTAATGCTGCTTATCACTTCCCTTTGGCTGCATGTTTTCTGGAGTGTCTTGACGCTGGTATCAATACAT**GTGAGTTTCATTATGGGACATAAAAATTTTGATGATATATATATATATATATATATATGCATGATTGCAGAATCATATTCCTTAGATGATTGGTGTTAGTATCTCTGTTTTTCTCTGTGTGGTGGATTTCCCATAGAATTAGCTGTTCTATTGAATGAGAGTAAAGCTGCATTGCTAAGATAAAAATTCTTTTATATCATTTAAATGTGCACTAAATGGTGTTAATTACTGAGTTTGATAACCATTGGGACACCTCTATGTAAACCAATGTTGGATGTAAATATTATAAATTAATCATTGGTGCGATCATTAATGAATGTTTTACCACCCAATTGATTGTATATTTGGATATAGTGAGTGGTGATTACTTGGTGTCTCCAAATCTCATTTTGCGTACCTGTGTCATTTCAG**GTGGTAATGATGCTTTCGGCTGGATATCTTAGAATTCGAAATGCTTTGCCACGACCAGTATGGACATATCCTATATCCTATATTGCTTTTCACACTTACTCTATTC**AGGCAAGTAGGCTTGTAATTGAATCTTTTATCTTCTCCTTATGAAATACTGTATTCCTTAATATATTTCCTGCTTGAAAAATAATGTTTTAAATTGGCTGTTGTTTGACTTCTTTGAATCTTAATATCCTGCTGTTAGAAAGCAGCAGTATTGGGCATACCTTTCTAACTTCGAAACTTTCAAACGTCGGTACAGTTAGACAAAATTTAAAGCAAGTTTTACCTTCTGAATAGATTTTTAATATTGTTTTCTGTTTCTGCAATGC**AGGGACTTTTGGAGAATGAGTACCTAGGGACTTCATTTGCAGTTGGGGAAGTGAGGACCATTTCTGGGATTCAGGCCCTTCGAAGTGCATACGATATATCTTCCAACAGTAATTCCAAATGGGAGAATCTGTTGGTATTGTTTCTTATGGCAATTGCATATCGTATACTAGTGCTTCTTGTACTGCATTTTCGTGTTGGAAAGAATGAATCAGTACTTAAGTTTTGCCGGTGTAATCAGGATACAAACAATCCAAGATGAATGGAATTTGCCCTGCCAGTCATGTTTATGTTCTGGCCTCTTGTTTCAGTTAGAAACTAATGATGAAATATAGAAATGCATACAATTAAAGGGGATGTTAGCCTGG**

>HbABCG5 scaffold1176(49817-51949)

**TTGATTATACAGCTGACGACAAAACAGCAGCAACTGTGCTTTCTTTATTAGTAAAGAGAGTATGCTAATGGTTGAAGTGGGTGGTCTTGAGAGAGAAGGTAGCTAGACAAGCCAGAAGCCACCCAAAAACCATTGAAAAATAAGGCTTCTTCAATGAAGAAACAGGGCTGTGAGATTGAAGCTAGAGGGATCACCTTCAAGATTTCCACCCAGAAAAGAAACTACCCTTTTAAAATCTTCAATGAAGATCAACAAATTAATCAGGAACTCAAACCAAATCTTGCAGAAAAGCATCTTCTTAATGGCGTTAATTGCAAAGCAAAGCCATCGGAAATCCTTGCCATTGTTGGTCCAAGTGGAGCTGGAAAATCATCCTTGCTTGAAGTCCTCGCCGGAAAACTTACCCCACAAAATGGTTCCATTCTTGTCAACCAAAATCCTGTCGACAAGGCTCAGTTCAAGAAGATATCAGGCTATGTCACACAGAGAGACACACTTTTTCCTCTACTCACAGTGGAGGAAACCCTTATGTTTAGTGCCAAGCTGCGTCTGAGGCTTCCTGAAGAACAACTGATATCAAGGGTTAAGTCCTTGGTCCAAGAACTTGGCCTGGAGCATGTAGCCATGACTCGTGTGGGTGACGAAAGGGTTCGTGGGATTTCCGGCGGAGAAAGGCGTCGAGTTTCGATTGGAGTTGATGTCATACATGACCCTAAAGTTCTAATTCTTGATGAGCCAACCTCTGGTCTTGATAGTACTTCTGCTTTACAGATTATTAGCATGCTTAAGGTTATGGCGGAAACAAGGGGTAGAACCATAATATTAAGCATTCACCAGCCTGGGTTTCGCATTGTGAAGCTGTTCAATTCAATACTTTTGATGGCTAATGGGTCGATTTTACATCATGGAACGGTGGATCAGCTTGGAGTTCATTTGAGGACAATGGGGATGCAGCTTCCTCTTCATGTTAATGTTGTTGAATTCGCTATCGAATCCATTGAAGCCATTCAGCAGCAGCAGCCAGAAAGTACTCCAGTATGGACAGCCCAACAGCAGATCAAGAAAGCAGAGGAAGGTGATAGTAGAAGTGGCAAGTTCACTCTTCAACAGCTCTTTCAGCAATCTAAAGTTGTCGACGAGGAAATTATCAATGCTGGGATTGATTTTCCTCGCGGATTTGCAAATTCCAGGTTTCAGGAGACTGTAATTCTCACTCATAGATTCTCCAAGAACATTTTTCGAACCAAGGAGCTCTTTGCATGCAGGACAATTCAAATGTTGATATCTGGGCTTGTTTTGGGTTCCATCTTTTCCAATGTTAAAGATGATTTGACAGGAGCAGAAGAAAGGGTAGGCCTATTTGCTTTTACATTAACATTCTTACTCTCTTGCACAACAGAGGCTCTGCCAATCTTCTTGCAAGAAAGGGAAATTCTAATGAAGGAGACCTCTTGTGGGAGCTACAGAGTCTCATCCTATGCTGTGGCTAACGGGCTTGTTTACTTGCCATTCCTACTGATTCTAGCCATATTATTCTCTATCCCATTATATTGGCTGGTGGGTCTAAATCCAAATTTCACAGCATTCATCCACTTCCTGCTCTTAATCTGGTTGATTCTCTACACTGCAAATTCAGTTGTGGTATGCATCAGTGCTCTAGTACCAAATTTCATTGTTGGAAATTCAGTGATATCAGGGGTGATGGGATCTTTCTTTTTGTTCTCTGGCTACTTCATCTCCAAGCATGGGATTCCAAACTACTGGATGTTCATGCATTATATATCACTTTTCAAGTACCCATTTGAAGGATTCTTGATAAACGAGTTCTCAAGATCAGGGAAATGCTTGGAATACATGTTTGGGACATGCATGGTTAGAGGAGAAGATGTGCTAAAAGAAGAAGGATATGGAGAGGAAAGTAGGTGGAGAAACGTGGTGGTAATGGTGTGCTTCATCTTTGTTTACAGGTTTATTTCTTATGCGATTCTAAGATGTAGATGCTCTGTCACAAGTCTCAAGGCTTCTCTGGTTTGATGAATCAGACTGTTTGCTCTCTATAATCTCTCCCTTTCACGAGAGTTCACAAGATGGTTCACATGTTTTTGGAATTTACTTAAGATAGCAGCCAAAAA**

>HbABCG7 scaffold2190(6818-18096)

**GGAAAATGGTGGGCCAGGTGGTGAAGTTTGGCGGGAACGGTTTCGGCCAGGTTCTGGCGGCGGCCGCGGTGGCTATCTTGGTCCGATTGTTTTCCGGTCCGGGCCCTGCTCTGTTACCGGAGGATGAATTTGCGGATGATGAGAGGAACGGCGTTCCCGGAGATGATAAAGCTGGCGAAGCTTCGGTCAATGGGAAAGTTGTTCCGGTTACTATCCGCTGGAACAACATCACGTGTTCTTTCTCTGATAAATCCTCTAAACAA**GTAAGTAGATATTCCACCTCCTAGAAAATTGTAATTTTTTACAGCTTTTCTTCAAGTTCTGCTTTGCTGCTAAGAAAATGTGGGAAAATTTGATTCACTTAGCTAATTTTTCCGAGCATTCATTATGCAATTTCAAGGAAACTAGCAAATTGATGATAGTTTAATTGTAG**GTGCGGTTTCTGCTAAAAAATGTGAGTGGAGAAGCAAAGCCTGGAAGATTGCTTGCGATAATGGGGCCATCAGGATCAGGAAAAACGACTTTGCTCAATGTTCTGGCGAGGCAGCTAATGGCATCACCTCGGCTGCATTTATCTGGTCTTTTGGAGGTCAATGGGATACCTATTTCAAATATAGCTTACAA**GTATGATTTATTTTGGAAGAAATGATAGTGATTTTATTTTATTTTAGTTTTTACAATCTATTTCCTTGAGTTTTTAGCGATAGTATTAGGTTTATTGTTATTGCAG**GTTTGCTTATGTGAGACAGGAGGATCTTCTCTTCTCGCAGCTGACAGTGAGGGAGACACTGTCCCTTGCTGCAGAACTTCAACTTCCAGAGATATCTTCGGTGGAGGAAAGAGATGAGTTTGTGAACAATTTGCTGTTCAAGCTAGGCCTG**GTAAGTAGGACTTGTGATATTTGATGTGAATATGTGAAATGAATTGAAAACTCTTGACATGAAATTATCTCCTCTAAATTTGAAGTCTAAGCATTTGCACTTGAAGCTGAACAATCTTAGTTTATATGCTTGAAACAAAAAATGAACCAAGTAAGTCACAATGGTCTTAAGTAATACCTCTTTAGACAACATTTCTTAGACATACACTTCATACAATCCTAATAAAATCAATTCACTGGTAAGTACCGTTTTCTCATAACACTAACACGGTACTAATGATTTATGCCACGAAGGCATAGATAAACAGTAGTCGTCATAGGAGTCGCCACCGGATAATATCCGGGACATATTCGTAATTTCTTTCGAGGGTCATAGATGGCAACTTTTTCATTGTAGAGTTTTTACAACCGTCGTTACTATAGCCCAATGTCTAGATTCGGGATAGTGGATTCGGTGGAGGAAAGAGATGAGTTTGTGAACAATTTGCTGTTCAAGCTAGGCCTGGTAAGTAGGACTTGTGATATTTGAAAACTCTTGACATGAAATTATCTCCTCTAAATTGGAAGTCTAAGCATTTGCACTTGAAGCTGAACAATCTTAGTTTATATGCTTGAAACAAAAAATGAACCAAGTAATACACCGGAAAGATGTTGGAGTCACAATGGTCTTAAGTAACACCTCTCTAGACAACATTTTTTAGATATACACTTCATATAATCCTAATAAAATCAATTCACTGGTAAGTACCGTTTTCTCATAACACTAACACGGTACTAATGATTTATGCCAGGAAGGCATAGATAAACAGTAGTCGTCATAGGAGTCGCCACCGGGTAATATCCGGGACATATTCATAATTTCTTTCGAGGATCATAGATGGCAACTTTTTCATTGTAGAGTTTTTACAACCGTCGTTACTATAGCCCAATGTCTAGATTCGGGATAGTGGGTACATAAGGGGAAGGTAAGGCACTCCTTACGCCTGGTCTAACCTCGTTAATTCGAGTGCCAGTTCTCTACTAATGTTTTTAGAAGTCTTGTTTATTATTCTTTTATAAAAATTTATCCTAAAAATGCAATTTTAATCCTATACACGCAAGTAATTCACAAAAAGGTTGTTGTTTACAAATAATTTTCATGCATAAGTAATTCATATCTATGGGGTTGTTAATTATTTAAATGATATTTACCAGATGACTAAAATTAATTTGTTATTGAGAATTTAATTTATAAACAAATTCAATTTCAAATAACCTTAAATTTCTATTTCACCTAATTAATTAATTTTCACTAAGTTACCATAAAGATAATTAAACTAAATTAATTATGTACATGTGTAATTTATTCTAATATCACATAAGGCTCAAACAATCACAATCTAATAAAGATTTTTAACATGTAAATTTATTTTACAATTATCAAGTATTAAATTAAATTTATTTAATTCCCTTATAGTATGGCAATGATATCCCTGGTTTACTCCCATTTAGCTCTATGTAAGTTCCATGCAAATGCCCTCTTTAGTACTTACCTTAGGGCTATTTACTAATTTTGTTTGTAATAAAAAAATACAGAAACTAAAGAAAATAAATAAAATAAGAAAATACAAATAACAATTCACGTTAAATTCTAACTCTAGTCTCCTAAAAGAGTTAAAATTCCTAATCAATTACAACTACTTAATGGTCTAAGTCTTCTAACATGATCCAAAGACTTCATAACACCTCTTGAATTAAAAAAAAACACAGAAAAATAGATCAAATATCAATTAAAACCCAAAAACACACAAGAATTTGCATAAACATATAATTTTCAGATTTTGGCAGATTGACAATAGTAAGGAATCTAATTTTTAAAAAAAGTTAGACATGCCTAAAAATCATGAAAAAATTACAAAAGATAGCCAACACATCATACAAGATCACAAAATTTATAAACTAAACAAAACCCTAACCGAATGATCTACATAAATCGTAACTTTCTTAAGTGACAGAATCGCACTACTTTTTTGTAAAATTCATAACTCATTAACCACAGAATGAATGCAAAACCAATTGGAAAAGATTCATAATATTTTGAAATTAATTTTAGACACCAGATTCACACAAAATTATTAAGAAACAAGAGAGATATGGGCTTCACAAGTCGGGTATACTGCAAATTGGATTTTACAGGGACCCTAACTTCAAACAGCCATAACTTATAAAATACAAAAGCTTTTTCAGTGATTCTTGAGCCTAAAACTAATAGAATTTCGTGAAGAATATGAATATAATTTTACTAAAATTTTTACATATTTAGAAAATAACAAAAAATATTAAAAACGCTAGACAAAAAACTGAATATGTGCAGAGTTGTGTATCCCTTCAAATTAAACATGATTACATGATCCACATGAACATGTAAGATAAATTAAAAGACAAAAAATATAGAACTAAATATTACGTGGCATAGATCTTGAAAAGAAAACAACAAAAACTAACCTTCAAGAAATCTTGCATGAAAATCTTCTTGAAGAAAAGAAAAAAAAATAAGAAAGAACTAAAAGAGTTTCTAATTATCTCTAAATTTTCTATAGCTCTAAGGTATTTCTAAATAGCCCTGATATTTCTGAACCTTCCCCTTTTCTGTTTTCTCCAGTAGAGTATTTACAGGGTTTTGAGAGAGAAGTGTGATAGGGTTTGGAGTTTTAAGGTGATAAGGTTTAGATTATTTAAACAACTGGGGTTGCAGAATTTGGGTGAGACTGGGATATGGATACGGTGTTCCAACTAATAGGAAGAGAGGGAAAGGGGGGCCGCACTAATAGAGAGTGAGGAAGAGAGTGGGACCAAGTGGGAGAGAGAGTTTGTATGGGAATGGGAGAGGGAGAGAGAAAAAAATATTTTTTTTTTATTTTAATTTTCAGAAAATAAATTAAATGGGTTCTGTTTAAAATTTCTAACTAGGCTTTCTTCAGTCCATAGGGCCCAATTAAACTTAATTAGGTCTATTTAAGAACTACTCATATTAAATTTAAACAAAATAAAATTTTATTTAAATTTAATTAATTTTTCCAAAATATATTATTATTCTTTTCTTCAAGAACATGCCATAAAATTAATAATTCAGTTCTATGCCTCCAATTATATCCTACAGTCATACAATTTTTCATAATCGGGTTCCCTATGGCCTCAATGCTCATGCTCATCACAAAATAAGGGTCTACAGTAACTTGGTTTCTTCATCAATATCTCTTCTGAGTAAATAATTCAAAATAACCATAGTGGTTTTAATGTGAAGCGATCATGCACTATGATTCATCTTTCACTTATTTTATTTCATTCTATTACTTCTGATTTAATTTGATTTCTTTCTGGGGTTTGCTTTTGTTCTTTATTTTCAAAG**GTCAGCTGTGCTGATTCCATTGTTGGTGATGCAAAAGTTCGTGGGATAAGTGGTGGTGAAAAGAAACGATTATCGCTAGCATGTGAACTTATTGCCAGCCCATCTGTCATATTTGTTGATGAGCCCACAACAG**GTATGCATGCTCCATGTGATCTGCTCAGACGATAGTCCTCAATGGGTCTTCTTTCGCAAGTTACATTTCATATGTAATATTTTCACCTGTGTCTCACTCCATGATTTGCATTTTCTATATAAAGCAATAATTATTAAATGTAAATCACTTAGACGGTGTTTGGTTTGACTTATAAGTTGGTCAAACCTACTTAGAAATAGAAAGTTGTAAGCATATTAGCGTTTGATTTAGCTTATAAGTTAAAAAAAAAAAAAAACTACTTAAAATAAGTCAAAAAGACTATATTATCCTAACAATTTTTAAAAGCATTATCCTCATTTTAACACTATAACACTTAATTACATATCCTTTTTGGTAATTTCAATAAATTAAATTACTTTTAAGCACTTTTTAACTAAACACTTAAACTACTTAAAAGTTTTTTTTAAGTAACTTTACCTAATAATTAATTACTTATTTTTAATTTGACTCGTAAATTAAAAAATATATTTTAGGTTAAAAGTTAAAAGTAAGTAGGCAAACCAAACATTCTCTTAGTTAAACTTAACAGAAGTGGGGTAAACTCCTTATCAATTGAATCTTAATGCACCTTAGAGGTCTTGGTAGGAGTACAAAGAAATAGAGGTTATAATATGCCATGTGTTGGCTGTGATTTCAACTAGTTGGTTGTATTTTTCAACCAAAATTTAGATATGCAGTTCTTGTGTGACAAATTTATTCTTCGCTTCTTATAGACTTTTTGTCAGGGTCTAAGAATGGTGTTCTCAGTTAATATTCAATGTGATTGGAGAGTGGCTCCATTAGACTCTAATATATTAACAATTTCTCTATATGTATTGAAATTTCCCTTTTTCTTTGATAATAAAGTCCTCTTGCTGAAAAAGAAAAGGAAAAAGAATCTTGATGTAATGAATTTTTGTTGCATTGAACTGTTAGCAAATAGACAATTGTAATGTGATTGGGTCCAATCAAATTTGAAAAAAGGGCTGTTATTTCTGGGCTTAGTTATGCTGCTTTAAGCTACATTTTTTTATTAGCTTCATTCACAAGAACTGAAAAATCAATTTACTGTTGTTCCAGTGATTTATGTTGTTGACTGCTGAATCGATGTTTTTCAG**GGCTTGATGCTTTCCAGGCAGAGAGAGTGATGGAAACTCTCCGACAACTTGCACAGGATGGACATACTGTAATTTGTTCTATTCACCAGCCCAGAGGTTCTGTGTACAGTAAATTTGATGGCATTGTTTTGCTAACAGAGGGTGCACTTGTTTATGCTGGTCCTGCACATGATGAACCACTGGCATACTTCACAAAGTTTGG**GTTAGCCAGACCTCTTTTTTCTCTTGTTGATTATCTTTATGGTTCATACTATGGATAGATTGTTTGTTATTTTCAAGATCTTTTGAATTTGCGACATTTCTCTAAATTATTACTGTGGGGTTTAGGAAGATATGAAGATCCAGGTTACATAATACATACTAAAGGAAAAAATTTTGGGTTAGCTTAAAGAATTGCACAAATGGATGCAAGCCCATGAAATTCACCTACCATCACTTGAGAATCAATACTGCTGGTGGAAGTGGATGTGGAACTAATGTCTGCCATGTGGATATAACCTTTTATTCATTCAATTATAGTTATCTGTTGATCAACTCAACTTTTCTAACATTTGTAATTTTTCAACATCTTAATTTTGATCTTGTACATCTTATGATGTTTCATCATTGTGTTATAGAAGTTCTGATAGGTATTATGGGTAGACTATCCACAAATAAATACTTCCAGCTTGCATCATTCGCCTTTAGTTTAATGTTGTATTATTTGGACCAGGGTATTATATATGGCAGTTTCTGTTGAATATGTTGCCTCCCCTTTCTATGGCATATAACTTTCTTCTCCGTTTTCGAGTAAACTGTTAGTGATTACATAGAAATAATAAATTATGATTTGTTATGTGTAGGTGTGTGCAACGTTGAATAGTTTTCATTTCTCTTTTGGTTTTTTTACTTTGGAACATTTGATAGCATTTTTTTAATGTGTTTTGGTTTCCTATTTAGAAGCTTTTCCTTTGTGTTTTATTACTGTTATTCTTGGCTAGCATTATCCATTTCCATGTAAAAAAACTTGAGCTGTAGACTAGGCTTACTGTCTTCACTGCTGTAGTTTTTCAAAAAGAAACAGAAATATGTTTCATTTGTGCATATAAGTGATGATTGATCATGTAAATGAATATATAGATGATTTTGCTACTATTAATTTTCAAGAGAACTGATTAATAATTGATTCATTCAACAG**GTATCGTTGCTCAGATCATGTTAATCCAGCTGAATTTTTGGCTGATCTCATATCTGTTGACTACAGTTCTGCTGAAAGTGTGTATTCTTCCCGGAAAAGGATAGATGGCCTTGTTGAGTCATTCTCAGAACAATTATCAACTGTGCTTTATGCAACTCCCTTTGCCAGCAGGGAGAGCCCTAAGAATGGCAAGAAGTTAAGCAAGAAAACTGTTGTAAAAAGGAAAGAGACTTGGTGGAGGCAGTTCTGGTTGCTCCTCAAACGTGCATGGATGCAA**GTTAGTTTCTCAACTGTAAAATAATTTTTTTTTGTCCAAATGTTCTCATTCTATGACATGGTGTGACGCAGCCAATGCCTTATATCTCAGAAAAAGGAAGTCTTCTCAAATAGTTGGTTTAAAAATAAATTACAGCCTTGACATTGTTTTACTTAGTTTTTTCTGCATCCTTAATCTTAGCTTCTCTATTTAACTACTAGTCAGCCAAACGGCAATTTCTGAGAGCAAAACTCTTATCTTTTACCCAATTTTAGATGACTGATTCACTCCTTGGGACCAGAATAGCAAACACAGAAATACAGTGTAGGACAGAATTTTGTTATGCAAGTTCATTGAATGTATTGGATTCTTGTCGAAAATGAATGGGACATCTAGGATGGTCAATAACCCTTGTAGTTTGTTCACATATATCAGTTCTCAATTAACTATAACCTGATTGGCTCCTTTTATTGAGTTGTTAG**GCTTCTCGTGATGGGCCAACAAACAAAGTTCGGGCAAGAATGTCAATTGCGTCAGCTATAATATTTGGATCAGTTTTCTGGAGAATGGGAAGATCTCAGACGTCCATACAGGACAGGATGGGATTGCTCCAG**GTGTGTTACGTCGTTGATAATCTTTTGAATATTTTAATATATTACTGTGGCAGTATACTTATATGGAGTTTGACAAAAATGCCTCAAGCTACAGTTCCTTGATACTTTATATGGACATTTGATCTTTCTCAG**GTTGCAGCAATAAACACAGCAATGGCTGCTCTCACGAAAACCGTGGGTGTGTTTCCCAAGGAACGTGCAATTGTGGACAGAGAGCGTGCAAAGGGATCTTATGCATTAGGACCATATCTGCTTTCTAAATTGATAGCTGAGATTCCTGTTGGAGCTGCATTTCCATTAATGTTTGGTGCTGTATTGTATCCCATGGCACGTCTCCATCCCACTTTGTCTAG**GTTTGTTCGTCTACCATTTTGATTGTTTTGACAAAGATATCGATATACAACCAGAAAATCAAGGCACTACATGATTCTAAGCTAAAAGATAGGACATTAACAGAATTTGACCAAGCAATGACTCTAAACATAAAGAAAATTTCTGCAGTAACTTGAATGATAATGCCCCAGAATCTCATATTACTGGGGGGTTCATGACGTCCCAATAATTGGGGGAGGGGAGAACCTCGACTAGTCTCCTTGATTAGCATGTCTCAACATCAGGCCAATGTGCAACCTTGCAATTTTTTTTTTTTGGGAATAAATGTTATAGGGCTTTTATATAATTTAGAGATGCAACTGAATAGCAAAATACTTGGATATATGTGTTAAGTGAAAAAGACTTGTAAGTAGAAATGAAAAATTGAAGGCCCCCAAAGGAACAAGAATATCGGAAAGTTTCCTTGGTGGTAAGTCCATAATCCCCAAAAGTAGTCATCTCATTAAGTAGGTGACACTGATTTTGCAGTCCCTAGATTTATCTAATGGAACATTTTTCATTTTTGTTTATGGAATAAAATAAAGTAGGATATATTGTCATGTGAACCTCAATCTAAAATTGGGGGAGGGGAGAACCTCGACTAGTCTCCTTGATTAGCATGTCTCAACATCAGGCCAATGTGCAACCTTGCAATTTTTTTTGTTTTGAGAATAAATGTTATAGGGCTTTTATATAATTTAGAGATGCAACTGAATAGCAAAATACTTGGATATATGTGTTTAGTGAAAAAGACTTGTAAGTAGAAATGAAAAATTGAAGGCCCCCAAAGGAACAAGAATATCGGAAAGTTTCCTTGGTGGTAAGTCCATAATCCCCAAAAGTAGTCATCTCTTAAGTAGATGACACTGATTTTGCAGTCCCTAGATTTATCTAATGGAACATTTTTCATTTTTGTTTATGGAATAAAATAAAGTAGGATATATTGTCATGTGAACCTTAATCTAGTGTTCTTTTTATGCTGAACTTATTTGGATGTAAGTATGTGAGATGATATCAGGAGGTGCAAAATGCTTACCTTCGAGCCTAAAACTGCTTTAAGAGAATATGACTTGAATCACAAGGAGAGTTAAAATGCTTATTTCCTTCTTCATAACTACAATAGTTTTCAACTCAATGATGGTTACTGATTTTCCTTTGCTAATAGAATTTCCTGGTTGGTATAGAAAACTTTTTCACTTATTATTATGTCTCTGTTGCAACATTTATTGGACGTGATAACATTAATCAATGTTAATGATGTGATGGGGAAATTTTGTATCAAGCATGTGGTGCACATGACAATAGTGCACTTTCATCTCAGTCACAATCATAGGGGGTGTCCATCTATGAATTTAGCTTGTGAATTCAACATGAATGTGAGAAGAGAGTGCATTATTTTCATGTGCACCATGGATTCTGAATAATTCCTCTGTTGGACTGGCTAGTTTGCGTATCTTTATTGTTGATGAAGAAGAGAGTGCATTATTTTCATGTGCACCATGGATTCTGAATAATTCCTCTGTTGGACTGGCTAGTTTGCGTATCTTTATTGTTGATGCATGGAAGGCAATCTGTTAAGGAATTTATTGGTTATTCCATTCATAATTCATGAAGTTACTGAATTATAACTCATGTTCTAG**GTTTGGAAAGTTCTGTGGAATTGTGACAGCAGAATCATTTGCTGCATCTGCAATGGGTCTAACTGTCGGAGCTATGGCTCCAACCACTGAAGCAGCAATGGCACTAGGACCTTCTCTTATGACAGTTTTTATTGTATTTGGAGGGTATTATGTCAATGCAGATAATACACCAATCATCTTCCGATGGATACCTAATGTTTCTCTGATAAGATG**GTAAACTGCTAATCATTCATATTTTAGACCCTTTCTTTCTAATTTTTTTACTAGATGATGATACACCAATCATCTTCCATTGTTCACCCACCTCCCCTCCTCTTATCAGTGATTGTGACTCCCTTGTCTTCTTGTGTTTTTTAG**GGCCTTTCAAGGGCTTTGTATAAATGAATTTAGCGGACTTAAGTTTGATCACCAGCATTCATTTGATATTGAAACTGGTGAACAG**GTAAGCTGACCTCATGGACTCCAGTATTTTAGATTGCAGCAAAAAAGGGTTTCCTGTTTTGGTGAAGAAAAACAGAATTTTCAAAAAAAAAATAAAAGATTTATTTTAATTAGAACCTTAATCACATTGAAGATTTAACTGGAAATTTGATTTTGCCAGGGAAAAATGTCATACAGTTTTATAAAGATGTTCCGTAATATTGTTTGTCTTTTAATCTTTTACGATTATGGTAAACTAGTCATATGCAATTGCACTTTGCACAGAAAATTAAAGATAAAATTATATAATAGTTTTAGTGACCAGCTCAATTAGTTCTCTTCTACTGTGAATTCTTTTGTAGTAACTGGAAATTCTACAGAAAAGACATTGTCAAATTGGCTAATTTATTTTAACAAGTTAAGCTGCCACTTCTCTGAAGTTGATTTATATGTATATTAAATCTTATTTTGCAG**GCACTCGAGCGGCTCTCCTTTGGAGGAAGCCACATTAATGATACTGTGATAGCTCAAAGTAGAATTTTGTTATTTTGGTATTGCACTACTTACCTTATCCTCCAAAAAAACAAGCCCAAATACCAACAGCTTGAGTCACTGCCTCTTGAACAAATCCAACCACAGCTACAGCTTGAGCCCGTGGAACCTGACAAAGTCAAACAACTAAACCCTCCAATGAAACAAGTCGAATTGAACCAGCAGCTTGAATCACCAGCTCTTGACCAAATTCGACCATTTATCTTAGAAG**GTTTGTAATTATTTGGGCATTAATGATATTGTTATAAGATGTGCCTGTATTTATTGATTTATCTGATACAG**GTGCTAAGTAAAGATGTCTATTGCAATAAAGGAGGTATCGTGCCTTTTCTGAGCTACTCTTATAGAACATGACAACAATAAGTTGATTTATGTATATCTGAAACTGAAGCAGCTTAGAAATCTCTCAGTGGAATATCAAATTATGTCATCTGCCAGTATAATTGAATGTTGGCACTCTGGTATTATCCTCTATTGCTCCAAATCATATTCAAGCATATGCGCTCCCTGGGTAAAGACAATGACTTAGCATGCATAGAAAGACATAAGAGCATAACAATGTGTATTCTTTTACCCAGCATGAGCAACCATTTTCGTTGACGTCAGTCTGTTCTGCCATTTTGAATTTTTTAATGCATTGATAGAACACCGACTCTTTTTGCATTGTAAAGTTCTCACTTAAAACTAGCTTTGATAACCATTTGTGGTTTTGTATCAGTAGAAAGGCG**

>HbABCG11 scaffold1370(15205-25965)

**AGGATGCCTGCATTTAGTGGGTGGGATATATATGGCACATTTTTGCATCTCTCGGGCATATGTGGCAGAAGAACATTTTCCTTTGGCTAGTTTGGAACAGTAAAGCCACTGCTATTTTCAAGCTCACTTGCAAAGAGGAACACCTGTTCATGAAGCAAATCTCACATATCCTCCCGTGGCTGCTTCTCTTCATCCTTCGCAGAGACTAAACGAAAGTGATCTTAATAAACCTAAGCATCCTTTTTCTTCATCACCATCTCTGCCTGATAATTCTGAAATCATGACTTCACTGGAAATGGAAACCTCTGATATTAACTCGGTTCAAACTCATACACCAAAGGAAGATGTTGATGATGCTGTTTTCTTGAGTTGGAAGGATTTATGGGTGACAGTTCGTGATGGAAGACATGGAAGCAGGTCAATCCTTCAGGGTCTTACTGCTTATGCCCAACCTGGAGAACTTCTTGCTATAATGGGTCCTTCAGGTTGTGGCAAGTCAACCCTTCTTGATGCGTTGGCAG**GTATTGATCTTGATCGTTCTCATATATAAGGACTACTAATTTTATTTAGCTTAAAGGCCTCATGCACTTTCTTACAAACCCTAAAAAATTTCCTTTTTAATTTATGTTATTTAAATATATAAAAATAGTATAAAATGTGAATATCTTCGGTAACATCAAATTTTAAAAAATATATATTTGTTTCTTTTACAGATTCCAATTGATTCTCTTGTTTACTTATATTTTTTAAATTGGTTTTTTTATAAATTTAAGTATTAATTTTTTTTTAGTGGATGATGTATTTTTATTAATGGGAAGTTAATTGTATAAAAAAAAATTTTTATTGGCCTCATTTTATGCATTGAAAATCTTAACATATATGAAAATTAATGGATATGTATAAGATTGATAAGGCTGAAATTGAGTTATGATTATATCGCAG**GGAGACTGAATTCAAACACAATACAAGCTGGAGAGGTCCTCATCAATGGCCACAAGCAGGCACTGGCTTACGGAACAACG**GTGAGCAGAATTTAAAGAATAAGAAACATATATTACTTTGATCTTGCATCTATGAATAATATAAAATATTCGACATGATAACAGTAGCAACAACAAATTATTTTTGATTCCAGTGACTAATTATCCTTTCGTTATTTGTAAAATTGTAATAG**GCTTATGTGACGCAAGATGATAATTTGGTCGCAACATTAACAGTTAGAGAAGCGGTTTACTACTCAGCTCAGCTTCAATTGCCAGACTCAATGTCCAACTCAGAAAAGAAGGAGAGAGCAGAGAGGACAATAAGAGAAATGGGTTTACAAGATGCCATGAACACAAGAATAGGAGGCTGGGGAGCTAAAGGCCTCAGTGGTGGCCAAAAGAGGAGAGTAAGCATTTGCATAGAGATCTTAACACACCCAAAACTTCTCTTCCTTGATGAACCCACAAGTGGCCTTGATAGTGCAGCTTCATATTATATCATGAGCAGAATTGCTAGCTTGGGCAGGAATGATGGAATCAGAAGAACTATCATTTTTTCCATTCATCAGCCTAGCAGTGAAGTCTTTCAGCTTTTTAACAATCTTTGCCTCCTTTCTTCTGGTAAAATGGTGTATTTTGGTCCTGCTTCTGCAGCCAATGAG**GTTAGCATCTGAAACATTTCACAAAATTAATTGATTCATGGTAATCGTTGGAGTAACTATATACTTGAAGTACTAAATAGAAAATAATCACTCTTTCTTTTGATTTTACGTAG**TTCTTCACCTTGAATGGCTTCCCTTGCCCAACCTATCAGAATCATTCTGATCACTTCTTGAAAACCATTAATAAGGATTTTGGAAGG**GTAAGCTATGTAAACCAATCAATAATATATATTTTTTTCTTCAAAACCATTATGAAAATAGCTGCAAAATTGGGGGAAGGGGAAGTTCCATATATCTGATAAAGAATGCTCCTATGACCAG**GATCTTGAGCAAGGAATGAGTGATGCAACGCCTACAGAAGAAGTGATTAATACCCTCATTCAATCCTATACATCGTCTGAAACTTACCAAGAAGTTCGAAAGAAAGTAGCAGAAATAAATAAAGAG**GTAATGTAATAACAAATGAAAATTTCAAATTGTAATTAGTTTACTAAACACATTTAATAACGGATCAGAGGTACCATCAAGCGTGGTGGCTTAGTGATATAAGCTTTTGGTGTTGGATGCATTCAACCTTGGTTCGACTCCCCACTCCAGTGTTGACTAAAGATTGTCTTCACTTGATATGAATGGGTGAATTGACTGCAGATTGTCTGTACTTTTGACGCCTTATAATTCACTATGACTCAATGGTAAGTCCTCTATTAAGTGAGGTTAAATTAGGGGTTATGAAACCATTTAACAGTGGACCTTGGATGTAGGGGCAATAATTGTTACATCCGGATAGCTCTTATTTTCTCCAGTTAAAAAGAAAAATCAAAGGAGGATATTGTTTTTGCATGGATTTTAG**GATTATGGAGCAATATTGGAGAAGAAGAGAAGTCATGCTAGTTTCCTTAATCAATTTCTTGTTCTTACAAGAAGATCCTTTGTGAACATGTACCGAGACATAGGATACTACCGGTTGCGCCTATTTGTCTATGTTGGATTGGCTTTCGGTCTGGCCACCATATATTATGATCTTGGCTCTAGCTATGGTTCCATCCAG**GTAACTAGTCACTTAAACATACGTTATAAACAGCTTAGAGTATCAAAATTAATTTCATTATATTTGTTTCCCTTGTTCCAG**GCTAGAGGTTCACTGCTTATGTTCATATCCACATTCCTCACGTTTATGGCCATTGGTGGATTCCCTTCTTTCGTGGAAGAAATGAAG**GTAATTTTCCTTTTCTATAAAGATTATCTTGATCCAAACTTTGTTTAATTTGAGGGATTATAAATTAGAGCTCAAATGAGACTATTTGAGAGATTTAATAAGCTTCATTTCTTTTATTCTTGATAGTTATTTATTTATTTTTATCTGAATACACATGTATAATGTCTCTAGTTATATATACTTATGTGTATTTAATGCTAGTAAAGAGTAGCATTATACTAGTAAAGCTGGTACTAGAAGGAGAAATAATAAATGTAGTTAGTGTTTATGCCTCGCAAATAGGACTAGATAGTGTGAGTAAACAAAGGTTTTGGGAAGATATAGATGATTTAATGCAAAGCATTGTGACGCCCCTCACCCGTCTACAGTGTAGCCGAGTAAGGAGTGCCACATTTGATGCTGAAGCACCCTATCTTATTTTATCATATCTATTGTAAACTTTTGATGTCATTTAAAACATGTATTCTATGTGTAGAATTTTTTTTTTATAACTTATTTCTGTGGAGACCCGGATAGAGCCTCCCCTGTTTTATTAGCATCTGGCGGGTTTTCCACTAATAACCTGTTAATATGTCCATATTCATTTCACACATTTTCATATCATATTCTATTGTATCATTACCTTCACAATTATTTATAAGATCTCAAAAAAATATCATCTATTGCATTCATATAGAACTTCATAGATAATAAATTACAAGTTTTTATTTCAATCTCAAGTTTTATTACAAGTCCAAAATGAAATACATCATGACTAGACATAATGAACCAAAATACTAGTCTATACATGGGCCCTACCAAGATACAAAAAACTGGTGAGGTGACTCTAGTCGAAGGCAAATCTGGTTGGAACTCTGTCCGAACACTACTATGACGGCTGCTGTTGTGGCGGCTGCTGATCTTCAGTACCTACGCGATGGAAAACCAACGCACTAAGCATAATGCTTAGTGGTGCATAATTTATAATAACAATAATTAAACGATTGAATTAATATCTGTTAATTATAATTTCAGGGTCTTTCACATTTTTATTGCTCTTTGAAAACAATTAACATTATCGAGATCTTTTATGATTACTTATTGTATTTTAAATCTTAATTAGTTTTTATTAGTGCCCAAGAAACCTATAACAAATTATAAAAGCTGGATGCGTGGGTGTATACTGGTTAGACAGCCATATGTCTGTCCAGTATACGTCTGTCGGGTATGAGACCCGCTGTCAGGCTTACAAGTCAAAAATAAAGTTAGGCAGAATGGCCAATGGGTAGGCATATAATCTGTAGAACAATCATATCAGATATATATTGTCAGTTCATGATTTGCTCTGTAAGCAGTACTGCTATTTGTGGTCCCTAATTGGTATACCAATTGATCCAACTATATACATATAAGCCTAGGCATACTTTGGGCAAATTAGTACTATTAAGTACTTATGGTTTTATTATTTCATACTATATACTATTAAGGGTTCATAACATATTTTTATTTCACTTCATGTACTACCAATGCTTTATACCATTTCCATATTATTATAATGGGAACAAGGTTCATAACATATTTTTATTTCACTTCATGTACTACCAATGCTTTATACCATTTCCATATTATTATAATGGGAACATAGGTCTCAAGCATTTATTCGTATAACTTATGTATCAACTAATTTCATGTCACCTTGTAGTGGGAAAGTAGGTCTCACATACCTATTTTATATAATTTAGCATGTAGAGACTTATTAGGGATAAGTCCTTACTAAAAACAATTTAATTCAAGAACCTTTCTTTAGGTTCAGTTTCACTGTTTTGACTTTACATATCACGTTGAGCAATTACACATTATTACTTATTTCTTTGAAATGTATATATATCACTTTATAGTGGAAAAATAGGTCCCATATACCCTTTTATGTGGTTTAGTATTTAATGGCTTGTTAGGATTAAATTCCTACCATAAGATAATTTTATCTCATGGACCTTTCTTTGGTTTCAGTTTTTGTGTCTTACTTTTATCTATCTATATGATCAATTTGTAGCCACTGTTTGGCCACACTTCCTTCATGGAAGTTGTTCTTCTATGTCTTATATTTATTTTCCTTTTTGAATCATGTCAATTTGATTTTTAGAACTCAAGTTATGGTCAGATAACCATATGTCTTATATTTATTTTCGTTTTTGAATCATGTCAATTTGATTTTTAAAACTCAAGTTATGGTCAGATAACCATAACTGACTCATTCCCAGATTCTCGTTCCTTAACTAGACAGCTTTTGGACTTAAATTTTACCTAATTAATGGAATAGGTTACAGTCACTTTTAGGCATGGTGATCTTCATAAAAATTGTTGCCCTCTTTCTTATGATCATTGAGAAATTTGTATCACAATTTTTCAAGTTTTTTGGAATTTTTTATGGCCAATTAATTGACCTGGGTTCATGTACCCTGTGCCAACAGGGGCTTAGTTTAGGACAGTGATTGCAGGTCACTTTTTGAGATTCTTAAATTCATAATTTGAGGAAGTTCCTAAAACAAAGTTGTAGCCCTATTTCTCAGGTTTTTGGAAAGGTATGGCTCATCTCAATTGGATTTTTCTAGTGGGAGTTATGATTAAAAGATTGTTCTGGGGTCAAGTGAAATTTGGTCAGCTTCTAGGTTTTGATGCAGTAACTTGTTCTAACTATTTGACCTAGTTCTCTTGCTTTTTGGGTTTTGGTCAAAATATCAATTTTGTAGGTATATATCTTGTGGACATTTTGCCAATAGTTTCACTACATTTGAGTTCTTTTAGACCAAGTTATTGCCATTTTACCAAAAATGGTCGGGTAAGTTCATGTCCAAAAATTTCTAGGAAACACAGGTTTGGATAGTTTTGTAATCCAACTTGGGCCAGCAATTTGGTTTGGTTCTTGGCATTTCTGGGTTCAGTGGTCCGCATGAAAATTGTAGCCCTATATCTAAGCTTTCCAATGGTATCAAATTCAAGTCATTTGGACTAGTATAGAGGGAGTTATGACCAAATGAACATGTATTATTCATTTTGTCATTTTCTACGTTTGGTGCAAGGTAATCTGGATTTGGGCAGTATTTAGGTCAAGTTTTGGATAGAATTTAGGTATGGTTTCTTCATGGAAAATGGGCTATTTTGGGTCTAGTTTCACTCCAATTGGCCTCATACCAATTGAAGCAACACACAATCAGTTATGATTCCTCAAACACACTGGACTCATTGAGTCCCAAACCTACAGAAAATGTCACTCCCAATATTCAATCTTCTCCAACTTCCTTACTTCAATTTGACATTCAAGGCACTTCTAAATATCATCAACACATCTCAACAATCCCAATTGCAAGGTATAATACAAAAGTACCCAAGTTAGGTCAAACCCTAACTCGCAATTTCAACCTTATGAAACTTCAATGTAAATCAACTTCAATGTAAATCAACTTCTAATACTTATAAATGCATCAATCAACATCACTAAATCACTTTATATACACTAGAGTCATCAAAGACCATCACTCACCATGGGTACCAAAAATTCACTCCCCTCATAACTCACCAATTTCTTTTAGTTTCTTACAAATTTCACTTCATTTTAACCTTAACTCAAGGATTAATAAAAGAAGAAAGGAAGATTAGGCACTAACCTCTTGTGACCCACTTCAAACTTTAACATTTCTTCTTCTTATGAATATCTAAAGCTTTCTTATGGTGTGGGCTAAATTTTTTATGAACCGAGCTATGGGTTTTGATGGGTAAATGAGGGAGGAATCAAGCTCAAAGCTTGAGCAAAAATAGTGGAAAAGAACACCATGGTAGCCAGCTCAAAAGAGGGAAAGAGAGAGAAGAAGCTGAGTGGAGAAGAAGAGGGTTGGTGACTTTTGTCATCTTGTGTCTATATATATGTATATTTTTATTGTACCTTATTTTTATTTTATGTCATAAGCCTTTTTCATTTTCTTTTTCTTTTCTTCTTCTTTTCTTTTCTCATTTTCCAATTCATTTCATAAATTTTTAATTTATGTTAATTATTTTATTTCCTAATTTTAATTTAATATTTAGGTCAAAATTCACCTCTAGGGGTGAAATGACCAAAATGCCCTTCATGGTGCTCATCAGGTTATTTTTATTATTTTATACTAATTAATAAATTCTTTAAATTTTATTTTTTCTAAATTTTCTTTAACATCTTTGATGTTATTTTTACCTAAATAAACCTTAAAATTGGGTCTCAAAATTATTTCCTATATTTATTAAATTTTCTTTACATTTATTTTATTAATTTAGGTCTCTTCACTATAGTTTAAGTGTAGTCCCATACAGTCTGACTGTCCGAATAGACATTGGTCGTCAGAACAGTAGAATGTACAGATTACCTACGGTGAGGGCGTTACAAGCATACCGAATGAAGATAATATTTTCATCAGTGGAGATTTGAATGGACATGTAGGAAGTGATAGGCAAGGTTATGAGAATGTTCATGGAAGTTTTGGTTTTGGCAGTCGAAATGAAGAGGAAAAAAGTATCCTGGATTTTGCTATGGCATACAACCTAATACTAGCAAATACCTACTTTATAAAAAGAGAGTCACATTTAGTGACTTTTAAAAGTGAGCAATATAGAAGCCAAATTGACTTCCTTTTAACTAGGAAGACAAAAAGAGCTCTATGCAAGGATTGCAAGGTCATTTCAAGAGAGGCTTTAACAAGTCAACATCGATTAGTGGTCTTGGATGTCAAGTTTAGGAACAATTCAAGTAAGGTTAGAAGAAATAGTGTAGCTCGAACAAAGTGGTGGGAGTTCAAAGGAGTAAAGCAAGTGAAGTTCAAAAATGAGCTTCTTGAGTCCGAAGCATGGAAACTGGATGTGGAGGCCAATGATATGTGGATACAGATGGCATCAAAGATTAGAGAAGTAGCTAGAAAAGTACTTGAAGATTCTAGAGAACATGGACCACCCTCAAAAGAGAGATGGTGGTGGAATAAGGCAGTACAAAAGGCAGTGAAGAGAAAAAGGAAATGGTATAAGAAATTACCTAAGTGTGATAATAATGAGGCATATGAACAGTACAAGATAGTAAAGAAAGAGACAAAAAAGGCAGTTAGTCAAGCAAGAGTGCAGGCTATTGAAAAGTTATATGAGAAACTTGGAACTAAAGAAGGAGAGAAAGATATTTATAGATTAGCAAGGAGAAGAGAAAAGAAATGTCAAGATCTCAATCAAGTTAGGTGCATTAAGAATAAAGAAGGAAAAATATTAGTAAAAGATGAGGACATTAAAGAAAGATTGAGAAATTATATTGATGATCTCTTAAATAATAGTAAAAGTGGTAATAGCGTGAATATAGACTACAGAATAATAGAAAAGAATATGAATTATACTAGAAGGATTAGATTTTTAGAAGTAAAGTAAGCACTTAAGAGAATGAAAGTGGGTAAAGCCTATGGATCCGATGAAATACCAATTGAAGTGTGGAAGTGTTTGTGAGATATGGGAGTGGCATGGTTAACTAAATTATTTAATAAGATTCTAAACTCAAAGAAAATGCCTTATAAATGGAGGAGTATTTTAGTACTTATTTTTAAAAATAAGGGAGACATACAGAGTTTCTCAAACTGCAGGAGAATTAAACTCATGAGCCAAATTATGAAGTTGTAGGAGAGAGTTGTGGAACATCGAATACGTCATGACACTTCTATCTCTCCCAATCAATTTGGCTTCATGCCCGGTCGTTCAACTATAGAAGCGATCTTTCTCATTAGATGCTTGATGGAGAAATATAGAGATGTGAAGAAAGATCTACACATGATTTTTATCAATTTGGAGAAGTCTTATGATAATGTTCTAAGAGATATCTTATGGAGAGTGTTAGAAAAAAATAGGATATCTATTAGGTACATACAAGTGTTAAAAGATGTATATGAAGGAGCAACTATTATTGTGCGCACAGTGGGAGGGGACACAAGAGGTTTTTCTATCTCAATTGGATTACACCAAGGTTCAGCTATAAGCCCTTACCTTTTACATTAGTTTTAGATGAATTGAAGAAATATATACAAGAGAGTATCTCTTGGTGCATGATGTTTGCAGATAATATAGTTTTAATAGATGAGACGCGAGGAGTTAAGTTAAAGAGAATGAAGACAAAATACATGCATTACAAGTTCAGTGAAGGCTAAACTAGTGATAGGAAAGGAGTTAGTTTGGATGGAGTGATACTGCCCCAAAGTAATCACTTTAAATATCTCAGCTTAATCCTTCAAGTAGATGGGGGATGTGAAGAGGATGTTAGTCATAGGATTAAAGCCGGATGGTTGAAGTGGAGAAGTGCCATGGGAGTTTTATGTGATCGCAAGATTCCCAATAAGTTGAAAGGAAAATTTTACTGTACAGCCATACGACCGGCCATGTTATATGGTAGTGAGTGTTGGGCATTGAAGGAGTCGTATGCGTCTAATATAAGAGTTGCAGAGATAAGAATGTTAAGGTGGATGAGTAGTCATATTAGACTAGATAAAATCTGTAATGAGAGTATTAGAGAAAAGGTAGGAGTAGTGTCAATTGAGGATAAGTTGAGAGAAGAGAGATTGAGTTGGTTTGATCATGTGAAGCATAGACATACGGAGACTCCAGTTAGACAAGTAGAGTACATTAGGTTAGATGATAGAAAGAAAAGAAGGGGTAGACCTAAATTGACTTCGAGGAGAGTAGTACAGCATGACCTAAAAGTATTACACATTTCTGAAGATTTAACCCAAAAATGTTTAGTATAGAGAAAGAGAATTCATATAGCTGATCCCAAATTATTAGAATAAAAACTGAGTTGAGTTGAGTTGAGTTATGTGTGTTTAATGCATATTGCCTCCATTGACTAAATTTTTGGATACGCCTGGTATACAGGTGTCTATTGTATAATGCATGAGACTCTAAAGCTTGCATTGTTTGCATTTTGATCACCGAATTGAATGGAGATGATTAGGAAAATTAACAAGGGATTTATATAGAAAGAAAGTCTTTATCATATTAATTATTTTTTTTCATCTTTATCATATTAATTATTTTTTTTTCAATTGCTAAACAAATTTTGCAACTACATCGAATCAG**GTATTTGTACGAGAAAGATTAAATGGGCATTATGGAACCACTGCGTTTATTTTTGCCAACACATTCTCTTCCATGCCATTCTTGTTAGTGATTTCACTGATTCCTGGAGCTATAGCTTACTATCTTACTGGACTTCAAAAAGGATTTGACCACTTTCTATGCTTTGCTTCCATAATATTTGCTTCTATGATATTGGTAGAGAGTGTAATGATGGTTGTAGCAAGTATTGTGCCTAATTTTCTGATGGGAATTATAGCTGGTGCTGGTATTCAAGGCCTCATGATCTTAGGTGGTGGATTTTTTCGGTTGCCCAATGATCTTCCAAAGCCATTTTGGAAGTACCCTTTATATTACATTGCCTTCCACAAGTATGCATACCAAGGAATGTTCAAGAATGAGTTTGAAGGGCTAAAATTGCAGAGTAATCAAGCTGCAGGAGGTATTCCTCGCATGATCAATGGTGAAGAAATTCTGAGAGATGTATGGCAAGTGGAAATGGGCTATTCCAAATGGGTTGATGTTGTAATCTTGCTTGGAATGGCAATTTTCTATAGATTTCTGTTCCTGATAATTATCAAGACTTCTGAAACCATCAAGCCTGTTATTATAGCTGCTACGGAAGTGCCTCCTAACGAAACAATTCATGGGGAGGCCTTGTAGAATCATATATGAAATAATTGTAATCTTACTGTTTTGAAGGCAGGAACTTGTTTTATTCATGCTAGGTTGTATGATGCAACTTTAAGAATGGAAATGGAATCTAGTGTTTGTGTATCTTAGCACTGATGATGAACTGAG**

>HbABCG15 scaffold0976(298229-303413)

**GTGAACTTGGTCAATACCACAGTAATTTTTTCACTAGTTCTGTTACCAGATATTCAATATATTGTCTTCTCTTTGTCACCCAGCTTTTTATAACTCTCTTTCTGGGATTGTTTTCGTCTACCCAATTCCAGGTCAAGAATTTTGCCTGGAAGTTATGGAGATAGAGCAGGTTATTAGTTGTCATGGTGATGGTGGTGGTAGTAGTAGTTGTGGGGATGCGGAGGCAGCAGCAGGTTTACCAAGAGGAGGAGAAGAAGTAATAGTAGGAAGATCTATGTACTTGGTATGGGAAGATTTAACAGTGTTGTTGCCAAACTTTAGTGAAGGACCTACAAGGAGATTGCTTAATGGGCTTAATGGGTATGCTGAACCTGGTAAGATCATGGCTATTATGGGTCCTTCTGGTTCTGGGAAATCCACCCTTCTTGATGCATTAGCAG**GTTTTGTCTCTCTCTCTCTCTCTCTCTCTCTCTCTTTCTCTCTCTCTCTCTCACACACATACACACAAACACACACATTCATGGCACCATTAACAATATTAAATACAGTATGGAGTATTTTCCAGATTGTAGAATACTAGCAAGTTACACTCAAAAGTGTGAAAGTACAAATGTTTCCCTTGATTTCTTGATTAACTAGATGTATCATTTTAGTACACATGCATGATTTGATGCCATTTAACAACTCACAATGTTTTCTTCTCCATTCTAAGGAAAAACAGTTCCTTGCTATGCCGGTTGATGAGCAAGAAAGGGAAAGTTCATGTCCTTGAAACCTGAATGATAACCTTAAATCCTTAAACTCTAATTGGTCGTGTTTGTGACTTTATATTTTATTCTTGTGATCCCAGACAGGCCATTTACGATCACGAATTGACGTCCCTTAGAATATCTTCTACCTCATGGCCAATGCCTTTGTTGGTTCCTCACAAGTGATCTGCAGTTGCTGACATTATTGTAAAATTGCATTGTCTTCGCAACTTGAGCAGATAGAGGATTTAATTTATTGCATTTCCCACACTAAACCATATTTGAGTTGGGATTAGTGAAATGGGTCCAGCTTACCCTTGCCTCCTGCTTCTAACTTACAAGGATATGCTTTGATGCATAGTTTTGTTAACCTTTTCTAGGTGTTAAACTAAGTTCAGGGTTTTCAAGTTTTTGGAGAAAAGTATCATTTGCTCTAAGAATATGGGGTAAGAAATGGAATTGGAAGCCTCTGATTCCCAGTTTTCCCACTGAAGTAGAAACTCTAAATAATTTTAAATTTTTAATTAAAGTGGTCAAAAAAAGAATTTTGCTTTTTTTATTATTATTATTATTATTATTATTATTTTTATCGTGCTTTCTTCTTTCTTTTCATTAGTGTTATTCAATTCAATCAATTAAAGTGTGAGAGAGCATTCTAATAACAACTTAAATGTTTATTCCTACCACCCACCAGCCTTCCTTTATAAACTCATTAATGCGTCTTCGCCTGCAACCCTTACATATATCGGACATTTTTTTTGGGACACTGTCTACTGATAAAATGATTTAGACTGATGAGGCCCTGTGACTGAAATGTGAAACAATACAATATAAGTTATCCTTATATATTTAGCAATATTTAATTATATCTTCTGTGGGAGTTGGGATAATTAATTTTACCGGTTGTAAGATTATGACGCATGTCCTTCACGCATCAACTCCATCATGTTCATTGGACGAGTCACTGTCCTACTAAATTTTCCTTTTCCCCCCTCTTTTTGGACCGCATCGATCTGTCAGTCCAAGTACTGTTAGTCAACGTTCATTGATTCCTGGCTTGCCTTTGAAAATTCAATCACCAAAACCAGATTTTTTTACTCATATGCTTTCTTCATATGGCAAAACAGAAGGTTTTATAAGCTTCCCTTTTTTTTTTTCTTTACAGTTAACTCATTGGTGTAAACTAATTTTTATGATAGGACCTGATATATGATACTTTATGTGATAGAATGGAATATTTAATGCAAAATGTCATTGAAAATGCTGCAG**GTAGACTCTCAGGAAATGTTATCATGACTGGAAATGTTCTGGTTAATGGGAAGAAGAGGAAACTTGGCTATGGTGGTGTC**GTAAGTTATTTAAATTATTTTGTCATTACACTTCAATTCCAAGAAGGAATTCTATGTAGTGAAATAATAATCCCTATAAAAATTTTAATTCAG**GCTTATGTAACACAAGAAAATACGTTGCTGGGAACTCTCACTGTTAGAGAAACATTAACTTACTCGGCTCATTTAAGGCTTCCAGGCAGCATGACCAGAGAAGAGATTGAAGGCATTGTACAAGGAACAATCATGGAAATGGGTCTCCAAGATTGCGCGGATCGGCTAATTGGAAATTGGCATTTGAGAGGAATAAGTGGTGGGGAGAAGAAAAGGCTAAGCATTGCACTTGAAATCCTTATTAGGCCACAACTTTTATTTCTTGATGAACCGACCAGTGGACTAGACAGTGCCTCAGCTTTCTTTGTTATTCAAACTCTCAAAAACATAGCTCACGATGGAAGAACAGTTATCTCTTCAATTCACCAGCCAAGTAGTGAAGTTTTTGCTCTCTTTGATGACCTTTTCCTGTTATCTAATGGTGAAGTTGTTTATTTCGGAGAAGCAAAGATGGCAGTAGAG**GTTACAATTGTTTTTAACTCTGTTATTTATGTTACTTCCATTCTCACCCCACTCTAACCTCAAGGCTAATCTTGCAG**TTTTTCGCTGAAGCTGGATTCCCATGTCCGAGTAGAAGAAATCCTTCAGATCATTTTCTACGTTGTATTAATTCAGACTTTGATCATGTCACTGCAACTTTGATGGGATCTCAAAGA**GTATGTGCAATGCAGTTTCCACTAATACTCTTTAATCAGTTTCTTCTTTAACATAAAATTTGTTTTACGATTTTGTAG**TCTAGTCCTCGACACCAGGAAATTCAAATATCATCAGATTCTTTGGCAAATTTGCCAACAGCAGAGATCAAGGCAGTGCTAGTTAAGAAATACAGGTTCTCCAACTATGCAGCAAGGGCAAAAGCTAGGATTAGAGAAATCTCGGCCACT**GTGAGCGAATTTCAATTTTAGATCCCTTGAAATGATATTCAAACCAAATTGTTCATTTTGGACTGAAATTCACCAATTTGAACACTTTTATAAGGGTAAATTTTGTACTGCAG**AAAGGGATTGAGGCTAAGAGGAAAAGGGAAAGCCAGGCAAACTGGAGCAAGCAGCTTTCAATATTGACACAAAGATCATTCACCAACATGTGGAGAGATTTGGGGTATTACTGGGTAAGGATAGGTATCTACATAGCTTTGTCTATTTGCGTTGGCACCATCTTTTTCGATGTTGGAAGGAGCTACACTGCAATTTTGGCTCGCGGAGCTTGTGGAGGATTCATATCAGGATTTATGACATTCATGTCCATTGGAGGCTTCCCTTCCTTTATTGAAGAACTTAAG**GTAAATGATTTGTTCTGTTCTGACTAGGGGTGAGCATTATTCGATTCAAACCGAATAAATTGAATCAAATCACCTTAATTCGATAATTCAGTTCGGTTAAACCGAATCAAATTGAATATTTTTATTGATTTTTGAATTGATATATTTTTATGGAGAATTTATGAATTATATGTAATTATATATATATAAATTATTTAATTTCATTGATTAATAGTTATTAGGTTCAAACCAAGGTTAAAATCAAATCAAATAACTAGAAAATCTAATCCAAATTAAAAAATCAATAAAAAATCAAAACTGATTGATTTGAACCAAATCAAACCAAAATAGAGCGATTAGATTTTTCATCCATTTTTAATTCGATTCGATTTCTAAAATTTGTAATTCAATTGTCATGATTTAATTCAGTTCGATTCGATTCGATTCGGTTCAAACCAAATGCTCACCCTAGTACTTCTGACGGGAAATACTTAATTGCCTATACCTGATTCAATATCGGTGTTAAAAATAATTACTTCCATTGTTTGTCTTGTGCTGTTTGAGCAG**GTCTTTTATAAAGAAAGACTCAATGGGCATTATGGGGTTGGAATATATATTCTGTCAAACTTCCTCTCTTCCTTCCCATACTTAACTGTGATGTCAGTGGCTACTGTGAGTATTACCTTTTACATGGTAAAATTTAGACCTGAGTTTTCGCATTTTGTGTATGCCTGCCTTGACCTTACAAGCAGCATAGCGGCAGTTGAAAGCTGCATGATGACCATAGCTTCGCTTGTTCCAAACTTCTTAATGGGAGTAATTGTTGGAGCCGGATATATT**GTAAGAACAAACGTTTATATATGCCTTTAATGCGTTATCATCCACCCTCCAAAAAAAAAAAACTATTATTTATTTGATGTTTGCTTCCAG**GGAATCCTGATGATGACCTCTGGGTTCTTTCGTTTGCTGCCTGAACTTCCCAAGGTCTTTTGGCGTTACCCTGTTTCTTATATCAATTATGGAGCATGGGGATTGCAG**GTAAAAGAGAACACAGCATAACATAGCAAACACCATGTCCCAGCACAGAATAAAACAGCGACAAAATTCATGCACATGTACTATTATCAATCTAATGTTTTAACTGCTGGTATAACGGAAATGCAG**GGTGCATACAAGAACGACATGATTGGGATTGAGTTTGATCCTTTAGTACCAGGTAACCCAAAACTGAAAGGCGAATTTGTTCTTAGTACCATGCTTGGTATTGATCTTCATCATTCAAAATGGTGGGACCTGGTGGCTGTTGTAGTCATTCTAATAGCTTTCAGACTTCTCTTCTATGCTATTCTCAAGTTCAAGGAGAGAACTATGCCCGTGTTTCATAAGCTCTACGCCCAGAGAACTATAAAACATCTCAAAAAGAGGCCATCCTTCAGGAACAGTTCACCCTTCCCTTCTTTCAGGAAAAGTTCACCTTTCCCTTCTACGAGGCACCAGACTCTACATTCTTTGTCTTCTCAAGAGGGTCTCAACTCTCCGATCCACTAGCAGCAGCACCAAATTAAACATATAGCAGTTATAGTCAAACTTCTTCTGTGATCTTGGAAAAATACTGGGTAAGGAGATAAGAAAGGAAGGTATAAATATGAAATTCAAAACTTCCTTCAAAGAATACTTATTGAATGTGTGCAAATATATGTTCGGGTATCTGATGACCACAGGCGGTCCATGG**

>HbABCG20 scaffold0029(3070096-3072473)

**ATCCTTAGCAAAAGAGTGTTGCAACTAAACACCAACAATAAAAACATTCATAGATGTCTCATGTAGCACTTTCTAAAGTGTCACAGCAGGGAAAAGGCTCTACCGGTGGTGACAACCTTCTGTAGCCCTACCCACCAAATGGAGCTCCAAAAGTACCATAGGACTTCCGAACCCTCCGTCTCTATCACCCTTTCTGAGCTCATAAAACGCGTCGAAGATGCTCAAAGTGACCATTCCAATGGCAGCACTCCTATCCGTCATCATGCCCTGGAGCTTGGCTATGCTTGTAGCTCTGTGTCCCCTTCAAACCCTTTTGTTCTTTCTTTTAATAACTTATCATACAGTGTCAAAGTTGGCCAAAAATTGACTTTTCCATTCTGTGGAAACGATTCAGATGACTCTCCTGAGACTGGCATCAAGGTTTTATTGAATGATATATCTGGAGAAGCCAGGGAAGGGGAAATCATGGCCGTTCTTGGGGCTAGTGGATCAGGTAAATCAACGTTGATCGATGCACTTGCAGACCGGATTTCGAAAGAAAGCTTGAAAGGGTCTGTGAGTTTGAACGGTGAGGTCTTGGAGTCAAGGCTTTTGAAAGTTATATCTGCTTATGTTATGCAAGATGATCTCTTGTTTCCGATGTTAACCATTGAAGAGACGCTAATGTTCTCTGCTGAGTTCAGGCTTCCACGCTCCCTCTCCAAGTCCAAAAAGAAAGCCAGAGTTGAGGCCTTGATCGATCAATTAGGCCTACGCAGCGCTGCGAAGACTGTGATTGGAGATGAAGGGCACAGAGGCGTTTCTGGAGGTGAAAGAAGGCGAGTCTCTATTGGGATTGACATAGTTCACGACCCAATTCTTTTGTTTCTTGATGAGCCAACCTCTGGACTCGACTCCACCAGTGCTTTTATGGTTGTTAAGGTGTTGCAGCGAATTGCCCGAAGTGGTAGCATTGTGATCATGTCCATCCACCAGCCCAGTTACCGAATCCTGAGCTTACTTGACCGTTTGATCTTCCTTTCACATGGTCAAACGGTGTACGCTGGCCCACCAGGTAGTCTTCCAGAATTCTTCGCAGAGTTTGGGCATCCAATCCCAGAGAATGAAAACCGAACTGAGTTTGCTCTGGACTTGATTCGAGAGCTTGAAGAAATTCCAGACGGAACCAGGACATTAGTTGAATTCAACAGGTCATGGCAAGCACAGGGCAAAAAGAATCCAAGAAATCGTATCAGCAATTCATCAAACCTTTCCCTTAAAGATGCAATAAGCGCAAGCATTTCCAAAGGGAAACTTGTCTCTGGCGCCACGAATGATTCCAACTTGTCATCTTCAGTCCCAACATTTGCCAATCCATTTTGGATTGAAATGTTAGTGATAGCTAAAAGATCACTCACAAACTCTAGAAGAATGCCTGAATTGTTTGGAATTCGCTTCGGTGCTGTTTTCATTACAGGTGTGATATTGGCTACAATTTTTTGGCATCTGGACAACTCTCCAAGAGGTGCCCAAGAAAGATTGGGCTTTTTCGCTTTTGCCATGTCCACAACTTACTATACATGTGCGGAATCCATCCCTGCCTTTCTCCAGGAACGTTACATTTTCATGAGAGAAACTGCCTACAATGCTTATCGCCGTTCTTCCTACGTTCTTGCTCACTCCCTAATTTCCATTCCTTCTCTAATTATACTCTCTATTGCCTTCGCAGCTACGACCTACTGGACAGTAGGGCTTGCGGGTGGAGCTTCTGGGTTCTTTTTCTTCTTCTTCACAATCTTATCTGCATTCTGGGCAGGAAGTTCATTCGTTACATTCCTCTCAGGCATCGTCTCTCACGTAATGCTAGGTTTCACAATTGTTGTTGCCATTTTGGCTTACTTCCTTCTCTTCAGTGGATTCTTCATTTCTCGGGATCGAATTCCTCCCTACTGGATATGGTTTCACTATCTCTCTCTGGTGAAATATCCATATGAAGGAGCTCTACAAAATGAGTTCCAGGATCCCACAAAATGCTTTGTAAGGGGCGTGCAAATGTTTGATAACACACCGCTGAGCGCTGTGCCTGTGGCGCTGAAACTCAAATTGTTGCAGAGCCTGAGCAACACCTTGGGCAGGAACGTAACAGGCAGCACTTGTATAGTCACTGGACCAGATATACTAAGGGGACAAGGTATTACAGATATAAGCAAATGGAGTTGTTTATGGATCACCATTGCTTGGGGATTTTTCTTTAGGGTCTTGTTTTACTTTACTTTGTTGTTGGGAAGCAAGAACAAGAGGAGGTGAATAAAGATTTTTTTTTTTTTTTTCTTTCTGAAATTAAACTGAGTTTCCTGGTAATTGTAGATTTTAAGTTTATCATAAGCTTGAGATGGCG**

>HbABCG21 scaffold2550(4316-9632)

**TGATATGATGCCCCCTGAGCAAGAGAGTTCCATTGCTGCAGGCAATAGTCCAGCTAATATCATGCTCACCAACTGGACGGAAACTGTTCCTGTTCATGCTGAGCCCTCTGTTTCTTCCATTAACGCCACCCCATGTTCACAAGATCGACTACCTGATCAACAAGAACCAACCCCATCAAGATTCTCTATTTTACGCGCGTCGTTACGTCCCGTAACTCTCATG**GTAATTAACAATCTACCTTAAGCAAGCACATATACCAACTATATATATATATATATATATATATATGAATAAACTTTTTTAATTTGGTGTTGCAG**TTTGTAGATGTTGCTTATAGTATTGATTTGTCCACAAAAGGAAGCTGTTGTTCTCCGAATGGATCAAAATCGACAAGAATCGTACTCAATGGTGTTAGTGGCATCGTTCGTCCTGGCGAGCTACTAGCAATGCTTGGACCATCGGGGAGCGGCAAGACTACCCTTTTAACAGCTCTTGCCGGTAGACTACCAGGAAAGGTCTCGGGTACCATAACATACAATGGCCTGCCCTTCTCCAGCTCTGTGAAGCGCAAAACAGGCTTTGTCGCTCAAGACGATGTCTTTTATCCCCATATAACAGTGATAGAGACCTTGACATTTGCTGCTCTATTAAGGCTACCAAAAATGCTCACCAGAGAAGAGAAAATAGAGCAAGCTGAGATGGTTATCGTGGAGCTTGGATTAACAAGGTGTAGAAACAGTGTAGTGGGTGGGCCTTTACTTCGGGGAATATCGGGTGGTGAACGCAAACGGGTCAGTATCGGGCAAGAAATGCTGGTAAACCCGAGTTTGTTGTTGCTTGATGAACCCACTTCAGGGCTTGACTCGACCACTGCTCAACGAATCATGGCCACCTTAAAAGGACTAGCACGTGGCGGTAGGACTCTCATAACCACCATTCATCAGCCTTCAAGTAGGCTTTACATGATGTTTGACAAGGTGGTGGTTTTGTCGGATGGATGTCCCATTTACAGCGGGCCAGCTGGTCGGGTCATGGAGTGTTTTGATTCGATAGGTTACGTGCCCGGGTTCAATTTTATGAACCCGGCTGATTTTCTGCTTGACCTTGCTAGTG**GTAAGTGATACTTCCCTTAATTATTAATCAATGTATGTTTCTTGCAAAAAATAAATAAAATAAATCAGTGTATATGTTAATTGAAGCTTGAAATAATATGTTTTTAAATGTATTAGTTAAAACTATTAAAATTTAGTTTAAAATTAAATTTCTTATGTTTTAAAATATTAAAATCTATTTGATATTATTGTTGAAATTAAGAGAAGATAATTATAGAATATTGTATTAATATTTAAAATTACAATATTTTTGTGTTTATTTCAAAAAGGAAAAGTAATCTTAAATTTTTAATATCAAAATATCAATAAAAATAACTGTTTTAACAATAAAAAAATATTAATATCAAAAATATATTATATTACTTTTTACATATTTCTTAATTATATAGTTTTATTAAAAATACATATATAATTAAAAATAATAAAATATATCAGATGAGTAAAATGTGATTTAGTCATATAATTAATTAATGGCTATAAGATATTTTAATATATTTATAATTAAAATTATTTAAAAAATAATATTAAATATTATATATACACTATAATTAAGAATAAATATTATATATACACTTATAATTTAAATTAGATTATATATATATATAATTGGTTCTTAATTTGATTTTAGTTTGGCTCTTAAGAGGTATCAATATTGAACCAAAATAATAAACATAAATTCGGTTTAGTATAGCTCGGTTCTTATAGCAATGATTCTTTTTTAGTTCTATTGTATATGCTTGGTGTGATTTTTCAGTTTGGAGTATCAGAAATGGTGCATAACCCTATTATTAATAATTTTTAACAAAGATAGTTGAATTATTATCATCTAGAATATAGTAACTCACCGTATACGGTTTTAGTTCTGAATTAGAATAAAATCAGTTTAATTTAGAATTCAAATCAGTCAAAATTGGATCGACTAAAACTAACTCTGAATTGGATTGGAATTGTTTGTTCTGGTCTGATCTGTTCAGTTTCAGATCAAAATTGGATTTTTTTAAAGGAAAATTTTAAAATATTTCAAGGTTAGTGTAATCAAAATCAGATCTCGTCATAACTGAGAGTGATCCAGAATTAGAATTAGAAGAGACGTTGCAATCCAATCCTTTCCCCTAAACCTTGAATCGAAACAGCCCAACCCAGACCGAGCACCCATACCGGCAATAGTGAACATGGAAAATCTGAAGATTTTTACGTCAGCTCTTAGAACTTTCGACTTTGGATGTCATATCCAGTGAAAAAAAAAATATGGAACTTCCCTCATCTTTATCTTTTGGTCTGTCATGGTTAATTACAATTTCTAGTTATGCTCTTAATTAATTACATCAAATAGGTAAGATTAACTCTTTTAATGCAAATTTCAAATAACATAAGATTTTCTCGCTAATTCTAGTATATTTTCTTTAATTTTCAAGTCTTTTTGGAGATGCAGGCATAGTTCCTGACAGGAAACAAGATGATAAAATGGAGGTTTATGGTAGAGTAGATCACCTTGATGATCAGAATTCAACAAAACAATCCTTGATATCATCATATAAGAAGAACTTATACCCTGCATTGAAAGCAGAGATTCATCAAAATTTGCAGGACCCAGCTCTTTCTGCATCGTCAGGAACTTCATCACTGAGAAGTATACGAAAAACTCTCCGCCTAAATAATTACTTGGTGTTGTTATCAACTGCAGTAGTTATATAGAACCTGTATTGATCATCTGCTAGTTTTTGGCAGATTCTGAGGATCAATGGACGACCACCTGGTGGCAGCAATTCAAGGTGCTGCTGAGAAGGGGTTTGCAAGAAAGGAAGCATGAATCCTACTCAGGCTTAAGGATTTTCCAAGTCATGTCAGTTTCGATTCTTTCAGGGCTTTTGTGGTTGCATTCTGATACGTCACACATACAAGATCAGGTATCTTTACCCTGCAGTCTGGATTATGCCTTCCAAGCTTTTAGTGAGAGTTATAAATATATAATATACTATACTATTGGTCTCTGTGATATTGTTTTTTATGACAAAAAATTTGGAACAGGTGGGACTTCTCTTCTTTTTATCCATCTTCTGGGGCTTTTTCCCTATGTTCAATGCTATATTCACATTCCCTCAGGAGCGACCAATGCTGATAAAAGAGCGTTCCTCGGGCATGTACCGCCTCTCCTCTTACTACTTTGCTCGAATGGCTGGTGACTTGCCAATGGAAACTAACTCTGAATTGGATTGGAATTGTTTGTTCTGGTCTGATCTGTTCAGTTTCAGATCAAAATTGGATTTTTTTAAAGAAAATTTTAAAAATATTTCAACGTTAGTGTAATCAAAATCAGATCTCGTCATAACTGAGAGTGATCCAGAATTAGAATTAGAAGAGACGTTGCAATCCAATCCTTTCCCCTAAACCTTGAATCGAAACAGCCCAACCCAGACCGAGCACCCCTACCGGCAATAGTGAACATGGAAAATCTGAAGATTTTTACGTCAGCTCTTAGAACTTTCGACTTTGGATGTCATATCCAGTGAAAAAAAAATATGGAACTTCCCTCATCCTTATCTTTTGGTCTGTCATGGTTAATTACAATTTCAAGTTATGCTCTTAATTAATTACATCAAATAGGTAAGATTAACTCTTTTAATGCAAATTTCAAATAACATAAGATTTTCTCGCTAATTCTAGTGTATTTTCTTTAATTTTCAAGTCTTTTTGGAGATGCAG**GCATAGTTCCTGACACGAAACAAGATGATAAAATGGGGGTTTATGGTAGAGTAGATCACCTTGATGATCAGAATTCAACAAAACAATCCTTGATATCATCATATAAGAAGAACTTATACCCTGCATTGAAAGCAGAGATACATCAAAATTTGCAGGACCCAGCTCTTTCTGCATCGTCAGGAACTTCATCACTGAGAA**GTATACGAAAAACTCTCCGCCTAAATAATTACTTGGTGTTGTTATCAACTGCAGTAGTTATATAGAACCTGTATTGATCATCTGCTAGTTTTTGGCAG**ATTCTGAGGATCAATGGACGACCACCTGGTGGCAGCAATTCAAGGTGCTGCTGAGAAGGGGTTTGCAAGAAAGGAAGCATGAATCCTACTCAGGCTTAAGGATTTTCCAAGTCATGTCAGTTTCGATTCTTTCAGGGCTTTTGTGGTTGCATTCTGATACGTCACACATACAAGATCAG**GTATCTTTACCCTGCAGTCTGAATTATGCCTTCCAAGCTTTTAGTGAGAGTTATAAATATATAATATACTATACTGTTGGTCTCTGTGATATTGTTTTTGATGACAAAACATTTGGAACAG**GTGGGACTTCTCTTCTTTTTATCCATCTTCTGGGGCTTTTTCCCTATGTTCAATGCTATATTCACATTCCCTCAGGAGCGACCAATGTTGATAAAAGAGCGTTCCTCGGGCATGTACCGCCTCTCCTCTTACTACTTTGCTCGAATGGCTGGTGACTTGCCAATGGAGCTTGTTCTGCCCACAATTTTTGTGATTTTCACATACTGGATGGGTGGCCTGAAGTCCTCTGTAATCACATTTGTATTAACCCTCTTGATCATCCTTTTCAATGTGCTAGTATCACAAGGACTAGGACTAGCTCTTGGTGCGATTTTAATGGAGGCAAAGCAGGCAACAACTTTAGCTTCAGTGACTATGCTTGTGTTCTTACTAGCCGGGGGATACTACATTCAGCATATCCCACCATTTATAGCCTGGTTGAAGTACATCTCATTTAGTCACTTTTGTTACAAGCTGCTGGTAGGAGTACAGTACTCAGCAAATGAGGTTTATGAATGTGAGCTGGGGATGGATTGCAGGGTAATGGATTTTCCTGCTATCAAGTGTCTGGGTACTGATAATAAGGGGTGGGATGTGGCTGCTCTGTCAATAATGTTGGTCGGATATAGGCTTCTGGCTTATGTGGCTCTGAGAATGTGGCAACCTCACTGACCCATATGATTAAGATTACTCATTATGACCATCTCTTTATTTGAGCAACAGGAACTAAGGAAATTGTTATCCAATTTTGGTCATTATCATCTTCAAGGGAAGAAATCCTGATGAAAGCTAGCTTGCATAAATGTAAGTTGGATTTGTTGCTAAAATACATTAAACTTGTGACCCGATAGTAGACAAATAATGATTAAAAGAGTATCTCTTTTATATGTTTGTACAGATCATAAATCCAAAATTACGAAGGGGA**

>HbABCG22 scaffold1583(33032-39288)

**GCAACTTTTATCTTCTCCTTAGTTCGTTATAGCTATTGTGATCCTTCACCTTTTTGATTAACAGAAAACAAAATACAAAAGTAAAGAAGCAAAAG**GTATGACTGATCTGATCATCATCTTCTAACTGATTGTCGGTGATGAATTTTTCTCTGTTTCTTTTTCTTTTGTCCCTCTTATTTTGCTTCAGTGATCTTTCTTTCTTTCTTTCTTTCTTTCTTTGTCTTGTTATCTTGCCAAGAAGAGAGAAAGGGATATTGGATTTTGCTCAACAAGCATGTTTCTGTCTTTCTTCTCCATTTTCCATTTCAAGATGCTGTTTCTTGTTACTTTGAAGCCCTTCTGCTAGTTAAATATTCAGTAACTTCTTAGTTCATTTTAATGATATTACAAATATATGCCATATAATAACCTTTTTCTTCTTTTCTTTTTAAACTTGGCTAGTTCATCATCCAACAGATGGAGATGGTGTGGCAACCTCCAAAACCAGTCATTTATAAATAATTTTTTTTTGAAATTGCAATTTATGATATAGAAAATCATGAGTTTGTATATATTCTCGCTCTACGACAGAAGAACAGTCTTTTTTCTTTACTTTTGGGTTCTTAATACGTTGATGAAGTCTCAGGTTTATATTTAATGATTTAATTTCATTTTTCATGCAG**AAGATTGTAGCATGGAGAAAACAAGCGCTTCCAGTTTAGTGAGAACAAAATCGGACCAACCAGTGGAGACGGTGGCGGCGGAGTTTAAATCTCCCCCGACCAATGAGGTGGGTGGAGGAGTGTCGGAAGGAAGTGGGACATTGTCGAGGAAGTCCAGCAAGCGGCAGATTATGGCGGCATCACCAGGGCGGAGCGGTGGAGGTGGCAAGAACACACACATAAGGAAGTCATGGAGTGCACAGATGAAGTTTGACTTGGATGACGTAAGCAGCGGTGCAGCTTTAAGCCGAGCCTCTAGTGCCAGCTTGGGCTTGTCTTTCTCCTTCACCGGCTTCACGGTGCCGCCGGACGAGATCGCGGACTCAAAGCCGTTTAGCGATGATGATATAC**GTAAGTAATTTGCATATTTCATGCAACTATACATTATATAGTATAATTTGAGTTTCTAGTACTAATTTGAAGCAAATCATTATTCAAAAATAAAAGATAGAAATATAATATTAAAAAATTAATGATAAAAATTAAAAAAATATAATATAATTTAATAATATATTTGGGTTTTTATTGACAAAAGTAAAGGTATAATGATTGGAAAACAATGAGCAATTGGGAAGGTAGCTAGTTAAGTTTACCAACTTTCTGATGGAAGGAATACACCAAAAAATAACAAGAACAGAAACAGCCAGTTAATTACTTTAAAAAGGAAATGAAGACATATTTGCATTTGGCAATTGACAACCATTTGCAAAAATGTAAAAAGTGGGCCCTTCTTTTCCATCTTTGATCAAGTGCTCAGTTTAAAAAAAATTATTTTATTAATATTTTATGATTAAAATTTATTAAATTTAATTTTAAATTTTTTTAATAAATATATTTTAAAAATACATTTTTCAAGCAATGTTAAATACATCCTAAATTTTCAATCTGTAACACCTGTTGCATTTTAATTTTGCCCATAATTATTTAAAAAAAAAATTCTTTATATAATAATATATAAAAACTGTTGGTGTCTTTTATTGCCCATTTTATTGTAATTTGGAATTAGTGTGTAGGTAACCAAACAAAAATTGAAAATCCTAATTACTGAATTTATATTTTTATTTAATTTTATAATTGAAAATTCATGCACACTTATACTAACACATTTATATAAATTAAAAAATTTTAAATATATATATAAAAAAAAGTGTTTTGGTGTTCTTATAAAAAAAAAAGCTCTTGTTAAAAATATTAGACAAAATAAAATATAATATAAAAAAAATAAATTTTCTAACAAAAATTTTTACCAAAATTGATTCAAGATATATTATAAATCATGAAACTTGAAAGAAATTATTAGGTGCAATTATTATATGTATAACAATCAATCTTGACTGTTGATTATAATGAGACTCACATAAAACTCATCTTAATTAACGATTATGATACAACTATTGTGCATACAATTGTTTCAACTATTAGTATTCTAAAATTTTTCAATGTGTAGAGAAGTTTTTCCCATAAGTCCGGCATGAATTTTGACTAGAGATATATATTGCAAGAAACGACAATTAAGTCATACAAATGGGATCCTAATTTCAAATGTAGGTGAAGCTACCTTTAAAGTGGAAGACAAATAATGGGGAATATTGGACCCCAACCCTCTTCAACAGTCAAACTGGCTTGATTTCTTTGTTAATTTCTTGGTCGCTAGTAGCTAGGGTGTAACTTCATTAGGTTTTTGATTATTTCTCCAATCGTCTTGGCTAAACTCACTCACCTAAATCAATTATGAATTATTTGAAAATGGATTTCATCACTTTTGTTTCTTCTCTTTCGTTTTCTTTCTTGTGACAACGATTTGAATCCTTATAATGACGCTACAGCACTGTTATATATGACTGATTGATCATTATTTGTGGGGAAACTTGAATTTGCAACCATGTATTTGTAAAGAAATATCATTCCGGAAAAAAAAAAAATCAATTACCTGGTCATTCAATTATCAGATTATTGACAGCATTGTATCCTGATCGTTAGCTAGCTAGTTTTTCTTTATATACCAAAGTTTTAATTCATTGCATAGTTATTTGTTTGCCCAAAAAGGATATACACACATATGTCTATGGCTGTTGCAG**CTGAGGATCTTGAAGCTGGGACGAGGAAACCGAAGTTTCAAACAGAACCCACCTTGCCGATTTTTCTCAAG**GTATATAATATAATAGCTATCTCATTGCCTGGCATGCAGGCCATTATATATAGATATGGCTGTATCTTCTTCTTCATTGTCCAAGAGCATGCACTGGTATCTTCAATGTGGTATGGTGCCTTGGATTGATTTCATATACATATTCTATTTGGACAGGCAAAGAAGGAGACTAATGTCTACGCGATCCATAGAACATGGCTAGCATGGTCAGGTTCAGTCTTGTACAGACAGTGTTTCATGATCTAGTATCACATTTATATTAATTTTGTACAAGACTCTCCCTTCTTTCCCAAAACAAATGGAATTAATATCATCAACTGCCTTAAATTCTTCTAACTTGCATGAAATAATAATGAATCTTAATTACCTTCCATATGCATCTTTGATTAGTCTTTAAGAGTACTCCCATAAGTAATGCTTTGCTATGGAAACAAGGGTGTGGACAGAAAGTTTTTTATTTATTTATTTTTTTTATATGTTGGGGAGGGGGGAGTCAAACCTGAGTCTCCAGGTTCAAGTATATATAGATACTCTGGACTATGCCCACTGCGGAAAGTTTATTAAGCCTTCAATTGTTAGTTACTACATAACTAGAATATTATGAAGAATGCAGTTAATTGCCACAAATTTACTGATCAGTTAAATATTTGTTCTTGATTTTGGAATATATTCTGCAG**TTCACAGATGTCACTTACAAGTTGATTATCAAGGGGATGACATCGACGGAGGAGAAGTATATCTTGAATGGAATCACTGGTTCAGTGAATCCAGGGCAAGTCTTGGCACTTATGGGACCTTCAGGAAGTGGAAAGACAACACTCTTGAATCTGCTTGGTGGCAGGTTAATTCAGCCCGGTGTTGGTGGTTCAATCACTTATAATGACCAACCATATTCCAAGTTTCTAAAAAGTAG**GTAAGCATTTCAATCTTACAATGGAGGACGTTTGTTCAACGGAAATTGCTTGGTTGATCACTTTTTTTTTCCCTGTGGACCATATATGACTTGGAAATAATCTGCAG**GATAGGATTTGTGACTCAAGACGACGTTTTGTTTCCTCACCTTACAGTGAAAGAAACATTAACATATGCAGCTCTCCTCCGATTGCCGAAGACATTGACAAAAGAACAGAAGGAAAAACGAGCCATAGATGTCATCTATGAGCTAGGCTTGGAGAG**GTATAAATATATAATCACAAATTGGTATTCTTGCTCATTTCATTCTTGCTAAATGAGAATCTTGATCTGCTGCATGCAG**GTGCCAAGACACTATGATTGGAGGCTCATTCGTCCGTGGCGTTTCAGGTGGGGAAAGGAAAAGAGTGTGTATTGGAAATGAGATTATAATCAACCCTTCTGTTTTGTTTCTTGATGAACCAACGTCTGGCTTGGATTCCACAACTGCTTTAAGGATAGTTCAGATGTTGCAGGATATAGCAGAG**GTATACATATGTACAATTAATGAAGTTCCTTTTCCTTTCTGATCTTCGCAATATTAACATAAAATCTGATGGCTGCAG**GCAGGGAAAACAGTGATAACAACAATCCACCAGCCATCAAGTAGACTCTTCCACAAGTTTGACAAGTTGATCCTTCTTGGGAAAGGGAGCTTGCTCTATTTTGGAAAAGCATCAGAAGCAATGCCCTACTTCTCATCTGTTGGTTGTAACCCACTTATTGCCATGAATCCAGCTGAGTTCTTGCTAGAACTTGCAAATGGAAACATAAATGATGTTTCTGTACCATCAGAATTGGAGGATAGAGTGCAAATGGAGAATTCAGACAACGAAACAAGAAATGGGAAGCCATCTCCTGCAGTTGTACATGAT**GTACTTAAAGTACTCTTATACACACAGACACACACACACACACTCTTAATTAGCTAGCTGCTCCTAAATTTGCAGAAACAATCTACTAGTCAACAAGATAATGGTTTCATATCTGTATCCTGCAG**TATCTTGTGGAAGCCTACGAGACACGAGTTGCAGAAAATGAGAAGAAGAAACTTATGGTTCCTATACCCCTTGATGAGGAAGTGAAGTTGAAAGTGTCTTCTCCGAAGCGACAATGGGGAGCAAGCTGGTGGGAACAATATACCTTATTATTCTGTAGAGGAATCAAAGAAAGGAGACATGACTATTTTAGCTGGTTGAGAATAACCCAGGTTCTTTCCACTGCTATTATCTTGGGATTACTATGGTGGCAATCAGAGAGTAGCAGCCCCAAAGGCCTGCAAGATCAG**GTAATCAATTAAGACACTGTATGATTCTATGGATACATGAACAATCAACCCCAAGAACTTGAAGGAACTTAACCCTTGTTTAATTGCAG**GCAGGGCTGCTTTTCTTCATTGCTGTTTTCTGGGGATTCTTTCCAGTCTTCACTGCCATCTTTACATTCCCTCAAGAAAGAGCTATGCTTAATAAGGAACGTGCAGCAGACATGTATAGACTGAGTGCTTATTTCTTGGCAAGGACTACAAGCGATCTTCCACTTGATCTGCTACTGCCAGTACTGTTCCTTGTCGTTGTCTATTTCATGGCAGGCTTGAGAATGAGTGCTGGTCCCTTTTTCCTCAGCCTGCTAACTGTTTTCCTCTGTATTGTTGCTGCTCAG**GTAAACCTTTCTGGGAACAGCAATAAAGAAAGTGAAATATATACCCAAACATTCAAACCTGATTAGTGTATTTAACCAAGGATGGCTTTAATGGCAGCAG**GGACTTGGACTAGCTATTGGAGCTACATTAATGGACTTGAAGAAGGCTACAACACTTGCTTCAGTAACTGTGATGACCTTTATGCTGGCTGGAGGATACTTTGTGAAG**GTAAAAACATGGACAATCTGATTATAGTTCATAATCTACATTAATTATTCAAATTACCCTGTTATATAATCAATAATTTGAATGAATTTTCTTATAATAATTCGATTATAG**ATTCGCAACACCCGGTGCATTTCACCAGATCCCATTTGCACAATAGGGAAAATTACTCAACATCCAATATTGAAGTTTCATCAGAGCATAAAATGGTCAAGGGTGAAGTGTATTCTTTCAGGGAAAAAGGGAAACACTATCTCTCCCAAAATAAATGCTGACTCCACATTTTCTCAAGCTTGGATTGGCATTCAGTTATAGAAAATGTAATAGTTAATACATAGTCTTCTCTTCCTTATGCAGAAAGTTCCAATATTCGTTGCTTGGATCCGCTATATGTCTTTCAACTATCACACTTACAAACTTCTTCTCAAGGTGCAATACGAGCATATGTCGCCCCCCATCAAAGGAATGAGAATGGACAATGGTTTAATGGAAGTAAGTGCCTTGGTGGCCATGGTTTTTGGCTACCGTCTCCTGGCATACATTTCTTTACGGAAGATGAAGCTTAATTGAGGATCCTAATAGCCTGAA**

>HbABCG28 scaffold1381(113619-121886)

**TATATCTTTTTGTCACCTGAAACCAGAAGCTTGAATTATGATTCGTGCTTTATAATTATATATTACTAGGTCATCATTTATACAAAACAGAAGAAGAAATGTTACTTTATGTAGTTTCTCCAGTCTACCATTTAGTTTACTACTTTATCGCTTCGTATATCCCAAGAATCAAGTTGGAATGTTGCTTGATTGATGTGTATGTATGTGTATTTTCTGGTCGATCTCTGATTATTTTTCTTCTTGTGCCATCGTTATTATTTTCTGAAGCTTGCCCACTAGGTTCCTATTGCCCACTTGCAAAACTCAATAACACAACTGGTGTATGTGAACC**GTAAGTTCAAATTCTAATTCTCTAATTACCATTCTATGCATCTGAGTGGCCTATAATGTGCAAAATATGTATATGACTTGGCAG**ATATCATTATCAACTACCTCCAGGACAGCCAAACCATACTTGTGGAGGAGCAAACATATGGGCTGATGTTGGTAGTAGTAGTGAGATATTCTGTTCAGCAGGATCATACTGTCCGACAACAGTCCAGAAAAATTCTTGCAGTAGTGG**GTATTAGTTTTTCCCCAACCTCTCTTATCCTTTTGCTGGTTATGACTATTCAGTGTGATGTGTTGACAATCAGGCTGTAATATTTTAACATTGTAGTTACGGCATTGATGTGTGGTTTTGATGACAATTAAACATTTCTGGCTGTCTTTCATGTTTGTTTGCTGATGCTATCTCATGTCTTTATGCAATTTCAG**ACATTATTGCAGGATGGGTTCCACATCTGAGAAAC**GTAAGGATTTTATTATTCTTTTTATTTTCATCAACATTGTTATTATTAAATTTTCCGGCTGTTGTGCATGTGCACATGCCTGTGCTGTGTTTCAGATTTATTGAAGGTTCAGGATTCATATACATGCCATTATTATATATCTTGTTTTTGTGTCTGGAGTTTTGCATTTCTTCTCTGCTGTATCGCTGAATTTGACCCTTGCCTATTAACTTGTAG**GTTGTTTCAAATTGACTTCATGCAAAGCAAACTCTACAAGTCAAAATATTCATGCATATGGAATAATGCTCATG**GTAAGTTGGTCAATTTACATCGGATGCAGTTGTATTATAACTAATCTTAGCTCCCTTCATGTCAACATTGTTAAGCTTTTCAAGTAAGACAAACGATTTTAATCTTAATGCTTGTACAACTTATCTATGCTTCCTAGAAAACTCTTAGTTTTTAATTCAGTTTTTACATAGTAATGACCTTTTTATGAAATATGCATTACATTCTTCTCTAATACTCTATAATTTGTAGCTGCTAATGGAAGTACATGCCATTTGTTTGCTTATGCAG**GTCGCTTTGACTACAGTGTTACTCATTATTTACAACTGCTCTGATCAAGTTATCACCACTCGAGAGAGGAGACTGGCCAAATCTAGGGAAGCAGGGGCAAGAAGTGCAAGGGAAACAGCAAGAGCACGTCAAAGGTGGAAAGTTGCAAAAGATGCTGCTAAGAAACATGCAAGTGGATTGCAAACTCATCTCTCACGGACTTTTTCTCGTAAAAAATATGGAAAATATCCTGAACAATTTAAGATTTTGAATCAAGATAAATCTGAGATGGAAGTTGATTTATATCCGCCTTCACACTCGAGTAATTTTTCTATTTCTACATCTCTGCCATCATCTGCACCATCAAAAGGAAAAAAAAAGGAACCTATTGACCTCATGCAAATGATGCATGAAATTGAGGTTGACCCTGATGGTTATGAGGGTATCAATCTTGAAGTTGCTGATCCAAATCCTACAAGACATATGCCAGAGGGAGAACAAATGACTACTCATACCCAGATTTTTAAGTATGCTTATGCTCAACTTGAGAAAGAGAAAGCTATGGAGCTAGAGAACAAGAATCTTACCTTCTCAGGAGTAGTTAATATGGCTATTAATACTGAAATCAAGAGAAGGCCTTTAATTGAGATTTCATTCAAAGAACTAACCCTTACTTTGAAAGCCAAGAACAAGCATCTTTTAAGGGGTGTAACTGGAAAAATAAAGCCTGGCCGTATTACAGCCGTCATGGGTCCATCAGGAGCTGGAAAGACAACCTTTCTTTCTGCTCTTGCTGGAAAACCAATTGGATGCAGGATGACTGGTTTGATTCTTATAAATGGAAAAAATCAATCCATCCACTCATATAAGAAAATCATTGGTTTTGTTCCACAAGATGATATTGTACATGGAAACTTAACTGTGGAAGAGAATCTCTGGTTCAGTGCTCATTGCAG**GTATATCTCAAACTTCTTTAACATTTCAACAGTAATTTCATGTACTATTTCTCAATTCCGCATACAGAATAAATCATATTATTGTCAGGCAGTAACTATGAGTTCATGACTAATAAACAATAGCTTGAATTATATGTAGGAGGGATGCAGCTATGCAGTTGTTCAGGACTTATTATATAAGATTGCTACTTTTGAATGTTGAAATTAATATATCTAGCTTGAGGAGATTAAATAATTAGAGTGAAGACATCTTTAATTAATTCAGAAAAGTTAAAAAAAAAAAAATCAAGTGCCCAATAACTTTATTTGCTATAATATCATATCCCAACTTATTGGCTCAGTCTGTTGTCCTACTATCTTTAGTTTCAATAGCTTCACATCTAATCTAAGTGAAGCTACAACCATTAAATGTATGCTAAACGTAAGGTGCTTAAACTCTTTTCTCCTTGCATTGTTTCCTTCATGAAATTAAGCATCATGTACTTAGTTTTGAATTATTTATTTTTAAGACTCAACCAGATTAACAAGTATAACTAGAATTGTGTTGGATTTCTTTTTCAATCTCAACATTACAGGACCAGAAGAACAAAAACAGAGAAAAGAAAGAGTCCCATCCCCCTCCCTGAATTTGTTGAATAACAATCAAGCATAAGACACCAGCCCACCCTCTCAAATTACTATTCCTGTGTCCATCCCCTTTGATATAATGATCTTATCAACTTTCTCCATGTCAGTTATGGTCATCCTTCTGATGAGATATATGAGTAGTTGTTCTAAGAAATTATTAAATTAATGTTCTTGCTTCTATTGTTATTTTTGCCTTTTTCTTGGTTCATCTATTTCTTTCTGCAATTTGCATGATGGCCTTCAACTAATCCCTCTGCACTTGTTGGTGCTTCTTTTTTCTATAATGGACTCTGTCACTCTGCTTATTGAAATGAAG**ACTATCTGCTGACTTGCCAAAGCCGGATAAGGTTCTGATTGTTGAAAGAGTCATTGAGTCTTTGGGGCTACAGACAGTGCGAGACTCCTTGGTTGGAACGGTAGAGAAGCGAGGAATTTCAGGAGGCCAGAAGAAGCGAGTAAATGTTGGACTGGAAATGGTAATGGAACCTTCACTTTTGATCTTAGATGAACCCACATCTGGTCTAGACAGTGCATCATCTCAGCTTCTTCTTAAAGCACTTCGGCGTGAAGCTCTTGAAGGGGTCAACATCTGCATGGTTGTTCACCAACCAAG**GTAATTTCCATAATAAGTAATCCTCACCTTTGATCTTTTGTTTTTTCATAATTTGAACTCACATAGTCACATTACATTCCTCTGGTTACACAAACACTGCATACCATATGAATGAGGAAAATAAGCTTATATTAACAAAGATTAGTGGAAAATCATGTGGATGGGATAGGGTTAAACACCAAATATAGCAGAATAATTATGCATATTTAAAGAAGAGCATGGCATAGGATAGATGTTAAAATTTTATTTTTGCCATTGGACAGTATTACCAATTACTATTTATTTCCTCTTGTGCGTCATTGTGATGGTTTTCTGAGATTGACCTATACTTCATGAAAATGTAAGTGTTAGAAAGTAACTTTTAAGTTGACTAAACTTAGTTGTGTCATGAATGTATATGCTGTCAGATTGTAGTCTTGGTAAAAGTACTGCATGTTGTTACTGCTGCAGTCTTGTCCAAACAACTTAAATCAACTTTCAAGTGGTATAATTGGTATATAATGACCTTTCAATTCATTTTGCATTCTTGGTCTATTTGAGTGTGGTTACTTCTACAGTAACTTTGTTTTTAACAGCTAGAACTTTTTCAGCTACACCTTGTTCAAAATGTTCGATGACTTAGTACTGCTGGCAAAAGGTGGCCTTATTGTCTATCATGGTCCAGCGAAGAAAGTTGAAGAATACTTTGCTTACCTTTCAATTCATTTTGCATTCTTGGTCTATTTGAGTGTGGTTACTTCTACAGTAACTTTGTTTTTAACAGCTAGAACTTTCTCAG**CTACACCTTGTTCAAAATGTTCGATGACTTAGTACTGCTGGCAAAAGGTGGCCTTATTGTCTATCATGGTCCAGCGAAGAAAGTTGAAGAATACTTTGCTGGCCTTGGGATTAATGTGCCAGAGCGTGTTAATCCCCCTGACCACTATATTGACATTTTGGAGGGTATAGTGACGCCGAGTCCAAGCTCAGGTGTGAATTATAAGGAACTTCCTATCAGATGGATGCTTCATAATGGGTACCCCATACCCCCAGATATGCAGCGGTATGCTGCTGGACTTGCATCTCCAGTGGACATAAATCCAGCGCATGAATCAAACCTTGGTGGTTTTGGAATGGAGGAACAATCTTTTGCAGGGGAATTGTGGCAAGATATGAAAAGTCATGTGGAGTTGCACAGGGATAAGATACGACATAATTTTTTAAAGTCCAGGGACTTGTCAAATCGGAGAACTCCAGGTGTTTTCTGGCAATACAGATACTTCCTTGGCAG**GTAAGTTCTTATTAAATTATGATTTATGACATTAAGATGTGTCTCAGTGTAATACAAGTTAAACATGTATCCATGTATTTTTCTATTGTTTATATTTTGTCAAGAAGTGTCTTTATGATTGTAAAATTGTTAAACTGAAGAGGTACCTTGTAATGTACTACCCAATAATGAGACTTCTGGAATGGTTGCCTCTAAAAGGTTTAGCCAAACATTATTGCTCATCCCTACCCTATATCTTAAACTCCTATCACTAGATAAGATGTTGCTTTTGCCTTTTAGTTGGGAACTTCGTTAAAGAATATTATTATAGATTATCTTTCGAAAACATAATAATATGTAGATTTACTTACATGTCATAATGCTAGTTACATTTAATCAGCATTTGCACGCTAAATAACAAGTAGCCGTAGTTTTGCCTCCAGGGAAAAGGAAGTGCGTTTTATCATATTGAGTTATCCATATAATATAGAGTGCTACCTGTTTCTGCTTTCCATTCTGTTATCCTAACTTATGATCTTCACAATCTTGCAG**AGTAGGTAAACAGCGACTAAGGGAAGCTAAAATACAGGCAATAGATTATTTGATCTTATTGCTTGCTGGAGTCTGCCTGGGATCCTTGGCAAAAGTAAACGATCAAACTTTTGGGGCTGCTGGTTACACATATACCATTATTGCAGTTT**GTAAGTTTGAAAAACTCTCAGTTGCTCAAGCTGTAAGGTTTTCTTTGAATTCAATTTGCTGTCTTGATTTGCAATTGCAGAAAATCAGAAATTATTTAAAGAAATTTGACTTGATTATTCTTGCTAACTCAGATTATTGTTTATCATTTGACATTGGACTGTAAAGTTGCATGTAAGGCATAAAACACAAAAACAATGCATAGTGATGTTTAGATCATTCAGTTTAGCATTCTTTTATGCACAAAATTGTTTTCTTACCACTACTTTTATTTCTGATACCAG**CTCTCCTTTGCAAAATTGCGGCTTTGAGATCGTTTTCTCTTGATAAGTTACAATACTGGAGAGAGAGCGCATCTGGCATCAGCAGCTTGGCTTACTTTCTTGCCAAGGATACAATTGATCATTTTAATACAGTGATTAAGCCTGTACTGTATCTCTCTATGTTCTATTCTTTCACAAACCCAAGGTCTTCCTTCACCGATAATTATGTTGTCCTGCTGTGCCTTATTTACTGTGTAACTGGTATAGCCTATGCATTGGCTATCTTTTTTGAACCAGGTCCGGCCCAACTG**GTGAGTGATGATGGTTCACAGTGAAACGATCATCTGCTTTTGAATATAGATTTTCAGATAGGCCACTATGCTCATATTGACGAGCTTTGTTTCTTGCAG**TGGTCAGTTATTCTTCCAGTTGTTTTGACTCTCATTGCAACACAAGCAAACCAGAGTAATACCTTGAAGAACATAGCTAATTTATGCTACCCAAAATGGGCTCTGGAAGCATTTGTGATTGCAAATGCTGAGAG**GTATGGCAATCATCTTCTTTGTGCATTTCAAATGTATGTTATATTCATTGGTCTACATATGTATCATAAAAACATATAAAAAGATGTTTCACGTTTGATGGTTTTGTGTTAACAAAAATACTCTCAATGTTATACTTAATTCCATGCCTACGGGCTTAATTTTTAAAATTAAAAAAAAAAAAAAAGCAAATTTTGTTATTTCTAGAAATTTATAATCATAATATAAAAGAAGCTTTATGTTTGAAAGTTTGGTGCTTAAATGTTTATGTTATAAATACTTCCAAGTCATTTTGATTGACATAGTTAGTTTTTCTATAAAAAATTTATTGGCACATGCTACCCATCAACTAGTACTTACAAGAGGGTGTAGACCTGATAAATCTGAAAAACCTGAATCACAAGATTTTACTAAAGATGCAAACAAATTACTTTTGATAGTGTTGATCACAGATGGAAGTTGCATGGTCTCAATTATGCACAAATTATTTGAGTATTTAATAGTGGTAACGGCATGTAATTCTTTATTGTACCATGCTTGGTAGGCTAGTACACACTTATTTTAGGGTGGTTGCTTCTAGGCAAGCCTCTTATCCCTAAGCCGTCATTTAGGCACCTTATTAAGTTGGCCCTTAATTACATGTTGATGTTATGGATGTTCCAAAGTATTAAAAAAATTATTTTTATAAATGAATAAACAACAAAATACACAATTGGCATGGGTTGGGTATAGATCAAGAGTTTTTCTTGCCTGGACCATAATCCATGCAAGAATTTTCCATGGATCAGATGGGTTTAGAAGTCGACAAGTTACTTCAAAAATGATTTAGCACTGGATTTAACTCATGACAAGAATTGTTTAACCTAGTAATTTAAAGAAAGGAAAATTTTTGTATTCTGAAAATCGTATCCTATGTATGGCTGTATGCCACCAAACCAAAGACCACTGTGGCCTTCTTTGTCTCATTTCATAACCTGAGAGTAACCTGTTGTTTAAAATCTACAATTTTACAAGAATTCAATTCAATTGATATTTTTTTGTCAATTCTTATGTTTAAAATGAAAGAATTCAAAAGTCATTTTCAAAGAAATAGAAATCAAGTATGATTGTGTGAGAATTCAATTGAATTCTTTTTTTTGCAAAGAATTTATTCCAAGCAAAGCCTAAAGGATTGTATTCTTCAAAGAAAAGTCGAATTTGAGGTGTGTCAGTCTGTCCACTCATGTTGTCTTGTAAATTTTTGGAGAATCATGCATGTTCAAGAATGAAGGAAAATGCTACTTGTGTTCACCCCCAAGTCCTTAGTATACCATATTAGCTATGATGTACATATTAATTAGCACACTACACGTGACTAAAATGGATTGTATTGACTAACAAAATTGAATTACATTAAAGTTCACATTTGAAAAGAATCTGCATAACCACAGTTCCTTGAATGAGGGAATGGAATCCACTATTTTCAGAAACTGCTAAAAAGTTGTGTTCTTATAAATTTATAATGATACACAAGTCCTAGAACACAACTAGGAGATTAATCTTAAAAACGTTTCATGTTGTTGATGATGCATAGAATGATTATTTTTGTTTTCATTTCCATTCAG**GTATTATGGAGTGTGGTTAATTACTCGTTGTGGTTCACTTCTGAAAACTGGGTATAATCTCCATCACTGGGGTCTTTGTGTATTCATTCTCATCCTCATCGGCATGGTTACTCGTTTTGTGGCATTCTTTGGTATGGTAACATTCAAAAAGAAGTAGAGTTTCACACTTTCAACTCCATTCTTTTGGCATTGTAAACTCCTGGTATCTACTTACAAATGTGGAATTCTGCAACATCAGGAGGCAGTGCCATCTTAAAAATTCAGTACAATGATACAAAAATTAAGGGTAGTTAATGATGCATAACTAGTGCAGCATTATAATAATAGTTTGTTTTCTTAGGTTTTCTCATTTATACTATTGTAGGTCATCCATTCTTGTAAAAATA**

>HbABCG40 scaffold1402(107311-114766)
[truncated: 57,466 more chars]
