# Supplementary material for: Whole-Transcriptome Survey of the Putative ATP-Binding Cassette (ABC) Transporter Family Genes in the Latex-Producing Laticifers of Hevea brasiliensis
Source: PLoS One. 2015 Jan 23;10(1):e0116857. doi: 10.1371/journal.pone.0116857 (PMC4304824; doi:10.1371/journal.pone.0116857)
Supplement: S4 Table — (DOC) [file pone.0116857.s005.doc]

Table S4. The predicted amino acid sequences of the 46 ABC protein genes identified in the *H. brasiliensis* latex*.

(*The TMD sequences are presented in italic, and the NBD sequences are bolded.)

>HbABCA1

MGTCRRQLKAMLRKNWLLKIRHPFVTAAEILLPTIVMLLLIAIRTRVDTRIHPAQPYIREDMFVEVGKGISPNFQLVLESLLAEEEFLAFAPDTEETRMMIHFLSMKFPLIREVSRVYKDEVELETYICSDLYGACNGVKNCS*NPKIKGAVIFHDQGPQLFDYSIRLNHTWAFSGFPDVKTIMDVNGPYLNDLELGVSPIPTMQYSFSGFFTLQQVVDSFIIFSAQQTGTKAAGGHIELPSSNSSISSLLKVPWMQYSPSKIRIAPFPTREYTDDEFQSIVKTVMGVLYLLGFLYPISRLISYSVFEKEQKIREGLYMMGLKDGIFHLSWFIAYALQFAISSGIITGCTMNNLFQYSDKSVVFVYFFSFGLSAIMLSFLISTFFTRAKTAVAVGTLSFLGAFFPYYTVNDEAVPMILKVLASLLSPTAFALGSINFADYERAHVGLRWSNIWRGSSGVNFLVCLLMMWLDTLLYCAVGLY*LDKVIPRENGVRYPWNFLFKNCFWRKKSMIKHHVPSLEVKLNGKLSNLGNDTVEPAVESISLDMKQQELDNRCIQIRNLHKVYATKGGSCAA**VNSLHLTLYENQILALLGHNGAGKSTTISMLVGLLPPTSGDALLFGKNILTDMDEIRNGLGVCPQHDILFPELTVREHLEMFATLKGVKEEILETSVTDMVDEVGLADKVNTVVRALSGGMKRKLSLGIALIGDSKVIILDEPTS**GMDPYSMRLTWQLIKKIKKGRIILLTTHSMDEADELGDRIAIMANGSLKCCGSSLFLKHQYGVGYTLTLVKSAPTASAAADIVYRHIPSAICVSEVGTEISFKLPLASSLSFESMFREIESCMRVSKSKISSSEDKNYLGIESYGISVTTLEEVFLRVAGCDYDGTDGFEQRSNILSSGSVVPTASHNHGSKRVFGSKLLGNYRKFIGFISALVGKACGLMVATVLSFINFIGMQCCSCCIISRSTFCQHTKALFIKRAISARRD*RKTIVFQLLIPAVFLLFGLLFLKIKPHPDQQSVSLTTSHFNPLLSGGGGGGPIPFDLSQPIAREVVKYIKGGWIQSFEKSVYKFPDSEGALANAIKAAGPTLGPVLLSMSEFLMSSFNESYQSRYGAVVMDDQNDDGSLGYTVLHNSSCQHAAPTYINVMNAAILRLATGDKNMTIRTRNHPLPMTKSQHLQRHDLDAFSAAVIINIAFSFIPASFAVAIVKEREVKAKHQQLISGVSVLSYWASTYIWDFISFLFPSFLAIVLFYIFGLDQFIGRDCFFPTLLIFLEYGLAIASSTYCLTFLFSDHTMAQNVVLLVHFFTGLVLMVISFIMGLIETTRSANNFLKNIFRISPGFCFADGLASLALLRQGMKDKSSDTVFDWNVTGASICYLGIESIIYFLLTI*GLELLPSHKLTPVTIKQCWRNFKNFWHGSSGFSEPLLKFPSEVVGVDFEEDIDVQTERNRVLSGSVDNAILYLRNLQKVYPGGKYGMKVA**VHSLTFSVQPGECFGFLGTNGAGKTTTLSMLSGEESPTDGTAFIFGKDIRSNPKAVRRHIGYCPQFDALLEFLTVREHLELYARIKGVADYRMEDIVIEKMVEFDLLRHADKPSFALSGGNKRKLSVAIAMIGDPPIVILDEPST**GMDPIAKRFMWEVISRLSTRQGKTAVILTTHSMNEAQALCTRIGIMVGGRLRCIGSPQHLKTRFGNHLELEVKPTEVSSVDLENLCQIIQERLLNIPSHPRSLLDDIEVCIGAVDCITSENASVAEISLSQEIIMLIGRWLGNEERAHTLLSMPVSDGVFGEQLAEQLVRDGGIPLPIFSEWWLAKEKFSAIDSFVLSSFPGATVQACNGLSVKYQLPYRDGLSLADVFGHLEQNRNQLGIAEYSISQATLETIFNHFAASL

>HbABCA2

MELQRGFPLLYQQSKALLKKNLSLSWRNKSASFLQLFSSLFFIFLLFCIEKAMNAANSDTTAYKSVLDPQPLVSPPIPPCEDKFYIKKPCF*DFVWSGNYSDRLNRIVSSIMANNPGRVITSNKVKSFRTTDDVDVWLLNNPMHCPGALHFKDINATVISYGVQTNSTRVMNRGYSEDPTFKFQIPLQIAAEREIARSIIGDPNFSWVVGLKEFAHPAKNNFSVLASIGPTFFLAFAMFGFVMQIGSLVVEKELKLRQAMSMTGLYESAYWFSWITWEGILSLVSSLLLVLFGMMFQFDMFKKNNFAVVFLVFYLFQLNMVGFAFLLSNFISKSSSATTVGFSVFIVGFFTQIVTIFGFPYGIHVASILRTLWSLFPPNLLGIAVNLLAQATATPEDVGISWSRRSECVRDDSAADKCVISINDVYLWLTSTFFVWFVLAIY*FDNIIPNAYGVRKPIFYFLKPGYWTGKGGNRVEEGGICSCMGSVPQQEHITPEDEDVLEEENIVKQEAKDGLVNPEVAVQVRGLAKVYAGTTKIGCCKCKKTSPYHA**LKGLWMNFAKDQLFCLLGPNGAGKTTAINCLTGLTPVTSGDALIYGYSIRSPVGMSNIRRIIGVCPQFDILWDALSGAEHLHLFASIKGLPPDSINLVAEESLAEVRLTEAAKVRTRSYSGGMRRRLSVAIALIGNPKLVILDEPTT**GMDPISRRHVWDIIQNAKKGRSIVLTTHSMEEADILSDRIGIMAKGRLRCIGTSIRLKSRFGTGFITNVSFIESNAEQSPPNASVDLSNQHEDVKQFFKYHLDVMPAEETKSYLTFVIPHDREKLLTRFFSELQDREREFAIADIQIGLATLEEVFLNIAKQAELESAAAEGRLVTLTLTSGASVQIPIGARFVGIPGTQSPENPRGIMVEVYWEQDDSGSLCISGHSAEMPLPLNVQQFISASQPRSNINFLRRGKEPVYGIMIDPNQISPSNY

>HbABCA7

MADTSHGPSSFWTQADALLRKNLTYQKRNAKTNCRLILFPFILCILLVITQSLLDHELNKASRKCGCKDVDINGNGQLEKVCGLQYSDAFQAATCSIPSPPQWPPLLQIPAPQYRAVRSEVIPFTDLPNDSCRSTGSCPVTILFTGNNQSLGENLAGNMFPSSFTINSSNYMDSLAYNALGSDTEPKRDNFID*PAFIENSTLYYVQHQCASNSTLSISVQSVIEFQKEAACVQDLKLWRNSSSEINEQLFKGYRKGNSDEKINEILAAYDFLNSNGNNFNVSIWYNSTYKEGDIQGQFNYLRVPRFVNLVSNAYLQFFQGPGTKMLFEFVKEMPKAASKINVDLASLLGTLFFTWVILQLFPVVLTSLVYEKQQKLRIMMKMHGLGDGPYWMISYTYFLSISLMYMLVFVIFGSVIGLKFFTLNDYGIQIVFYFIYINLQISVAFLVAAFFSNVKTATVVGYIGVFGTGLLGGFLFANFVEDSSFPRGWIIVLELYPGFSLYRGLYEFSQYTFTGNAMGTHGMRWGNLSDSKNGMRQVLIIMFVEWLVLLFVAYY*VDQVLSSGSGKSPLFFLQNFGKKRPSSFRKPSLQRQGSKVFVDMDKPDVIQEREKVEHLLLEPTTTHAIICDNLQKVYPGRDGNPEKLAV**RGISLALPPGECFGMLGPNGAGKTSFISMMIGLTKPTSGTAYVQGLDIRTHMDWIYTSMGVCPQHDLLWETLTGREHLLFYGRLKNLKGSALIQAVEESLRSVNLFNGGVADKQAGKYSGGMKRRLSVAISLIGDPKVVYMDEPST**GLDPASRSNLWNVVKRAKQDRAIILTTHSMEEAEALCDRLGVFVDGSLQCIGNPKELKGRYGGSYVFTMTTSLDHEQEVVMMVQQLSPNAERTYHTSGTQKFEMPKNEVRIADVFHAVEIAKSRFPVFAWGLSDTTLEDVFIKVANGA

>HbABCB1

MSQESQEIKTIEQWKWSEMQGLELVPPAHDPFINNTASAPPTPTLTINSKEHQQQEENHQETVLERREMDNTTPKKDGSGAGSSSSPSGNGEKSGDVATVGFGELFRFADGLDYVL*MAIGSIGALVHGSSLPLFLRFFADLVNSFGSNANDMDKMMQEVLKYAFYFLIVGAAIWASSWAEISCWMWTGERQTTRMRIKYLEAALNQDIQYFDTEVRTSDVVFAINTDAVMVQDAISEKLGNFLHYMATFVSGFVVGFTAVWQLALVTLAVVPLIAVIAAIHTNTLAKLSGKSQEALSQAGNIVEQTIVQIRVVLAFVGESRALQGYSSALKVAQRVGYKSGFAKGMGLGATYFVVFCCYALLLWYGGYLVRHHYTNGGLAIATMFAVMIGGLAL*GQSAPSMGAFAKAKVAATKIFRIIDHKPAVDRNSESGLELDSVTGLVELKNVDFSYPSRPDVRI**LNNFTLNVSAGKTIALVGSSGSGKSTVVSLIERFYDPNSGQVLLDGHDIKTLKLRWLRQQIGLVSQEPALFATTIKENILLGRPDADQIEIEEAARVANAHSFIVKLPEGFDTQVGERGLQLSGGQKQRIAIARAMLKNPAILLLDEATS**ALDSESEKLVQEALDRFMIGRTTLVIAHRLSTIRKADLVAVLQQGSVTEIGTHDELIAKGENGVYAKLIRMQEMAHETAMNNARKSSARPSSARNSVSSPIIARNSSYGRSPYSRRLSDFSTSDFSLSLDATHSNYRLEKLAFKEQASSFWRLAKMNSPEWVYA*LVGSVGSVICGSLSAFFAYVLSAVLSVYYNQNHAYMSREIGKYCYLLIGLSSAALIFNTLQHFFWDIVGENLTKRVREKMLSAVLKNEMAWFDQEENESARIAARLALDANNVRSAIGDRISVIVQNTALMLVACTAGFVLQWRLALVLISVFPLVVAATVLQKMFMTGFSGDLEAAHAKATQLAGEAIANVRTVAAFNSESQIVGLFDTNLQIPLRRCFWKGQIAGSGFGIAQFSLYASYALGLWYASWLVKHGISDFSKTIRVFMVLMVSANGA*AETLTLAPDFIKGGRAMRSVFDLLDRKTEIEPDDPDATAVPDRLRGEVELKHVDFSYPTRPDVPI**FRDLNLRARAGKTLALVGPSGCGKSSVIALIQRFYEPSSGRVMIDGKDIRKYNLKSLRKHIAMVPQEPCLFAATIYENIAYGHESATEAEIIEAATLANAHKFISGLPDGYKTFVGERGVQLSGGQKQRIAIARALVRRAELMLLDEATS**ALDAESERSVQEALDRACSGKTTIVVAHRLSTIRNAHVIAVIDDGKVAEQGSHSHLLKNYPDGCYARMIQLQRFTHSQVIGMTSGSSSSARQKDDEEREG

>HbABCB11

MAEENGDPRMHEANTSNSQEQEKHSSSNGSKENDKEKAKTVPFLKLFSFADSTDVLL*MITGTVGAIGNGVSMPLMSLLLGQMIDSFGGNQSDKDIVNIVSKVSLKYVYLAVGAGAAAFLQVTCWMVTGERQAARIRSYYLKTILRQDIAFFDKETNTGEVVGRMSGDTVLIQDAMGEKVGKFLQLMATFIGGFVIAFVKGWMLALVMLSAIPLLVLAGATVSILISRMATRGQNAYAEAATVVEQTIGSIRTVASFTGEKRAISVYNKYLQIAYKSGAHEGFASGVGIGIVMLVVFSSYAMAVWFGAKMILEKGYSGGQVINVIVAVLTGSMSLGQTSPCMSAFASGQAAAYKMFETIDRKPEIDAYDTSGRVLDDIHGDIELKDVYFSYPARP*DEEI**FSGFSLSIPSGTTAALVGHSGSGKSTVISLIERFYDPKSGEILIDGINLKEFQLKWIRGKIGLVSQEPVLFSSSIKDNIAYGKDGATIEEIRAAAELANAAKFIDKLPQGLDTMVGEHGTQLSGGQKQRIAIARAILKDPRILLLDEATS**ALDAESERVVQEALDRIMVNRTTVIVAHRLTTVRNADMIAVIHRGKMVEKGTHSELLEDPDGAYTQLIRLQEVNKETEQAPQDYSRSEISMESFRQSSQRRSLRRSISRGSSRNSSHHSLSLSFGLPTGFNGPENDLADVEDFPSKEQIPEVPIRRLAYLNKPELPV*LIVGTIAASINGTILPIYGILISKAIKTFFEPPHELRKDSKFWALMFTTLGLASFVVHPFRTYFFSVAGSKLIQRIRSMCFEKVVHMEIGWFDEPEHSSGAIGARLSTDAATVRALVGDALAQMVQNIATAVAAMVIAFTASWQLAFIILALIPLIGVNGVVQVKFMKGFSADAKMMYEEASQVANDAVGSIRTVASFCAEEKVMQLYEKKCEGPMWTGVRLGLISGIGFGLSSFFLFCFYATSFYAGARLVEGGHITFADVFQVFFALTMAAVG*ISQSSSIGTDSTKAKAAAASVFAIIDRKSKIDPSDESGTTIENVRGEIELHHVSFKYPSRPDIQI**FRDLSLTIRSGKTVALVGESGSGKSTVIALLQRFYDPDSGHITLDGIEIQKLQLRWLRQQMGLVSQEPVLFNDTIRANIAYGKEGDATEAEIIAAAELANAHKFISGLQQGYEAAVGERGVQLSGGQKQRVAIARAIVKSPKILLLDEATS**ALDAESERVVQDALDRVMVNRTTVVVAHRLSTIKNADVIAVVKNGVIVEKGRHETLINIKDGFYASLVALHMSAQTA

>HbABCB13

*MQTGERQTARLRLEYFQSVLKKDMNFFDTEAGDSNIIYHISSDAILVQDAIGDKTGHAIRYLCQFIVGFAIGFASVWQLTLLTLAVVPLIAIAGGAYTVIMSTLSEKGEAAYAEAGKVADEVISQIRTVYSFVGEHKAIEAYSRSLTSALKLGKKSGIAKGVGVGFTYGLLFCAWAMLLWYSSILVRHHITNGAKAFTMIINVIFSGFALG*QAAPNLAAIAKGRAAAATIINMIETGSNPSKRSEHGSELPKVEGKIEFSNVCFAYPSRPSKV**FENLSFTISAGKTFAVVGPSGSGKSTVISMVQRFYDPNSGKILLDGHDLKTLRLKWLREQMGLVSQEPALFATTIADNILFGKEDANMDKIVQAAKAANAHSFIQQLPDGYHTQVGEGGTQLSGGQKQRIAIARAVLRNPKILLFDEATS**ALDAESEFIVQQALNKIMSNRTTIIVAHRLSTIRDVDTIIVLKNGQVAESGSHLDLITKGGDYATLVSLQVSEHPTRSNSIGGSEASGNSSFRQLPHSQNNQQDFKSISIRELQSKDDGMPLQKHSPTPSILELLKLNAPEWPCA*LLGSLGAILAGMEAPLFALGITHVLTAFYSHDASEMRHEIQRVSLIFVGLAVVTVPIYLLQHYFYTLMGERLTTRVRLSMFSAILCNEIGWFDLEENNTGSLTSALSADATLVRSALSDRLSTIVQNAALTVTACAIAFTLSWRIAAVVVASLPLLVGASIAEQLFLKGFGGDYHAYSRATAVAREALTNIRTVAAFGAEERISVQFASELNKPNKQALLRGHISGFGYGLTQLFAFGSYALGLWYASVLIRHKESNFGHIIKSFMVLIITALAI*AETLALTPDIVKGSQALGSVFNVLHRKTAIDTNDLSSKVVTDIKGDIEFRNVNFKYPARLDITI**FELLNLKVPAGKSLAVVGPSGSGKSTIISLILRFYDPISGTVLIDGCDIKTLNLKSLRLKIGLVQQEPALFSTTIYENIKYGNENASEIEIMKAAKAANAHGFISTMPEGYKTHVGDRGLQLSGGQKQRVAIARAILKDPSILLLDEATS**ALDTASEKLVQEALDKLMEGRTTVMVAHRLSTIRDADSIAVLQHGRVAEIGSHKQLMGKPGSIYKQLISLQQEESIQS

>HbABCB15

MVKKKSSHVGSIRSIFMHADGVD*WFLMVLGVIGSVGDGFSTPLVLFVTSKLMNNIGGASSFQSDFSHNINKNALALCYLACGQWVVCFVEGYCWTRTGERQATRMRARYLKAVLRQEVGYFDLHVTSTAEVITSVSNDSFVIQDVLSEKVPNLLMNASMFFGCYLVGFLLLWRLAIVGFPFIVILVIPGLMYGRTLMGLARKIKEEYNKAGTIAEQALSSIRTVYAFVGESKTVTAYSAALDFSVKLGLKQGLAKGLAIGSNGVVFAIWSFMSYYGSRLVMYHNARGGTVFAVGASIAVGGLAL*GAGLSNVKYLSEACTAGERIMEVIRRIPRIDLENLEGEILENVGGEVEFKHVEFAYPSRPESII**FKDFTLKIPAGRTVALVGGSGSGKSTVIALLQRFYDPLDGEILLDGVAIDKLQLKWLRSQMGLVSQEPALFATSIKENILFGKEDATMEEVVEAAKASNAHNFICQLPQGYDTQVGERGVQMSGGQKQRIAIARAIIKAPRILLLDEATS**ALDSESERIVQQALDKAAIGRTTIIIAHRLSTIRNVDVITVVQNGQVMETGSHDELMEIEDGLYTTLIRLQQTEKEKSNEDDQYHIPSSSLISKMDMNNTSSRRLSMVSRTSSANSIAPSRASVNAENIQLEEQKFPVPSFRRLLALNLPEWKQA*SFGCLGAILFGGVQPLYAFAMGSMISVYFYTDHDEIKKRIRIYSLCFLGLSIFTFIVNIVQHYNFAYMGEYLTKRIREKMLSKMLTFEVGWFDQDENSSGAICSRLAKDANVVRSLVGDRMALVVQTVSAVVIACTMGLFIAWRLAIVMIAVQPLIIVCFYTRRVLLKSMSHKAIKAQDESSKLAAEAVSNLRTITAFSSQDRILRMLEKAQEGPLRESIRQSLFAGIGLGTSQSLMSCTWALDFWYGGKLISKGYITAKDLFETFMILVSTGRV*IADAGSMTTDLAKGSDAVGSVFAVLDRYTKIEPEGADGLKPEMIMGHVELRDVNFAYPARPDVII**FEGFSIKIEAGKSTALVGQSGSGKSTIIGLIERFYDPIRGIVKIDGRDIKSYHLRSLRKHIALVSQEPTLFAGTIRENIAYGTSKNDESEIIEAAKAANAHDFIAGLKDGYDTWCGDRGVQLSGGQKQRIAIARAILKNPTVLLLDEATS**ALDSQSEKVVQDALERVMIGRTSVVVAHRLSTIQNCDLIAVLDKGQVVEQGTHSSLLAKGPTGAYFSLVSLQRTPHNSTTTASHTFN

>HbABCB19

MAAETVDTTTTTNTSSKASLPEAEKKKEQSLPFYHLFSFADKYDWLL*MISGSLGAIIHGSSMPVFFLLFGEMVNGFGKNQSDLPKMTHEVSKYALYFVYLGLVVCISSYAEIACWMYTGERQVGTLRKKYLEAVLKQDVGFFDTDARTGDIVFSVSTDTLLVQDAISEKVGNFIHYLSTFLAGLVVGFVSAWRLALLSVAVIPGIAFAGGLYAYTLTGLTSKSRESYAQAGIIAEQAIAQVRAVYSYVGESKALNSYSDAIQNTLKLGYKAGMAKGLGLGCTYGIACMSWALVFWYAGVFIRNGQSDGGKAFTAIFSAIVGGMSL*GQSFSNLGAFSKGKVAGYKLMEIIKQKPSIIQDPSDGKCLPGVNGNIEFKDVTFSYPSRPDVMI**FRDFSIFFPAGKTVAVVGGSGSGKSTVVSLIERFYDPNQGQVLLDNVDLKTLQLRWLRDQIGLVNQEPALFATTILENILYGKPDATMDGVEAAASAANAHSFITLLPNGYNTQVGERGVQLSGGQKQRIAIARAMLKNPKILLLDEATS**ALDAGSESIVQEALDRLMVGRTTIVVAHRLSTIRNVDTIAVIQQGQVVETGTHEELIAKGRAYASLIRFQEMARNRDFANPSTRRSRSSRLSHSLSTKSLSLRSGSLRNLSYSYSTGADGRIEMISNAETDRKNPAPDGYFCRLLKLNAPEWPYS*VMGAIGSVLSGFIGPTFAIVMSNMIEVFYYRNPASMERKTKEYVFIYIGAGLYAVVAYLIQHYFFSIMGENLTTRVRRMMLAAILRNEVGWFDEEEHNSSLVAARLATDAADVKSAIAERISVILQNMTSLLTSFIVAFIVEWRVSLLILATFPLLVLANFAQQLSLKGFAGDTAKAHAKTSMIAGEGVSNIRTVAAFNAQDKILSLFCYELRVPQRRSLRRSQTSGLLFGLSQLALYASEALILWYGAHLVSKGSSTFSKVIKVFVVLVITANSV*AETVSLAPEIIRGGEAVGSVFSILDRSTRIDSDDPEAEPVEAVHGEIELRHVDFAYPSRSDVPV**FKDLNLRIRAGQSQALVGASGCGKSSVIALIERFYDPTAGKVMIDGKDIRRLNLKSLRLKIGLVQQEPALFAASIFDNIAYGKDGATEAEVIEAARAANVHGFVSALPDGYKTPVGERGVQLSGGQKQRIAIARAVLKDPAILLLDEATS**ALDAESECVLQEALERLMRGRTTVLVAHRLSTIRGVDSIGVVQDGRIVEQGSHSELISRVDGAYSRLLQLQHHQI

>HbABCB20

MMISRGLFGWSPPHIQPLTPVSEVSEPPESPSPYLDTSAEAAAAAAAAAQAEAEEEMEEPEDLDPPPAAVPFSRLFACADRLDWCLMIVGSLAAAAHGTALVVYLHYFAKIVQVMGIPPDR*PEDRFDRFKDLSLTIVYIAVGVFAAGWIEVSCWILTGERQTAVIRSNYVQVLLNQDMSFFDTYGNNGDIVSQVLSDVLLIQSALSEKVGNYIHNMATFFSGLVIGFINCWQIALITLATGPFIVAAGGVSNIFLHRLAESIQDAYAEAASVAEQAVSYIRTLYAFTNETLAKYSYATSLQATLRYGILISLVQGLGLGFTYGLAICSCALQLWVGRFLVTHNKAHGGEIITALFAVILSGLGLNQ*AATNFYSFDQGRIAAYRLYEMISRSSSTVNQDGNTLVSVLGNIEFRNVYFSYLSRPDIPI**LSGFYLTVPAKKAVALVGRNGSGKSSIIPLMERFYDPNLGEVLLDGENIKNLKLEWLRSLIGLVTQEPALLSLSIKDNIAYGRDATLDQIEEAAKIAHAHTFISSLERGYETQVGRAGLALTEEQKIKLSIARAVLLNPTILLLDEVTG**GLDFEAERTVQEALDLLMLGRSTIIIARRLSLIRNADYIAVMEEGQLVEMGTHDELLNLDGLYAELLKCEEAAKLPRRMPARNYNETNAFQIEKDSSASHSFQEPSSPKMMKSPSLQRVPGVLRPPDGTFNSQESPKALSPPPEKMMENGLPLDGADKEPSIRRQDSFEMRLPELPKIDIQSANRQTSNGSDPESPVSPLLTSDPKNERSHSQTFSRPHSHSDDIPTKFKDGKDTKHREAPSFWRLAELSFAE*WLYAVLGSIGAAIFGSFNPLLAYVIALIVTAYYRPERHHLQQDVDKWCLIIACMGVVTVVANFLQHFYFGIMGEKMTERVRRMMFSAMLRNEVGWFDEEENSADTLSMRLANDATFVRAAFSNRLSIFIQDSAAVVVAIIIGMLLQWRLALVALATLPILMVSAIAQKLWLAGFSRGIQEMHRKASLVLEDAVRNIYTVVAFCAGNKVMELYRLQLKKIFRESFLHGMAIGFAFGFSQFLLFACNALLLWYTAYSVKNHYMDLPTAIKEYMVFSFATFAL*VEPFGLAPYILKRRKSLISVFEIIDRVPKIDPDDNSALKPPNVYGSIELKNIDFCYPTRPEVLVLSNFSLKVNGGQTVAVVGVSGSGKSTIISLIERFYDPVAGQVLLDGRDLKLYNLRWLRSHLGVVQQEPIIFSTTIKENIIYARHNASEAEMKEAARIANAHHFISSLPHGYDTHVGMRGVDLTPGQKQRIAIARVVLKNAPILLLDEASSSIESESSRVVQEALDTLIMGNKTTILIAHRAAMMRHVDNIVVLNGGRIVEEGTHDSLMVKNGLYVRLMQPHFGKGLRQHRLV

>HbABCB25

MLGASKWFRFSTRELILRNGSCKKPVLLAQNNILLRGSISSSSSVISGSYSAYLPWKNLRSRRSSAGPLNLKNFLSDSGFSSAPSRPPSAMLNGRVLFSTSAPSNPNANQNHGAKSSIATTKSEEKHVADMKILRTLASYLWMKDNFEFRLR*VITALAFLVGAKVLNVQVPFLFKLAVDWLSTATGNATALASFTTANSTLIALFATPASVLIGYGIARSGASAFNELRTAVFSKVALRTIRQVSRKVFSHLHDLDLRFHLSRETGGLNRIIDRGSRAINFILSSMVFNVVPTVLEISMVSGILAYKFGAPFAWITSLSVAAYVAFTLSVTQWRTKFRKAMNKADNDANTRAIDSLINYETVKYFNNEAFEADKYDEFLKRYEGAALKTQRSLAFLNFGQNVIFSSALSIAMVLCSHGIMNGQMTVGDLVMVNGLLFQLSLPL*NFLGSVYRETIQSLVDMKSMFQLLEERADIRDKDVAKPLKLNRGSIQFENVHFSYLVERKI**LDGISFVVPAGKSVAIVGTSGSGKSTILRLLFRFFDTDSGSIKIDGQDIRDVTLESLRRSIGVVPQDTVLFNDTIFHNIHYGRLSTTEEEVYDAARRAAIHDTILNFPEKYSTVVGERGLKLSGGEKQRVALARAFLKAPPILLCDEATS**ALDSTTEAEILSALKSLANNRTSIFIAHRLTTAMQCDEIIVLENGKVVEQGPHEVLLTKAGRYAQLWAQQNNSVDSLDAAIKLEA

>HbABCB26

MAVLLCNPLAQRCLLSSLHCKNQRPLSIRPAANTKLRFSPSEFSLSSGRRCFCPLKSSSINGFSIAKKDHVEQFEGEQREENFELHGRIRKFFEFLPSILPGGNWWSFSEDVEIKFLAKPVTMWRALGRMWQLVAHDRW*VIFAAFSVLIVAAVSEISIPHFLTASIFSAQSTQIALFHQNVRLLVLLCVIAGICSGLRGCFFGIANMILVKRMRETLYSALLLQDISFFDNETVGDLTSRLGSDCQQVSRVIGNDLNLILRNAVQGTGALIYLLILSLPLGLCTLIICSTLAAVMLIYGMYQKKAAKLTQEFTASANQVAQEAFSLMRTVRIYGTENLELERYKLWLEKLASISLRQSAAYGFWNLSFNTLYHSTQVIAVLVGGTFILGGHITAEKLTKFILYSEWLIYST*WWVGDNLSSLMQSVGASEKVFQLMDLLPSDQFISKGLKLQRLVGQIEFVNVSFYYPSRAAIPV**LQHVNLSVHPGQVVAIVGLSGSGKSTLVNLLLRLYEPTNGQILIDGFPLRELDIKWFRERIGYVGQEPKLFRMDISSNIRYGCTRDISQKDVEWAAKQAYAHDFISSLPNGYETVVDDDLLSGGQKQRIAIARAILRDPAILILDEATS**ALDAESEHNIKGVLRAIGSDFTTKRTVIVIAHRLSTIQAADRIVVMNGGQIIEMGNHRELLHQDGLYARLTRRQADAVA

>HbABCB28

MKICQATWECETISCWVSLVLRSTARKSMASATGVLLQINPTRFPIPKLHARPINKHKLRQSQLSISSAFCHFPPFSQSYVKRWSTKISTISCAYVSGPPTLSEPDPKVDASESTTEEVQSTKLISWGLLWSLLLKHKL*RLGVSVLALVGCTTCTLSMPLFSGRFFEVLIGARPEPLWRLLSKVGLLYSLEPICTVIFVVNMNAVWEKVMSRLRAHIFRRVLIQKVEFFDRYKIGELSALLTFDLGSLKDIVNENISRDRGFRALSEVIGTICILFALAPQLAPILGILMLSVSVLVATYKRSTIPVFKAHGMAQASISDCVTETFSAIRTVRSFSGEKRQMLMFGSQVLAYQGSGIKLGTFKSVNESLTRIAVYISLMALYCLGGSKVKAGELSVGTVASFIGYTFTLTFAV*QGLVNTFGDLRGTFAAVERINSVLSEVEIDEALANGLEREIQEKENHDEITKLFFVNGYSGKNRYLNAHYMSALKSASNLSTYAWSGDVCLEDVHFSYPLRPDVEI**LNGLNLKLKCGTVTALVGPSGAGKSTIVQLLARFYEPTRGKITVAGEDVRTFDKTEWARVVSIVNQEPVLFSVSVGENIAYGLPDDNVSKDDIIKAAKAANAHEFIISLPQGYDTLVGERGGLLSGGQRQVCNKL**FREIER

>HbABCB29

MKALSLQIQPKTPFPRLPLFHLKPKPLSSPPISRTHNPTLKLPSNTQLKPLNSSNLPSLLPFTQQNPKPNPISHTFHSLSTIRPYVLSQHKLILLGWLCSFVSVLSLTNLVPKFGKFSATIGKVDVVALRNEGLVLAALLLAKLIATYWQHALLWEAALNAGYKIRVHAFERVLHRELG*FFEGGSGVSTGDIAYRITAEAADVADTLYALLNTTVPSALQLSAMATQMWAISPVLSLISATVIPCMALVIAYLGERLRKISKKAHLSIATLSAHLNEVLPAIVFVKANNAELCESARFQRLAYADLSEHLKKKKMKALIPQIIQIIYFGALFTLCCGSLVISRGCFDGCSMVSFVTSLVFLIEPI*QDVGKAYNEWKQGEPAIERLFDLISFKSKVIEKPDAVDLANVTGDIKFCDISFKYGNNRPFV**LNSLNLHIKAGETVALVGPSGGGKTTLVKLLLRLYDPLSGCILVDDQNIQNIRLESLRRHVGLVSQDISLFSGTVAENIGYRDLMTEIDTEKVEAAARIANADEFIRMLPKGYKTYIGPRGSSLSGGQKQRVAIARALYQDSSILILDEATS**ALDSRSELLVRQAVQHLMENHTVLVIAHRLETVMMAKRVFILDGGKLEELTSSNLLGGHSNSGSSTGLVV

>HbABCC2

MAFEPLVWYCRPVANGLWTRAVENAFGAYTPCATDTLVVVISHLVLMALCFYRIWLTKKDFKIQRFCLRSKRYNYFLGLLAGYSTAEPLFRLIMGISTLNIDGQKELAPYEIVSLIIEALAWCSVLVMISVETKVYIREFRWFVRFGVLYTLVGDAVMFNLILAVKEFYNSSVLYLYISEVFVQVLFGILLLVYVPDLDSYPDYTPLRSEYVDDVDYQELPGGEYVCPEQHVNIFSKTIFAWMNPIMKLGYKRPLTEKDIWKLDMWDRTETLNDRFQKCWAEESRRPNPWLLRALNSSLGGR*FWWGGFWKIGNDVSQFVGPLLLNQLLQSMQEGDPAWIGYIYAFSIFAGVVLGVLFEAQYFQNVMRIGYRLRSTLIAAVFRKSLRLTHESRRKFASGKITNLMTTDAEALQQVCQSLHTLWSAPFRIIIAMVLLFQQLGVASLLGAVILVLLFPIQTFVISRMQKLSKEGLQRTDKRIGLMNEILAAMDAVKCYAWENSFQAKVQTVRDDELSWFRKASLLGACNGFILNSIPVVVTVISFGMFTLLGGDLTPARAFTSLSLFAVLRFPL*FMLPNIITQVVNANVSLKRLEELLLAEERILLPNPPLETGQPAISIKNGYFSWDSKAERPT**LSNVNVDIPIGSLVAIVGSTGEGKTSLISAMLGELPAISDASAVIRGTVAYVPQVSWIFNATVRDNILFGSPFDSARYEKAIDVTSLQHDLDLLPGGDLTEIGERGVNISGGQKQRVSMARAVYSNSDVYIFDDPLS**ALDAHVARQVFDKCIKGELSAKTRVLVTNQLHFLSQVDRIMLVHEGMVKEEGTFEELSNNGMLFQKLMENAGKMEEYVEEKENGETFDLKTSSKPIANGAMNDLRKNATETKKRKEGKSILIKQEERETGVVSWNVLMRYKNALGGAWV*VLILFMCYVLTEVLRVSSSTWLSNWTDQGTTKIHGPLYYNLIYSLLSFGQVMVTLLNSYWLIISSLYAARRLHDAMLNSILRAPMVFFHTNPLGRIINRFAKDLGDIDRNVAPFVNMFLGQVSQLLSTFILIGIVSTMSLWAIMPLLVLFYGAYLYYQSTAREVKRLDSISRSPVYAQFGEALNGLSTIRAYKAYDRMADINGRSMDNSIRFTLVNMSANRWLAIRLETLGGIMIWLTATFAVMQNGRAENQQAFASTMGLLLSYALNITGLL*TGVLRLASLAENSLNAVERIGTYIDLPSEAPPIIEGNRPPPGWPSSGSIKFEGVVLRYRPELPPV**LHGLSFMVSPSDKVGIVGRTGAGKSSMLNTLFRIVELERGRILIDGCDIAKFGLMDLRKVLGIIPQSPVLFSGTVRFNLDPFNEHNDADLWEALERAHLKDVIRRNSLGLNAEVSEAGENFSVGQRQLLSLARALLRRSKILVLDEATA**AVDVRTDALIQKTIREEFKSCTMLIIAHRLNTIIDCDRILLLDSGQVLEYDTPEELLSNEDSAFSKMVQSTGAANAQYLRSLVLGGEGESRFRTRENKQLDGQRKWLASSRWAAAAQFAIAVSLTSSHNDLQRLEVADEDSILKKTKDAVVTLQGVLEGKHDKVIDESLNQYQISREGWWSALYKMVEGLAMMSRLGRNRLHQSEGFEDRSIDWDHVEM

>HbABCC5

MGITLLLNNIVTQSTHPVLKAIQGLPVFELASICINLTLFLVFLFIISARQILVCVSRIRLLKDDTPVASSSPIRRSTADGEIRVVTVSTGFKLVLLCCFYVLFLQFLALGFDGVSLIREAVNGKVVDWSIIAFPAAQGLAWFVLSFSALHCKFKASEKFPLLLRVWWLFSFFISLCNLYVDGRSFLVEGAKHLNSHVVVNLAATPAIAFLCFVAVRGITGIQVCRNSDLQEPLLLEEESGCLKVTPYSNAGLFSLATLSWLNPLLSIGAKRPLELKDIPLLAPKDRAKTNYKVLNLNWEKLKAENPSKQPSLAWSILKSFWKEA*ACNAIFALVNTLVSYVGPYMISYFVEYLGGKETFPHEGYILAGIFFSAKLVETLTTRQWYLGVDILGMHVRSALTAMVYRKGLRLSSLAKQSHTSGEIVNYMAVDVQRVGDYSWYLHDIWMLPLQIILALAILYKNVGIASIATLVSTIISIIVTVPLAKIQEDYQDKLMAAKDDRMRKTSECLRNMRILKLQAWEDRYRVKLEEMRDVEFRWLRKALYSQAFITFIFWSSPIFVAVVTFGTSILLGGQLTAGGVLSALATFRILQEPL*RNFPDLVSMMAQTKVSLDRISGFLQEEELQADATLVLPRGMTNMAIEVNDGEFCWDPSSSSSRPT**LSGIHMKVQRGMRVAVCGMVGSGKSSFLSCILGEIPKISGEVRICGTAAYVSQSAWIQSGNIEENILFGSPMDKAKYKNVIHACSLKKDLELFSHGDQTIIGDRGINLSGGQKQRVQLARALYQDADIYLLDDPFS**AVDAHTGSELFKEYILTALASKTVIFVTHQVEFLPTADLILVLKEGRIIQAGKYDDLLQAGTDFKALVSAHHEAIGAMDIPTHSSDDSDESLSLDGSVIFNKKCDATGSNVDILAKEVQESASVSDQKAIKEKKKAKRSRKKQLVQEEERVRGRVNMKVYLSYMAAAYKGLLIPLIILAQALFQFLQIASNWWMAWANPQ*TEGGQSRVSPMVLLGVYMALAFGSSWFIFVRAVLVATFGLAAAQKLFLKMLRSVFRAPMSFFDSTPAGRVLNRVSIDQSVVDLDIPFRLGGFASTTIQLLGIVGVMTKVTWQVLLLVVPMAVACLWMQKYYMASSRELVRIVSIQKSPIIHLFGESIAGAATIRGFGQEKRFMKRNLYLLDCFARPFFCSLAAIEWLCLRMELLSTFVFAFCMILLVSFPHGSIDPSMAGLAVTYGLNLNARL*SRWILSFCKLENKIISIERIYQYSQIPSEAPSVIEGFRPPSSWPENGTIDLIDLKVRYAENLPTV**LHGITCTFPGGKKIGIVGRTGSGKSTLIQALFRLIEPAEGRIIIDNIDISMIGLHDLRSRLSIIPQDPTLLEGTIRGNLDPLEEHSDQEIWQALDKSQLGEIVRRKDQKLDTPVVENGDNWSVGQRQLVSLGRALLKQARILVLDEATA**SVDTATDNLIQKIIRTEFKDCTVCTIAHRIPTVIDSDLVLVLSDGVH

>HbABCC13

MGKFPFLLSHMIAPFATKAMEWKNLICPNSPFVWDGNKISECFDNIVLGFGANVVTVLMISILAITLRNARGSHRMNFREKVVFHFLPALGACLSFVDMVFLLKKELNGDFIVYHEWLFKSSQLILWTTIIISVKWACFHDLFCNWLLCIWWIMKALLEILHLHKTFSSLEALECLKESSVVLLDIMFGITINIIRIKQSSSKASSMEDPLLSVNMDIEGGFPGDSGNTWSSWDLMTFKAITSVMKRGVIKQLGFEDLLWLPNDMEPSTCHDRLLSFWRAQQGSSNPFLFKAICYAYGWP*YFCIGLLKLLNDCIGFAGPLLLNKLIRFLQQGSAHWTGYVLALSLGLTSILKSFLDTQYSFHLAKLKLKLRSGIMTVIYQKCLCVTLAERSKFSEGEIQTFMSVDADRTVNLCNSFHEIWGLPLQIGVALYLLYTQVKFAFLSGLAITILLVPVNKWISELIASATEKMMKQKDERIRRTGEILTHIRTLKMYGWEHLFSSWLMDTRSSEVKHLATRKYLDAWCVFFWATTPTLFSLFTFGLFTLMGHQLEAATVFTCVALFNNLISPL*NSFPWVINGLIDAFISTRRLSRFLCCSEYRHELEQRAESPSVLKNYQSDIISEDMAIIMHDACCAWSSSDEQQQNLV**LNHVTLSVPKGSFIAIIGEVGSGKSSLLSAILGEMWLIHGSVHSNGSLAYVPQVPWILSGTVRDNVLFGKSYESKRYSDTLKACALDVDISLMAGGDMAYIEEKGVNLSGGQRTRLALARAIYQGSDVYMLDDVLS**AVDAEVARLILHNAILGPLMNQKTRVLCTHNVQAISSADMIVVMDKGHVKWVGSSADLSVSSFSAFSPQNDFDILPNLQGQELSKNTSIEGRKSFSLEEEFIHISEEAQEIVEVEQRKEGKVELAVYKNYAAF*CGWFITVVICLSAILMQASRNGNDLWLSYWVDATGSSQADYSTSFYLVVLCIFCIVNSSLTLVRAFSFAFGGLHAAVQVHNTLLNKIIDAPVQFFDQTPAGRILNRFSSDLYTIDDSLPFILNSLLAHFVGLLGIAIVLSYVQVVFLLLLLPFWFIYSKLQFFYRSTSRELRRLDSVSRSPIYATFTETLDGSSTIRAFKSEDCFLVKFIELVALYQRTSYSEIIASLWLSLRLQLLAAFIISFVAMMAVVGSRGYLPISFGTPGLVGLALSYATPIVSSL*GSFLTSFTETEKEMVSVERALQYMDIPQEELRGSQSLNLDWPFQGLIEFQNVTMRYMPSLPPA**LNGVTFTILGGTQVGIVGRTGAGKSSVLNALFRLTPICSGCILVDDLNITHVPVRDLRAHFSVVPQSPFLFEGSLRDNLDPLRMSNDLEIWNILEKCHVKEEVEMAGGLDIHVKQSGSSFSVGQRQLLCLARALLKSSKVLCLDECTA**NVDTQTASVLQNAISTECKGMTVITIAHRISTVMNMDNILVLDHGNVIEQGNPQTLLQDEFSRFSRLAKASTM

>HbABCD1

MPSLQLLQLTEHGRSLLASRRKSLLFAGGILVFGGTAAYVKSRHGCKKFDSIDHYNGLRGDNDKSDKQVTKEAKKII*QKKGSLKSLHVLASVLLSEMGKRGTRDLLAMIAIAVLRTALSNRLAKVQGFLFRAAFLRRVPLFFRLISENILLCFLLSTIHSTSKYVTGTLSLCFRKILTKRIHAHYFENMAYYKISHVDGRITNPEQRIASDVPRFCSELSELVQDDLTAVTDGLLYTWRLCSYASPKYLFWILGYVLGAGTMIRNFSPAFGKLMSKEQQLEGEYRRLHSRLRTHAESIAFYGGERREESHIQQKFKDLVRHMRVVLYDHWWFGMIQDFLLKYLGATVAVVLIIEPFF*AGHLRPDASTLGRATMLSNLRYHTSVIISLFQSPGTLSISSRRLNRLSGYADRIHELIVISRELNCDDKTSLQRSGSRNYFSEADYVEFSGVKVVTPTGNVL**VEDLTLKVESGSNLLITGPNGSGKSSLFRVLGGLWPLVSGHIVKPGVGSDLNKEIFYVPQRPYTAVGTLRDQLIYPLTVDQEVEPLTRSGMVELLKNVDLEYLLDRYPPEQEVNWGEELSLGEQQRLGMARLFYHKPKFAILDECTS**AVTTDMEERFCAKVLAMGTSCITISHRPALVAFHDVVLSLDGEGGWRVSYKRRDSADLKEPGTNDTRASKTERKSDAMLVQRAFATSDKDSTFSNSKSQSYISEVIVACPSADPGLPLPIVPQLQ*RDPRVLALRVAAMFKILVPTLLDKQGAQLLAVAVLVVSRTWVSDRIASLNGTTVKFVLEQDKTSFIRLIGVSILQSAASSFIAPSLRHLTARLALGWRIHLTQHLLSNYLRNNAFYKVFHMSSKNIDADQRITDDLEKLTRDLSGLVTGMVKPLVDILWFTWRMKLLTGQRGVAILYTYMLLGLGFLRTVTPDFGDLASREQQLEGTFRFMHERLCTHAESVAFFGGGAREKAMIESRFSELLDHSLLLLKKKWLYGILDDFVTKQLPHNVTWGLSLLYAM*EHKGDRAQVSTQGELAHALRFLASVVSQSFLAFGDILELHKKFLELSGSINRIFELEELLDTAQSGDWLVDKLSTSMESDSNVKDAISFVEVDIITPAQKLL**ARRLTCDIVRGKSLLVTGPNGSGKSSIFRVLRGLWPIVSGRLAKASQLNNEDSESGCGIFYVPQRPYTCLGTLRDQIVYPLSHDEAALMTLKLHGEDKISGDTTKILDARLKAILENVRLNYLLEREEGGWDANLNWEDILSLGEQQRLGMARLFFHKPKFGILDECTN**ATSVDVEEQLYRLAKDMNITVVTSSQRPALIPFHSVELRLIDGEGNWELRTIRQ

>HbABCD2

MIVETQAHRLVLLSASTCIYTPSSQSHKSCHVQAGLFTPRFPLPISTGLRTWTYRFKLAVTDSSLSAPPTPPPPDKDDAQRKVP*ELQTLFRRFWKVAAPYWFSDDKVQARLQLGAVFALTLATTGISVGFNFLGRDFYNALANKDQEQFTKQLLYYLGGFAGGIPFFVLRDYAREILALRWRSWMTKYYMERYLKNQAFYKIQSQSIIDNPDQRIVDDLSSFTGTALSFSLILFNAAVDLISFSNILYGIYPPLFVVLLLYSVGGTAISVYLGRGLVTLNFLQEKKEADFRYGLVRVRENAESIAFYGGEENEMQLLLQRFRSAFENLTQLLISSRNLEFFTNGYRYLIQILPAAVVAPMYF*SGKIEFGVINQSVSAFNHILGDFSLIVYQFQSISAFSAVIDRLGEFDDVLDSSSSKQLPELSEEISLSYCNYRSSLILEFNGSIPVDSRQKLLSIENLTLQTPTSKATL**IRDLSLVINEKDHLLVMGPSGSGKTSLLRALSGLWNVGRGKITFYLNDGDDPQLPTSSELPANEINTSHENAGELEGPINRNSRGIFFLPQRPYMVLGTLRQQLLYPTWADDKTPMSDGTKPVGSVSFLMGKTNSENVRGKPGKPTTDDLIQVLENVRLGYILSQFGSLDSTYEWSSVLSLGEQQRLAFARLLLSKPKLVLLDESTS**ALDEVNEAHLYRQIEAAGITYVSVGHRRTLYKHHNMVLRISTADLNRNKRNWDIESINPGALYNLSSQ

>HbABCE2

MADRLTRIAIVSSDRCKPKKCRQECKKSCPVVKTGKLCIEVTPASKIAFISEELCIGCGICVKKCPFEAIQIINLPKDLDKDTTHRYGPNTFKLHRLPV**PRPGQVLGLVGTNGIGKSTALKVLAGKLKPNLGRFNNPPDWQEILTYFRGSELQNYFTRILEDNLKAIIKPQYVDHIPKAVQGNVGQVLDQKDEREMKAELCDDLELNQVIDRNVGDLSGGELQRFAIAVVAIQNAEIYMFDEPSS**YLDVKQRLKAAQVIRSLLRPNSYVIVVEHDLSVLDYLSDFICCLYGKPGAYGVVTLPFSVREGINIFLAGFVPTENLRFRDESLTFKVAETPQESAEEIETYARYKYPTMTKTQGNFK**LRVIEGEFTDSQIIVMLGENGTGKTTFIRMLAGLLKPDSVEDSDVEIPEFNVSYKPQKISPKFQSTVRHLLHQKIRDSYTHPQFVSDVMKPLLIEQLMDQEVVNLSGGELQRVALCLCLGKPADIYLIDEPSA**YLDSEQRIVASKVIKRFILHAKKTAFVVEHDFIMATYLADRVIVYEGRPSVDCAANSPQSLLTGMNLFLSHLDITFRRDPTNYRPRINKLDSTKDREQKAAGSYYYLDD

>HbABCF1

MVSDASKKKAAQKKAAAAAKRGGKAAAAAASSKATAAAAAASSADNGSVDNLSNGVGAIQISDRTCTGVLCSHPLSRDIRIESLSVTFHGHDLI**VDSLLELNYGRRYGLLGLNGCGKSTLLTAIGCRELPIPEHMDIYHLTREIEASDMSALQAVISCDEERVKLEKEAEILGTQEDGGGETLERIYERLEAIDASTAEKRAAEILYGLGFNKQMQSKKTRDFSGGWRMRIALARALFMNPTILLLDEPTN**HLDLEACVWLEETLKRFDRILVVVSHSQDFLNGVCTNIIHMQNKKLKIYTGNYDQYVQTRAELEENQMKQYKWEQEQIASMKEYIARFGHGSAKLARQAQSKEKTLAKMERGGLTEKVVRDKVLVFRFVDVGKLPPPVLQFVEVTFGYTPDNLI**YKNLDFGVDLDSRIALVGPNGAGKSTLLKLMTGDLVPTDGMVRRHNHLRIAQFHQHLAEKLDLDMSALQFMIKEYPGNEEERMRAAIGKFGLTGKAQVMPMKNLSDGQRSRVIFAWLAYRQPHLLLLDEPTN**HLDIETIDSLAEALNEWDGGLVLVSHDFRLINQVAEEIWVCENQAVTRWEGDIMDFKEHLKKKAGLSD

>HbABCF3

MTEVASSAVHEVLGRRVQDVDQPIIDYIINVLADDDFDFGEEGEGAFEAIGELLVGAGCVSDFSECRLVCCKLSEKFGKHGLVKAKPTVRSLTTPLRMNDGMDEEVPVKKPEVMDGPVLSERDRAKLERRKRKEERQREAQYQMHLAEMEAVRAGMPVVCVNHDIGSGPTVKDIHMENFSISVGGRDLI**VDGSVTLSFGRHYGLVGRNGTGKTTFLRHMAMHAIDGIPANCQILHVEQEVVGDDTSALQCVLNTDIERTQLLQEEARLLAQQRELEFEGENGDLKGDHNGAIDKDGIAPRLEEIYKRLEFIDAYSAEARAASILAGLSFSPEMQKKATKTFSGGWRMRIALARALFIEPDLLLLDEPTN**HLDLHAVLWLESYLMKWPKTFIVVSHAREFLNTVVTDILHLHAQKLSAYKGNYDTFEKTREEQIKNQQKAFEANERSRAHMQSFIDKFRYNAKRASLVQSRIKALERMGHVDEIVNDPDYKFEFPTPDDRPGPPIISFSDASFGYPGGPML**FKNLNFGIDLDSRIAMVGPNGIGKSTILKLIAGELQPSSGTIFRSAKVRIAVFSQHHVDGLDLSSNPLLYMMRCFPGVPEQKLRAHLGSFGVTGNLALQPMYTLSGGQKSRVAFAKITFRKPHIILLDEPSN**HLDLDAVEALIQGLVLFQGGILMVSHDEHLISGSVEELWVVSQGRVTPFHGTFQDYKKILQSS

>HbABCF4

MGKKKAGEASVGTKANASNKDGKKEKISVAALLANMDQKPDKPKRGSTSSLSTAKARAPKVSSYTDGIDLPPDEEDDYASEDEQQHAGAKRQSSRQQRGEPKLLDISVTDKELKKREKKELLAAYTAEQAKKEALKDDHDAFTVVIGSRASVLEGEDDADANVKDITIDNFSVSARGKEL**LKNASVKISHGKRYGLVGPNGMGKSTLLKLIAWRKIPVPKNIDVLLVEQEVVGDDKTALEAVVAANEELLKIRQEVASLQNATSATGNEDGDDDINGNDAGEKLAELYEKLQILGSDAAEAQASKILAGLGVTREMQGRPTQSFSGGWRMRISLARALFVQPTLLLLDEPTN**HLDLRAVLWLEEYLCRWKKTLVVVSHDRDFLNTVCNEIIHLHDLKLHVYRGNFDDFESGYEQRRKEMNKKFEIYDKQVKAAKRSGNRVQQEKVKDRAKFAAAKEASKSKAKGKVDEDEPLSEAPKKWKDYSVEFHFPEPTELTPPLLQLIEVSFSYPNREDFR**LSNVDVGIDMGTRVAIVGPNGAGKSTLLNLLAGDLVPTEGEVRRSQKLRIGRYSQHFVDLLTMDETPVQYLLRLHPEQEGLSKQEAVRAKLGKFGLPSHNHLTPILKLSGGQKARVVFTSISMLRPHILLLDEPTN**HLDMQSIDALADALDEFTGGVVLVSHDSRLVSCVCEDEERSEIWVVENGTVNAYPGTFEEYKEELQREIKAEVDD

>HbABCF5

MDLSIKFHRLDLHSSFVTGSHLFGASKSLRLPRFRHSSKPIKNDHSSLKIAAPFVCRRGNSKITAQLSTATVETSVAEPETDIESLFSSSSSDEFGRKGAHKHSHTGASGISSGIKLENISKSYKGVTV**LKDVTWEVKKGEKVGLVGVNGAGKTTQLRIITGQEEPDSGNVIKAKSNMKIAFLSQEFEVSLSRTVKEEFMSAFEEEMEIAGRLEKVQKAIEGAVEDLELMGRLLDEFDLLQRRAQAVDLDEVDAKISKLMPELGFAPEDSDRLVASFSSGWQMRMSLGKILLQDPDLLLLDEPTN**HLDLDTIEWLEGYLQKQDVPMVIISHDRAFLDQLCTKIVETEMGVARTYEGNYSQFLVSKAAWIEAQYAAWEKQQKEIEQTRGLISRLGAGANSGRASSAEKKLERLREEDQIEKPFQRKQMKICFPERGRSGRTVVMIKNLEFSYEDKVL**FNRTNLTIERGEKIAIIGPNGCGKSTLLKLIMGLVKQKAGEIVLGEHNVLPNYFEQNQAEALDLDKTVLQTVEEVAEDWRTDDIKGLLGRCNFKADMLDRKVSLLSGGEKARLAFCKFMVKPSTLLVLDEPTN**HLDIPSKEMLEEAITEYKGTIITVSHDRYFIKQIVNRVIEVKDGQLQDYAGDYNYYLEKNLDARARELEREAELEERAPKVKAKSKMSKAEKEARKKQKMQAFQAAKQKSKGLKNSKRWK

>HbABCG3

MEEIQSQSDNYRSSSSSASSPASRVPSSNFFYLRKPGSLRQPISFEDSPEWEDTDVDVRVEEGGDTINVAATPASPCLSKLNSGSLPSPPLPENAVVARKIAGSSVVWKDLTVTIKGKRKYSDKV**VKSSSGYALPGTMTVIMGPAKSGKSTLLRAIAGRLHHSAKMYGEVFVNGTKSHLPYGSYGFVERETTLIGSLTVREHLYYSALLQLPGFFCQKKTVVEDAIHAMSLSDYANKLIGGHCYMKGIPNGERRRVSIARELVMRPHILFIDEPLY**HLDSVSALLMMVTLKKLASTGFTLIFTIYQSSTEVFGLFDRICLLSNGNTLFFGETLACLQHFSNAGFPCPIMQSPSDHFLRAINTDFDRIIAMCKNWQDDHGDFSSVNMDTAVAIRTLEATYKSSADAAAVETMILKLTEKEGPYLKSKGKASSA*TRIAVLTWRSLLIMSREWKYYWLRLIICMLLTLCIGTVFSGLGHSLSSVVMRVAAIFAFISFTSLLSIAGAPSLLKEIKIYASEESNGHSGALVFLLGQLLSSIPFLFLISISSSLVFYFLIGLRDEFSLLMYFVLNFFICLLVNEGLMLLITSLWLHVFWSVLTLVSIHVVMMLSAGYLRIRNALPRPVWTYPISYIAFHTYSIQGLLENEY*LGTSFAVGEVRTISGIQALRSAYDISSNSNSKWENLLVLFLMAIAYRILVLLVLHFRVGKNESVLKFCRCNQDTNNPR

>HbABCG5

MKKQGCEIEARGITFKISTQKRNYPFKIFNEDQQINQELKPNLAEKHL**LNGVNCKAKPSEILAIVGPSGAGKSSLLEVLAGKLTPQNGSILVNQNPVDKAQFKKISGYVTQRDTLFPLLTVEETLMFSAKLRLRLPEEQLISRVKSLVQELGLEHVAMTRVGDERVRGISGGERRRVSIGVDVIHDPKVLILDEPTS**GLDSTSALQIISMLKVMAETRGRTIILSIHQPGFRIVKLFNSILLMANGSILHHGTVDQLGVHLRTMGMQLPLHVNVVEFAIESIEAIQQQQPESTPVWTAQQQIKKAEEGDSRSGKFTLQQLFQQSKVVDEEIINAGIDFPRGFANSRF*QETVILTHRFSKNIFRTKELFACRTIQMLISGLVLGSIFSNVKDDLTGAEERVGLFAFTLTFLLSCTTEALPIFLQEREILMKETSCGSYRVSSYAVANGLVYLPFLLILAILFSIPLYWLVGLNPNFTAFIHFLLLIWLILYTANSVVVCISALVPNFIVGNSVISGVMGSFFLFSGYFISKHGIPNYWMFMHYISLFKYPFEGFLINEF*SRSGKCLEYMFGTCMVRGEDVLKEEGYGEESRWRNVVVMVCFIFVYRFISYAILRCRCSVTSLKASLV

>HbABCG7

MVGQVVKFGGNGFGQVLAAAAVAILVRLFSGPGPALLPEDEFADDERNGVPGDDKAGEASVNGKVVPVTIRWNNITCSFSDKSSKQVRFL**LKNVSGEAKPGRLLAIMGPSGSGKTTLLNVLARQLMASPRLHLSGLLEVNGIPISNIAYKFAYVRQEDLLFSQLTVRETLSLAAELQLPEISSVEERDEFVNNLLFKLGLVSCADSIVGDAKVRGISGGEKKRLSLACELIASPSVIFADEPTT**GLDAFQAERVMETLRQLAQDGHTVICSIHQPRGSVYSKFDDIFLLTEGALVYAGPAHDEPLAYFTKFGYRCSDHVNPAEFLADLISVDYSSAESVYSSRKRIDGLVESFSEQLSTVLYATPFASRESPKNGKKLSKKTVVKRKETWW*RQFWLLLKRAWMQASRDGPTNKVRARMSIASAIIFGSVFWRMGRSQTSIQDRMGLLQVAAINTAMAALTKTVGVFPKERAIVDRERAKGSYALGPYLLSKLIAEIPVGAAFPLMFGAVLYPMARLHPTLSRFGKFCGIVTAESFAASAMGLTVGAMAPTTEAAMALGPSLMTVFIVFGGYYVNADNTPIIFRWIPNVSLIRWAFQGLCINEF*SGLKFDHQHSFDIETGEQALERLSFGGSHINDTVIAQSRILLFWYCTTYLILQKNKPKYQQLESLPLEQIQPQLQLEPVEPDKVKQLNPPMKQVELNQQLESPALDQIRPFILEGAK

>HbABCG11

MTSLEMETSDINSVQTHTPKEDVDDAVFLSWKDLWVTVRDGRHGSRSI**LQGLTAYAQPGELLAIMGPSGCGKSTLLDALAGRLNSNTIQAGEVLINGHKQALAYGTTAYVTQDNNLVATLTVREAVYYSAQLQLPDSMSNSEKKERAERTIREMGLQDARNTRIGGWGAKGLSGGQKRRVSICIEILTHPKLLFLDEPTS**GLDSAASYYIMSRIASLGRNDGIRRTIIFSIHQPSSEVFQLFNNLCLLSSGKMVYFGPASAANEFFTLNGFPCPTYQNHSDHFLKTINKDFERDLEQGMSDATPTEEVINTLIQSYTSSETYQEVRKKVAEINKEDYGAILEKKRSHASFL*NQFLVLTRRSFVNMYRDIGYYRLRLFVYVGLAFGLATIYYDLGSSYGSIQARGSLLMFISTFLTFMAIGGFPSFVEEMKVFVRERLNGHYGTTAFIFANTFSSKPFLLVISLIPGAIAYYLTGLQKGFDHFLCFASIIFASMILVESVMMVVASIVPNFLMGIIAGAGIQGLMILGGGFFRLPNDLPKPFWKYPLYYIAFHKYAYQGMFKNEF*EGLKLQSNQAAGGIPRMINGEEILRDVWQVEMGYSKWVDVVILLGMAIFYRFLFLIIIKTSETIKPVIIAATEVPPNETIHGEAL

>HbABCG15

MEIEQVISCHGDGGGSSSCGDAEAAAGLPRGGEEVIVGRSMYLVWEDLTVLLPNFSEGPTRRL**LNGLNGYAEPGKIMAIMGPSGSGKSTLLDALAGRLSGNVIMTGNVLVNGKKRKLGYGGVAYVTQENTLLGTLTVRETLTYSAHLRLPGSMTREEIEGIVQGTIMEMGLQDCADRLIGNWHLRGISGGEKKRLSIALEILIRPQLLFLDEPTS**GLDSASAFFVIQTLKNIAHDGRTVISSIHQPSSEVFALFDDLFLLSNGEVVYFGEAKMAVEFFAEAGFPCPSRRNPSDHFLRCINSDFDHVTATLMGSQRSSPRHQEIQISSDSLANLPTAEIKAVLVKKYRFSNYAARAKARIREISATKGIEAKRKRESQANWS*KQLSILTQRSFTNMWRDLGYYWVRIGIYIALSICVGTIFFDVGRSYTAILARGACGGFISGFMTFMSIGGFPSFIEELKVFYKERLNGHYGVGIYILSNFLSSFPYLTVMSVATVSITFYMVKFRPEFSHFVYACLDLTSSIAAVESCMMTIASLVPNFLMGVIVGAGYIGILMMTSGFFRLLPELPKVFWRYPVSYINYGAWGLQGAYKNDM*IGIEFDPLVPGNPKLKGEFVLSTMLGIDLHHSKWWDLVAVVVILIAFRLLFYAILKFKERTMPVFHKLYAQRTIKHLKKRPSFRNSSPFPSFRKSSPFPSTRHQTLHSLSSQEGLNSPIH

>HbABCG20

MELQKYHRTSEPSVSITLSELIKRVEDAQSDHSNGSTPIRHHALELGYACSSVSPSNPFVLSFNNLSYSVKVGQKLTFPFCGNDSDDSPETGIKVL**LNDISGEAREGEIMAVLGASGSGKSTLIDALADRISKESLKGSVSLNGEVLESRLLKVISAYVMQDDLLFPMLTIEETLMFSAEFRLPRSLSKSKKKARVEALIDQLGLRSAAKTVIGDEGHRGVSGGERRRVSIGIDIVHDPILLFLDEPTS**GLDSTSAFMVVKVLQRIARSGSIVIMSIHQPSYRILSLLDRLIFLSHGQTVYAGPPGSLPEFFAEFGHPIPENENRTEFALDLIRELEEIPDGTRTLVEFNRSWQAQGKKNPRNRISNSSNLSLKDAISASISKGKLVSGATNDSNLSSSVPTFANPFWI*EMLVIAKRSLTNSRRMPELFGIRFGAVFITGVILATIFWHLDNSPRGAQERLGFFAFAMSTTYYTCAESIPAFLQERYIFMRETAYNAYRRSSYVLAHSLISIPSLIILSIAFAATTYWTVGLAGGASGFFFFFFTILSAFWAGSSFVTFLSGIVSHVMLGFTIVVAILAYFLLFSGFFISRDRIPPYWIWFHYLSLVKYPYEGALQNEF*QDPTKCFVRGVQMFDNTPLSAVPVALKLKLLQSLSNTLGRNVTGSTCIVTGPDILRGQGITDISKWSCLWITIAWGFFFRVLFYFTLLLGSKNKRR

>HbABCG21

MMPPEQESSIAAGNSPANIMLPNWTETVPVHAEPSVSSINATPCSQDRLPDQQEPTPSRFSILRASLRPVTLMFVDVAYSIDLSTKGSCCSPNGSKSTRIV**LNGVSGIVRPGELVAMLGPSGSGKTTLLTALAGRLPGKVSGTITYNGLPFSSSVKRKTGFVAQDDVFYPHLTVIETLTFAALLRLPKMLTREEKIEQAEMVIVELGLTRCRNSVVGGPLLRGISGGERKRVSIGQEMLVNPSLLLLDEPTS**GLDSTTAQRIMATLKGLARGGRTLITTIHQPSSRLYMMFDKVVVLSDGCPIYSGPAGRVMECFDSIGYVPGFNFMNPADFLLDLASGIVPDTKQDDKMGVYGRVDHLDDQNSTKQSLISSYKKNLYPALKAEIHQNLQDPALSASSGTSSLRNSEDQWTTTWW*QQFKVLLRRGLQERKHESYSGLRIFQVMSVSILSGLLWLHSDTSHIQDQVGLLFFLSIFWGFFPMFNAIFTFPQERPMLIKERSSGMYRLSSYYFARMAGDLPMELVLPTIFVIFTYWMGGLKSSVITFVLTLLIILFNVLVSQGLGLALGAILMEAKQATTLASVTMLVFLLAGGYYIQHIPPFIAWLKYISFSHFCYKLLVGVQY*SANEVYECELGMDCRVMDFPAIKCLGTDNKGWDVAALSIMLVGYRLLAYVALRMWQPH

>HbABCG22

MEKTSASSLVRTKSDQLVETVAADFKSPPTNEVGGGVLEGSGTLSRKSSKRQIMAASPGRSGGGGKNTHIRKSWSAQMKFDLDDVSSGAALSRASSASLGLSFSFTGFTVPPDEIADSKSFSDDDIPEDLEAGTRKPKFQTEPTLPIFLKFTDVTYKLIIKGMTSTEEKYI**LNGITGSVNPGQVLALMGPSGSGKTTLLNLLGGRLIQPGVGGSITYNDQPYSKFLKSRIGFVTQDDVLFPHLTVKETLTYAALLRLPKTLTKEQKEKRAIDVIYELGLERCQDTMIGGSFVRGVSGGERKRVCIGNEIIINPSVLFLDEPTS**GLDSTTALRIVQMLQDIAEAGKTVITTIHQPSSRLFHKFDKLILLGKGSLLYFGKASEAMPYFSSVGCNPLIAMNPAEFLLELANGNINDVSVPSELEDRVQMENSDNETRNGKPSPAVVHDYLVEAYETRVAENEKKKLMVPIPLDEEVKLKVSSPKRQWGASWW*EQYTLLFCRGIKERRHDYFSWLRITQVLSTAIILGLLWWQSESSSPKGLQDQAGLLFFIAVFWGFFPVFTAIFTFPQERAMLNKERAADMYRLSAYFLARTTSDLPLDLLLPVLFLVVVYFMAGLRMSAGPFFLSLLTVFLCIVAAQGLGLAIGATLMDLKKATTLASVTVMTFMLAGGYFVKKVPIFVAWIRYMSFNYHTYKLLLKVQY*EHMSPPIKGMRMDNGLMEVSALVAMVFGYRLLAYISLRKMKLN

>HbABCG28

MLLYVVSPVYHLVYYFIASYIPRIKLECCLIDVYVCVFSGRSLIIFLLVPSLLFSEACPLGSYCPLAKLNNTTGVCEPYHYQLPPGQPNHTCGGANIWADVGSSSEIFCSAGSYCPTTVQKNSCSSGHYCRMGSTSEKRCFKLTSCKANSTSQNIHAYGIMLMVALTTVLLIIYNCSDQVITTRERRLAKSREAGARSARETARARQRWKVAKDAAKKHASGLQTHLSRTFSRKKYGKYPEQFKILNQDKSEMEVDLYPPSHSSNFSISTSLPSSAPSKGKKKEPIDLMQMMHEIEVDPDGYEGINLEVADPNPTRHMPEGEQMTTHTQIFKYAYAQLEKEKAMELENKNLTFSGVVNMAINTEIKRRPLIEISFKELTLTLKAKNKHL**LRGVTGKIKPGRITAVMGPSGAGKTTFLSALAGKPIGCRMTGLILINGKNQSIHSYKKIIGFVPQDDIVHGNLTVEENLWFSAHCRLSADLPKPDKVLIVERVIESLGLQTVRDSLVGTVEKRGISGGQKKRVNVGLEMVMEPSLLILDEPTS**GLDSASSQLLLKALRREALEGVNICMVVHQPSYTLFKMFDDLVLLAKGGLIVYHGPAKKVEEYFAGLGINVPERVNPPDHYIDILEGIVTPSPSSGVNYKELPIRWMLHNGYPIPPDMQRYAAGLASPVDINPAHESNLGGFGMEEQSFAGELWQDMKSHVELHRDKIRHNFLKSRDLSNRRTPGVFWQYRYFLGRVGKQRLREAKIQAIDYLILLLAGVCLGSLAKVNDQTFGAAGYTYTIIAVSLLCKIAALRSFSLDKLQYWRESASGISSLAYFLAKDTIDHFNTVIKPVLYLSMFYSFTNPRSSFTDNYVVLLCLIYCVTGIAYALAIFFEPGPAQLWSVILPVVLTLIATQANQSNTLKNIANLCYPKWALEAFVIANAERYYGVWLITRCGSLLKTGYNLHHWGLCVFILILIGMVTRFVAFFGMVTFKKK

>HbABCG40

MEGDHYRASTSLRRGSSSVFRNNGLDVFSRSSRDEDDEEALKWAALEKLPTYDRLRKGILVSVSKGGANEIDVDNLGFQERKTLLERLVKVAEEDNEKFLLKLKNRIDRVGIEIPTIEVRYEHLNIEAEALVGSNALPSFLNFTMSIAEVFLNCLHVLPNRKRPLTI**LKDVSGVIKPSRMTLLLGPPSSGKTTLLLALAGKLDPNLKVSGNVTYNAHTLNEFIPQRTAAYISQHDLHIGEMTVRETLAFSARCQGVGTQHEMLAELSRREKAANIKPDPDLDVFMKAAATEGQETSVVTDYVLKILGLDICADTLVGNEMIRGISGGQRKRVTTGEMLVGPAKALFMDEIST**GLDSSTTFSIINSLRQSIHILNGTAVISLLQPAPETYNLFDDIILLSDGQVVYQGPREQVLGFFEYMGFRCPERKGVADFLQEVTSKKDQQQYWARKDQPYRFVTVNEFAEAFQSYEVGRRIAEDLSVPFDRTKNHPAALTTKHYGVGKM*ELLKANLSREYLLMKRNSFVYIFKLTQLIVMATIGMTLFFRTEMKRDTLEDAGVYLGALFFSLITIMFNGMAELSMTIAKLPVFYKQRNLLFFPAWSYSIPSWILKIPVTFVEVGVWVFITYYVIGFDPNVGRLFKQYMLLLLVNQMASALFRFIASVGRNMIVANTFGSFALLTLFALGGFILKRPEIKKWWIWGYWISPLMYGQNAIVANEF*LGHSWNHIPANSNSTESLGVQFIKNNGFFPHAYWYWLGLGASAGYIFVFNILYTVALTFLDQFEKPQAIISDEPEERERSGGAIQLSQVESSHRNNTESGTSGIDESNHNKKKGMVLPFEPHSITFDNVIYSVDMPQEMKNQGVVEDKLVL**LKGVSGAFRPGVLTALMGVSGAGKTTLMDVLAGRKTGGYIEGDIRISGYPKKQETFARISGYCEQNDIHSPHVTVYESLVYSAWLRLPSDVNSETRKMFVEEVMELVELNPLRQALVGLPGVNGLSTEQRKRLTIAVELVANPSIIFMDEPTS**GLDARAAAIVMRTVRNTVDTGRTVVCTIHQPSIDIFEAFDELFLMKRGGEEIYVGPLGRHSCHLIKYFEGIEGVSKITDGYNPATWMLEVSSSAQELSLGVDFANIYRNSDLYRRNKAMIQELSKPAPGTKDLYFPTQYSQPFL*TQCMACLWKQSWSYWRNPPYTGVRFWFTTFIALMFGTIFWDLGSKLEKEGDLSNAMGSMYVAVLFLGVQNSSSVQPVVAIERTVFYREKAAGMYSAMPYAYAQALIELPYIFTQAGVYSLITYAMIGFEWTAAKFFWYLFFLYFTLLYFTYYGMMAVAVTPNHHIASIISSAFYAIWNLFAGFIVPRPKMPVWWRWYYWGCPISWTLYGLFASQF*GDITKPLGETGKTVEQYVSDFYGIKHNFLGACAGVIIGIDTLFAVIFAISIKAFNFQRR

>HbABCI1

MSLRKPPLPRILLDNVSCMRNAQQI**LRHVNVSIHDGGALVLTGSNGSGKTTFLRMLAGFSKPSAGQILWNGHDVTESGVFHQYKLHLNWLSLKDAIKEKFTVLDNVQWFEILEGKQGNSLPALELMGLGRLAKEKARMLSMGQRKRLQLARLVAIDRPIWLLDEPSV**ALDDEGVKLLECIIAEHRKKGGIVFVATHLPIEIEDAMYLRLPPRFPRRMTLVDMLDRADIS

>HbABCI6

MASLHLNFPSSSPPSPLLRSPLPSKLKTSLLYLPLFSPTASRSLSLRFRSNHRRPGRPFHLVSAALSAVDSPETTSSDGSDSEKKPLLEVKNLTAVIAETNQEI**LKGVNLVVHEGEVHAVMGKNGSGKSTFSKVLVGHPDYEVTGGSVMFKGENLLDMEPEERSLAGLFMSFQSPVEIPGVNNIDFLNMAYNARRRKLALPELGPIEFYAYLFPKLELVNMKSDFLNRNVNEGFSGGERKRNEILQLAVLGADLAMLDEIDS**GLDIDALKDVAKAVNGILTPKNSVLMITHYLRLLEFIQPTYIHIMEDGRIVKTGDISIAKVLEKEGYKAISPS

>HbABCI7

MSASVFAPQIHTSTTITSRLKPKPKHRIKTSVSLIRTPVIQASFSDPFVLQLAETFEDSLSPSPSPSLQKLRDSSAESLLSIPWPSRKDEPFRFTDTSFIKQSQIHPISKPPSSYHLTNISNDSHLPNIVIVDGFILNSLSNSSNLPHGVYVGSLLNDPKDKIAQTLTKFSDDFQWGDLFWSINGLGAPDVAVVYVPAGVRVENPIHLSYVSVEGGEEGSNKLPVSNPRVLVVVEEGGEVGIIEEFTNVGSNDKCYWANSVLEVMIEEGAKVRHSYIQSQSLNSAHIKWTSVQQKSTSTYELVEVSTGGKLSRHNLHLQQLGPDTSTELSTFHLSLGGQTQDLHSRLVLDHPRAYSRQLHKCIVAHSQGQAVFDGNVKVNRYAQQTDAGQLTRSLLLEPRATVNVKPNLQIIADDVKCSHGAAISDLEESQLFYFQARGIDLEMARKALVFSFGAEVIERCPYSFVRKQVENHVKELLSSTSQGSSNAAGSG

>HbABCI8

MASLLANGMSSFSPQSFSDSAKLSKPFSFPLKPAFPKTPRPANLFKIRADVGFESKTINTNSGNSASTSTTSSDEKIREILRNRDYDKKFGFNVDIDSFSIPKGLSKETIRLISSLKEEPDWMLEFRLNAFEKFLKMKEPKWSDNMYPPIDFQDICYYSAPKKKPTLNSLDEADPELLMYFDRLGVPLNERNRLANVAVDAVLDSVSIATTHRQTLEKAGVIFCSISEAIKKYPDLVRKYLGRVVPSEDNYYAALNSAVFSDGSFCYIPKDTKCPMQISTYFRINAMETGQFERTLIVADDRSFVEYLEGCTAPSYDTNQLHAAVVELYCAEGAEIKYSTVQNWYAGDEEGKGGIYNFVTKRGLCAGDRSKISWTQVETGSAITWKYPSVVLEGDDTVGEFYSVALTNNYQQADTGTKMIHKGKNTRSRIISKGISAGNSRNCYRGLVQIQSKADNARNSSQCDSMLIGDTAAANTYPYIQVKNPTARVEHEASTSKIGEDQLFYFQQRGIDYEKAMAAMISGFCQDVFNELPDEFGAEVNQLMSLKLEGSVG

>HbABCI10

MDLSTVFGVSTRIPCLHSSPRARSNITENVAIEGRNLSFSFATRQGKFFPI**LKDCSLRVPSGQLWMLLGPNGCGKSTLLKILAGLLQPTSGTLYVKRPKSFVFQNPDHQVVMPTVEADVAFGLGKFILTEDEVRQRVSKALDDVGMSTYMQRPVQTLSGGQKQRVAIAGALAEACKVLLLDELTT**FLDQNDQIGVIKAVKNSLVASDEITALWVTHRLEELEYADGAFYMENGRVVKVGDGSSIMDFINARQASYFNQTNS

>HbABCI11

MATSTSSVFFVINQRPIQSPAPFSPKSCYRKLSFKRLHQSHRVSCDYSCVEVRGVSYRPPGTQLNL**LNEVSFSLPEKSFGLIFGQSGSGKTTLLQLLAGLSEPTSGSIGIQRYGNDGNPCHSPEPLPPEKVGIVFQFPERYFVADTILNEVIFGWPRLKGSLQLKERLALNLQKAITWVGLNGISLEKDPHSLSGGYKRRLALAIQLVQVPDLLILDEPLA**GLDWKARADVINLLKQLKKELTVLVVSHDLKELAALVDHSWRMEMGGYLKEELLPI

>HbABCI13

MVSLSGSILFPATVLHGCTRFSIWSTDTISSFCYKQRKVVCACIAPPRNMGNNEYPATKFTNLYNSEQFGAVLEPEDDSDILIECRNVYKSFGEKDI**LRGVSFKIRHGEAVGIIGPSGTGKSTILKIIAGLLAPDKGEVFIRGRRRDGLISDQDMSGLRIGLVFQSAALFDSLTVRENVGFLLYENSTMGEEQILEIVKETLASVGLKGVEDRTPSELSGGMKKRVALARSIIFDITKKSIEPEVLLYDEPTA**GLDPIASTVVEDLIRSVHMIGEDALGKPGKIASYVVVTHQHSTIRRAVDRLLFLYKGKIVWQGMTHEFTTSSNPIVQQFASGSLDGPIRY

>HbABCI14

MDIKTLNSSQLCFSVGAHTHKFLSPKRQTKLFVAPNTNDGHPSVSLLDEETNTNHAPSSEKETFLSKWSPPGYLWRGLSVLVLAGQVVVRTLKGKIHW*RNTLQQLERVGPRSVGVCLLTSAFVGMAFTIQFVREFTRLGLQRSVGGVLALAFSRELSPVVTSIVVAGRIGSAFAAELGTMQVSEQTDTLRVLGTNPVDYLVTPRVIASCFALPFLTLMCFTVGMASSALLADGVFGISINIILDSARRILRSWDLISAMIKSQVFGAIISIVSCAWGVTTSGGAKGVGESTTSAVVISLVGIFIADFALSYCF*FQGVGDSLKNAM

>HbABCI15

MVGNSLAPVSTCPVALSSSLIIVPPGSSSFLPRLPLRQRKRLMRVRAMSADAGHGQAASSSSSSEKKNPLAVVLEVPQSIWRQTLKPLSDFGFGRRSIWEGGVGLFLVSGAVLLALSLAWLRGFQLRSKFRKYVAVFEFAQACGICTGTPVRIRGVTVGNVIQVNPSLRSIEAVVEVEDDKIIIPRNSLIEVNQSGLLMETLIDITPKDPIPSPSVGPLDGECVKEGLIVCDRQKIKGHQGVSLDALVGIFTRLGREMEEIGVSKSYSLAERVAAVIEEAKPLLTKIQEMAEDVQPLLSEVRDKGLLKEVENLTRSLTQASEDLRKAHTSIMTPENTELIQKSVYSLIFTLKNLENISSDILGFTGDEATRKNLKALIKSLSRML

>HbABCI17

MSSASPLLTSPSPMGSFFRAQASPADDRQEHLLAVGDIEAGGDDPQNKFRIRDLTKKSDAGVTI**LSGVNLDIPKGVVVGIIGPSGSGKSTLLRSMNRLWEPPSGTVFLDGHDIRDLDVLSLRRKVGMLFQIPALFEGTVADNIRFGPQLRGQKLPDHEVHKLLALADLDSSFHKKNGNELSVGQAQRVALARTLANEPEVLLLDEPTS**ALDPISTQNIEDVIVKLKKTQGMTIVMVSHSIKQIQRIADVVCLLVNGEVVEVLKPDELSQAKHPMAQRFLQLSS

>HbABCI18

MSFEEDEE**SMEHTLLVVREVSVYKIPPRSTSGGYKCGDWLQSDKIWSGRLRVVSCKDRCEIRLEDPNSGELFAACFVNPGQRETSVETVLDSSRYFVLKIEDGIGKHAFIGLGFAERNEAFDFNVALSDHEKYVRREHEKQIGETSESDTHIDIHPAVNHRLKEGETIRINVKH**KPSSGTGMLSAAGLSGDHSGTGKPKTLGIAPPPSGAGKLRSPLPPPPNDPAAARITAANHSVYIKAPKENTKRSTDPLSDLSPLERSLPSATSGSTKTTASGWAAF

>HbABCI19

MAEKAWKSRESEEDENDKQNSISVRGMQFAYEGQPPL**FYDFNLNIPPGSRCLLVGANGSGKTTLLKILAGKHMVGGRDVVRVLNLSAFHDTQLVC**GGDLAYLGGSWTKTVGSAGEIPLQGDFSAEHMIFGVEGIDPARREKLIDLLDIDLQWRMHKVSDGQRRRVQICMGLLVPFKVLLLDEVTVDLDVVARLDLLEFFKEECEQRGATLVYATHIFDGLETWATHLAYIQDGELRRSEKLTEVHELKSSANLLSVVESWLRYETRHEKKKPTNPPAQNQRTSPFGTSPFMSSRHMAYYR

>HbABCI20

MGFVEHQEPTIEINDLKFTYPGIDGHPPPGSKPL**IDDFSLTLKTGDRCLLVGSNGAGKTTILKILGGKHMVEPQMVRVLGRSAFHDTALTSSGDLCYLGGEWRRDVAFAGFEVPIQMDVSAEKMIFGVAGIDPQRRAELIKVLDIDLSWRMHKVSDGQRRRVQICMGLLRPFKVLLLDEITV**DLDVLARADLLKFLRKECEERGATIIYATHIFDGLEEWPTHIVYVAHGKLQLAMPMAKVKEISNLSLMRTVESWLRKERDEERKRRKERKASGLPEFEKQMEGSRVTGDPASAAVRAMNNGWAAGRLHSTIAGEENFFLSSNRVLRQ
