# Supplementary material for: Whole-Transcriptome Survey of the Putative ATP-Binding Cassette (ABC) Transporter Family Genes in the Latex-Producing Laticifers of Hevea brasiliensis
Source: PLoS One. 2015 Jan 23;10(1):e0116857. doi: 10.1371/journal.pone.0116857 (PMC4304824; doi:10.1371/journal.pone.0116857)
Supplement: S5 Table — (DOC) [file pone.0116857.s006.doc]

Table S5. Gene expression analyses of the *H. brasiliensis* latex ABC transporters by the RT-qPCR reactions *

| Genes | ET |  | Me-JA |  | Tapping | Genes | ET |  | Me-JA |  | Tapping |
| --- | --- | --- | --- | --- | --- | --- | --- | --- | --- | --- | --- |
|  | Ratio(ET:CK) |  | Ratio(JA:CK) |  | Ratio(T7:T1) |  | Ratio(ET:CK) |  | Ratio(JA:CK) |  | Ratio(T7:T1) |
| *HbABCA1* | 1.41 |  | 0.47* |  | 0.81 | *HbABCG3* | 1.77* |  | 1.68 |  | 1.11 |
| *HbABCA2* | 3.12** |  | 0.77 |  | 2.21* | *HbABCG5* | 2.12* |  | 0.89 |  | 1.87** |
| *HbABCA7* | 0.57 |  | 0.19* |  | 0.19* | *HbABCG7* | 0.75 |  | 1.23 |  | 0.63 |
| *HbABCB1* | 0.41* |  | 0.15** |  | 0.53* | *HbABCG11* | 5.94* |  | 4.13* |  | 8.00* |
| *HbABCB11* | 0.97 |  | 0.75 |  | 1.13 | *HbABCG15* | 0.74 |  | 0.32* |  | 0.75 |
| *HbABCB13* | 4.31** |  | 0.89 |  | 1.77* | *HbABCG20* | 10.34* |  | 3.54 |  | 4.70* |
| *HbABCB15* | 2.39* |  | 2.02** |  | 8.32** | *HbABCG21* | 3.36** |  | 2.08** |  | 2.28* |
| *HbABCB19* | 19.67** |  | 4.91* |  | 16.56** | *HbABCG22* | 1.33* |  | 1.28 |  | 0.78* |
| *HbABCB20* | 0.60 |  | 0.18** |  | 0.17* | *HbABCG28* | 0.58* |  | 1.37 |  | 1.86* |
| *HbABCB25* | 0.62* |  | 0.32* |  | 0.45* | *HbABCG40* | 0.91 |  | 0.18* |  | 2.05* |
| *HbABCB26* | 1.43* |  | 0.55 |  | 1.66* | *HbABCI1* | 3.71* |  | 1.42 |  | 5.31* |
| *HbABCB28* | 3.02** |  | 1.14 |  | 1.48* | *HbABCI6* | 1.88 |  | 1.34 |  | 2.45* |
| *HbABCB29* | 1.31 |  | 0.98 |  | 1.42* | *HbABCI7* | 1.75 |  | 1.02 |  | 4.00* |
| *HbABCC2* | 5.50* |  | 1.03 |  | 0.25* | *HbABCI8* | 0.03** |  | 0.12** |  | 0.27* |
| *HbABCC5* | 3.63** |  | 2.07* |  | 3.89** | *HbABCI10* | 0.33* |  | 1.38 |  | 5.43** |
| *HbABCC13* | 4.11* |  | 2.73 |  | 2.51* | *HbABCI11* | 1.47* |  | 1.71* |  | 1.31 |
| *HbABCD1* | 2.95* |  | 4.10** |  | 3.43** | *HbABCI13* | 3.50 |  | 2.00* |  | 9.47** |
| *HbABCD2* | 1.12 |  | 0.14* |  | 3.34* | *HbABCI14* | 0.87 |  | 1.12 |  | 0.77 |
| *HbABCE2* | 1.03 |  | 0.03** |  | 2.02* | *HbABCI15* | 0.98 |  | 0.85 |  | 1.12 |
| *HbABCF1* | 1.67* |  | 1.88** |  | 1.71* | *HbABC*17 | 0.67* |  | 0.79 |  | 0.84 |
| *HbABCF3* | 1.30 |  | 0.66 |  | 0.26* | *HbABCI18* | 1.27 |  | 1.59 |  | 1.13 |
| *HbABCF4* | 1.52* |  | 0.68 |  | 0.21** | *HbABCI19* | 3.00* |  | 3.42* |  | 5.00* |
| *HbABCF5* | 2.75* |  | 1.50 |  | 0.51* | *HbABCI20* | 2.61* |  | 0.70 |  | 3.67* |

* Mature, virgin rubber trees were tapped sequentially for seven times in a S/2 d/3 system (a half spiral every three days) or firstly stimulated with Ethrel (ET) or Me-JA for 0.5, 1.5, 4.0, 8.0, and 24.0 h, respectively, and untreated trees were left as control samples (CK). Fresh latex from each tapping or time point of treatment was collected and used for isolation of total RNA. The relative abundance of transcripts was determined by RT-qPCR and calculated with the mean value of the triplicate. Ratios were calculated using the relative transcript abundances between (1) rubber trees that were untreated or stimulated for 24.0 h, (2) rubber trees that were tapped for the first time (T1) and the seventh time (T7). The statistical significance of the values was determined by the *t*-test. The *P* value <0.05 and <0.01 were considered to be significant and very significant in the cases, and * indicates significant at 0.05 level contrast to control, while ** indicates significant at 0.01 level contrast to the control.
